# Enantioselective synthesis of bicyclo[3.n.1]alkanes by chiral phosphoric acid-catalyzed desymmetrizing Michael cyclizations†

**DOI:** 10.1039/c5sc00753d

**Published:** 2015-04-30

**Authors:** Alan R. Burns, Amaël G. E. Madec, Darryl W. Low, Iain D. Roy, Hon Wai Lam

**Affiliations:** a EaStCHEM, School of Chemistry, University of Edinburgh Joseph Black Building, The King's Buildings, David Brewster Road Edinburgh EH9 3FJ UK.; b School of Chemistry, University of Nottingham, University Park Nottingham NG7 2RD UK hon.lam@nottingham.ac.uk

## Abstract

2,2-Disubstituted cyclic 1,3-diketones containing a tethered electron-deficient alkene undergo chiral phosphoric acid-catalyzed desymmetrizing Michael cyclizations to give bridged bicyclic products in high enantioselectivities. Both bicyclo[3.2.1]octanes and bicyclo[3.3.1]nonanes are accessible using this methodology.

## Abstract 2

2,2-Disubstituted cyclic 1,3-diketones containing a tethered electron-deficient alkene undergo chiral phosphoric acid-catalyzed desymmetrizing Michael cyclizations to give bridged bicyclic products in high enantioselectivities.

## Graphical abstract

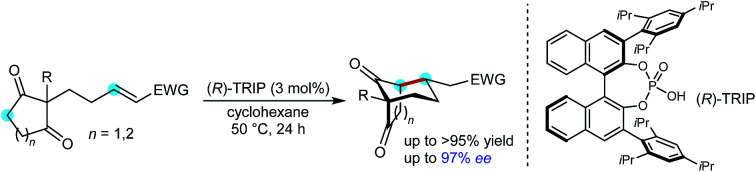

Bicyclo[3.n.1]alkanes appear in numerous biologically active natural products (selected examples are shown in Fig. 1) and have widespread applications in organic synthesis.^1,2^ Although many creative approaches for the synthesis of these structures have been devised,^1,2^ the preparation of enantiomerically enriched chiral bicyclo[3.n.1]alkanes by asymmetric catalysis currently represents only a small fraction of these methods.^3,4^ In view of the present level of development, increasing the number of available catalytic enantioselective reactions to access these compounds is an important area of research. Herein, we report a new approach for the enantioselective synthesis of bicyclo[3.2.1]octanes and bicyclo[3.3.1]nonanes by chiral phosphoric acid-catalyzed Michael cyclizations of 1,3-diones onto tethered electron-deficient alkenes. These reactions give products containing three new stereogenic centers, including an all-carbon quaternary center, resulting from the formal enantioselective, desymmetrizing enolization of 2,2-disubstituted cyclic 1,3-diketones. In addition, these reactions further demonstrate the ability of chiral phosphoric acids to promote transformations of unactivated ketones by enolization, which has so far been relatively underexplored.^5^1
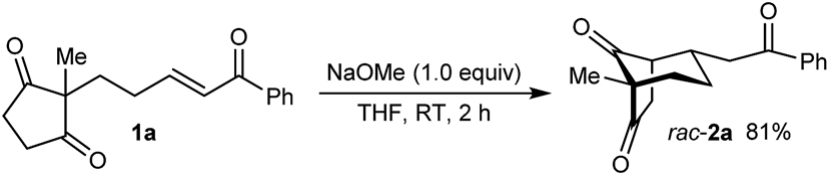


**Fig. 1:**
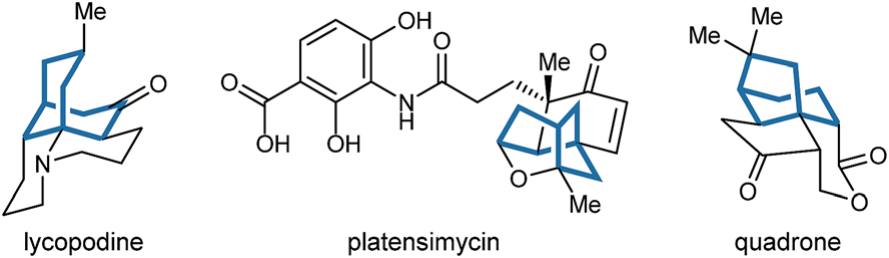
Natural products containing bicyclo[3.n.1]alkanes.

As part of our interest in the catalytic enantioselective desymmetrization of 2,2-disubstituted cyclic 1,3-diketones,^6^ we observed that enone dione 1a can undergo an intramolecular Michael addition to form the chiral bicyclo[3.2.1]octane *rac*-2a under basic conditions.^7^ For example, treatment of 1a with NaOMe in THF at room temperature gave *rac*-2a in 81% yield (eqn (1)). Following this result, the development of a catalytic enantioselective variant captured our interest. However, a challenging feature of this reaction that differentiates it from the significant majority of catalytic enantioselective Michael reactions described previously^8^ is that stereogenicity is first generated in the *enolization step*, rather than the carbon–carbon bond-forming step (Scheme 1).^9^ Therefore, any chiral catalyst employed has to, at first glance, facilitate the enantioselective, desymmetrizing enolization^10^ of a 2,2-disubstituted cyclic 1,3-diketone. Due to the two substituents at the prochiral 2-position (methyl *versus* primary alkyl) possessing very similar steric properties, this task appeared to be far from trivial.

**Scheme 1:**
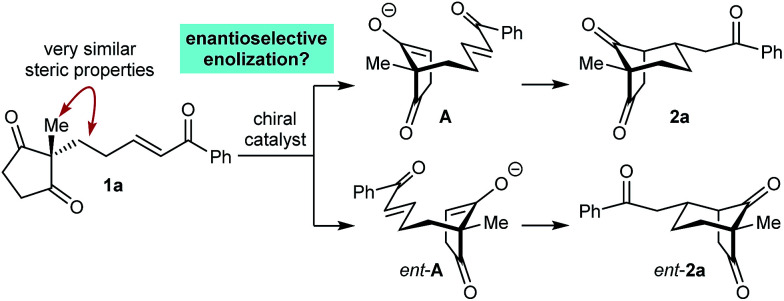
Asymmetric induction in the formation of 2a.

We hypothesized that a solution to this challenge could be obtained under conditions where enolization of 1a is reversible, potentially enabling rapid interconversion of the enolates A and *ent*-A. Under such Curtin–Hammett conditions,^11^ carbon–carbon bond formation could then be the enantiodetermining step. Furthermore, it appeared likely that chiral catalysts capable of binding to both the enolate oxygen atom and the enone carbonyl group would increase the energy difference between diastereomeric transition states, thus maximizing the prospects of achieving high enantioselectivities.

In the last ten years, chiral Brønsted acids, such as phosphoric acids, have emerged as extremely versatile catalysts for a diverse range of transformations.^12–14^ In many cases, both the Lewis acidity of the hydroxyl group and the Lewis basicity of the phosphoryl group of the catalyst play key roles in the simultaneous activation of electrophile–nucleophile pairs.^13^ We were therefore hopeful that chiral phosphoric acids would be suitable bifunctional catalysts for the enantioselective cyclization of 1a, and this indeed turned out to be the case (Table 1).^15^ For example, heating 1a in the presence of various BINOL-derived phosphoric acids 4a–4d (5 mol%) in toluene at 80 °C for 14–18 h led to complete consumption of 1a to form bicyclo[3.2.1]octane 2a as the major product (Table 1, entries 1–4).^16^ Small traces of a diastereomeric product 3a,^16^ in which the phenyl ketone-containing substituent occupies an axial position, were detected by TLC analysis, but the exact diastereomeric ratios could not be determined by ^1^H NMR analysis due to overlapping signals. Furthermore, promising enantioselectivities were obtained with phosphoric acids 4b–4d containing sterically more hindered aryl groups at the 3,3′-positions (entries 2–4). Catalysts 4b and 4c, possessing 2,4,6-trisubstituted aryl groups, gave the best results (entries 2 and 3). Switching to the H_8_-BINOL scaffold in catalyst 4e was detrimental (entry 5, compare with entry 2), while the vaulted biaryl-derived phosphoric acid 4f gave a low enantioselectivity (entry 6). Due to its commercial availability and relative ease of synthesis compared with 4c, phosphoric acid 4b (TRIP) was selected for further investigations. Changing the solvent to cyclohexane^17^ and lowering the reaction temperature to 50 °C gave marginally superior results (entries 7 and 8).

**Table 1:** Evaluation of catalysts and reaction conditions for the Michael cyclization of 1a^a^

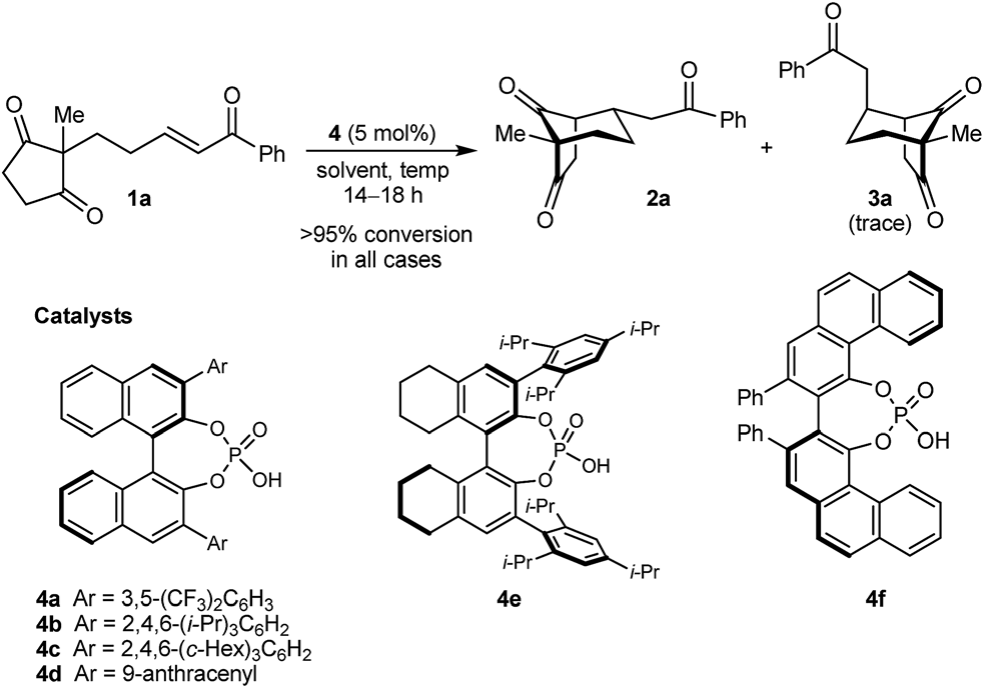				
Entry	Catalyst	Solvent	Temp. (°C)	ee^b^ of 2a (%)
1	4a	Toluene	80	28
2	4b	Toluene	80	89
3	4c	Toluene	80	90
4	4d	Toluene	80	76
5	4e	Toluene	80	74
6	4f	Toluene	80	17
7	4b	Cyclohexane	80	90
8	4b	Cyclohexane	50	91

a Reactions were conducted using 0.05 mmol of 1a. Complete consumption of 1a was observed in all cases by ^1^H NMR analysis.

b Determined by HPLC on a chiral stationary phase.

With an effective catalyst and solvent identified, the scope of the reaction with respect to the preparation of bicyclo[3.2.1] octanes was investigated (Table 2). The catalyst loading of 4b could be decreased from 5 mol% to 3 mol% without detriment, and the reactions were complete after 24 h. Under these conditions, a range of enone diones 1a–1k underwent Michael cyclizations to give products 2a–2k in generally high yields and with good to high enantioselectivities (86–95% ee).^16^ The process is compatible with electron-donating (entries 2 and 3) or electron-withdrawing aryl groups (4 and 5) on the enone carbonyl, as well as 2-naphthyl (entries 6 and 7) or *tert*-butyl groups (entry 8). Additional Lewis basic heteroatoms in heteroarene substituents such as 2-pyridyl, 2-furyl, or 2-thienyl groups were also tolerated (entries 9–11). Finally, replacement of the methyl group at the 2-position of the cyclic 1,3-diketone with an ethyl group did not affect the efficiency of the reaction (entry 12). In some cases, the reactions were highly diastereoselective, and only one product was detected (entries 2, 3, 6–8, 11, and 12). Although small but appreciable quantities of the minor diastereomeric products 3 were also formed in other cases, these were readily separated from the major isomers, the yields of which remained high (entries 1, 4, 5, 9, and 10). The enantiomeric excesses of the minor product were comparable to those of the major product in some cases (entries 1, 4, and 10), but were lower for substrates containing a 3-chlorophenyl or 2-pyridyl group attached to the enone carbonyl (entries 5 and 9). The process is also amenable to being conducted on a gram-scale. For example, the cyclization of 1f (1.00 g, 3.12 mmol) using 1.6 mol% of phosphoric acid 4b gave 2f as the only observable diastereomer in 84% yield and 90% ee after 90 h (entry 7).

**Table 2:** Enantioselective Michael cyclizations to give bicyclo[3.2.1]octanes^a^

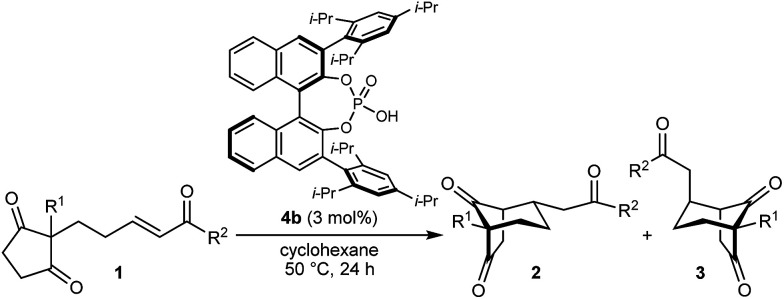				
Entry	R^1^	R^2^	Major product 2	Minor product 3
			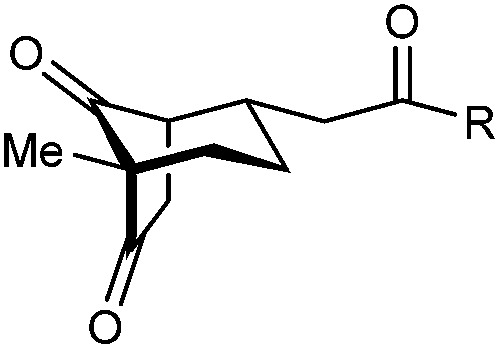	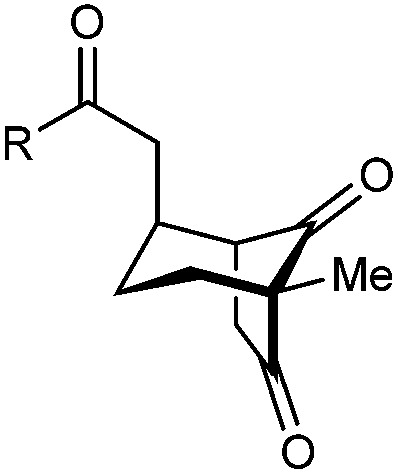
1	Me	Ph	2a 93%, 91% ee	3a 7%, 87% ee
2		4-MeC_6_H_4_	2b 91%, 92% ee	3b —b
3		4-MeOC_6_H_4_	2c 92%, 91% ee	3c —b
4		4-ClC_6_H_4_	2d 80%, 94% ee	3d 11%, 85% ee
5		3-ClC_6_H_4_	2e 79%, 86% ee	3e 13%, 66% ee
6		2-Naphthyl	2f 97%, 91% ee	3f —b
7^c^		2-Naphthyl	2f 84%, 90% ee	3f —b
8		*t*-Bu	2g 96%, 95% ee	3g —b
9^d^		2-Pyridyl	2h 76%, 87% ee	3h 20%, 29% ee
10		2-Furyl	2i 80%, 88% ee	3i 17%, 90% ee
11		2-Thienyl	2j 97%, 92% ee	3j —b

			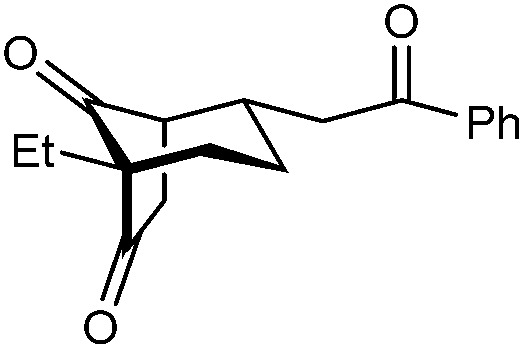	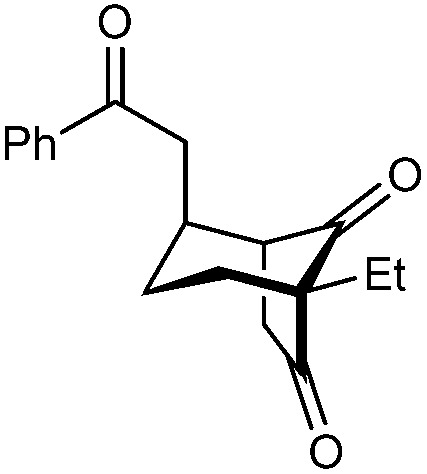
12	Et	Ph	2k 95%, 93% ee	3k —b

a The reactions were performed with 1a–k (0.20 mmol) in cyclohexane (2 mL). Yields are of pure isolated single diastereomers. Enantiomeric excesses were determined by HPLC analysis on a chiral stationary phase.

b The minor product was not detected.

c Conducted using 1f (1.00 g, 3.12 mmol) and 1.6 mol% of 4b in cyclohexane/toluene (4 : 1) at 50 °C for 90 h.

d The reaction was conducted in toluene.

The synthesis of bicyclo[3.3.1]nonanes 6 with good enantioselectivities is also possible using this methodology (Table 3). As with the corresponding bicyclo[3.2.1]octanes, many of these reactions also resulted in diastereomeric products (Table 3, entries 1, 4–9, and 11–13). In general, the enantiomeric excess of the major products were slightly lower compared with those of the bicyclo[3.2.1] octanes 2 (see Table 2), though interestingly, the minor diastereomers 7 were usually formed in higher enantioselectivities. The process remained broadly tolerant of different (hetero)arenes at the enone carbonyl group, including *ortho*-substituted phenyl groups (entries 8 and 9). A 4-nitrophenyl ketone led to a more modest enantioselectivity for the major product (entry 6). The reaction was also compatible with an alkyl substituent at the enone carbonyl group that possesses enolizable protons; the cyclization of 5l gave products 6l and 7l in 59% combined yield (entry 12). Again, variation of the substituent at the 2-position of the 1,3-diketone was possible, with allyl (entries 13 and 14), phenyl (entries 15 and 16), and *para*-methoxyphenyl groups (entries 17 and 18) providing good results. In particular, aryl substituents at the 2-position had a beneficial effect on the enantioselectivity compared with the corresponding methyl-substituted analogues. For example, 6o and 6q were obtained in significantly higher enantioselectivity (94% ee, entries 15 and 17) compared with 6a (82% ee, entry 1).

**Table 3:** Enantioselective Michael cyclizations to give bicyclo[3.3.1]nonanes^a^

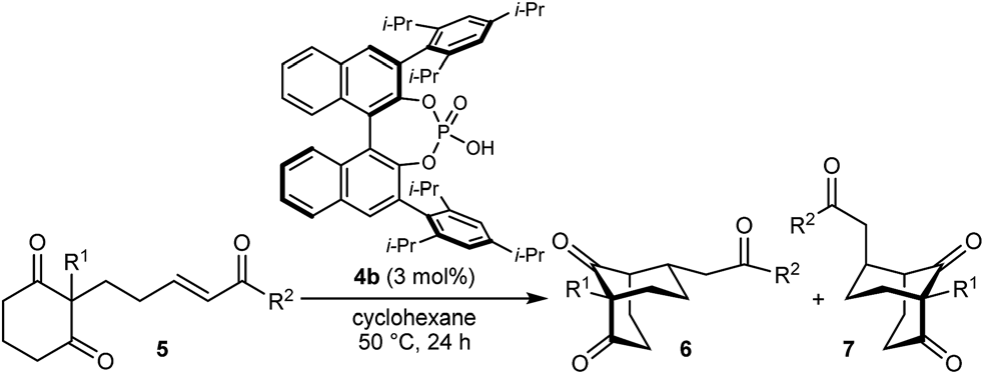				
Entry	R^1^	R^2^	Major product 6	Minor product 7
			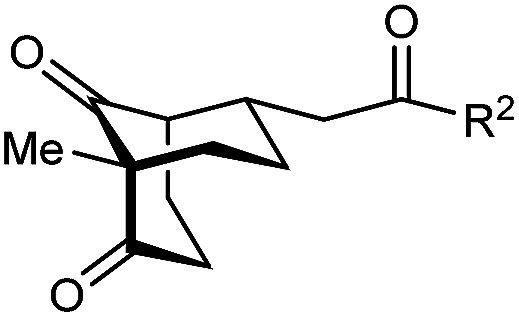	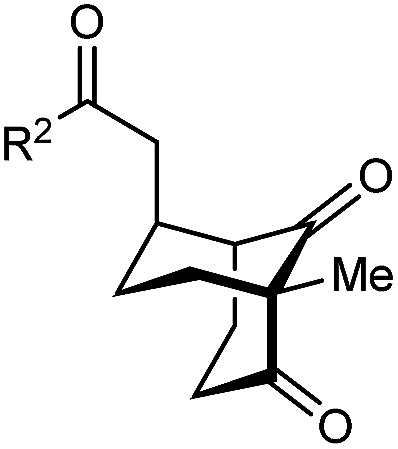
1	Me	Ph	6a 77%, 82% ee	7a 14%, 94% ee
2		4-MeC_6_H_4_	6b 95%, 86% ee	7b —b
3		4-MeOC_6_H_4_	6c 94%, 87% ee	7c —b
4		4-FC_6_H_4_	6d 82%, 86% ee	7d 13%, 86% ee
5		4-ClC_6_H_4_	6e 73%, 87% ee	7e 19%, 96% ee
6^c^		4-NO_2_C_6_H_4_	6f 85%, 72% ee	7f 15%, 88% ee
7^d^		3-CF_3_C_6_H_4_	6g 68%, 86% ee	7g 21%, 94% ee
8		2-MeOC_6_H_4_	6h 60%, 83% ee	7h 24%, 93% ee
9		2-ClC_6_H_4_	6i 75%, 92% ee	7i 20%, 85% ee
10		2-Naphthyl	6j 96%, 87% ee	7j —b
11		2-Pyridyl	6k 75%, 82% ee	7k 19%, 46% ee
12		CH_2_CH_2_OBn	6l 35%, 92% ee	7l 24%, 80% ee

			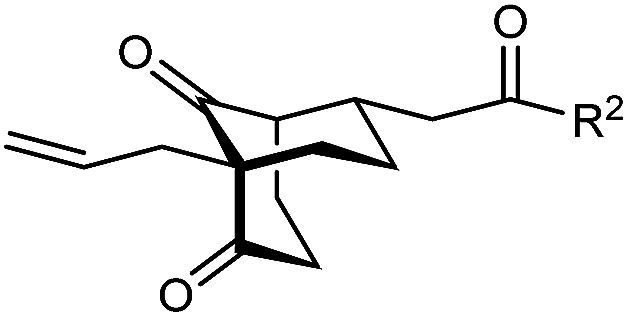	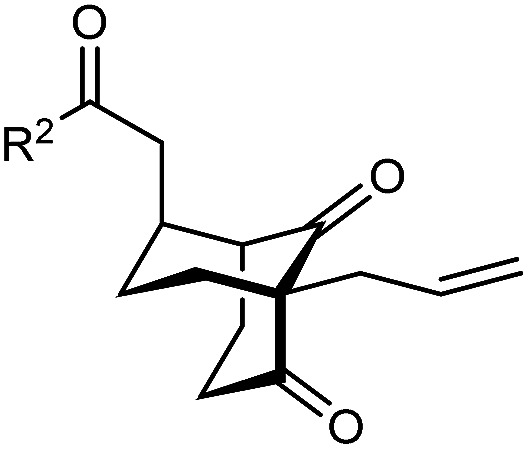
13	Allyl	Ph	6m 89%, 86% ee	7m 6%, 94% ee
14		4-ClC_6_H_4_	6n 94%, 88% ee	7n —b

			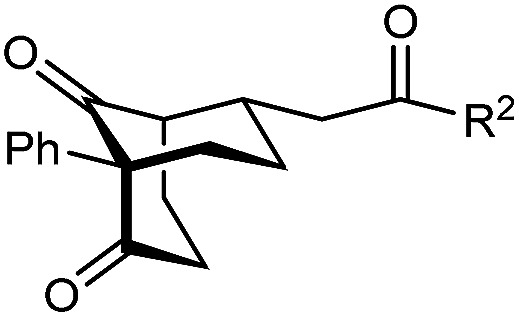	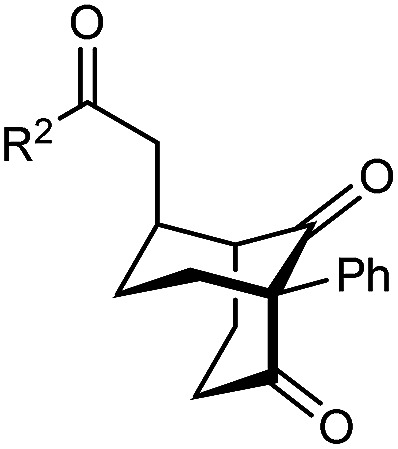
15	Ph	Ph	6o 68%, 94% ee	7o —b
16		2-Thienyl	6p 50%, 97% ee	7p —b

			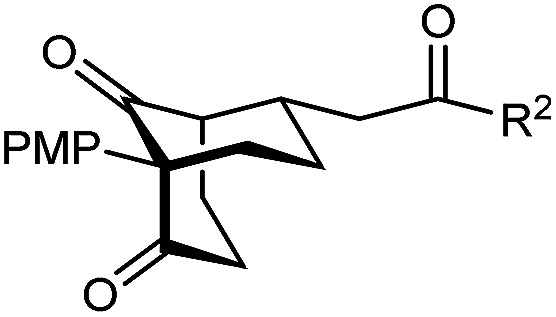	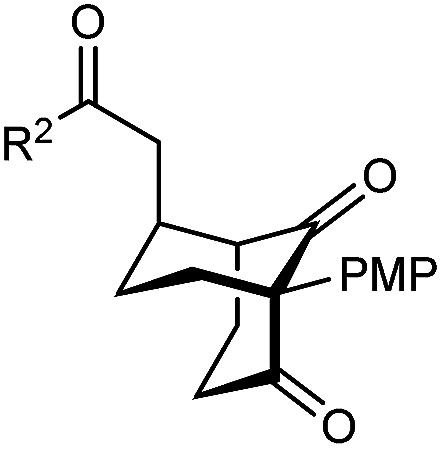
17	PMP	Ph	6q 63%, 94% ee	7q —b
18		2-Thienyl	6r 49%, 92% ee	7r —b

a The reactions were performed with 5a–5r (0.20 mmol) in cyclohexane (2 mL). Yields are of pure isolated single diastereomers. Enantiomeric excesses were determined by HPLC analysis on a chiral stationary phase.

b The minor product was not detected.

c The reaction was conducted in toluene.

d The reaction was conducted in cyclohexane (4 mL). PMP = *para*-methoxyphenyl.

The process is not limited to α,β-unsaturated ketones as the Michael acceptor. For example, substrate 8, containing an α,β-unsaturated amide, successfully underwent cyclization to give bicyclo[3.3.1]nonane 9 in 52% yield and 77% ee, with the starting material being recovered in 40% yield (eqn (2)). Due to the lower reactivity of 8 compared with α,β-unsaturated ketones, a higher catalyst loading, temperature, and reaction time were required for reasonable results. Interestingly, the benzyl ester analogue of 8, in which the alkene is expected to be more electrophilic than in 8, was unreactive towards chiral phosphoric acid-catalyzed Michael cyclizations. This observation suggests that in addition to the electrophilicity of the electron-deficient alkene, the Lewis basicity of the oxygen atom of the α,β-unsaturated carbonyl (to facilitate binding of the chiral phosphoric acid) is also important for reactivity. Furthermore, the 2-alkenylbenzoxazole-containing substrate 10 also underwent cyclization in a good yield (eqn (3)) although the enantioselectivity of this reaction was modest (62% ee).^18^2
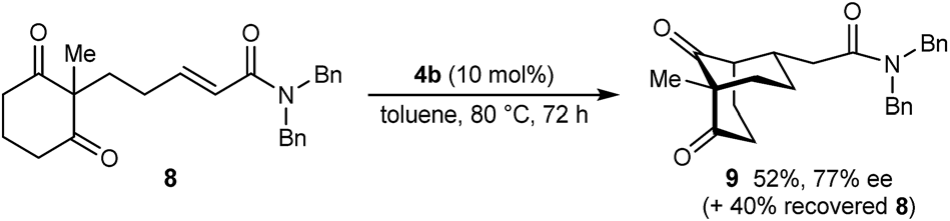
3
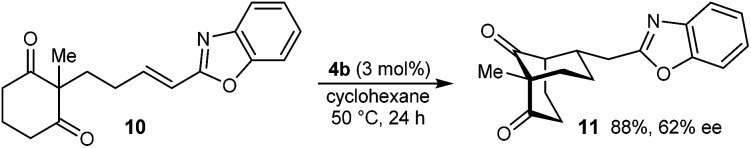


Scheme 2 presents a working hypothesis for the mode of action of the chiral phosphoric acid 4b, using substrate 1a for illustration. We propose that the catalyst promotes reversible keto-enol tautomerization of 1a,^5^ and is able to simultaneously bind the carbonyl group of the electrophilic enone and the hydroxyl group of the nucleophilic enol. The formation of the enantiomers 2a and *ent*-2a of the major product can be explained by chair-like conformations B and C, respectively, in which the enone occupies a pseudoequatorial position. The preferential formation of 2a is consistent with cyclization through conformation B being favored, in which the enol attacks the *Si*-face of the alkene.^19^ The formation of the two enantiomers 3a and *ent*-3a of the minor product can be explained by conformations D and E, where the enone occupies a pseudoaxial position. Again, attack of the *Si*-face of the alkene is favored (conformation D), leading to the preferential formation of 3a.^19^ This model is similar to one proposed by List and co-workers to explain the mode of enantioinduction in asymmetric chiral phosphoric acid-catalyzed Fischer indolizations.^20^

**Scheme 2:**
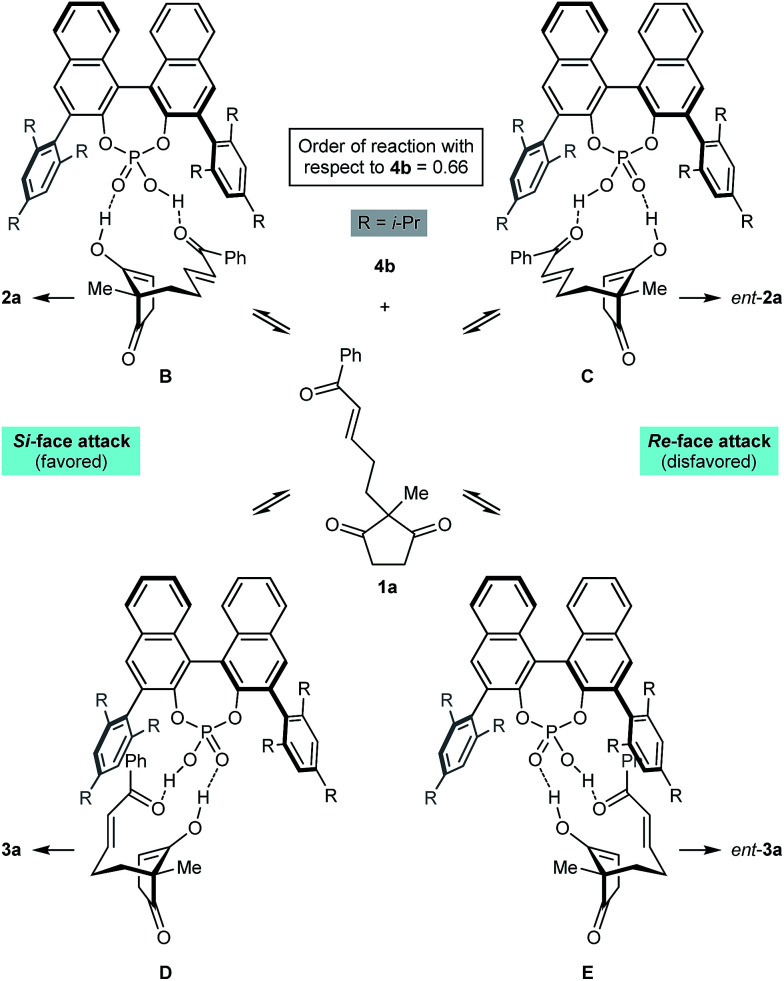
Proposed mode of action of catalyst 4b.

Preliminary kinetic studies were also performed on the cyclization of 5d into 6d and 7d in toluene-d8 using different concentrations of catalyst 4b. From these experiments, the order of the reaction with respect to 4b was calculated to be 0.66.^21^ This non-integer value confirms the mechanism of the reaction is indeed complex, and may involve a series of equilibria as presented in Scheme 2. The complexity of the mechanism was further confirmed by a reaction in which the enantiomeric excess of 2j was measured during the course of the cyclization; the ee of 2j was not constant throughout, and increased from 70% ee after 1 h (15% conversion) to 90% ee after 24 h (75% conversion).^21,22^ Rationalization of these observations awaits the results of further studies.

In summary, the enantioselective synthesis of bicyclo[3.2.1]octanes and bicyclo[3.3.1]nonanes has been achieved by the chiral phosphoric acid-catalyzed Michael cyclizations of enone diones. These reactions involve the unusual enantioselective desymmetrization of 2,2-disubstituted cyclic 1,3-diketones, in which the bifunctional activation of the substrate by the catalyst is likely to be critical for success. This work further demonstrates the utility of chiral Brønsted acids in the enantioselective preparation of stereochemically complex structures, and investigation of these catalysts in other desymmetrization processes are likely to result in further advances in future. These studies, along with further mechanistic experiments, are topics for future study in our group.

## Supplementary Material

SC-006-C5SC00753D-s001

SC-006-C5SC00753D-s002

## References

[cit1] Presset M., Coquerel Y., Rodriguez J. (2013). Chem. Rev..

[cit2] Zhao W. (2010). Chem. Rev..

[cit3] Presset M., Coquerel Y., Rodriguez J. (2012). ChemCatChem.

[cit4] Itagaki N., Kimura M., Sugahara T., Iwabuchi Y. (2005). Org. Lett..

[cit5] Mori K., Katoh T., Suzuki T., Noji T., Yamanaka M., Akiyama T. (2009). Angew. Chem., Int. Ed..

[cit6] Low D. W., Pattison G., Wieczysty M. D., Churchill G. H., Lam H. W. (2012). Org. Lett..

[cit7] Danishefsky S., Koppel G., Levine R. (1968). Tetrahedron Lett..

[cit8] Schneider C., Abels F. (2014). Org. Biomol. Chem..

[cit9] During the final preparation of this manuscript, the synthesis of 2-azabicyclo[3.3.1]nonanes by catalytic enantioselective desymmetrizing Michael cyclizations was reported see ref. 4q

[cit10] (a) Simpkins N. S. and Weller M. D., in Stereochemical Aspects of Organolithium Compounds, Verlag Helvetica Chimica Acta, 2010, pp. 1–52

[cit11] Seeman J. I. (1983). Chem. Rev..

[cit12] Akiyama T., Itoh J., Yokota K., Fuchibe K. (2004). Angew. Chem., Int. Ed..

[cit13] (d) Kampen D. , Reisinger C. M. and List B., in Asymmetric Organocatalysis, ed. B. List, 2009, pp. 395–456 10.1007/978-3-642-02815-1_1 21494945

[cit14] Higuchi K., Suzuki S., Ueda R., Oshima N., Kobayashi E., Tayu M., Kawasaki T. (2015). Org. Lett..

[cit15] Akiyama T., Katoh T., Mori K. (2009). Angew. Chem., Int. Ed..

[cit16] The relative and absolute stereochemistries of the products described herein were assigned by analogy to those of products 2d, 3a, 6e, and 6f, which were determined by X-ray crystallography. See the ESI.†

[cit17] Although the substrate and phosphoric acid 4b are poorly soluble in cyclohexane at room temperature, the reaction mixtures become homogeneous upon heating to 50 °C

[cit18] Baschieri A., Bernardi L., Ricci A., Suresh S., Adamo M. F. A. (2009). Angew. Chem..

[cit19] However, the precise manner in which the catalyst 4b favors *Si*-face attack is not readily explained at the present time

[cit20] Müller S., Webber M. J., List B. (2011). J. Am. Chem. Soc..

[cit21] See ESI for full details.†

[cit22] This experiment was conducted in toluene rather than cyclohexane to ensure complete homogeneity of the reaction mixture

