# Supplementary material for: Enantioselective synthesis of bicyclo[3.n.1]alkanes by chiral phosphoric acid-catalyzed desymmetrizing Michael cyclizations
Source: Chem Sci. 2015 Apr 30;6(6):3550–5. doi: 10.1039/c5sc00753d (PMC5659222; doi:10.1039/c5sc00753d)
Supplement: SC-006-C5SC00753D-s001 [file SC-006-C5SC00753D-s001.pdf]

Electronic Supplementary Material (ESI) for Chemical Science.
This journal is © The Royal Society of Chemistry 2015

Enantioselective Synthesis of Bicyclo[3.n.1]alkanes by Chiral Phosphoric Acid-Catalyzed Desymmetrizing Michael Cyclizations

Alan R. Burns,[a,b,†] Amaël G. E. Madec,[b,†] Darryl W. Low,[a] Iain D. Roy,[a] and Hon Wai Lam*[,a,b]

[a] *EaStCHEM, School of Chemistry, University of Edinburgh, Joseph Black Building, The King's Buildings, David Brewster Road, Edinburgh, EH9 3FJ (UK)*

[b] *School of Chemistry, University of Nottingham, University Park, Nottingham, NG7 2RD (UK)*

† These authors contributed equally.

Supporting Information

Contents **Page**

General Information 2

Preparation of Chiral Phosphoric Acids 3

Preparation of Cyclization Precursors 3

Enantioselective Michael Cyclizations 19

Tests for the Self-Disproportionation of Enantiomers (SDE) Phenomenon 46

Preliminary Kinetic Experiments 49

Measurement of Enantioselectivity of **2j** with Increasing Conversion 50

NMR Spectra 54

HPLC Traces 129

General Information

All commercially available reagents were used as received. “Petroleum ether” refers to Sigma-Aldrich product 24587 (petroleum ether boiling point 40-60 °C). Thin layer chromatography (TLC) was performed on Merck DF-Alufoilien 60F_{254} 0.2 mm precoated plates. Product spots were visualized by UV light at 254 nm, and subsequently developed using potassium permanganate or vanillin solution as appropriate. Flash column chromatography was carried out using silica gel (Fisher Scientific 60Å particle size 35-70 micron). Melting points were recorded on a Griffin melting point apparatus and are uncorrected. Infra-red spectra were recorded on a Nicolet Avatar 360 FT instrument on the neat compound using an attenuated total reflection (ATR) accessory with a diamond crystal and a germanium sample plate or on a Bruker Tensor 27 FT instrument as a $CHCl_3$ solution. NMR spectra were acquired on Bruker AVA500, Bruker AVA400, Bruker DPX400, or Bruker DPX300 spectrometers. ^{1}H and ^{13}C NMR spectra were referenced to external tetramethylsilane via the residual protonated solvent (^{1}H) or the solvent itself (^{13}C). All chemical shifts are reported in parts per million (ppm). For $CDCl_3$, the shifts are referenced to 7.27 ppm for ^{1}H NMR spectroscopy and 77.0 ppm for ^{13}C NMR spectroscopy. Abbreviations used in the description of resonances are: s (singlet), d (doublet), t (triplet), q (quartet), quin (quintet), app (apparent), br (broad), m (multiplet). Coupling constants (J) are quoted to the nearest 0.1 Hz. Assignments were made using the DEPT sequence with secondary pulses at 90° and 135° and 2D COSY and HSQC experiments. Proton-decoupled ^{19}F NMR spectra were recorded on a Bruker DPX300 (282 MHz), a Bruker DPX400 (376 MHz), or a Bruker AV400 (376 MHz) spectrometer. Chemical shifts (δ) are quoted in parts per million (ppm) downfield of $CFCl_3$ (δ = 0 ppm), using fluorobenzene as internal reference (C_6H_5F at –113.5 ppm). Proton-decoupled ^{31}P NMR spectra were recorded on a Bruker DPX400 (162 MHz), or a Bruker AV400 (162 MHz) spectrometer. Chemical shifts (δ) are quoted in parts per million (ppm) downfield of tetramethylsilane, using residual protonated solvent as internal reference (aqueous 85% H_3PO_4 at 162 MHz with respect to tetramethylsilane at 400.00 MHz). High-resolution mass spectra were recorded using electrospray ionization (ESI) or electron impact ionization (EI) techniques. Optical rotations were performed on a Bellingham and Stanley ADP 400 polarimeter. Chiral HPLC analysis was performed on an Agilent 1290 series or Agilent 1260 series instrument using 4.6 x 250 mm columns. Authentic racemic samples of products for chiral HPLC assay determinations were obtained using (±)-CSA (20 mol%) as a racemic catalyst, or NaOMe (1.0 equiv) as an achiral base in THF.

Preparation of Phosphoric Acids

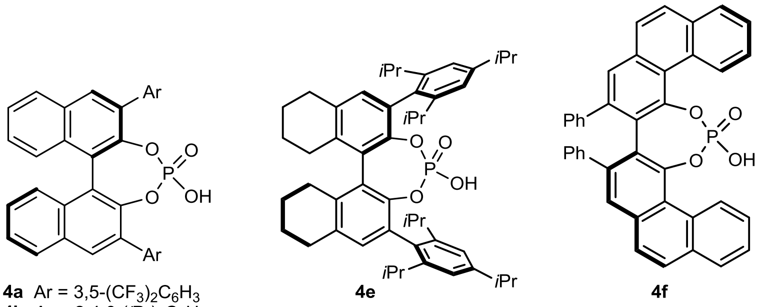

4a Ar = 3,5-$(CF_3)_2C_6H_3$
4b Ar = 2,4,6-$(i\text{Pr})_3C_6H_2$
4c Ar = 2,4,6-$(c\text{Hex})_3C_6H_2$
4d Ar = 9-anthracenyl
4e
4f

Phosphoric acids **4a** and **4f** were purchased from commercial sources. Phosphoric acids **4b**,[1] **4c**,[2] **4d**,[3] and **4e**[2] were prepared according to the reported procedures (**4b** and **4d** are also commercially available).

Preparation of Cyclization Precursors

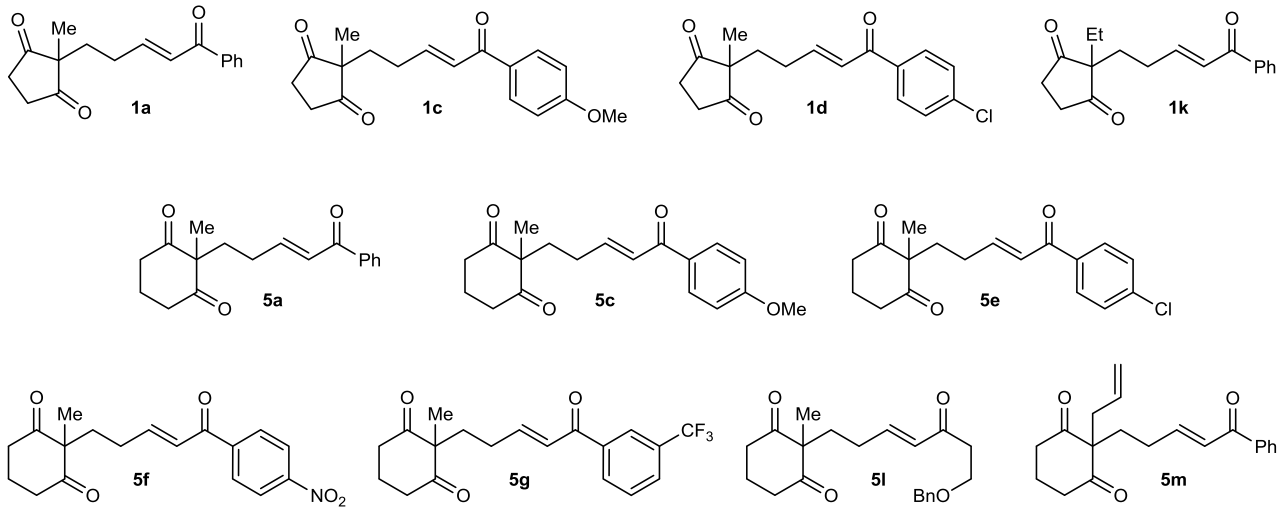

Substrates were prepared *via* a Wittig reaction of the corresponding aldehyde and phosphorane. Enone diones **1a**,[4] **1c**,[5] **1d**,[5] **1k**,[5] **5a**,[4] **5c**,[5] **5e**,[5] **5f**,[5] **5g**,[5] **5l**,[5] and **5m**[5] are known compounds prepared *via* literature procedures.

1. M. Klussmann, L. Ratjen, S. Hoffmann, V. Wakchaure, R. Goddard, B. List, *Synlett* **2010**, 2189-2192.
2. F. Romanov-Michailidis, L. Guénée, A. Alexakis, *Angew. Chem., Int. Ed.* **2013**, *52*, 9266-9270.
3. F. Romanov-Michailidis, L. Guénée, A. Alexakis, *Org. Lett.* **2013**, *15*, 5890-5893.
4. R. R. Huddleston, M. J. Krische, *Org. Lett.* **2003**, *5*, 1143-1146.
5. A. R. Burns, J. Solana González, H. W. Lam, *Angew. Chem., Int. Ed.* **2012**, *51*, 10827-10831.

3-(1-Ethyl-2,5-dioxocyclopentyl)propanal (S1)[5]

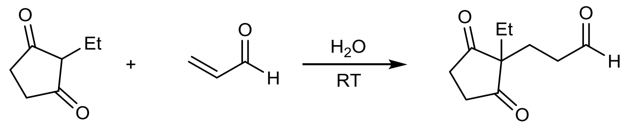

S1

To a stirred solution of 2-ethyl-1,3-cyclopentanedione (500 mg, 3.96 mmol) in H_2O (20 mL) was added acrolein (0.40 mL, 5.94 mmol) in one portion and the resulting mixture was stirred at room temperature for 22 h. The reaction mixture was extracted with CH_2Cl_2 and the combined organic layerss were dried ($MgSO_4$), filtered, and concentrated *in vacuo* to give the aldehyde **S1** as a yellow oil (679 mg, 94%) that displayed spectroscopic data consistent with those reported in the literature.[5]

3-(2,6-Dioxo-1-phenylcyclohexyl)propanal (S2)

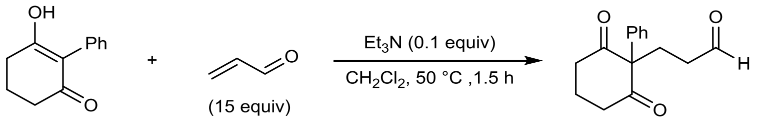

S2

To a suspension of 3-hydroxy-2-phenylcyclohex-2-en-1-one[6] (1.00 g, 5.30 mmol) and Et_3N (82 µL, 0.59 mmol) in CH_2Cl_2 (4 mL) was added acrolein (5.31 mL, 79.5 mmol) and the reaction was heated at 50 °C for 1.5 h. The reaction was concentrated *in vacuo* to afford the crude residue. Purification of the residue by column chromatography (15% EtOAc/petroleum ether) gave the *aldehyde* **S2** as an orange oil (323 mg, 25%). R_f = 0.35 (15% EtOAc/petroleum ether); IR 2940, 1707 (C=O), 1680 (C=O), 1601, 1499, 1448, 1379, 1240, 1154, 914 cm^{-1}; ^1H NMR (300 MHz, $CDCl_3$) δ 9.65-9.62 (1H, m, **H**C=O), 7.45-7.29 (3H, m, Ar**H**), 7.05-6.95 (2H, m, Ar**H**), 2.85-2.71 (2H, m, $C\mathbf{H_A}H_BCH_2C\mathbf{H_A}H_B$), 2.62-2.49 (2H, m, $CH_A\mathbf{H_B}CH_2CH_A\mathbf{H_B}$), 2.39-2.23 (4H, m, $C\mathbf{H_2}C\mathbf{H_2}CHO$), 1.97-1.83 (1H, m, $CH_2C\mathbf{H_A}H_BCH_2$), 1.79-1.64 (1H, m, $CH_2CH_A\mathbf{H_B}CH_2$); ^{13}C NMR (75 MHz, $CDCl_3$) δ 207.1 (2 x C), 201.8 (C), 137.5 (C), 129.7 (2 x CH), 128.2 (CH), 126.5 (2 x CH), 75.0 (C), 40.3 (CH_2), 39.0 (2 x CH_2), 27.1 (CH_2), 17.5 (CH_2); HRMS (ESI) Exact mass calculated for $C_{16}H_{20}NaO_4$ $[M+Na+MeOH]^+$: 299.1254 found: 299.1246.

6. (a) T. N. Wheeler, *J. Org. Chem.* **1979**, *44*, 4906-4912. (b) S. Reddy Chidipudi, I. Khan, H. W. Lam, *Angew. Chem., Int. Ed.* **2012**, *51*, 12115-12119.

3-(2,6-Dioxo-1-phenylcyclohexyl)propanal (S3)

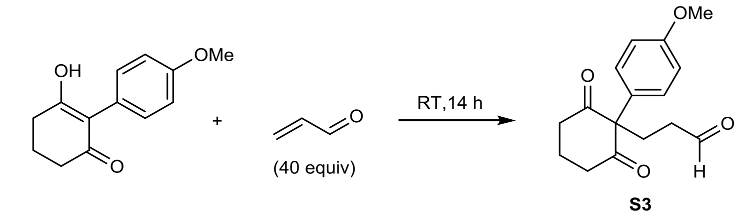

A solution of 3-hydroxy-2-(4-methoxyphenyl)cyclohex-2-en-1-one[6b] (600 mg, 2.75 mmol) and acrolein (7.35 mL, 110 mmol) was stirred at room temperature for 14 h. The reaction mixture was concentrated *in vacuo* to afford the crude residue. Purification of the residue by column chromatography (15% acetone/petroleum ether) gave the aldehyde **S3** as a yellow oil (400 mg, 53%). R_f = 0.30 (15% acetone/petroleum ether); IR 2960, 2837, 1725 (C=O), 1698 (C=O), 1608, 1511, 1255, 1187, 1033, 832cm^{-1}; ^{1}H NMR (400 MHz, $CDCl_3$) δ 9.58 (1H, t, *J* = 1.5 Hz, **H**C=O), 6.89-6.84 (4H, m, Ar**H**), 3.76 (3H, s, C**H**$_3$), 2.78-2.69 (2H, m, C**H$_A$**H$_B$CH$_2$C**H$_A$**H$_B$), 2.54-2.45 (2H, m, CH$_A$**H$_B$**CH$_2$CH$_A$**H$_B$**), 2.31-2.25 (2H, m, C**H**$_2$CHO), 2.23-2.17 (2H, m, C**H**$_2$CH$_2$CHO), 1.91-1.79 (CH$_2$C**H$_A$**H$_B$CH$_2$), 1.73-1.60 (CH$_2$CH$_A$**H$_B$**CH$_2$); ^{13}C NMR (100.6 MHz, $CDCl_3$) δ 207.2 (2 x C), 201.8 (C), 159.3 (C), 129.1 (C), 127.7 (2 x CH), 115.0 (2 x CH), 74.1 (C), 55.2 (CH_3), 40.2 (CH_2), 38.7 (2 x CH_2), 26.9 (CH_2), 17.3 (CH_2); HRMS (ESI) Exact mass calculated for $C_{16}H_{19}O_4$ $[M+H]^+$: 275.1278 found: 275.1268.

Representative Procedure for the Preparation of Phosphoranes

1-(3-Chlorophenyl)-2-(triphenyl-λ^5-phosphanylidene)ethan-1-one (S4)

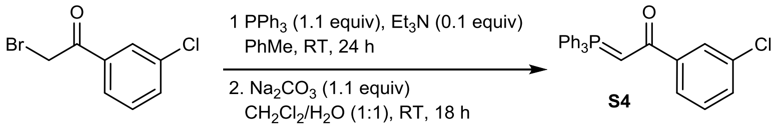

To a stirred solution of 2-bromo-3'-chloroacetophenone (4.40 g, 18.9 mmol) and Et_3N (0.26 mL, 1.89 mmol) in toluene (63 mL) was added PPh_3 (5.44 g, 20.7 mmol) and the mixture was stirred at room temperature for 24 h. The resulting precipitate was filtered, washed copiously with Et_2O, and dried *in vacuo* to give the phosphonium salt. To a stirred suspension of the phosphonium salt in CH_2Cl_2 (20 mL) was added Na_2CO_3 (2.20 g, 20.7 mmol) in H_2O (20 mL) and the resulting biphasic solution was stirred vigorously at room temperature for 18 h. The layers were separated and the aqueous layers was extracted with CH_2Cl_2 (20mL). The combined organic layerss were dried ($MgSO_4$), filtered, and concentrated *in vacuo* to give the *phosphorane* **S4** as a pale brown solid (6.57 g, 84%). R_f = 0.14 (40% EtOAc/petroleum ether); m.p. 135-137 °C (cyclohexane/CH_2Cl_2); IR 1514, 1441, 1435, 1378, 1105, 887, 742 cm^{-1}; ^{1}H NMR (400 MHz, $CDCl_3$) δ 7.97 (1H, t, *J* = 1.8 Hz, Ar**H**), 7.85 (1H, dt, *J* = 7.4, 1.4 Hz, Ar**H**), 7.77-7.68 (6H, m, Ar**H**), 7.62-7.56 (3H, m, Ar**H**),

7.53-7.46 (6H, m, Ar**H**), 7.36-7.26 (2H, m, Ar**H**), 4.43 (1H, br s, Ph_3P=C**H**); ^{13}C NMR (100.6 MHz, $CDCl_3$) δ 183.0 (C, d, *J* = 3.4 Hz), 143.2 (C, d, *J* = 14.9 Hz), 133.8 (C), 133.1 (6 x CH, *J* = 10.2 Hz), 132.2 (3 x CH, *J* = 2.9 Hz), 129.2 (CH), 129.0 (CH), 128.92 (6 x CH, *J* = 12.4 Hz), 127.2 (CH), 126.7 (3 x C, d, *J* = 91.4 Hz), 125.0 (CH), 51.4 (CH, d, *J* = 112.1 Hz); ^{31}P NMR (162 MHz, $CDCl_3$) δ 16.7; HRMS (ESI) Exact mass calculated for $C_{26}H_{21}ClOP$ $[M+H]^+$: 415.1013, found: 415.1014.

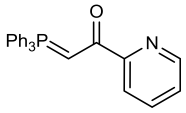

1-(Pyridin-2-yl)-2-(triphenyl-λ⁵-phosphanylidene)ethan-1-one (S5). The title compound was prepared according to the Representative Procedure from (2-bromoacetyl)pyridin-1-ium bromide[7] (9.50 g, 33.8 mmol) to give an off-white gummy solid (11.6 g, 90%). R_f = 0.18 (9/18/73 MeOH/EtOAc/petroleum ether); IR 2959, 2928, 1724 (C=O), 1572, 1522, 1483, 1438, 1397, 1239, 1107 cm^{-1}; ^1H NMR (400 MHz, $CDCl_3$) δ 8.59 (1H, d, *J* = 4.0 Hz, Ar**H**), 8.15 (1H, d, *J* = 7.9 Hz, Ar**H**), 7.80-7.70 (6H, m, Ar**H**), 7.60-7.53 (3H, m, Ar**H**), 7.52-7.45 (6H, m, Ar**H**), 7.31-7.25 (2H, m, Ar**H**), 5.32 (1H, d, *J* = 21.4 Hz, Ph_3P=C**H**); ^{13}C NMR (100.6 MHz, $CDCl_3$) δ 196.5 (C, d, *J* = 5.7 Hz), 148.0 (CH), 141.1 (C) 136.6 (CH), 133.3 (6 x CH, d, *J* = 10.2 Hz), 132.1 (3 x CH, d, *J* = 2.7 Hz), 128.9 (6 x CH, d, *J* = 12.3 Hz), 127.0 (3 x C, d, *J* = 91.5 Hz), 126.1 (3 x C, d, *J* = 91.5 Hz), 124.1 (CH), 120.6 (CH), 51.9 (CH, d, *J* = 110.9 Hz); ^{31}P NMR (162 MHz, $CDCl_3$) δ 17.4; HRMS (ESI) Exact mass calculated for $C_{25}H_{21}NOP$ $[M+H]^+$: 382.1365, found: 382.1369.

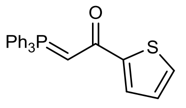

1-(Thiophen-2-yl)-2-(triphenyl-λ⁵-phosphanylidene)ethan-1-one (S6). The title compound was prepared according to the Representative Procedure from 2-bromo-1-(thiophen-2-yl)ethan-1-one[8] (3.80 g, 18.5 mmol) to give a beige solid (5.87 g, 82%). R_f = 0.27 (70% EtOAc/petroleum ether); m.p. 209-211 °C (cyclohexane/CH_2Cl_2); IR 1506, 1384, 1231, 1107, 880 cm^{-1}; ^1H NMR (400 MHz, $CDCl_3$) δ 7.78-7.66 (6H, m, Ar**H**), 7.61-7.52 (4H, m, Ar**H**), 7.52-7.44 (6H, m, Ar**H**), 7.30 (1H, d, *J* = 4.9 Hz, Ar**H**), 7.02 (1H, dd, *J* = 4.9, 3.7 Hz, Ar**H**), 4.32 (1H, br s, Ph_3P=C**H**); ^{13}C NMR (100.6 MHz, $CDCl_3$) δ 178.3 (C, d, *J* = 4.1 Hz), 148.5 (C, d, *J* = 18.1 Hz), 133.1 (6 x CH, d, *J* = 10.3 Hz), 132.1 (3 x CH, d, *J* = 2.8 Hz), 128.9 (6 x CH, d, *J* = 12.3 Hz), 127.3 (3 x C, d, *J* = 91.4 Hz), 127.11 (CH), 127.09 (CH), 126.8 (3 x C, d, *J* = 91.4 Hz), 126.0 (CH), 50.2 (CH, d, *J* = 113.3 Hz); ^{31}P NMR (162 MHz, $CDCl_3$) δ 16.3; HRMS (ESI) Exact mass calculated for $C_{24}H_{20}OPS$ $[M+H]^+$: 387.0967, found: 387.0965.

7. F. Mjambili, M. Njoroge, K. Naran, C. De Kock, P. J. Smith, V. Mizrahi, D. Warner, K. Chibale, *Bioorg. Med. Chem. Lett.* **2014**, *24*, 560-564.
8. J. Chen, D. Liu, N. Butt, C. Li, D. Fan, Y. Liu, W. Zhang, *Angew. Chem., Int. Ed.* **2013**, *52*, 11632-11636.

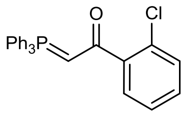

1-(2-Chlorophenyl)-2-(triphenyl-λ⁵-phosphanylidene)ethan-1-one (S7). The title compound was prepared according to the Representative Procedure from 2-bromo-1-(2-chlorophenyl)ethan-1-one[8] (2.33 g, 10.0 mmol) to give a pale yellow solid (4.15 g, >95%, but contaminated with a small quantity of Ph_3P=O). R_f = 0.30 (70% EtOAc/petroleum ether); m.p. 144-146 °C (cyclohexane/CH_2Cl_2); IR 3059, 1528, 1435, 1393, 1189, 1121, 748 cm^{-1}; ^{1}H NMR (400 MHz, $CDCl_3$) δ 7.81-7.72 (6H, m, Ar**H**), 7.71-7.53 (4H, m, Ar**H**), 7.53-7.44 (6H, m, Ar**H**), 7.37-7.29 (1H, m, Ar**H**), 7.24-7.15 (2H, m, Ar**H**), 4.11 (1H, br s, Ph_3P=C**H**); ^{13}C NMR (100.6 MHz, $CDCl_3$) δ 185.5 (C, d, *J* = 3.4 Hz), 143.2 (C, d, *J* = 15.9 Hz), 133.2 (6 x CH, d, *J* = 10.3 Hz), 132.2 (3 x CH, d, *J* = 2.9 Hz), 130.9 (C), 129.6 (CH), 129.3 (CH), 128.7 (CH), 128.9 (6 x CH, d, *J* = 12.3 Hz), 126.5 (3 x C, d, *J* = 90.9 Hz), 55.4 (CH, d, *J* = 106.2 Hz); ^{31}P NMR (162 MHz, $CDCl_3$) δ 14.8; HRMS (ESI) Exact mass calculated for $C_{26}H_{21}ClOP$ $[M+H]^+$: 415.1013, found: 415.1014.

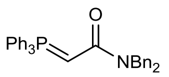

***N,N*-Dibenzyl-2-(triphenyl-λ⁵-phosphanylidene)acetamide (S8)**. The title compound was prepared according to the Representative Procedure from *N,N*-dibenzyl-2-chloroacetamide[9] (4.90 g, 20.0 mmol) to give a brown oil (9.69 g, >95%). R_f = 0.36 (70% EtOAc/petroleum ether); IR 3056, 1653 (C=O), 1636, 1541, 1495, 1437, 1183, 1120, 1028, 721 cm^{-1}; ^{1}H NMR (400 MHz, $CDCl_3$) δ 7.80-7.62 (6H, m, Ar**H**), 7.62-7.40 (9H, m, Ar**H**), 7.39-7.15 (10H, m, Ar**H**), 4.63-4.46 (4H, m, 2 x C**H**$_2$Ph), 3.02 (1H, br s, Ph_3P=C**H**); ^{13}C NMR (100.6 MHz, $CDCl_3$) δ 171.1 (C), 139.7 (C), 137.3 (3 x C, d, *J* = 90.9 Hz), 136.6 (3 x C, d, *J* = 90.9 Hz), 133.0 (6 x CH, d, *J* = 9.8 Hz), 132.0 (6 x CH, d, *J* = 9.9 Hz), 131.9 (3 x CH, d, *J* = 2.8 Hz), 131.4 (CH), 128.9 (CH), 128.54 (CH), 128.49 (CH), 128.42 (CH), 128.38 (CH), 128.24 (CH), 126.16 (CH), 127.6 (CH), 126.5 (CH), 126.3 (CH), 50.7 (CH_2), 47.9 (CH_2), 31.5 (CH, d, *J* = 130.9 Hz), peaks not in metafile; ^{31}P NMR (162 MHz, $CDCl_3$) δ 18.1; HRMS (ESI) Exact mass calculated for $C_{34}H_{30}NOP$ $[M+H]^+$: 500.2138, found: 500.2147.

2-Bromomethylbenzoxazole (S9)[10]

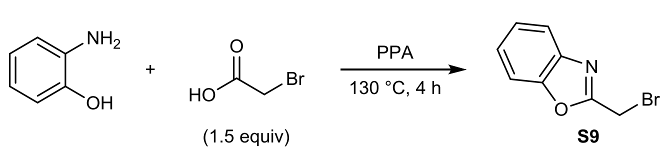

Prepared according to a modified literature procedure:[10] To a mixture of 2-aminophenol (2.50 g, 22.9 mmol) in polyphosphoric acid (22.9 g) was added bromoacetic acid (4.78 g, 34.4 mmol) and the resulting mixture was stirred at 130 °C for 4 h. The reaction was poured into ice water (1.0 L)

9. D. Bernier, A. J. Blake, S. Woodward, *J. Org. Chem.* **2008**, *73*, 4229-4232.
10. A. M. S. Soares, S. P. G. Costa, M. S. T. Gonçalves, *Tetrahedron* **2010**, *66*, 8189-8195.

and the mixture was stirred for 1 h to give a fine brown precipitate, which was collected by filtration. The solid was washed with cold water (2 x 100 mL), dissolved in CH_2Cl_2 (250 mL), and the solution was washed with saturated aqueous $NaHCO_3$ solution (2 x 250 mL). The organic layer was dried ($MgSO_4$), filtered, and concentrated *in vacuo* to leave 2-bromomethylbenzoxazole (**S9**) as a brown oil (3.49 g, 16.5 mmol, 72%). R_f = 0.33 (10% EtOAc/hexane); IR 3046, 2970, 1611, 1566, 1452, 1422, 1346, 1290, 1240, 1215, 1173, 1117, 1001, 951, 858, 837, 762, 746, 691, 592 cm^{-1}; ^{1}H NMR (500 MHz, $CDCl_3$) δ 7.74-7.68 (1H, m, Ar**H**), 7.54-7.48 (1H, m, Ar**H**), 7.38-7.29 (2H, m, Ar**H**), 4.57 (2H, s, C**H**$_2$); ^{13}C NMR (125.8 MHz, $CDCl_3$) δ 161.0 (C), 151.1 (C), 141.0 (C), 126.0 (CH), 124.8 (CH), 120.5 (CH), 110.8 (CH), 20.6 (CH_2); HRMS (EI) Exact mass calculated for $C_8H_6ON^{79}Br$ $[M]^+$: 210.9626, found: 210.9627.

2-Benzoxazolylmethyl triphenylphosphonium bromide (S10)

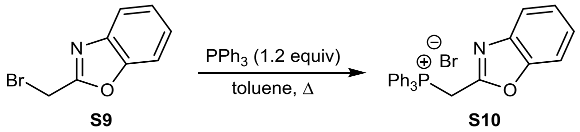

A solution of 2-bromomethylbenzoxazole (**S9**) (1.06 g, 5.00 mmol) and triphenylphosphine (1.57 g, 6.00 mmol) in toluene (50 mL) was heated to reflux for 2 h. The mixture was cooled to room temperature and the precipitate was collected by filtration and washed with toluene (2 x 25 mL) to leave the *phosphonium salt* **S10** as a pale yellow solid. (1.65 g, 84%). m.p. decomposes at ~90 °C; IR 3051, 2814, 2743, 1609, 1560, 1452, 1437, 1238, 1107, 995, 847, 748, 719, 689, 556 cm^{-1}; ^{1}H NMR (500 MHz, $CDCl_3$) δ 7.91 (6H, dd, J = 13.2, 7.8 Hz, Ar**H**), 7.78-7.71 (3H, m, Ar**H**), 7.67-7.60 (6H, m, Ar**H**), 7.50 (1H, app d, J = 6.8 Hz, Ar**H**), 7.34 (1H, app d, J = 7.3 Hz, Ar**H**), 7.29-7.20 (2H, m, Ar**H**), 6.07 (2H, d, J = 14.9 Hz, C**H**$_2$); ^{13}C NMR (125.8 MHz, $CDCl_3$) δ 155.8 (C), 150.9 (C), 140.5 (C, br s), 135.3 (3 x CH, d, J = 2.2 Hz), 134.1 (6 x CH, d, J = 10.6 Hz), 130.2 (6 x CH, d, J = 13.1 Hz), 125.7 (CH), 124.6 (CH), 119.9 (CH), 117.4 (3 x C, d, J = 88.2 Hz), 110.8 (CH), 26.9 (CH_2, d, J = 54.3 Hz); ^{31}P NMR (161.9 MHz, $CDCl_3$) δ 22.0; HRMS (EI) Exact mass calculated for $C_{26}H_{20}ON^{79}BrP$ $[M–H]^+$: 472.0460, found: 472.0460.

Representative Procedure for Preparation of Cyclization Precursors via Wittig reaction

2-Methyl-2-[(*E*)-5-(4-methylphenyl)-5-oxopent-3-en-1-yl]cyclopentane-1,3-dione (1b)

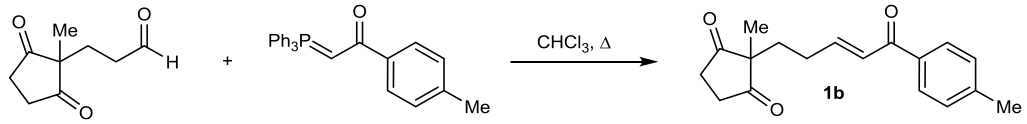

To a stirred solution of 3-(1-methyl-2,5-dioxocyclopentyl)propanal[11] (589 mg, 3.50 mmol) in $CHCl_3$ (12 mL) was added 1-(4-methylphenyl)-2-(triphenyl-λ^5-phosphanylidene)ethan-1-one[12] (1.66 g, 4.20 mmol) in one portion at room temperature and the resulting mixture was stirred under reflux for 16 h before being concentrated *in vacuo*. Purification of the residue by column chromatography (20 to 40% EtOAc/petroleum ether) gave the *enone* **1b** as an off-white solid (897 mg, 90%). R_f = 0.38 (40% EtOAc/petroleum ether); m.p. 76-77 °C (cyclohexane/CH_2Cl_2); IR 2924, 1717 (C=O), 1670 (C=O), 1620, 1603, 1308, 802 cm^{-1}; ^{1}H NMR (400 MHz, $CDCl_3$) δ 7.81 (2H, d, *J* = 8.2 Hz, Ar**H**), 7.26 (2H, d, *J* = 8.2 Hz, Ar**H**), 6.92-6.85 (2H, m, C**H**=C**H**), 2.88-2.68 (4H, m, COC**H**$_2$C**H**$_2$CO), 2.41 (3H, s, ArC**H**$_3$), 2.25-2.17 (2H, m, C**H**$_2$CH=), 1.91-1.82 (2H, m, C**H**$_2$CH$_2$CH=), 1.17 (3H, s, C**H**$_3$CC=O); ^{13}C NMR (100.6 MHz, $CDCl_3$) δ 215.9 (2 x C), 189.8 (C), 146.6 (CH), 143.6 (C), 135.0 (C), 129.2 (2 x CH), 128.6 (2 x CH), 126.6 (CH), 56.1 (C), 35.0 (2 x CH_2), 32.9 (CH_2), 27.8 (CH_2), 21.6 (CH_3), 19.8 (CH_3); HRMS (ESI) Exact mass calculated for $C_{18}H_{20}NaO_3$ $[M+Na]^+$: 307.1305, found: 307.1298.

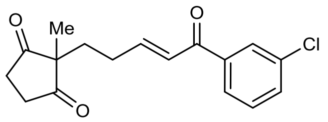

2-[(*E*)-5-(3-Chlorophenyl)-5-oxopent-3-en-1-yl]-2-methylcyclopentane-1,3-dione (1e). The title compound was prepared according to the Representative Procedure from 3-(1-methyl-2,5-dioxocyclopentyl)propanal[11] (505 mg, 3.00 mmol) and phosphorane **S4** (1.49 g, 3.60 mmol). Purification by column chromatography (20 to 30% EtOAc/petroleum ether) gave an orange solid (608 mg, 66%). R_f = 0.28 (30% EtOAc/petroleum ether); m.p. 56-57 °C (cyclohexane/CH_2Cl_2); IR 2932, 1714 (C=O), 1666 (C=O), 1621, 1570, 1419, 1253, 1208, 1079, 10338, 791, 727 cm^{-1}; ^{1}H NMR (400 MHz, $CDCl_3$) δ 7.86 (1H, t, *J* = 1.8 Hz, Ar**H**), 7.76 (1H, dt, *J* = 7.8, 1.2 Hz, Ar**H**), 7.53 (1H, ddd, *J* = 8.0, 2.1, 1.1 Hz, Ar**H**), 7.41 (1H, t, *J* = 7.8 Hz, Ar**H**), 6.91 (1H, dt, *J* = 15.4, 6.7 Hz, CH$_2$C**H**=), 6.77 (1H, dt, *J* = 15.4, 1.3 Hz, CH$_2$CH=C**H**), 2.95-2.66 (4H, m, COC**H**$_2$C**H**$_2$CO), 2.28-2.17 (2H, m, C**H**$_2$CH=), 1.93-1.81 (2H, m, C**H**$_2$CH$_2$CH=), 1.18 (3H, s, C**H**$_3$); ^{13}C NMR (100.6 MHz, $CDCl_3$) δ 215.8 (2 x C), 189.0 (C), 148.2 (CH), 139.2 (C), 134.9 (C), 132.7 (CH), 129.9 (CH), 128.6 (CH), 126.5 (CH), 126.2 (CH), 56.1 (C), 35.0 (2 x CH_2), 32.6 (CH_2), 27.8 (CH_2), 19.9

11. J. Deschamp, O. Riant, *Org. Lett.* **2009**, *11*, 1217-1220.
12. D. G. Stark, L. C. Morrill, P.-P. Yeh, A. M. Z. Slawin, T. J. C. O'Riordan, A. D. Smith, *Angew. Chem., Int. Ed.* **2013**, *52*, 11642-11646.

(CH_3); HRMS (ESI) Exact mass calculated for $C_{17}H_{17}ClNaO_3$ $[M+Na]^+$: 327.0758, found: 327.0751.

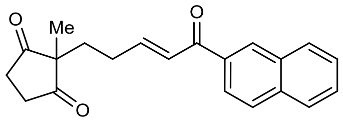

2-Methyl-2-[(*E*)-5-(naphthalen-2-yl)-5-oxopent-3-en-1-yl]cyclopentane-1,3-dione (1f). The title compound was prepared according to the Representative Procedure from 3-(1-methyl-2,5-dioxocyclopentyl)propanal[11] (589 mg, 3.50 mmol) and 1-(naphthalen-2-yl)-2-(triphenyl-λ^5-phosphanylidene)ethan-1-one[12] (1.81 g, 4.20 mmol). Purification by column chromatography (20 to 40% EtOAc/petroleum ether) gave a yellow solid (770 mg, 69%). R_f = 0.22 (30% EtOAc/petroleum ether); m.p. 83-84 °C (cyclohexane/CH_2Cl_2); IR 1717 (C=O), 1701, 1668 (C=O), 1652, 1646, 1507, 1457, 1178, 808 cm^{-1}; ^{1}H NMR (400 MHz, $CDCl_3$) δ 8.42 (1H, s, Ar**H**), 8.03-7.95 (2H, m, Ar**H**), 7.94-7.85 (2H, m, Ar**H**), 7.64-7.52 (2H, m, Ar**H**), 7.05-6.89 (2H, m, C**H**=C**H**), 2.94-2.68 (4H, m, COC**H**$_2$C**H**$_2$CO), 2.35-2.19 (2H, m, C**H**$_2$CH=), 1.98-1.86 (2H, m, C**H**$_2$CH$_2$CH=), 1.19 (3H, s, C**H**$_3$); ^{13}C NMR (100.6 MHz, $CDCl_3$) δ 215.9 (2 x C), 190.1 (C), 147.1 (CH), 135.4 (C), 134.9 (C), 132.5 (C), 130.0 (CH), 129.5 (CH), 128.5 (CH), 128.4 (CH), 127.8 (CH), 126.8 (CH), 126.6 (CH), 124.4 (CH), 56.1 (C), 35.0 (2 x CH_2), 32.9 (CH_2), 27.9 (CH_2), 19.9 (CH_3); HRMS (ESI) Exact mass calculated for $C_{21}H_{20}NaO_3$ $[M+Na]^+$: 343.1305, found: 343.1293.

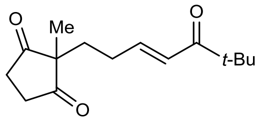

(*E*)-2-(6,6-Dimethyl-5-oxohept-3-en-1-yl)-2-methylcyclopentane-1,3-dione (1g). The title compound was prepared according to the Representative Procedure from 3-(1-methyl-2,5-dioxocyclopentyl)propanal[11] (700 mg, 4.20 mmol) and 3,3-dimethyl-1-(triphenyl-λ^5-phosphanylidene)butan-2-one[13] (1.62 g, 4.50 mmol). Purification by column chromatography (20% EtOAc/petroleum ether) gave a yellow solid (470 mg, 44%). R_f = 0.43 (30% EtOAc/petroleum ether); m.p. 49-50 °C ($CHCl_3$); IR 1765 (C=O), 1687 (C=O), 1624, 1508, 1477, 1367, 1239, 1152, 1077, 949 cm^{-1}; ^{1}H NMR (400 MHz, $CDCl_3$) δ 6.80-6.70 (1H, m, CH$_2$C**H**=), 6.73 (1H, dt, *J* = 15.2, 1.4 Hz, CH$_2$CH=C**H**), 2.88-2.67 (4H, m, COC**H**$_2$C**H**$_2$CO), 2.15-2.04 (2H, m, C**H**$_2$CH=), 1.82-1.76 (2H, m, C**H**$_2$CH$_2$CH=), 1.14 (3H, s, C**H**$_3$CC=O), 1.12 (9H, s, C(C**H**$_3$)$_3$); ^{13}C NMR (100.6 MHz, $CDCl_3$) δ 215.9 (2 x C) , 203.9 (C), 144.9 (CH), 124.9 (CH), 56.1 (C), 42.8 (C), 35.0 (2 x CH_2), 33.0 (CH_2), 27.4 (CH_2), 26.1 (3 x CH_3), 19.7 (CH_3); HRMS (ESI) Exact mass calculated for $C_{15}H_{22}NaO_3$ $[M+Na]^+$: 273.1461, found: 273.1470.

13. R. F. Al-Bashir, N. A. Al-Awadi, O. M. E. El-Dusouqui, *J. Phys. Org. Chem.* **2011**, *24*, 311-319.

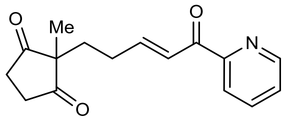

2-Methyl-2-[(*E*)-5-oxo-5-(pyridin-2-yl)pent-3-en-1-yl]cyclopentane-1,3-dione (1h). The title compound was prepared according to the Representative Procedure from 3-(1-methyl-2,5-dioxocyclopentyl)propanal[11] (505 mg, 3.00 mmol) and phosphorane **S5** (1.37 g, 3.60 mmol). Purification by column chromatography (20 to 40% EtOAc/petroleum ether) gave a brown solid (762 mg, 94%). R_f = 0.19 (40% EtOAc/petroleum ether); m.p. 107-109 °C (cyclohexane/CH_2Cl_2); IR 1718 (C=O), 1680 (C=O), 1619, 1179, 995, 754 cm^{-1}; ^1H NMR (500 MHz, $CDCl_3$) δ 8.69 (1H, ddd, *J* = 4.8, 1.7, 0.9 Hz, Ar**H**), 8.10 (1H, dt, *J* = 7.9, 1.0 Hz, Ar**H**), 7.85 (1H, td, *J* = 7.7, 1.7 Hz, Ar**H**), 7.54 (1H, dt, *J* = 15.7, 1.5 Hz, $CH_2CH=C\mathbf{H}$), 7.47 (1H, ddd, *J* = 7.6, 4.8, 1.2 Hz, Ar**H**), 7.04 (1H, dt, *J* = 15.7, 6.9 Hz, $CH_2C\mathbf{H}=$), 2.86-2.71 (4H, m, $COC\mathbf{H_2}C\mathbf{H_2}CO$), 2.30-2.22 (2H, m, $C\mathbf{H_2}CH=$), 1.95-1.88 (2H, m, $C\mathbf{H_2}CH_2CH=$), 1.17 (3H, s, $C\mathbf{H_3}$); ^{13}C NMR (125.8 MHz, $CDCl_3$) δ 216.0 (2 x C), 189.1 (C), 153.8 (C), 148.8 (CH), 147.5 (CH), 137.0 (CH), 126.9 (CH), 125.3 (CH), 122.9 (CH), 56.1 (C), 35.0 (2 x CH_2), 32.9 (CH_2), 27.9 (CH_2), 19.7 (CH_3); HRMS (ESI) Exact mass calculated for $C_{16}H_{17}NNaO_3$ $[M+Na]^+$: 294.1101, found: 294.1105.

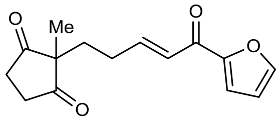

2-[(*E*)-5-(Furan-2-yl)-5-oxopent-3-en-1-yl]-2-methylcyclopentane-1,3-dione (1i). The title compound was prepared according to the Representative Procedure from 3-(1-methyl-2,5-dioxocyclopentyl)propanal[11] (505 mg, 3.00 mmol) and 1-(furan-2-yl)-2-(triphenyl-λ^5-phosphanylidene)ethan-1-one[14] (1.33 g, 3.60 mmol). Purification by column chromatography (10 to 40% EtOAc/petroleum ether) gave a yellow solid (582 mg, 74%). R_f = 0.25 (40% EtOAc/petroleum ether); m.p. 80-81 °C (cyclohexane/CH_2Cl_2); IR 1719 (C=O), 1667 (C=O), 1613, 1566, 1467, 1319, 1157, 1046, 884, 781 cm^{-1}; ^1H NMR (500 MHz, $CDCl_3$) δ 7.61 (1H, dd, *J* = 1.7, 0.7 Hz, Ar**H**), 7.22 (1H, dd, *J* = 3.6, 0.7 Hz, Ar**H**), 6.96 (1H, dt, *J* = 15.5, 6.9 Hz, $CH_2C\mathbf{H}=$), 6.73 (1H, dt, *J* = 15.5, 1.5 Hz, $CH_2CH=C\mathbf{H}$), 6.55 (1H, dd, *J* = 3.6, 1.7 Hz, Ar**H**), 2.89-2.67 (4H, m, $COC\mathbf{H_2}C\mathbf{H_2}CO$), 2.24-2.16 (2H, m, $C\mathbf{H_2}CH=$), 1.90-1.81 (2H, m, $C\mathbf{H_2}CH_2CH=$), 1.16 (3H, s, $C\mathbf{H_3}$); ^{13}C NMR (125.8 MHz, $CDCl_3$) δ 215.9 (2 x C), 177.7 (C), 153.1 (C), 146.6 (CH), 146.5 (CH), 125.7 (CH), 117.7 (CH), 112.4 (CH), 56.1 (C), 35.0 (2 x CH_2), 32.7 (CH_2), 27.6 (CH_2), 19.8 (CH_3); HRMS (ESI) Exact mass calculated for $C_{15}H_{16}NaO_4$ $[M+Na]^+$: 283.0941, found: 283.0932.

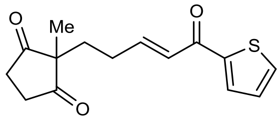

2-Methyl-2-[(*E*)-5-oxo-5-(thiophen-2-yl)pent-3-en-1-yl]cyclopentane-1,3-dione (1j). The title compound was prepared according to the Representative Procedure from 3-(1-methyl-2,5-

14. S. J. Sabounchei, V. Jodaian, S. Salehzadeh, S. Samiee, A. Dadrass, M. Bayat, H. R. Khavasi, *Helv. Chim. Acta* **2010**, *93*, 1105-1119.

dioxocyclopentyl)propanal[11] (505 mg, 3.00 mmol) and phosphorane **S6** (1.39 g, 3.60 mmol). Purification by column chromatography (20 to 40% EtOAc/petroleum ether) gave a pale yellow solid (553 mg, 67%). R_f = 0.26 (40% EtOAc/petroleum ether); m.p. 80-82 °C (cyclohexane/CH_2Cl_2); IR 2934, 1716 (C=O), 1660 (C=O), 1607, 1415, 1274, 1229, 953, 752 cm^{-1}; ^{1}H NMR (400 MHz, $CDCl_3$) δ 7.74 (1H, dd, *J* = 3.8, 1.1 Hz, Ar**H**), 7.66 (1H, dd, *J* = 4.9, 1.1 Hz, Ar**H**), 7.15 (1H, dd, *J* = 4.9, 3.8 Hz, Ar**H**), 6.94 (1H, dt, *J* = 15.3, 6.9 Hz, $CH_2C\mathbf{H}$=), 6.74 (1H, dt, *J* = 15.3, 1.4 Hz, $CH_2CH=C\mathbf{H}$), 2.92-2.67 (4H, m, $COC\mathbf{H}_2C\mathbf{H}_2CO$), 2.27-2.17 (2H, m, $C\mathbf{H}_2CH$=), 1.92-1.83 (2H, m, $C\mathbf{H}_2CH_2CH$=), 1.18 (3H, s, $C\mathbf{H}_3$); ^{13}C NMR (100.6 MHz, $CDCl_3$) δ 215.9 (2 x C), 181.8 (C), 146.4 (CH), 144.8 (C), 133.9 (CH), 132.0 (CH), 128.2 (CH), 126.2 (CH), 56.1 (C), 35.0 (2 x CH_2), 32.8 (CH_2), 27.7 (CH_2), 19.9 (CH_3); HRMS (ESI) Exact mass calculated for $C_{15}H_{16}NaO_3S$ $[M+Na]^+$: 299.0712, found: 299.0706.

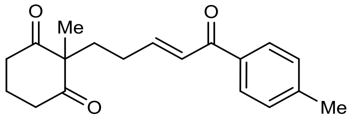

2-Methyl-2-[(*E*)-5-(4-methylphenyl)-5-oxopent-3-en-1-yl]cyclohexane-1,3-dione (5b). The title compound was prepared according to the Representative Procedure from 3-(1-methyl-2,6-dioxocyclohexyl)propanal[11] (638 mg, 3.50 mmol) and 1-(4-methylphenyl)-2-(triphenyl-λ^5-phosphanylidene)ethan-1-one (1.66 g, 4.20 mmol). Purification by column chromatography (10 to 40% EtOAc/petroleum ether) gave a yellow solid (793 mg, 76%). R_f = 0.44 (40% EtOAc/petroleum ether); m.p. 69-70 °C (cyclohexane/EtOAc); IR 2960, 1690 (C=O), 1665 (C=O), 1617, 1604, 1424, 1302, 1182, 1030, 812 cm^{-1}; ^{1}H NMR (400 MHz, $CDCl_3$) δ 7.85-7.80 (2H, m, Ar**H**), 7.29-7.23 (2H, m, Ar**H**), 6.93 (1H, dt, *J* = 15.4, 6.3 Hz, $CH_2C\mathbf{H}$=), 6.85 (1H, dt, *J* = 15.4, 1.1 Hz, $CH_2CH=C\mathbf{H}$), 2.76-2.61 (4H, m, $C\mathbf{H}_2CH_2C\mathbf{H}_2$), 2.41 (3H, s, $ArC\mathbf{H}_3$), 2.19-2.11 (2H, m, $C\mathbf{H}_2CH$=), 2.04-1.92 (4H, m, $CH_2C\mathbf{H}_2CH_2$ and $C\mathbf{H}_2CH_2CH$=), 1.31 (3H, s, $C\mathbf{H}_3CC=O$); ^{13}C NMR (100.6 MHz, $CDCl_3$) δ 209.9 (2 x C), 190.0 (C), 147.2 (CH), 143.5 (C), 135.1 (C), 129.2 (2 x CH), 128.6 (2 x CH), 126.3 (CH), 64.9 (C), 37.9 (2 x CH_2), 34.3 (CH_2), 28.1 (CH_2), 21.6 (CH_3), 21.1 (CH_3), 17.5 (CH_2); HRMS (ESI) Exact mass calculated for $C_{19}H_{22}NaO_3$ $[M+Na]^+$: 321.1461, found: 321.1444.

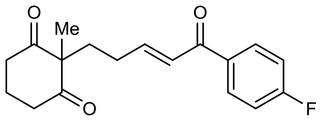

2-[(*E*)-5-(4-Fluorophenyl)-5-oxopent-3-en-1-yl]-2-methylcyclohexane-1,3-dione (5d). The title compound was prepared according to a modification of the Representative Procedure from 3-(1-methyl-2,6-dioxocyclohexyl)propanal[11] (1.20 g, 6.60 mmol) and 1-(4-fluorophenyl)-2-(triphenyl-λ-5-phosphanylidene)ethan-1-one[15] (3.10 g, 7.90 mmol) using toluene (60 mL) as solvent and by heating to 90 °C for 14 h. Purification by column chromatography (20 to 40% EtOAc/petroleum

15. E. Venkateswararao, M.-S. Kim, V. K. Sharma, K.-C. Lee, S. Subramanian, E. Roh, Y. Kim, S.-H. Jung, *Eur. J. Med. Chem.* **2013**, *59*, 31-38.

ether) gave a pale yellow solid (1.10 g, 55%). R_f = 0.27 (40% EtOAc/petroleum ether); m.p. 123-125 °C (CH_2Cl_2); IR 2936, 2860, 1727 (C=O), 1696 (C=O), 1671, 1622, 1599, 1507, 1486, 1306, 1240, 988 cm^{-1}; ^{1}H NMR (400 MHz, $CDCl_3$) δ 7.96-7.85 (2H, m, Ar**H**), 7.15-7.04 (2H, m, Ar**H**), 6.92 (1H, dt, *J* = 15.4, 6.3 Hz, CH_2C**H**=), 6.56 (1H, dt, *J* = 15.4, 1.0 Hz, CH_2CH=C**H**), 2.76-2.56 (4H, m, C**H**$_2CH_2C$**H**$_2$), 2.18-2.06 (2H, m, C**H**$_2$CH=), 2.02-1.88 (4H, m, C**H**$_2CH_2$CH= and CH_2C**H**$_2CH_2$), 1.28 (3H, s, C**H**$_3$); ^{13}C NMR (100.6 MHz, $CDCl_3$) δ 209.8 (2 x C), 188.7 (C), 165.4 (C, d, *J* = 253.4 Hz), 148.0 (CH), 133.9 (C, d, *J* = 3.0 Hz), 131.1 (2 x CH, d, *J* = 9.2 Hz), 125.8 (CH), 115.5 (2 x CH, d, *J* = 21.8 Hz,), 64.8 (C), 37.9 (2 x CH_2), 33.9 (CH_2), 28.0 (CH_2), 21.4 (CH_3), 17.4 (CH_2); ^{19}F NMR (282 MHz, $CDCl_3$) δ −105.7; HRMS (ESI) Exact mass calculated for $C_{18}H_{19}FNaO_3$ $[M+Na]^+$: 303.1377, found: 303.1391.

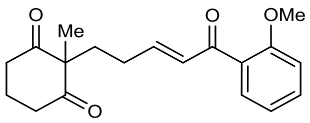

2-[(*E*)-5-(2-methoxyphenyl)-5-oxopent-3-en-1-yl]-2-methylcyclohexane-1,3-dione (5h). The title compound was prepared according to a modification of the Representative Procedure from 3-(1-methyl-2,6-dioxocyclohexyl)propanal[11] (455 mg, 2.50 mmol) and 1-(2-methoxyphenyl)-2-(triphenyl-λ^5-phosphanylidene)ethan-1-one[16] (1.23 g, 3.00 mmol), using toluene (30 mL) as solvent and by heating to 90 °C for 16 h. Purification by column chromatography (30 to 40% EtOAc/petroleum ether) gave a yellow oil (504 mg, 66%). R_f = 0.25 (40% EtOAc/petroleum ether); IR 2943, 2841, 1726 (C=O), 1696 (C=O), 1662, 1617, 1599, 1376, 1286, 1025 cm^{-1}; ^{1}H NMR (400 MHz, $CDCl_3$) δ 7.42-7.30 (2H, m, Ar**H**), 6.94-6.85 (2H, m, Ar**H**), 6.64 (1H, dt, *J* = 15.6, 6.3 Hz, CH_2C**H**=), 6.56 (1H, app d, *J* = 15.6 Hz, CH_2CH=C**H**), 3.76 (3H, s, OC**H**$_3$), 2.58 (4H, t, *J* = 6.9 Hz, C**H**$_2CH_2C$**H**$_2$), 2.05-1.96 (2H, m, C**H**$_2$CH=), 1.91-1.81 (4H, m, CH_2C**H**$_2CH_2$ and C**H**$_2CH_2$CH=), 1.19 (3H, s, CC**H**$_3$); ^{13}C NMR (100.6 MHz, $CDCl_3$) δ 209.7 (2 x C), 192.9 (C), 157.5 (C), 146.5 (CH), 132.4 (CH), 130.8 (CH), 129.7 (CH), 128.6 (C), 120.2 (CH), 111.3 (CH), 64.6 (C), 55.3 (CH_3), 37.6 (2 x CH_2), 34.0 (CH_2), 27.6 (CH_2), 20.7 (CH_3), 17.2 (CH_2); HRMS (ESI) Exact mass calculated for $C_{19}H_{23}O_4$ $[M+H]^+$: 315.1588, found: 315.1591.

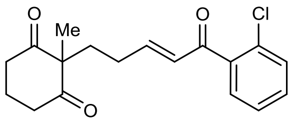

2-[(*E*)-5-(2-Chlorophenyl)-5-oxopent-3-en-1-yl]-2-methylcyclohexane-1,3-dione (5i). The title compound was prepared according to the Representative Procedure from 3-(1-methyl-2,6-dioxocyclohexyl)propanal (680 mg, 3.73 mmol)[11] and phosphorane **S7** (1.86 g, 4.48 mmol). Purification by column chromatography (20 to 40% EtOAc/petroleum ether) gave an orange oil (482 mg, 41%). R_f = 0.33 (40% EtOAc/petroleum ether); IR 2961, 1725 (C=O), 1693 (C=O), 1658, 1618, 1432, 1301, 1026,

16. A. Kermagoret, P. Braunstein, *Dalton Trans.* **2008**, 822-831.

765, 739 cm^{-1}; ^{1}H NMR (400 MHz, $CDCl_3$) δ 7.43-7.36 (2H, m, Ar**H**), 7.35-7.28 (2H, m, Ar**H**), 6.59 (1H, dt, *J* = 15.8, 6.7 Hz, CH_2C**H**=), 6.41 (1H, dt, *J* = 15.8, 1.4 Hz, CH_2CH=C**H**), 2.75-2.57 (4H, m, C**H**$_2$$CH_2$C**H**$_2$), 2.15-2.05 (2H, m, C**H**$_2$CH=), 2.03-1.85 (4H, m, CH_2C**H**$_2$$CH_2$, C**H**$_2$$CH_2$CH=), 1.28 (3H, s, C**H**$_3$); ^{13}C NMR (100.6 MHz, $CDCl_3$) δ 209.9 (2 x C), 194.1 (C), 150.7 (CH), 138.8 (C), 131.2 (CH), 131.1 (C), 130.7 (CH), 130.2 (CH), 129.1 (CH), 126.7 (CH), 64.8 (C), 38.0 (2 x CH_2), 33.6 (CH_2), 28.2 (CH_2), 21.9 (CH_3), 17.5 (CH_2); HRMS (ESI) Exact mass calculated for $C_{18}H_{19}ClNaO_3$ $[M+Na]^+$: 341.0915, found: 341.0897.

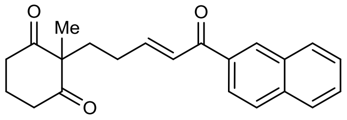

2-Methyl-2-[(*E*)-5-(naphthalen-2-yl)-5-oxopent-3-en-1-yl]cyclohexane-1,3-dione (5j). The title compound was prepared according to the Representative Procedure from 3-(1-methyl-2,6-dioxocyclohexyl)propanal[11] (638 mg, 3.50 mmol) and 1-(naphthalen-2-yl)-2-(triphenyl-λ^5-phosphanylidene)ethan-1-one (1.81 g, 4.20 mmol). Purification by column chromatography (20 to 40% EtOAc/petroleum ether) gave a yellow solid (695 mg, 59%). R_f = 0.38 (40% EtOAc/petroleum ether); m.p. 88-89 °C (cyclohexane/EtOAc); IR 2961, 1723 (C=O), 1692 (C=O), 1667, 1617, 1465, 1323, 1277, 1192, 1124, 1029, 817 cm^{-1}; ^{1}H NMR (400 MHz, $CDCl_3$) δ 8.43 (1H, s, Ar**H**), 8.01 (1H, dd, *J* = 8.6, 1.7 Hz, Ar**H**), 7.98 (1H, app d, *J* = 8.0 Hz, Ar**H**), 7.94-7.86 (2H, m, Ar**H**), 7.64-7.53 (2H, m, Ar**H**), 7.08-6.96 (2H, m, C**H**=C**H**), 2.78-2.63 (4H, m, C**H**$_2$$CH_2$C**H**$_2$), 2.26-2.17 (2H, m, C**H**$_2$CH=), 2.09-2.02 (2H, m, C**H**$_2$$CH_2$CH=), 1.98 (2H, quintet, *J* = 6.8 Hz, CH_2C**H**$_2$$CH_2$), 1.33 (3H, s, C**H**$_3$); ^{13}C NMR (100.6 MHz, $CDCl_3$) δ 209.9 (2 x C), 190.3 (C), 147.7 (CH), 135.4 (C), 135.0 (C), 132.5 (C), 130.0 (CH), 129.5 (CH), 128.5 (CH), 128.3 (CH), 127.8 (CH), 126.7 (CH), 126.3 (CH), 124.4 (CH), 64.9 (C), 38.0 (2 x CH_2), 34.2 (CH_2), 28.2 (CH_2), 21.3 (CH_3), 17.5 (CH_2); HRMS (ESI) Exact mass calculated for $C_{22}H_{22}NaO_3$ $[M+Na]^+$: 357.1461, found: 357.1450.

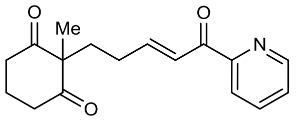

2-Methyl-2-[(*E*)-5-oxo-5-(pyridin-2-yl)pent-3-en-1-yl]cyclohexane-1,3-dione (5k). The title compound was prepared according to the Representative Procedure from 3-(1-methyl-2,6-dioxocyclohexyl)propanal[11] (547 mg, 3.00 mmol) and phosphorane **S5** (1.30 g, 3.41 mmol). Purification by column chromatography (20 to 40 to 50% EtOAc/petroleum ether) gave a grey solid (608 mg, 71%). R_f = 0.22 (40% EtOAc/petroleum ether); m.p. 73-74 °C (cyclohexane/EtOAc); IR 2931, 1722, 1688 (C=O), 1675 (C=O), 1627, 1582, 1329, 1220, 1136, 1036, 996, 785, 744 cm^{-1}; ^{1}H NMR (400 MHz, $CDCl_3$) δ 8.70 (1H, ddd, *J* = 4.8, 1.7, 0.9 Hz, Ar**H**), 8.11 (1H, dt, *J* = 7.9, 1.0 Hz, Ar**H**), 7.85 (1H, td, *J* = 7.7, 1.7 Hz, Ar**H**), 7.57 (1H, dt, *J* = 15.7, 1.5 Hz, CH_2CH=C**H**), 7.47 (1H, ddd, *J* = 7.6, 4.8, 1.2 Hz, Ar**H**), 7.13 (1H, dt, *J* = 15.7, 6.7 Hz, CH_2C**H**=), 2.69 (4H, app t, *J* = 6.6 Hz, C**H**$_2$$CH_2$C**H**$_2$), 2.25-2.16 (2H, m, C**H**$_2$CH=), 2.09-2.02 (2H, m, C**H**$_2$$CH_2$CH=), 2.01-1.89 (2H,

m, $CH_2CH_2CH_2$), 1.31 (3H, s, C**H**$_3$); ^{13}C NMR (100.6 MHz, $CDCl_3$) δ 209.9 (2 x C), 189.3 (C), 154.0 (C), 148.8 (CH), 148.0 (CH), 137.0 (CH), 126.8 (CH), 125.0 (CH), 122.9 (CH), 65.0 (C), 38.0 (2 x CH_2), 34.5 (CH_2), 28.1 (CH_2), 20.5 (CH_3), 17.6 (CH_2); HRMS (ESI) Exact mass calculated for $C_{17}H_{19}NNaO_3$ [M+Na]$^+$: 308.1257, found: 308.1245.

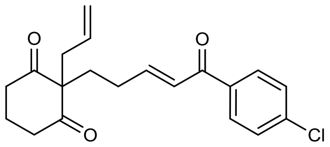

2-[(*E*)-5-(4-Chlorophenyl)-5-oxopent-3-en-1-yl]-2-(prop-2-en-1-yl)cyclohexane-1,3-dione (5n). The title compound was prepared according to the Representative Procedure from 3-[2,6-dioxo-1-(prop-2-en-1-yl)cyclohexyl]propanal[11] (194 mg, 0.93 mmol) and 1-(4-chlorophenyl)-2-(triphenylphosphoranylidene)ethanone[17] (464 mg, 1.12 mmol). Purification by column chromatography (20 to 30% EtOAc/petroleum ether) gave a pale yellow solid (180 mg, 56%). R_f = 0.46 (40% EtOAc/petroleum ether); m.p. 57-59 °C (CH_2Cl_2); IR 2928, 1715, 1688 (C=O), 1673 (C=O), 1619, 1584, 1440, 1331, 1293, 1215, 1089, 1007, 928, 800 cm^{-1}; ^{1}H NMR (400 MHz, $CDCl_3$) δ 7.85 (2H, d, *J* = 8.5 Hz, Ar**H**), 7.44 (2H, d, *J* = 8.5 Hz, Ar**H**), 6.94 (1H, dt, *J* = 15.4, 6.4 Hz, CH_2C**H**=CH), 6.79 (1H, app d, *J* = 15.4 Hz, CH_2CH=C**H**), 5.69-5.49 (1H, m, CH_2C**H**=CH_2), 5.18-5.00 (2H, m, CH_2CH=C**H**$_2$), 2.75-2.56 (4H, m, C**H**$_2$$CH_2$C**H**$_2$), 2.54 (2H, d, *J* = 7.4 Hz, C**H**$_2$CH=CH_2), 2.17-1.95 (5H, m, C**H**$_2$C**H**$_2$CH= and CH_2C**H**$_\mathbf{A}$$H_B$$CH_2$), 1.94-1.81 (1H, m, $CH_2$$CH_A$**H**$_\mathbf{B}$$CH_2$); ^{13}C NMR (100.6 MHz, $CDCl_3$) δ 209.8 (2 x C), 189.3 (C), 148.5 (CH), 139.1 (C), 136.1 (C), 131.7 (CH), 129.9 (2 x CH), 128.9 (2 x CH), 125.9 (CH), 119.8 (CH_2), 68.3 (C), 41.8 (CH_2), 39.4 (2 x CH_2), 32.6 (CH_2), 28.3 (CH_2), 16.9 (CH_2); HRMS (ESI) Exact mass calculated for $C_{20}H_{21}ClNaO_3$ [M+Na]$^+$: 367.1071, found: 367.1056.

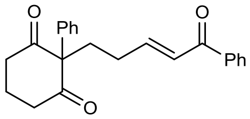

2-[(*E*)-5-Oxo-5-phenylpent-3-en-1-yl]-2-phenylcyclohexane-1,3-dione (5o). The title compound was prepared according to a modification of the Representative Procedure from aldehyde **S2** (300 mg, 1.30 mmol) and 1-phenyl-2-(triphenyl-λ^5-phosphanylidene)ethan-1-one (570 mg, 1.50 mmol) using toluene (30 mL) as solvent and by heating to 90 °C for 14 h. Purification by column chromatography (15% EtOAc/toluene) gave a yellow oil (110 mg, 25%). R_f = 0.68 (40 % EtOAc/petroleum ether); IR 2934, 2870, 1728 (C=O), 1698 (C=O), 1670, 1620, 1599, 1494, 1312, 1267, 1025 cm^{-1}; ^{1}H NMR (300 MHz, $CDCl_3$) δ 7.91-7.85 (2H, m, Ar**H**), 7.57-7.50 (1H, m, Ar**H**), 7.48-7.29 (5H, m, Ar**H**), 7.06-6.92 (3H, m, 2 x Ar**H** and CH_2C**H**=), 6.73 (1H, app d, *J* = 15.4 Hz, CH_2CH=C**H**), 2.85-2.72 (2H, m, C**H**$_\mathbf{A}$$H_B$$CH_2$C**H**$_\mathbf{A}$$H_B$), 2.63-2.52 (2H, m, CH_A**H**$_\mathbf{B}$$CH_2$$CH_A$**H**$_\mathbf{B}$), 2.19-2.15 (4H, m, C**H**$_2$C**H**$_2$CH=), 1.98-1.83 (1H, m, CH_2C**H**$_\mathbf{A}$$H_B$$CH_2$), 1.79-1.65 (1H, m, $CH_2$$CH_A$**H**$_\mathbf{B}$$CH_2$); ^{13}C NMR

17. D. Belmessieri, L. C. Morrill, C. Simal, A. M. Z. Slawin, A. D. Smith, *J. Am. Chem. Soc.* **2011**, *133*, 2714-2720.

(75 MHz, $CDCl_3$) δ 207.2 (2 x C), 191.0 (C), 149.5 (CH), 137.9 (C), 137.5 (C), 132.5 (CH), 129.7 (2 x CH), 128.5 (2 x CH), 128.4 (2 x CH), 128.1 (CH), 126.5 (2 x CH), 125.8 (CH), 75.3 (C), 39.1 (2 x CH_2), 33.2 (CH_2), 28.9 (CH_2), 17.5 (CH_2); HRMS (ESI) Exact mass calculated for $C_{23}H_{22}NaO_3$ $[M+Na]^+$: 369.1461, found: 369.1454.

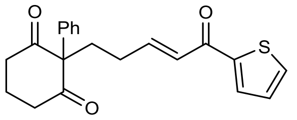

2-[(*E*)-5-Oxo-5-(thiophen-2-yl)pent-3-en-1-yl]-2-phenylcyclohexane-1,3-dione (5p). The title compound was prepared according to a modification of the Representative Procedure from aldehyde **S2** (300 mg, 1.30 mmol) and phosphorane **S6** (475 mg, 1.30 mmol) using toluene (30 mL) as solvent and by heating at 90 °C for 14 h. Purification by column chromatography (20% EtOAc/petroleum ether) gave a yellow oil (260 mg, 55%). R_f = 0.59 (40% EtOAc/petroleum ether); IR 3011, 2414, 1728 (C=O), 1698 (C=O), 1659, 1614, 1517, 1418, 1235, 976, 660 cm^{-1}; ^1H NMR (500 MHz, $CDCl_3$) δ 7.73 (1H, dd, *J* = 3.8, 1.1 Hz, Ar**H**), 7.62 (1H, dd, *J* = 4.9, 1.1 Hz, Ar**H**), 7.43-7.28 (3H, m, Ar**H**), 7.13 (1H, dd, *J* = 4.9, 3.8 Hz, Ar**H**), 7.07-7.00 (3H, m, 2 x Ar**H** and $CH_2C\mathbf{H}$=), 6.73 (1H, app d, *J* = 15.2 Hz, $CH_2CH=C\mathbf{H}$), 2.86-2.72 (2H, m, $C\mathbf{H_A}H_BCH_2C\mathbf{H_A}H_B$), 2.66-2.49 (2H, m, $CH_A\mathbf{H_B}CH_2CH_A\mathbf{H_B}$), 2.21-2.12 (4H, m, $C\mathbf{H_2}C\mathbf{H_2}CH$=), 1.98-1.82 (1H, m, $CH_2C\mathbf{H_A}H_BCH_2$), 1.79-1.66 (1H, m, $CH_2CH_A\mathbf{H_B}CH_2$); ^{13}C NMR (125.8 MHz, $CDCl_3$) δ 207.2 (2 x C), 182.3 (C), 148.5 (CH), 145.1 (C), 137.5 (C), 133.5 (CH), 131.8 (CH), 129.7 (2 x CH), 128.1 (CH), 128.0 (CH), 126.5 (2 x CH), 125.1 (CH), 75.3 (C), 39.1 (2 x CH_2), 33.2 (CH_2), 28.8 (CH_2), 17.5 (CH_2); HRMS (ESI) Exact mass calculated for $C_{21}H_{20}NaO_3S$ $[M+Na]^+$: 375.1025, found: 375.1014.

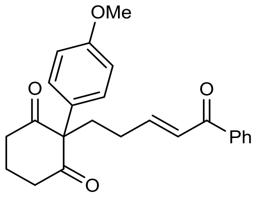

2-(4-Methoxyphenyl)-2-[(*E*)-5-oxo-5-phenylpent-3-en-1-yl]cyclohexane-1,3-dione (5q). The title compound was prepared according to a modification of the Representative Procedure from aldehyde **S3** (215 mg, 0.80 mmol) and 1-phenyl-2-(triphenyl-λ^5-phosphanylidene)ethan-1-one (387 mg, 1.00 mmol) using toluene (30 mL) as solvent and by heating at 90 °C for 14 h. Purification by column chromatography (20% EtOAc/toluene) gave a yellow oil (143 mg, 48%). R_f = 0.51 (40% EtOAc/petroleum ether); IR 2960, 2939, 1727 (C=O), 1697 (C=O), 1669, 1648, 1511, 1295, 1255, 1033, 832 cm^{-1}; ^1H NMR (500 MHz, $CDCl_3$) δ 7.91-7.85 (2H, m, Ar**H**), 7.56-7.51 (1H, m, Ar**H**), 7.47-7.42 (2H, m, Ar**H**), 7.00-6.92 (3H, m, 2 x Ar**H** and $CH_2C\mathbf{H}$=), 6.91-6.87 (2H, m, Ar**H**), 6.79 (1H, dt, *J* = 15.5, 1.3 Hz, $CH_2CH=C\mathbf{H}$), 3.79 (3H, s, $C\mathbf{H_3}$), 2.82-2.74 (2H, m, $C\mathbf{H_A}H_BCH_2C\mathbf{H_A}H_B$), 2.58-2.51 (2H, m, $CH_A\mathbf{H_B}CH_2CH_A\mathbf{H_B}$), 2.20-2.11 (4H, m, $C\mathbf{H_2}C\mathbf{H_2}CH$=), 1.94-1.86 (1H, m, $CH_2C\mathbf{H_A}H_BCH_2$), 1.77-1.67 (1H, m, $CH_2CH_A\mathbf{H_B}CH_2$); ^{13}C NMR (125.8 MHz, $CDCl_3$) δ 207.4 (2 x C), 191.0 (C), 159.3 (C), 149.6 (CH), 138.0 (C), 132.5 (CH), 129.3 (C), 128.5 (2 x CH), 128.4 (2 x CH), 127.8 (2 x CH), 125.7 (CH), 115.0 (2 x CH), 74.6 (C), 55.3 (CH_3), 39.0 (2 x CH_2), 33.2 (CH_2),

29.0 (CH_2), 17.4 (CH_2); HRMS (ESI) Exact mass calculated for $C_{24}H_{24}NaO_4$ $[M+Na]^+$: 399.1567, found: 399.1564.

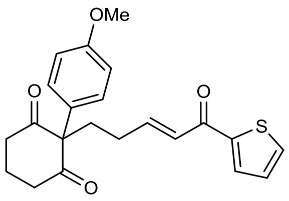

2-(4-Methoxyphenyl)-2-[(*E*)-5-oxo-5-(thiophen-2-yl)pent-3-en-1-yl]cyclohexane-1,3-dione (5r). The title compound was prepared according to a modification of the Representative Procedure from aldehyde **S3** (400 mg, 1.50 mmol) and phosphorane **S6** (732 mg, 1.90 mmol) using toluene (20 mL) as solvent and by heating at 90 °C for 14 h. Purification by column chromatography (15% acetone/petroleum ether) gave a yellow oil (440 mg, 79%). R_f = 0.45 (40 % EtOAc/petroleum ether); IR 2960, 2840, 1727 (C=O), 1698 (C=O), 1659, 1610, 1511, 1417, 1255, 976, 832 cm^{-1}; ^1H NMR (500 MHz, $CDCl_3$) δ 7.72 (1H, dd, *J* = 3.8, 1.1 Hz, Ar**H**), 7.61 (1H, dd, *J* = 4.9, 1.1 Hz, Ar**H**), 7.12 (1H, dd, *J* = 4.9, 3.8 Hz, Ar**H**), 7.07-6.98 (1H, m, $CH_2C\mathbf{H}$=), 6.95-6.86 (4H, m, Ar**H**), 6.73 (1H, app d, *J* = 15.3 Hz, $CH_2CH=C\mathbf{H}$), 3.77 (3H, s, $C\mathbf{H}_3$), 2.83-2.72 (2H, m, $C\mathbf{H_A}H_BCH_2C\mathbf{H_A}H_B$), 2.58-2.50 (2H, m, $CH_A\mathbf{H_B}CH_2CH_A\mathbf{H_B}$), 2.20-2.09 (4H, m, $C\mathbf{H}_2C\mathbf{H}_2CH$=), 1.95-1.83 (1H, m, $CH_2C\mathbf{H_A}H_BCH_2$), 1.77-1.65 (1H, m, $CH_2CH_A\mathbf{H_B}CH_2$); ^{13}C NMR (100.6 MHz, $CDCl_3$) δ 207.4 (2 x C), 182.3 (C), 159.3 (C), 148.6 (CH), 145.1 (C), 133.4 (CH), 131.8 (CH), 129.3 (C), 128.0 (CH), 127.8 (2 x CH), 125.0 (CH), 115.0 (2 x CH), 74.5 (C), 55.3 (CH_3), 39.0 (2 x CH_2), 33.1 (CH_2), 28.8 (CH_2), 17.4 (CH_2); HRMS (ESI) Exact mass calculated for $C_{22}H_{22}NaO_4S$ $[M+Na]^+$: 405.1131, found: 405.1119.

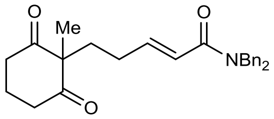

(*E*)-*N,N*-Dibenzyl-5-(1-methyl-2,6-dioxocyclohexyl)pent-2-enamide (8). The title compound was prepared according to the Representative Procedure from 3-(1-methyl-2,6-dioxocyclohexyl)propanal[11] (1.64 g, 9.00 mmol) and phosphorane **S8** (4.99 g, 10.0 mmol). Purification by column chromatography (35% EtOAc/petroleum ether) gave a pale yellow solid (2.20 g, 60%). R_f = 0.18 (40% EtOAc/petroleum ether); m.p. 79-80 °C (CH_2Cl_2); IR 2927, 1721 (C=O), 1689 (C=O), 1653, 1607, 1442, 1424, 1216, 1028, 749, 699 cm^{-1}; ^1H NMR (400 MHz, $CDCl_3$) δ 7.44-7.19 (8H, m, Ar**H**), 7.16 (2H, d, *J* = 7.2 Hz, Ar**H**), 6.93 (1H, dt, *J* = 14.9, 6.8 Hz, $CH_2C\mathbf{H}$=), 6.25 (1H, dt, *J* = 14.9, 1.3 Hz, $CH_2CH=C\mathbf{H}$), 4.63 (2H, s, $C\mathbf{H}_2Ph$), 4.49 (2H, s, $C\mathbf{H}_2Ph$), 2.63 (4H, t, *J* = 6.8 Hz, $C\mathbf{H}_2CH_2C\mathbf{H}_2$), 2.07-1.97 (2H, m, $C\mathbf{H}_2CH$=), 1.96-1.84 (4H, m, $C\mathbf{H}_2CH_2CH$= and $CH_2C\mathbf{H}_2CH_2$), 1.24 (3H, s, $C\mathbf{H}_3$); ^{13}C NMR (100.6 MHz, $CDCl_3$) δ 209.8 (2 x C), 166.7 (C), 145.8 (CH), 137.2 (C), 136.5 (C), 128.8 (2 x CH), 128.5 (2 x CH), 128.2 (2 x CH), 127.6 (CH), 127.3 (CH), 126.5 (2 x CH), 120.9 (CH), 64.9 (C), 49.8 (CH_2), 48.4 (CH_2), 37.8 (2 x CH_2), 34.7 (CH_2), 27.7 (CH_2), 20.3 (CH_3), 17.5 (CH_2); HRMS (ESI) Exact mass calculated for $C_{26}H_{29}NNaO_3$ $[M+Na]^+$: 426.2040, found: 426.2043.

2-[(*E*)-4-Benzoxazol-2-yl-but-3-enyl]-2-methylcyclohexane-1,3-dione (10)

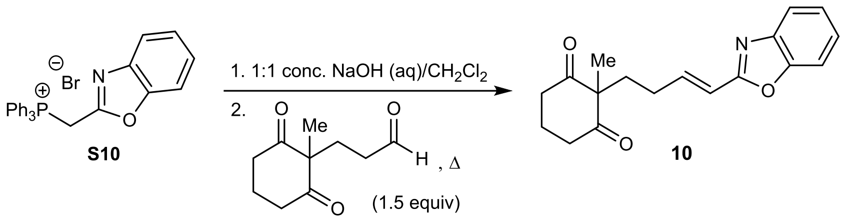

To a solution of the phosphonium bromide **S10** (470 mg, 1.00 mmol) in CH_2Cl_2 (10 mL) at room temperature was added concentrated aqueous NaOH solution (10 mL) and the resulting mixture was stirred for 15 min. The aqueous layer was separated and extracted with CH_2Cl_2 (10 mL). The combined organic layers were dried ($MgSO_4$) and filtered before 3-(1-methyl-2,6-dioxocyclohexyl)propanal[11] (273 mg, 1.50 mmol) was added to the resulting solution which was then heated to reflux for 12 h. The mixture was cooled to room temperature, dried ($MgSO_4$), filtered, and concentrated *in vacuo*. Purification of the residue by column chromatography (5% EtOAc/hexane) gave the *alkenylbenzoxazole* **10** as a colorless oil (181 mg, 61%). R_f = 0.29 (20% EtOAc/hexane); IR 2930, 1724, 1692 (C=O), 1659, 1537, 1454, 1427, 1242, 1177, 1026, 966, 851, 762, 746, 623 cm^{-1}; ^1H NMR (400 MHz, $CDCl_3$) δ 7.69-7.66 (1H, m, Ar**H**), 7.49-7.46 (1H, m, Ar**H**), 7.33-7.29 (2H, m, Ar**H**), 6.92 (1H, dt, *J* = 15.8, 6.9 Hz, ArCH=C**H**), 6.41 (1H, dt *J* = 15.8, 1.3 Hz, ArC**H**=CH), 2.76-2.62 (4H, m, C**H**$_2$), 2.22-2.15 (2H, m, C**H**$_2$), 2.07-2.01 (2H, m, C**H**$_2$), 2.00-1.94 (2H, m, C**H**$_2$), 1.32 (3H, s, C**H**$_3$); ^{13}C NMR (100.6 MHz, $CDCl_3$) δ 209.9 (2 x C), 162.1 (C), 150.3 (C), 142.3 (CH), 141.9 (C), 125.0 (CH), 124.3 (CH), 119.8 (CH), 117.5 (CH), 110.3 (CH), 64.8 (C), 38.0 (2 x CH_2), 34.4 (CH_2), 28.4 (CH_2), 21.5 (CH_3), 17.5 (CH_2); HRMS (EI) Exact mass calculated for $C_{18}H_{19}NO_3$ $[M]^+$: 297.1359, found: 297.1359.

Enantioselective Michael Cyclizations

Representative Procedure

(1*R*,2*S*,5*R*)-5-Methyl-2-(2-oxo-2-phenylethyl)bicyclo[3.2.1]octane-6,8-dione (2a) and (1*S*,2*S*,5*S*)-5-methyl-2-[2-oxo-2-(4-methylphenyl)ethyl]bicyclo[3.2.1]octane-6,8-dione (3a).

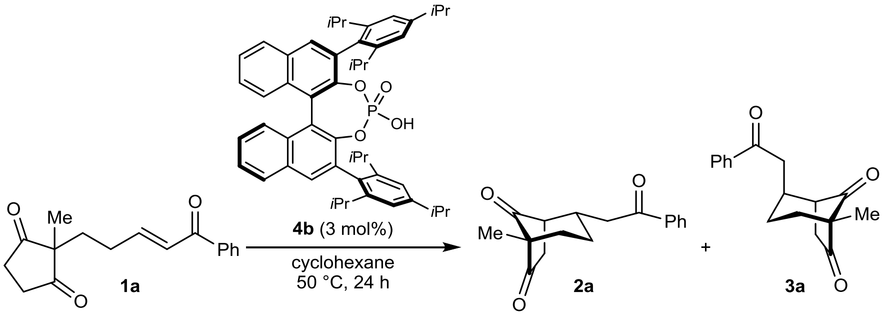

A suspension of enone **1a** (54 mg, 0.20 mmol) and (*R*)-TRIP (**4b**, 4.5 mg, 0.006 mmol) in cyclohexane (2 mL) was stirred at 50 °C for 24 h. After cooling to room temperature, the reaction mixture was diluted with EtOAc (20 mL) and washed with sat. $NaHCO_3$ (aq.) (20 mL). The aqueous layers was extracted with EtOAc (20 mL) and the combined organic layers were dried ($MgSO_4$), filtered, and concentrated *in vacuo*. Purification of the residue by column chromatography (20% EtOAc/petroleum ether) gave the *bicyclo[3.2.1]octanes* **2a** as a pale yellow solid (50 mg, 93%) and **3a** as white solid (4 mg, 7%).

Data for **2a**: R_f = 0.40 (20% EtOAc/petroleum ether); m.p. 104-107 °C (CH_2Cl_2); $[\alpha]_D^{20}$ −13.3 (*c* 0.96, $CHCl_3$); IR 2934, 1768 (C=O), 1728 (C=O), 1681 (C=O), 1452, 1410, 1286, 1240, 977, 660 cm^{-1}; ^1H NMR (400 MHz, $CDCl_3$) δ 7.95-7.91 (2H, m, Ar**H**), 7.67-7.58 (1H, app t, *J* = 7.4 Hz, Ar**H**), 7.55-7.47 (2H, app t, *J* = 7.6 Hz, Ar**H**), 3.13-2.95 (3H, m, C**H**CH_2C(O)Ar), 2.92 (1H, dd, *J* = 7.5, 1.2 Hz, C**H**C=O), 2.75 (1H, d, *J* = 19.2 Hz, C$\mathbf{H_A}H_B$C(O)CCH_3), 2.57 (1H, dd, *J* = 19.2, 7.6 Hz, $CH_A\mathbf{H_B}$C(O)CCH_3), 1.97 (1H, app dd, *J* = 11.8, 6.0 Hz, C$\mathbf{H_A}H_B$$CCH_3$), 1.94-1.83 (2H, m, $CH_A\mathbf{H_B}CCH_3$ and C$\mathbf{H_A}H_B$$CH_2CCH_3$), 1.56-1.36 (1H, m, $CH_A\mathbf{H_B}CH_2CCH_3$), 1.08 (3H, s, C$\mathbf{H_3}$); ^{13}C NMR (100.6 MHz, $CDCl_3$) δ 215.7 (C), 211.6 (C), 197.3 (C), 136.5 (C), 133.5 (CH), 128.8 (2 x CH), 128.0 (2 x CH), 58.7 (C), 49.8 (CH), 42.4 (CH_2), 42.1 (CH_2), 41.1 (CH), 39.3 (CH_2), 24.9 (CH_2), 11.8 (CH_3); HRMS (ESI) Exact mass calculated for $C_{17}H_{18}NaO_3$ $[M+Na]^+$: 293.1148, found: 293.1148; Enantiomeric excess was determined by HPLC with a Chiralpak IA-3 column (60:40 *iso*-hexane:*i*-PrOH, 1.5 mL/min, 254 nm, 25 °C); t_r (major) = 5.5 min, t_r (minor) = 8.6 min; 91% ee.

Data for **3a**: R_f = 0.45 (20% EtOAc/petroleum ether); m.p. 127-129 °C (CH_2Cl_2); $[\alpha]_D^{20}$ +12.7 (*c* 0.37, $CHCl_3$); IR 2930, 1764 (C=O), 1726 (C=O), 1687 (C=O), 1450, 1408, 1281, 1240, 982, 643 cm^{-1}; ^1H NMR (400 MHz, $CDCl_3$) δ 8.00-7.93 (2H, m, Ar**H**), 7.57 (1H, app tt, *J* = 7.6, 1.4 Hz,

Ar**H**), 7.48 (2H, app t, *J* = 7.6 Hz, Ar**H**), 3.26 (1H, dd, *J* = 17.3, 5.9 Hz, C$\mathbf{H_A}$H$_B$C(O)Ar), 3.21-3.13 (1H, m, C**H**CH$_2$C(O)Ar), 2.96 (1H, dd, *J* = 17.3, 7.5 Hz, CH$_A$$\mathbf{H_B}$C(O)Ar), 2.92-2.86 (1H, m, C**H**C=O), 2.79-2.73 (2H, m, C$\mathbf{H_2}$C(O)CCH$_3$), 2.12-1.97 (3H, m, C$\mathbf{H_2}$CCH$_3$ and C$\mathbf{H_A}$H$_B$CH$_2$CCH$_3$), 1.59-1.53 (1H, m, CH$_A$$\mathbf{H_B}CH_2CCH_3$); ^{13}C NMR (100.6 MHz, CDCl$_3$) δ 216.7 (C), 211.7 (C), 197.9 (C), 136.7 (C), 133.4 (CH), 128.7 (2 x CH), 128.1 (2 x CH), 59.2 (C), 50.1 (CH), 44.1 (CH$_2$), 41.63 (CH), 41.57 (CH$_2$), 40.1 (CH$_2$), 23.0 (CH$_2$), 12.0 (CH$_3$); HRMS (ESI) Exact mass calculated for $C_{17}H_{18}NaO_3$ [M+Na]$^+$: 293.1148, found: 293.1139; Enantiomeric excess was determined by HPLC with a Chiralpak IA-3 column (70:30 *iso*-hexane:*i*-PrOH, 2.0 mL/min, 230 nm, 25 °C); t_r (major) = 3.2 min, t_r (minor) = 3.8 min; 87% ee.

Slow diffusion of cyclohexane into a solution of **3a** in CH_2Cl_2 gave crystals that were suitable for X-ray crystallography:

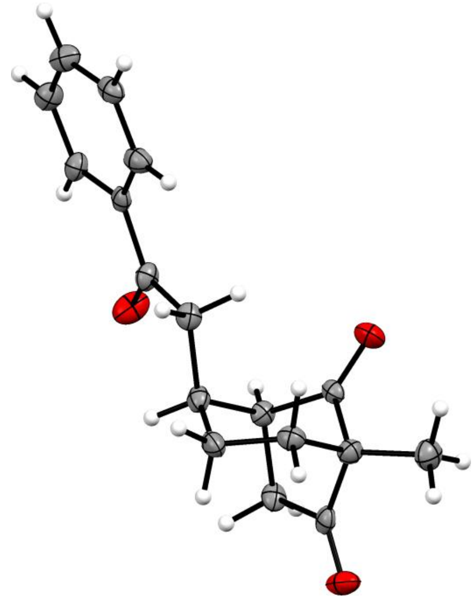

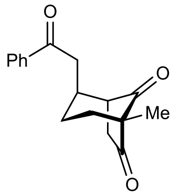

3a
CCDC 1045001

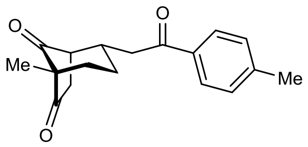

(1*R*,2*S*,5*R*)-5-Methyl-2-[2-oxo-2-(4-methylphenyl)ethyl]bicyclo[3.2.1] octane-6,8-dione (2b). The title compound was prepared according to the Representative Procedure from enone **1b** (57 mg, 0.20 mmol). Purification by column chromatography (20% EtOAc/petroleum ether) gave a white solid (52 mg, 91%) as a >95:5 ratio of diastereomers. R_f = 0.38 (20% EtOAc/petroleum ether); m.p. 102-104 °C (CH_2Cl_2); $[\alpha]_D^{20}$ −9.5 (c 0.99, CHCl$_3$); IR 2934, 1768 (C=O), 1728 (C=O), 1682 (C=O), 1572, 1452, 1410, 1240, 977, 660 cm^{-1}; ^{1}H NMR (400 MHz, CDCl$_3$) δ 7.83 (2H, d, *J* = 8.1 Hz, Ar**H**), 7.28 (2H, d, *J* = 8.1 Hz, Ar**H**), 3.07-2.92 (3H, m, C**H**C$\mathbf{H_2}$C(O)Ar), 2.90 (1H, dd, *J* = 7.6, 1.4 Hz, C**H**C=O), 2.74 (1H, d, *J* = 19.2 Hz, C$\mathbf{H_A}$H$_B$C(O)CCH$_3$), 2.55 (1H, dd, *J* = 19.2, 7.6 Hz, CH$_A$$\mathbf{H_B}$C(O)CCH$_3$), 2.42 (3H, s, ArC$\mathbf{H_3}$), 1.96 (1H, app dd, *J* = 11.9, 5.7 Hz, C$\mathbf{H_A}$H$_B$CCH$_3$), 1.91-1.81 (2H, m, CH$_A$$\mathbf{H_B}CCH_3$ and C$\mathbf{H_A}$H$_B$CH$_2$CCH$_3$), 1.55-1.35 (1H, m, CH$_A$$\mathbf{H_B}CH_2CCH_3$), 1.07 (3H, s, C$\mathbf{H_3}$CC=O); ^{13}C NMR (100.6 MHz, CDCl$_3$) δ 215.8 (C), 211.7 (C), 197.0 (C), 144.4 (C), 134.1 (C),

129.4 (2 x CH), 128.2 (2 x CH), 58.7 (C), 49.8 (CH), 42.3 (CH_2), 42.1 (CH_2), 41.3 (CH), 39.3 (CH_2), 24.9 (CH_2), 21.6 (CH_3), 11.8 (CH_3); HRMS (ESI) Exact mass calculated for $C_{18}H_{20}NaO_3$ $[M+Na]^+$: 307.1305, found: 307.1291; Enantiomeric excess was determined by HPLC with a Chiralpak IA-3 column (70:30 *iso*-hexane:*i*-PrOH, 1.5 mL/min, 254 nm, 25 °C); t_r (major) = 10.9 min, t_r (minor) = 27.4 min; 92% ee.

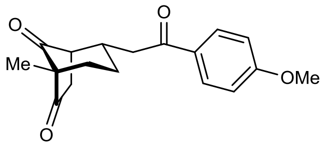

(1*R*,2*S*,5*R*)-2-[2-(4-Methoxyphenyl)-2-oxoethyl]-5-methylbicyclo[3.2.1]octane-6,8-dione (2c). The title compound was prepared according to the Representative Procedure from enone **1c** (60 mg, 0.20 mmol). Purification by column chromatography (25% EtOAc/petroleum ether) gave a white solid (55 mg, 92%) as a >95:5 ratio of diastereomers. R_f = 0.26 (20% EtOAc/petroleum ether); m.p. 107-110 °C (CH_2Cl_2); $[\alpha]_D^{20}$ −15.0 (*c* 0.95, $CHCl_3$); IR 2934, 1768 (C=O), 1727 (C=O), 1674 (C=O), 1569, 1468, 1290, 1156, 839, 644 cm^{-1}; ^{1}H NMR (400 MHz, $CDCl_3$) δ 7.91 (2H, d, *J* = 8.9 Hz, Ar**H**), 6.94 (2H, d, *J* = 8.9 Hz, Ar**H**), 3.87 (3H, s, OC**H**$_3$), 3.06-2.86 (4H, m, C**H**C**H**C**H**$_2$C(O)Ar), 2.74 (1H, d, *J* = 19.2 Hz, C**H**$_\mathbf{A}$H$_B$C(O)CCH$_3$), 2.55 (1H, dd, *J* = 19.2, 7.6 Hz, CH$_A$**H**$_\mathbf{B}$C(O)CCH$_3$), 2.01-1.79 (3H, m, C**H**$_2$CCH$_3$ and C**H**$_\mathbf{A}$H$_B$CH$_2$CCH$_3$), 1.55-1.35 (1H, m, CH$_A$**H**$_\mathbf{B}$CH$_2$CCH$_3$), 1.06 (3H, s, CC**H**$_3$); ^{13}C NMR (100.6 MHz, $CDCl_3$) δ 215.8 (C), 211.7 (C), 195.8 (C), 163.8 (C), 130.3 (2 x CH), 129.6 (C), 113.9 (2 x CH), 58.7 (C), 55.5 (CH_3), 49.8 (CH), 42.2 (CH_2), 42.1 (CH_2), 41.4 (CH), 39.3 (CH_2), 25.0 (CH_2), 11.8 (CH_3); HRMS (ESI) Exact mass calculated for $C_{18}H_{20}NaO_4$ $[M+Na]^+$: 323.1254, found: 323.1248; Enantiomeric excess was determined by HPLC with a Chiralpak IA-3 column (50:50 *iso*-hexane:*i*-PrOH, 2.0 mL/min, 254 nm, 25 °C); t_r (major) = 7.7 min, t_r (minor) = 17.1 min; 91% ee.

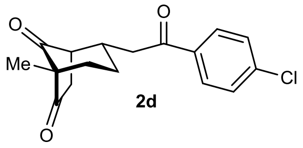

2d

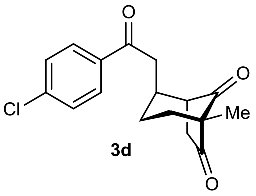

3d

(1*R*,2*S*,5*R*)-2-[2-(4-Chlorophenyl)-2-oxoethyl]-5-methylbicyclo[3.2.1]octane-6,8-dione (2d) and (1*S*,2*S*,5*S*)-2-[2-(4-chlorophenyl)-2-oxoethyl]-5-methylbicyclo[3.2.1]octane-6,8-dione (3d). The title compounds **2d** and **3d** were prepared according to the Representative Procedure from enone **1d** (61 mg, 0.20 mmol). Purification by column chromatography (20% EtOAc/petroleum ether) gave **2d** as an off-white solid (49 mg, 80%) and **3d** as a colorless film (7 mg, 11%).

Data for **2d**: R_f = 0.18 (20% EtOAc/petroleum ether); m.p. 117-121°C (CH_2Cl_2); $[\alpha]_D^{20}$ −10.0 (*c* 1.00, $CHCl_3$); IR 2934, 1768 (C=O), 1728 (C=O), 1687 (C=O), 1590, 1452, 1285, 1094, 980, 646 cm^{-1}; ^{1}H NMR (400 MHz, $CDCl_3$) δ 7.88 (2H, d, *J* = 8.6 Hz, Ar**H**), 7.46 (2H, d, *J* = 8.6 Hz, Ar**H**), 3.07-2.92 (3H, m, C**H**C**H**$_2$C(O)Ar), 2.91 (1H, app d, *J* = 7.6 Hz, C**H**C=O), 2.73 (1H, d, *J* = 19.2

Hz, C**H**$_\mathbf{A}$H$_B$C(O)CCH$_3$), 2.57 (1H, dd, J = 19.2, 7.6 Hz, CH$_A$**H**$_\mathbf{B}$C(O)CCH$_3$), 1.97 (1H, app dd, J = 12.0, 5.8 Hz, C**H**$_\mathbf{A}$H$_B$CCH$_3$), 1.93-1.83 (2H, m, CH$_A$**H**$_\mathbf{B}$CCH$_3$, C**H**$_\mathbf{A}$H$_B$CH$_2$CCH$_3$), 1.52-1.37 (1H, m, CH$_A$**H**$_\mathbf{B}$CH$_2$CCH$_3$), 1.08 (3H, s, C**H**$_3$); ^{13}C NMR (100.6 MHz, CDCl$_3$) δ 215.6 (C), 211.5 (C), 196.0 (C), 140.0 (C), 134.8 (C), 129.4 (2 x CH), 129.1 (2 x CH), 58.7 (C), 49.7 (CH), 42.3 (CH$_2$), 42.1 (CH$_2$), 41.0 (CH), 39.3 (CH$_2$), 24.9 (CH$_2$), 11.8 (CH$_3$); HRMS (ESI) Exact mass calculated for $C_{17}H_{17}ClNaO_3$ [M+Na]$^+$: 327.0758, found: 327.0750. Enantiomeric excess was determined by HPLC with a Chiralpak IA-3 column (60:40 *iso*-hexane:*i*-PrOH, 1.5 mL/min, 230 nm, 25 °C); t_r (major) = 7.5 min, t_r (minor) = 10.5 min; 94% ee.

Slow diffusion of cyclohexane into a solution of **2d** in CH_2Cl_2 gave crystals that were suitable for X-ray crystallography:

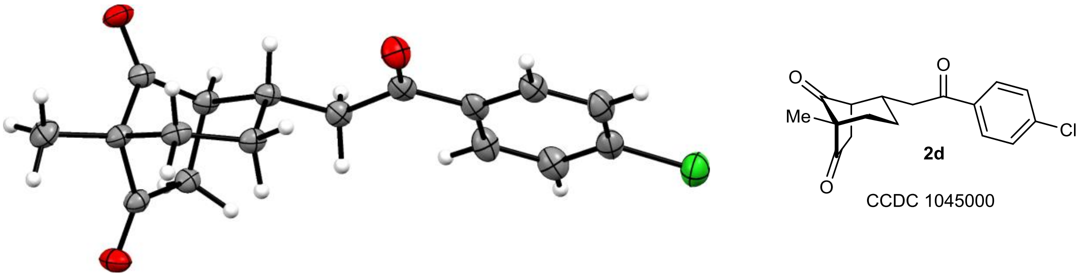

Data for **3d**: R$_f$ = 0.20 (20% EtOAc/petroleum ether); $[\alpha]_D^{20}$ +38.4 (*c* 0.40, CHCl$_3$); IR 2929, 1725 (C=O), 1686 (C=O), 1590, 1489, 1459, 1350, 1142, 1092, 980 cm^{-1}; ^{1}H NMR (400 MHz, CDCl$_3$) δ 7.91 (2H, d, J = 8.6 Hz, Ar**H**), 7.46 (2H, d, J = 8.6 Hz, Ar**H**), 3.24 (1H, dd, J = 17.2, 6.1 Hz, C**H**$_\mathbf{A}$H$_B$C(O)Ar), 3.20-3.13 (1H, m, C**H**CH$_2$C(O)Ar), 2.96-2.87 (2H, m, CH$_A$**H**$_\mathbf{B}$C(O)Ar and C**H**C=O), 2.81-2.70 (2H, m, C**H**$_2$C(O)CCH$_3$), 2.11-1.97 (3H, m, C**H**$_2$CCH$_3$ and C**H**$_\mathbf{A}$H$_B$CH$_2$CCH$_3$), 1.59-1.53 (1H, m, CH$_A$**H**$_\mathbf{B}$CH$_2$CCH$_3$, overlapped with H$_2$O), 1.07 (3H, s, C**H**$_3$); ^{13}C NMR (100.6 MHz, CDCl$_3$) δ 216.8 (C), 211.6 (C), 196.7 (C), 140.0 (C), 135.0 (C), 129.5 (2 x CH), 129.0 (2 x CH), 59.2 (C), 50.0 (CH), 44.1 (CH$_2$), 41.6 (CH$_2$ and CH), 40.1 (CH$_2$), 23.0 (CH$_2$), 12.0 (CH$_3$); HRMS (ESI) Exact mass calculated for $C_{17}H_{17}ClNaO_3$ [M+Na]$^+$: 327.0758, found: 327.0757. Enantiomeric excess was determined by HPLC with a Chiralpak IA-3 column (60:40 *iso*-hexane:*i*-PrOH, 1.5 mL/min, 230 nm, 25 °C); t_r (major) = 4.6 min, t_r (minor) = 5.5 min; 85% ee.

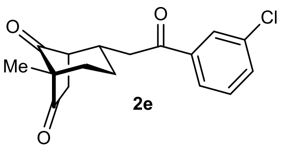

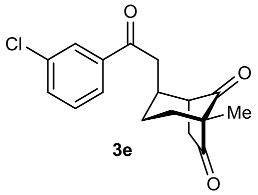

(1*R*,2*S*,5*R*)-2-[2-(3-Chlorophenyl)-2-oxoethyl]-5-methylbicyclo[3.2.1]octane-6,8-dione (2e) and (1*S*,2*S*,5*S*)-2-[2-(3-chlorophenyl)-2-oxoethyl]-5-methylbicyclo[3.2.1]octane-6,8-dione (3e). The title compounds **2e** and **3e** were prepared according to the Representative Procedure from enone **1e** (61 mg, 0.20 mmol). Purification by column chromatography (25% EtOAc/petroleum ether) gave **2e** as an off-white solid (48 mg, 79%) and **3e** as a colorless film (8 mg, 13%).

Data for **2e**: R_f = 0.14 (20% EtOAc/petroleum ether); m.p. 89-91 °C (CH_2Cl_2); $[\alpha]_D^{20}$ −17.6 (*c* 1.22, $CHCl_3$); IR 2934, 1769 (C=O), 1728 (C=O), 1691 (C=O), 1572, 1453, 1420, 1240, 1044, 984 cm^{-1}; ^{1}H NMR (400 MHz, $CDCl_3$) δ 7.90 (1H, app t, *J* = 2.1 Hz, Ar**H**), 7.83-7.78 (1H, m, Ar**H**), 7.56 (1H, ddd, *J* = 8.0, 2.1, 1.0 Hz, Ar**H**), 7.43 (1H, app t, *J* = 8.0 Hz, Ar**H**), 3.08-2.92 (3H, m, C**H**C**H**$_2$C(O)Ar), 2.90 (1H, dd, *J* = 7.5, 1.5 Hz, C**H**C=O), 2.73 (1H, d, *J* = 19.2 Hz, C**H$_A$**H$_B$C(O)CCH$_3$), 2.57 (1H, dd, *J* = 19.2, 7.6 Hz, CH$_A$**H$_B$**C(O)CCH$_3$), 1.97 (1H, app dd, *J* = 12.0, 5.7 Hz, C**H$_A$**H$_B$CCH$_3$), 1.92-1.83 (2H, m, C**H$_A$**H$_B$CH$_A$**H$_B$**CCH$_3$), 1.52-1.38 (1H, m, CH$_A$**H$_B$**CH$_2$CCH$_3$), 1.07 (3H, s, C**H$_3$**); ^{13}C NMR (100.6 MHz, $CDCl_3$) δ 215.6 (C), 211.5 (C), 196.0 (C), 138.0 (C), 135.2 (C), 133.4 (CH), 130.1 (CH), 128.1 (CH), 126.1 (CH), 58.7 (C), 49.7 (CH), 42.5 (CH_2), 42.0 (CH_2), 40.9 (CH), 39.3 (CH_2), 24.9 (CH_2), 11.8 (CH_3); HRMS (ESI) Exact mass calculated for $C_{17}H_{17}ClNaO_3$ $[M+Na]^+$: 327.0758, found: 327.0743; Enantiomeric excess was determined by HPLC with a Chiralpak AD-H column (95:5 *iso*-hexane:*i*-PrOH, 1.5 mL/min, 210 nm, 25 °C); t_r (minor) = 26.7 min, t_r (major) = 28.7 min; 86% ee.

Data **3e**: R_f = 0.21 (20% EtOAc/petroleum ether); $[\alpha]_D^{20}$ +6.7 (*c* 0.50, $CHCl_3$); IR 2927, 1764 (C=O), 1725 (C=O), 1690 (C=O), 1572, 1455, 1373, 1262, 1085, 870 cm^{-1}; ^{1}H NMR (400 MHz, $CDCl_3$) δ 7.94 (1H, app t, *J* = 2.1 Hz, Ar**H**), 7.87-7.83 (1H, m, Ar**H**), 7.57 (1H, ddd, *J* = 8.0, 2.1, 1.1 Hz, Ar**H**), 7.44 (1H, app t, *J* = 8.0 Hz, Ar**H**), 3.24 (1H, dd, *J* = 17.4, 6.1 Hz, C**H$_A$**H$_B$C(O)Ar), 3.21-3.13 (1H, m, C**H**CH$_2$C(O)Ar), 2.96-2.87 (2H, m, CH$_A$**H$_B$**C(O)Ar and C**H**C=O), 2.80-2.70 (2H, m, C**H**$_2$C(O)CCH$_3$), 2.12-2.98 (3H, m, C**H**$_2$CCH$_3$ and C**H$_A$**H$_B$CH$_2$CCH$_3$), 1.59-1.53 (1H, m, CH$_A$**H$_B$**CH$_2$CCH$_3$ overlapped with H_2O), 1.07 (3H, s, C**H$_3$**); ^{13}C NMR (100.6 MHz, $CDCl_3$) δ 216.7 (C), 211.5 (C), 196.6 (C), 138.2 (C), 135.1 (C), 133.4 (CH), 130.1 (CH), 128.2 (CH), 126.2 (CH), 59.2 (C), 50.0 (CH), 44.1 (CH_2), 41.6 (CH), 41.5 (CH_2), 40.3 (CH_2), 23.0 (CH_2), 12.0 (CH_3); HRMS (ESI) Exact mass calculated for $C_{17}H_{17}ClNaO_3$ $[M+Na]^+$: 327.0758, found: 327.0752; Enantiomeric excess was determined by HPLC with a Chiralpak AD-H column (95:5 *iso*-hexane:*i*-PrOH, 1.5 mL/min, 210 nm, 25 °C); t_r (major) = 12.0 min, t_r (minor) = 17.4 min; 66% ee.

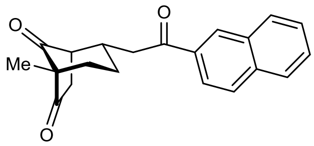

(1*R*,2*S*,5*R*)-5-Methyl-2-[2-(naphthalen-2-yl)-2-oxoethyl]bicyclo[3.2.1]octane-6,8-dione (2f). The title compound was prepared according to the Representative Procedure from enone **1f** (64 mg, 0.20 mmol). Purification by column chromatography (20% EtOAc/petroleum ether) gave an off white solid (62 mg, 97%) as a >95:5 ratio of diastereomers. R_f = 0.39 (30% EtOAc/petroleum ether); m.p. 131-134 °C (CH_2Cl_2); $[\alpha]_D^{20}$ −16.1 (*c* 1.19, $CHCl_3$); IR 2933, 1768 (C=O), 1728 (C=O), 1681 (C=O), 1598, 1469, 1453, 1276, 1096, 825 cm^{-1}; ^1H NMR (400 MHz, $CDCl_3$) δ 8.45 (1H, s, Ar**H**), 8.04-7.96 (2H, m, Ar**H**), 7.95-7.88 (2H, m, Ar**H**), 7.67-7.56 (2H, m, Ar**H**), 3.20 (1H, dd, *J* = 16.4, 7.3 Hz, C$\mathbf{H_A}$$H_B$C(O)Ar), 3.15 (1H, dd, *J* = 16.4, 6.2 Hz, C$H_A$$\mathbf{H_B}$C(O)Ar), 3.09-3.00 (1H, m, C**H**CH_2C(O)Ar), 2.97 (1H, dd, *J* = 7.6, 2.1 Hz, C**H**C=O), 2.80 (1H, d, *J* = 19.2 Hz, C$\mathbf{H_A}$$H_B$C(O)C$CH_3$), 2.60 (1H, dd, *J* = 19.2, 7.7 Hz, C$H_A$$\mathbf{H_B}$C(O)C$CH_3$), 2.03-1.84 (3H, m, C$\mathbf{H_A}$$H_BC\mathbf{H_2}CCH_3$), 1.58-1.46 (1H, m, C$H_A$$\mathbf{H_B}$$CH_2CCH_3$), 1.09 (3H, s, C$\mathbf{H_3}$); ^{13}C NMR (100.6 MHz, $CDCl_3$) δ 215.7 (C), 211.7 (C), 197.3 (C), 135.7 (C), 133.9 (C), 132.5 (C), 129.8 (CH), 129.6 (CH), 128.8 (CH), 128.7 (CH), 127.8 (CH), 127.0 (CH), 123.6 (CH), 58.7 (C), 49.9 (CH), 42.5 (CH_2), 42.2 (CH_2), 41.3 (CH), 39.4 (CH_2), 25.0 (CH_2), 11.9 (CH_3); HRMS (ESI) Exact mass calculated for $C_{21}H_{20}NaO_3$ $[M+Na]^+$: 343.1310, found: 343.1297; Enantiomeric excess was determined by HPLC with a Chiralpak AD-H column (95:5 *iso*-hexane:*i*-PrOH, 1.5 mL/min, 254 nm, 25 °C); t_r (minor) = 51.1 min, t_r (major) = 55.7 min; 91% ee.

Gram-scale experiment: A suspension of enone **1f** (1.00 g, 3.12 mmol) and (*R*)-TRIP (**4b**, 35 mg, 0.05 mmol) in 4:1 cyclohexane:toluene (32 mL) was stirred at 50 °C for 90 h. After cooling to room temperature, the reaction mixture was diluted with EtOAc (100 mL) and washed with saturated aqueous $NaHCO_3$ solution (100 mL). The combined aqueous layers were extracted with EtOAc (100 mL) and the combined organic layers were dried ($MgSO_4$), filtered, and concentrated *in vacuo*. Purification of the residue by column chromatography (20% EtOAc/petroleum ether) gave the *bicyclo[3.2.1]octane* **2f** as an off-white solid (835 mg, 84%) as a >95:5 ratio of diastereomers. Enantiomeric excess was determined by HPLC with a Chiralpak AD-H column (95:5 *iso*-hexane:*i*-PrOH, 1.5 mL/min, 254 nm, 25 °C); t_r (minor) = 51.6 min, t_r (major) = 57.1 min; 90% ee.

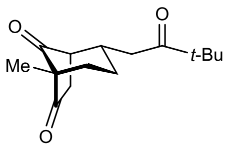

(1*R*,2*S*,5*R*)-2-(3,3-Dimethyl-2-oxobutyl)-5-methylbicyclo[3.2.1]octane-6,8-dione (2g). The title compound was prepared according to the Representative Procedure from enone **1g** (50 mg, 0.20 mmol). Purification by column chromatography (25% EtOAc/petroleum ether) gave a white solid (48 mg, 96%) as a >95:5 ratio of diastereomers. R_f = 0.38 (20% EtOAc/petroleum ether); m.p. 111-113 °C (CH_2Cl_2); $[\alpha]_D^{20}$ −34.1 (*c* 0.64, $CHCl_3$); IR 2972, 1768 (C=O), 1726 (C=O), 1704 (C=O), 1477, 1369, 1227, 1221, 1198, 986

cm^{-1}; ^{1}H NMR (400 MHz, $CDCl_3$) δ 2.89-2.75 (2H, m, C**H**C=O and C**H**$CH_2C(O)C(CH_3)_3$), 2.65 (1H, d, *J* = 19.0 Hz, C**H$_A$**H_BC(O)CCH_3), 2.63-2.47 (3H, m, C**H$_2$**CH_A**H$_B$**C(O)$CCH_3)_3$), 1.97-1.74 (3H, m, C**H$_A$**H_BCCH_3 and C**H$_A$**H_BCH_A**H$_B$**CCH_3), 1.35-1.27 (1H, m, CH_A**H$_B$**CH_2CCH_3), 1.14 (9H, s, C(C**H$_3$**)$_3$), 1.07 (3H, m, C**H$_3$**); ^{13}C NMR (100.6 MHz, $CDCl_3$) δ 215.8 (C), 212.9 (C), 211.8 (C), 58.6 (C), 49.8 (CH), 44.2 (C), 42.1 (CH_2), 40.6 (CH), 40.4 (CH_2), 39.2 (CH_2), 26.1 (3 x CH_3), 24.7 (CH_2), 11.8 (CH_3); HRMS (ESI) Exact mass calculated for $C_{15}H_{22}NaO_3$ $[M+Na]^+$: 273.1461 found: 273.1463; Enantiomeric excess was determined by HPLC with a CHIRALPAK AD-H column (85:15 *iso*-hexane:*i*-PrOH, 1.0 mL/min, 210 nm, 25 °C); t_r (minor) = 7.2 min, t_r (major) = 9.6 min; 95% ee.

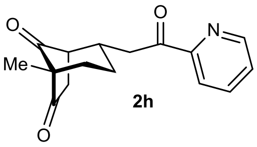

2h

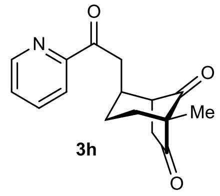

3h

(1*R*,2*S*,5*R*)-5-Methyl-2-[2-oxo-2-(pyridin-2-yl)ethyl]bicyclo[3.2.1]octane-6,8-dione (2h) and (1*S*,2*S*,5*S*)-5-methyl-2-[2-oxo-2-(pyridin-2-yl)ethyl]bicyclo[3.2.1]octane-6,8-dione (3h). The title compounds **2h** and **3h** were prepared according to a modification of the Representative Procedure from enone **1h** (54 mg, 0.20 mmol) using toluene (2 mL) as solvent. Purification by column chromatography (40% EtOAc/petroleum ether) gave **2h** as a yellow oil (41 mg, 76%) and **3h** as a pale brown film (11 mg, 20%).

Data for **2h**: R_f = 0.29 (40% EtOAc/petroleum ether); $[\alpha]_D^{20}$ −11.1 (*c* 2.03, $CHCl_3$); IR 2979, 2934, 2874, 1767 (C=O), 1727 (C=O), 1700 (C=O), 1584, 1452, 1044, 997 cm^{-1}; ^{1}H NMR (400 MHz, $CDCl_3$) δ 8.67 (1H, ddd, *J* = 4.7, 1.6, 0.9 Hz, Ar**H**), 8.04 (1H, dt, *J* = 7.8, 1.0 Hz, Ar**H**), 7.85 (1H, td, *J* = 7.7, 1.7 Hz, Ar**H**), 7.49 (1H, ddd, *J* = 7.6, 4.8, 1.2 Hz, Ar**H**), 3.35 (1H, dd, *J* = 16.7, 7.1 Hz, C**H$_A$**H_BC(O)Ar), 3.25 (1H, dd, *J* = 16.7, 6.7 Hz, CH_A**H$_B$**C(O)Ar), 3.04-2.92 (1H, m, C**H**CH_2C(O)Ar), 2.88 (1H, dd, *J* = 7.6, 2.1 Hz, C**H**C=O), 2.82 (1H, d, *J* = 19.3 Hz, C**H$_A$**H_BC(O)CCH_3), 2.54 (1H, dd, *J* = 19.3, 7.7 Hz, CH_A**H$_B$**C(O)CCH_3), 1.96 (1H, app dd, *J* = 11.7, 5.7 Hz, C**H$_A$**H_BCCH_3), 1.91-1.81 (2H, m, C**H$_A$**H_BCH_A**H$_B$**CCH_3), 1.56-1.42 (1H, m, CH_A**H$_B$**CH_2CCH_3), 1.06 (3H, s, C**H$_3$**); ^{13}C NMR (100.6 MHz, $CDCl_3$) δ 216.1 (C), 211.9 (C), 199.3 (C), 152.9 (C), 149.0 (CH), 137.1 (CH), 127.5 (CH), 121.9 (CH), 58.8 (C), 49.9 (CH), 42.2 (CH_2), 41.5 (CH_2), 41.3 (CH), 39.4 (CH_2), 24.9 (CH_2), 11.8 (CH_3); HRMS (ESI) Exact mass calculated for $C_{16}H_{18}NO_3$ $[M+H]^+$: 272.1281, found: 272.1275; Enantiomeric excess was determined by HPLC with a Chiralpak IA-3 column (70:30 *iso*-hexane:*i*-PrOH, 1.5 mL/min, 230 nm, 25 °C); t_r (major) = 7.4 min, t_r (minor) = 11.9 min; 87% ee.

Data for **3h**: R_f = 0.34 (40% EtOAc/petroleum ether); $[\alpha]_D^{20}$ −2.8 (*c* 0.20, $CHCl_3$); IR 2978, 2934, 2859, 1764 (C=O), 1726 (C=O), 1699 (C=O), 1585, 1453, 1045, 996 cm^{-1}; ^{1}H NMR (400 MHz,

$CDCl_3$) δ 8.69 (1H, ddd, *J* = 4.8, 1.7, 1.1 Hz, Ar**H**), 8.04 (1H, dt, *J* = 7.8, 1.1 Hz, Ar**H**), 7.85 (1H, td, *J* = 7.8, 1.1 Hz, Ar**H**), 7.49 (1H, ddd, *J* = 7.8, 4.8, 1.1 Hz, Ar**H**), 3.49 (1H, dd, *J* = 18.0, 6.8 Hz, C**H**$_\mathbf{A}$H$_B$C(O)Ar), 3.32 (1H, dd, *J* = 18.0, 7.5 Hz, CH$_A$**H**$_\mathbf{B}$C(O)Ar), 3.17-3.08 (1H, m, C**H**CH$_2$C(O)Ar), 3.01-2.94 (1H, m, C**H**C=O), 2.83-2.68 (2H, m, C**H**$_\mathbf{2}$C(O)CCH$_3$), 2.19-1.95 (3H, m, C**H**$_\mathbf{A}$H$_B$C**H**$_\mathbf{2}$CCH$_3$), 1.59-1.53 (1H, m, CH$_A$**H**$_\mathbf{B}$CH$_2$CCH$_3$ overlapped with H_2O), 1.07 (3H, s, C**H**$_\mathbf{3}$); ^{13}C NMR (100.6 MHz, $CDCl_3$) δ 216.2 (C), 212.1 (C), 199.9 (C), 153.0 (C), 149.1 (CH), 136.9 (CH), 127.4 (CH), 121.8 (CH), 59.2 (C), 50.0 (CH), 44.2 (CH_2), 41.7 (CH_2), 41.4 (CH), 39.5 (CH_2), 23.2 (CH_2), 12.1 (CH_3); HRMS (ESI) Exact mass calculated for $C_{16}H_{18}NO_3$ $[M+H]^+$: 272.1281, found: 272.1300; Enantiomeric excess was determined by HPLC with a Chiralpak AD-H column (90:10 *iso*-hexane:*i*-PrOH, 1.0 mL/min, 230 nm, 25 °C); t_r (minor) = 15.3 min, t_r (major) = 16.5 min; 29% ee. Due to the low enantiomeric excess, the absolute stereochemistry of **3h** should be considered as a tentative assignment.

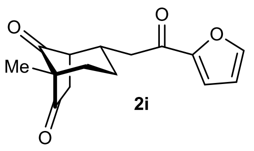

2i

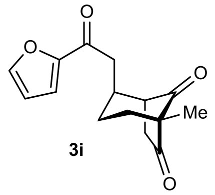

3i

(1*R*,2*S*,5*R*)-2-[2-(Furan-2-yl)-2-oxoethyl]-5-methylbicyclo[3.2.1]octane-6,8-dione (2i) and (1*S*,5*S*,6*S*)-6-[2-(furan-2-yl)-2-oxoethyl]-1-methylbicyclo[3.3.1]nonane-2,9-dione (3i). The title compounds **2i** and **3i** were prepared according to the Representative Procedure from enone **1i** (52 mg, 0.20 mmol). Purification by column chromatography (40% EtOAc/petroleum ether) gave **2i** as a white solid (42 mg, 81%) and **3i** as an off-white solid (9 mg, 17%).

Data for **2i**: R_f = 0.34 (40% EtOAc/petroleum ether); m.p. 137-140 °C (CH_2Cl_2); $[\alpha]_D^{20}$ −15.2 (*c* 0.40, $CHCl_3$); IR 2934, 1768 (C=O), 1728 (C=O), 1675 (C=O), 1560, 1469, 1396, 1290, 1043, 884 cm^{-1}; ^{1}H NMR (400 MHz, $CDCl_3$) δ 7.59 (1H, dd, *J* = 1.7, 0.7 Hz, Ar**H**), 7.22 (1H, dd, *J* = 3.6, 0.7 Hz, Ar**H**), 6.57 (1H, dd, *J* = 3.6, 1.7 Hz, Ar**H**), 2.96-2.84 (4H, m, C**H**C**H**C**H**$_\mathbf{2}$C(O)Ar), 2.76 (1H, d, *J* = 19.3 Hz, C**H**$_\mathbf{A}$H$_B$C(O)CCH$_3$), 2.56 (1H, dd, *J* = 19.3, 7.6 Hz, CH$_A$**H**$_\mathbf{B}$C(O)CCH$_3$), 1.96 (1H, app dd, *J* = 12.1, 5.6 Hz, C**H**$_\mathbf{A}$H$_B$CCH$_3$), 1.90-1.81 (2H, m, C**H**$_\mathbf{A}$H$_B$CH$_A$**H**$_\mathbf{B}$CCH$_3$), 1.53-1.40 (1H, m, CH$_A$**H**$_\mathbf{B}$CH$_2$CCH$_3$), 1.06 (3H, s, C**H**$_\mathbf{3}$); ^{13}C NMR (100.6 MHz, $CDCl_3$) δ 215.7 (C), 211.6 (C), 186.6 (C), 152.6 (C), 146.6 (CH), 117.3 (CH), 112.6 (CH), 58.7 (C), 49.8 (CH), 42.3 (CH_2), 42.1 (CH_2), 41.2 (CH), 39.2 (CH_2), 24.9 (CH_2), 11.8 (CH_3); HRMS (ESI) Exact mass calculated for $C_{15}H_{16}NaO_4$ $[M+Na]^+$: 283.0941, found: 283.0931; Enantiomeric excess was determined by HPLC with a Chiralpak IA-3 column (70:30 *iso*-hexane:*i*-PrOH, 1.5 mL/min, 230 nm, 25 °C); t_r (major) = 6.9 min, t_r (minor) = 10.7 min; 88% ee.

Data for **3i**: R_f = 0.39 (40% EtOAc/petroleum ether); m.p. 132-134 °C (CH_2Cl_2); $[\alpha]_D^{20}$ +22.4 (*c* 1.00, $CHCl_3$); IR 2935, 1764 (C=O), 1726 (C=O), 1675 (C=O), 1468, 1454, 1397, 1285, 1035, 884

cm^{-1}; ^{1}H NMR (400 MHz, $CDCl_3$) δ 7.61 (1H, dd, *J* = 1.7, 0.7 Hz, Ar**H**), 7.25 (1H, dd, *J* = 3.6, 0.7 Hz, Ar**H**), 6.56 (1H, dd, *J* = 3.6, 1.7 Hz, Ar**H**), 3.13-3.03 (2H, m, C**H**$_2$C(O)Ar), 2.90-2.79 (2H, m, C**H**C**H**CH_2C(O)Ar), 2.76-2.71 (2H, m, C**H**$_2$C(O)CCH_3), 2.08-1.97 (3H, m, C**H$_A$**H_BC**H**$_2$$CCH_3$), 1.62-1.52 (1H, m, CH_A**H$_B$**CH_2CCH_3 overlapped with H_2O), 1.07 (3H, s, C**H**$_3$); ^{13}C NMR (100.6 MHz, $CDCl_3$) δ 216.4 (C), 211.6 (C), 187.0 (C), 152.6 (C), 146.7 (CH), 117.6 (CH), 112.4 (CH), 59.2 (C), 50.1 (CH), 44.1 (CH_2), 41.6 (CH_2), 41.4 (CH), 39.9 (CH_2), 22.9 (CH_2), 12.0 (CH_3); HRMS (ESI) Exact mass calculated for $C_{15}H_{16}NaO_4$ $[M+Na]^+$: 283.0941, found: 283.0943; Enantiomeric excess was determined by HPLC with a Chiralpak IA-3 column (90:10 *iso*-hexane:*i*-PrOH, 1.5 mL/min, 254 nm, 25 °C); t_r (major) = 10.5 min, t_r (minor) = 12.3 min; 90% ee.

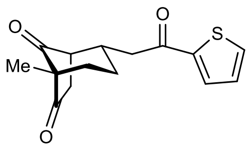

(1*R*,2*S*,5*R*)-5-Methyl-2-[2-oxo-2-(thiophen-2-yl)ethyl]bicyclo[3.2.1]octane-6,8-dione (2j). The title compound was prepared according to the Representative Procedure from enone **1j** (55 mg, 0.20 mmol). Purification by column chromatography (40% EtOAc/petroleum ether) gave an off-white solid (53 mg, 97%) as a >95:5 ratio of diastereomers. R_f = 0.33 (40% EtOAc/petroleum ether); m.p. 141-143 °C (CH_2Cl_2); $[\alpha]_D^{20}$ −7.7 (*c* 1.06, $CHCl_3$); IR 2934, 1768 (C=O), 1728 (C=O), 1660 (C=O), 1571, 1453, 1415, 1355, 1022, 860 cm^{-1}; ^{1}H NMR (400 MHz, $CDCl_3$) δ 7.71 (1H, dd, *J* = 3.8, 1.1 Hz, Ar**H**), 7.68 (1H, dd, *J* = 5.0, 1.1 Hz, Ar**H**), 7.15 (1H, dd, *J* = 5.0, 3.8 Hz, Ar**H**), 3.04-2.86 (4H, m, C**H**C**H**C**H**$_2$C(O)Ar), 2.75 (1H, d, *J* = 19.2 Hz, C**H$_A$**H_BC(O)CCH_3), 2.57 (1H, dd, *J* = 19.2, 7.6 Hz, CH_A**H$_B$**C(O)CCH_3), 1.96 (1H, app dd, *J* = 11.9, 5.7 Hz, C**H$_A$**$H_B$$CCH_3$), 1.92-1.81 (2H, m, C**H$_A$**$H_B$$CH_A$**H$_B$**$CCH_3$), 1.54-1.40 (1H, m, CH_A**H$_B$**CH_2CCH_3), 1.07 (3H, s, C**H**$_3$); ^{13}C NMR (100.6 MHz, $CDCl_3$) δ 215.6 (C), 211.6 (C), 190.2 (C), 143.9 (C), 134.3 (CH), 132.1 (CH), 128.3 (CH), 58.7 (C), 49.7 (CH), 43.2 (CH_2), 42.0 (CH_2), 41.5 (CH), 39.3 (CH_2), 24.9 (CH_2), 11.8 (CH_3); HRMS (ESI) Exact mass calculated for $C_{15}H_{16}NaO_3S$ $[M+Na]^+$: 299.0712, found: 299.0699. Enantiomeric excess was determined by HPLC with a Chiralpak IA-3 column (70:30 *iso*-hexane:*i*-PrOH, 1.5 mL/min, 230 nm, 25 °C); t_r (major) = 10.5 min, t_r (minor) = 14.3 min; 92% ee.

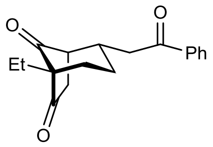

(1*R*,2*S*,5*R*)-5-Ethyl-2-(2-oxo-2-phenylethyl)bicyclo[3.2.1]octane-6,8-dione (2k). The title compound was prepared according to the Representative Procedure from enone **1k** (57 mg, 0.20 mmol). Purification by column chromatography (20% EtOAc/petroleum ether) gave a white solid (54 mg, 95%) as a >95:5 ratio of diastereomers. R_f = 0.41 (25% EtOAc/petroleum ether); m.p. 115-117 °C (CH_2Cl_2); $[\alpha]_D^{20}$ −19.9 (*c* 1.03, $CHCl_3$); IR 2941, 1766 (C=O), 1724 (C=O), 1686 (C=O), 1599, 1462, 1373, 1245, 989, 945 cm^{-1}; ^{1}H NMR (400 MHz, $CDCl_3$) δ 7.95-7.90 (2H, m, Ar**H**), 7.59 (1H, dt, *J* = 7.4, 1.3 Hz, Ar**H**),

7.51-7.45 (2H, m, Ar**H**), 3.09-2.92 (3H, m, $C\mathbf{H}C\mathbf{H}_2C(O)Ph$), 2.88 (1H, dd, J = 7.6, 1.9 Hz, $C\mathbf{H}C{=}O$), 2.74 (1H, d, J = 19.1 Hz, $C\mathbf{H}_\mathbf{A}H_BC(O)CCH_2$), 2.50 (1H, dd, J = 19.1, 7.7 Hz, $CH_A\mathbf{H}_\mathbf{B}C(O)CCH_2$), 1.97-1.81 (3H, m, $C\mathbf{H}_\mathbf{A}H_BC\mathbf{H}_2CCH_2$), 1.68 (1H, dq, J = 14.9, 7.4 Hz, $C\mathbf{H}_\mathbf{A}H_BCH_3$), 1.61 (1H, dq, J = 14.9, 7.4 Hz, $CH_A\mathbf{H}_\mathbf{B}CH_3$), 1.50-1.35 (1H, m, $CH_A\mathbf{H}_\mathbf{B}CH_2CCH_2$), 0.80 (3H, t, J = 7.4 Hz, $C\mathbf{H}_3$); ^{13}C NMR (100.6 MHz, $CDCl_3$) δ 216.0 (C), 211.9 (C), 197.3 (C), 136.5 (C), 133.5 (CH), 128.8 (2 x CH), 128.0 (2 x CH), 63.2 (C), 50.1 (CH), 42.4 (CH_2), 41.5 (CH), 41.1 (CH_2), 40.0 (CH_2), 24.8 (CH_2), 20.9 (CH_2), 9.1 (CH_3); HRMS (ESI) Exact mass calculated for $C_{18}H_{20}NaO_3$ $[M+Na]^+$: 307.1305, found: 307.1308; Enantiomeric excess was determined by HPLC with a Chiralpak IA-3 column (60:40 *iso*-hexane:*i*-PrOH, 1.5 mL/min, 254 nm, 25 °C); t_r (major) = 4.5 min, t_r (minor) = 6.1 min; 93% ee.

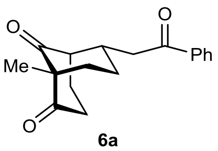

6a

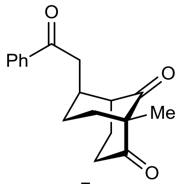

7a

(1*R*,5*R*,6*S*)-1-Methyl-6-(2-oxo-2-phenylethyl)bicyclo[3.3.1]nonane-2,9-dione (6a) and (1*S*,5*S*,6*S*)-1-methyl-6-(2-oxo-2-phenylethyl)bicyclo[3.3.1]nonane-2,9-dione (7a). The title compounds **6a** and **7a** were prepared according to the Representative Procedure from enone **5a** (57 mg, 0.20 mmol). Purification by column chromatography (20 to 30% EtOAc/petroleum ether) gave **6a** as a white solid (44 mg, 77%) and **7a** as a colorless film (8 mg, 14%).

Data for **6a**: R_f = 0.50 (40% EtOAc/petroleum ether); m.p. 142-144 °C (*iso*-hexane/CH_2Cl_2); $[\alpha]_D^{20}$ −20.2 (*c* 1.95, $CHCl_3$); IR 3040, 2938, 1732 (C=O), 1703 (C=O), 1598, 1581, 1450, 1375, 1278, 980 cm^{-1}; ^{1}H NMR (300 MHz, $CDCl_3$) δ 7.97-7.89 (2H, m, Ar**H**), 7.63-7.55 (1H, m, Ar**H**), 7.52-7.44 (2H, m, Ar**H**), 3.08 (1H, dd, J = 15.6, 6.9 Hz, $C\mathbf{H}_\mathbf{A}H_BC(O)Ar$), 3.00 (1H, dd, J = 15.6, 5.4 Hz, $CH_A\mathbf{H}_\mathbf{B}C(O)Ar$), 2.93-2.85 (1H, m, $C\mathbf{H}C{=}O$), 2.85-2.71 (1H, m, $C\mathbf{H}CH_2C(O)Ar$), 2.60 (1H, dt, J = 16.2, 6.5 Hz, $CH_2C\mathbf{H}_\mathbf{A}H_BC{=}O$), 2.40 (1H, dt, J = 16.2, 8.8 Hz, $CH_2CH_A\mathbf{H}_\mathbf{B}C{=}O$), 2.28-2.17 (1H, m, $C\mathbf{H}_\mathbf{A}H_BCCH_3$), 2.04-1.92 (2H, m, $C\mathbf{H}_2CH_2C{=}O$), 1.81-1.62 (2H, m, $C\mathbf{H}_\mathbf{A}H_BCH_A\mathbf{H}_\mathbf{B}CCH_3$), 1.57-1.47 (1H, m, $CH_A\mathbf{H}_\mathbf{B}CH_2CCH_3$), 1.16 (3H, s, $C\mathbf{H}_3$); ^{13}C NMR (100.6 MHz, $CDCl_3$) δ 211.8 (C), 211.7 (C), 197.7 (C), 136.6 (C), 133.4 (CH), 128.7 (2 x CH), 128.0 (2 x CH), 62.7 (C), 48.4 (CH), 41.5 (CH_2), 41.1 (CH_2), 40.6 (CH), 38.9 (CH_2), 25.7 (CH_2), 16.8 (CH_2), 16.6 (CH_3); HRMS (ESI) Exact mass calculated for $C_{18}H_{20}NaO_3$ $[M+Na]^+$: 307.1305, found: 307.1302; Enantiomeric excess was determined by HPLC with a Chiralpak IA-3 column (60:40 *iso*-hexane:*i*-PrOH, 1.5 mL/min, 254 nm, 25 °C); t_r (major) = 4.8 min, t_r (minor) = 5.9 min; 82% ee.

Data for **7a**: R_f = 0.56 (40% EtOAc/petroleum ether); $[\alpha]_D^{20}$ +41.8 (*c* 0.63, $CHCl_3$); IR 3011, 2936, 1732 (C=O), 1701 (C=O), 1598, 1450, 1374, 1282, 1017 cm^{-1}; ^{1}H NMR (400 MHz, $CDCl_3$) δ 7.97-7.92 (2H, m, Ar**H**), 7.62-7.56 (1H, m, Ar**H**), 7.51-7.44 (2H, m, Ar**H**), 3.11 (1H, dd, J = 16.3, 5.8

Hz, C**H**$_A$H$_B$C(O)Ar), 3.04-2.96 (1H, m, C**H**CH$_2$C(O)Ar), 2.93 (1H, dd, J = 16.3, 7.0 Hz, CH$_A$**H**$_B$C(O)Ar), 2.79-2.67 (2H, m, C**H**C=O and CH$_2$C**H**$_A$H$_B$C=O), 2.40 (1H, dt, J = 16.0, 9.1 Hz, CH$_2$CH$_A$**H**$_B$C=O), 2.33-2.18 (2H, m, C**H**$_A$H$_B$CH$_2$C=O and C**H**$_A$H$_B$CCH$_3$), 2.08-1.90 (2H, m, C**H**$_A$H$_B$CH$_2$CCH$_3$ and CH$_A$**H**$_B$CH$_2$C=O), 1.83 (1H, td, J = 13.7, 4.6 Hz, CH$_A$**H**$_B$CCH$_3$), 1.57-1.48 (1H, m, CH$_A$**H**$_B$CH$_2$CCH$_3$), 1.17 (3H, s, C**H**$_3$); ^{13}C NMR (125.8 MHz, CDCl$_3$) δ 213.2 (C), 211.9 (C), 198.1 (C), 136.8 (C), 133.4 (CH), 128.7 (2 x CH), 128.0 (2 x CH), 63.1 (C), 49.7 (CH), 41.7 (CH), 40.7 (CH$_2$), 39.2 (CH$_2$), 38.8 (CH$_2$), 23.6 (CH$_2$), 22.4 (CH$_2$), 16.8 (CH$_3$); HRMS (ESI) Exact mass calculated for $C_{18}H_{20}NaO_3$ [M+Na]$^+$: 307.1305, found: 307.1301; Enantiomeric excess was determined by HPLC with a Chiralpak IA-3 column (90:10 *iso*-hexane:*i*-PrOH, 1.5 mL/min, 254 nm, 25 °C); t$_r$ (major) = 7.1 min, t$_r$ (minor) = 8.9 min; 94% ee.

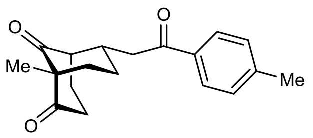

(1*R*,5*R*,6*S*)-1-Methyl-6-[2-oxo-2-(4-methylphenyl)ethyl]bicyclo[3.3.1]nonane-2,9-dione (6b). The title compound was prepared according to the Representative Procedure from enone **5b** (60 mg, 0.20 mmol). Purification by column chromatography (20 to 30% EtOAc/*iso*-hexane) gave a colorless glassy film (57 mg, >95%) as a >95:5 ratio of diastereomers. R$_f$ = 0.63 (40% EtOAc/petroleum ether); $[\alpha]_D^{20}$ −46.4 (*c* 1.00, CHCl$_3$); IR 3023, 2937, 1731 (C=O), 1703 (C=O), 1626, 1607, 1573, 1453, 1410, 1376, 1278, 1109, 979 cm^{-1}; ^{1}H NMR (400 MHz, CDCl$_3$) δ 7.84 (2H, d, J = 8.1 Hz, Ar**H**), 7.28 (2H, d, J = 8.1 Hz, Ar**H**), 3.04 (1H, dd, J = 16.2, 7.7 Hz, C**H**$_A$H$_B$C(O)Ar), 2.98 (1H, dd, J = 16.2, 6.3 Hz, CH$_A$**H**$_B$C(O)Ar), 2.92-2.85 (1H, m, C**H**C=O), 2.84-2.71 (1H, m, C**H**CH$_2$C(O)Ar), 2.60 (1H, dt, J = 16.2, 6.5 Hz, CH$_2$C**H**$_A$H$_B$C=O), 2.46-2.34 (1H, m, CH$_2$CH$_A$**H**$_B$C=O), 2.43 (3H, s, ArC**H**$_3$), 2.27-2.16 (1H, m, C**H**$_A$H$_B$CCH$_3$), 2.04-1.93 (2H, m, C**H**$_2$CH$_2$C=O), 1.79-1.63 (2H, m, C**H**$_A$H$_B$CH$_A$**H**$_B$CCH$_3$), 1.55-1.40 (1H, m, CH$_A$**H**$_B$CH$_2$CCH$_3$), 1.16 (3H, s, C**H**$_3$C=O); ^{13}C NMR (100.6 MHz, CDCl$_3$) δ 211.9 (C), 211.8 (C), 197.4 (C), 144.3 (C), 134.2 (C), 129.4 (2 x CH), 128.2 (2 x CH), 62.7 (C), 48.5 (CH), 41.4 (CH$_2$), 41.1 (CH$_2$), 40.7 (CH), 39.0 (CH$_2$), 25.7 (CH$_2$), 21.6 (CH$_3$), 16.8 (CH$_2$), 16.6 (CH$_3$); HRMS (ESI) Exact mass calculated for $C_{19}H_{22}NaO_3$ [M+Na]$^+$: 321.1461, found: 321.1451; Enantiomeric excess was determined by HPLC with a Chiralpak IA-3 column (80:20 *iso*-hexane:*i*-PrOH, 1.5 mL/min, 210 nm, 25 °C); t$_r$ (major) = 7.7 min, t$_r$ (minor) = 11.0 min; 86% ee.

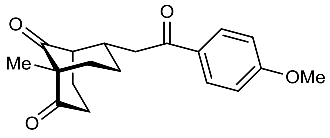

(1*R*,5*R*,6*S*)-6-[2-(4-Methoxyphenyl)-2-oxoethyl]-1-methylbicyclo[3.3.1]nonane-2,9-dione (6c). The title compound was prepared according to the Representative Procedure from enone **5c** (63 mg, 0.20 mmol). Purification by column chromatography (30 to 40% EtOAc/petroleum ether) gave a white solid (59 mg, 94%) as a >95:5 ratio of diastereomers. R$_f$ = 0.11 (20% EtOAc/petroleum

ether); m.p. 110-112 °C (CH_2Cl_2); $[\alpha]_D^{20}$ −11.4 (c 1.12, $CHCl_3$); IR 2937, 1731 (C=O), 1703 (C=O), 1676 (C=O), 1650, 1575, 1511, 1262, 1112, 831 cm^{-1}; ^{1}H NMR (400 MHz, $CDCl_3$) δ 7.92 (2H, d, *J* = 8.7 Hz, Ar**H**), 6.95 (2H, d, *J* = 8.7 Hz, Ar**H**), 3.88 (3H, s, OC**H**$_3$), 3.01 (1H, dd, *J* = 15.9, 7.7 Hz, C**H**$_A$H$_B$C(O)Ar), 2.95 (1H, dd, *J* = 15.9, 6.2 Hz, CH$_A$**H**$_B$C(O)Ar), 2.91-2.85 (1H, m, C**H**C=O), 2.82-2.72 (1H, m, C**H**CH$_2$C(O)Ar), 2.60 (1H, app dt, *J* = 16.2, 6.5 Hz, CH$_2$C**H**$_A$H$_B$C=O), 2.46-2.35 (1H, m, CH$_2$CH$_A$**H**$_B$C=O), 2.27-2.17 (1H, m, C**H**$_A$H$_B$CCH$_3$), 2.05-1.93 (2H, m, C**H**$_2$CH$_2$C=O), 1.78-1.64 (2H, m, C**H**$_A$H$_B$CH$_A$**H**$_B$CCH$_3$), 1.55-1.41 (1H, m, CH$_A$**H**$_B$CH$_2$CCH$_3$), 1.16 (3H, s, C**H**$_3$); ^{13}C NMR (100.6 MHz, $CDCl_3$) δ 211.3 (C), 211.5 (C), 196.3 (C), 163.7 (C), 130.4 (2 x CH), 129.7 (C), 113.9 (2 x CH), 62.7 (C), 55.5 (CH_3), 48.5 (CH), 41.2 (CH_2), 41.1 (CH_2), 40.8 (CH), 39.0 (CH_2), 25.7 (CH_2), 16.8 (CH_2), 16.6 (CH_3); HRMS (ESI) Exact mass calculated for $C_{19}H_{22}NaO_4$ $[M+Na]^+$: 337.1410, found: 337.1406; Enantiomeric excess was determined by HPLC with a Chiralpak IA-3 column (60:40 *iso*-hexane:*i*-PrOH, 1.5 mL/min, 254 nm, 25 °C); t_r (major) = 6.4 min, t_r (minor) = 11.4 min; 87% ee.

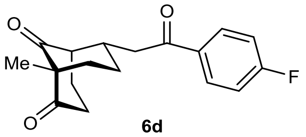

6d

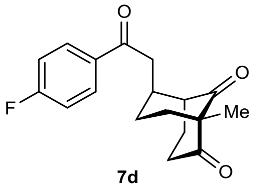

7d

(1*R*,5*R*,6*S*)-6-[2-(4-Fluorophenyl)-2-oxoethyl]-1-methylbicyclo[3.3.1]nonane-2,9-dione (6d) and (1*S*,5*S*,6*S*)-6-[2-(4-fluorophenyl)-2-oxoethyl]-1-methylbicyclo[3.3.1]nonane-2,9-dione (7d). The title compounds **6d** and **7d** were prepared according to the Representative Procedure from enone **5d** (60 mg, 0.20 mmol). Purification by column chromatography (20% EtOAc/petroleum ether) gave **6d** as a colorless oil (49 mg, 82%) and **7d** as a colorless film (8 mg, 13%).

Data for **6d**: R_f = 0.56 (40% EtOAc/petroleum ether); $[\alpha]_D^{20}$ −14.5 (*c* 1.18, $CHCl_3$); IR 2935, 1731 (C=O), 1703 (C=O), 1599, 1508, 1453, 1411, 1276, 983, 838 cm^{-1}; ^{1}H NMR (400 MHz, $CDCl_3$) δ 8.03-7.91 (2H, m, Ar**H**), 7.21-7.10 (2H, m, Ar**H**), 3.05 (1H, dd, *J* = 15.8, 7.1 Hz, C**H**$_A$H$_B$C(O)Ar), 2.98 (1H, dd, *J* = 15.8, 5.5 Hz, CH$_A$**H**$_B$C(O)Ar), 2.92-2.85 (1H, m, C**H**C=O), 2.84-2.72 (1H, m, C**H**CH$_2$C(O)Ar), 2.61 (1H, ddd, *J* = 16.2, 7.0, 5.9 Hz, CH$_2$C**H**$_A$H$_B$C=O), 2.41 (1H, app dt, *J* = 16.2, 8.8 Hz, CH$_2$CH$_A$**H**$_B$C=O), 2.29-2.19 (1H, m, C**H**$_A$H$_B$CCH$_3$), 2.06-1.93 (2H, m, C**H**$_2$CH$_2$C=O), 1.80-1.62 (2H, m, C**H**$_A$H$_B$CH$_A$**H**$_B$CCH$_3$), 1.56-1.43 (1H, m, CH$_A$**H**$_B$CH$_2$CCH$_3$), 1.17 (3H, s, C**H**$_3$); ^{13}C NMR (100.6 MHz, $CDCl_3$) δ 211.8 (C), 211.7 (C), 196.0 (C), 165.9 (C, d, *J* = 255.6 Hz), 133.0 (C, d, *J* = 3.1 Hz), 130.7 (CH, d, *J* = 9.4 Hz), 115.9 (CH, d, *J* = 21.9 Hz), 62.7 (C), 48.4 (CH), 41.4 (CH_2), 41.0 (CH), 40.5 (CH_2), 38.9 (CH_2), 25.7 (CH_2), 16.8 (CH_2), 16.6 (CH_3); ^{19}F NMR (376 MHz, $CDCl_3$) δ −104.4; HRMS (ESI) Exact mass calculated for $C_{18}H_{19}FNaO_3$ $[M+Na]^+$: 325.1210, found: 325.1203; Enantiomeric excess was determined by HPLC with a Chiralpak IA-3 column

(90:10 *iso*-hexane:*i*-PrOH, 1.5 mL/min, 254 nm, 25 °C); t_r (major) = 7.0 min, t_r (minor) = 8.6 min; 86% ee.

Data for **7d**: R_f = 0.63 (40% EtOAc/petroleum ether); $[\alpha]_D^{20}$ +20.2 (*c* 0.45, $CHCl_3$); IR 2931, 1731 (C=O), 1700 (C=O), 1599, 1507, 1456, 1374, 1157, 999, 837 cm^{-1}; ^1H NMR (400 MHz, $CDCl_3$) δ 8.04-7.93 (2H, m, Ar**H**), 7.22-7.10 (2H, m, Ar**H**), 3.10 (1H, dd, *J* = 16.2, 6.1 Hz, $C\mathbf{H_A}H_BC(O)Ar$), 3.04-2.95 (1H, m, C**H**$CH_2C(O)Ar$), 2.89 (1H, dd, *J* = 16.2, 6.5 Hz, $CH_A\mathbf{H_B}C(O)Ar$), 2.78-2.67 (2H, m, C**H**C=O and $CH_2C\mathbf{H_A}CH_BC{=}O$), 2.48-2.18 (3H, m, $CH_2CH_AC\mathbf{H_B}C{=}O$, $C\mathbf{H_A}H_BCCH_3$, and $C\mathbf{H_A}H_BCH_2C{=}O$), 2.11-1.92 (2H, m, $C\mathbf{H_A}H_BCH_2CCH_3$ and $CH_A\mathbf{H_B}CH_2C{=}O$), 1.82 (1H, app td , *J* = 13.8, 4.6 Hz, $CH_A\mathbf{H_B}CCH_3$), 1.57-1.48 (1H, m, $CH_A\mathbf{H_B}CH_2CCH_3$ overlapped with H_2O), 1.18 (3H, s, $C\mathbf{H_3}$); ^{13}C NMR (100.6 MHz, $CDCl_3$) δ 213.2 (C), 211.8 (C), 196.5 (C), 165.6 (C, d, *J* = 255.6 Hz), 133.3 (CH, d, *J* = 3.2 Hz), 130.7 (2 x CH, d, *J* = 9.3 Hz), 115.8 (2 x CH, d, *J* = 21.9 Hz), 63.1 (C), 49.7 (CH), 41.7 (CH_2), 40.6 (CH), 39.2 (CH_2), 38.8 (CH_2), 23.6 (CH_2), 22.4 (CH_2), 16.8 (CH_3); ^{19}F NMR (376 MHz, $CDCl_3$) δ −104.6; HRMS (ESI) Exact mass calculated for $C_{18}H_{19}FNaO_3$ $[M+Na]^+$: 325.1216, found: 325.1244; Enantiomeric excess was determined by HPLC with a Chiralpak IA-3 column (90:10 *iso*-hexane:*i*-PrOH, 1.5 mL/min, 230 nm, 25 °C); t_r (major) = 7.5 min, t_r (minor) = 9.9 min; 86% ee.

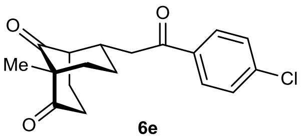

6e

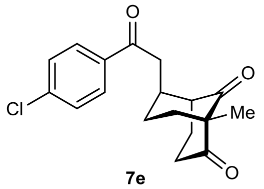

7e

(1*R*,5*R*,6*S*)-6-[2-(4-Chlorophenyl)-2-oxoethyl]-1-methylbicyclo[3.3.1]nonane-2,9-dione (6e) and (1*S*,5*S*,6*S*)-6-[2-(4-chlorophenyl)-2-oxoethyl]-1-methylbicyclo[3.3.1]nonane-2,9-dione (7e). The title compounds **6e** and **7e** were prepared according to the Representative Procedure from enone **5e** (64 mg, 0.20 mmol). Purification by column chromatography (20 to 30% EtOAc/*iso*-hexane) gave **6e** as a white solid (47 mg, 73%) and **7e** as a colorless film (12 mg, 19%).

Data for **6e**: R_f = 0.48 (40% EtOAc/petroleum ether); m.p. 155-157 °C (*iso*-hexane/CH_2Cl_2); $[\alpha]_D^{20}$ −22.1 (c 1.80, $CHCl_3$); IR 3018, 2937, 1731 (C=O), 1703 (C=O), 1626, 1590, 1453, 1401, 1277, 1094, 982 cm^{-1}; ^1H NMR (400 MHz, $CDCl_3$) δ 7.88 (2H, d, *J* = 8.8 Hz, Ar**H**), 7.46 (2H, d, *J* = 8.6 Hz, Ar**H**), 3.04 (1H, dd, *J* = 16.5, 7.6 Hz, $C\mathbf{H_A}H_BC(O)Ar$), 2.98 (1H, dd, *J* = 16.5, 6.7 Hz, $CH_A\mathbf{H_B}C(O)Ar$), 2.91-2.84 (1H, m, C**H**C=O), 2.83-2.72 (1H, m, C**H**$CH_2C(O)Ar$), 2.60 (1H, ddd, *J* = 16.2, 6.9, 6.0 Hz, $CH_2C\mathbf{H_A}H_BC{=}O$), 2.40 (1H, dt, *J* = 16.2, 8.9 Hz $CH_2CH_A\mathbf{H_B}C{=}O$), 2.28-2.19 (1H, m, $C\mathbf{H_A}H_BCCH_3$), 2.08-1.90 (2H, m, $C\mathbf{H_2}CH_2C{=}O$), 1.80-1.65 (2H, m, $C\mathbf{H_A}H_BCH_A\mathbf{H_B}CCH_3$), 1.54-1.40 (1H, m, $CH_A\mathbf{H_B}CH_2CCH_3$), 1.17 (3H, s, $C\mathbf{H_3}$); ^{13}C NMR (125.8 MHz, $CDCl_3$) δ 211.8 (C), 211.6 (C), 196.4 (C), 139.9 (C), 134.9 (C), 129.4 (2 x CH), 129.1 (2 x CH), 62.7 (C), 48.3 (CH), 41.4 (CH_2), 41.0 (CH_2), 40.4 (CH), 38.9 (CH_2), 25.6 (CH_2), 16.7 (CH_2), 16.6 (CH_3); HRMS

(ESI) Exact mass calculated for $C_{18}H_{19}ClNaO_3$ $[M+Na]^+$: 341.0915, found: 341.0912; Enantiomeric excess was determined by HPLC with a Chiralpak IA-3 column (60:40 *iso*-hexane:*i*-PrOH, 1.5 mL/min, 254 nm, 25 °C); t_r (major) = 5.5 min, t_r (minor) = 6.7 min; 87% ee.

Slow diffusion of cyclohexane into a solution of **6e** in CH_2Cl_2 gave crystals that were suitable for X-ray crystallography:

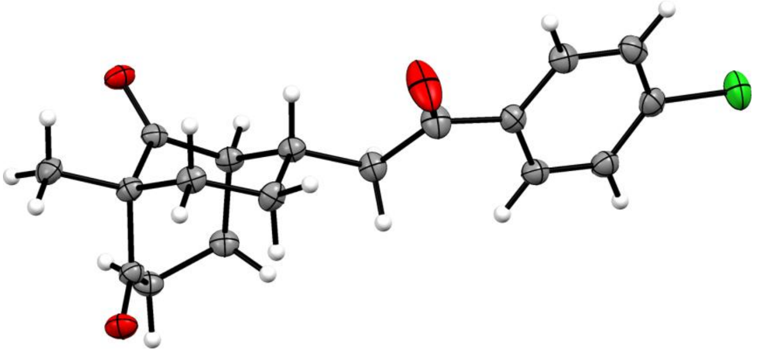

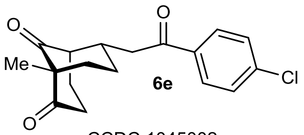

CCDC 1045002

Data for **7e**: R_f = 0.56 (40% EtOAc/petroleum ether); $[\alpha]_D^{20}$ +33.1 (c 0.41, $CHCl_3$); IR 3011, 2937, 1733 (C=O), 1700 (C=O), 1590, 1455, 1375, 1283, 1094, 999 cm^{-1}; ^1H NMR (400 MHz, $CDCl_3$) δ 7.89 (2H, d, *J* = 8.6 Hz, Ar**H**), 7.46 (2H, d, *J* = 8.6 Hz, Ar**H**), 3.08 (1H, dd, *J* = 16.8, 6.4 Hz, C**H**$_{\mathbf{A}}$H$_B$C(O)Ar), 3.03-2.93 (1H, m, C**H**CH$_2$C(O)Ar), 2.88 (1H, dd, *J* = 16.8, 8.8 Hz, CH$_A$**H**$_{\mathbf{B}}$C(O)Ar), 2.78-2.66 (2H, m, C**H**C=O and CH$_2$C**H**$_{\mathbf{A}}$H$_B$C=O), 2.40 (1H, dt, *J* = 15.9, 9.2 Hz, CH$_2$CH$_A$**H**$_{\mathbf{B}}$C=O), 2.33-2.19 (2H, m, C**H**$_A$H$_B$CH$_2$C=O and C**H**$_{\mathbf{A}}$H$_B$CCH$_3$), 2.09-1.89 (2H, m, C**H**$_{\mathbf{A}}$H$_B$CH$_2$CCH$_3$ and CH$_A$**H**$_{\mathbf{B}}$CH$_2$C=O), 1.82 (1H, td, *J* = 13.7, 4.6 Hz, CH$_A$**H**$_{\mathbf{B}}$CCH$_3$), 1.55-1.47 (1H, m, CH$_A$**H**$_{\mathbf{B}}$CH$_2$CCH$_3$), 1.17 (3H, s, C**H**$_3$); ^{13}C NMR (125.8 MHz, $CDCl_3$) δ 213.2 (C), 211.8 (C), 196.8 (C), 139.9 (C), 135.1 (C), 129.5 (2 x CH), 129.0 (2 x CH), 63.0 (C), 49.7 (CH), 41.6 (CH), 40.6 (CH_2), 39.2 (CH_2), 38.8 (CH_2), 23.6 (CH_2), 22.4 (CH_2), 16.8 (CH_3); HRMS (ESI) Exact mass calculated for $C_{18}H_{19}ClNaO_3$ $[M+Na]^+$: 341.0915, found: 341.0916; Enantiomeric excess was determined by HPLC with a Chiralpak IA-3 column (95:5 *iso*-hexane:*i*-PrOH, 1.5 mL/min, 254 nm, 25 °C); t_r (major) = 12.8 min, t_r (minor) = 17.4 min; 96% ee.

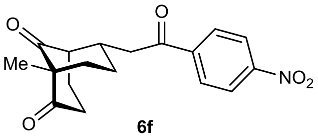

6f

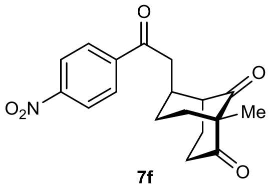

7f

(1*R*,5*R*,6*S*)-1-Methyl-6-[2-(4-nitrophenyl)-2-oxoethyl]bicyclo[3.3.1]nonane-2,9-dione (6f) and (1*S*,5*S*,6*S*)-1-Methyl-6-[2-(4-nitrophenyl)-2-oxoethyl]bicyclo[3.3.1]nonane-2,9-dione (7f). The title compounds **6f** and **7f** were prepared according to a modification of the Representative Procedure from enone **5f** (66 mg, 0.20 mmol) using toluene (2 mL) as solvent. Purification by column chromatography (20 to 40% EtOAc/*iso*-hexane) gave **6f** as a pale yellow solid (56 mg, 85%) and **7f** as an off-white solid (10 mg, 15%).

Data for **6f**: R_f = 0.35 (40% EtOAc/petroleum ether); m.p. 142-144 °C (*iso*-hexane/CH_2Cl_2); $[\alpha]^{20}_{D}$ −23.7 (c 0.80, $CHCl_3$); IR 2934, 2860, 1732 (C=O), 1702 (C=O), 1626, 1604, 1530 (NO_2), 1348 (NO_2), 1318, 1109, 855 cm^{-1}; ^1H NMR (400 MHz, $CDCl_3$) δ 8.37-8.29 (2H, m, Ar**H**), 8.14-8.07 (2H, m, Ar**H**), 3.12 (1H, dd, *J* = 17.1, 7.6 Hz, C$\mathbf{H_A}$$H_B$C(O)Ar), 3.06 (1H, dd, *J* = 17.1, 6.3 Hz, C$H_A$$\mathbf{H_B}$C(O)Ar), 2.89 (1H, dt, *J* = 8.3, 3.0 Hz, C**H**C=O), 2.86-2.73 (1H, m, C**H**CH_2C(O)Ar), 2.68-2.53 (1H, m, CH_2C$\mathbf{H_A}$$H_B$C=O), 2.41 (1H, dt, *J* = 16.2, 8.9 Hz, CH_2C$H_A$$\mathbf{H_B}$C=O), 2.29-2.20 (1H, m, C$\mathbf{H_A}$$H_BCCH_3$), 2.10-1.89 (2H, m, C$\mathbf{H_2}$$CH_2$C=O), 1.81-1.62 (2H, m, C$\mathbf{H_A}$$H_BCH_A$$\mathbf{H_B}CCH_3$), 1.56-1.41 (1H, m, C$H_A$$\mathbf{H_B}$$CH_2CCH_3$), 1.16 (3H, s, C$\mathbf{H_3}$); ^{13}C NMR (100.6 MHz, $CDCl_3$) δ 211.6 (C), 211.4 (C), 196.0 (C), 150.5 (C), 140.9 (C), 129.1 (2 x CH), 124.0 (2 x CH), 62.6 (C), 48.3 (CH), 41.9 (CH_2), 40.9 (CH_2), 40.1 (CH), 38.9 (CH_2), 25.6 (CH_2), 16.7 (CH_3), 16.6 (CH_2); HRMS (ESI) Exact mass calculated for $C_{18}H_{19}NNaO_5$ $[M+Na]^+$: 352.1155, found: 352.1145; Enantiomeric excess was determined by HPLC with a Chiralpak IA-3 column (60:40 *iso*-hexane:*i*-PrOH, 1.5 mL/min, 254 nm, 25 °C); t_r (major) = 11.8 min, t_r (minor) = 13.8 min; 72% ee.

Slow diffusion of cyclohexane into a solution of **6e** in CH_2Cl_2 gave crystals that were suitable for X-ray crystallography:

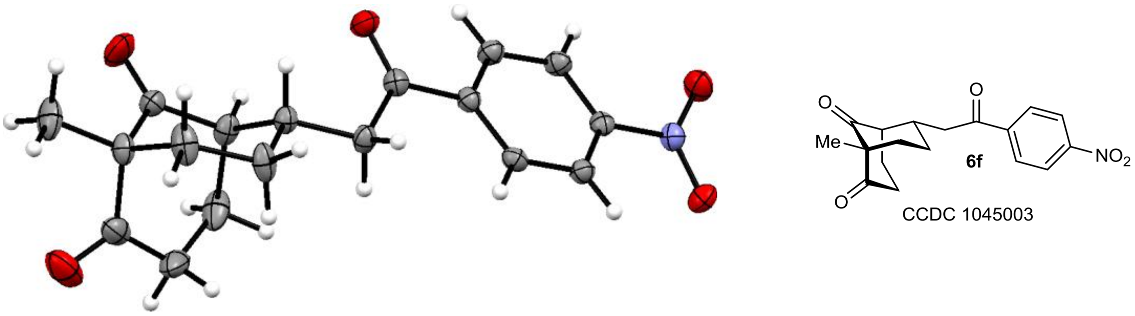

Data for **7f**: R_f = 0.42 (40% EtOAc/petroleum ether); m.p. 102-104 °C (cyclohexane/CH_2Cl_2); $[\alpha]^{20}_{D}$ +61.6 (c 0.27, $CHCl_3$); IR 3012, 2937, 1733 (C=O), 1700 (C=O), 1604, 1530 (NO_2), 1455, 1347 (NO_2), 1013, 855 cm^{-1}; ^1H NMR (400 MHz, $CDCl_3$) δ 8.36-8.30 (2H, m, Ar**H**), 8.13-8.07 (2H, m, Ar**H**), 3.17 (1H, dd, *J* = 17.0, 6.3 Hz, C$\mathbf{H_A}$$H_B$C(O)Ar), 3.05-2.98 (1H, m, C**H**CH_2C(O)Ar), 2.94 (1H, dd, *J* = 17.0, 6.6 Hz, C$H_A$$\mathbf{H_B}$C(O)Ar), 2.78-2.67 (2H, m, C**H**C=O and CH_2C$\mathbf{H_A}$$H_B$C=O), 2.40 (1H, dt, *J* = 15.6, 9.2 Hz, CH_2C$H_A$$\mathbf{H_B}$C=O), 2.35-2.21 (2H, m, C$\mathbf{H}_A$$H_B$$CH_2$C=O and C$\mathbf{H_A}$$H_BCCH_3$), 2.12-1.90 (2H, m, C$\mathbf{H_A}$$H_B$$CH_2CCH_3$ and C$H_A$$\mathbf{H_B}$$CH_2$C=O), 1.82 (1H, td, *J* = 13.8, 4.6 Hz, C$H_A$$\mathbf{H_B}CCH_3$), 1.57-1.47 (1H, m, C$H_A$$\mathbf{H_B}$$CH_2CCH_3$), 1.17 (3H, s, C$\mathbf{H_3}$); ^{13}C NMR (100.6 MHz, $CDCl_3$) δ 213.1 (C), 211.6 (C), 196.6 (C), 150.5 (C), 141.1 (C), 129.1 (2 x CH), 124.0 (2 x CH), 63.0 (C), 49.5 (CH), 41.5 (CH), 41.3 (CH_2), 39.2 (CH_2), 38.9 (CH_2), 23.6 (CH_2), 22.3 (CH_2), 16.8 (CH_3); HRMS (ESI) Exact mass calculated for $C_{18}H_{19}NNaO_5$ $[M+Na]^+$: 352.1155, found: 352.1150; Enantiomeric excess was determined by HPLC with a Chiralpak IA-3 column (60:40 *iso*-hexane:*i*-PrOH, 1.5 mL/min, 254 nm, 25 °C); t_r (major) = 6.8 min, t_r (minor) = 9.0 min; 88% ee.

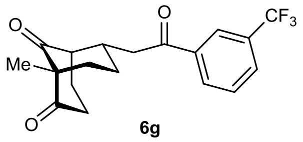
6g

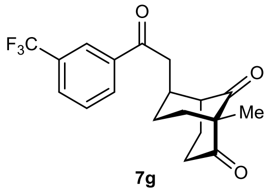
7g

(1*R*,5*R*,6*S*)-1-Methyl-6-{2-oxo-2-[3-(trifluoromethyl)phenyl]ethyl}bicyclo[3.3.1]nonane-2,9-dione (6g) and (1*S*,5*S*,6*S*)-1-methyl-6-{2-oxo-2-[3-(trifluoromethyl)phenyl]ethyl}bicyclo[3.3.1]nonane-2,9-dione (7g). The title compounds **6g** and **7g** were prepared according to a modification of the Representative Procedure from enone **5g** (71 mg, 0.20 mmol) using cyclohexane (4 mL) as solvent. Purification by column chromatography (20 to 30% EtOAc/*iso*-hexane) gave **6g** as a pale yellow solid (48 mg, 68%) and **7g** as a pale yellow film (15 mg, 21%).

Data for **6g**: R_f = 0.28 (30% EtOAc/petroleum ether); m.p. 94-96 °C (*iso*-hexane/CH_2Cl_2); $[\alpha]_D^{20}$ −23.6 (c 1.90, $CHCl_3$); IR 3024, 2937, 1732 (C=O), 1702 (C=O), 1612, 1453, 1333, 1173, 1137, 1072 cm^{-1}; ^{1}H NMR (300 MHz, $CDCl_3$) δ 8.18 (1H, s, Ar**H**), 8.13 (1H, d, *J* = 7.8 Hz, Ar**H**), 7.85 (1H, d, *J* = 7.8 Hz, Ar**H**), 7.64 (1H, t, *J* = 7.8 Hz, Ar**H**), 3.11 (1H, dd, *J* = 16.0, 6.6 Hz, C$\mathbf{H_A}$$H_B$C(O)Ar), 3.03 (1H, dd, *J* = 16.0, 5.2 Hz, C$H_A$$\mathbf{H_B}$C(O)Ar), 2.89 (1H, ddd, *J* = 7.0, 6.7, 3.4 Hz, C**H**C=O), 2.86-2.74 (1H, m, C**H**CH_2C(O)Ar), 2.61 (1H, ddd, *J* = 16.2, 7.1, 5.9 Hz, CH_2C$\mathbf{H_A}$$H_B$C=O), 2.41 (1H, dt, *J* = 16.2, 8.8 Hz, CH_2C$H_A$$\mathbf{H_B}$C=O), 2.30-2.19 (1H, m, C$\mathbf{H_A}$$H_BCCH_3$), 2.11-1.90 (2H, m, C$\mathbf{H_2}$$CH_2$C=O), 1.82-1.65 (2H, m, C$\mathbf{H_A}$$H_BCH_A$$\mathbf{H_B}CCH_3$), 1.58-1.37 (1H, m, C$H_A$$\mathbf{H_B}$$CH_2CCH_3$), 1.16 (3H, s, C$\mathbf{H_3}$); ^{13}C NMR (100.6 MHz, $CDCl_3$) δ 211.7 (C), 211.5 (C), 196.2 (C), 137.1 (C), 131.4 (C, q, *J* = 33.0 Hz), 131.2 (CH), 129.8 (CH, q, *J* = 3.5 Hz), 129.5 (CH), 124.8 (CH, q, *J* = 3.8 Hz), 123.6 (C, q, *J* = 272.5 Hz), 62.7 (C), 48.3 (CH), 41.5 (CH_2), 41.0 (CH_2), 40.2 (CH), 38.9 (CH_2), 25.7 (CH_2) 16.8 (CH_2), 16.6 (CH_3); HRMS (ESI) Exact mass calculated for $C_{19}H_{19}F_3NaO_3$ $[M+Na]^+$: 375.1179, found: 375.1172; Enantiomeric excess was determined by HPLC with a Chiralpak IA-3 column (95:5 *iso*-hexane:*i*-PrOH, 1.5 mL/min, 230 nm, 25 °C); t_r (major) = 15.0 min, t_r (minor) = 17.5 min; 86% ee.

Data for **7g**: R_f = 0.33 (30% EtOAc/petroleum ether); $[\alpha]_D^{20}$ +42.8 (*c* 1.30, $CHCl_3$); IR 3011, 2937, 1732 (C=O), 1699 (C=O), 1613, 1489, 1375, 1332, 1173, 1137, 1073, 1002, 927 cm^{-1}; ^{1}H NMR (400 MHz, $CDCl_3$) δ 8.19 (1H, s, Ar**H**), 8.12 (1H, d, *J* = 7.9 Hz, Ar**H**), 7.85 (1H, d, *J* = 7.9 Hz, Ar**H**), 7.63 (1H, t, *J* = 7.9 Hz, Ar**H**), 3.13 (1H, dd, *J* = 16.8, 6.1 Hz, C$\mathbf{H_A}$$H_B$C(O)Ar), 3.05-2.97 (1H, m, C**H**CH_2C(O)Ar), 2.94 (1H, dd, *J* = 16.8, 6.7 Hz, C$H_A$$\mathbf{H_B}$C(O)Ar), 2.79-2.66 (2H, m, C**H**C=O and CH_2C$\mathbf{H_A}$$H_B$C=O), 2.40 (1H, dt, *J* = 15.8, 9.2 Hz, CH_2C$H_A$$\mathbf{H_B}$C=O), 2.35-2.20 (2H, m, C$\mathbf{H_A}$$H_B$$CH_2$C=O and C$\mathbf{H_A}$$H_BCCH_3$), 2.11-1.90 (2H, m, C$\mathbf{H_A}$$H_B$$CH_2CCH_3$ and C$H_A$$\mathbf{H_B}$$CH_2$C=O), 1.83 (1H, td, *J* = 13.7, 4.6 Hz, C$H_A$$\mathbf{H_B}CCH_3$), 1.58-1.48 (1H, m, C$H_A$$\mathbf{H_B}$$CH_2CCH_3$), 1.17 (3H, s, C$\mathbf{H_3}$); ^{13}C NMR (100.6 MHz, $CDCl_3$) δ 213.1 (C), 211.8 (C), 196.7 (C), 137.3 (C), 131.4 (C, q, *J* = 33.1 Hz), 131.2 (CH), 129.8 (CH, q, *J* = 3.6 Hz), 129.4 (CH), 124.8 (C, q, *J* = 3.7 Hz), 123.6 (C, q,

J = 272.5 Hz), 63.0 (C), 49.6 (CH), 41.5 (CH), 40.8 (CH_2), 39.2 (CH_2), 38.8 (CH_2), 23.6 (CH_2), 22.4 (CH_2), 16.8 (CH_3); HRMS (ESI) Exact mass calculated for $C_{19}H_{19}F_3NaO_3$ $[M+Na]^+$: 375.1179, found: 375.1184; Enantiomeric excess was determined by HPLC with a Chiralpak IA-3 column (95:5 *iso*-hexane:*i*-PrOH, 1.5 mL/min, 230 nm, 25 °C); t_r (major) = 8.6 min, t_r (minor) = 13.0 min; 94% ee.

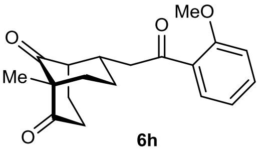

6h

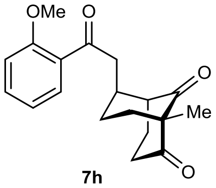

7h

(1*R*,5*R*,6*S*)-6-[2-(2-Methoxyphenyl)-2-oxoethyl]-1-methylbicyclo[3.3.1]nonane-2,9-dione (6h) and (1*S*,5*S*,6*S*)-6-[2-(2-methoxyphenyl)-2-oxoethyl]-1-methylbicyclo[3.3.1]nonane-2,9-dione (7h). The title compounds **6h** and **7h** were prepared according to the Representative Procedure from enone **5h** (63 mg, 0.20 mmol). Purification by column chromatography (20% EtOAc/petroleum ether) gave **6h** as a pale yellow solid (38 mg, 60%) and **7h** as a pale yellow oil (15 mg, 24%).

Data for **6h**: R_f = 0.23 (20% EtOAc/petroleum ether); m.p. 133-135 °C (CH_2Cl_2); $[\alpha]_D^{20}$ −24.0 (*c* 1.12, $CHCl_3$); IR 2937, 1730 (C=O), 1703 (C=O), 1680 (C=O), 1485, 1466, 1437, 1245, 1025, 984 cm^{-1}; ^1H NMR (400 MHz, $CDCl_3$) δ 7.66 (1H, dd, *J* = 7.6, 1.8 Hz, Ar**H**), 7.48 (1H, ddd, *J* = 8.4, 7.6, 1.8 Hz, Ar**H**), 7.03 (1H, app td, *J* = 7.6, 0.9 Hz, Ar**H**), 6.97 (1H, app d, *J* = 8.4 Hz, Ar**H**), 3.92 (3H, s, $OC\mathbf{H}_3$), 3.05 (2H, d, *J* = 6.9 Hz, $C\mathbf{H}_2C(O)Ar$), 2.88-2.83 (1H, m, C**H**C=O), 2.79-2.68 (1H, m, $C\mathbf{H}CH_2C(O)Ar$), 2.58 (1H, app dt, *J* = 16.4, 6.7 Hz, $CH_2C\mathbf{H_A}H_BC{=}O$), 2.39 (1H, app dt, *J* = 16.4, 8.6 Hz, $CH_2CH_A\mathbf{H_B}C{=}O$), 1.99-1.92 (2H, m, $C\mathbf{H}_2CH_2C{=}O$), 1.78-1.62 (2H, m, $C\mathbf{H_A}H_BCH_A\mathbf{H_B}CCH_3$), 1.53-1.42 (1H, m, $CH_A\mathbf{H_B}CH_2CCH_3$), 1.15 (3H, s, $CC\mathbf{H}_3$); ^{13}C NMR (100.6 MHz, $CDCl_3$) δ 212.12 (C), 212.06 (C), 200.1, (C), 158.3 (C), 133.8 (CH), 130.3 (CH), 128.0 (C), 120.9 (CH), 111.6 (CH), 62.8 (C), 55.6 (CH_3), 48.6 (CH), 46.7 (CH_2), 41.2 (CH_2), 40.6 (CH), 39.0 (CH_2), 25.7 (CH_2), 16.9 (CH_2), 16.6 (CH_3); HRMS (ESI) Exact mass calculated for $C_{19}H_{22}NaO_4$ $[M+Na]^+$: 337.1410, found: 337.1397; Enantiomeric excess was determined by HPLC with a Chiralpak IA-3 column (90:10 *iso*-hexane:*i*-PrOH, 1.5 mL/min, 254 nm, 25 °C); t_r (major) = 11.8 min, t_r (minor) = 13.7 min; 83% ee.

Data for **7h**: R_f = 0.29 (20% EtOAc/petroleum ether); $[\alpha]_D^{20}$ −18.4 (*c* 0.38, $CHCl_3$); IR 2928, 1727 (C=O), 1700 (C=O), 1598, 1486, 1465, 1375, 1290, 1057, 883 cm^{-1}; ^1H NMR (400 MHz, $CDCl_3$) δ 7.65 (1H, dd, *J* = 7.7, 1.8 Hz, Ar**H**), 7.48 (1H, ddd, *J* = 8.4, 7.3, 1.8 Hz, Ar**H**), 7.04-6.95 (2H, m, Ar**H**), 3.91 (3H, s, $OC\mathbf{H}_3$), 3.10 (1H, dd, *J* = 17.0, 6.2 Hz, $C\mathbf{H_A}H_BC(O)Ar$), 3.04-2.91 (2H, m, $C\mathbf{H}CH_A\mathbf{H_B}C(O)Ar$), 2.77-2.66 (2H, m, C**H**C=O and $CH_2C\mathbf{H_A}H_BC{=}O$), 2.39 (1H, app dt, *J* = 16.2, 9.0 Hz, $CH_2CH_A\mathbf{H_B}C{=}O$), 2.29-2.16 (2H, m, $C\mathbf{H_A}H_BCCH_3$ and $C\mathbf{H_A}H_BCH_2C{=}O$), 2.03-1.89 (2H, m, $C\mathbf{H_A}H_BCH_2CCH_3$ and $CH_A\mathbf{H_B}CH_2C{=}O$), 1.85 (1H, ddd, *J* = 18.0, 13.0, 3.4 Hz, $CH_A\mathbf{H_B}CCH_3$),

1.55-1.47 (1H, m, $CH_A\mathbf{H_B}CH_2CCH_3$), 1.15 (3H, s, $C\mathbf{H}_3$); ^{13}C NMR (100.6 MHz, $CDCl_3$) δ 213.1 (C), 212.2 (C), 200.5, (C), 158.4 (C), 133.6 (CH), 130.1 (CH), 128.3 (C), 120.8 (CH), 111.6 (CH), 63.1 (C), 55.5 (CH_3), 49.8 (CH), 46.0 (CH_2), 41.7 (CH_2), 39.1 (CH), 38.8 (CH_2), 23.5 (CH_2), 22.5 (CH_2), 16.8 (CH_3), HRMS (ESI) Exact mass calculated for $C_{19}H_{22}NaO_4$ $[M+Na]^+$: 337.1410, found: 337.1394; Enantiomeric excess was determined by HPLC with a Chiralpak IA-3 column (90:10 *iso*-hexane:*i*-PrOH, 1.5 mL/min, 254 nm, 25 °C); t_r (major) = 8.6 min, t_r (minor) = 9.7 min; 93% ee.

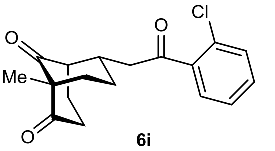

6i

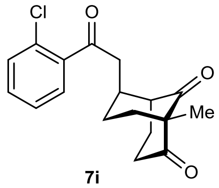

7i

(1*R*,5*R*,6*S*)-6-[2-(2-Chlorophenyl)-2-oxoethyl]-1-methylbicyclo[3.3.1]nonane-2,9-dione (6i) and (1*S*,5*S*,6*S*)-6-[2-(2-chlorophenyl)-2-oxoethyl]-1-methylbicyclo[3.3.1]nonane-2,9-dione (7i). The title compounds **6i** and **7i** were prepared according to the Representative Procedure from enone **1i** (64 mg, 0.20 mmol). Purification by column chromatography (20 to 25% EtOAc/*iso*-hexane) gave **6i** as a colorless film (48 mg, 75%) and **7i** as a colorless film (13 mg, 20%).

Data for **6i**: R_f = 0.25 (30% EtOAc/petroleum ether); $[\alpha]_D^{20}$ −15.0 (*c* 1.00, $CHCl_3$); IR 3043, 2938, 1731 (C=O), 1703 (C=O), 1592, 1469, 1453, 1433, 1376, 982 cm^{-1}; ^{1}H NMR (400 MHz, $CDCl_3$) δ 7.46-7.38 (3H, m, Ar**H**), 7.37-7.31 (1H, m, Ar**H**), 3.07 (1H, dd, *J* = 16.9, 7.6 Hz, $C\mathbf{H_A}H_BC(O)Ar$), 3.01 (1H, dd, *J* = 16.9, 6.2 Hz, $CH_A\mathbf{H_B}C(O)Ar$), 2.92-2.85 (1H, m, C**H**C=O), 2.81-2.70 (1H, m, C**H**$CH_2C(O)Ar$), 2.57 (1H, ddd, *J* = 16.3, 7.2, 5.9 Hz, $CH_2C\mathbf{H_A}H_BC$=O), 2.39 (1H, app dt, *J* = 16.3, 8.8 Hz, $CH_2CH_A\mathbf{H_B}C$=O), 2.26-2.17 (1H, m, $C\mathbf{H_A}H_BCCH_3$), 2.04-1.86 (2H, m, $C\mathbf{H}_2CH_2C$=O), 1.81-1.64 (2H, m, $C\mathbf{H_A}H_BCH_A\mathbf{H_B}CCH_3$), 1.54-1.41 (1H, m, $CH_A\mathbf{H_B}CH_2CCH_3$), 1.16 (3H, s, $C\mathbf{H}_3$); ^{13}C NMR (100.6 MHz, $CDCl_3$) δ 211.8 (C), 211.6 (C), 201.0 (C), 139.2 (C), 132.0 (CH), 130.7 (CH), 130.6 (C), 128.7 (CH), 127.1 (CH), 62.7 (C), 48.3 (CH), 45.9 (CH_2), 41.0 (CH_2), 40.4 (CH), 38.9 (CH_2), 25.6 (CH_2), 16.8 (CH_2), 16.6 (CH_3); HRMS (ESI) Exact mass calculated for $C_{18}H_{20}ClO_3$ $[M+H]^+$: 319.1095, found: 319.1091; Enantiomeric excess was determined by HPLC with a Chiralpak IA-3 column (90:10 *iso*-hexane:*i*-PrOH, 1.5 mL/min, 210 nm, 25 °C); t_r (major) = 9.9 min, t_r (minor) = 11.6 min; 92% ee.

Data for **7i**: R_f = 0.31 (30% EtOAc/petroleum ether); $[\alpha]_D^{20}$ +22.8 (*c* 1.42, $CHCl_3$); IR 3011, 2938, 1732 (C=O), 1702 (C=O), 1591, 1470, 1455, 1433, 1375, 1287, 1069,1016 cm^{-1}; ^{1}H NMR (400 MHz, $CDCl_3$) δ 7.47-7.37 (3H, m, Ar**H**), 7.34 (1H, ddd, *J* = 7.3, 6.6, 2.1 Hz, Ar**H**), 3.13-2.86 (3H, m, $C\mathbf{H}C\mathbf{H}_2C(O)Ar$), 2.81-2.65 (2H, m, C**H**C=O and $CH_2C\mathbf{H_A}H_BC$=O), 2.39 (1H, dt, *J* = 15.9, 9.1 Hz, $CH_2CH_A\mathbf{H_B}C$=O), 2.28 (1H, ddd, *J* = 18.2, 9.1, 4.4 Hz, $C\mathbf{H_A}H_BCH_2C$=O), 2.25-2.18 (1H, m, $C\mathbf{H_A}H_BCCH_3$), 2.07-1.88 (2H, m, $C\mathbf{H_A}H_BCH_2CCH_3$ and $CH_A\mathbf{H_B}CH_2C$=O), 1.80 (1H, td, *J* = 13.7, 4.6 Hz, $CH_A\mathbf{H_B}CCH_3$), 1.59-1.49 (1H, m, $CH_A\mathbf{H_B}CH_2CCH_3$ overlapped with H_2O), 1.14 (3H, s,

C**H**$_3$); ^{13}C NMR (100.6 MHz, $CDCl_3$) δ 212.8 (C), 211.8 (C), 201.2 (C), 139.2 (C), 131.9 (CH), 130.7 (C), 130.6 (CH), 128.6 (CH), 127.0 (CH), 63.0 (C), 49.5 (CH), 45.3 (CH_2), 41.6 (CH), 39.1 (CH_2), 38.8 (CH_2), 23.4 (CH_2), 22.4 (CH_2), 16.8 (CH_3); HRMS (ESI) Exact mass calculated for $C_{18}H_{19}ClNaO_3$ $[M+Na]^+$: 341.0915, found: 341.0925; Enantiomeric excess was determined by HPLC with a Chiralpak IA-3 column (95:5 *iso*-hexane:*i*-PrOH, 1.5 mL/min, 210 nm, 25 °C); t_r (major) = 10.8 min, t_r (minor) = 12.1 min; 85% ee.

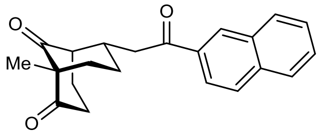

(1*R*,5*R*,6*S*)-1-Methyl-6-[2-(naphthalen-2-yl)-2-oxoethyl]bicyclo[3.3.1]nonane-2,9-dione (6j). The title compound was prepared according to the Representative Procedure from enone **5j** (67 mg, 0.20 mmol). Purification by column chromatography (20 to 25% EtOAc/*iso*-hexane) gave a yellow oil (64 mg, 96%) as a >95:5 ratio of diastereomers. R_f = 0.30 (30% EtOAc/petroleum ether); $[\alpha]_D^{20}$ −16.7 (*c* 2.20, $CHCl_3$); IR 3009, 2937, 1731 (C=O), 1703 (C=O), 1628, 1598, 1469, 1453, 1412, 1376, 1278, 1181, 1125, 823 cm^{-1}; ^{1}H NMR (400 MHz, $CDCl_3$) δ 8.45 (1H, s, Ar**H**), 8.04-7.95 (2H, m, Ar**H**), 7.94-7.87 (2H, m, Ar**H**), 7.67-7.55 (2H, m, Ar**H**), 3.21 (1H, dd, *J* = 16.4, 7.7 Hz, C$\mathbf{H_A}$$H_B$C(O)Ar), 3.15 (1H, dd, *J* = 16.4, 6.5 Hz, C$H_A$$\mathbf{H_B}$C(O)Ar), 2.94 (1H, dd, *J* = 9.4, 5.4 Hz, C**H**C=O), 2.91-2.81 (1H, m, C**H**CH_2C(O)Ar), 2.63 (1H, app dt, *J* = 16.3, 6.5 Hz, CH_2C$\mathbf{H_A}$$H_B$C=O), 2.42 (1H, app dt, *J* = 16.3, 8.8 Hz, CH_2C$H_A$$\mathbf{H_B}$C=O), 2.28-2.20 (1H, m, C$\mathbf{H_A}$$H_BCCH_3$), 2.03 (2H, app dt, *J* = 8.8, 6.1 Hz, C$\mathbf{H_2}$$CH_2$C=O), 1.85-1.67 (2H, m, C$\mathbf{H_A}$$H_BCH_A$$\mathbf{H_B}CCH_3$), 1.59-1.46 (1H, m, C$H_A$$\mathbf{H_B}$$CH_2CCH_3$), 1.17 (3H, s, C**H**$_3$); ^{13}C NMR (125.8 MHz, $CDCl_3$) δ 211.9 (C), 211.8 (C), 197.7 (C), 135.7 (C), 134.0 (C)¸132.4 (C), 129.7 (CH), 129.5 (CH), 128.7 (2 x CH), 127.8 (CH), 127.0 (CH), 123.7 (CH), 62.7 (C), 48.5 (CH), 41.5 (CH_2), 41.1 (CH_2), 40.7 (CH), 39.0 (CH_2), 25.7 (CH_2), 16.8 (CH_2), 16.6 (CH_3); HRMS (ESI) Exact mass calculated for $C_{22}H_{22}NaO_3$ $[M+Na]^+$: 357.1461, found: 357.1460; Enantiomeric excess was determined by HPLC with a Chiralpak IA-3 column (80:20 *iso*-hexane:*i*-PrOH, 1.5 mL/min, 254 nm, 25 °C); t_r (major) = 9.5 min, t_r (minor) = 11.4 min; 87% ee.

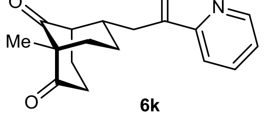

6k

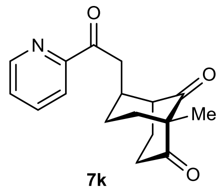

7k

(1*R*,5*R*,6*S*)-1-Methyl-6-[2-oxo-2-(pyridin-2-yl)ethyl]bicyclo[3.3.1]nonane-2,9-dione (6k) and (1*S*,5*S*,6*S*)-1 methyl-6-[2-oxo-2-(pyridin-2-yl)ethyl]bicyclo[3.3.1]nonane-2,9-dione (7k). The title compounds **6k** and **7k** were prepared according to the Representative Procedure from enone **5k** (57 mg, 0.20 mmol). Purification by column chromatography (20% EtOAc/petroleum ether) gave **6k** as a white solid (43 mg, 75%) and **7k** as a pale brown solid (11 mg, 19%).

Data for **6k**: R_f = 0.31 (40% EtOAc/petroleum ether); m.p. 101-103 °C (CH_2Cl_2); $[\alpha]_D^{20}$ −28.4 (*c* 1.06, $CHCl_3$); IR 2937, 1730 (C=O), 1701 (C=O), 1584, 1453, 1376, 1242, 996, 835 cm^{-1}; ^1H NMR (400 MHz, $CDCl_3$) δ 8.66 (1H, ddd, *J* = 4.7, 1.7, 1.0 Hz, Ar**H**), 8.03 (1H, dt, *J* = 7.9, 1.0 Hz Ar**H**), 7.85 (1H, td, *J* = 7.7, 1.7 Hz, Ar**H**), 7.49 (1H, ddd, *J* = 7.6, 4.7, 1.0 Hz, Ar**H**), 3.35 (1H, dd, *J* = 16.4, 7.4 Hz, C$\mathbf{H_A}$$H_B$C(O)Ar), 3.26 (1H, dd, *J* = 16.4, 6.3 Hz, C$H_A$$\mathbf{H_B}$C(O)Ar), 2.93-2.75 (2H, m, C**H**C=O and C**H**CH_2C(O)Ar), 2.61 (1H, ddd, *J* = 16.4, 7.2, 6.2 Hz, CH_2C$\mathbf{H_A}$$H_B$C=O), 2.39 (1H, app dt, *J* = 16.4, 8.2 Hz, CH_2C$H_A$$\mathbf{H_B}$C=O), 2.24-2.16 (1H, m, C$\mathbf{H_A}$$H_BCCH_3$), 2.11-1.91 (2H, m, C$\mathbf{H_2}$$CH_2$CO), 1.78-1.65 (2H, m, C$\mathbf{H_A}$$H_BCH_A$$\mathbf{H_B}CCH_3$), 1.57-1.44 (1H, m, C$H_A$$\mathbf{H_B}$$CH_2CCH_3$), 1.15 (3H, s, C$\mathbf{H_3}$); ^{13}C NMR (100.6 MHz, $CDCl_3$) δ 212.1 (C), 212.0 (C), 199.7 (C), 153.0 (C), 149.0 (CH), 137.0 (CH), 127.4 (CH), 121.9 (CH), 62.8 (C), 48.5 (CH), 41.2 (CH_2), 40.6 (CH), 40.5 (CH_2), 39.0 (CH_2), 25.8 (CH_2), 16.9 (CH_2), 16.5 (CH_3); HRMS (ESI) Exact mass calculated for $C_{17}H_{19}NO_3$ $[M+H]^+$: 286.1438, found: 286.1422; Enantiomeric excess was determined by HPLC with a Chiralpak IA-3 column (80:20 *iso*-hexane:*i*-PrOH, 1.5 mL/min, 230 nm, 25 °C); t_r (major) = 7.2 min, t_r (minor) = 9.1 min; 82% ee.

Data for **7k**: R_f = 0.36 (40% EtOAc/petroleum ether); m.p. 115-118 °C (CH_2Cl_2); $[\alpha]_D^{20}$ +36.0 (*c* 0.15, $CHCl_3$); IR 3010, 2937, 1732 (C=O), 1700 (C=O), 1585, 1455, 1375, 1243, 998, 926 cm^{-1}; ^1H NMR (400 MHz, $CDCl_3$) δ 8.67 (1H, ddd, *J* = 4.8, 1.7, 1.0 Hz, Ar**H**), 8.03 (1H, dt, *J* = 7.9, 1.0 Hz, Ar**H**), 7.84 (1H, td, *J* = 7.9, 1.7 Hz, Ar**H**), 7.49 (1H, ddd, *J* = 7.6, 4.8, 1.2 Hz, Ar**H**), 3.33 (1H, dd, *J* = 15.4, 4.9 Hz, C$\mathbf{H_A}$CH_BC(O)Ar), 3.28 (1H, dd, *J* = 15.4, 4.5 Hz, CH_AC$\mathbf{H_B}$C(O)Ar), 2.99-2.92 (1H, m, C**H**CH_2C(O)Ar), 2.82 (1H, td, *J* = 9.6, 2.8 Hz, C**H**C=O), 2.70 (1H, ddd, *J* = 16.0, 7.4, 5.0 Hz, CH_2C$\mathbf{H_A}$$H_B$C=O), 2.39 (1H, app dt, *J* = 16.0, 9.1 Hz, CH_2C$H_A$$\mathbf{H_B}$C=O), 2.27 (1H, ddd, *J* = 18.1, 9.0, 4.4 Hz, C$\mathbf{H_A}$$H_B$$CH_2$C=O), 2.22-2.16 (1H, m, C$\mathbf{H_A}$$H_BCCH_3$), 2.02-1.89 (3H, m, C$H_A$$\mathbf{H_B}$$CH_2$C=O and C$\mathbf{H_A}$$H_BCH_A$$\mathbf{H_B}CCH_3$), 1.57-1.44 (1H, m, C$H_A$$\mathbf{H_B}$$CH_2CCH_3$), 1.15 (3H, s, C$\mathbf{H_3}$);.$^{13}C$ NMR (100.6 MHz, $CDCl_3$) δ 212.8 (C), 212.3 (C), 200.1 (C), 153.1 (C), 149.0 (CH), 136.9 (CH), 127.4 (CH), 121.8 (CH), 63.1 (C), 49.6 (CH), 41.6 (CH_2), 40.1 (CH), 39.0 (CH_2), 38.8 (CH_2), 23.7 (CH_2), 22.5 (CH_2), 16.8 (CH_3); HRMS (ESI) Exact mass calculated for $C_{17}H_{19}NO_3$ $[M+H]^+$: 286.1438, found: 286.1451; Enantiomeric excess was determined by HPLC with a Chiralpak AD-H column (90:10 *iso*-hexane:*i*-PrOH, 1.0 mL/min, 230 nm, 25 °C); t_r (major) = 13.8 min, t_r (minor) = 15.5 min; 46% ee.

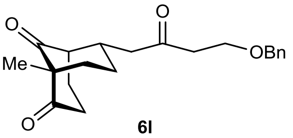

6l

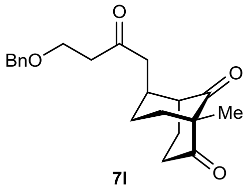

7l

(1*R*,5*R*,6*S*)-6-[4-(Benzyloxy)-2-oxobutyl]-1-methylbicyclo[3.3.1]nonane-2,9-dione (6l) and (1*S*,5*S*,6*S*)-6-[4-(benzyloxy)-2-oxobutyl]-1-methylbicyclo[3.3.1]nonane-2,9-dione (7l). The title compounds **6l** and **7l** were prepared according to the Representative Procedure from enone **5l** (75 mg, 0.22 mmol). Purification by column chromatography (20 to 30% EtOAc/cyclohexane) gave **6l** as a pale yellow oil (26 mg, 35%) and **7l** as a pale yellow oil (18 mg, 24%).

Data for **6l**: R_f = 0.20 (30% EtOAc/petroleum ether); $[\alpha]_D^{20}$ −24.3 (*c* 1.35, $CHCl_3$); IR 3011, 2935, 1704 (C=O), 1602, 1454, 1376, 1276, 1240, 1102, 909 cm^{-1}; ^{1}H NMR (400 MHz, $CDCl_3$) δ 7.39-7.25 (5H, m, Ar**H**), 4.49 (2H, s, PhC**H**$_2$O), 3.77-3.71 (2H, m, C**H**$_2$OBn), 2.81-2.74 (1H, m, C**H**C=O), 2.71-2.58 (3H, m, OCH$_2$C**H**$_2$C=O and C**H**CH$_2$C(O)CH$_2$), 2.57-2.46 (3H, m, CHC**H**$_2$C(O)CH$_2$ and C**H$_A$**H$_B$C(O)CCH$_3$), 2.34 (1H, dt, *J* = 16.4, 8.8 Hz, CH$_A$**H$_B$**C(O)CCH$_3$), 2.19-2.12 (1H, m, C**H$_A$**H$_B$CCH$_3$), 1.95-1.74 (2H, m, C**H**$_2$CHC=O), 1.72-1.59 (2H, m, CH$_A$**H$_B$**CCH$_3$, C**H$_A$**H$_B$CH$_2$CCH$_3$ overlapped with H_2O), 1.37-1.27 (1H, m, CH$_A$**H$_B$**CH$_2$CCH$_3$),1.14 (3H, s, C**H**$_3$); ^{13}C NMR (100.6 MHz, $CDCl_3$) δ 211.9 (C), 211.8 (C), 206.9 (C), 137.8 (C), 129.5 (CH), 128.4 (2 x CH), 127.8 (2 x CH), 73.3 (CH$_2$), 65.4 (CH$_2$), 62.7 (C), 48.3 (CH), 46.4 (CH$_2$), 43.2 (CH$_2$), 41.0 (CH$_2$), 39.6 (CH), 38.9 (CH$_2$), 25.5 (CH$_2$), 16.7 (CH$_2$), 16.5 (CH$_3$); HRMS (ESI) Exact mass calculated for $C_{21}H_{26}NaO_4$ $[M+H]^+$: 365.1723, found: 365.1723; Enantiomeric excess was determined by HPLC with a Chiralpak AD-H column (90:10 *iso*-hexane:*i*-PrOH, 1.5 mL/min, 210 nm, 25 °C); t_r (major) = 12.6 min, t_r (minor) = 14.8 min; 92% ee.

Data for **7l**: R_f = 0.28 (30% EtOAc/petroleum ether); $[\alpha]_D^{20}$ +29.0 (*c* 0.97, $CHCl_3$); IR 3011, 2935, 1702 (C=O), 1602, 1455, 1375, 1276, 1240, 1101, 1028 cm^{-1}; ^{1}H NMR (400 MHz, $CDCl_3$) δ 7.39-7.25 (5H, m, Ar**H**), 4.50 (2H, s, PhC**H**$_2$O), 3.80-3.68 (2H, m, C**H**$_2$OBn), 2.85-2.74 (1H, m, C**H**CH$_2$C(O)CH$_2$), 2.74-2.62 (4H, m, BnOCH$_2$C**H**$_2$C=O, C**H**C=O, and C**H$_A$**H$_B$C(O)CCH$_3$), 2.57 (1H, dd, *J* = 17.9, 7.0 Hz, CHC**H$_A$**H$_B$C(O)CH$_2$), 2.44 (1H, dd, *J* = 17.9, 7.3 Hz, CHCH$_A$**H$_B$**C(O)CH$_2$), 2.35 (1H, dt, *J* = 16.1, 9.2 Hz, CH$_A$**H$_B$**C(O)CCH$_3$), 2.17-2.08 (2H, m, C**H$_A$**H$_B$CHC=O and C**H$_A$**H$_B$CCH$_3$), 1.98-1.81 (2H, m, C**H$_A$**H$_B$CH$_2$CCH$_3$ and CH$_A$**H$_B$**CHC=O), 1.70 (1H, td, *J* = 13.7, 4.6 Hz, CH$_A$**H$_B$**CCH$_3$), 1.43-1.35 (1H, m, CH$_A$**H$_B$**CH$_2$CCH$_3$), 1.11 (3H, s, C**H**$_3$); ^{13}C NMR (100.6 MHz, $CDCl_3$) δ 213.0 (C), 212.0 (C), 207.3 (C), 137.9 (C), 129.6 (CH), 128.4 (2 x CH), 127.7 (2 x CH), 73.3 (CH$_2$), 65.3 (CH$_2$), 63.0 (C), 49.4 (CH), 45.5 (CH$_2$), 43.6 (CH$_2$), 41.1 (CH), 39.0 (CH$_2$), 38.8 (CH$_2$), 23.3 (CH$_2$), 22.3 (CH$_2$), 16.7 (CH$_3$); HRMS (ESI) Exact mass calculated for $C_{21}H_{26}NaO_4$ $[M+H]^+$: 365.1723, found: 365.1722; Enantiomeric excess was determined by HPLC with a Chiralpak AD-H column (90:10 *iso*-hexane:*i*-PrOH, 1.5 mL/min, 210 nm, 25 °C); t_r (major) = 8.4 min, t_r (minor) = 9.7 min; 80% ee.

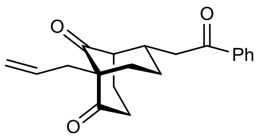

6m

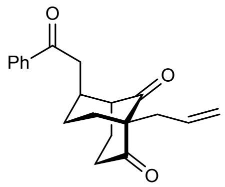

7m

(1*S*,5*R*,6*S*)-1-Allyl-6-(2-oxo-2-phenylethyl)bicyclo[3.3.1]nonane-2,9-dione (6m) and (1*R*,5*S*,6*S*)-1-allyl-6-(2-oxo-2-phenylethyl)bicyclo[3.3.1]nonane-2,9-dione (7m). The title compounds **6m** and **7m** were prepared according to the Representative Procedure from enone **5m** (62 mg, 0.20 mmol). Purification by column chromatography (20 to 25% EtOAc/*iso*-hexane) gave **6m** as an off-white solid (55 mg, 89%) and **7m** as a colorless film (4 mg, 6%).

Data for **6m**: R_f = 0.42 (30% EtOAc/petroleum ether); m.p. 69-70 °C (*iso*-hexane/CH_2Cl_2); $[\alpha]_D^{20}$ −27.2 (*c* 1.60, $CHCl_3$); IR 3083, 3022, 2948, 1736 (C=O), 1703 (C=O), 1626, 1599, 1449, 1002, 925 cm^{-1}; ^{1}H NMR (500 MHz, $CDCl_3$) δ 7.96-7.90 (2H, m, Ar**H**), 7.62-7.56 (1H, m, Ar**H**), 7.51-7.45 (2H, m, Ar**H**), 5.80 (1H, dddd, *J* = 16.8, 10.8, 8.2, 6.4 Hz, C**H**=CH_2), 5.08-4.97 (2H, m, CH=C**H**$_2$), 3.03 (1H, dd, *J* = 16.3, 7.5 Hz, C**H**$_A$H$_B$C(O)Ar), 2.97 (1H, dd, *J* = 16.3, 6.2 Hz, CH$_A$**H**$_B$C(O)Ar), 2.90 (1H, app dt, *J* = 9.7, 3.0 Hz, C**H**CH_2C(O)Ar), 2.79-2.70 (1H, m, C**H**C=O), 2.58 (1H, ddd, *J* = 15.1, 6.5, 3.4 Hz, C**H**$_A$H$_B$C(O)CCH_2CH=), 2.47 (1H, dd, *J* = 13.5, 6.4 Hz, C**H**$_A$H$_B$CH=), 2.34 (1H, dd, *J* = 13.5, 8.2 Hz, CH$_A$**H**$_B$CH=), 2.26-2.17 (2H, m, CH$_A$**H**$_B$C(O)CCH_2CH= and C**H**$_A$H$_B$CCH_2CH=), 2.11-2.00 (1H, m, C**H**$_A$CH$_B$$CH_2$C=O), 1.86 (1H, dddd, *J* = 14.2, 11.6, 6.5, 2.7 Hz, CH$_A$C**H**$_B$$CH_2$C=O), 1.75-1.63 (2H, m, C**H**$_A$H$_B$CH$_A$**H**$_B$CCH_2CH=), 1.47-1.32 (1H, m, CH$_A$**H**$_B$$CH_2CCH_2$CH=); ^{13}C NMR (125.8 MHz, $CDCl_3$) δ 211.6 (C), 211.5 (C), 197.8 (C), 136.6 (C), 133.4 (CH), 133.1 (CH), 128.8 (2 x CH), 128.0 (2 x CH), 118.6 (CH_2), 65.8 (C), 48.6 (CH), 41.3 (CH_2), 41.2 (CH), 40.6 (CH_2), 39.8 (CH_2), 36.3 (CH_2), 25.0 (CH_2), 16.2 (CH_2); HRMS (ESI) Exact mass calculated for $C_{20}H_{23}O_3$ $[M+H]^+$: 311.1642, found: 311.1641; Enantiomeric excess was determined by HPLC with a Chiralpak IA-3 column (60:40 *iso*-hexane:*i*-PrOH, 1.5 mL/min, 254 nm, 25 °C); t_r (major) = 4.4 min, t_r (minor) = 5.4 min; 86% ee.

Data for **7m**: R_f = 0.53 (30% EtOAc/petroleum ether); $[\alpha]_D^{20}$ +26.4 (*c* 0.33, $CHCl_3$); IR 3043, 2935, 1733 (C=O), 1701 (C=O), 1600, 1449, 1279, 1247, 1004, 926 cm^{-1}; ^{1}H NMR (500 MHz, $CDCl_3$) δ 7.97-7.92 (2H, m, Ar**H**), 7.62-7.56 (1H, m, Ar**H**), 7.52-7.45 (2H, m, Ar**H**), 5.81 (1H, dddd, *J* = 16.7, 10.1, 8.2, 6.5 Hz, C**H**=CH_2), 5.10-4.98 (2H, m, CH=C**H**$_2$), 3.10 (1H, dd, *J* = 16.1, 5.5 Hz, C**H**$_A$H$_B$C(O)Ar), 2.98-2.85 (2H, m, C**H**CH$_A$**H**$_B$C(O)Ar), 2.78-2.73 (1H, m, C**H**C=O), 2.68 (1H, ddd, *J* = 15.2, 6.6, 3.1 Hz, C**H**$_A$H$_B$C(O)CCH_2CH=), 2.49 (1H, dd, *J* = 13.5, 6.4 Hz, C**H**$_A$H$_B$CH=), 2.40-2.28 (2H, m, CH$_A$**H**$_B$CH= and CH$_A$**H**$_B$C(O)CCH_2CH=), 2.25-2.13 (2H, m, C**H**$_A$H$_B$CCH_2CH= and C**H**$_A$CH$_B$$CH_2$C=O), 2.11-2.00 (1H, m, CH$_A$C**H**$_B$$CH_2$C=O), 1.90-1.79 (2H, m, C**H**$_A$H$_B$CH$_A$**H**$_B$CCH_2CH=), 1.54-1.46 (1H, m, CH$_A$**H**$_B$$CH_2CCH_2$CH=); ^{13}C NMR (125.8 MHz, $CDCl_3$) δ 212.9 (C), 211.5 (C), 198.1 (C), 136.8 (C), 133.4 (CH), 133.0 (CH), 128.7 (2 x CH),

128.0 (2 x CH), 118.7 (CH_2), 66.3 (C), 49.9 (CH), 42.1 (CH), 40.8 (CH_2), 40.2 (CH_2), 37.8 (CH_2), 36.5 (CH_2), 23.0 (CH_2), 22.2 (CH_2); HRMS (ESI) Exact mass calculated for $C_{20}H_{22}NaO_3$ $[M+Na]^+$: 333.1461, found: 333.1452; Enantiomeric excess was determined by HPLC with a Chiralpak IA-3 column (90:10 *iso*-hexane:*i*-PrOH, 1.5 mL/min, 254 nm, 25 °C); t_r (major) = 6.3 min, t_r (minor) = 7.6 min; 94% ee.

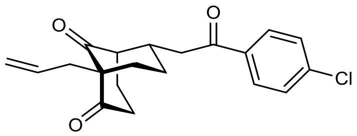

(1*S*,5*R*,6*S*)-1-Allyl-6-[2-(4-chlorophenyl)-2-oxoethyl]bicyclo[3.3.1]nonane-2,9-dione (6n). The title compound was prepared according to the Representative Procedure from enone **5n** (69 mg, 0.20 mmol). Purification by column chromatography (15 to 20% EtOAc/petroleum ether) gave a white solid (65 mg, 94%) as a >95:5 ratio of diastereomers. R_f = 0.42 (30% EtOAc/petroleum ether); m.p. 100-102 °C (CH_2Cl_2); $[\alpha]_D^{20}$ −22.5 (*c* 1.09, $CHCl_3$); IR 2929, 1736 (C=O), 1703 (C=O), 1639, 1590, 1449, 1349, 1280, 1094, 985 cm^{-1}; ^{1}H NMR (400 MHz, $CDCl_3$) δ 7.86 (2H, d, *J* = 8.7 Hz, Ar**H**), 7.45 (2H, d, *J* = 8.7 Hz, Ar**H**), 5.79 (1H, dddd, *J* = 16.7, 10.1, 8.2, 6.4 Hz, C**H**=CH_2), 5.08-4.96 (2H, m, CH=C**H$_2$**), 2.99 (1H, dd, *J* = 16.4, 7.5 Hz, C**H$_A$**H_BC(O)Ar), 2.93 (1H, dd, *J* = 16.4, 6.2 Hz, CH_A**H$_B$**C(O)Ar), 2.88 (1H, app dt, *J* = 9.9, 2.9 Hz C**H**C=O), 2.77-2.66 (1H, m, C**H**CH_2C(O)Ar), 2.57 (1H, ddd, *J* = 15.0, 6.5, 3.3 Hz, C**H$_A$**H_BC(O)CCH_2CH=), 2.46 (1H, dd, *J* = 13.5, 6.4 Hz, C**H$_A$**H_BCH=), 2.32 (1H, dd, *J* = 13.5, 8.2 Hz, CH_A**H$_B$**CH=), 2.24-2.13 (2H, m, CH_A**H$_B$**C(O)CCH_2CH= and C**H$_A$**H_BCCH_2CH=), 2.10-1.99 (1H, m, C**H$_A$**$CH_B$$CH_2$C=O), 1.89-1.78 (1H, m, CH_AC**H$_B$**CH_2C=O), 1.73-1.62 (2H, m, C**H$_A$**$H_B$$CH_A$**H$_B$**C$CH_2$CH=), 1.46-1.30 (1H, m, CH_A**H$_B$**CH_2CCH_2CH=); ^{13}C NMR (100.6 MHz, $CDCl_3$) δ 211.5 (C), 211.4 (C), 196.4 (C), 139.9 (C), 134.9 (C), 133.1 (CH), 129.4 (2 x CH), 129.1 (2 x CH), 118.6 (CH_2), 65.7 (C), 48.5 (CH), 41.2 (CH_2), 41.0 (CH), 40.6 (CH_2), 39.7 (CH_2), 36.3 (CH_2), 25.0 (CH_2), 16.1 (CH_2); HRMS (ESI) Exact mass calculated for $C_{20}H_{21}ClNaO_3$ $[M+Na]^+$: 367.1071, found: 367.1052; Enantiomeric excess was determined by HPLC with a Chiralpak IC column (90:10 *iso*-hexane:EtOH, 1.0 mL/min, 210 nm, 25 °C); t_r (major) = 13.4 min, t_r (minor) = 14.4 min; 88% ee.

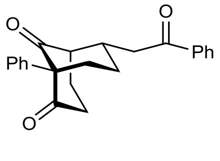

(1*S*,5*R*,6*S*)-6-(2-Oxo-2-phenylethyl)-1-phenylbicyclo[3.3.1]nonane-2,9-dione (6o). The title compound was prepared according to the Representative Procedure from enone **5o** (69 mg, 0.20 mmol). Purification by column chromatography (20 to 30% EtOAc/petroleum ether) gave a brown solid (47 mg, 68%) as a >95:5 ratio of diastereomers. R_f = 0.17 (20% EtOAc/petroleum ether); m.p. 163-166 °C (CH_2Cl_2); $[\alpha]_D^{20}$ +13.2 (*c* 0.44, $CHCl_3$); IR 2954, 2929, 1736 (C=O), 1706 (C=O), 1688 (C=O), 1599, 1500, 1449, 1268, 1092, 985 cm^{-1}; ^{1}H NMR (400 MHz, $CDCl_3$) δ 7.99-7.94 (2H, m, Ar**H**), 7.65-7.57 (1H, m,

Ar**H**), 7.54-7.46 (2H, m, Ar**H**), 7.39-7.28 (3H, m, Ar**H**), 7.14-7.08 (2H, m, Ar**H**), 3.15-2.98 (3H, m, C**H**$_2$C(O)Ph and C**H**C=O), 2.94-2.74 (3H, m, C**H**CH$_2$C(O)Ph, CH$_2$C**H$_A$**H$_B$C=O, and C**H$_A$**H$_B$CPh), 2.61 (1H, ddd, *J* = 14.5, 12.2, 8.9 Hz, CH$_2$CH$_A$**H$_B$**C=O), 2.32-2.17 (2H, m, CH$_A$**H$_B$**CPh and C**H$_A$**CH$_B$CH$_2$C=O), 2.04-1.85 (2H, m, CH$_A$C**H$_B$**CH$_2$C=O and C**H$_A$**H$_B$CH$_2$CPh), 1.64-1.46 (1H, m, CH$_A$**H$_B$**CH$_2$CPh); ^{13}C NMR (100.6 MHz, CDCl$_3$) δ 210.6 (C), 210.2 (C), 197.6 (C), 136.6 (C), 136.2 (C), 133.5 (CH), 128.8 (2 x CH), 128.4 (2 x CH), 128.1 (2 x CH), 127.7 (CH), 127.6 (2 x CH), 70.0 (C), 48.6 (CH), 41.2 (CH$_2$), 41.1 (CH$_2$ and CH), 37.8 (CH$_2$), 24.8 (CH$_2$), 16.2 (CH$_2$); HRMS (ESI) Exact mass calculated for $C_{23}H_{22}NaO_3$ [M+Na]$^+$: 369.1461, found: 369.1454; Enantiomeric excess was determined by HPLC with a Chiralpak AD-H column (80:20 *iso*-hexane:*i*-PrOH, 1.5 mL/min, 210 nm, 25 °C); t_r (major) = 13.1 min, t_r (minor) = 19.3 min; 94% ee.

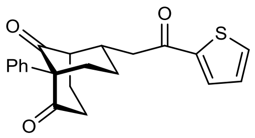

(1*S*,5*R*,6*S*)-6-[2-Oxo-2-(thiophen-2-yl)ethyl]-1-phenylbicyclo[3.3.1]nonane-2,9-dione (6p). The title compound was prepared according to a modification of the Representative Procedure from enone **5p** (70 mg, 0.20 mmol) in toluene (2 mL). Purification by column chromatography (40% EtOAc/petroleum ether) gave a yellow solid (35 mg, 50%) as a >95:5 ratio of diastereomers. R_f = 0.18 (40% EtOAc/petroleum ether); m.p. 145-149 °C (CH$_2$Cl$_2$); $[\alpha]_D^{20}$ +29.9 (*c* 0.91, CHCl$_3$); IR 2934, 1736 (C=O), 1707 (C=O), 1661 (C=O), 1518, 1416, 1357, 1266, 1090, 840 cm^{-1}; ^{1}H NMR (400 MHz, CDCl$_3$) δ 7.73 (1H, dd, *J* = 3.8, 1.1 Hz, Ar**H**), 7.68 (1H, dd, *J* = 5.0, 1.1 Hz, Ar**H**), 7.38-7.27 (3H, m, Ar**H**), 7.16 (1H, dd, *J* = 5.0, 3.8 Hz, Ar**H**), 7.13-7.09 (2H, m, Ar**H**), 3.08 (1H, app dt, *J* = 10.3, 2.7 Hz, C**H**C=O), 3.01 (1H, dd, *J* = 15.5, 7.7 Hz, C**H$_A$**H$_B$C(O)Ar), 2.95 (1H, dd, *J* = 15.5, 6.3 Hz, CH$_A$**H$_B$**C(O)Ar), 2.89-2.74 (3H, m, C**H**CH$_2$C(O)Ar, CH$_2$C**H$_A$**H$_B$C=O, and C**H$_A$**H$_B$CPh), 2.61 (1H, ddd, *J* = 14.5, 12.3, 9.0 Hz, CH$_2$CH$_A$**H$_B$**C=O), 2.30-2.17 (2H, m, CH$_A$**H$_B$**CPh and C**H$_A$**CH$_B$CH$_2$C=O), 2.02-1.84 (2H, m, CH$_A$C**H$_B$**CH$_2$C=O and C**H$_A$**H$_B$CH$_2$CPh), 1.61-1.48 (1H, m, CH$_A$**H$_B$**CH$_2$CPh); ^{13}C NMR (100.6 MHz, CDCl$_3$) δ 210.5 (C), 210.0 (C), 190.5 (C), 144.0 (C), 136.1 (C), 134.3 (CH), 132.1 (CH), 128.4 (2 x CH), 128.3 (CH), 127.7 (CH), 127.6 (2 x CH), 69.9 (C), 48.5 (CH), 42.0 (CH$_2$), 41.5 (CH), 41.0 (CH$_2$), 37.6 (CH$_2$), 24.7 (CH$_2$), 16.1 (CH$_2$); HRMS (ESI) Exact mass calculated for $C_{21}H_{20}NaO_3$ [M+Na]$^+$: 375.1025, found: 375.1011; Enantiomeric excess was determined by HPLC with a Chiralpak AD-H column (75:25 *iso*-hexane:*i*-PrOH, 1.5 mL/min, 210 nm, 25 °C); t_r (major) = 12.2 min, t_r (minor) = 18.2 min; 97% ee.

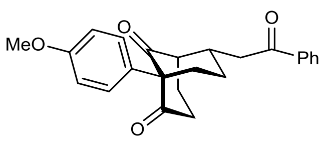

(1*S*,5*R*,6*S*)-1-(4-Methoxyphenyl)-6-(2-oxo-2-phenylethyl)bicyclo[3.3.1]nonane-2,9-dione (6q). The title compound was prepared according to the Representative Procedure from enone **5q** (75 mg, 0.20 mmol). Purification by column chromatography (20 to 40% EtOAc/petroleum ether)

gave a white solid (47 mg, 63%) as a >95:5 ratio of diastereomers. R_f = 0.17 (40% EtOAc/petroleum ether); m.p. 179-180 °C (CH_2Cl_2); $[\alpha]_D^{20}$ +14.6 (*c* 0.94, $CHCl_3$); IR 1735 (C=O), 1705 (C=O), 1685 (C=O), 1599, 1516, 1424, 1239, 929 cm^{-1}; ^{1}H NMR (400 MHz, $CDCl_3$) δ 7.99-7.93 (2H, m, Ar**H**), 7.64-7.57 (1H, m, Ar**H**), 7.53-7.47 (2H, m, Ar**H**), 7.07-7.01 (2H, m, Ar**H**), 6.92-6.87 (2H, m, Ar**H**), 3.80 (3H, s, C**H**$_3$), 3.11-3.06 (1H, m, C**H**C=O), 3.09 (1H, dd, *J* = 16.3, 7.6 Hz, C**H**$_A$H$_B$C(O)Ph), 3.02 (1H, dd, *J* = 16.3, 6.0 Hz, CH$_A$**H**$_B$C(O)Ph), 2.91-2.86 (1H, m, C**H**CH$_2$C(O)Ph), 2.81-2.73 (2H, m, CH$_2$C**H**$_A$H$_B$C=O and C**H**$_A$H$_B$CAr), 2.59 (1H, ddd, *J* = 14.5, 12.2, 8.9 Hz, CH$_2$CH$_A$**H**$_B$C=O), 2.28-2.15 (2H, m, CH$_A$**H**$_B$CAr, C**H**$_A$CH$_B$CH$_2$C=O), 2.02-1.85 (2H, m, CH$_A$C**H**$_B$CH$_2$C=O and C**H**$_A$H$_B$CH$_2$CAr), 1.59-1.46 (1H, m, CH$_A$**H**$_B$CH$_2$CAr); ^{13}C NMR (100.6 MHz, $CDCl_3$) δ 210.8 (C), 210.5 (C), 197.6 (C), 158.9 (C), 136.6 (C), 133.5 (CH), 128.8 (2 x CH), 128.7 (2 x CH), 128.2 (C), 128.1 (2 x CH), 113.9 (2 x CH), 69.4 (C), 55.2 (CH_3), 48.7 (CH), 41.2 (CH_2), 41.1 (CH), 41.0 (CH_2), 38.0 (CH_2), 24.8 (CH_2), 16.2 (CH_2); HRMS (ESI) Exact mass calculated for $C_{24}H_{24}NaO_4$ $[M+Na]^+$: 399.1567, found: 399.1565. Enantiomeric excess was determined by HPLC with a Chiralpak AD-H column (75:25 *iso*-hexane:*i*-PrOH, 1.5 mL/min, 210 nm, 25 °C); t_r (major) = 18.8 min, t_r (minor) = 32.6 min; 94% ee.

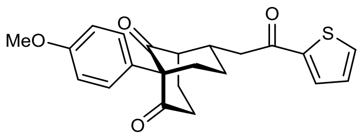

(1*S*,5*R*,6*S*)-1-(4-Methoxyphenyl)-6-[2-oxo-2-(thiophen-2-yl)ethyl]bicyclo[3.3.1]nonane-2,9-dione (6r). The title compound was prepared according to the Representative Procedure from enone **5r** (77 mg, 0.20 mmol). Purification by column chromatography (30% EtOAc/petroleum ether) gave a pale yellow solid (38 mg, 49%) as a >95:5 ratio of diastereomers. R_f = 0.15 (40% EtOAc/petroleum ether); m.p. 164-167 °C (CH_2Cl_2); $[\alpha]_D^{20}$ +14.2 (*c* 0.81, $CHCl_3$); IR 2939, 2839, 1730 (C=O), 1705 (C=O), 1661 (C=O), 1515, 1465, 1416, 1254, 1184 cm^{-1}; ^{1}H NMR (400 MHz, $CDCl_3$) δ 7.73 (1H, dd, *J* = 3.8, 1.1 Hz, Ar**H**), 7.68 (1H, dd, *J* = 5.0, 1.1 Hz, Ar**H**), 7.16 (1H, dd, *J* = 5.0, 3.8 Hz, Ar**H**), 7.05-7.01 (2H, m, Ar**H**), 6.91-6.86 (2H, m, Ar**H**), 3.79 (3H, s, C**H**$_3$), 3.08 (1H, app dt, *J* = 10.2, 2.6 Hz, C**H**C=O), 3.01 (1H, dd, *J* = 15.5, 7.7 Hz, C**H**$_A$H$_B$C(O)Ar), 2.94 (1H, dd, *J* = 15.5, 6.3 Hz, CH$_A$**H**$_B$C(O)Ar), 2.89-2.82 (1H, m, C**H**CH$_2$C(O)Ar), 2.81-2.73 (2H, m, CH$_2$C**H**$_A$H$_B$C=O and C**H**$_A$H$_B$CAr), 2.58 (1H, ddd, *J* = 14.5, 12.3, 8.9 Hz, CH$_2$CH$_A$**H**$_B$C=O), 2.28-2.14 (2H, m, CH$_A$**H**$_B$CAr and C**H**$_A$CH$_B$CH$_2$C=O), 2.01-1.84 (2H, m, CH$_A$C**H**$_B$CH$_2$C=O and C**H**$_A$H$_B$CH$_2$CAr), 1.60-1.47 (1H, m, CH$_A$**H**$_B$CH$_2$CAr); ^{13}C NMR (100.6 MHz, $CDCl_3$) δ 210.7 (C), 210.4 (C), 190.5 (C), 158.9 (C), 144.0 (C), 134.3 (CH), 132.1 (CH), 128.7 (2 x CH), 128.3 (CH), 128.1 (C), 113.9 (2 x CH), 69.3 (C), 55.2 (CH_3), 48.5 (CH), 42.1 (CH_2), 41.5 (CH), 41.0 (CH_2), 37.8 (CH_2), 24.9 (CH_2), 16.2 (CH_2); HRMS (ESI) Exact mass calculated for $C_{24}H_{24}NaO_4$ $[M+Na]^+$: 405.1131, found: 405.1119; Enantiomeric excess was determined by HPLC with a Chiralpak AD-H

column (75:25 *iso*-hexane:*i*-PrOH, 1.5 mL/min, 254 nm, 25 °C); t_r (major) = 21.9 min, t_r (minor) = 39.0 min; 92% ee.

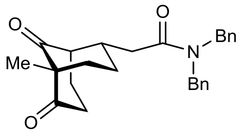

***N,N*-Dibenzyl-2-[(1*R*,2*S*,5*R*)-5-methyl-6,9-dioxobicyclo[3.3.1]nonan-2-yl]acetamide (9)**. The title compound was prepared according to a modification of the Representative Procedure from α,β-unsaturated amide **8** (81 mg, 0.20 mmol) and phosphoric acid **4b** (15 mg, 0.02 mmol) in toluene (2 mL) and by heating at 80 °C for 72 h. Purification by column chromatography (20 to 40% EtOAc/petroleum ether) gave a colorless film (42 mg, 52%) as a >95:5 ratio of diastereomers [along with recovered starting material (32 mg, 40%)]. Rf = 0.33 (40% EtOAc/petroleum ether); $[\alpha]_D^{20}$ −15.1 (*c* 2.10, $CHCl_3$); IR 3066, 2936, 1731 (C=O), 1703 (C=O), 1644 (C=O), 1496, 1467, 1453, 1361, 1240, 1079 cm^{-1}; ^1H NMR (400 MHz, $CDCl_3$) δ 7.43-7.27 (6H, m, Ar**H**), 7.25-7.18 (2H, m, Ar**H**), 7.13 (2H, d, *J* = 7.1 Hz, Ar**H**), 4.66 (1H, d, *J* = 14.7 Hz, C**H**$_A$H$_B$Ph), 4.60 (1H, d, *J* = 14.7 Hz, CH$_A$**H**$_B$Ph), 4.48 (1H, d, *J* = 17.9 Hz, C**H**$_A$'H$_B$'Ph), 4.43 (1H, d, *J* = 17.9 Hz, CH$_A$'**H**$_B$'Ph), 2.88 (1H, dt, *J* = 8.6, 2.8 Hz, C**H**C=O), 2.77-2.67 (1H, m, C**H**CH_2C(O)NBn_2), 2.54-2.38 (3H, m, C**H**$_2$C(O)CCH_3 and C**H**$_A$H$_B$C(O)NBn_2), 2.32 (1H, dt, *J* = 16.2, 8.9 Hz, CH$_A$**H**$_B$C(O)NBn_2), 2.20 (1H, ddd, *J* = 13.3, 4.7, 1.6 Hz, C**H**$_A$H$_B$CCH_3), 1.88 (1H, dtd, *J* = 14.4, 8.8, 5.7 Hz, C**H**$_A$CH$_B$$CH_2$C=O), 1.81-1.69 (2H, m, CH$_A$C**H**$_B$$CH_2$C=O and C**H**$_AH_B$$CH_2CCH_3$), 1.65 (1H, dd, *J* = 13.4, 4.8 Hz, CH$_A$**H**$_B$CCH_3), 1.41-1.31 (1H, m, CH$_A$**H**$_B$$CH_2CCH_3$), 1.15 (3H, s, C**H**$_3$); ^{13}C NMR (100.6 MHz, $CDCl_3$) δ 212.0 (C), 211.7 (C), 170.9 (C), 137.1 (C), 136.3 (C), 129.1 (2 x CH), 128.7 (2 x CH), 128.3 (2 x CH), 127.8 (CH), 127.6 (CH), 126.1 (2 x CH), 62.6 (C), 50.1 (CH_2), 48.7 (CH_2), 48.4 (CH), 41.2 (CH), 41.0 (CH_2), 38.9 (CH_2), 36.3 (CH_2), 25.6 (CH_2), 16.7 (CH_2), 16.6 (CH_3); HRMS (ESI) Exact mass calculated for $C_{26}H_{30}NO_3$ $[M+H]^+$: 404.2220, found: 404.2217; Enantiomeric excess was determined by HPLC with a Chiralpak IA-3 column (90:10 *iso*-hexane:*i*-PrOH, 1.5 mL/min, 210 nm, 25 °C); t_r (major) = 24.1 min, t_r (minor) = 27.6 min; 77% ee.

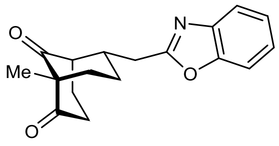

(1*R*,5*R*,6*S*)-6-[2-(Benzo[*d*]oxazol-2-yl)-2-oxoethyl]-1-methylbicyclo[3.3.1]nonane-2,9-dione (11). The title compound was prepared according to the Representative Procedure from alkenylbenzoxazole **10** (59 mg, 0.20 mmol). Purification by column chromatography (40% EtOAc/petroleum ether) gave a yellow solid (52 mg, 88%) as a >95:5 ratio of diastereomers. R_f = 0.24 (40% EtOAc/petroleum ether); m.p. 129-133 °C (CH_2Cl_2); $[\alpha]_D^{20}$ −18.9 (*c* 1.10, $CHCl_3$); IR 2937, 1732 (C=O), 1703 (C=O), 1614, 1572, 1455, 1344, 1242, 1106, 1003 cm^{-1}; ^1H NMR (400 MHz, $CDCl_3$) δ 7.69-7.64 (1H, m, Ar**H**), 7.51-7.45 (1H, m, Ar**H**), 7.35-7.28 (2H, m, Ar**H**), 3.05

(1H, dd, *J* = 12.1, 4.7 Hz, C**H_A**H_BC=N), 3.00 (1H, dd, *J* = 12.1, 4.6 Hz, CH_A**H_B**C=N), 2.84 (1H, app dt, *J* = 7.6, 3.9 Hz, C**H**C=O), 2.81-2.70 (1H, m, C**H**CH_2C=N), 2.62 (1H, app dt, *J* = 16.3, 6.5 Hz, C**H_A**H_BC=O), 2.40 (1H, app dt, *J* = 16.3, 6.5 Hz, CH_A**H_B**C=O), 2.27-2.21 (1H, m, C**H_A**H_BCCH_3), 2.08-2.00 (2H, m, C**H_2**CH_2C=O), 1.78-1.53 (3H, m, C**H_2**CH_A**H_B**CCH_3), 1.15 (3H, s, C**H_3**); ^{13}C NMR (100.6 MHz, $CDCl_3$) δ 211.6 (C), 211.5 (C), 164.1 (C), 150.8 (C), 141.1 (C), 124.9 (CH), 124.4 (CH), 119.7 (CH), 110.4 (CH), 62.6 (C), 47.9 (CH), 42.8 (CH), 40.7 (CH_2), 38.9 (CH_2), 32.0 (CH_2), 25.6 (CH_2), 16.5 (CH_3), 16.3 (CH_2); HRMS (ESI) Exact mass calculated for $C_{18}H_{20}NO_3$ $[M+H]^+$: 298.1438, found: 298.1422; Enantiomeric excess was determined by HPLC with a Chiralpak AD-H column (95:5 *iso*-hexane:*i*-PrOH, 0.8 mL/min, 254 nm, 25 °C); t_r (minor) = 29.1 min, t_r (major) = 31.4 min; 62% ee.

Tests for the Self-Disproportionation of Enantiomers (SDE) Phenomenon

Recently, the phenomenon termed "Self-Disproportionation of Enantiomers" (SDE) has been described, which provides the possibility for significant changes to the enantiomeric composition of samples of compounds to occur during achiral column chromatography.[18] To check whether the SDE phenomenon was observed for the series of compounds described herein, two representative bicyclic products were passed through a normal silica gel column and the enantiomeric excesses of various fractions were measured, as described below. From these results, it was concluded that a small but measurable SDE was observed for these compounds. However, the effect of the SDE on the quoted enantiomeric excesses of the products is likely to be minimal, given the small magnitude of the SDE and the straightforward purification of the products, meaning that the significant majority of material was collected during column chromatography.

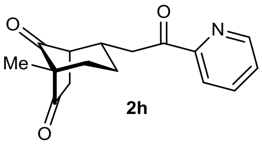

2h

(1*R*,2*S*,5*R*)-5-Methyl-2-[2-oxo-2-(pyridin-2-yl)ethyl]bicyclo[3.2.1]octane-6,8-dione (2h). Previously purified bicycle **2h** (87% ee) was passed through a silica gel column (40% EtOAc/petroleum ether) and collected over 12 fractions. The enantiomeric excesses for fractions 1, 7, and 12 were determined by HPLC with a Chiralpak IA-3 column (70:30 *iso*-hexane:*i*-PrOH, 1.5 mL/min, 230 nm, 25 °C).

Fraction 1: t_r (major) = 8.0 min, t_r (minor) = 13.8 min; 90% ee.

Fraction 7: t_r (major) = 8.3 min, t_r (minor) = 14.4 min; 84% ee.

Fraction 12: t_r (major) = 8.5 min, t_r (minor) = 15.0 min; 86% ee.

These results show that **2h** exhibits a small but observable SDE (the original sample was 87% ee).

Racemate:

data acquired by: AM
on: 5/19/2014
location: Vial 82

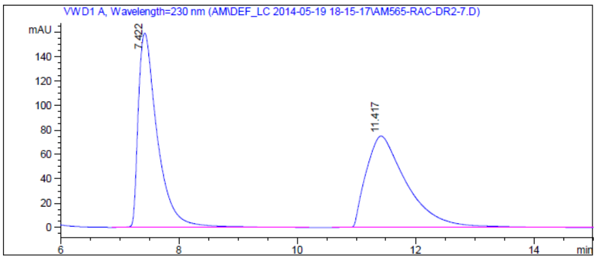

Meas. R	Area %	Width	Symmetr.
7.422	49.869	0.334	0.456
11.417	50.131	0.739	0.525

Original sample (87% ee):

data acquired by: AM
on: 5/19/2014
location: Vial 83

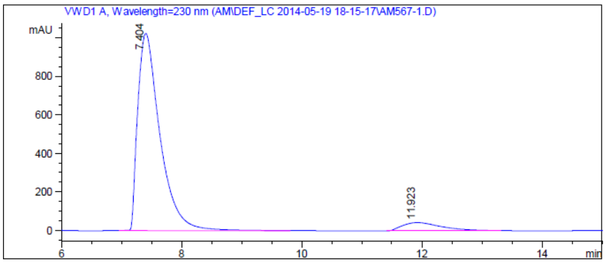

Meas. R	Area %	Width	Symmetr.
7.404	93.350	0.430	0.532
11.923	6.650	0.737	0.585

18. (a) V. A. Soloshonok, *Angew. Chem., Int. Ed.* **2006**, *45*, 766-769. (b) V. A. Soloshonok, C. Roussel, O. Kitagawa, A. E. Sorochinsky, *Chem. Soc. Rev.* **2012**, *41*, 4180-4188. (c) Y. Suzuki, J. Han, O. Kitagawa, J. L. Acena, K. D. Klika, V. A. Soloshonok, *RSC Adv.* **2015**, *5*, 2988-2993.

Fraction 1 (90% ee)

data acquired by: HWL
on: 12/15/2014
location: Vial 85

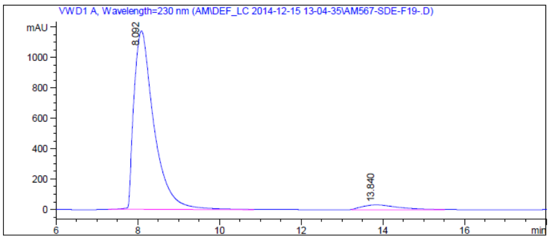

Meas. R	Area %	Width	Symmetr.
8.092	94.940	0.569	0.554
13.840	5.060	1.069	0.573

Fraction 7 (84% ee):

data acquired by: HWL
on: 12/15/2014
location: Vial 86

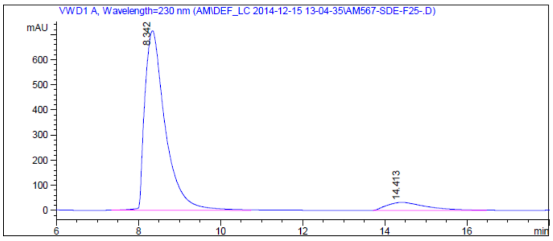

Meas. R	Area %	Width	Symmetr.
8.342	92.071	0.573	0.566
14.413	7.929	1.097	0.577

Fraction 12 (86% ee):

data acquired by: HWL
on: 12/15/2014
location: Vial 87

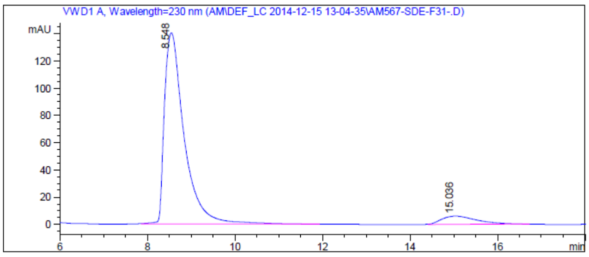

Meas. R	Area %	Width	Symmetr.
8.548	92.961	0.520	0.518
15.036	7.039	0.913	0.642

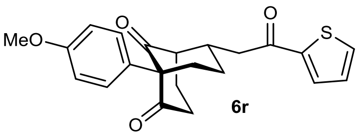

(1*S*,5*R*,6*S*)-1-(4-Methoxyphenyl)-6-[2-oxo-2-(thiophen-2-yl)ethyl]bicyclo[3.3.1]nonane-2,9-dione (6r). Previously purified bicycle **6r** (92% ee) was passed through a silica gel column (40% EtOAc/petroleum ether) and collected over 9 fractions. The enantiomeric excesses for fractions 1, 5, and 9 were determined by HPLC with a Chiralpak AD-H column (75:25 *iso*-hexane:*i*-PrOH, 1.5 mL/min, 254 nm, 25 °C).

Fraction 1: t_r (major) = 22.9 min, t_r (minor) = 41.8 min; 92% ee.

Fraction 5: t_r (major) = 22.7 min, t_r (minor) = 41.4 min; 90% ee.

Fraction 9: t_r (major) = 22.5 min, t_r (minor) = 40.9 min; 89% ee.

These results show that **6r** may exhibit a small but observable SDE (the original sample was 92% ee), but the contribution of instrumental error to the deviation of ±3% ee cannot be excluded.

Racemate:

Data file: C:\CHEM32\1\DATA\AMAEL\DEF_LC 2014-07-11 16-55-38\AM633-RAC-3.D
Sample name: AM633-rac-3
Instrument: AGILENT 1260 **Acq. operator:** SYSTEM
Injection date: 7/11/2014 5:45:46 PM
Acq. method: ADH75B25A.45MIN.1.5 ML.80UL.M

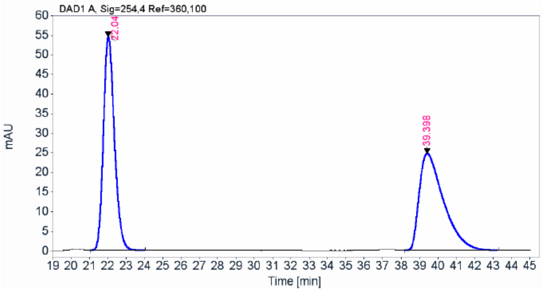

Signal: DAD1 A, Sig=254,4 Ref=360,100

RT [min]	Type	Width [min]	Area	Height	Area%
22.040	BB	0.6455	2270.964	54.3501	50.09
39.398	BB	1.3229	2263.209	24.6101	49.91

Original sample (92% ee):

Data file: C:\CHEM32\1\DATA\AMAEL\DEF_LC 2014-07-16 21-07-04\AM641-EE-1.D
Sample name: AM641-ee-1
Instrument: AGILENT 1260 **Acq. operator:** SYSTEM
Injection date: 7/16/2014 9:14:35 PM
Acq. method: ADH75B25A.45MIN.1.5 ML.80UL.M

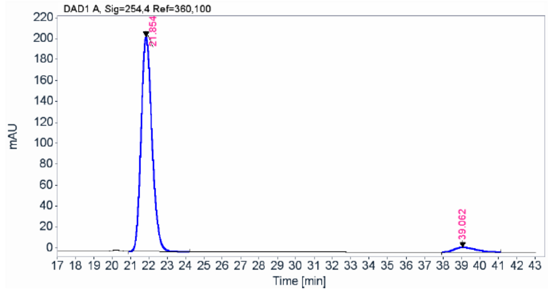

Signal: DAD1 A, Sig=254,4 Ref=360,100

RT [min]	Type	Width [min]	Area	Height	Area%
21.854	BB	0.6371	8413.978	204.9108	95.82
39.062	MM	1.3600	367.122	4.4991	4.18

Fraction 1 (92% ee):

Data file: C:\CHEM32\1\DATA\ARB2014\DEF_LC 2014-12-18 10-18-18\ARB-AM641-FR26B.D
Sample name: arb-am641-fr26b
Instrument: AGILENT 1260 **Acq. operator:** SYSTEM
Injection date: 12/18/2014 10:24:53 AM
Acq. method: ADH75B25A.45MIN.1.5 ML.30UL.M

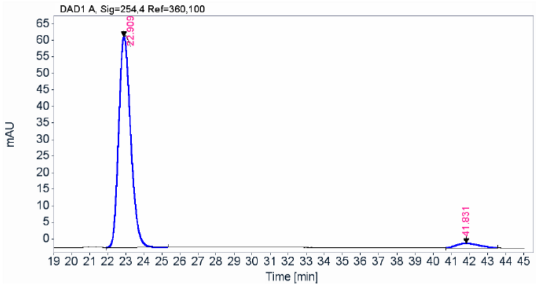

Signal: DAD1 A, Sig=254,4 Ref=360,100

RT [min]	Type	Width [min]	Area	Height	Area%
22.909	BB	0.7125	2927.579	63.6605	95.94
41.831	MM	1.4898	124.044	1.3877	4.06

Fraction 5 (90% ee):

Data file: C:\CHEM32\1\DATA\ARB2014\DEF_LC 2014-12-18 10-18-18\ARB-AM641-FR30B.D
Sample name: arb-am641-fr30b
Instrument: AGILENT 1260 **Acq. operator:** SYSTEM
Injection date: 12/18/2014 11:11:05 AM
Acq. method: ADH75B25A.45MIN.1.5 ML.30UL.M

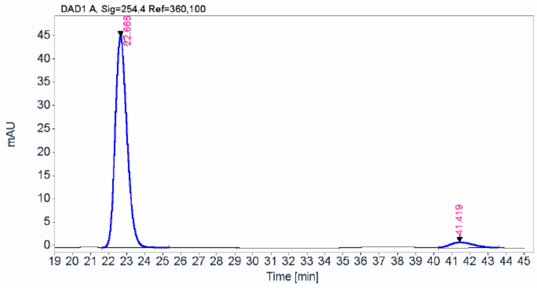

Signal: DAD1 A, Sig=254,4 Ref=360,100

RT [min]	Type	Width [min]	Area	Height	Area%
22.666	BB	0.7003	2044.489	45.1458	95.15
41.419	MM	1.5174	104.116	1.1436	4.85

Fraction 9 (89% ee):

Data file: C:\CHEM32\1\DATA\ARB2014\DEF_LC 2014-12-18 10-18-18\ARB-AM641-FR34B.D
Sample name: arb-am641-fr34b
Instrument: AGILENT 1260 **Acq. operator:** SYSTEM
Injection date: 12/18/2014 11:57:21 AM
Acq. method: ADH75B25A.45MIN.1.5 ML.30UL.M

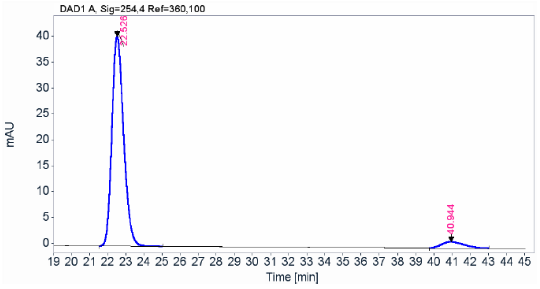

Signal: DAD1 A, Sig=254,4 Ref=360,100

RT [min]	Type	Width [min]	Area	Height	Area%
22.526	BB	0.6971	1813.603	40.4484	94.29
40.944	MM	1.5052	109.922	1.2171	5.71

Preliminary Kinetic Experiments

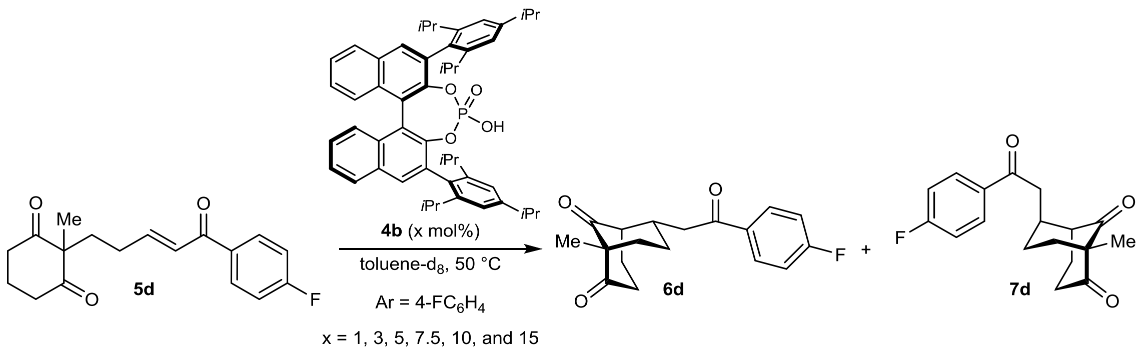

Representative procedure for experiment conducted at 1 mol% catalyst loading:

A solution of enone **5d** (30 mg, 0.10 mmol), fluorobenzene (internal reference, *ca.* 10 μL), and (*R*)-TRIP (**4b**, 0.0547 M in toluene-d_8, 18 μL, 0.001 mmol) in toluene-d_8 (1 mL) was stirred at room temperature for 5 min until the solution became homogeneous. A 0.5 mL aliquot was removed and transferred to an NMR tube. ^{19}F NMR analysis of the sample at 50 °C was performed (with a d1 relaxation time of 5 s to increase the accuracy of integration), with spectra taken every 137 s until the reaction reached approximately 20% conversion. From a plot of [**5d**] against time, the initial rate for the reaction was calculated.

This process was repeated for catalyst loadings of 3, 5, 7.5, 10, and 15 mol%, and initial rates for each experiment were calculated. A plot of ln[initial rate] against ln[**4b**] gives a straight line, the gradient of which corresponds to the reaction order with respect to catalyst.

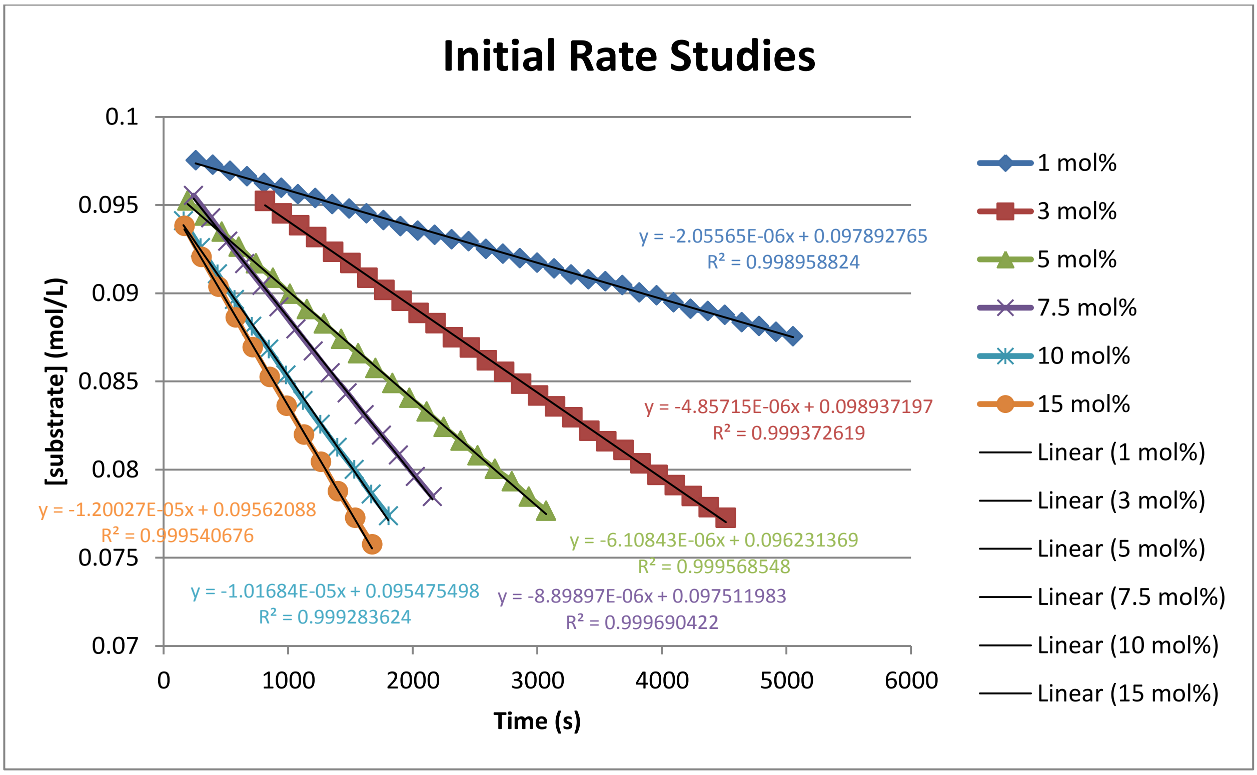

Conc. of catalyst (M)	ln[catalyst]	Initial rate (molL^{-1}s^{-1})	ln[initial rate]
0.001	-6.907755279	2.05565E-06	-13.0949
0.003	-5.80914299	4.85715E-06	-12.2351
0.005	-5.298317367	6.10843E-06	-12.0058
0.0075	-4.892852258	8.89897E-06	-11.6296
0.01	-4.605170186	1.01684E-05	-11.4962
0.015	-4.199705078	1.20027E-05	-11.3304

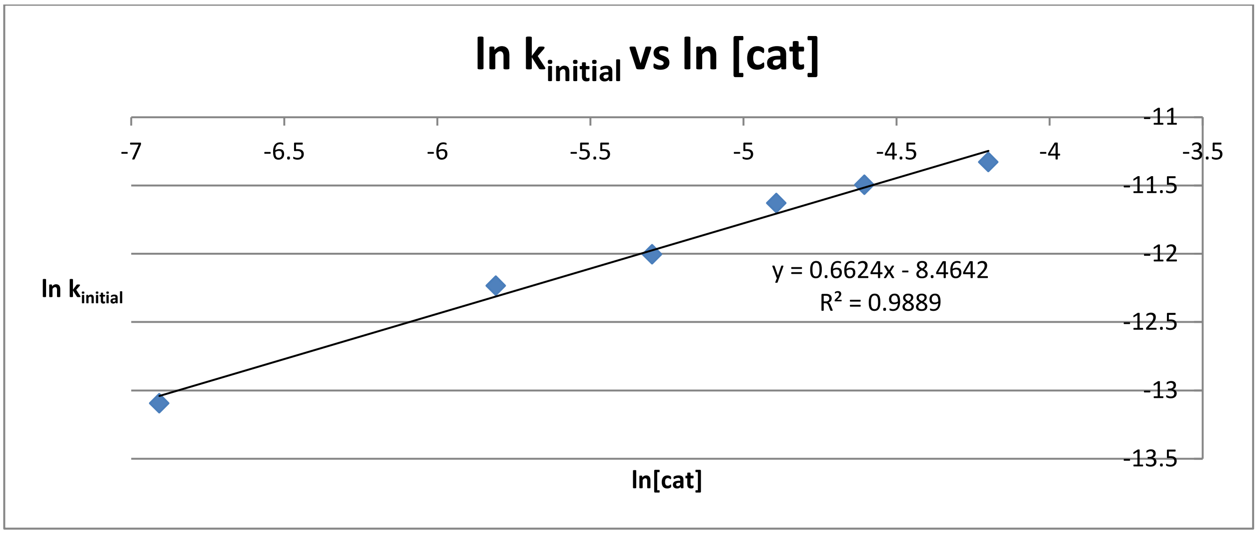

Therefore, the order with respect to catalyst **4b** is **0.66**.

Measurement of the Enantioselectivity of 2j with Increasing Conversion

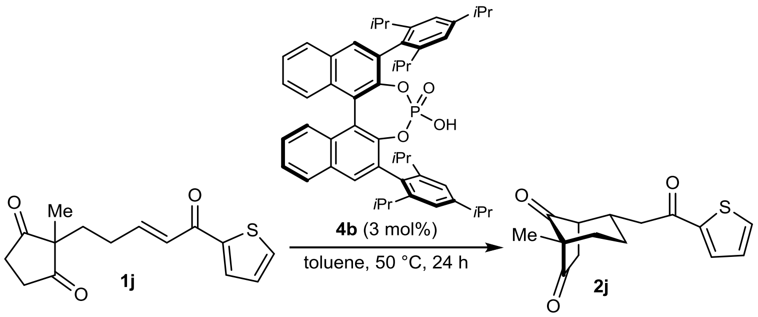

A solution of enone **1j** (138.0 mg, 0.50 mmol) and (*R*)-TRIP (**4b**, 11.3 mg, 0.015 mmol) in toluene (5 mL) was stirred at 50 °C for 24 h (toluene was selected as the solvent rather than cyclohexane to ensure complete homogeneity of the reaction mixture). In order to measure the enantiomeric excess of **2j** over the course of the reaction, 400 µL aliquots were removed at various time intervals. The aliquots were concentrated *in vacuo* and analyzed by ^{1}H NMR spectroscopy to determine the conversion. A small quantity of the aliquot was then purified by preparative thin-layer chromatography to obtain a pure sample of **2j** for measuring the enantiomeric excess. (The

enantiomeric excess of several aliquots were checked before and after concentration *in vacuo*; no changes in enantiomeric excess were observed.) The following results were obtained:

**Time (h)**	**Conversion (%)**	**ee** (%)
1	15	70
2	18	74
3	21	80
4	25	81
5	28	81
7	35	86
9.5	46	88
24	75	90

HPLC conditions: Chiralpak IA-3 column (70:30 *iso*-hexane:*i*-PrOH, 1.5 mL/min, 230 nm, 25 °C).

Racemate

data acquired by: AM
on: 5/22/2014
location: Vial 53

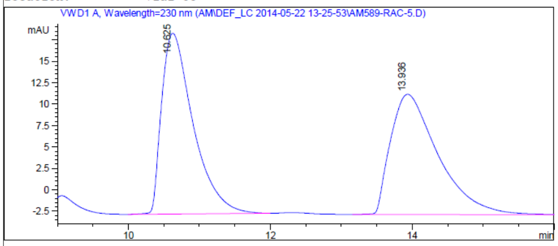

Meas. R	Area %	Width	Symmetr.
10.625	49.780	0.475	0.509
13.936	50.220	0.723	0.524

1 h, 15% conversion, 70% ee

data acquired by: HWL
on: 4/1/2015
location: Vial 31

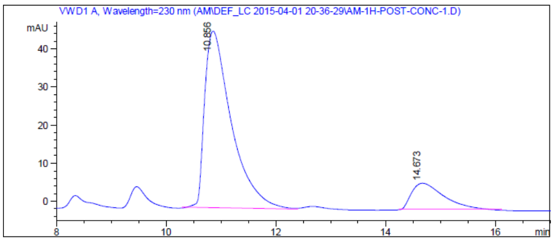

Meas. R	Area %	Width	Symmetr.
10.856	85.061	0.565	0.442
14.673	14.939	0.674	0.499

2 h, 18% conversion, 74% ee

data acquired by: HWL
on: 4/1/2015
location: Vial 32

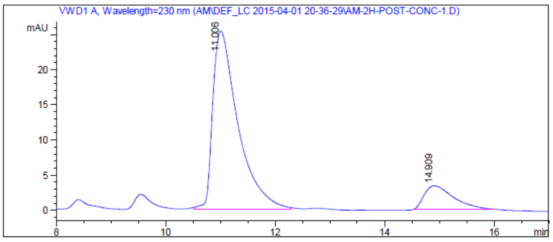

Meas. R	Area %	Width	Symmetr.
11.006	86.853	0.535	0.463
14.909	13.147	0.616	0.522

3 h, 21% conversion, 80% ee

data acquired by: HWL
on: 4/1/2015
location: Vial 33

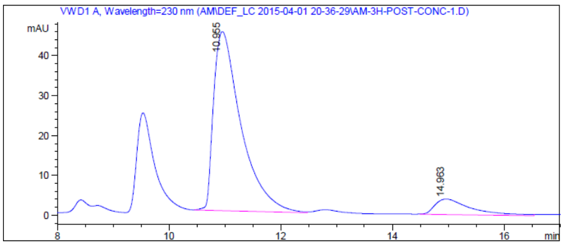

Meas. R	Area %	Width	Symmetr.
10.955	89.788	0.570	0.436
14.963	10.212	0.747	0.438

4 h, 25% conversion, 81% ee

data acquired by: HWL
on: 4/1/2015
location: Vial 34

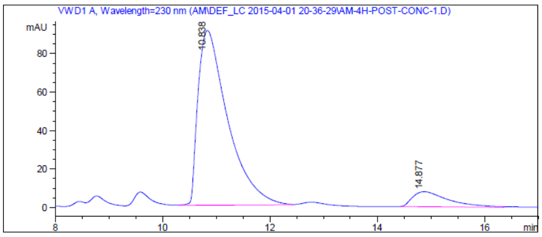

Meas. R	Area %	Width	Symmetr.
10.838	90.600	0.641	0.438
14.877	9.400	0.781	0.453

5 h, 28% conversion, 81% ee

data acquired by: HWL
on: 4/1/2015
location: Vial 35

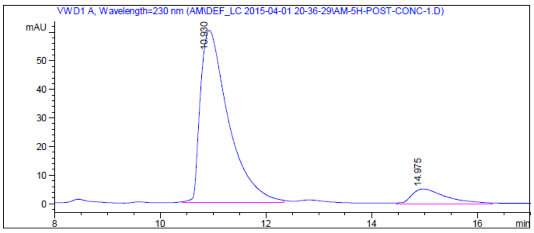

Meas. R	Area %	Width	Symmetr.
10.930	90.440	0.610	0.433
14.975	9.560	0.751	0.469

7 h, 35% conversion, 86% ee

data acquired by: HWL
on: 4/1/2015
location: Vial 36

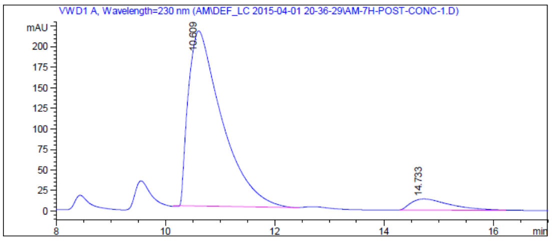

Meas. R	Area %	Width	Symmetr.
10.609	93.181	0.714	0.412
14.733	6.819	0.824	0.471

9.5 h, 46% conversion, 88% ee

data acquired by: HWL
on: 4/1/2015
location: Vial 37

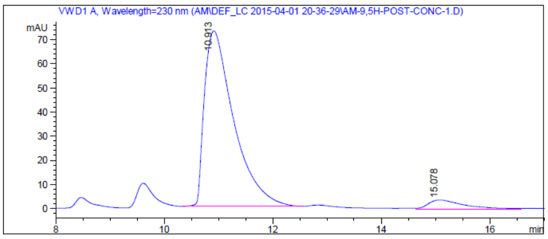

Meas. R	Area %	Width	Symmetr.
10.913	94.174	0.627	0.461
15.078	5.826	0.753	0.462

24 h, 75% conversion, 90% ee

data acquired by: HWL
on: 4/2/2015
location: Vial 42

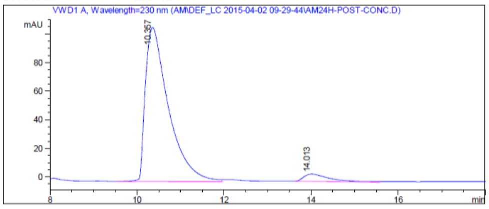

Meas. R	Area %	Width	Symmetr.
10.357	95.061	0.546	0.413
14.013	4.939	0.684	0.416

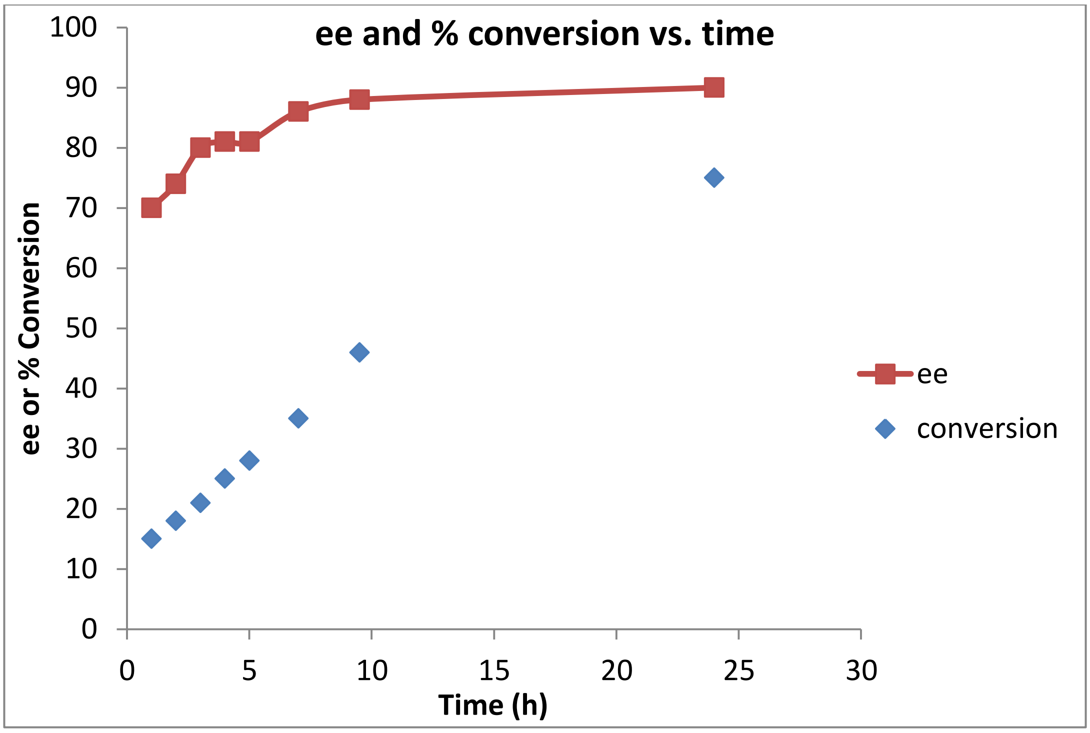
ee and % conversion vs. time
ee or % Conversion
100
90
80
70
60
50
40
30
20
10
0
0
5
10
15
20
25
30
Time (h)
ee
conversion

NMR Spectra

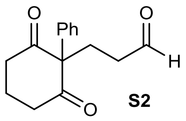

S2

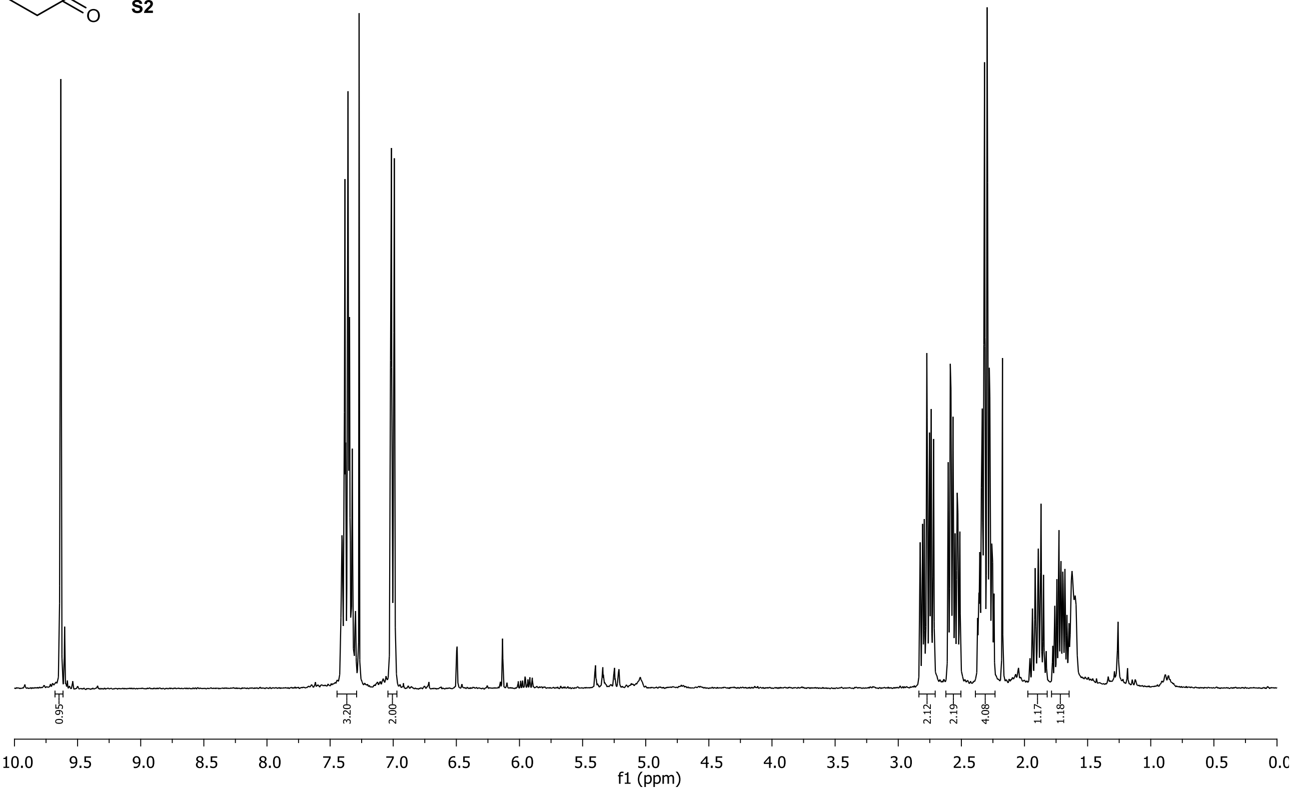

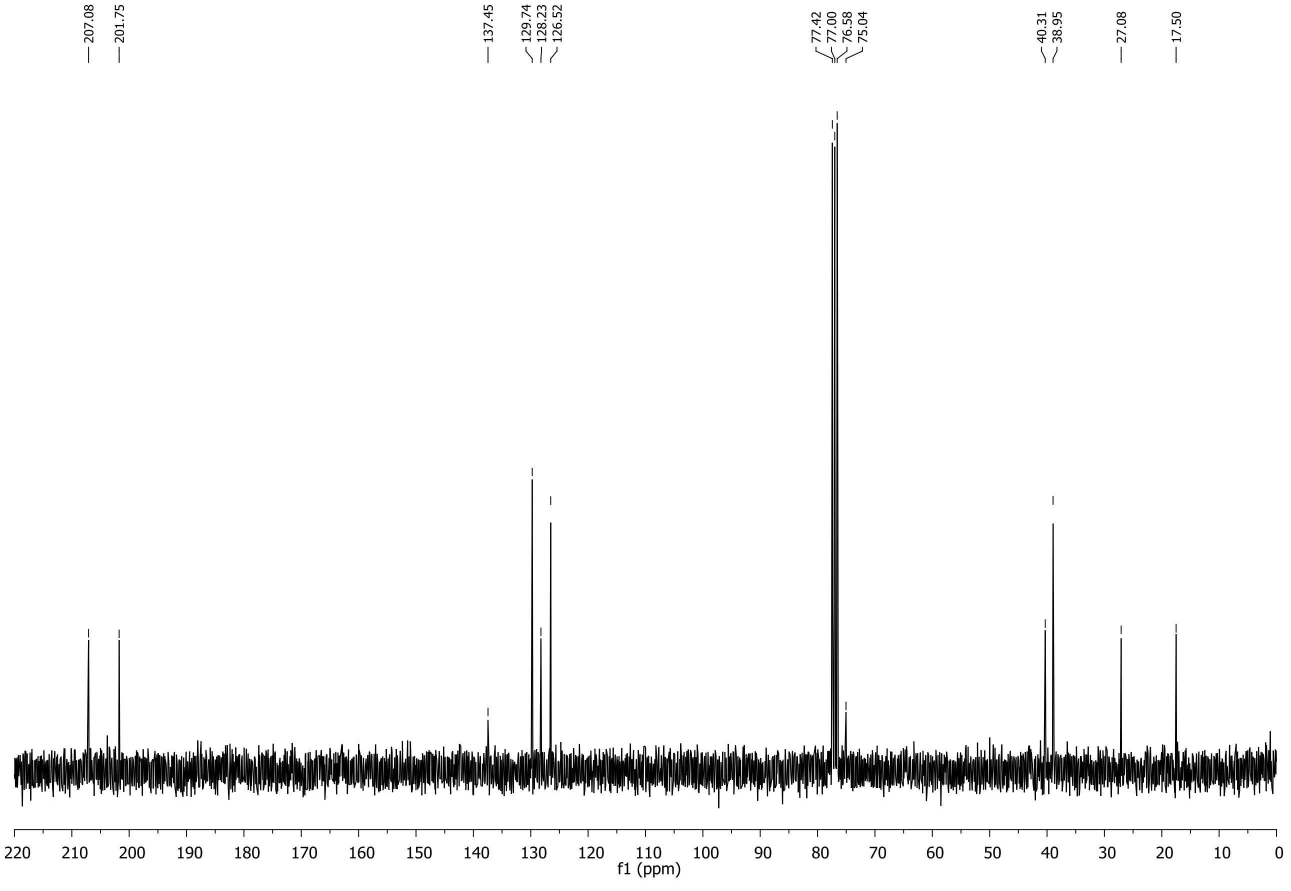

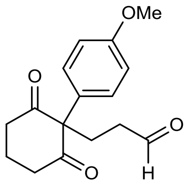
OMe
O
O
O
H

S3

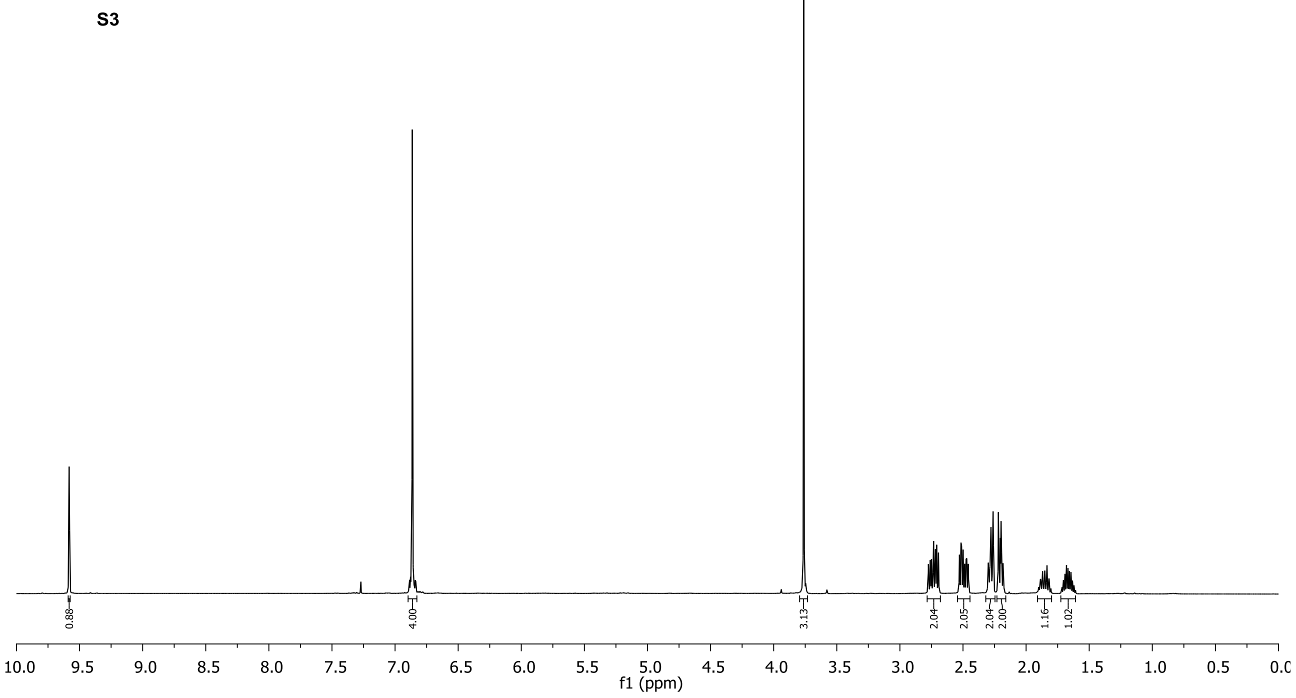
0.88
4.00
3.13
2.04
2.05
2.04
2.00
1.16
1.02
10.0 9.5 9.0 8.5 8.0 7.5 7.0 6.5 6.0 5.5 5.0 4.5 4.0 3.5 3.0 2.5 2.0 1.5 1.0 0.5 0.0
f1 (ppm)

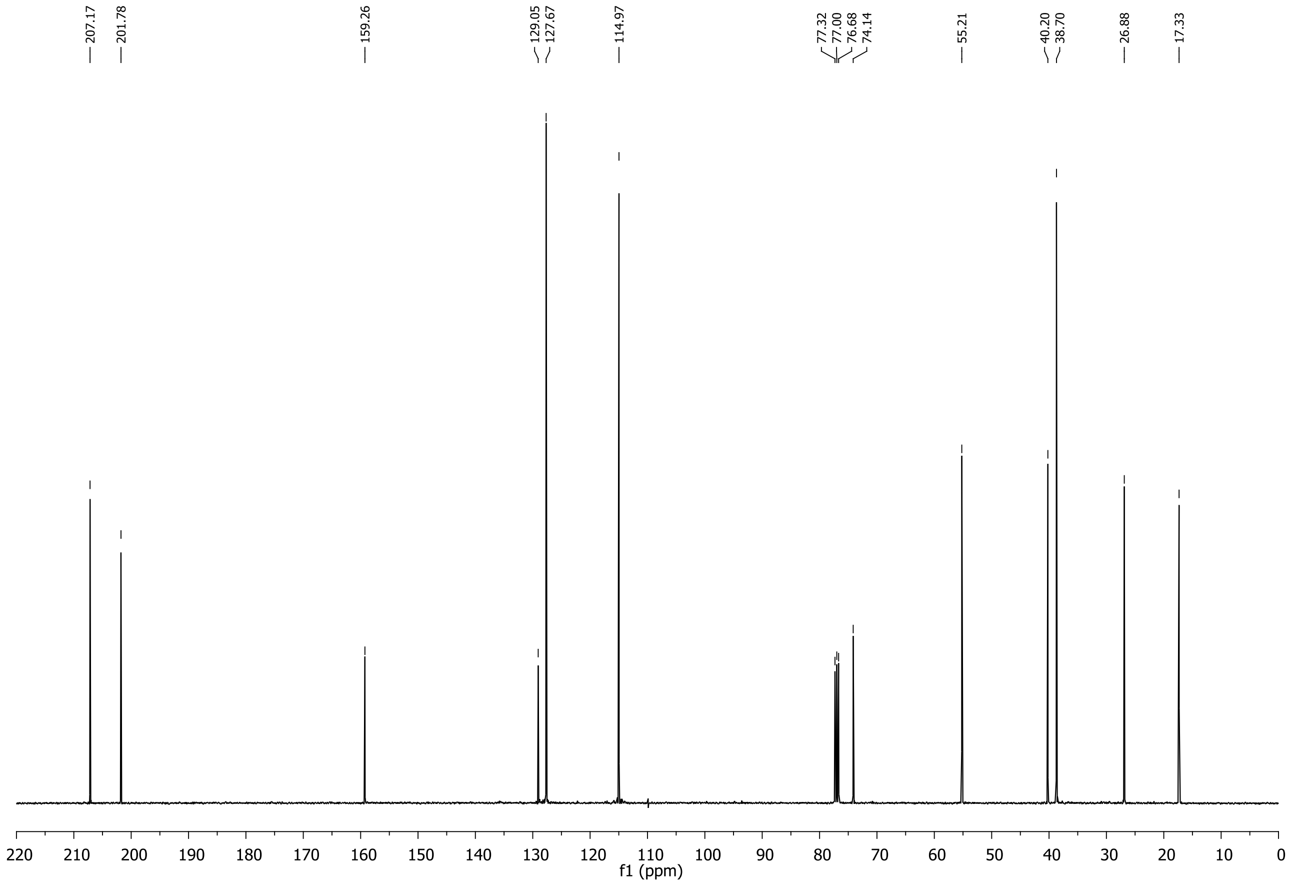
207.17
201.78
159.26
129.05
127.67
114.97
77.32
77.00
76.68
74.14
55.21
40.20
38.70
26.88
17.33
220 210 200 190 180 170 160 150 140 130 120 110 100 90 80 70 60 50 40 30 20 10 0
f1 (ppm)

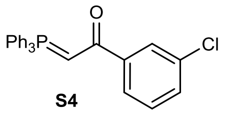
Ph3P
O
Cl

S4

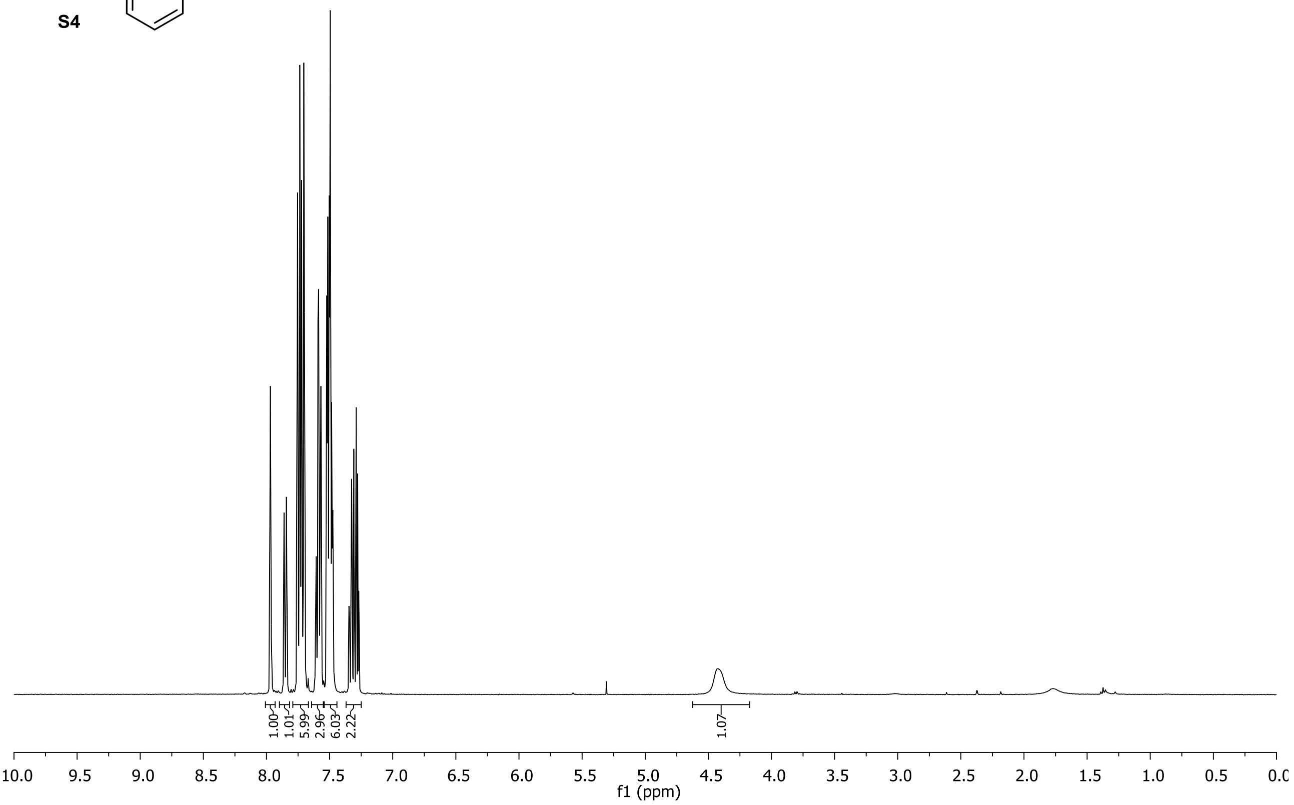
1.00
1.01
5.99
2.96
6.03
2.22
1.07
10.0 9.5 9.0 8.5 8.0 7.5 7.0 6.5 6.0 5.5 5.0 4.5 4.0 3.5 3.0 2.5 2.0 1.5 1.0 0.5 0.0
f1 (ppm)

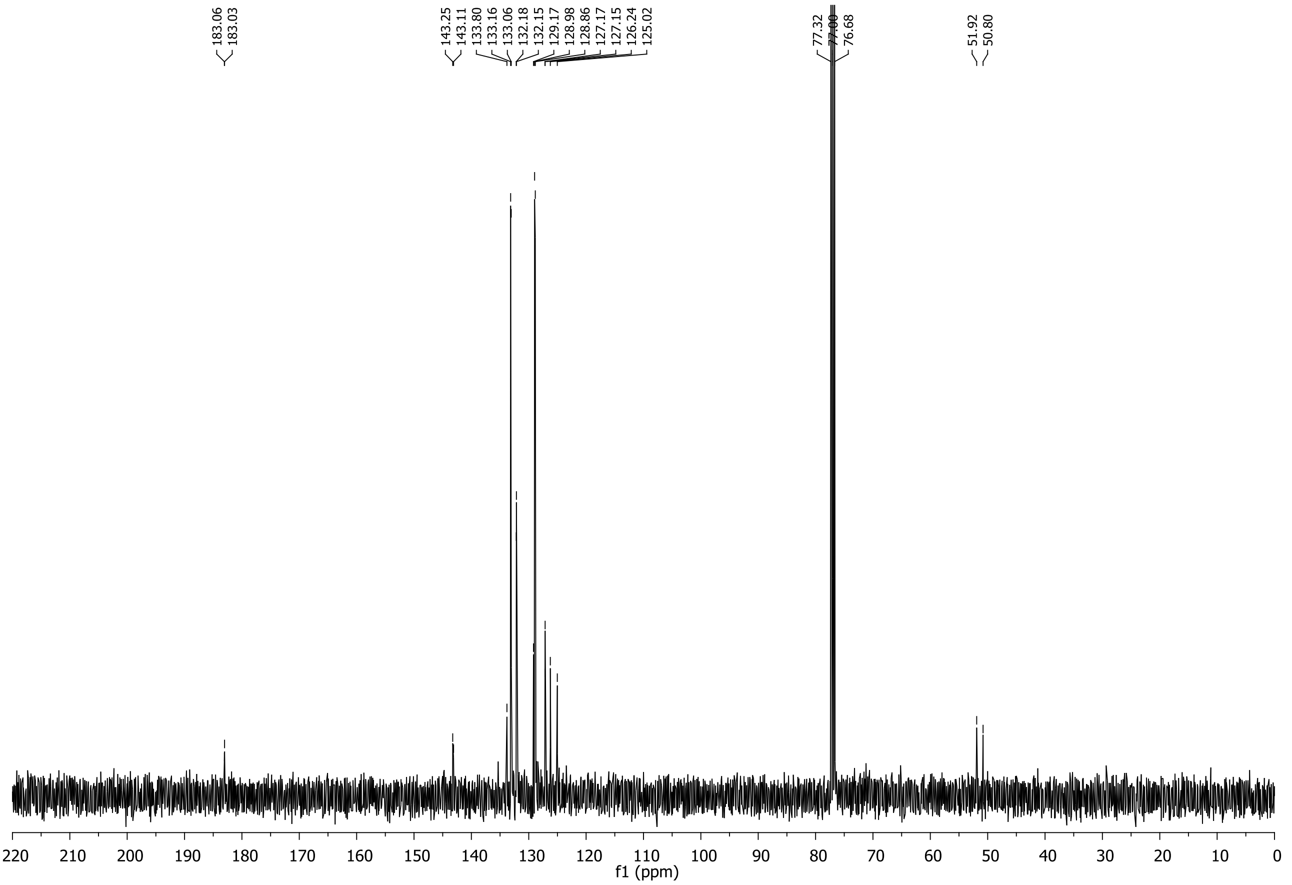
183.06
183.03
143.25
143.11
133.80
133.16
133.06
132.18
132.15
129.17
128.98
128.86
127.17
127.15
126.24
125.02
77.32
77.00
76.68
51.92
50.80
220 210 200 190 180 170 160 150 140 130 120 110 100 90 80 70 60 50 40 30 20 10 0
f1 (ppm)

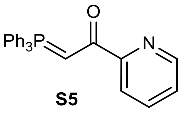
O
Ph3P
N

S5

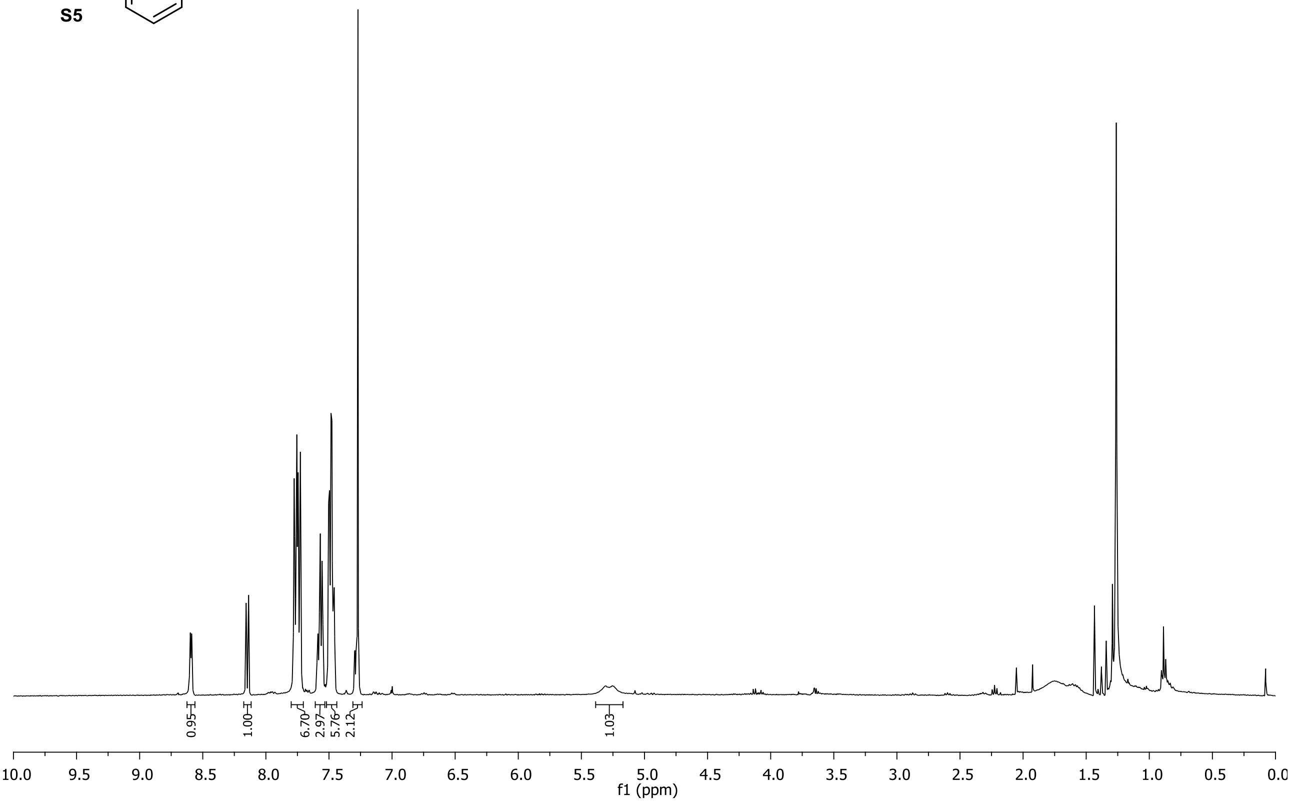
0.95
1.00
6.70
2.97
5.76
2.12
1.03
10.0 9.5 9.0 8.5 8.0 7.5 7.0 6.5 6.0 5.5 5.0 4.5 4.0 3.5 3.0 2.5 2.0 1.5 1.0 0.5 0.0
f1 (ppm)

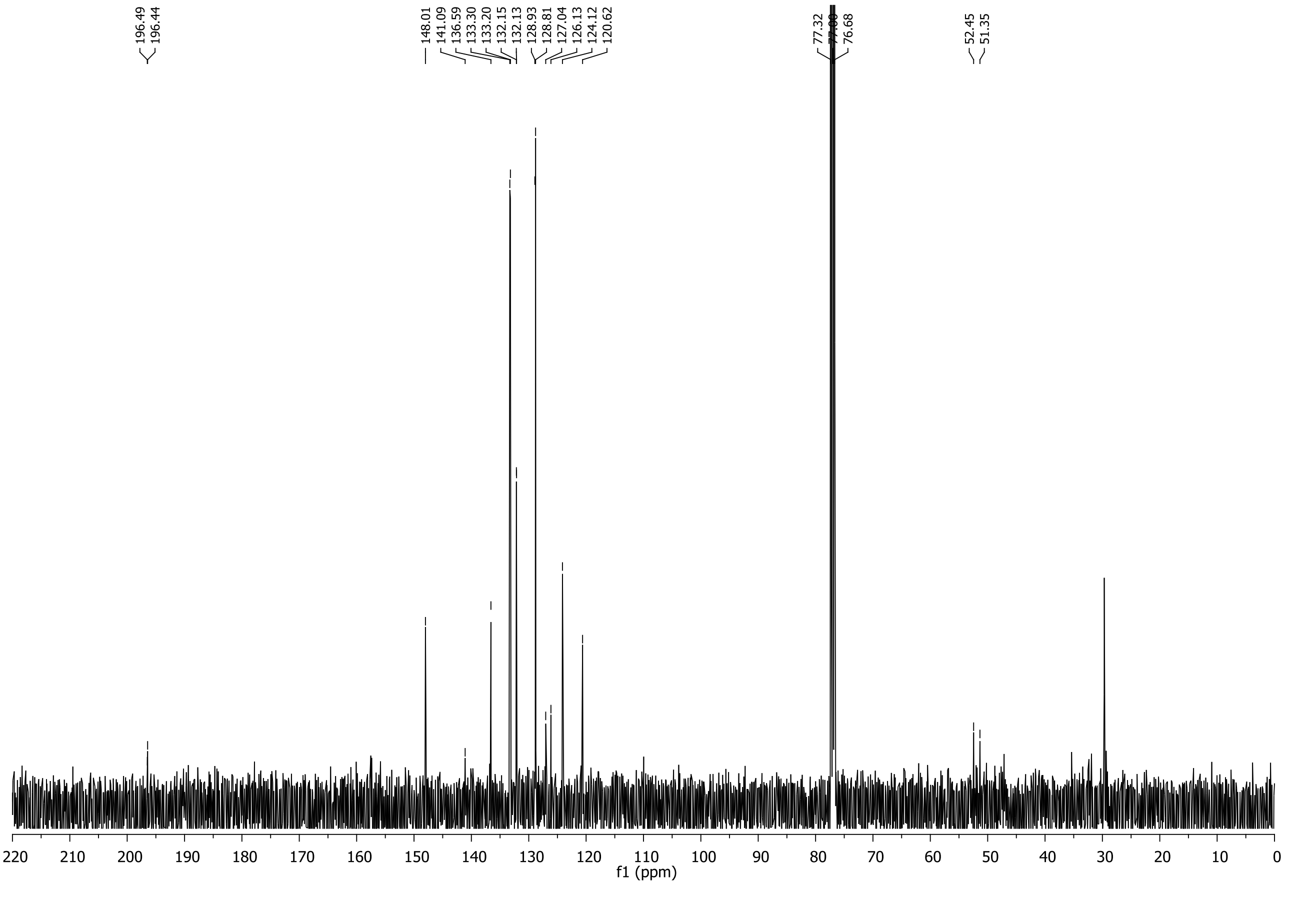
196.49
196.44
148.01
141.09
136.59
133.30
133.20
132.15
132.13
128.93
128.81
127.04
126.13
124.12
120.62
77.32
77.00
76.68
52.45
51.35
220 210 200 190 180 170 160 150 140 130 120 110 100 90 80 70 60 50 40 30 20 10 0
f1 (ppm)

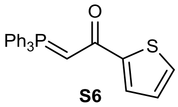
Ph3P
O
S

S6

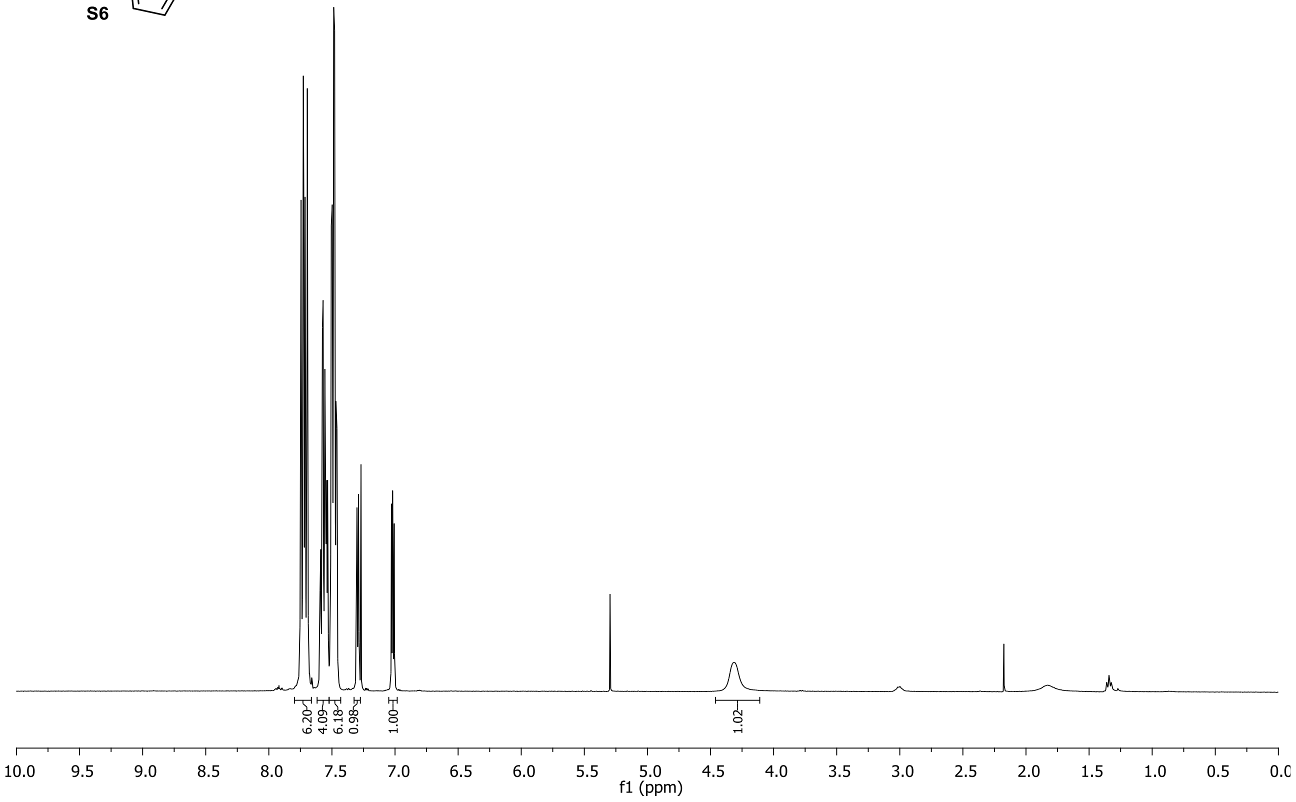
6.20
4.09
6.18
0.98
1.00
1.02
10.0 9.5 9.0 8.5 8.0 7.5 7.0 6.5 6.0 5.5 5.0 4.5 4.0 3.5 3.0 2.5 2.0 1.5 1.0 0.5 0.0
f1 (ppm)

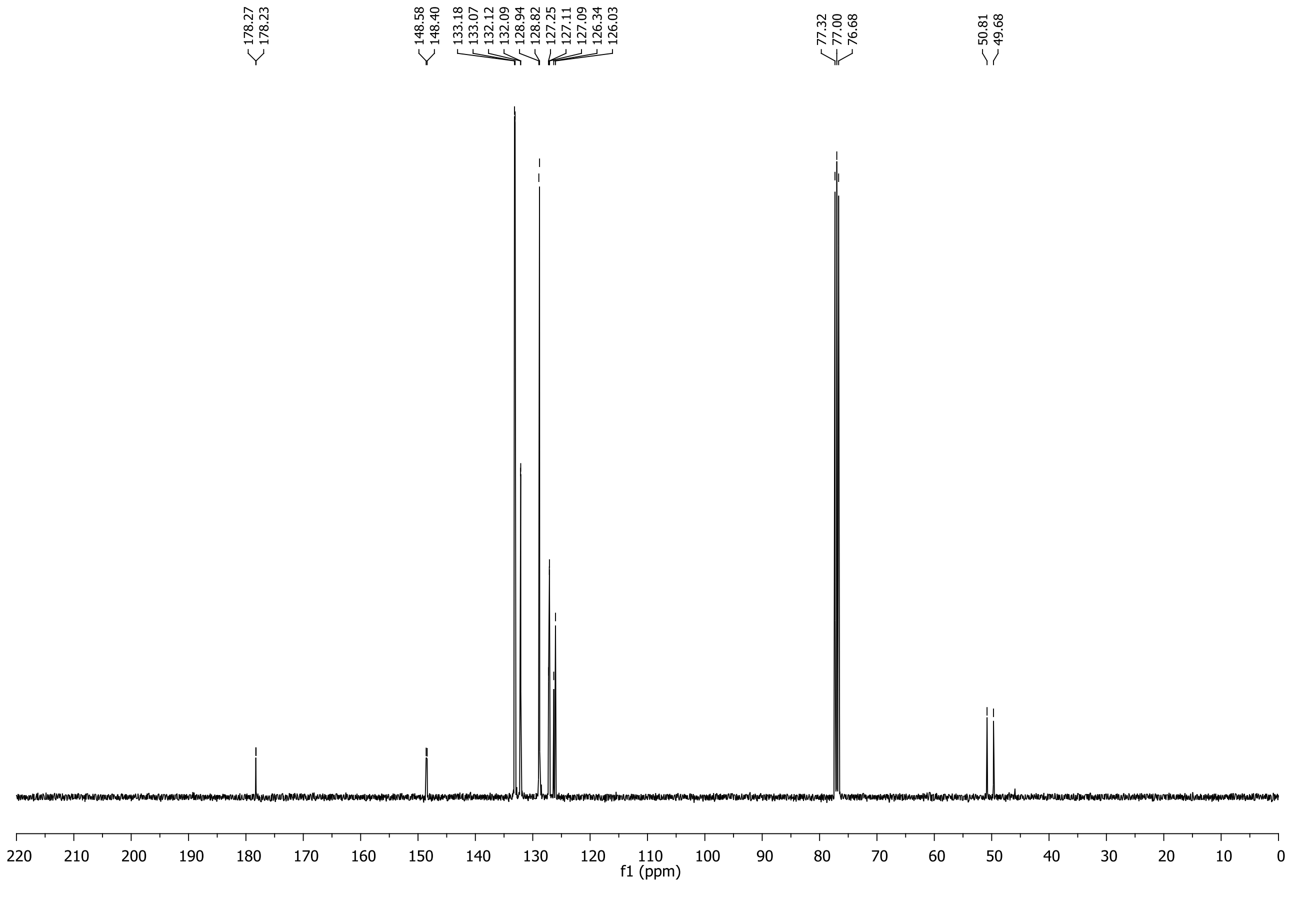
178.27
178.23
148.58
148.40
133.18
133.07
132.12
132.09
128.94
128.82
127.25
127.11
127.09
126.34
126.03
77.32
77.00
76.68
50.81
49.68
220 210 200 190 180 170 160 150 140 130 120 110 100 90 80 70 60 50 40 30 20 10 0
f1 (ppm)

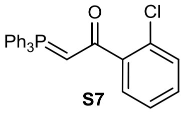
O
Cl
Ph3P

S7

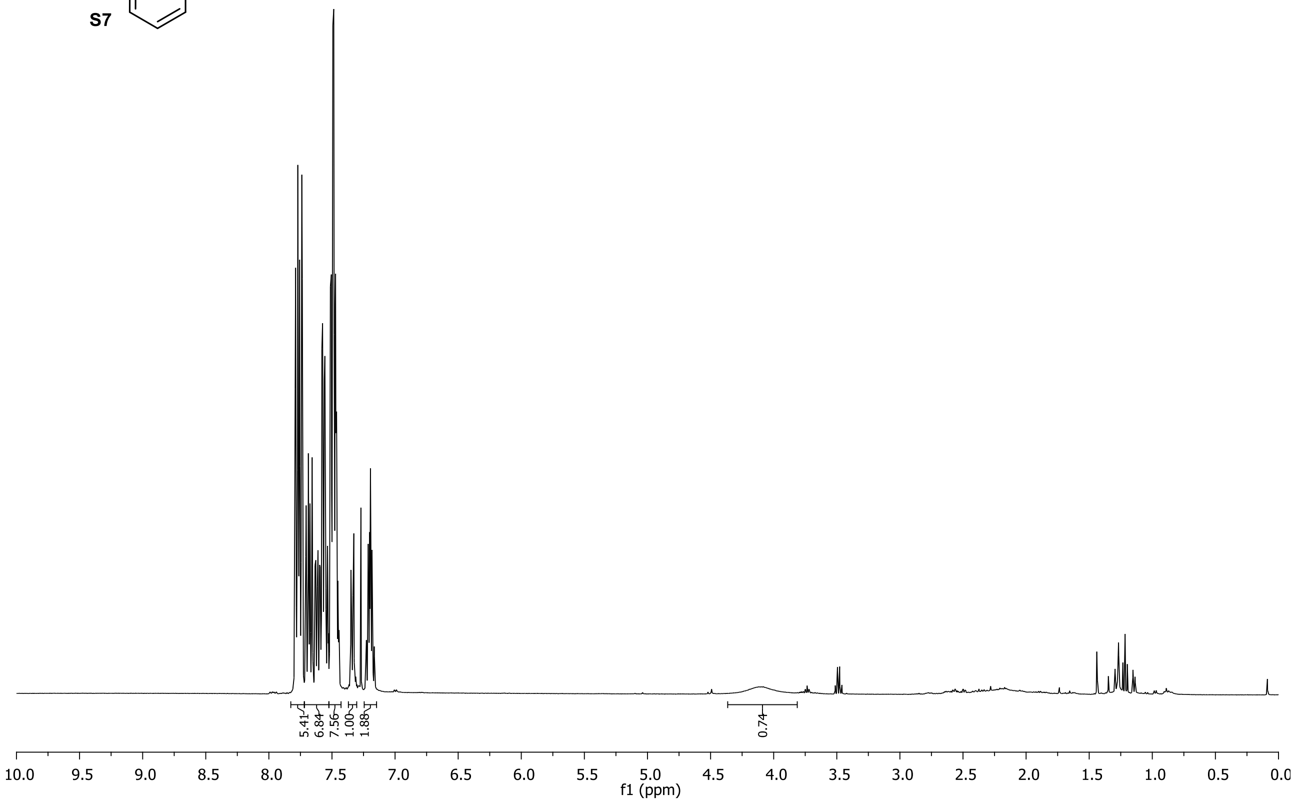
5.41
6.84
7.56
1.00
1.88
0.74
10.0 9.5 9.0 8.5 8.0 7.5 7.0 6.5 6.0 5.5 5.0 4.5 4.0 3.5 3.0 2.5 2.0 1.5 1.0 0.5 0.0
f1 (ppm)

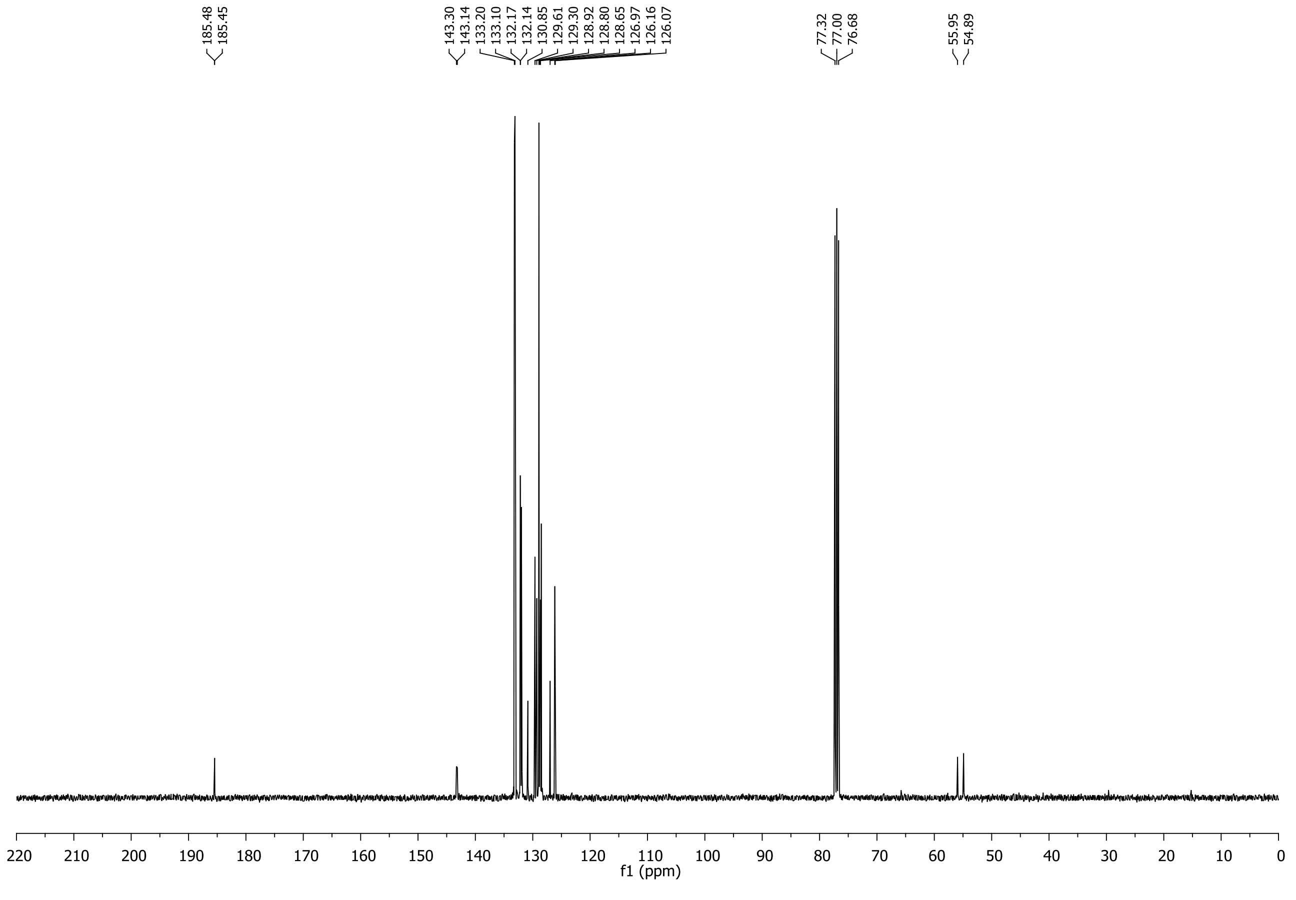
185.48
185.45
143.30
143.14
133.20
133.10
132.17
132.14
130.85
129.61
129.30
128.92
128.80
128.65
126.97
126.16
126.07
77.32
77.00
76.68
55.95
54.89
220 210 200 190 180 170 160 150 140 130 120 110 100 90 80 70 60 50 40 30 20 10 0
f1 (ppm)

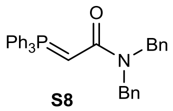
Ph3P
O
N
Bn
Bn

S8

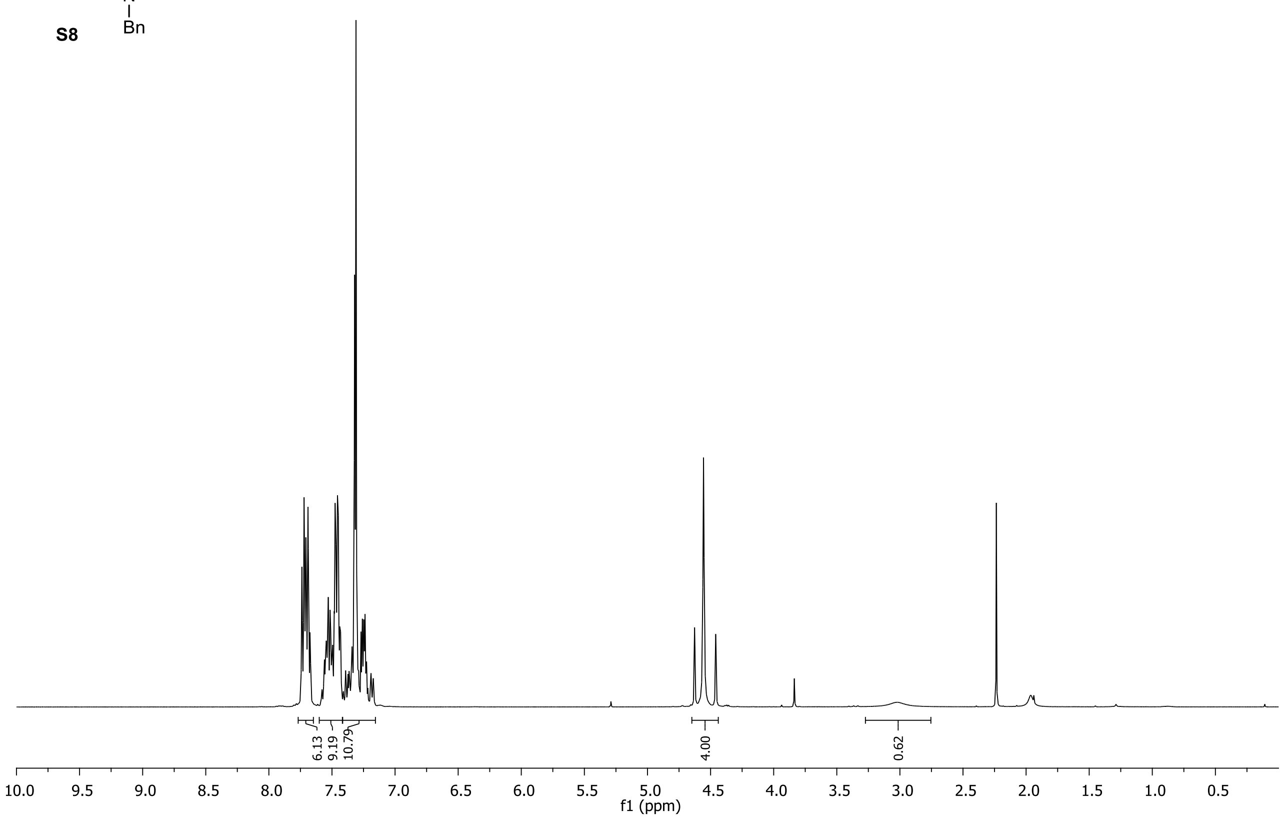
6.13
9.19
10.79
4.00
0.62
10.0 9.5 9.0 8.5 8.0 7.5 7.0 6.5 6.0 5.5 5.0 4.5 4.0 3.5 3.0 2.5 2.0 1.5 1.0 0.5
f1 (ppm)

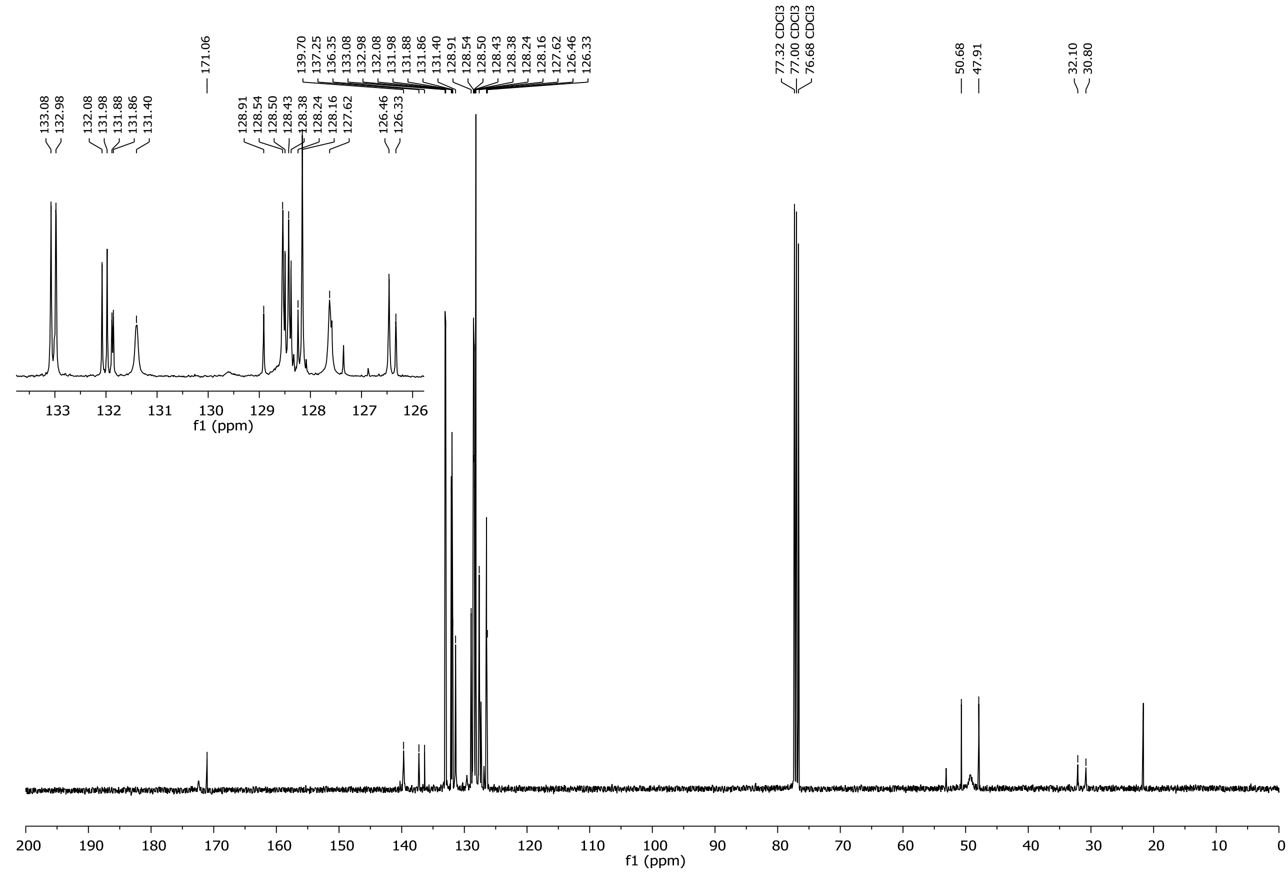
171.06
139.70
137.25
136.35
133.08
132.98
132.08
131.98
131.88
131.86
131.40
128.91
128.54
128.50
128.43
128.38
128.24
128.16
127.62
126.46
126.33
77.32 CDCl3
77.00 CDCl3
76.68 CDCl3
50.68
47.91
32.10
30.80
133 132 131 130 129 128 127 126
f1 (ppm)
200 190 180 170 160 150 140 130 120 110 100 90 80 70 60 50 40 30 20 10 0
f1 (ppm)

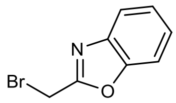
N
Br
O

S9

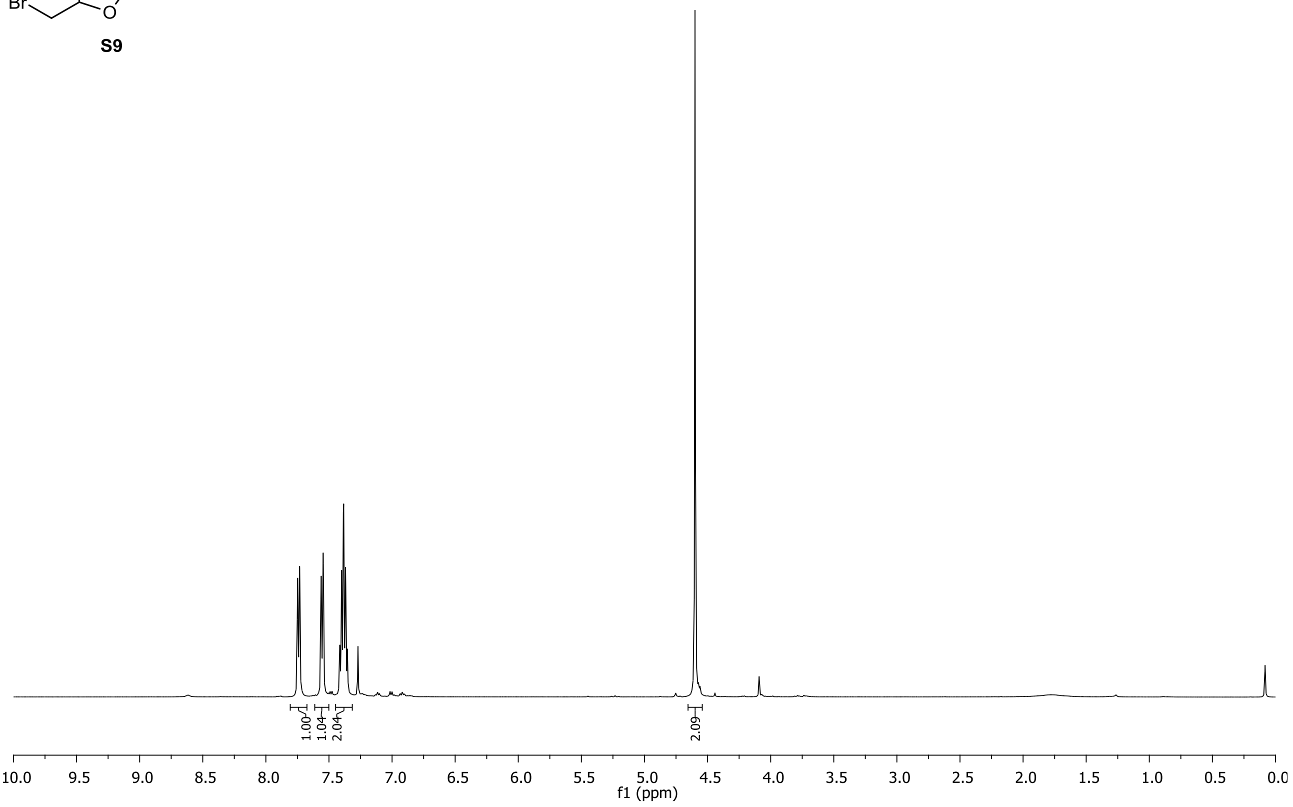
1.00
1.04
2.04
2.09
10.0 9.5 9.0 8.5 8.0 7.5 7.0 6.5 6.0 5.5 5.0 4.5 4.0 3.5 3.0 2.5 2.0 1.5 1.0 0.5 0.0
f1 (ppm)

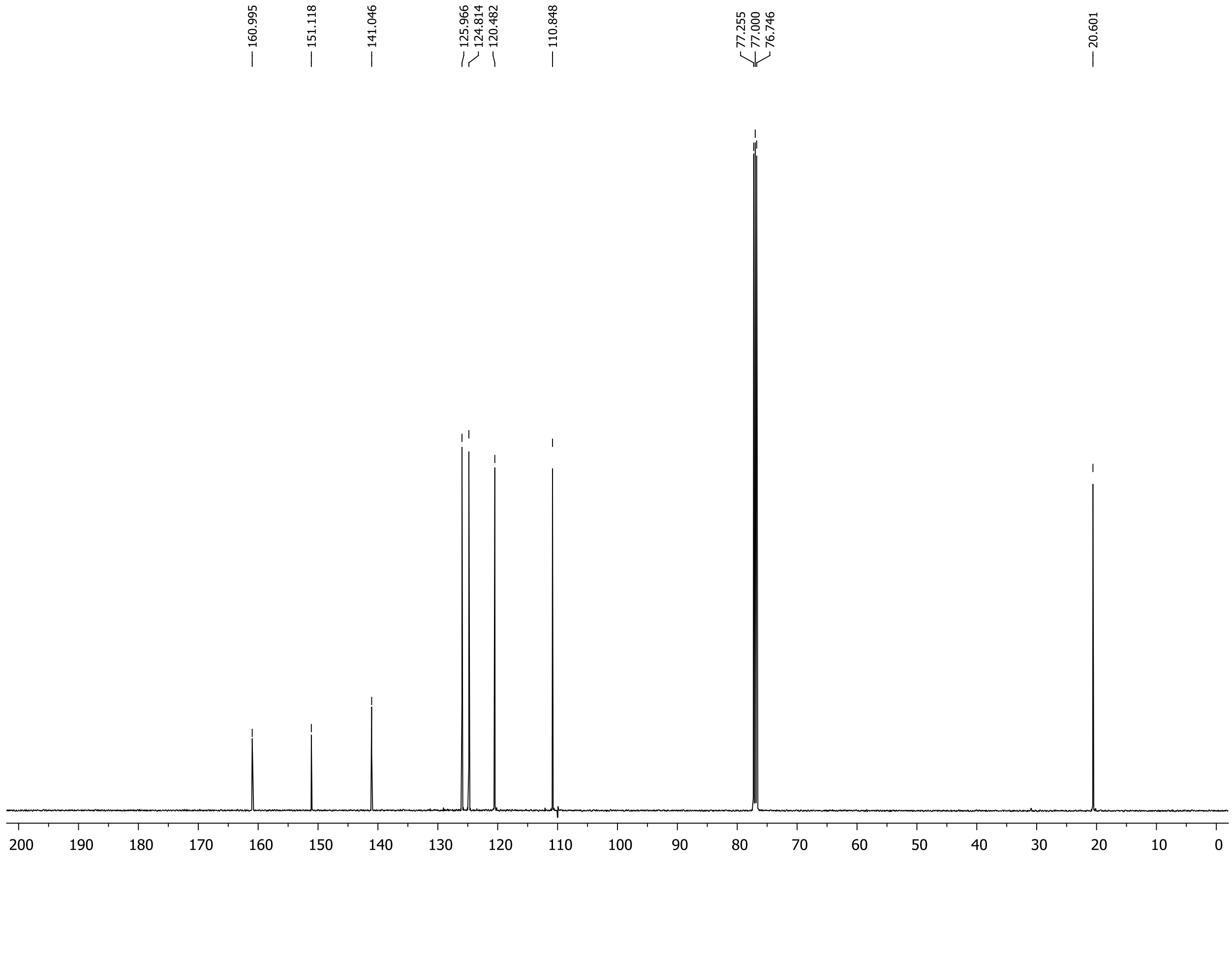
160.995
151.118
141.046
125.966
124.814
120.482
110.848
77.255
77.000
76.746
20.601
200 190 180 170 160 150 140 130 120 110 100 90 80 70 60 50 40 30 20 10 0

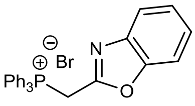
Br
Ph3P
N
O

S10

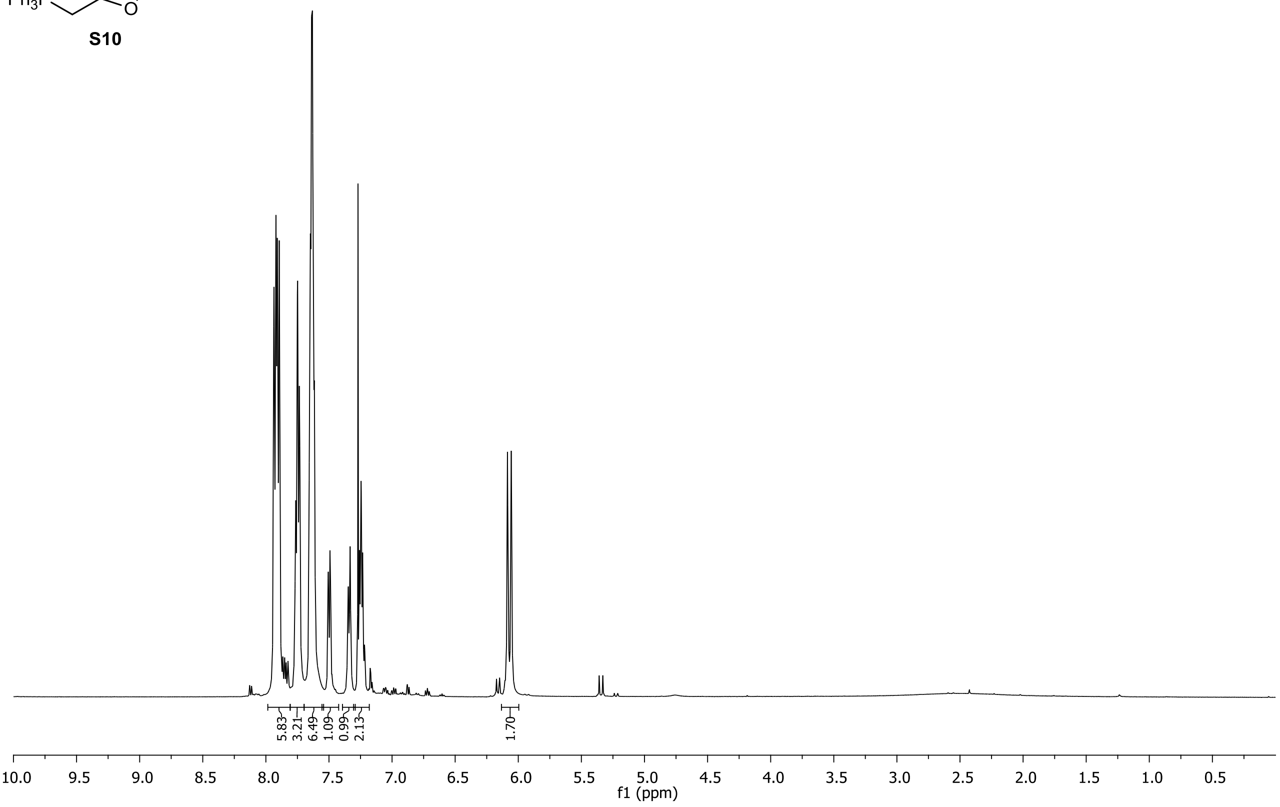
5.83
3.21
6.49
1.09
0.99
2.13
1.70
10.0 9.5 9.0 8.5 8.0 7.5 7.0 6.5 6.0 5.5 5.0 4.5 4.0 3.5 3.0 2.5 2.0 1.5 1.0 0.5
f1 (ppm)

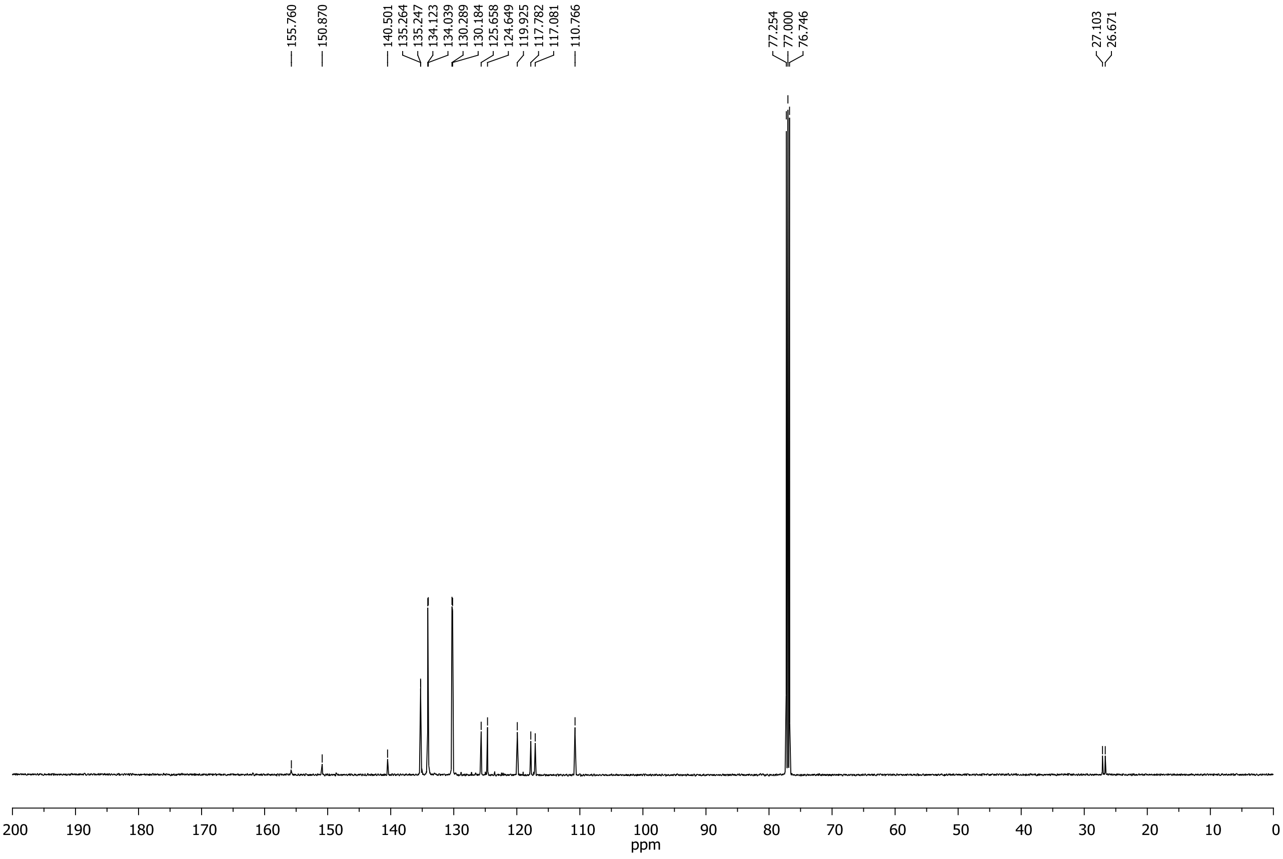
155.760
150.870
140.501
135.264
135.247
134.123
134.039
130.289
130.184
125.658
124.649
119.925
117.782
117.081
110.766
77.254
77.000
76.746
27.103
26.671
200 190 180 170 160 150 140 130 120 110 100 90 80 70 60 50 40 30 20 10 0
ppm

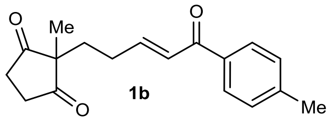
O
Me
O
O
1b
Me

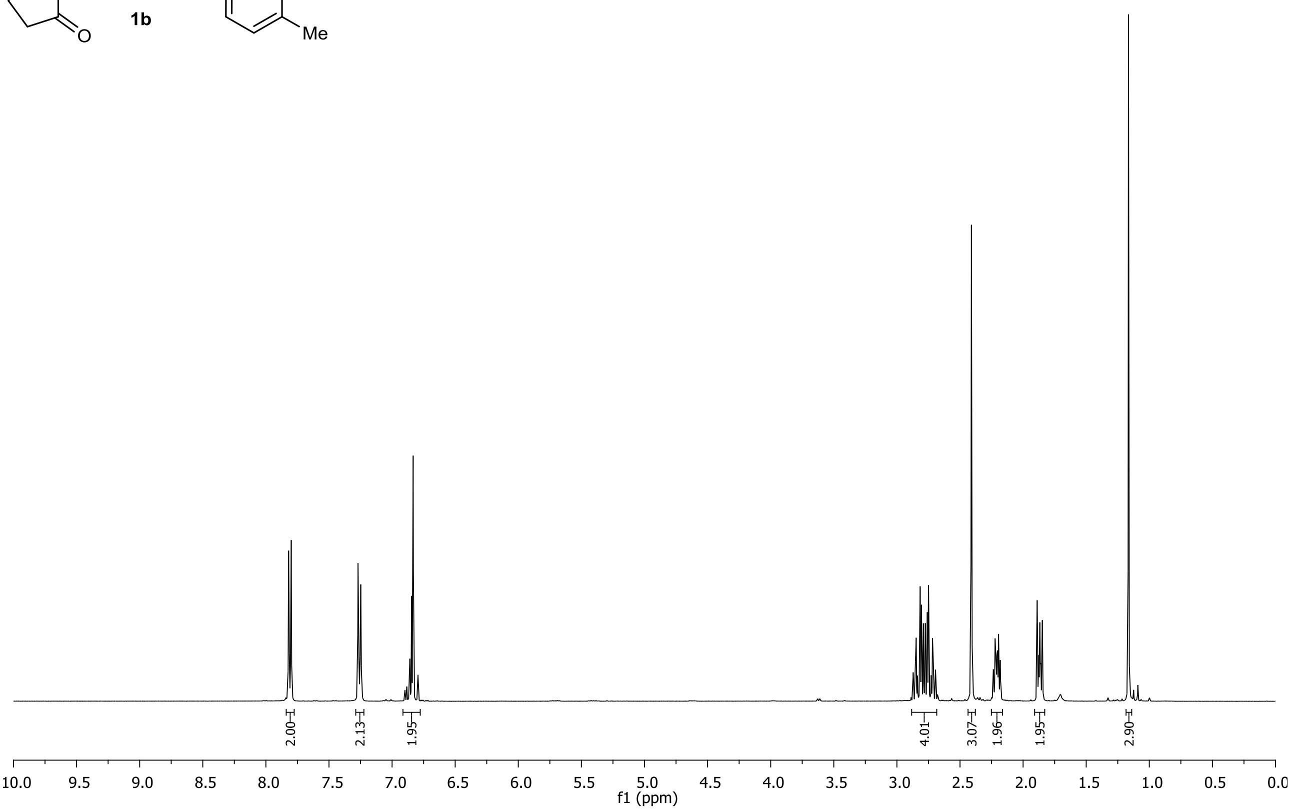
2.00
2.13
1.95
4.01
3.07
1.96
1.95
2.90
10.0 9.5 9.0 8.5 8.0 7.5 7.0 6.5 6.0 5.5 5.0 4.5 4.0 3.5 3.0 2.5 2.0 1.5 1.0 0.5 0.0
f1 (ppm)

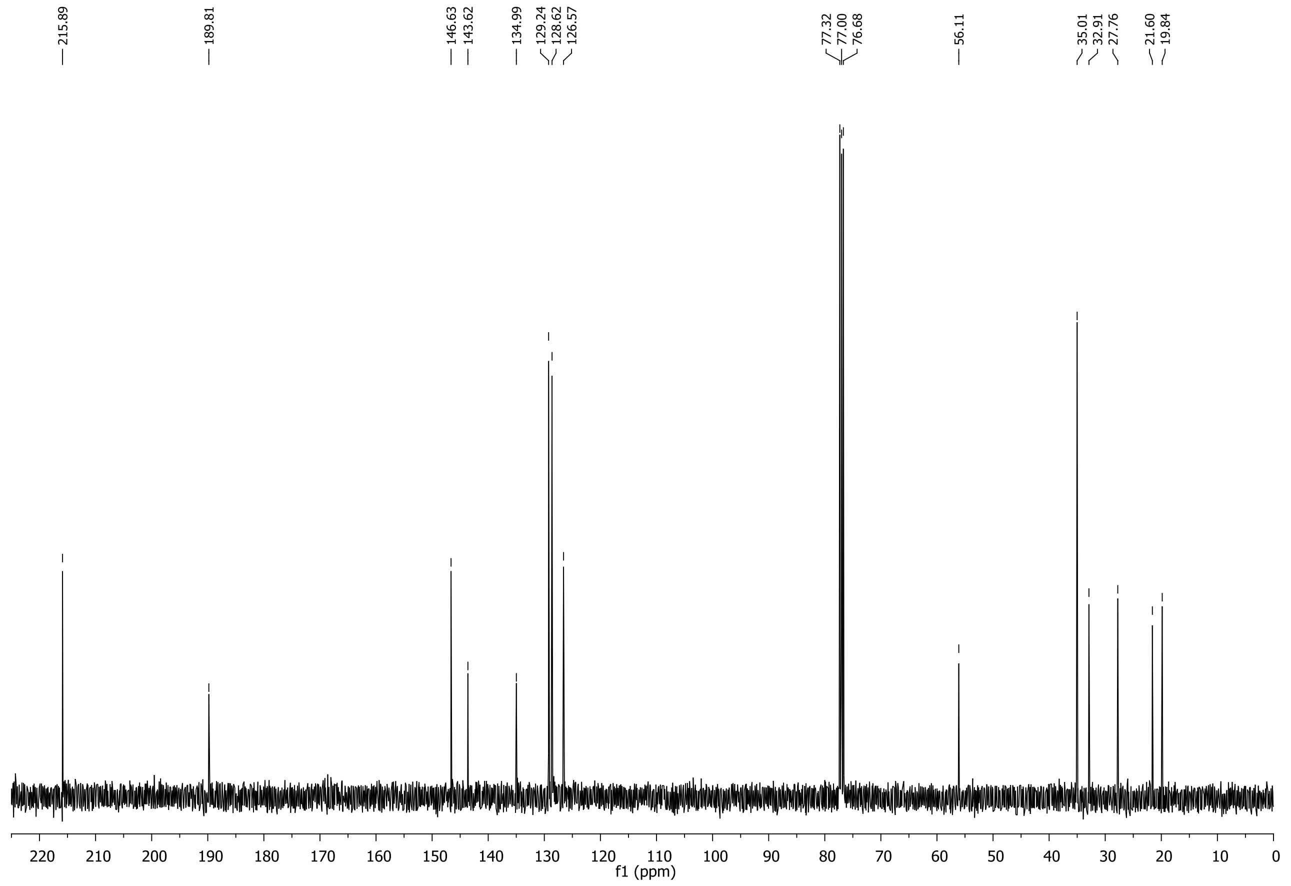
215.89
189.81
146.63
143.62
134.99
129.24
128.62
126.57
77.32
77.00
76.68
56.11
35.01
32.91
27.76
21.60
19.84
220 210 200 190 180 170 160 150 140 130 120 110 100 90 80 70 60 50 40 30 20 10 0
f1 (ppm)

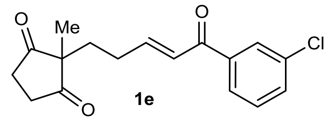
Me
O
O
O
Cl

1e

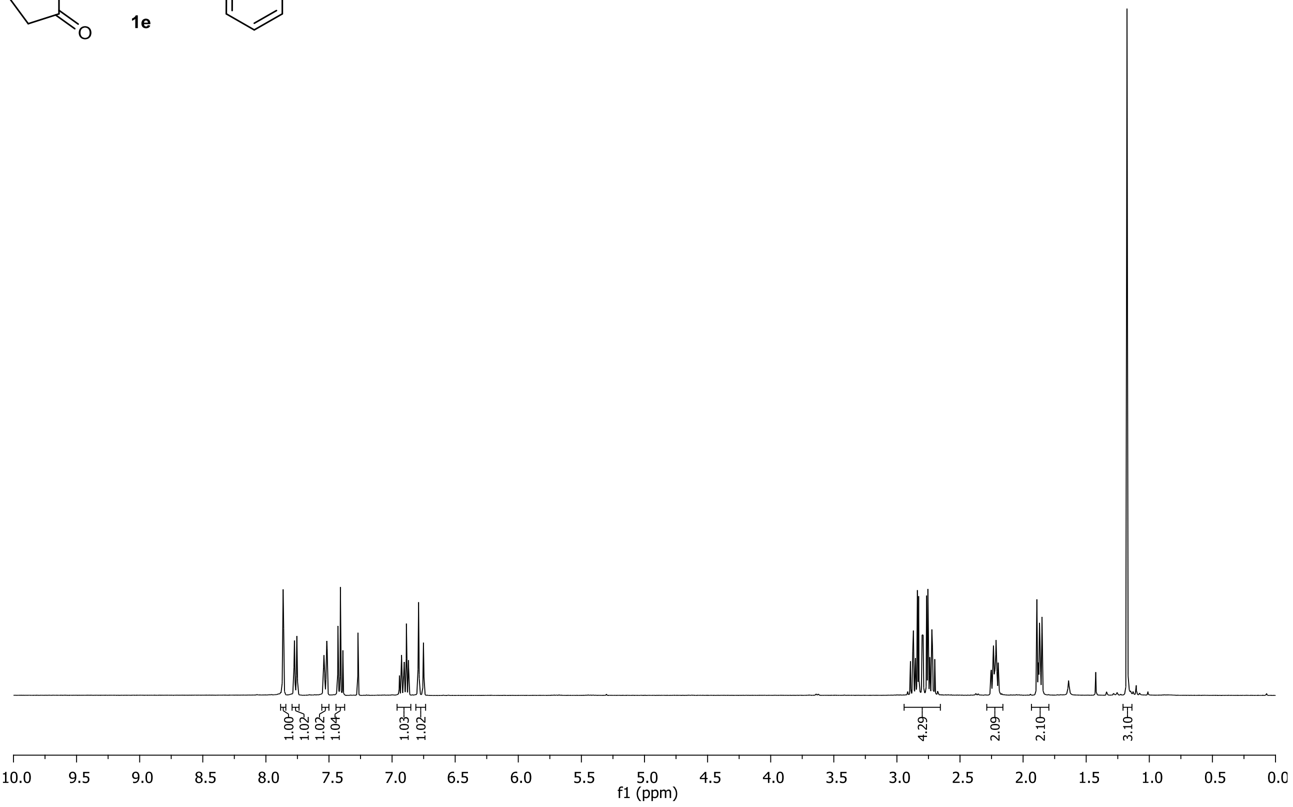
1.00
1.02
1.02
1.04
1.03
1.02
4.29
2.09
2.10
3.10
10.0 9.5 9.0 8.5 8.0 7.5 7.0 6.5 6.0 5.5 5.0 4.5 4.0 3.5 3.0 2.5 2.0 1.5 1.0 0.5 0.0
f1 (ppm)

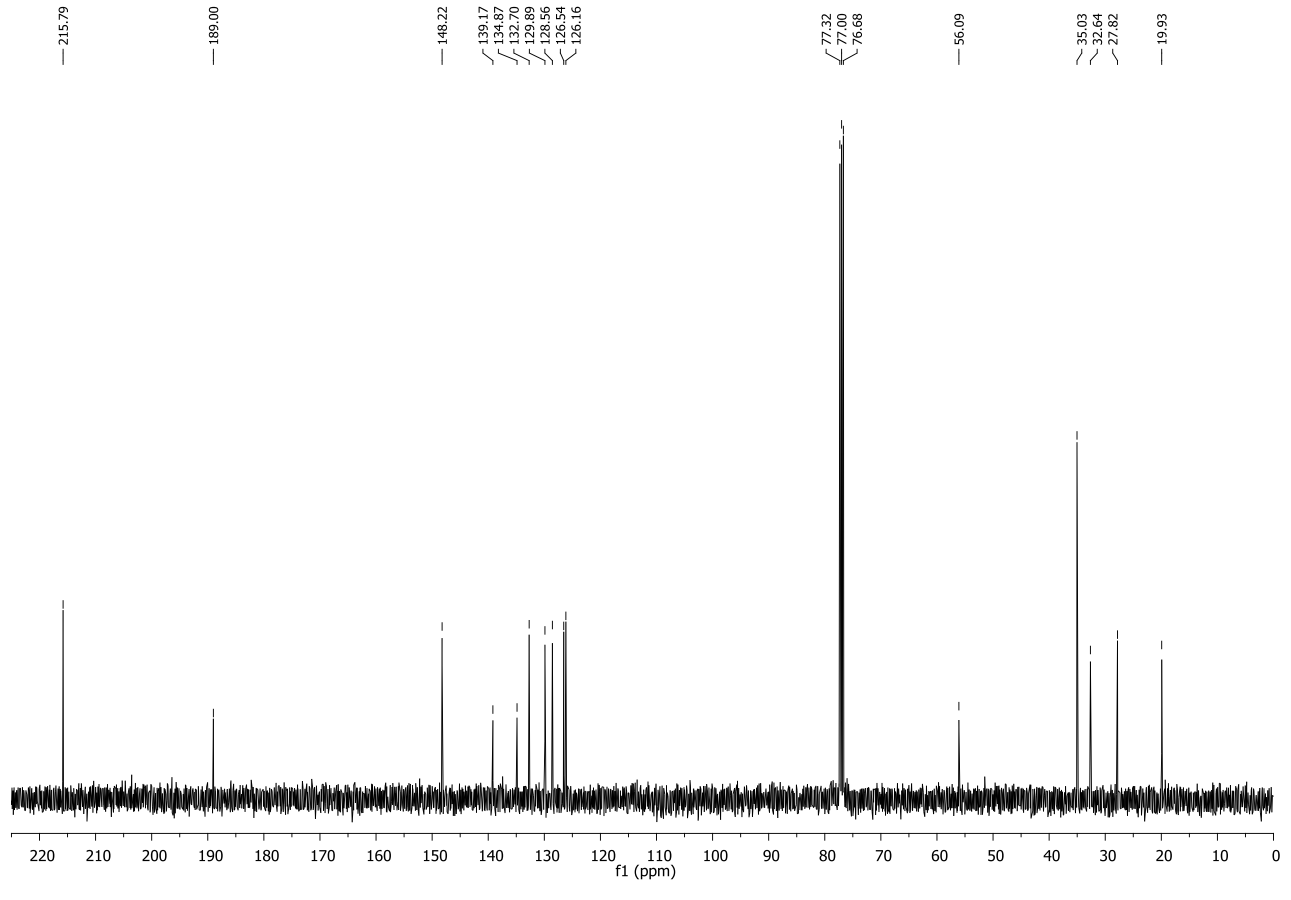
215.79
189.00
148.22
139.17
134.87
132.70
129.89
128.56
126.54
126.16
77.32
77.00
76.68
56.09
35.03
32.64
27.82
19.93
220 210 200 190 180 170 160 150 140 130 120 110 100 90 80 70 60 50 40 30 20 10 0
f1 (ppm)

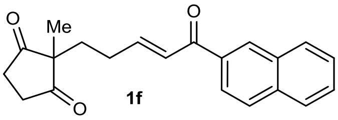

1f

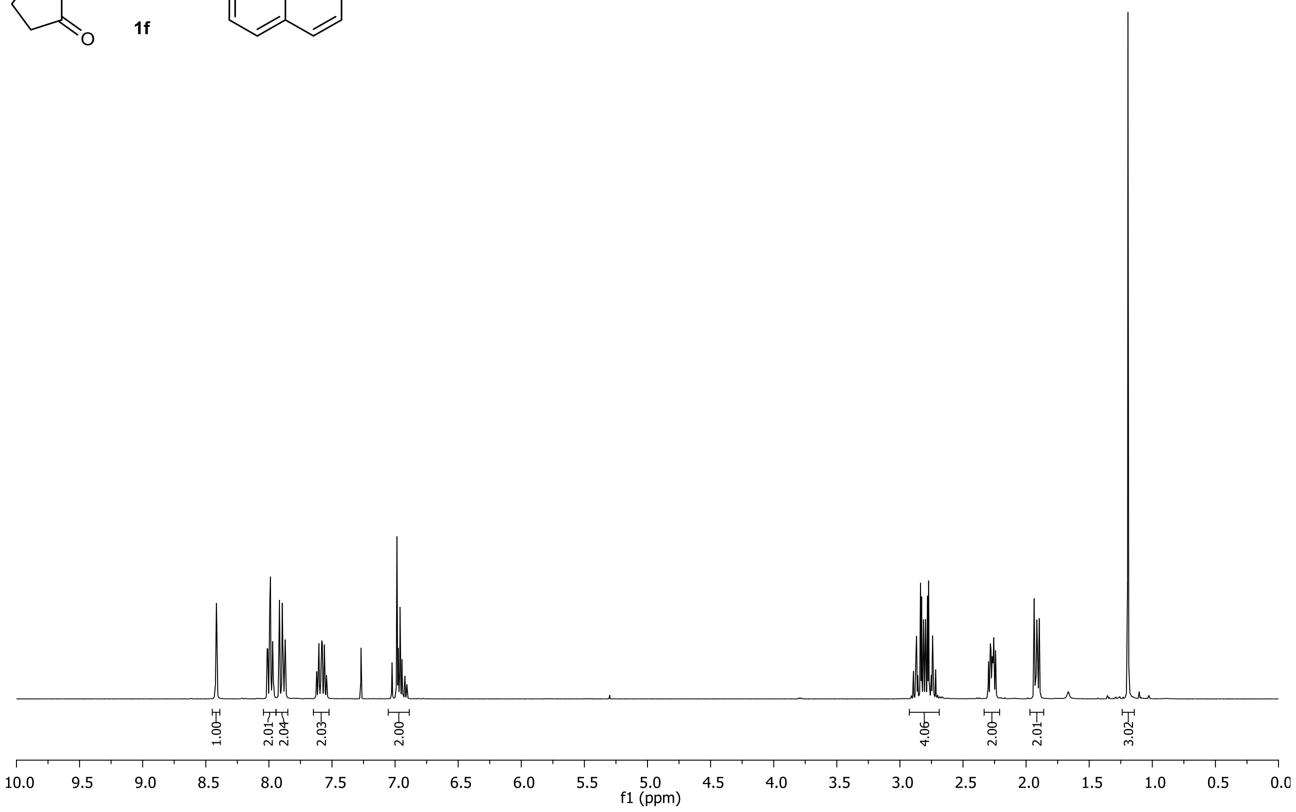

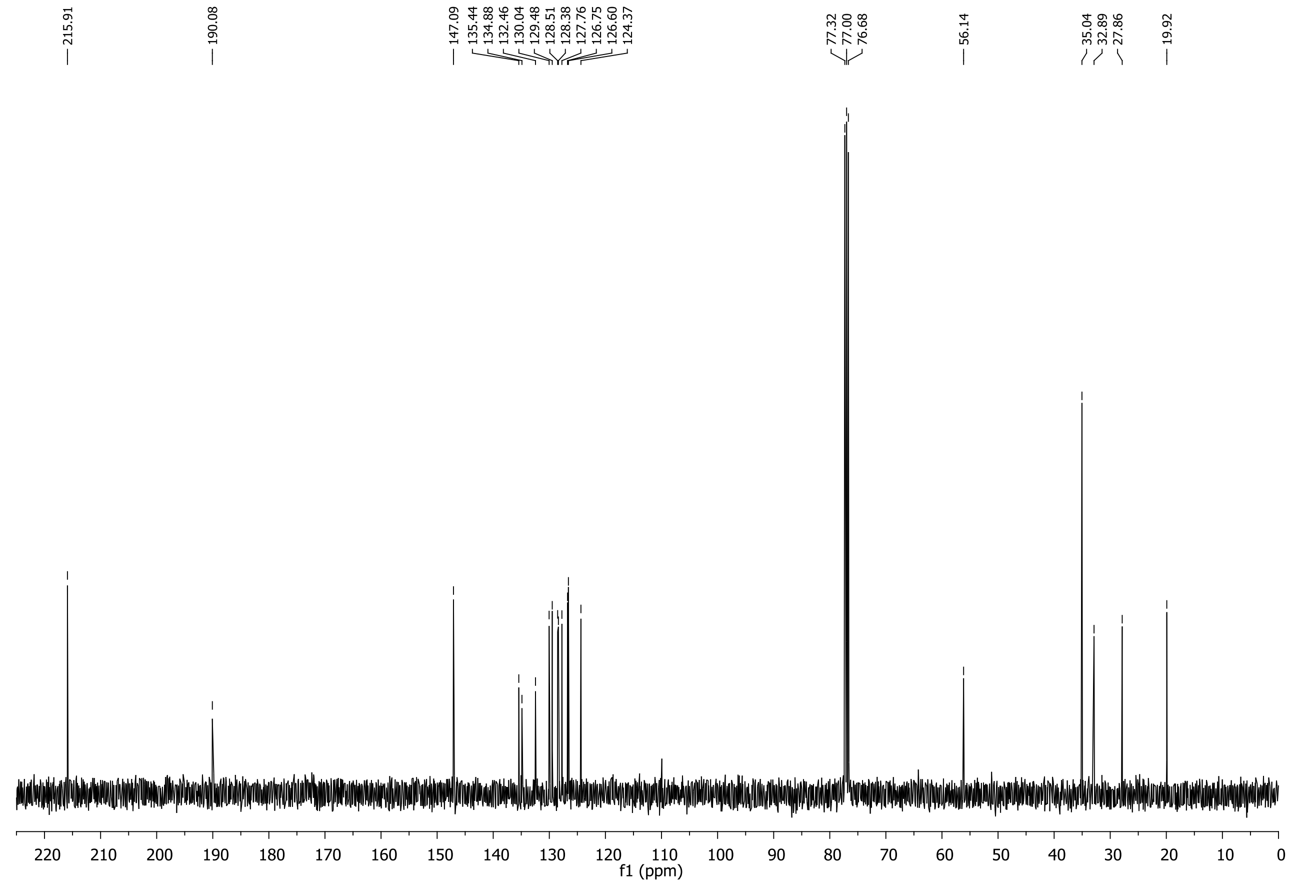

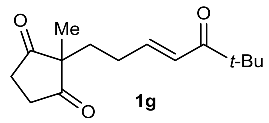
Me
O
O
O
t-Bu

1g

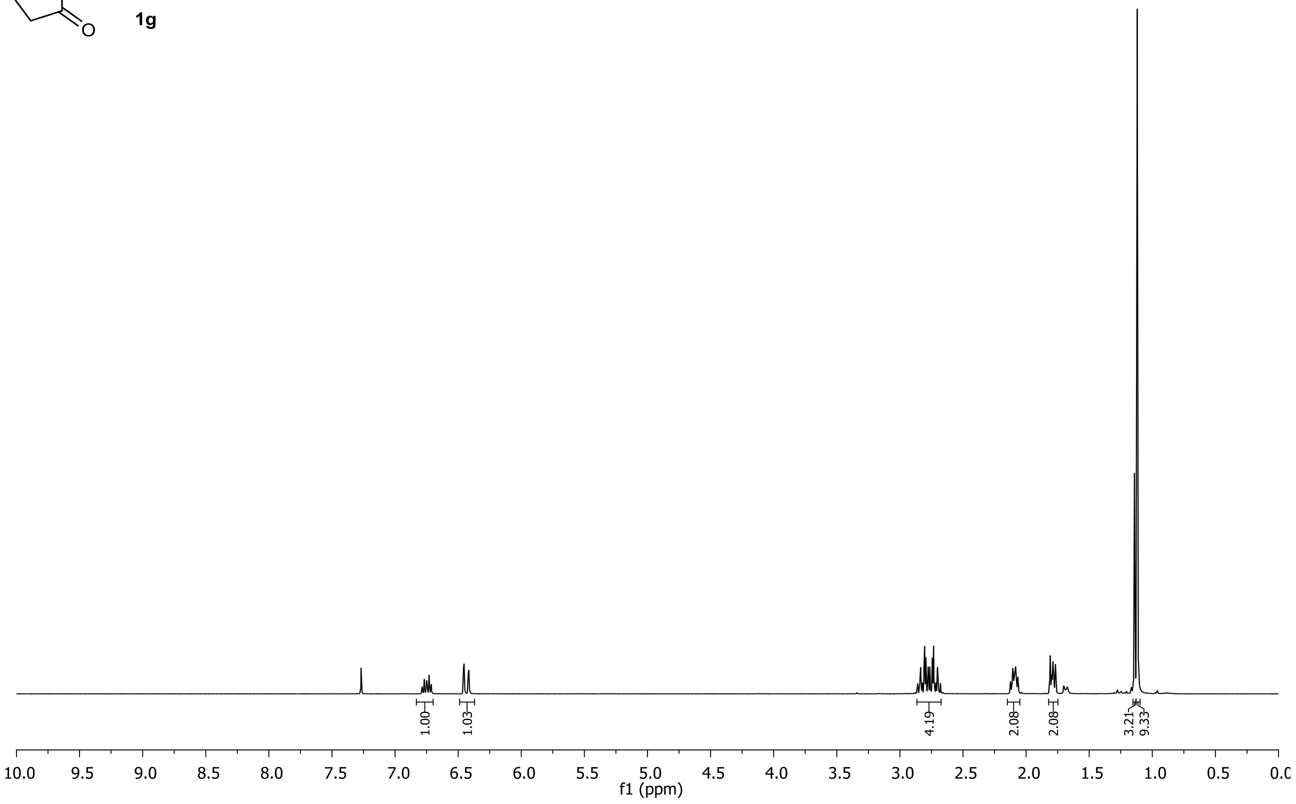
1.00
1.03
4.19
2.08
2.08
3.21
9.33
10.0 9.5 9.0 8.5 8.0 7.5 7.0 6.5 6.0 5.5 5.0 4.5 4.0 3.5 3.0 2.5 2.0 1.5 1.0 0.5 0.0
f1 (ppm)

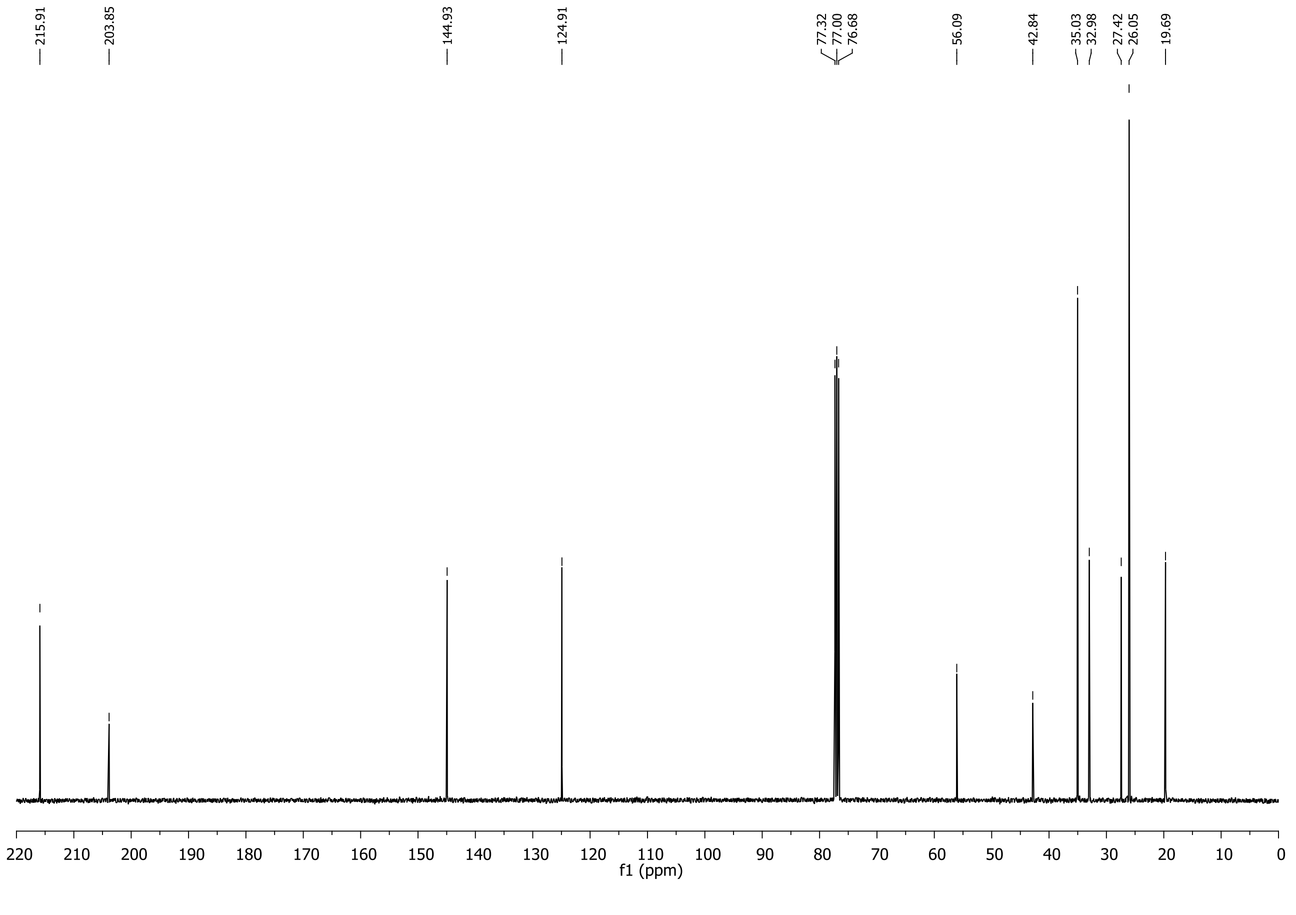
215.91
203.85
144.93
124.91
77.32
77.00
76.68
56.09
42.84
35.03
32.98
27.42
26.05
19.69
220 210 200 190 180 170 160 150 140 130 120 110 100 90 80 70 60 50 40 30 20 10 0
f1 (ppm)

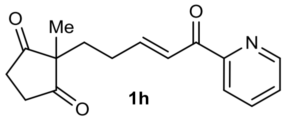
O
Me
O
N
O

1h

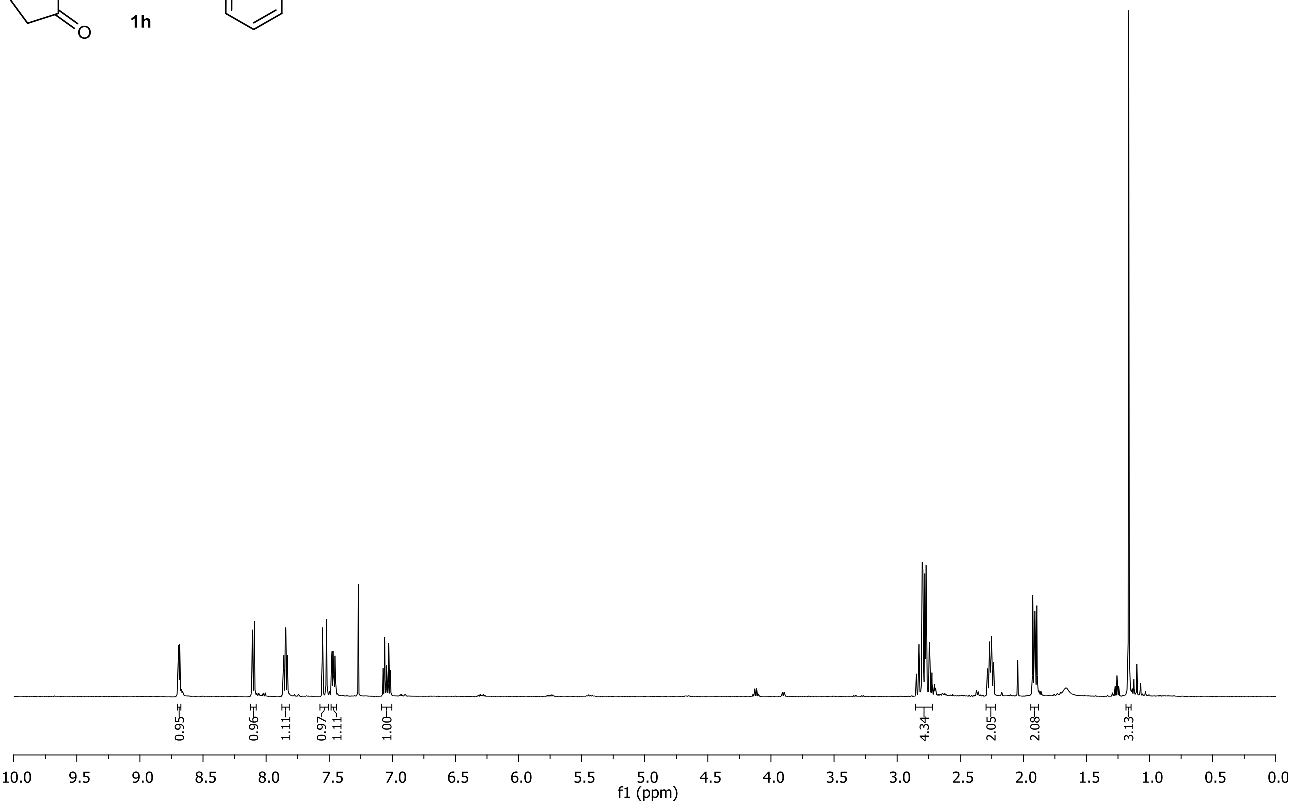
0.95
0.96
1.11
0.97
1.11
1.00
4.34
2.05
2.08
3.13
10.0 9.5 9.0 8.5 8.0 7.5 7.0 6.5 6.0 5.5 5.0 4.5 4.0 3.5 3.0 2.5 2.0 1.5 1.0 0.5 0.0
f1 (ppm)

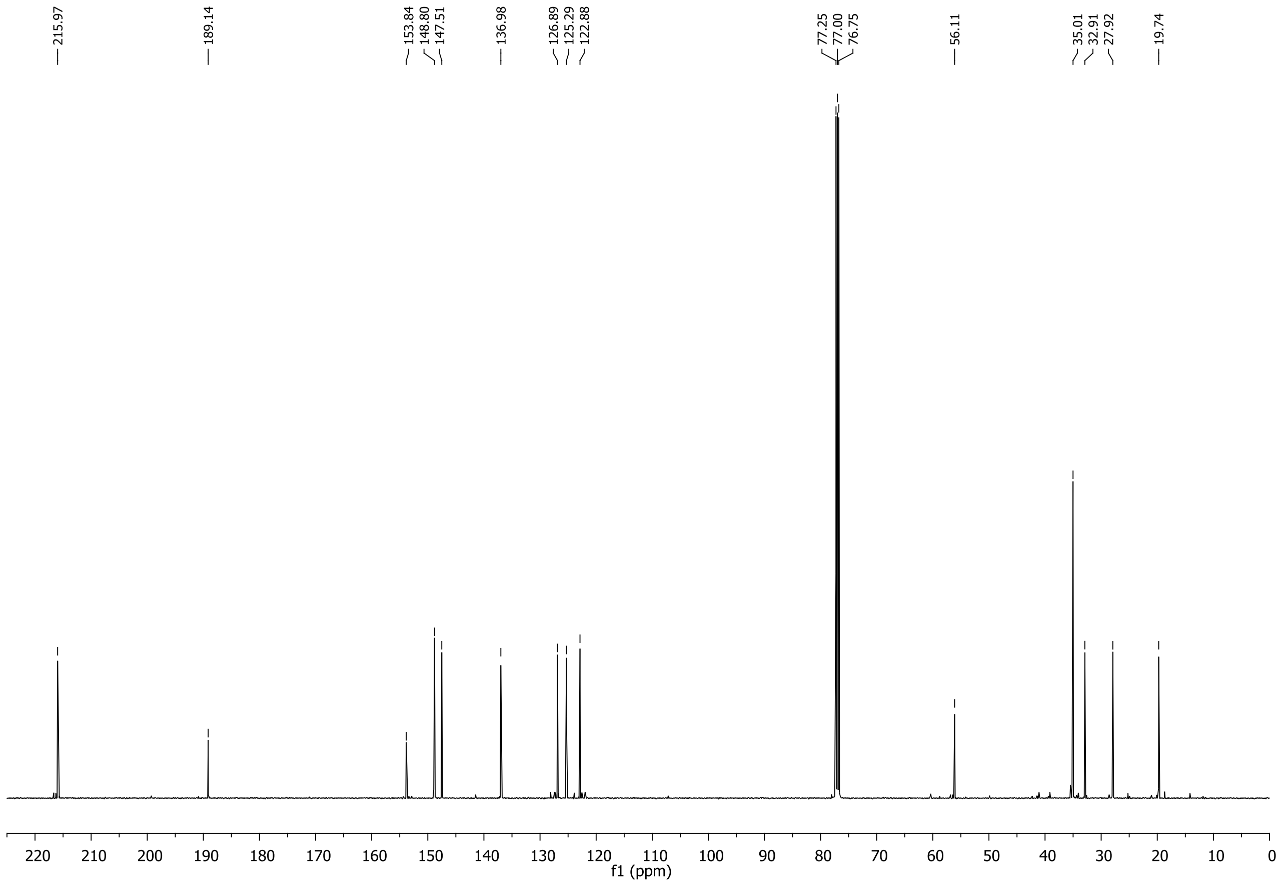
215.97
189.14
153.84
148.80
147.51
136.98
126.89
125.29
122.88
77.25
77.00
76.75
56.11
35.01
32.91
27.92
19.74
220 210 200 190 180 170 160 150 140 130 120 110 100 90 80 70 60 50 40 30 20 10 0
f1 (ppm)

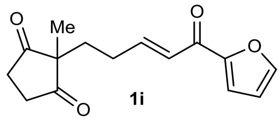
O
Me
O
O
O

1i

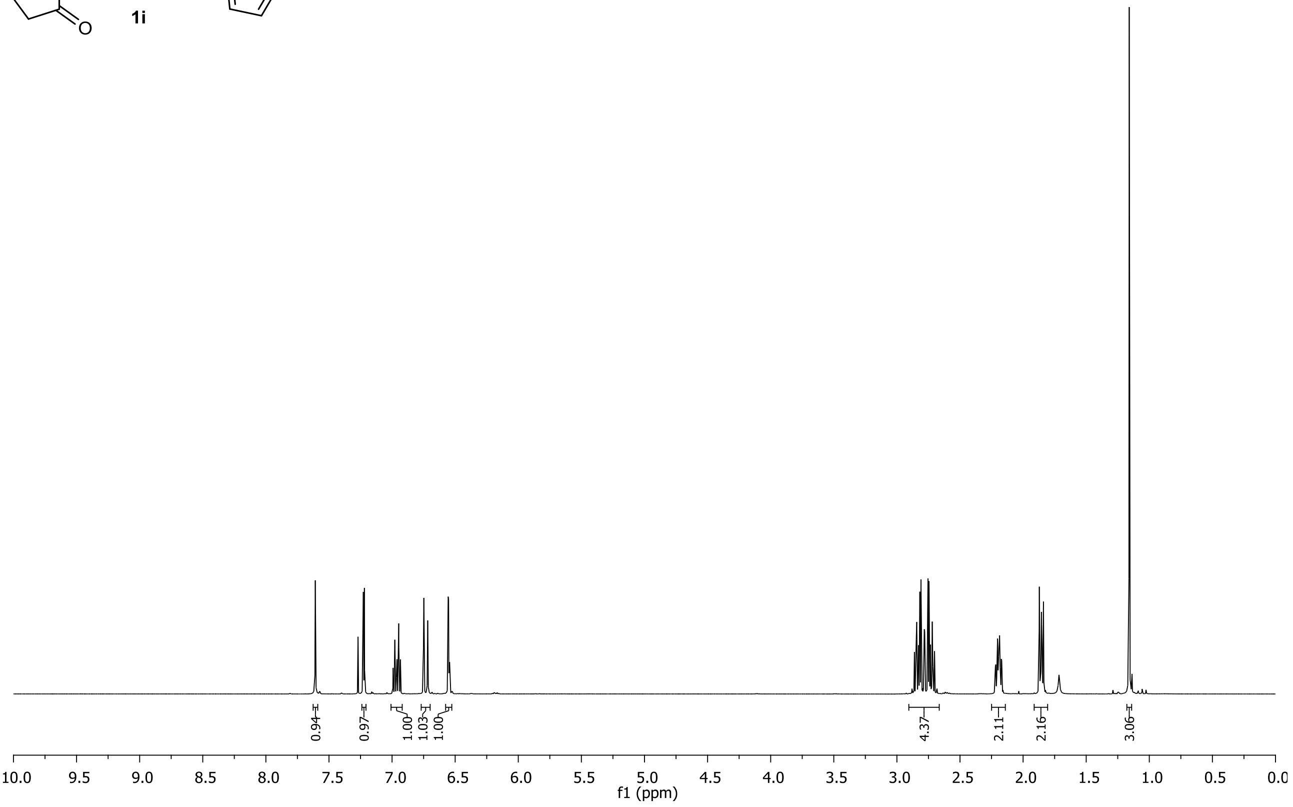
0.94
0.97
1.00
1.03
1.00
4.37
2.11
2.16
3.06
10.0 9.5 9.0 8.5 8.0 7.5 7.0 6.5 6.0 5.5 5.0 4.5 4.0 3.5 3.0 2.5 2.0 1.5 1.0 0.5 0.0
f1 (ppm)

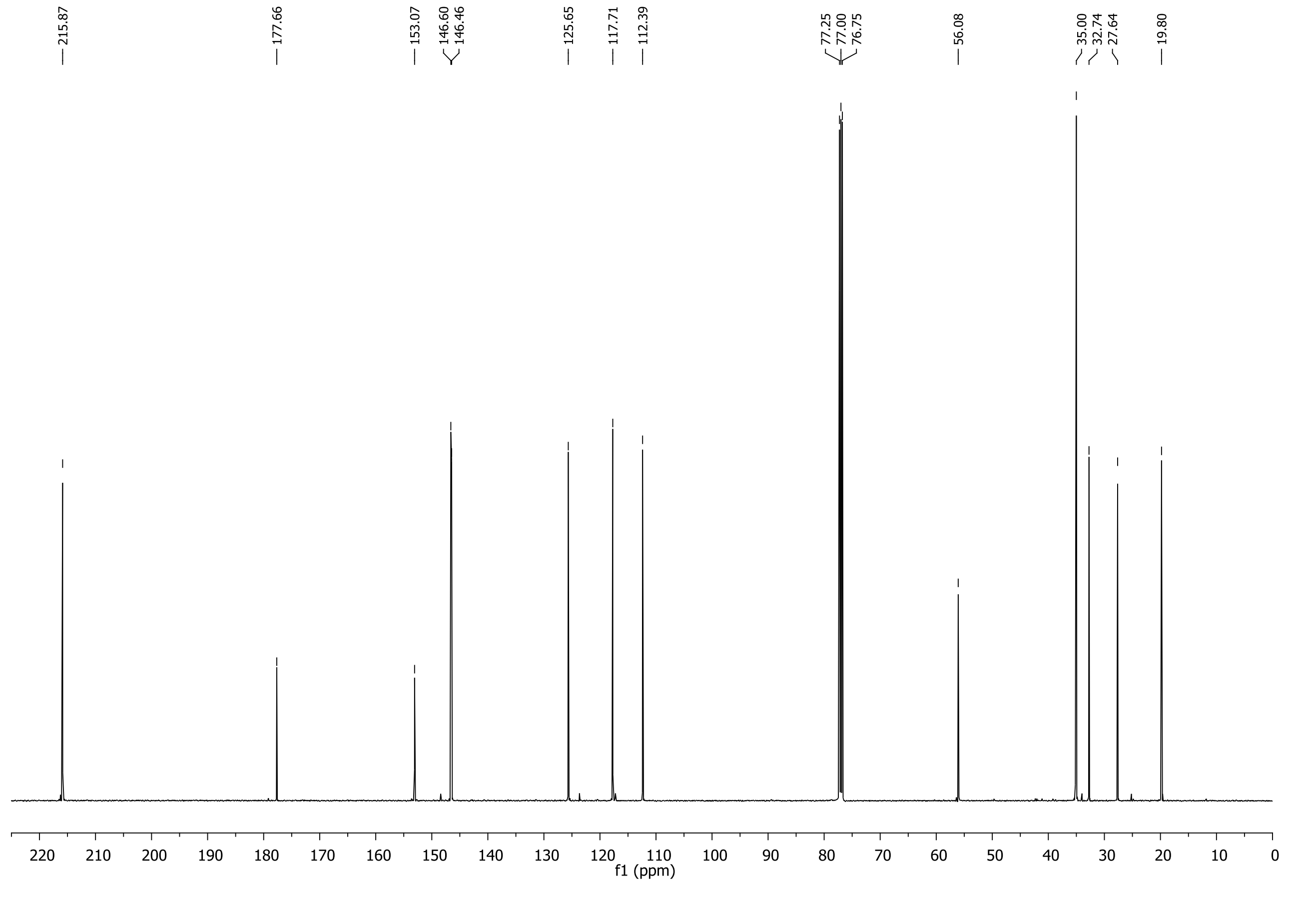
215.87
177.66
153.07
146.60
146.46
125.65
117.71
112.39
77.25
77.00
76.75
56.08
35.00
32.74
27.64
19.80
220 210 200 190 180 170 160 150 140 130 120 110 100 90 80 70 60 50 40 30 20 10 0
f1 (ppm)

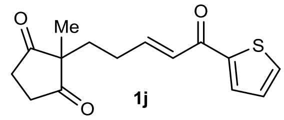
O
Me
O
S
O

1j

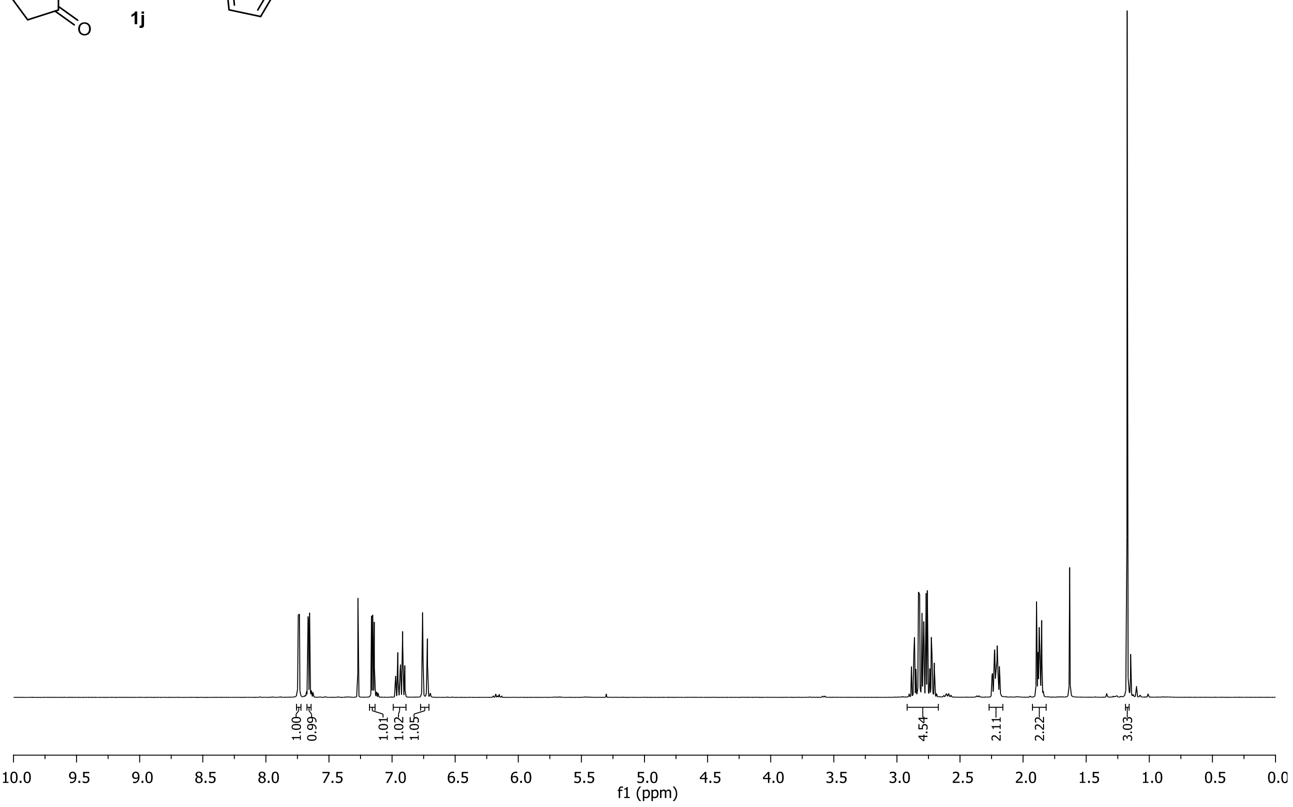
1.00
0.99
1.01
1.02
1.05
4.54
2.11
2.22
3.03
10.0 9.5 9.0 8.5 8.0 7.5 7.0 6.5 6.0 5.5 5.0 4.5 4.0 3.5 3.0 2.5 2.0 1.5 1.0 0.5 0.0
f1 (ppm)

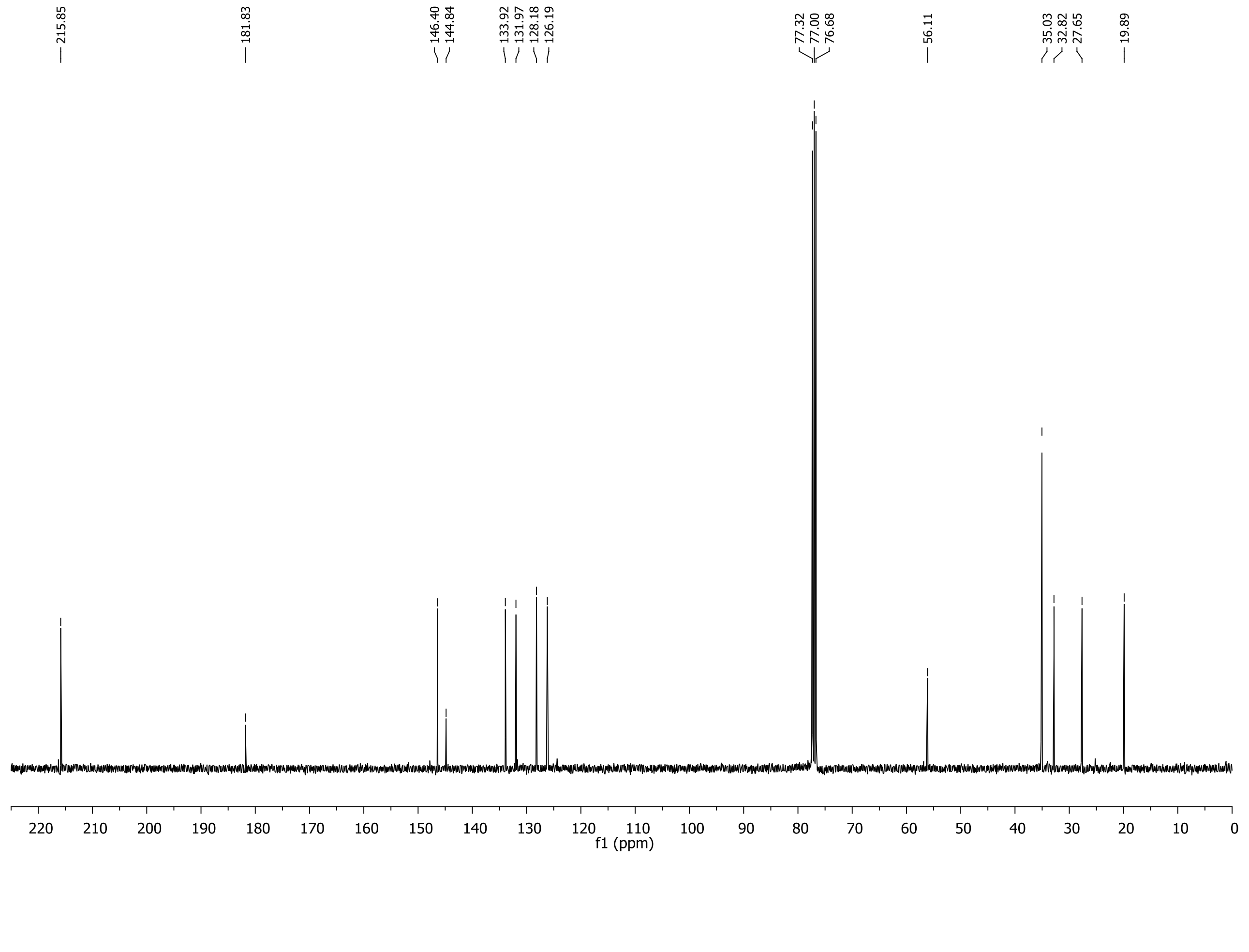
215.85
181.83
146.40
144.84
133.92
131.97
128.18
126.19
77.32
77.00
76.68
56.11
35.03
32.82
27.65
19.89
220 210 200 190 180 170 160 150 140 130 120 110 100 90 80 70 60 50 40 30 20 10 0
f1 (ppm)

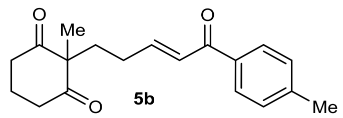
O
Me
O
O
Me

5b

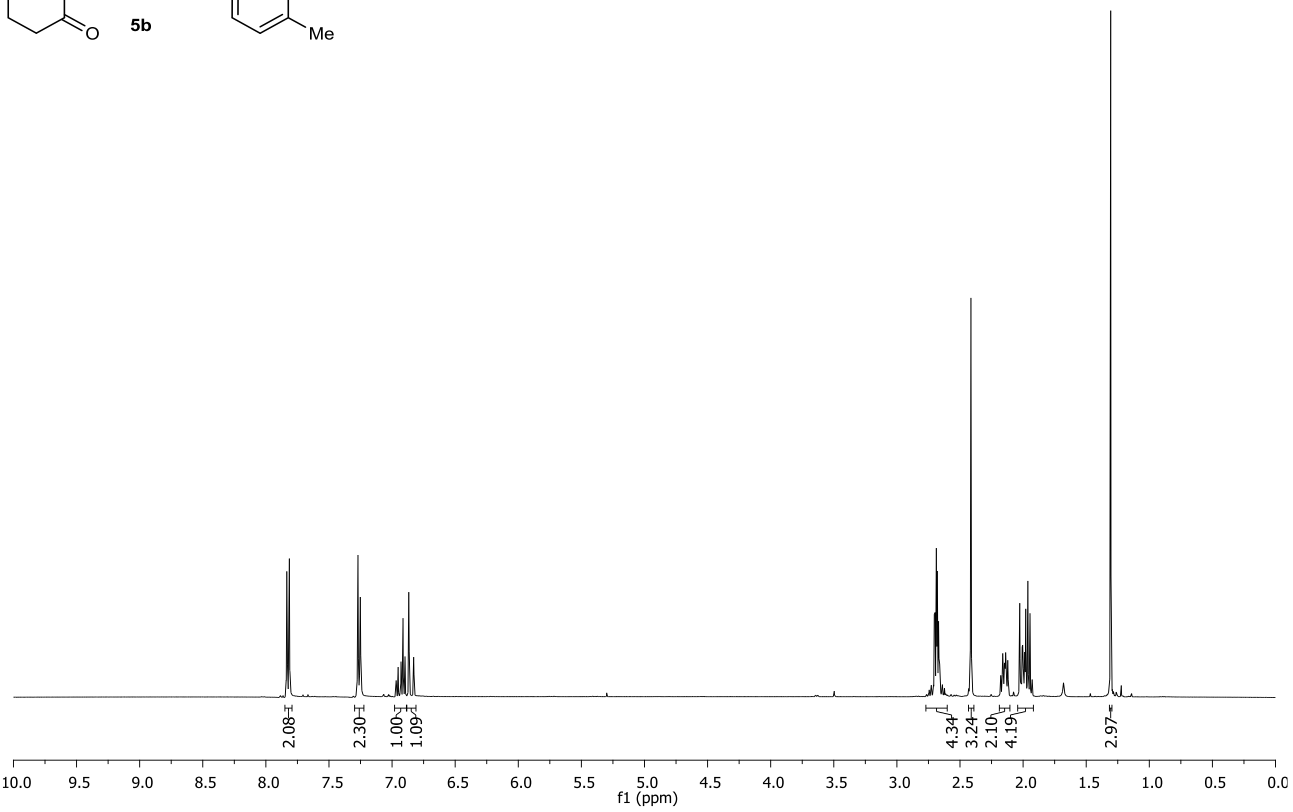
2.08
2.30
1.00
1.09
4.34
3.24
2.10
4.19
2.97
10.0 9.5 9.0 8.5 8.0 7.5 7.0 6.5 6.0 5.5 5.0 4.5 4.0 3.5 3.0 2.5 2.0 1.5 1.0 0.5 0.0
f1 (ppm)

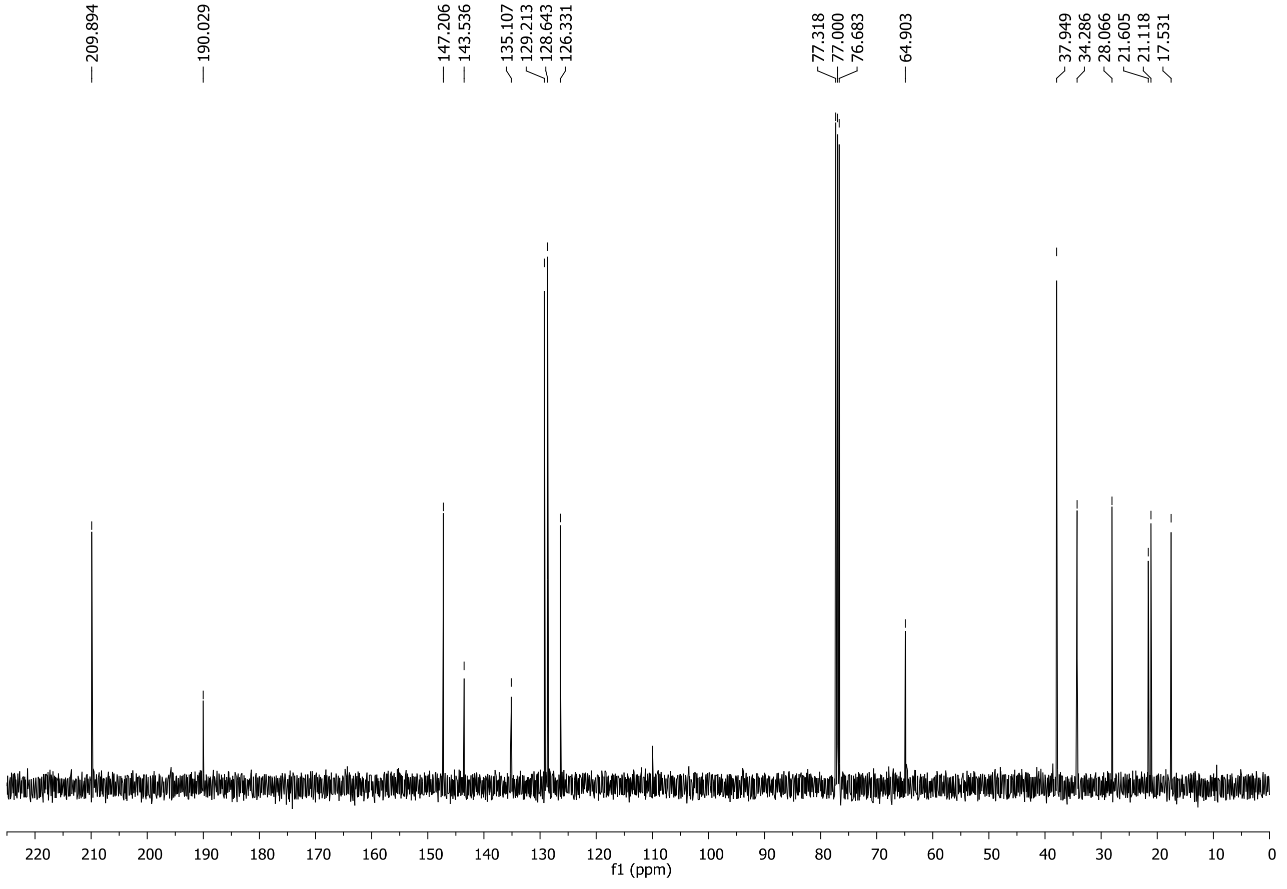
209.894
190.029
147.206
143.536
135.107
129.213
128.643
126.331
77.318
77.000
76.683
64.903
37.949
34.286
28.066
21.605
21.118
17.531
220 210 200 190 180 170 160 150 140 130 120 110 100 90 80 70 60 50 40 30 20 10 0
f1 (ppm)

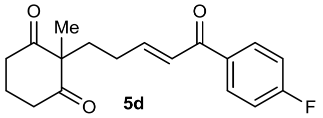
Me
O
O
O
F

5d

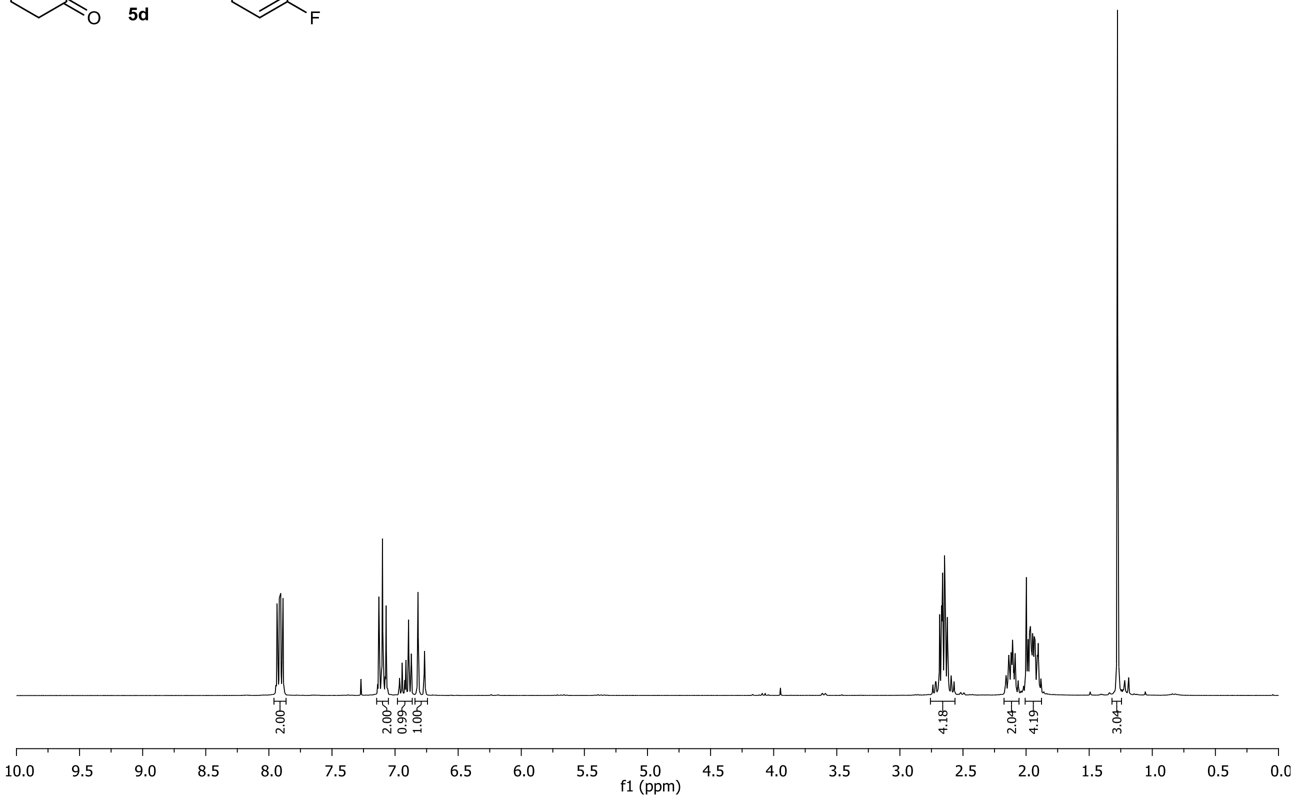
2.00
2.00
0.99
1.00
4.18
2.04
4.19
3.04
10.0 9.5 9.0 8.5 8.0 7.5 7.0 6.5 6.0 5.5 5.0 4.5 4.0 3.5 3.0 2.5 2.0 1.5 1.0 0.5 0.0
f1 (ppm)

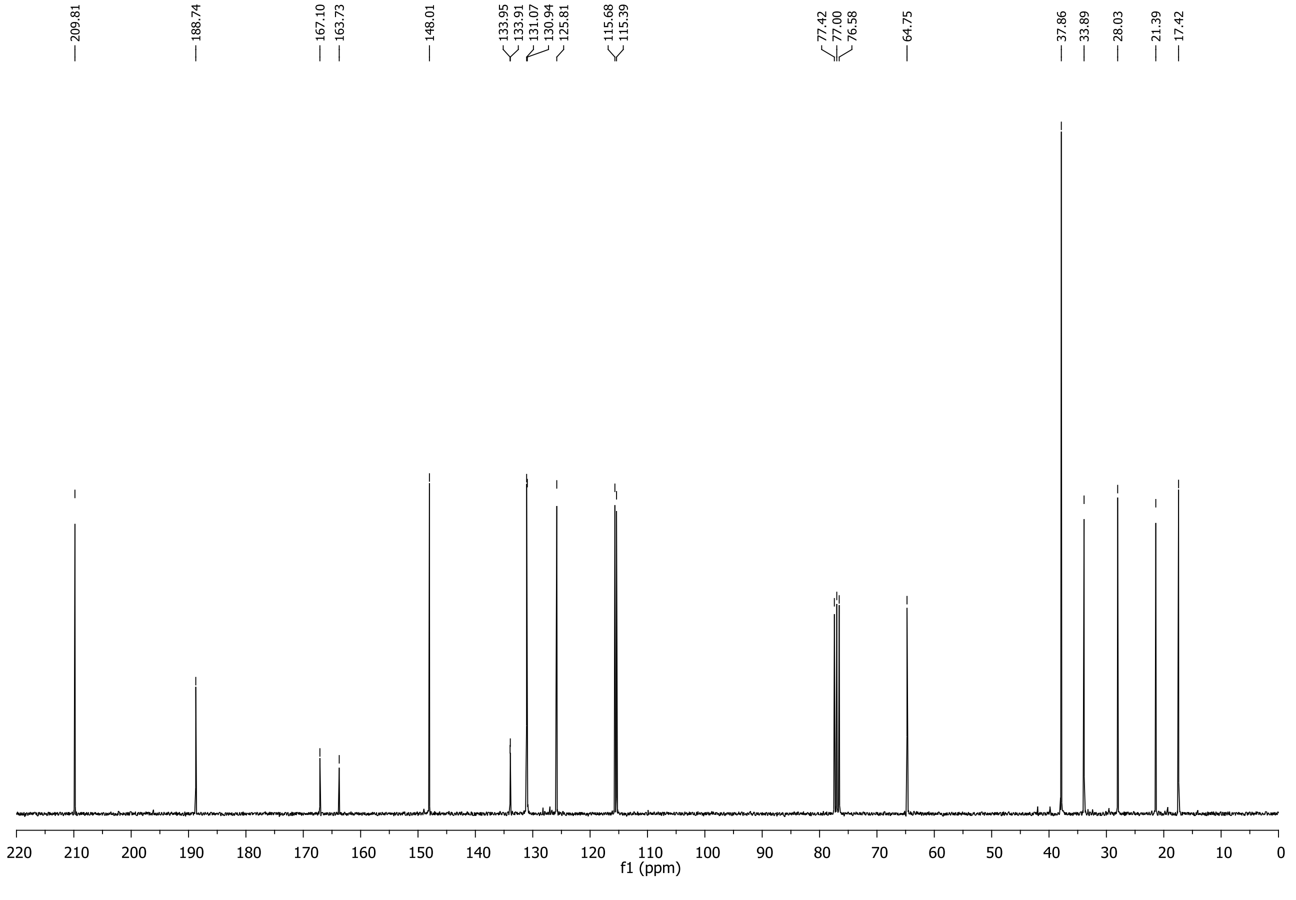
209.81
188.74
167.10
163.73
148.01
133.95
133.91
131.07
130.94
125.81
115.68
115.39
77.42
77.00
76.58
64.75
37.86
33.89
28.03
21.39
17.42
220 210 200 190 180 170 160 150 140 130 120 110 100 90 80 70 60 50 40 30 20 10 0
f1 (ppm)

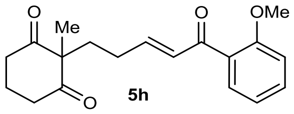
O
Me
O
OMe
O
5h

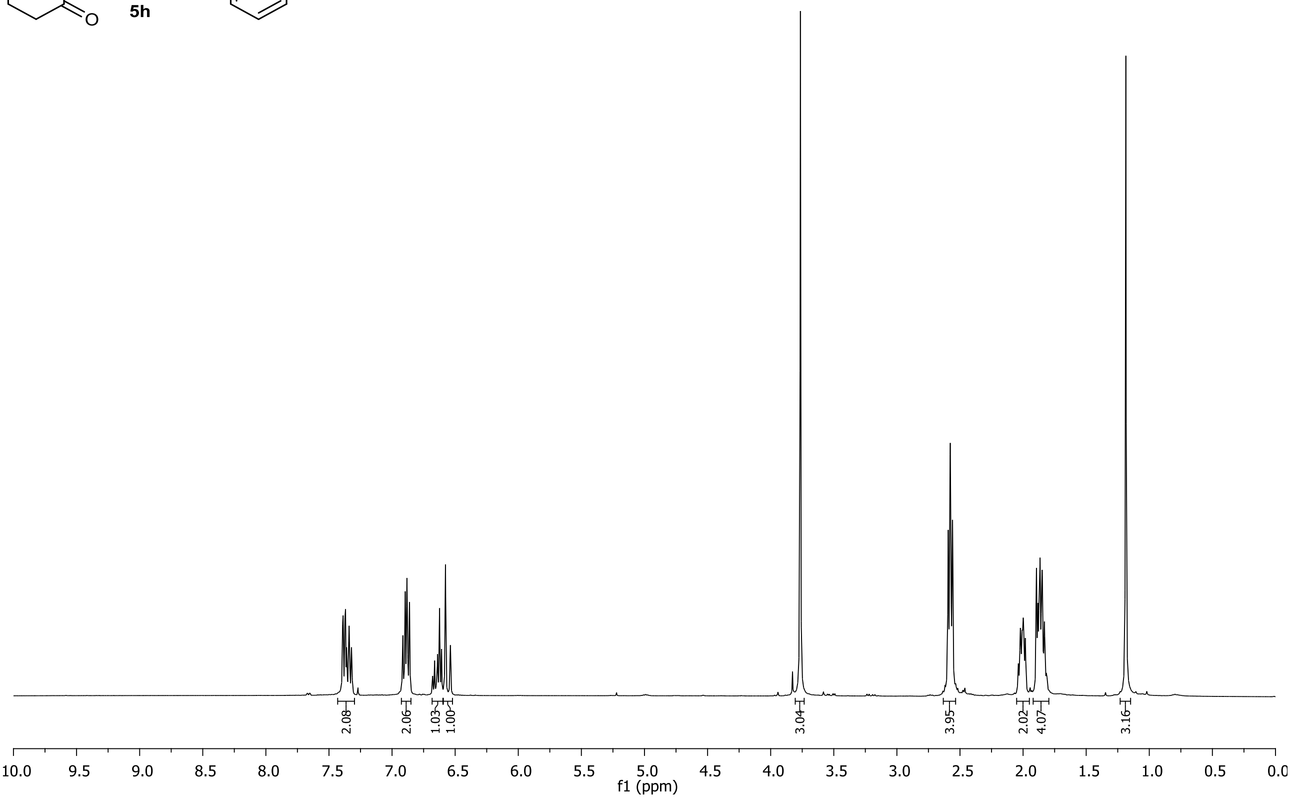
2.08
2.06
1.03
1.00
3.04
3.95
2.02
4.07
3.16
10.0 9.5 9.0 8.5 8.0 7.5 7.0 6.5 6.0 5.5 5.0 4.5 4.0 3.5 3.0 2.5 2.0 1.5 1.0 0.5 0.0
f1 (ppm)

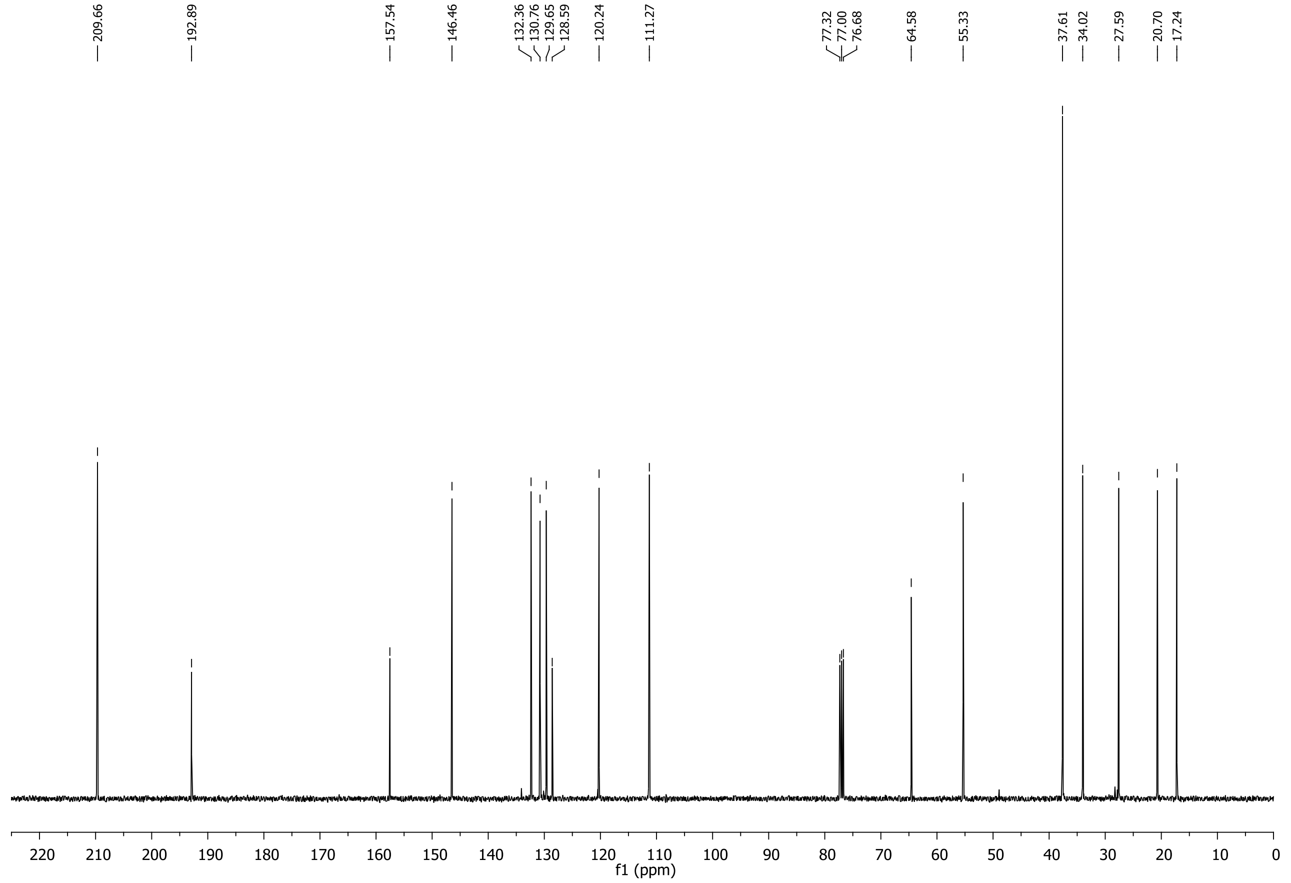
209.66
192.89
157.54
146.46
132.36
130.76
129.65
128.59
120.24
111.27
77.32
77.00
76.68
64.58
55.33
37.61
34.02
27.59
20.70
17.24
220 210 200 190 180 170 160 150 140 130 120 110 100 90 80 70 60 50 40 30 20 10 0
f1 (ppm)

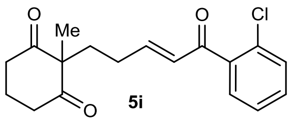
O
Me
O
Cl
O

5i

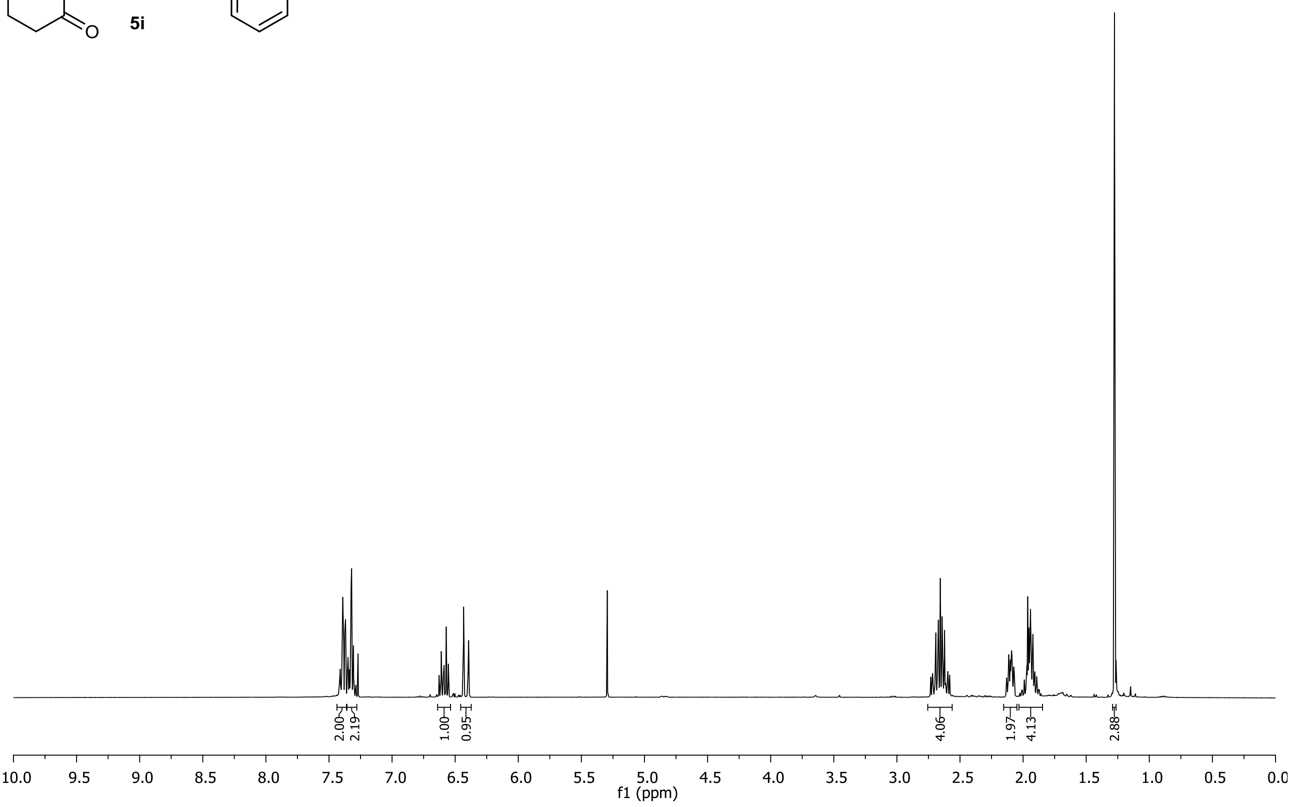
2.00
2.19
1.00
0.95
4.06
1.97
4.13
2.88
10.0 9.5 9.0 8.5 8.0 7.5 7.0 6.5 6.0 5.5 5.0 4.5 4.0 3.5 3.0 2.5 2.0 1.5 1.0 0.5 0.0
f1 (ppm)

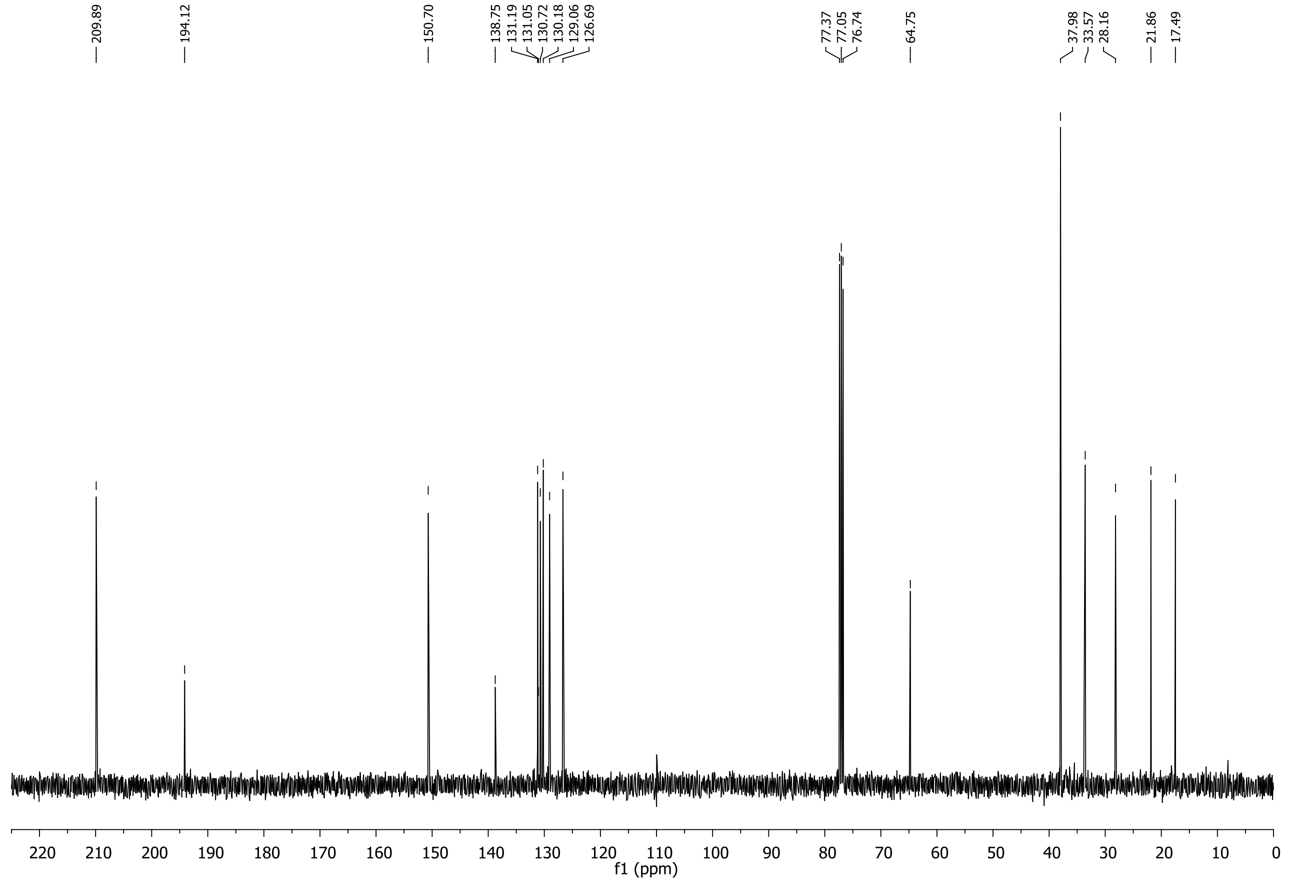
209.89
194.12
150.70
138.75
131.19
131.05
130.72
130.18
129.06
126.69
77.37
77.05
76.74
64.75
37.98
33.57
28.16
21.86
17.49
220 210 200 190 180 170 160 150 140 130 120 110 100 90 80 70 60 50 40 30 20 10 0
f1 (ppm)

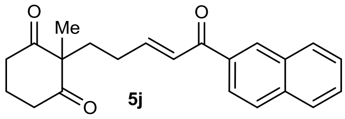
O
Me
O
O

5j

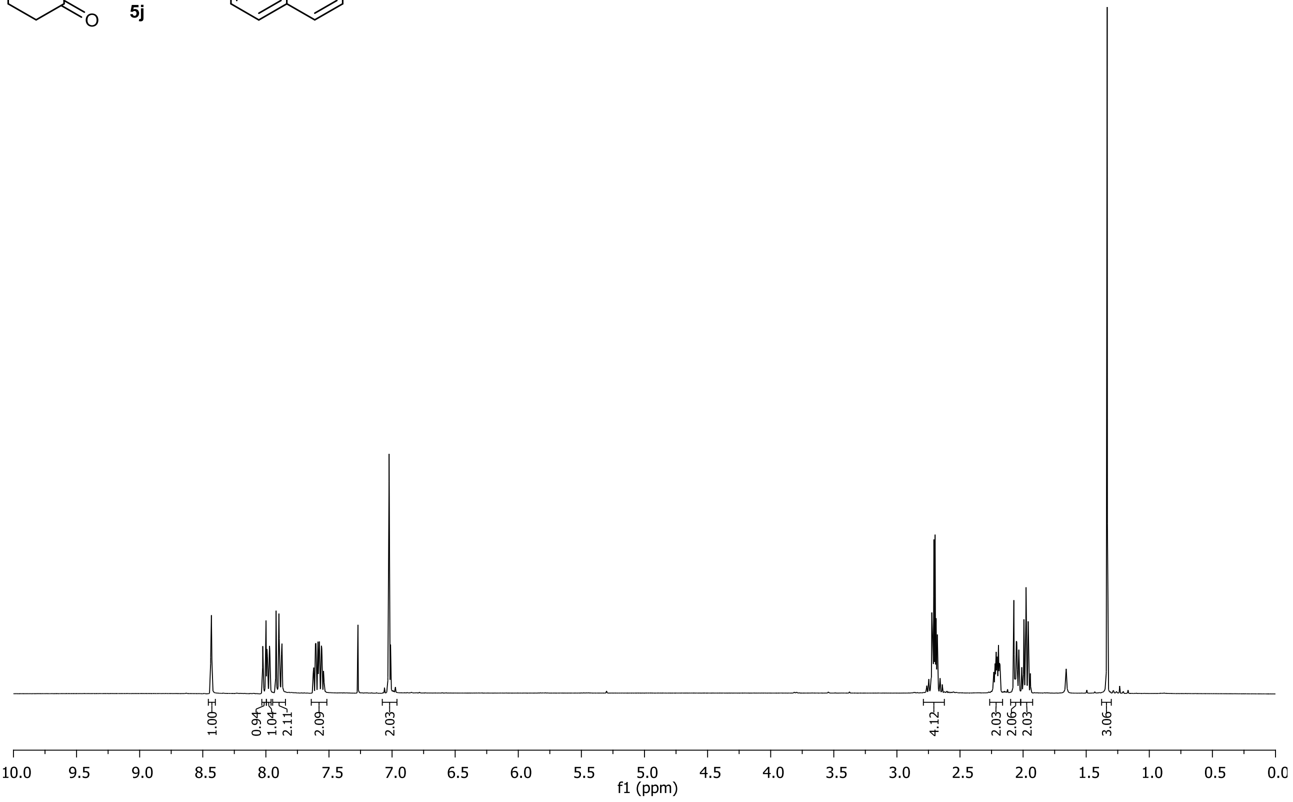
1.00
0.94
1.04
2.11
2.09
2.03
4.12
2.03
2.06
2.03
3.06
10.0 9.5 9.0 8.5 8.0 7.5 7.0 6.5 6.0 5.5 5.0 4.5 4.0 3.5 3.0 2.5 2.0 1.5 1.0 0.5 0.0
f1 (ppm)

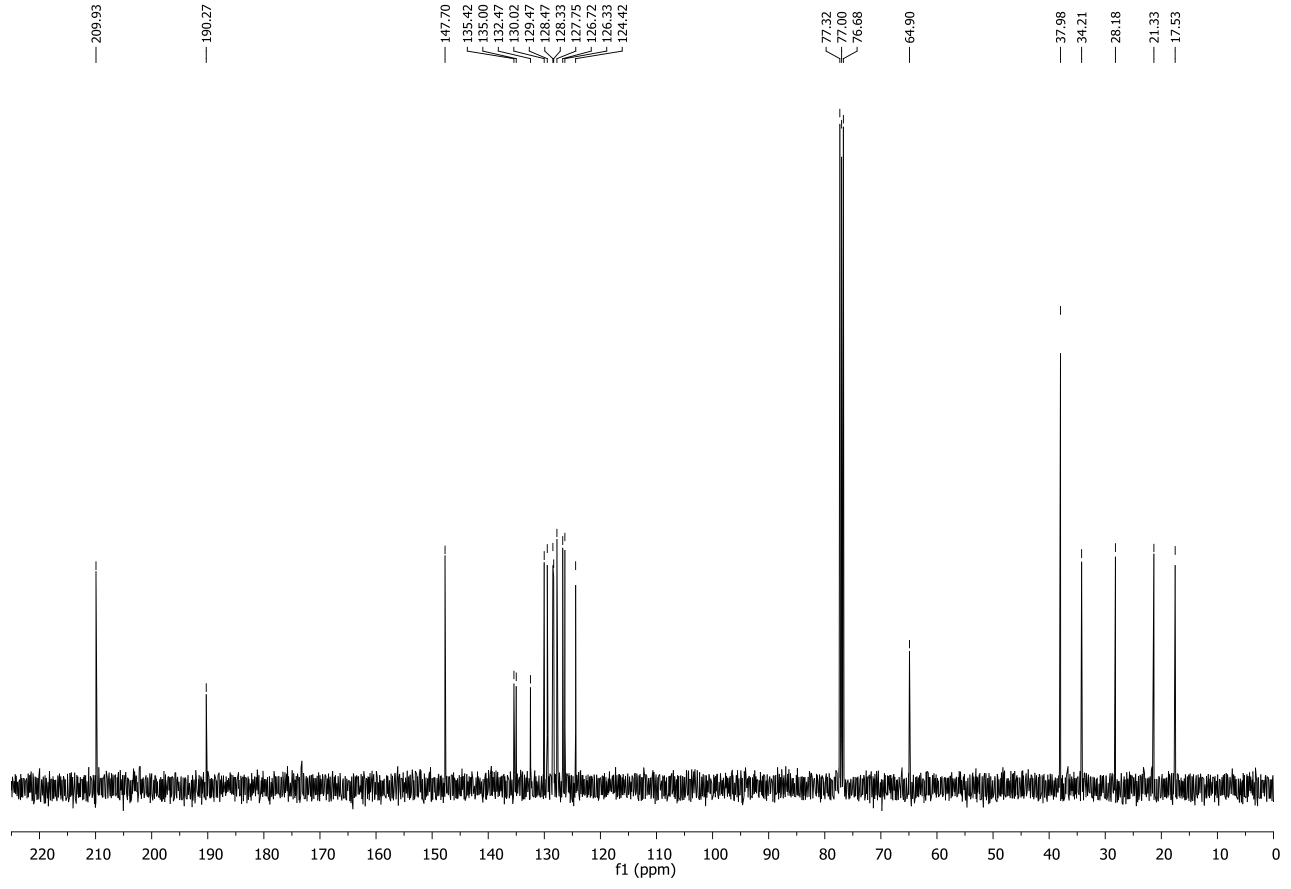
209.93
190.27
147.70
135.42
135.00
132.47
130.02
129.47
128.47
128.33
127.75
126.72
126.33
124.42
77.32
77.00
76.68
64.90
37.98
34.21
28.18
21.33
17.53
220 210 200 190 180 170 160 150 140 130 120 110 100 90 80 70 60 50 40 30 20 10 0
f1 (ppm)

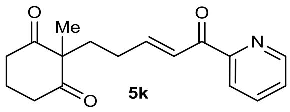
Me
N
5k

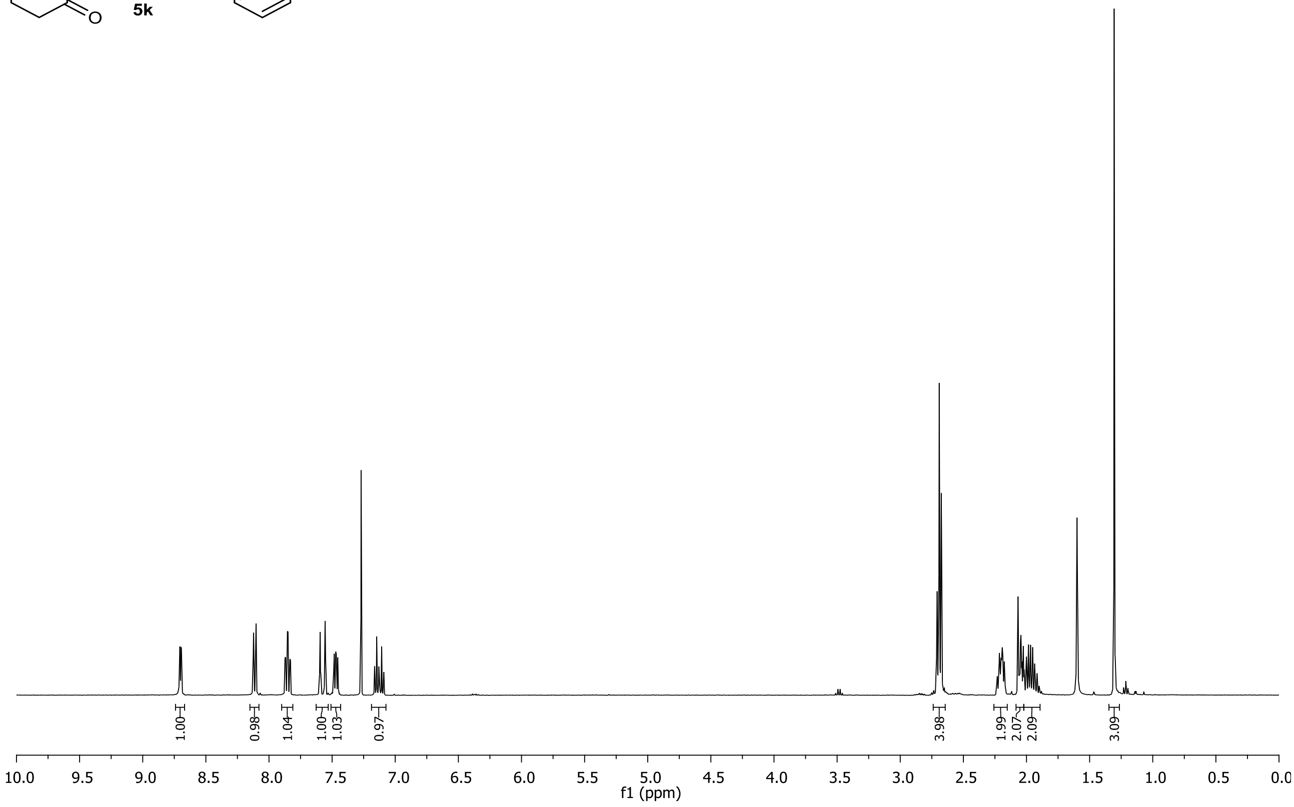
1.00
0.98
1.04
1.00
1.03
0.97
3.98
1.99
2.07
2.09
3.09
10.0 9.5 9.0 8.5 8.0 7.5 7.0 6.5 6.0 5.5 5.0 4.5 4.0 3.5 3.0 2.5 2.0 1.5 1.0 0.5 0.0
f1 (ppm)

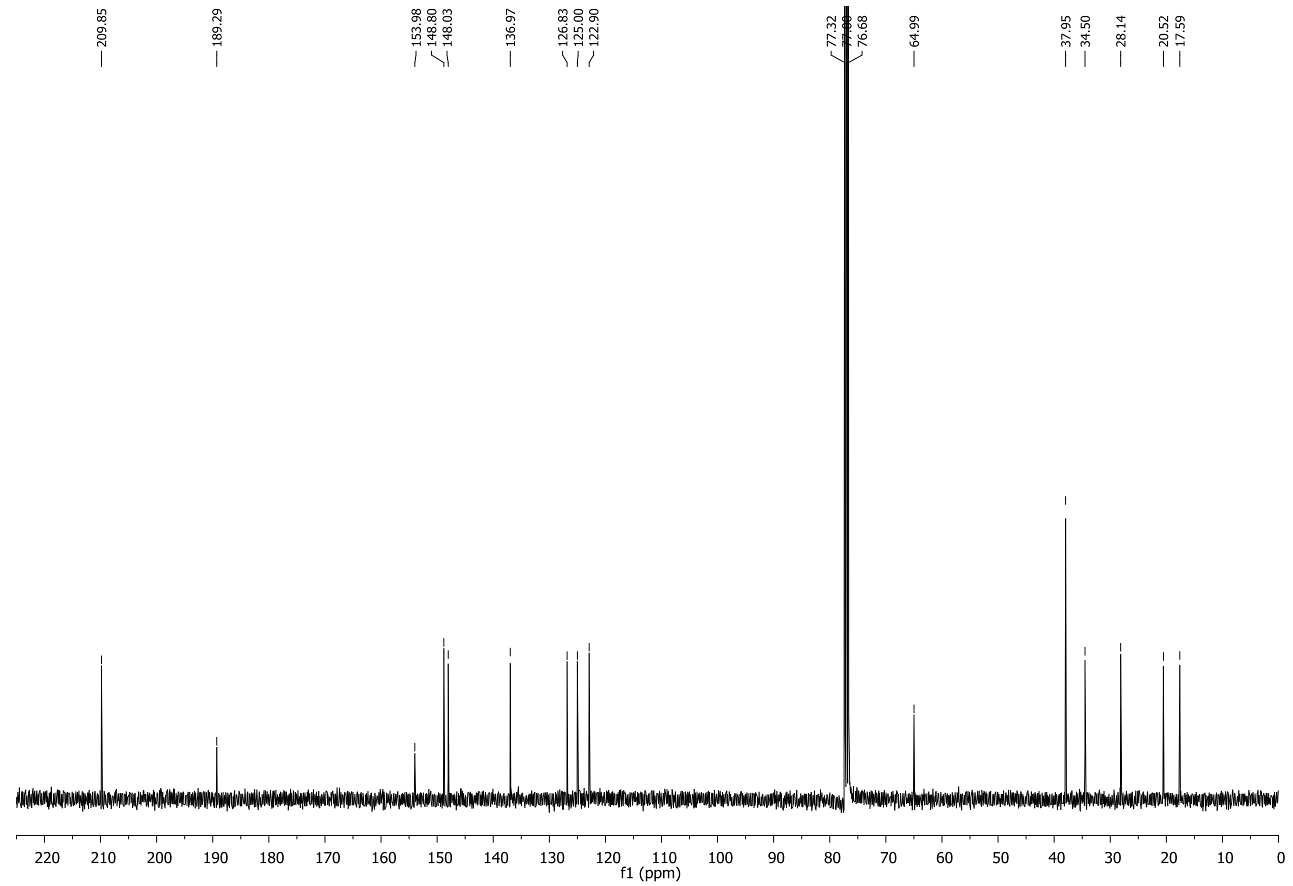
209.85
189.29
153.98
148.80
148.03
136.97
126.83
125.00
122.90
77.32
76.68
64.99
37.95
34.50
28.14
20.52
17.59
220 210 200 190 180 170 160 150 140 130 120 110 100 90 80 70 60 50 40 30 20 10 0
f1 (ppm)

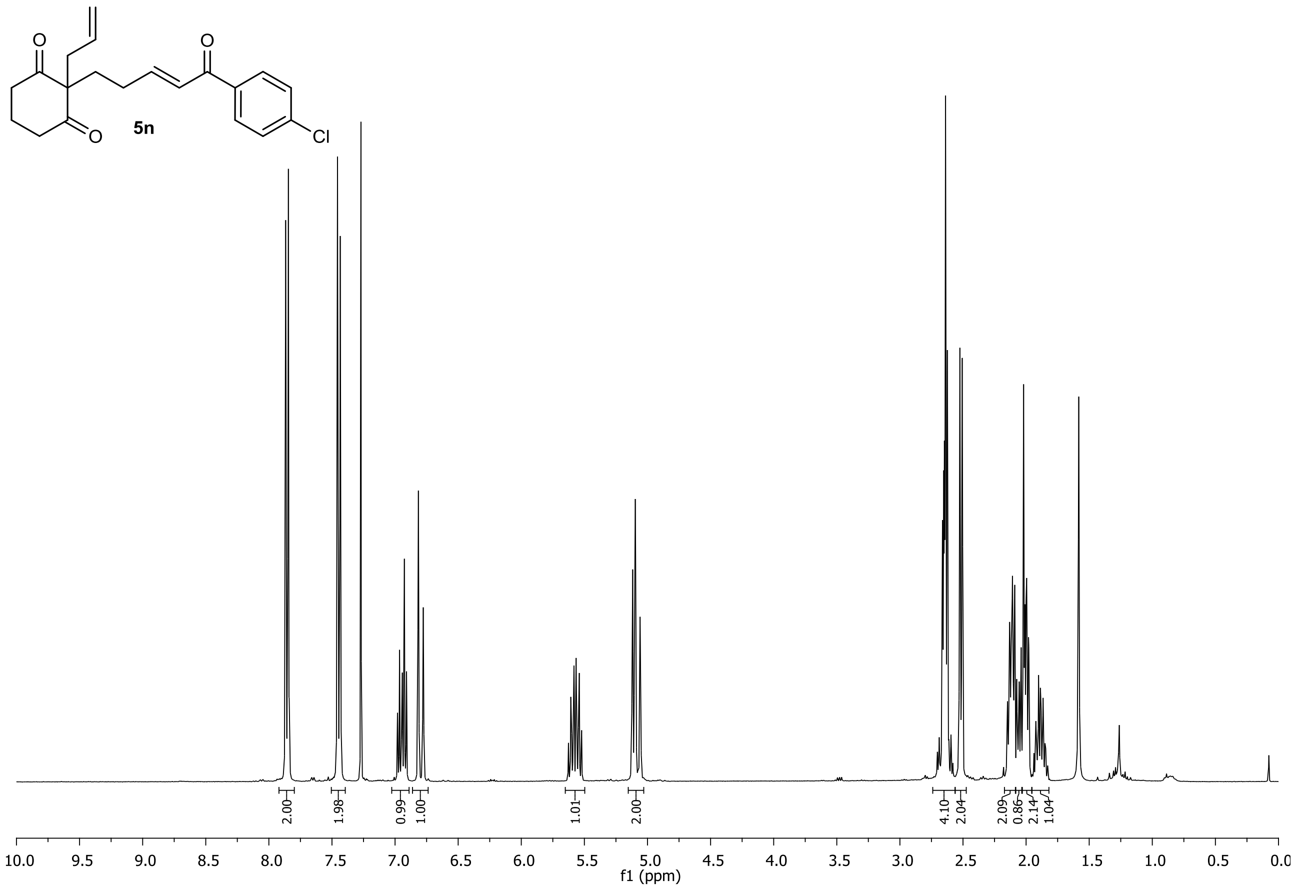
O
O
O
5n
Cl
2.00
1.98
0.99
1.00
1.01
2.00
4.10
2.04
2.09
0.86
2.14
1.04
10.0 9.5 9.0 8.5 8.0 7.5 7.0 6.5 6.0 5.5 5.0 4.5 4.0 3.5 3.0 2.5 2.0 1.5 1.0 0.5 0.0
f1 (ppm)

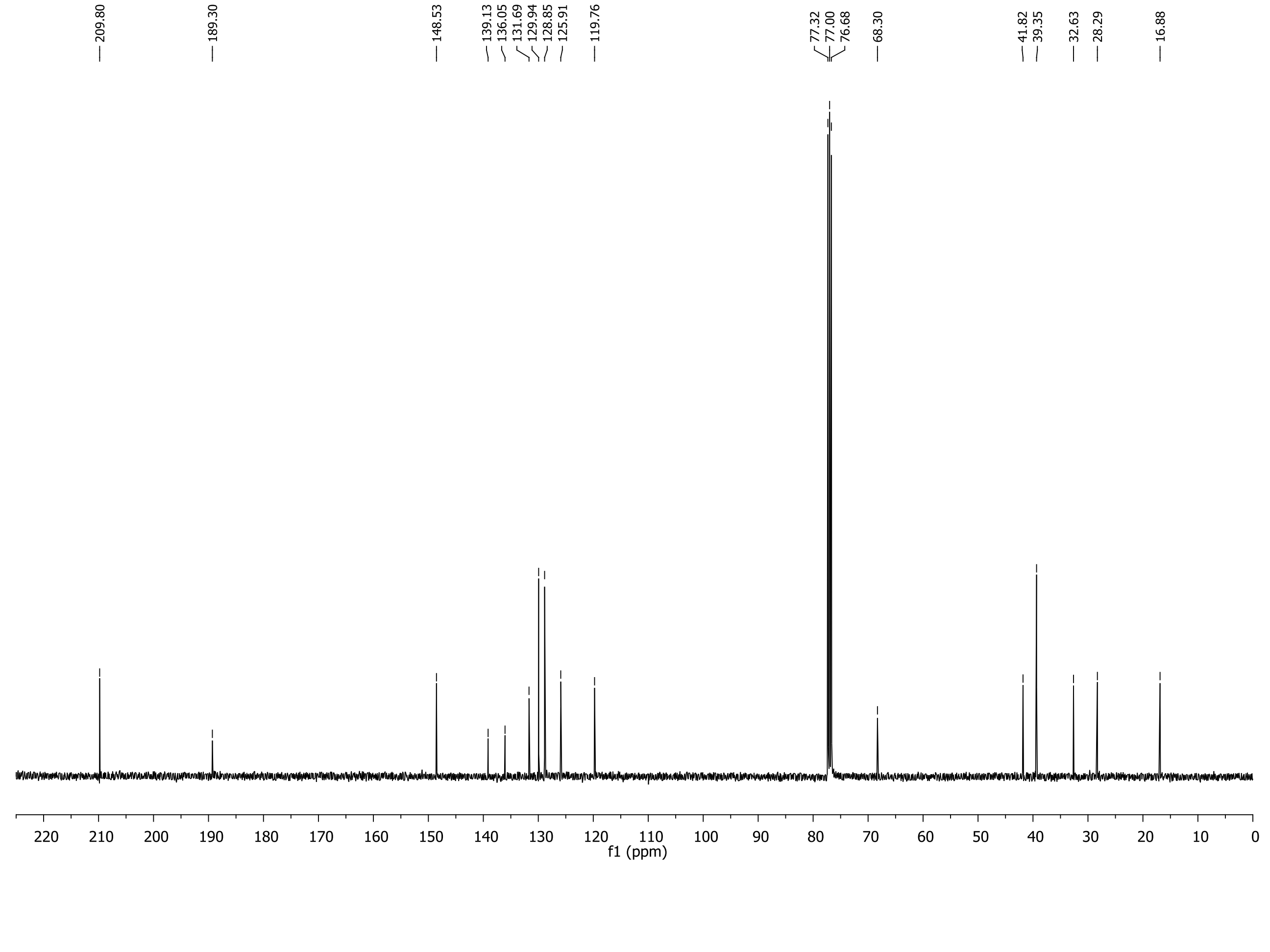
209.80
189.30
148.53
139.13
136.05
131.69
129.94
128.85
125.91
119.76
77.32
77.00
76.68
68.30
41.82
39.35
32.63
28.29
16.88
220 210 200 190 180 170 160 150 140 130 120 110 100 90 80 70 60 50 40 30 20 10 0
f1 (ppm)

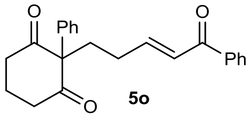
O
Ph
O
Ph
O

5o

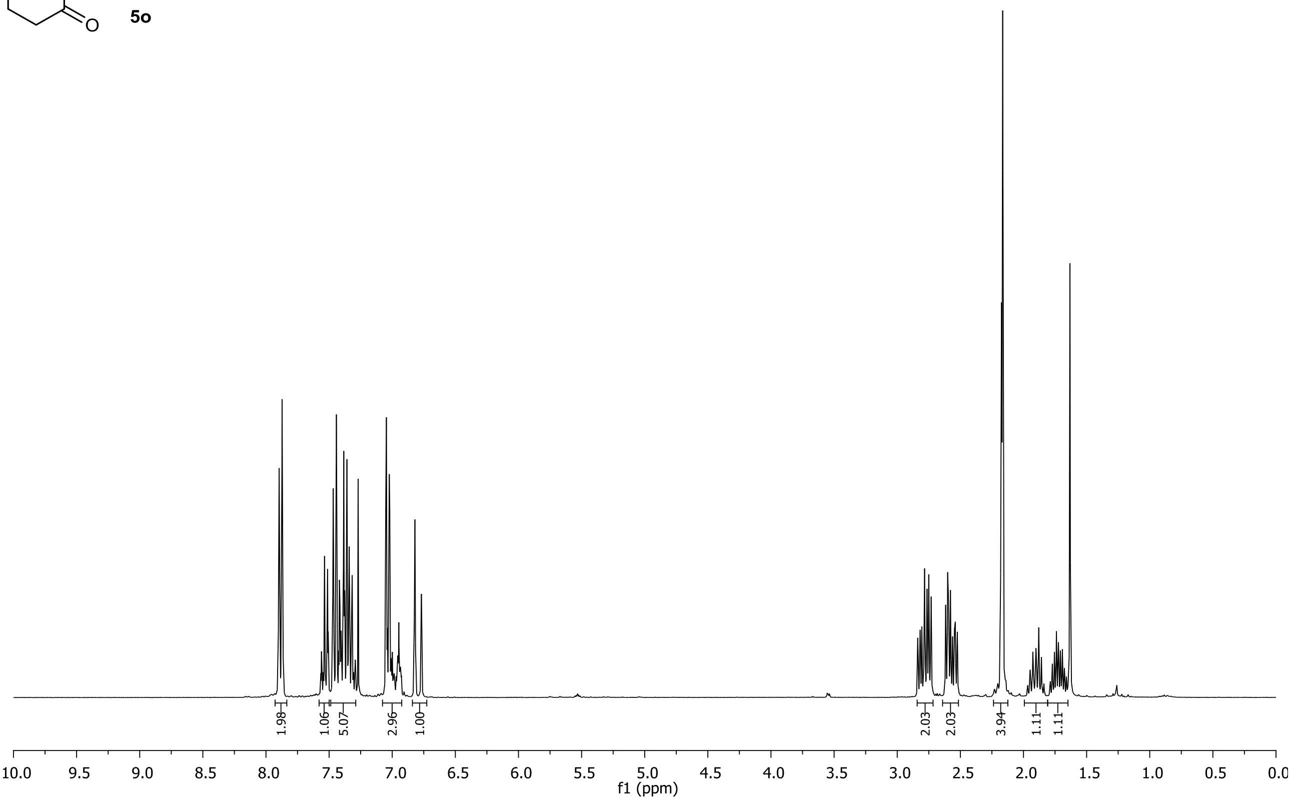
1.98
1.06
5.07
2.96
1.00
2.03
2.03
3.94
1.11
1.11
10.0 9.5 9.0 8.5 8.0 7.5 7.0 6.5 6.0 5.5 5.0 4.5 4.0 3.5 3.0 2.5 2.0 1.5 1.0 0.5 0.0
f1 (ppm)

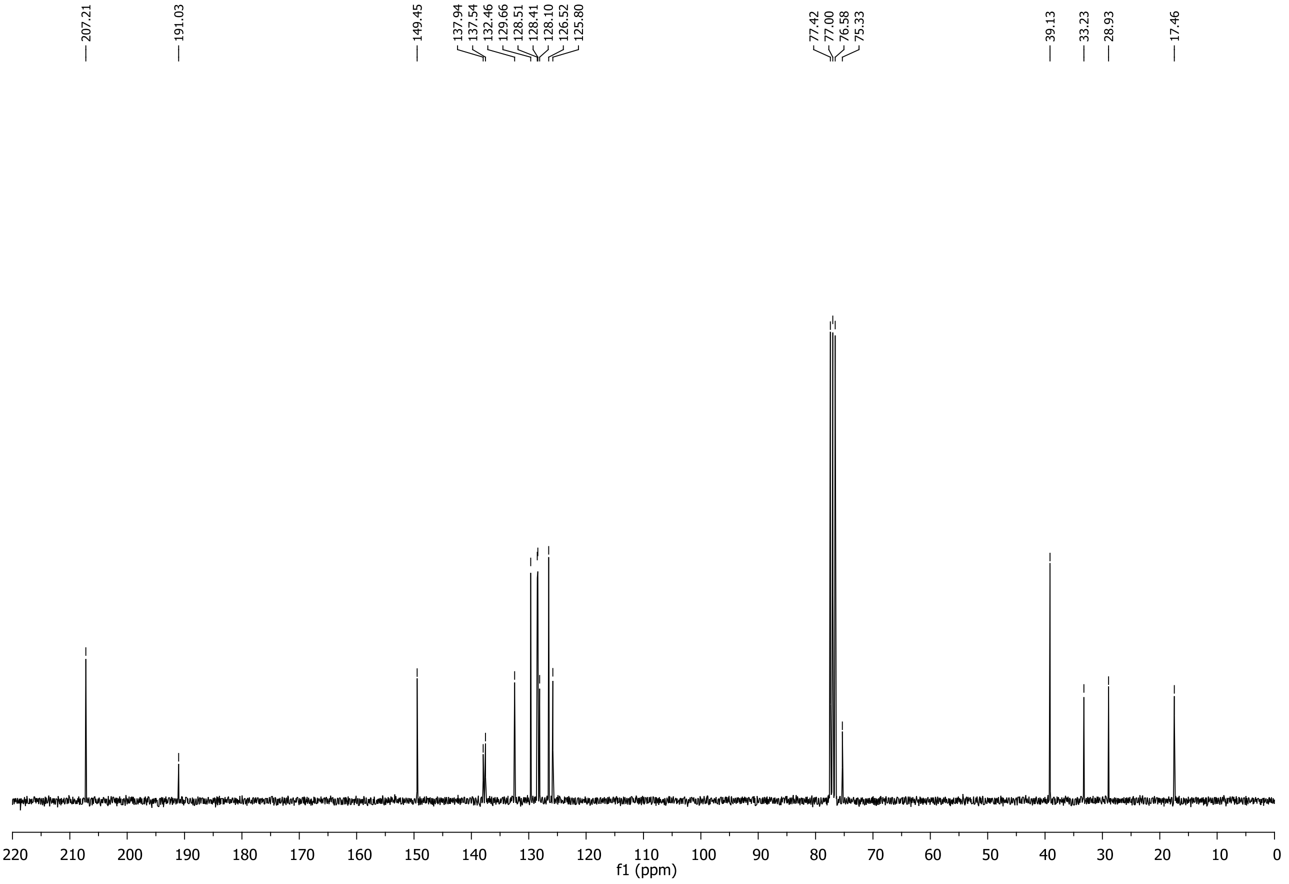
207.21
191.03
149.45
137.94
137.54
132.46
129.66
128.51
128.41
128.10
126.52
125.80
77.42
77.00
76.58
75.33
39.13
33.23
28.93
17.46
220 210 200 190 180 170 160 150 140 130 120 110 100 90 80 70 60 50 40 30 20 10 0
f1 (ppm)

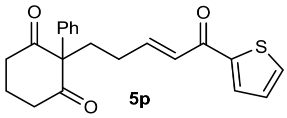
O
Ph
O
S
O

5p

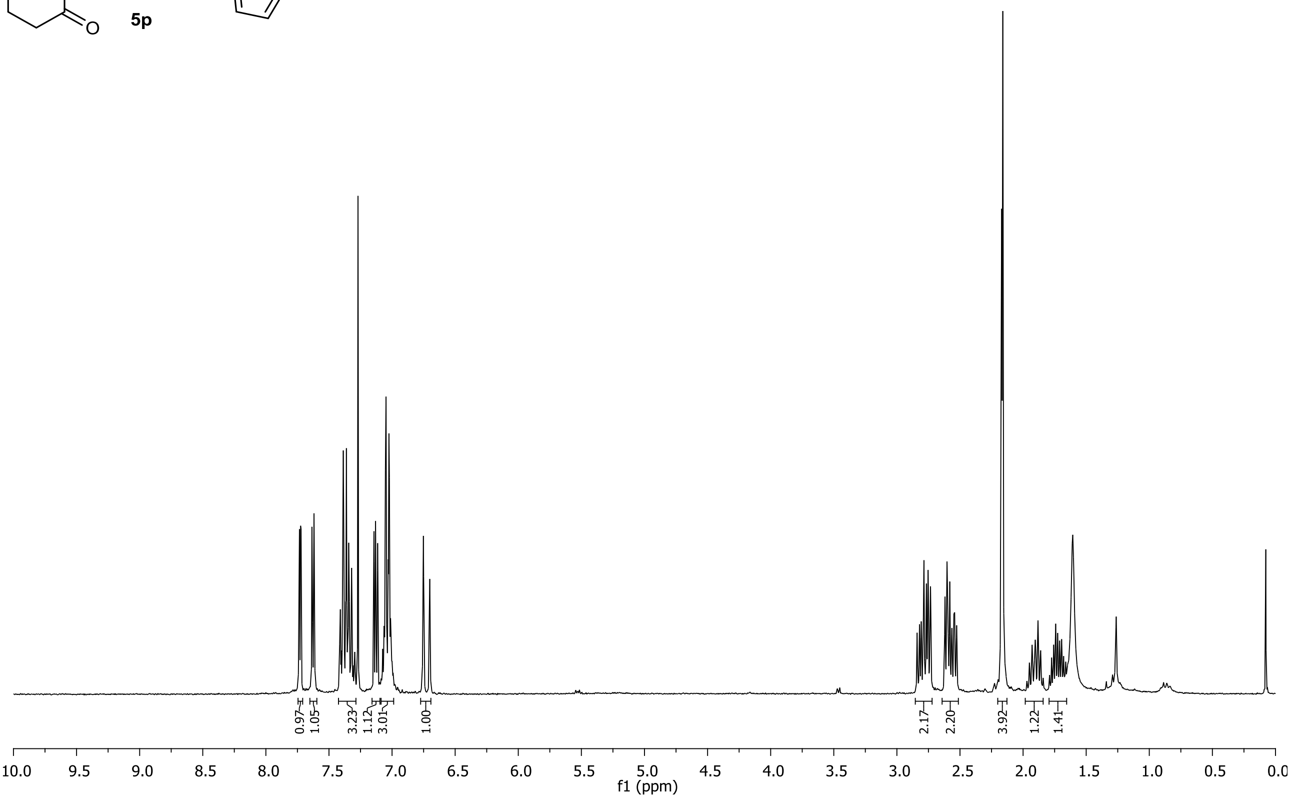
0.97
1.05
3.23
1.12
3.01
1.00
2.17
2.20
3.92
1.22
1.41
10.0 9.5 9.0 8.5 8.0 7.5 7.0 6.5 6.0 5.5 5.0 4.5 4.0 3.5 3.0 2.5 2.0 1.5 1.0 0.5 0.0
f1 (ppm)

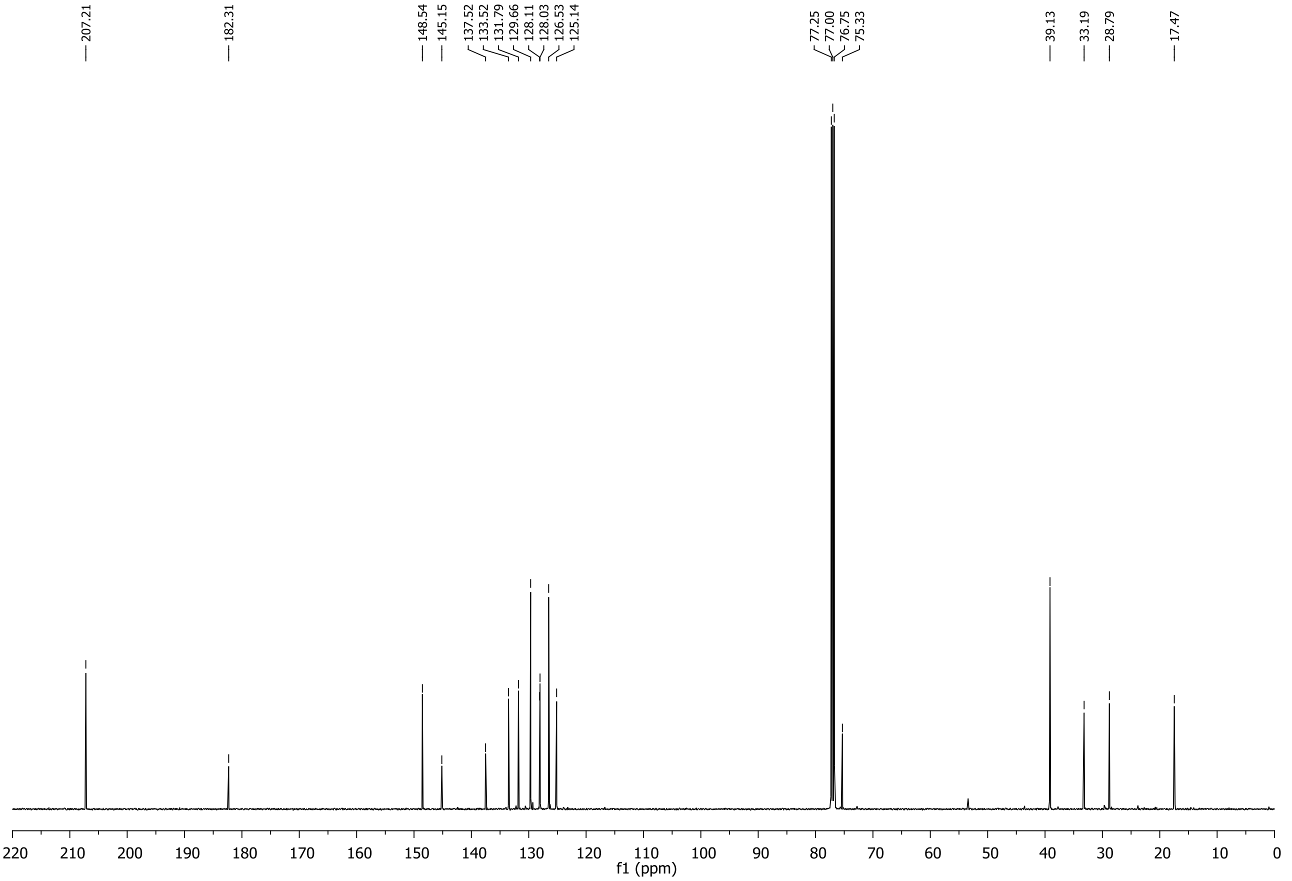
207.21
182.31
148.54
145.15
137.52
133.52
131.79
129.66
128.11
128.03
126.53
125.14
77.25
77.00
76.75
75.33
39.13
33.19
28.79
17.47
220 210 200 190 180 170 160 150 140 130 120 110 100 90 80 70 60 50 40 30 20 10 0
f1 (ppm)

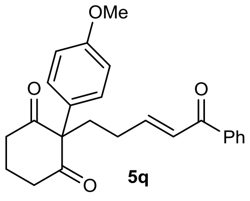
OMe
O
O
O
Ph

5q

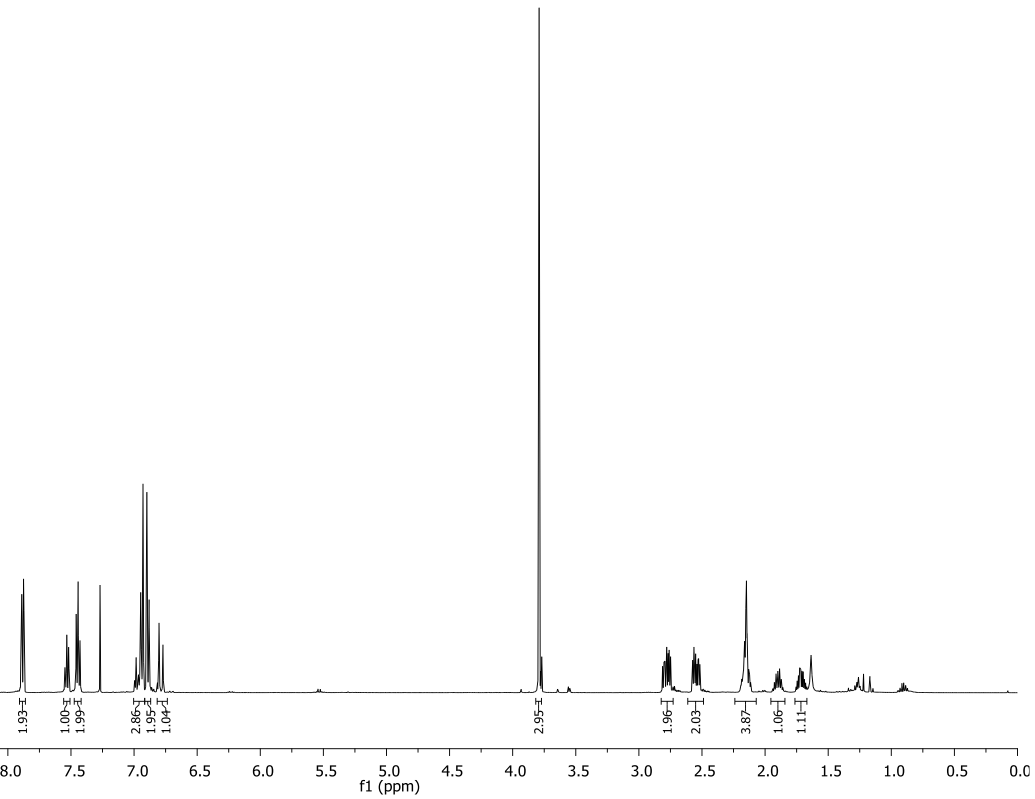
1.93
1.00
1.99
2.86
1.95
1.04
2.95
1.96
2.03
3.87
1.06
1.11
10.0 9.5 9.0 8.5 8.0 7.5 7.0 6.5 6.0 5.5 5.0 4.5 4.0 3.5 3.0 2.5 2.0 1.5 1.0 0.5 0.0
f1 (ppm)

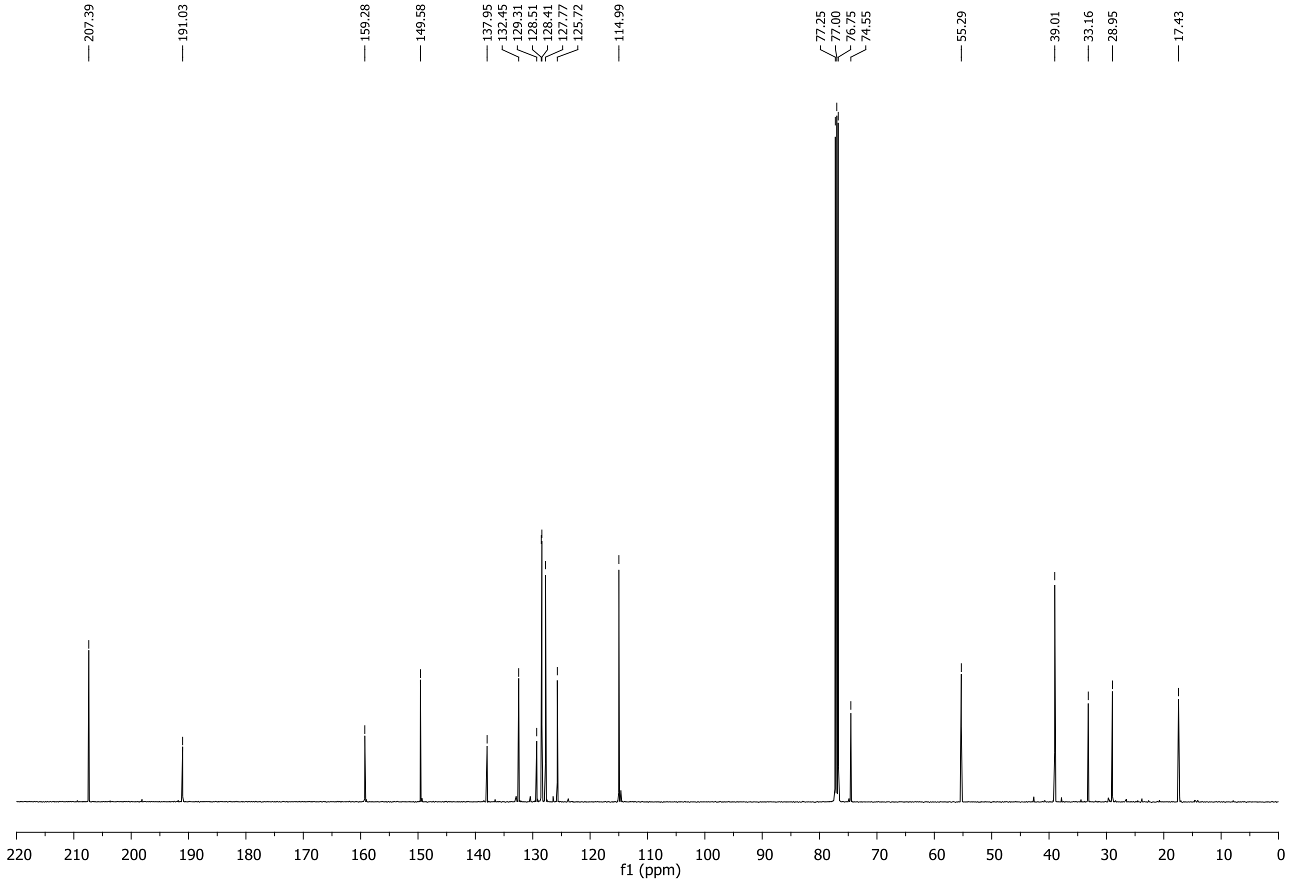
207.39
191.03
159.28
149.58
137.95
132.45
129.31
128.51
128.41
127.77
125.72
114.99
77.25
77.00
76.75
74.55
55.29
39.01
33.16
28.95
17.43
220 210 200 190 180 170 160 150 140 130 120 110 100 90 80 70 60 50 40 30 20 10 0
f1 (ppm)

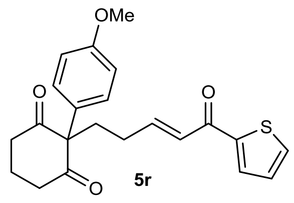
OMe
O
O
O
S
5r

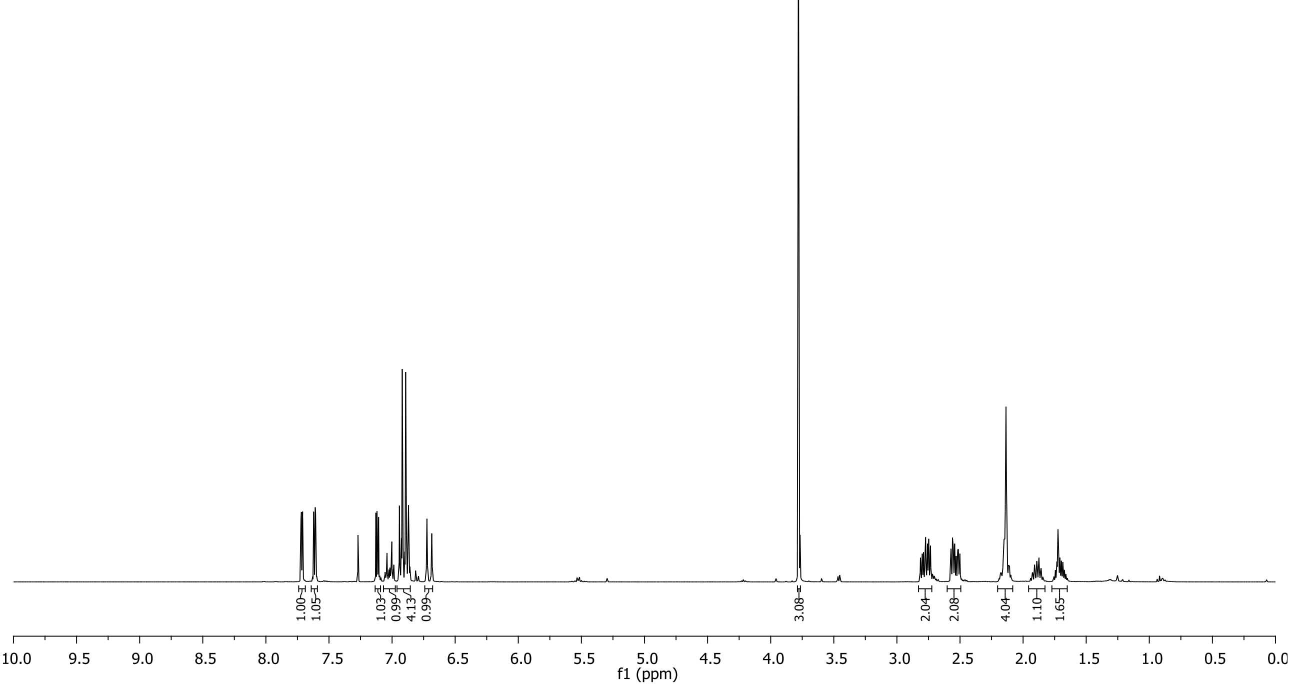
1.00
1.05
1.03
0.99
4.13
0.99
3.08
2.04
2.08
4.04
1.10
1.65
10.0 9.5 9.0 8.5 8.0 7.5 7.0 6.5 6.0 5.5 5.0 4.5 4.0 3.5 3.0 2.5 2.0 1.5 1.0 0.5 0.0
f1 (ppm)

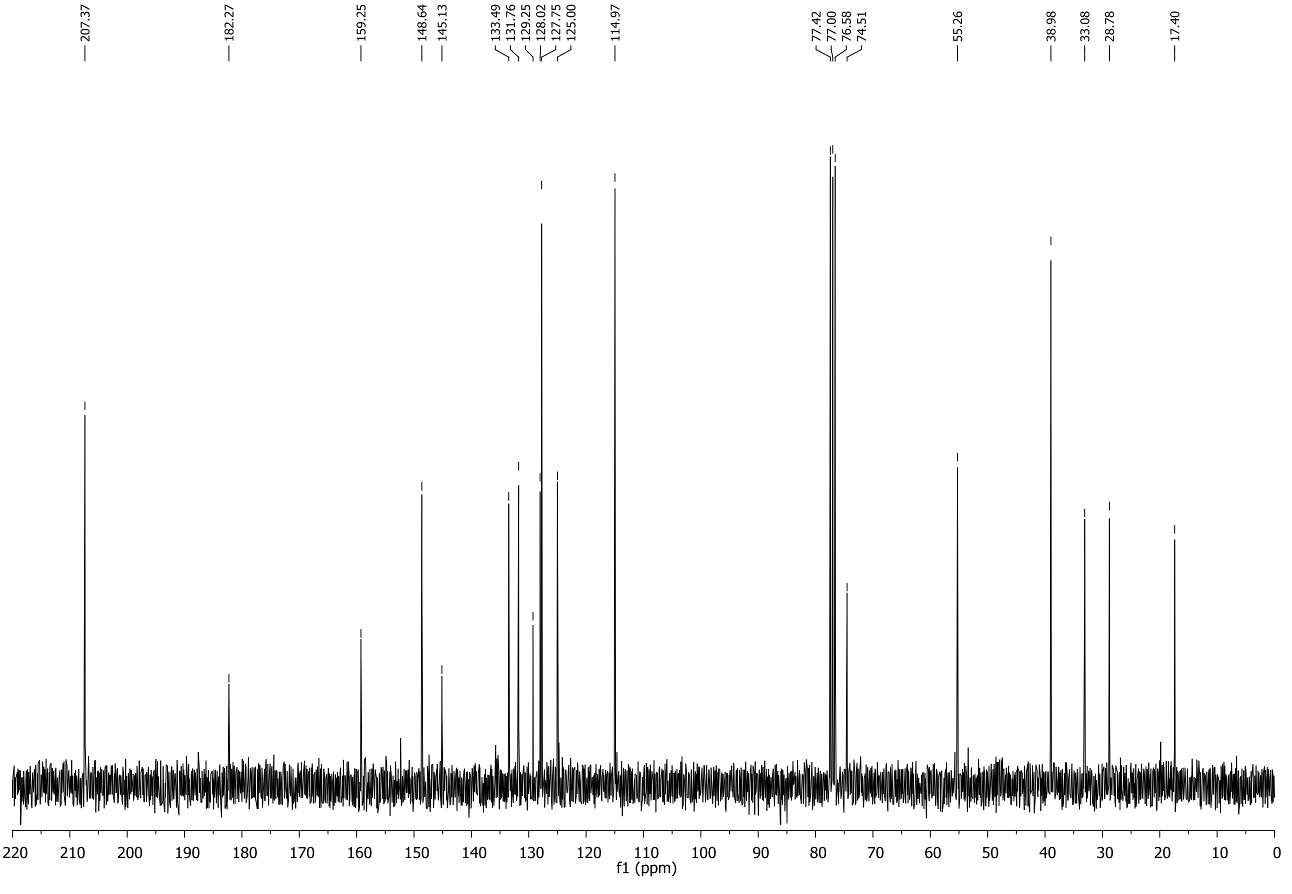
207.37
182.27
159.25
148.64
145.13
133.49
131.76
129.25
128.02
127.75
125.00
114.97
77.42
77.00
76.58
74.51
55.26
38.98
33.08
28.78
17.40
220 210 200 190 180 170 160 150 140 130 120 110 100 90 80 70 60 50 40 30 20 10 0
f1 (ppm)

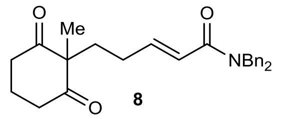

Me
NBn2

8

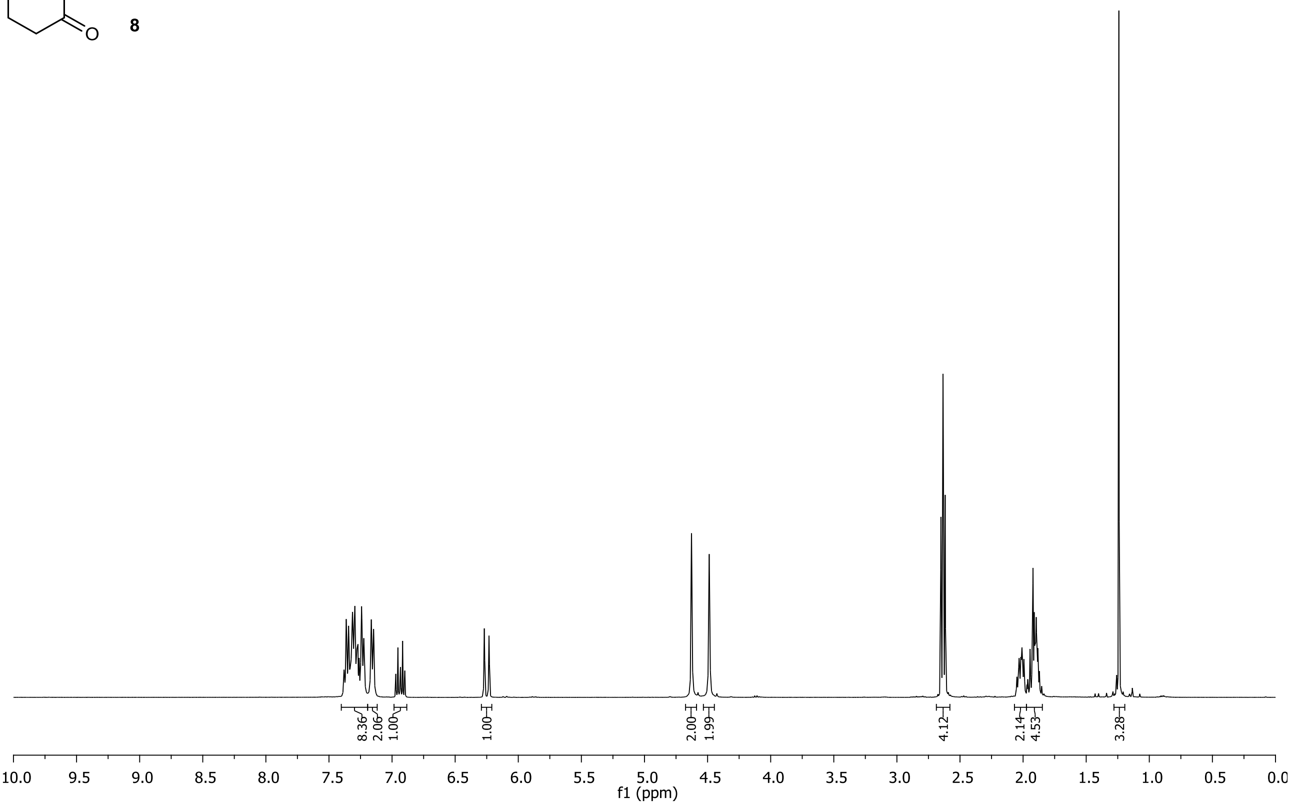

8.36
2.06
1.00
1.00
2.00
1.99
4.12
2.14
4.53
3.28
10.0 9.5 9.0 8.5 8.0 7.5 7.0 6.5 6.0 5.5 5.0 4.5 4.0 3.5 3.0 2.5 2.0 1.5 1.0 0.5 0.0
f1 (ppm)

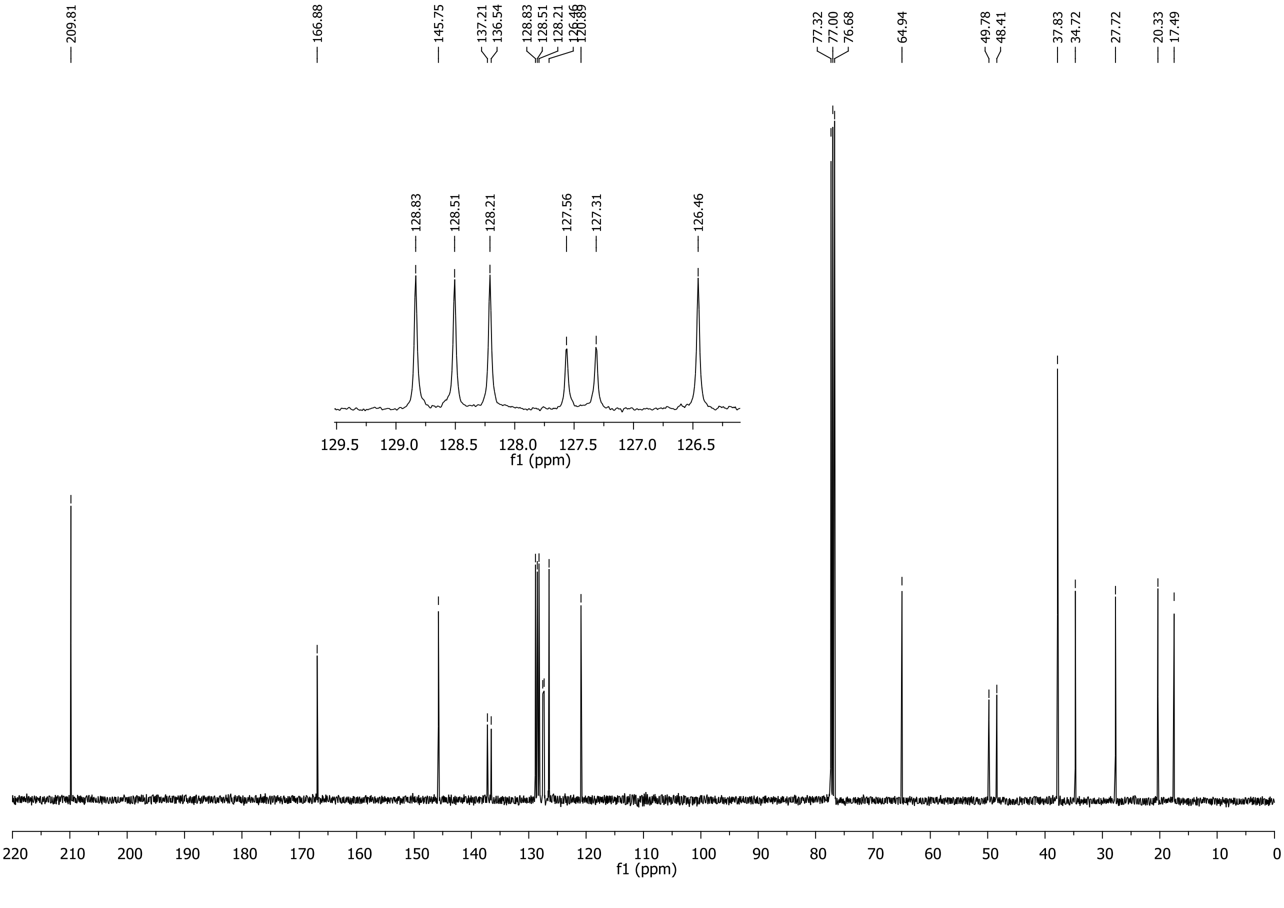

209.81
166.88
145.75
137.21
136.54
128.83
128.51
128.21
126.46
120.89
77.32
77.00
76.68
64.94
49.78
48.41
37.83
34.72
27.72
20.33
17.49
128.83
128.51
128.21
127.56
127.31
126.46
129.5 129.0 128.5 128.0 127.5 127.0 126.5
f1 (ppm)
220 210 200 190 180 170 160 150 140 130 120 110 100 90 80 70 60 50 40 30 20 10 0
f1 (ppm)

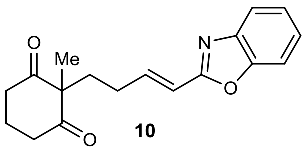
O
Me
N
O
O

10

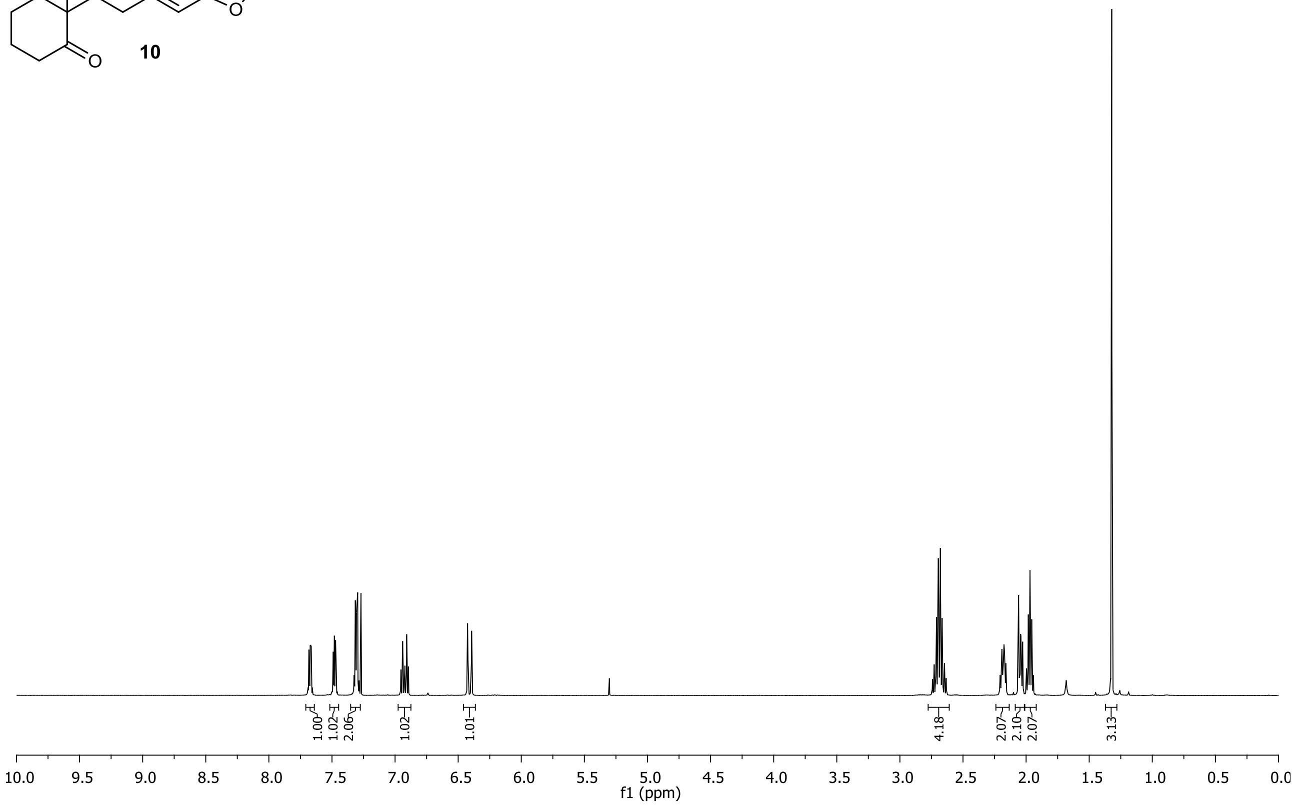
1.00
1.02
2.06
1.02
1.01
4.18
2.07
2.10
2.07
3.13
10.0 9.5 9.0 8.5 8.0 7.5 7.0 6.5 6.0 5.5 5.0 4.5 4.0 3.5 3.0 2.5 2.0 1.5 1.0 0.5 0.0
f1 (ppm)

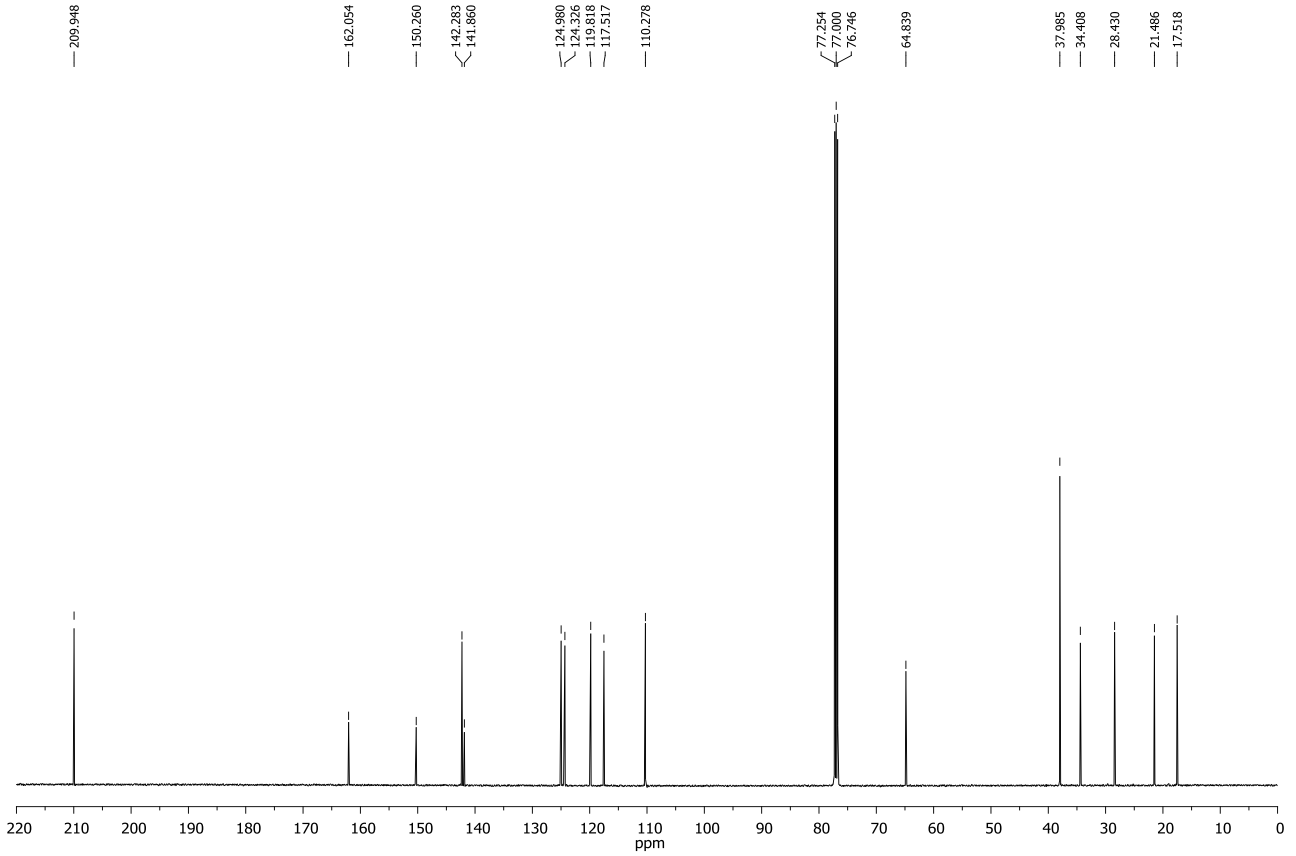
209.948
162.054
150.260
142.283
141.860
124.980
124.326
119.818
117.517
110.278
77.254
77.000
76.746
64.839
37.985
34.408
28.430
21.486
17.518
220 210 200 190 180 170 160 150 140 130 120 110 100 90 80 70 60 50 40 30 20 10 0
ppm

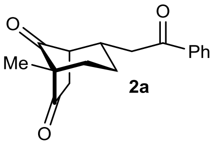
O
O
Me
Ph
O

2a

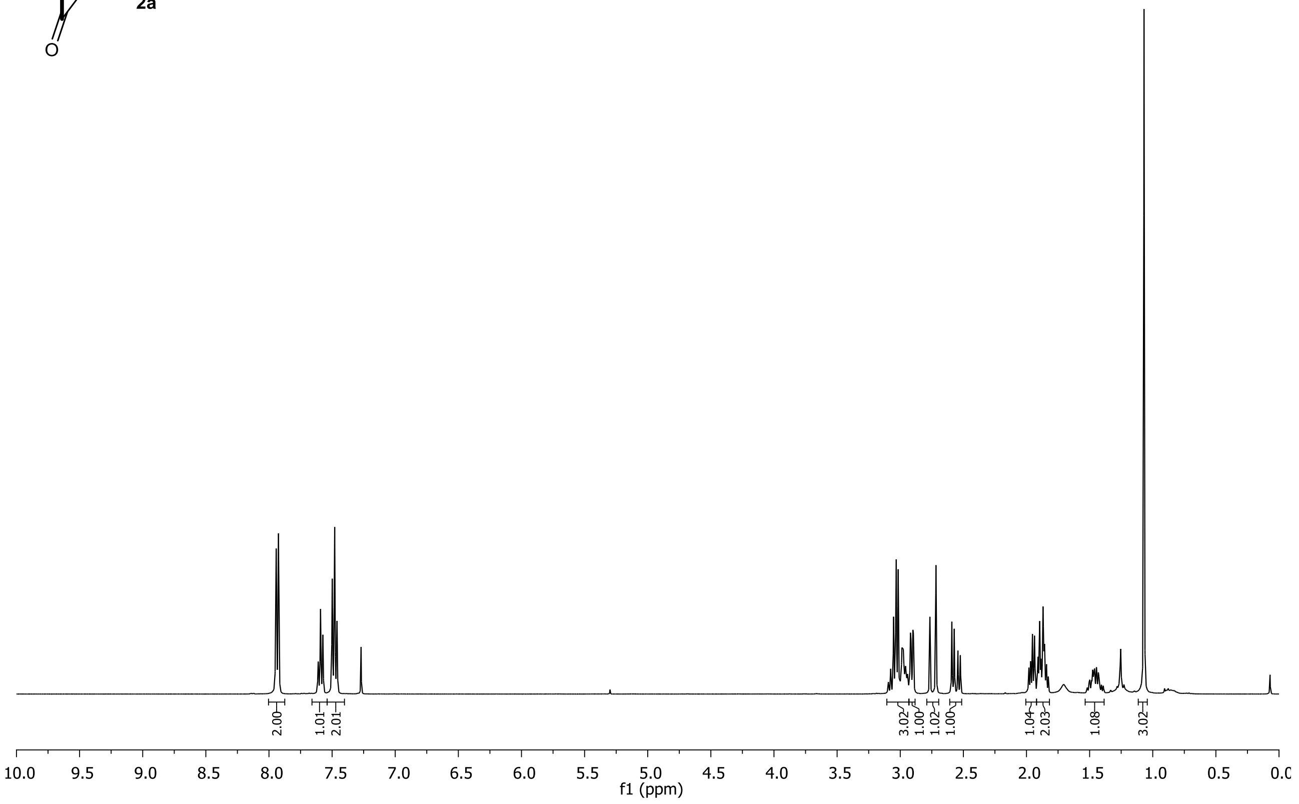
2.00
1.01
2.01
3.02
1.00
1.02
1.00
1.04
2.03
1.08
3.02
10.0 9.5 9.0 8.5 8.0 7.5 7.0 6.5 6.0 5.5 5.0 4.5 4.0 3.5 3.0 2.5 2.0 1.5 1.0 0.5 0.0
f1 (ppm)

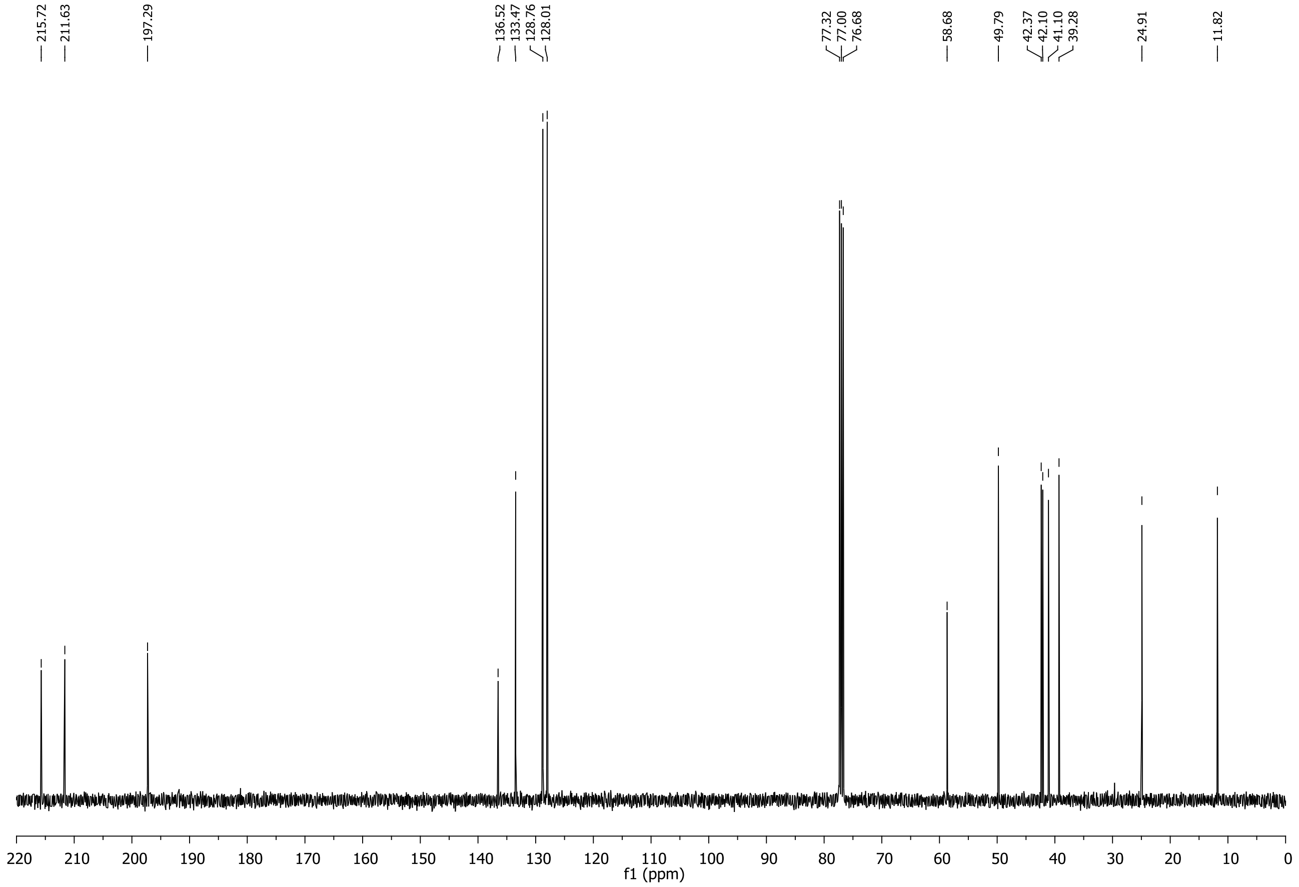
215.72
211.63
197.29
136.52
133.47
128.76
128.01
77.32
77.00
76.68
58.68
49.79
42.37
42.10
41.10
39.28
24.91
11.82
220 210 200 190 180 170 160 150 140 130 120 110 100 90 80 70 60 50 40 30 20 10 0
f1 (ppm)

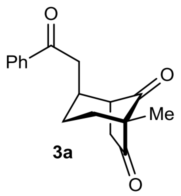
Ph
O
O
Me
O

3a

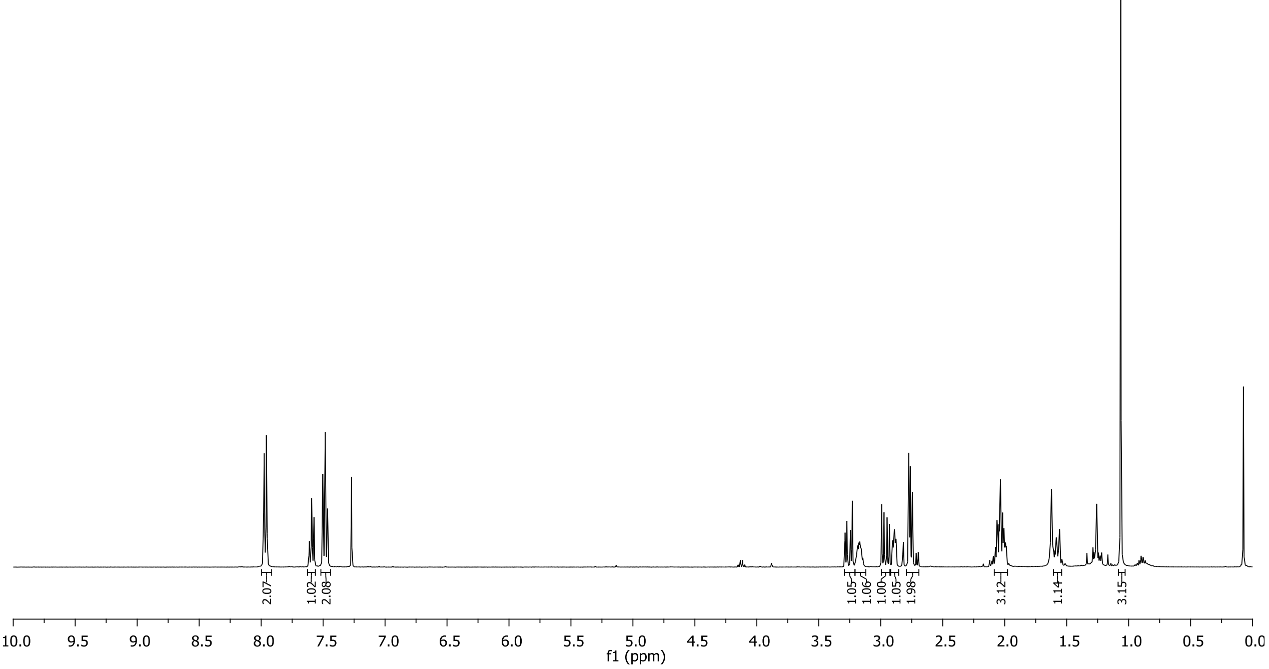
2.07
1.02
2.08
1.05
1.06
1.00
1.05
1.98
3.12
1.14
3.15
10.0 9.5 9.0 8.5 8.0 7.5 7.0 6.5 6.0 5.5 5.0 4.5 4.0 3.5 3.0 2.5 2.0 1.5 1.0 0.5 0.0
f1 (ppm)

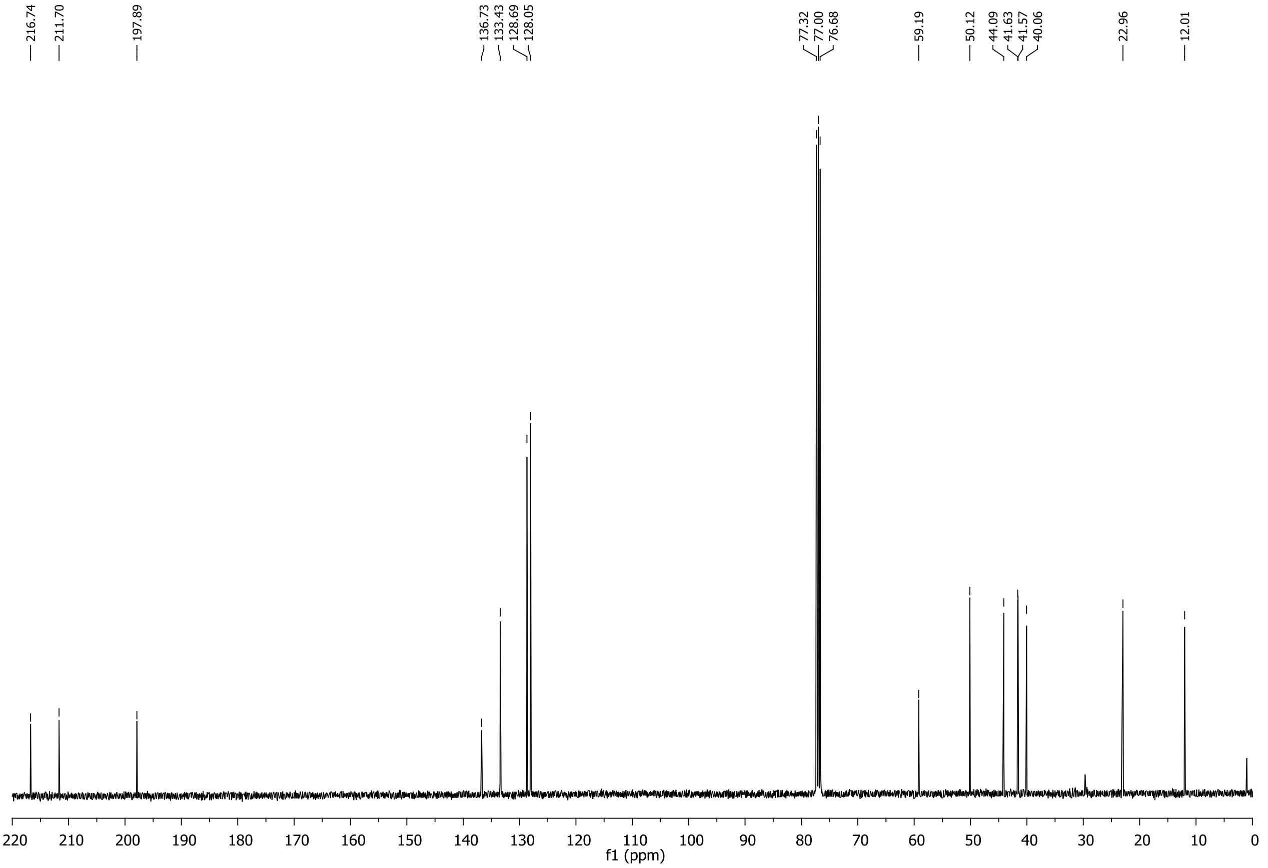
216.74
211.70
197.89
136.73
133.43
128.69
128.05
77.32
77.00
76.68
59.19
50.12
44.09
41.63
41.57
40.06
22.96
12.01
220 210 200 190 180 170 160 150 140 130 120 110 100 90 80 70 60 50 40 30 20 10 0
f1 (ppm)

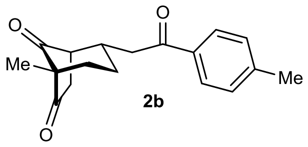
O
Me
O
O
Me

2b

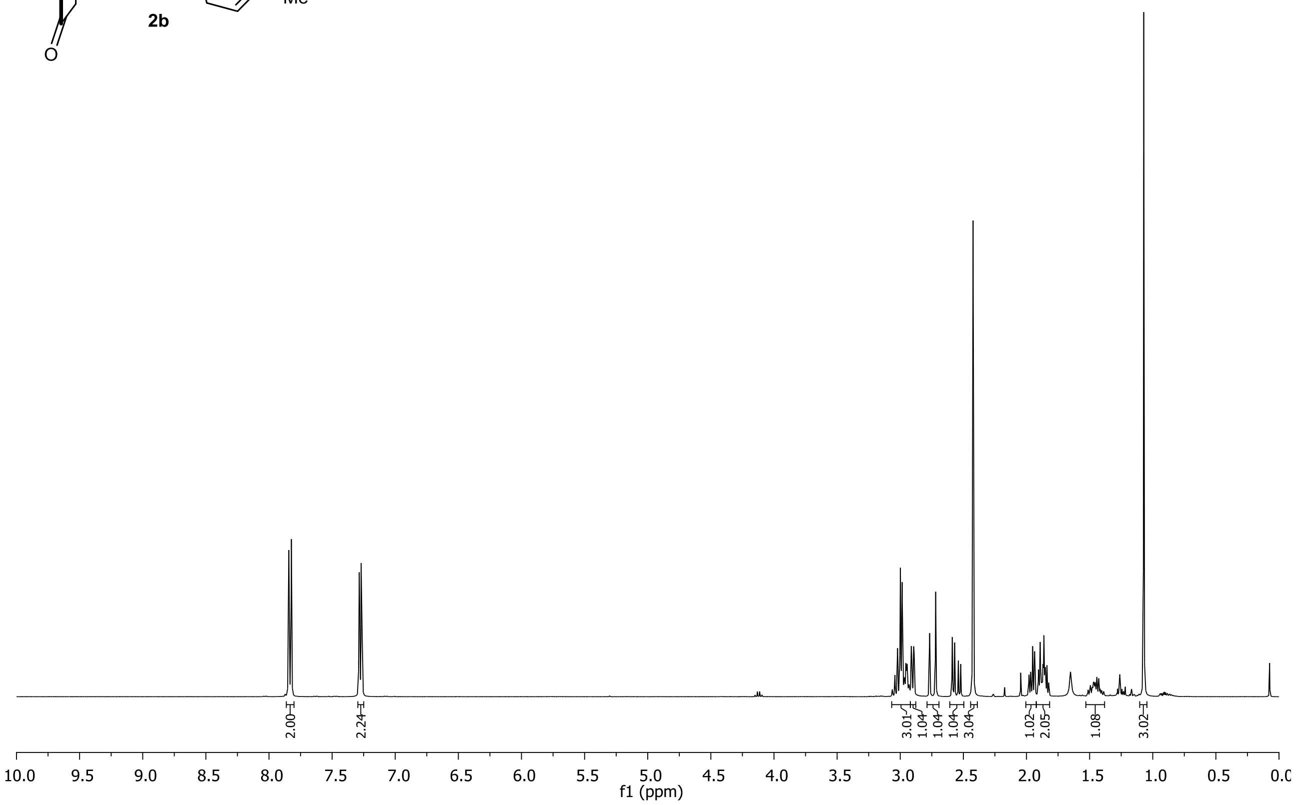
2.00
2.24
3.01
1.04
1.04
1.04
3.04
1.02
2.05
1.08
3.02
10.0 9.5 9.0 8.5 8.0 7.5 7.0 6.5 6.0 5.5 5.0 4.5 4.0 3.5 3.0 2.5 2.0 1.5 1.0 0.5 0.0
f1 (ppm)

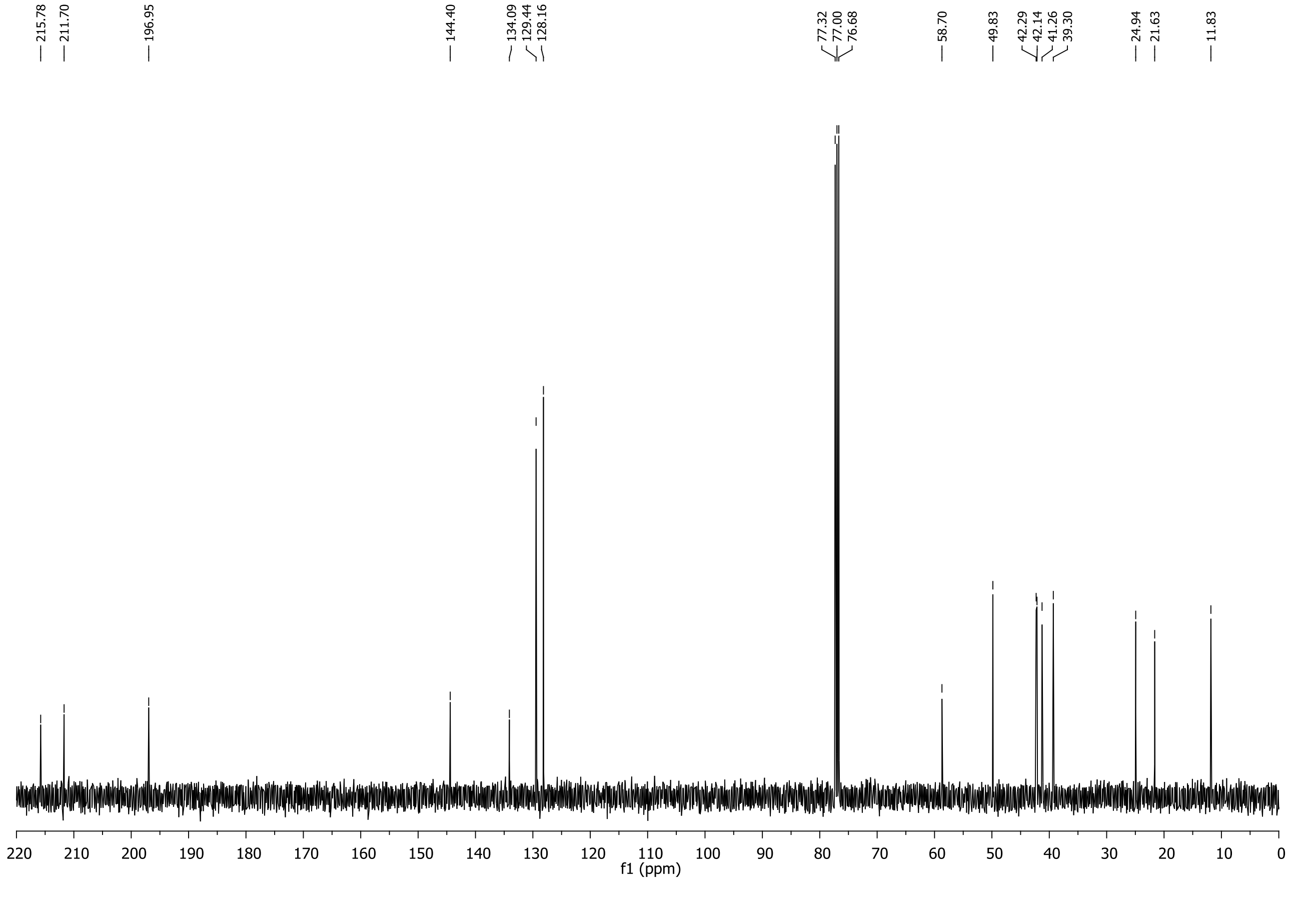
215.78
211.70
196.95
144.40
134.09
129.44
128.16
77.32
77.00
76.68
58.70
49.83
42.29
42.14
41.26
39.30
24.94
21.63
11.83
220 210 200 190 180 170 160 150 140 130 120 110 100 90 80 70 60 50 40 30 20 10 0
f1 (ppm)

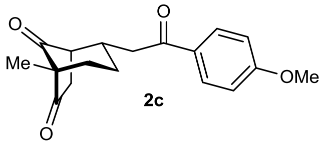
O
O
Me
OMe
O

2c

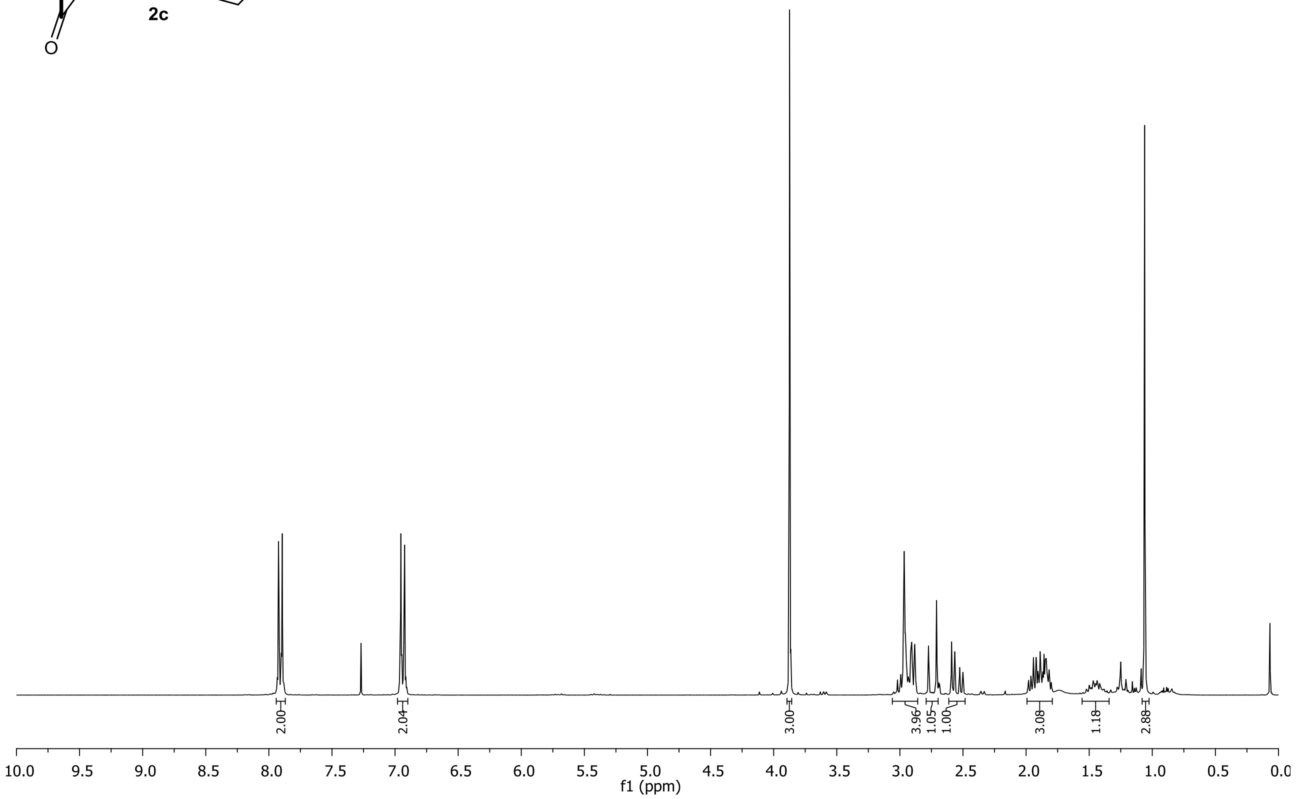
2.00
2.04
3.00
3.96
1.05
1.00
3.08
1.18
2.88
f1 (ppm)

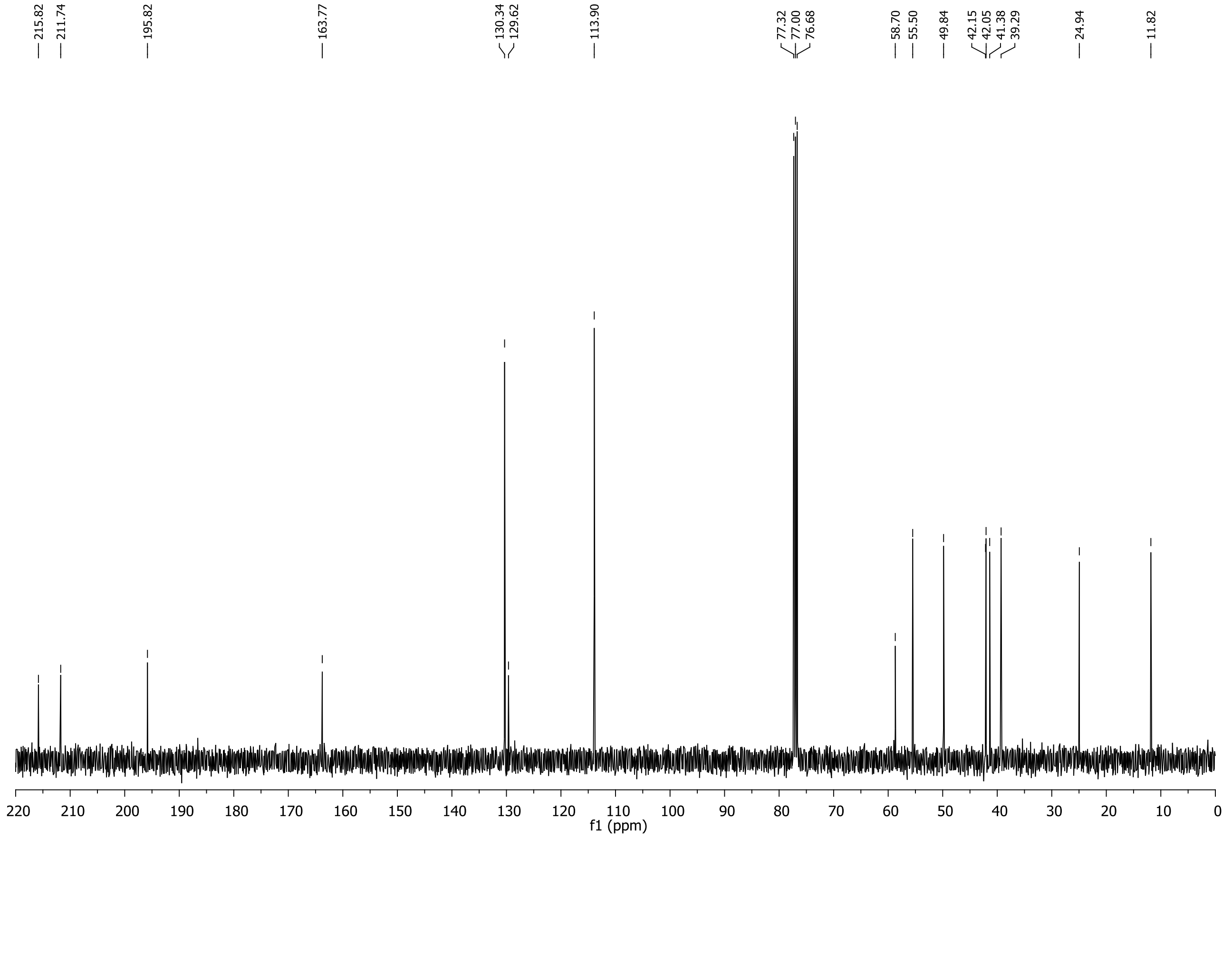
215.82
211.74
195.82
163.77
130.34
129.62
113.90
77.32
77.00
76.68
58.70
55.50
49.84
42.15
42.05
41.38
39.29
24.94
11.82
f1 (ppm)

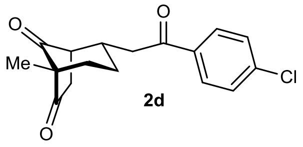
O
O
Me
Cl
O

2d

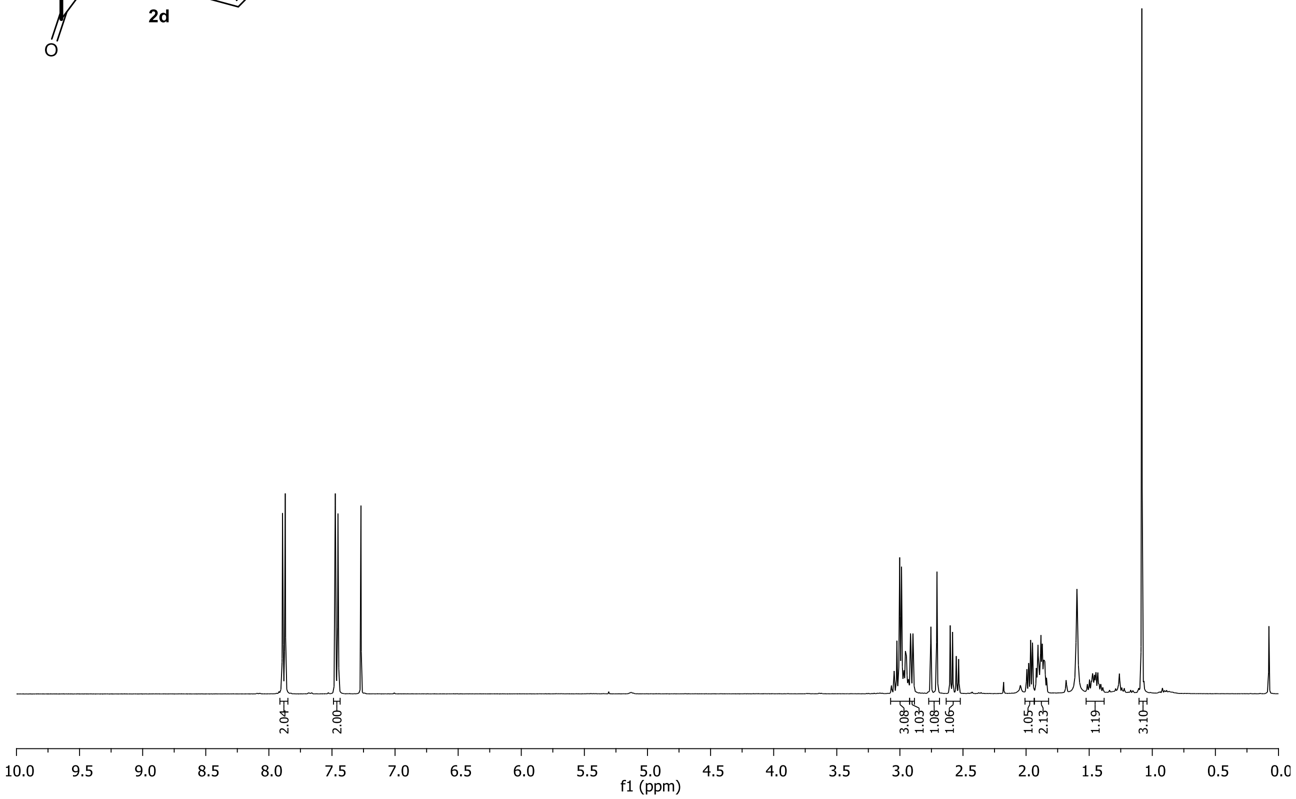
2.04
2.00
3.08
1.03
1.08
1.06
1.05
2.13
1.19
3.10
10.0 9.5 9.0 8.5 8.0 7.5 7.0 6.5 6.0 5.5 5.0 4.5 4.0 3.5 3.0 2.5 2.0 1.5 1.0 0.5 0.0
f1 (ppm)

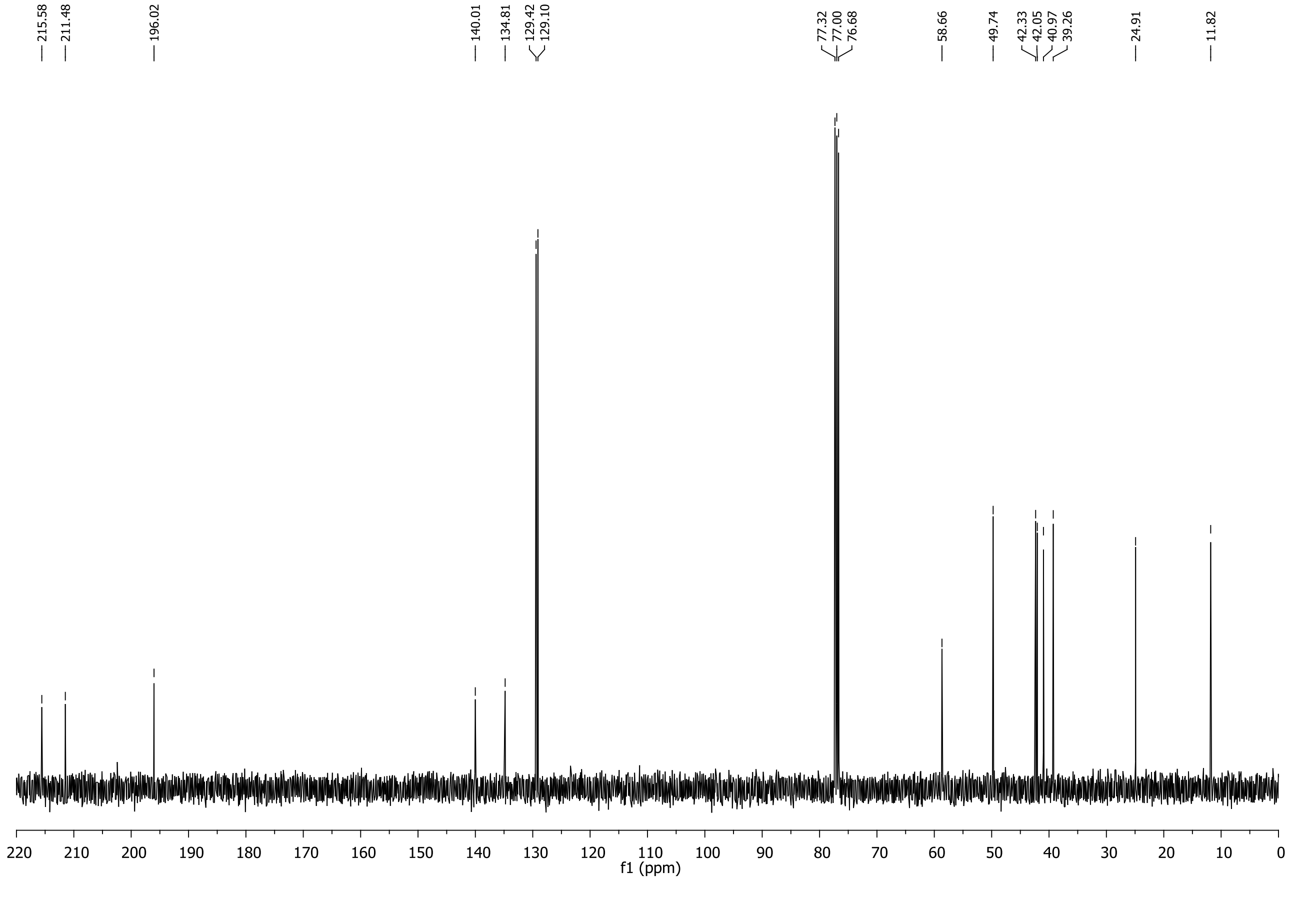
215.58
211.48
196.02
140.01
134.81
129.42
129.10
77.32
77.00
76.68
58.66
49.74
42.33
42.05
40.97
39.26
24.91
11.82
220 210 200 190 180 170 160 150 140 130 120 110 100 90 80 70 60 50 40 30 20 10 0
f1 (ppm)

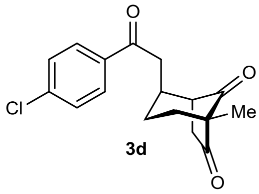
O
O
Cl
Me
O

3d

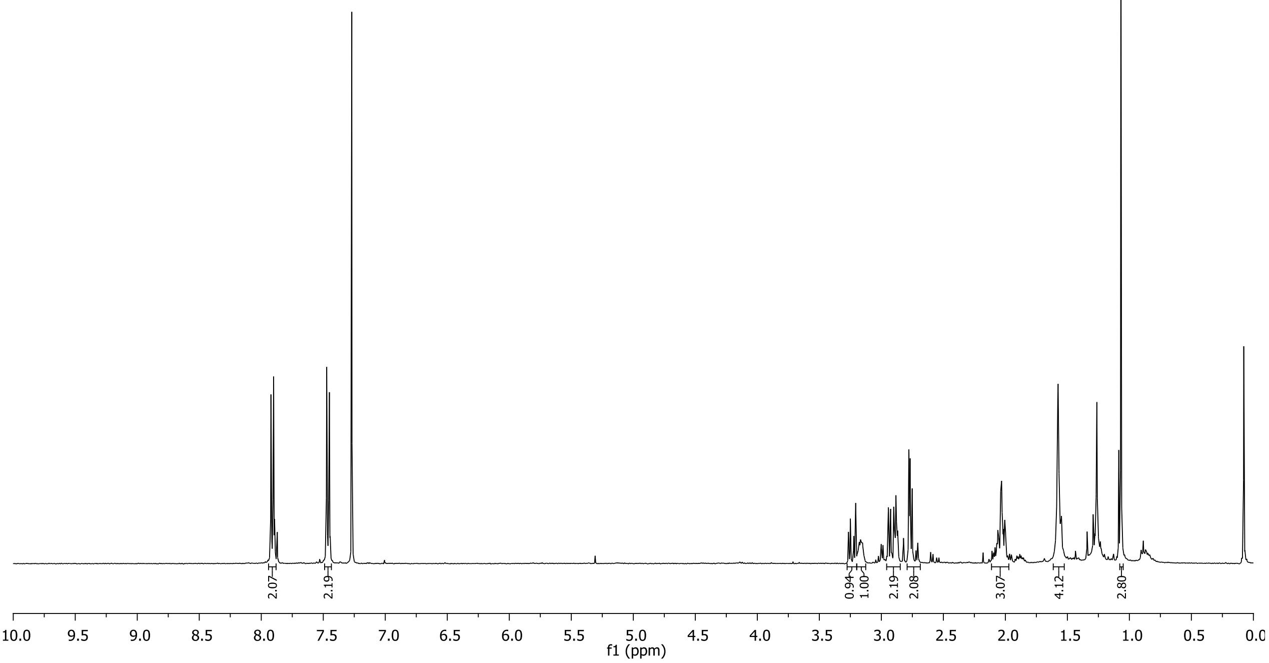
2.07
2.19
0.94
1.00
2.19
2.08
3.07
4.12
2.80
10.0 9.5 9.0 8.5 8.0 7.5 7.0 6.5 6.0 5.5 5.0 4.5 4.0 3.5 3.0 2.5 2.0 1.5 1.0 0.5 0.0
f1 (ppm)

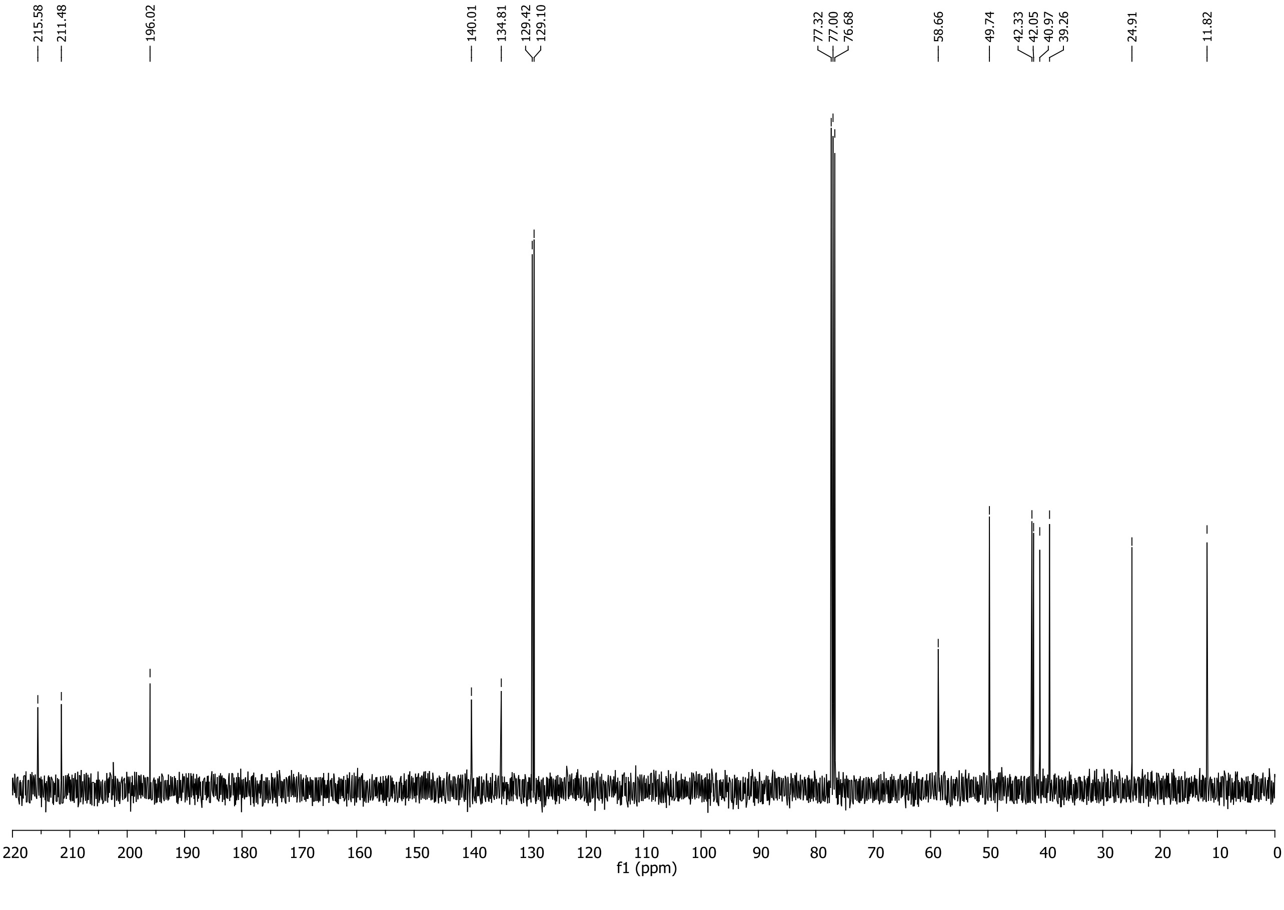
215.58
211.48
196.02
140.01
134.81
129.42
129.10
77.32
77.00
76.68
58.66
49.74
42.33
42.05
40.97
39.26
24.91
11.82
220 210 200 190 180 170 160 150 140 130 120 110 100 90 80 70 60 50 40 30 20 10 0
f1 (ppm)

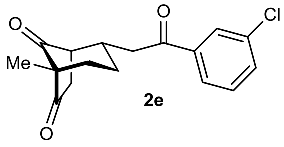
O
O
Cl
Me
O

2e

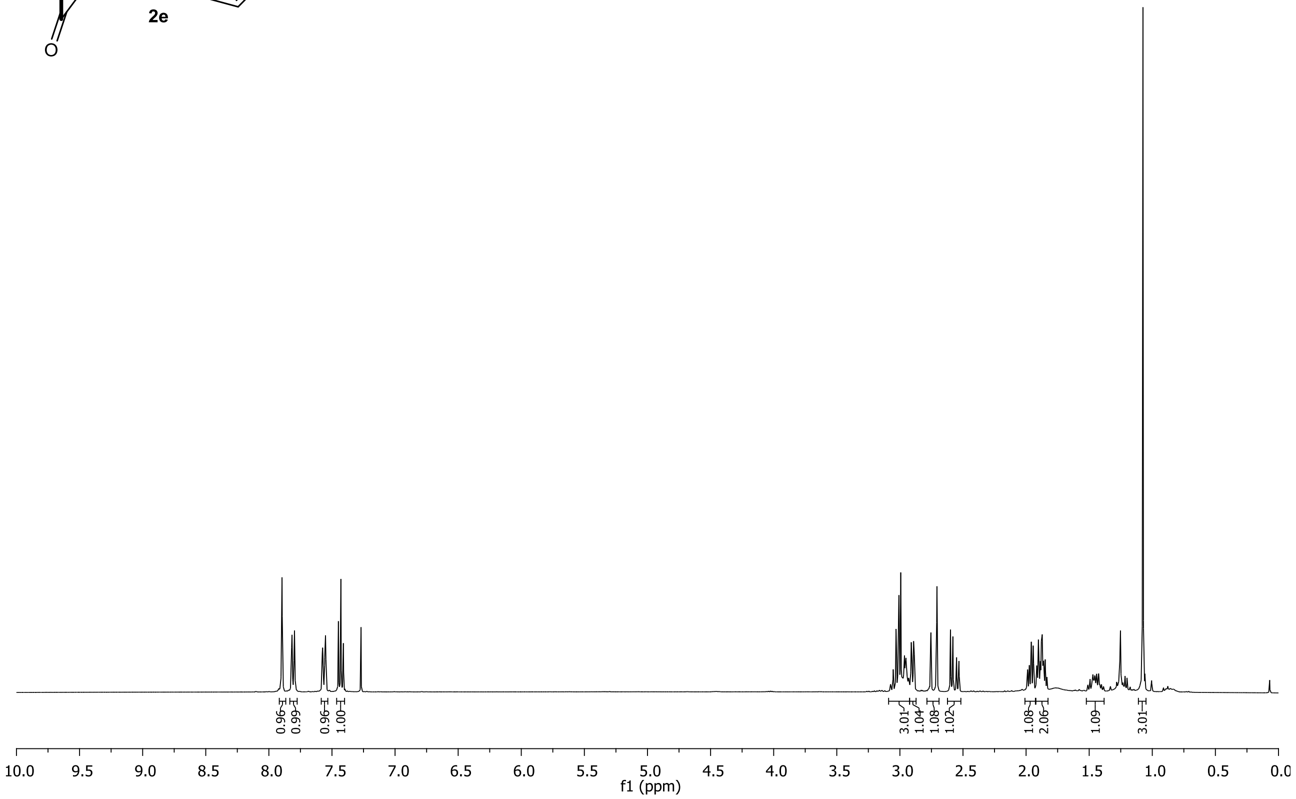
0.96
0.99
0.96
1.00
3.01
1.04
1.08
1.02
1.08
2.06
1.09
3.01
10.0 9.5 9.0 8.5 8.0 7.5 7.0 6.5 6.0 5.5 5.0 4.5 4.0 3.5 3.0 2.5 2.0 1.5 1.0 0.5 0.0
f1 (ppm)

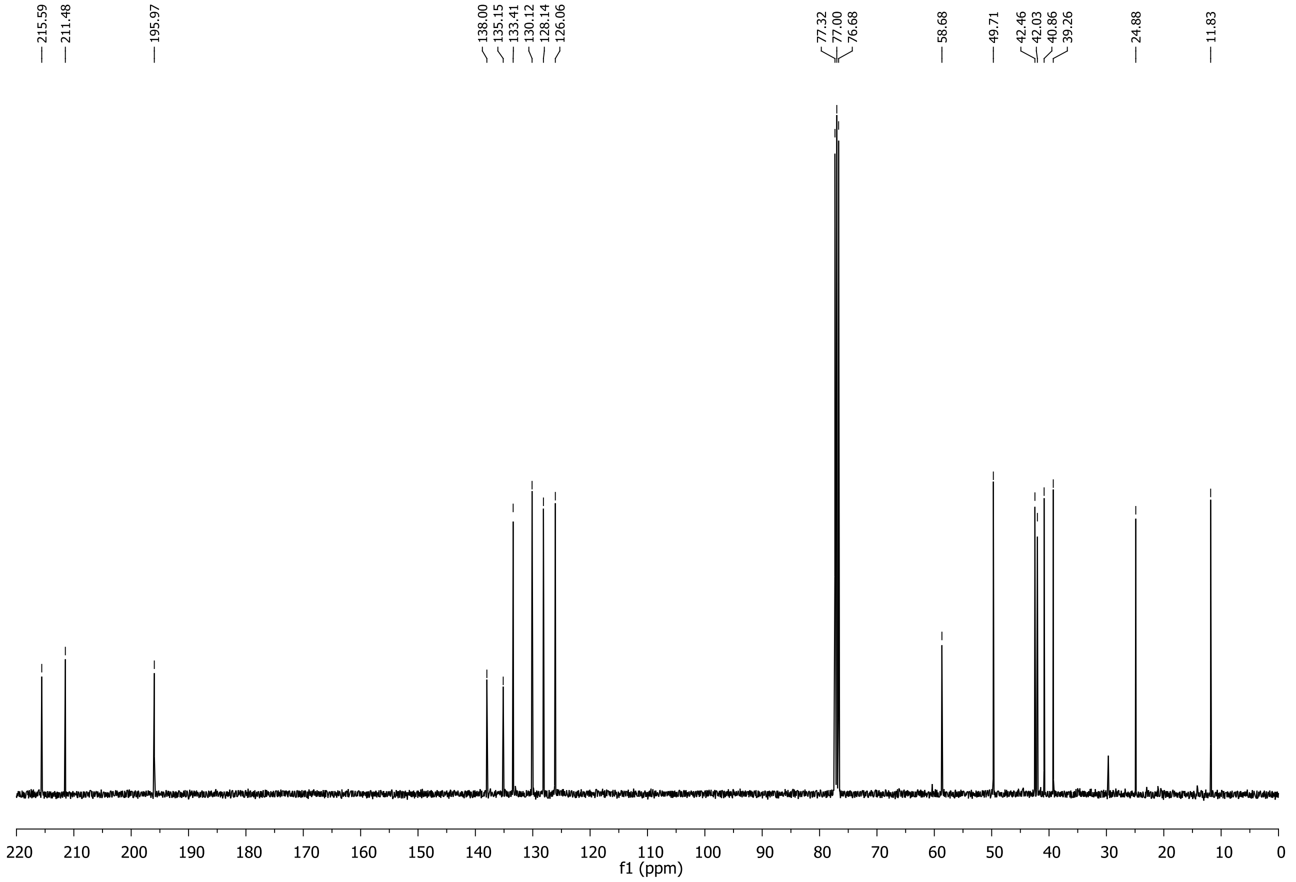
215.59
211.48
195.97
138.00
135.15
133.41
130.12
128.14
126.06
77.32
77.00
76.68
58.68
49.71
42.46
42.03
40.86
39.26
24.88
11.83
220 210 200 190 180 170 160 150 140 130 120 110 100 90 80 70 60 50 40 30 20 10 0
f1 (ppm)

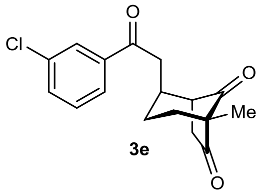
O
Cl
O
Me
O

3e

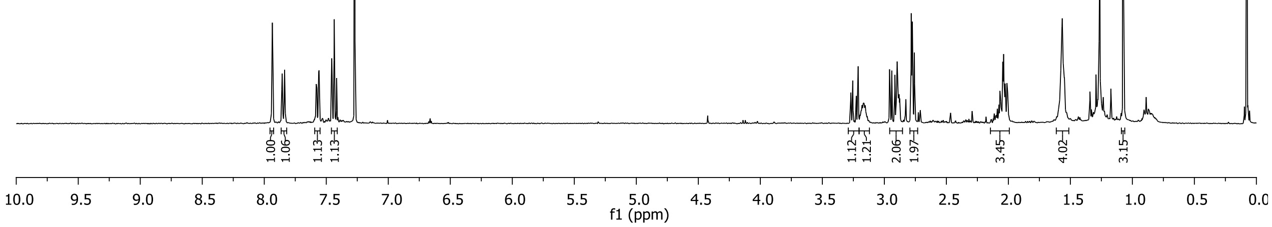
1.00
1.06
1.13
1.13
1.12
1.21
2.06
1.97
3.45
4.02
3.15
10.0 9.5 9.0 8.5 8.0 7.5 7.0 6.5 6.0 5.5 5.0 4.5 4.0 3.5 3.0 2.5 2.0 1.5 1.0 0.5 0.0
f1 (ppm)

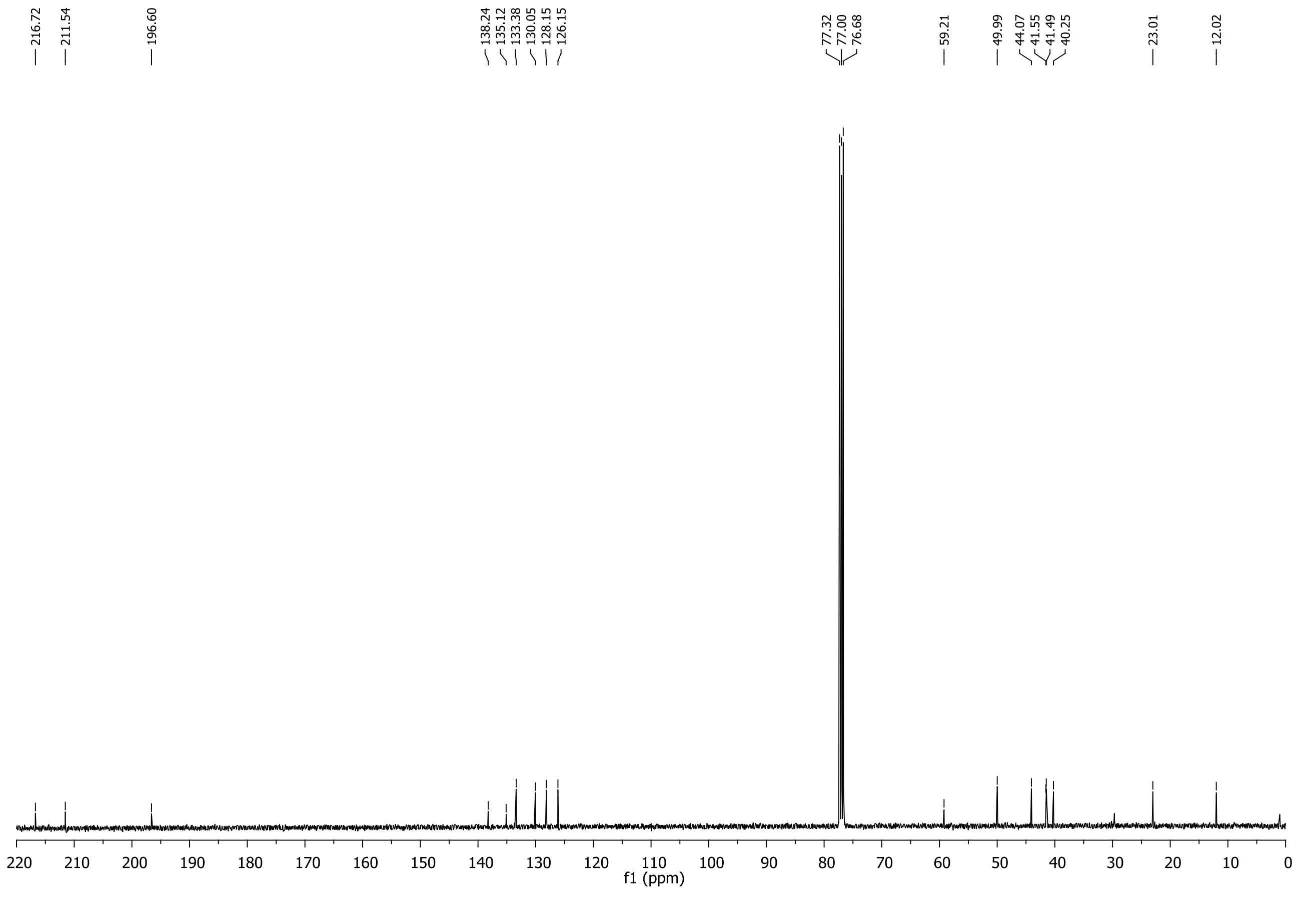
216.72
211.54
196.60
138.24
135.12
133.38
130.05
128.15
126.15
77.32
77.00
76.68
59.21
49.99
44.07
41.55
41.49
40.25
23.01
12.02
220 210 200 190 180 170 160 150 140 130 120 110 100 90 80 70 60 50 40 30 20 10 0
f1 (ppm)

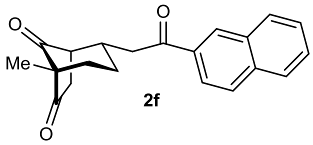
O
O
Me
O

2f

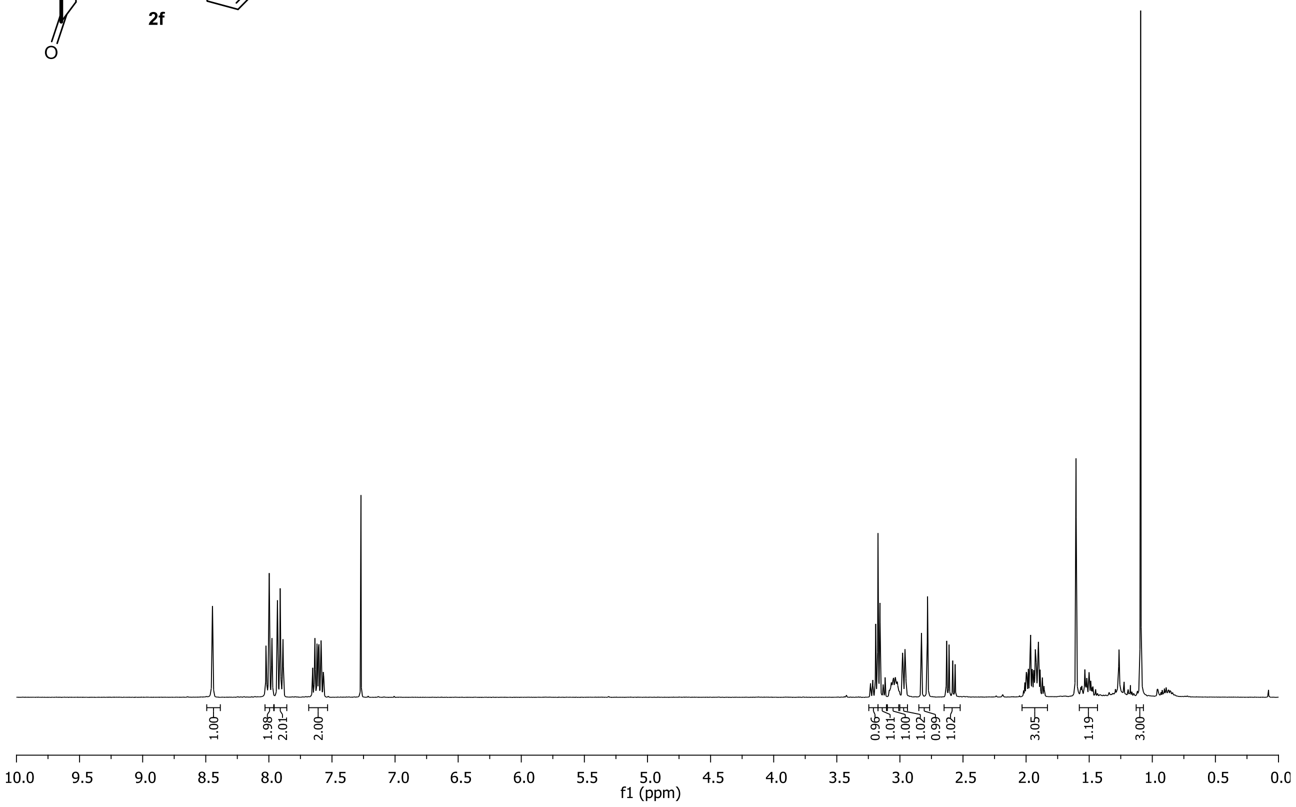
1.00
1.98
2.01
2.00
0.96
1.01
1.00
1.02
0.99
1.02
3.05
1.19
3.00
10.0 9.5 9.0 8.5 8.0 7.5 7.0 6.5 6.0 5.5 5.0 4.5 4.0 3.5 3.0 2.5 2.0 1.5 1.0 0.5 0.0
f1 (ppm)

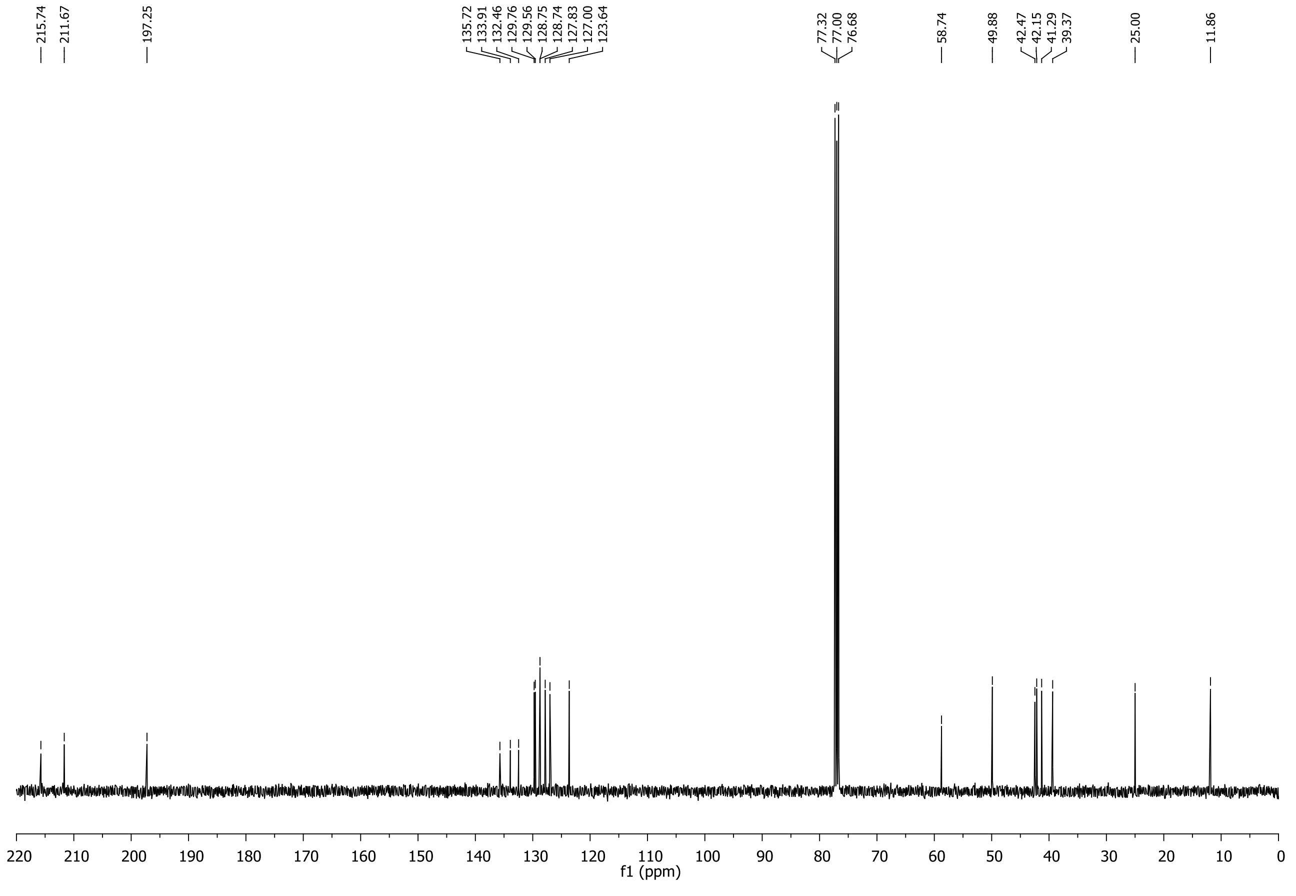
215.74
211.67
197.25
135.72
133.91
132.46
129.76
129.56
128.75
128.74
127.83
127.00
123.64
77.32
77.00
76.68
58.74
49.88
42.47
42.15
41.29
39.37
25.00
11.86
220 210 200 190 180 170 160 150 140 130 120 110 100 90 80 70 60 50 40 30 20 10 0
f1 (ppm)

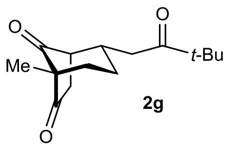
O
O
Me
O
t-Bu

2g

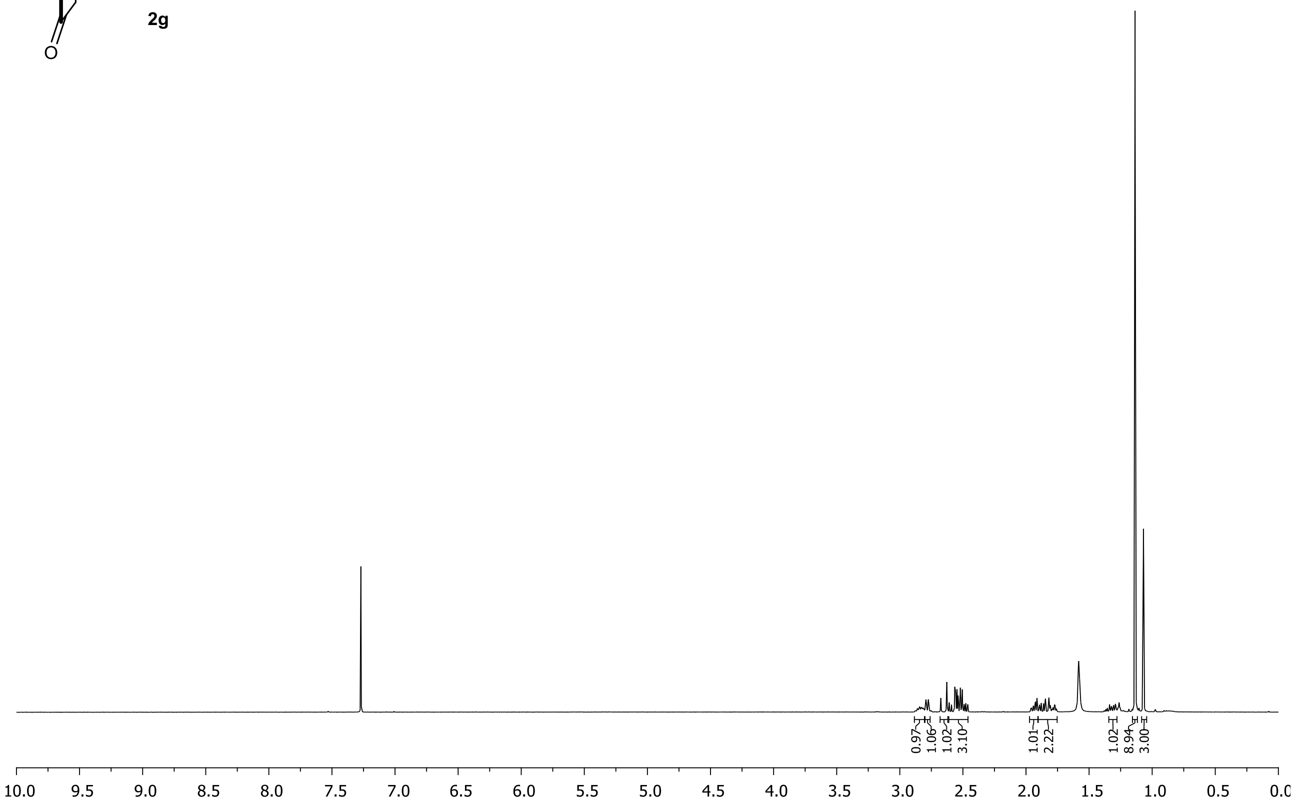
0.97
1.06
1.02
3.10
1.01
2.22
1.02
8.94
3.00
10.0 9.5 9.0 8.5 8.0 7.5 7.0 6.5 6.0 5.5 5.0 4.5 4.0 3.5 3.0 2.5 2.0 1.5 1.0 0.5 0.0

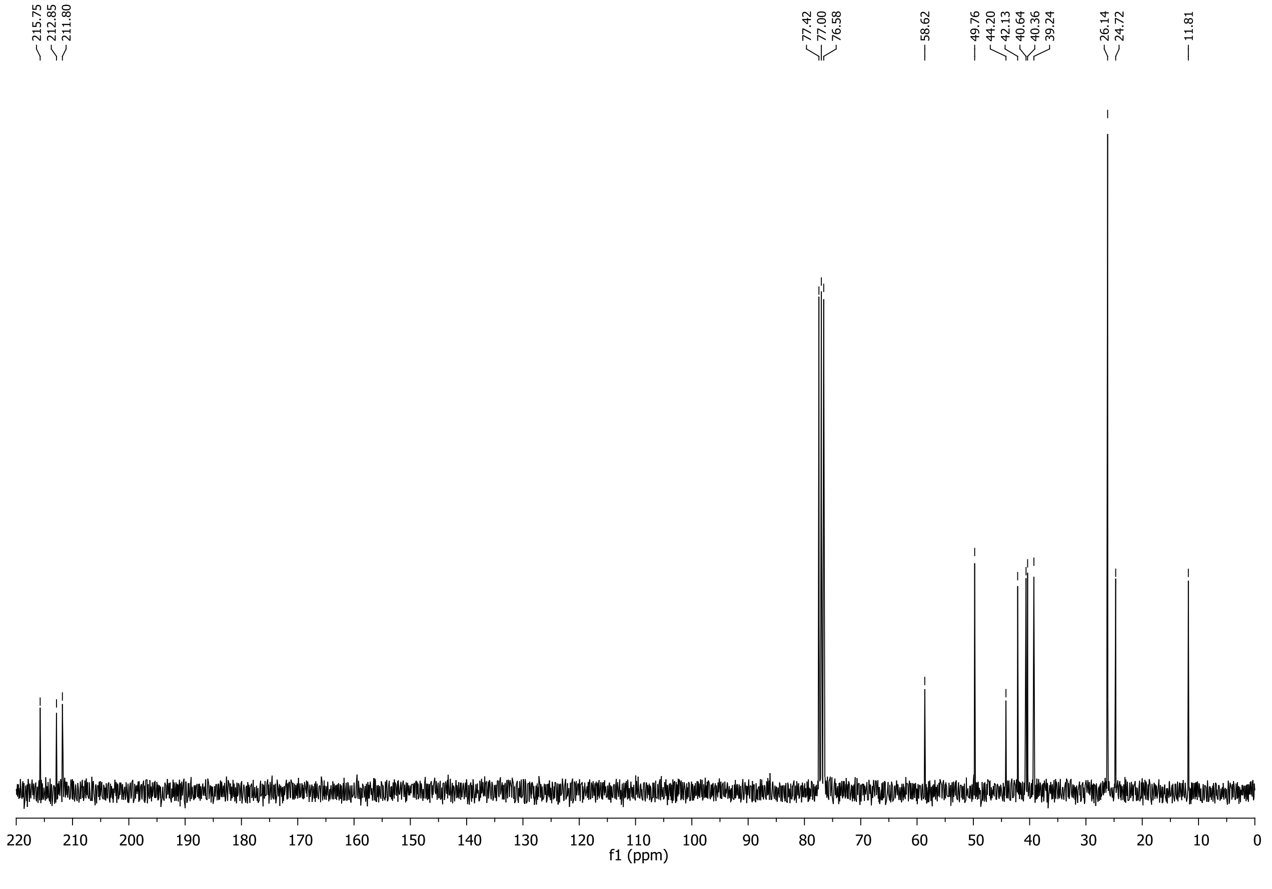
215.75
212.85
211.80
77.42
77.00
76.58
58.62
49.76
44.20
42.13
40.64
40.36
39.24
26.14
24.72
11.81
220 210 200 190 180 170 160 150 140 130 120 110 100 90 80 70 60 50 40 30 20 10 0
f1 (ppm)

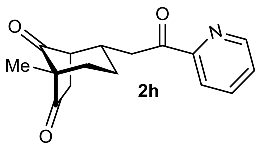
Me
O
N

2h

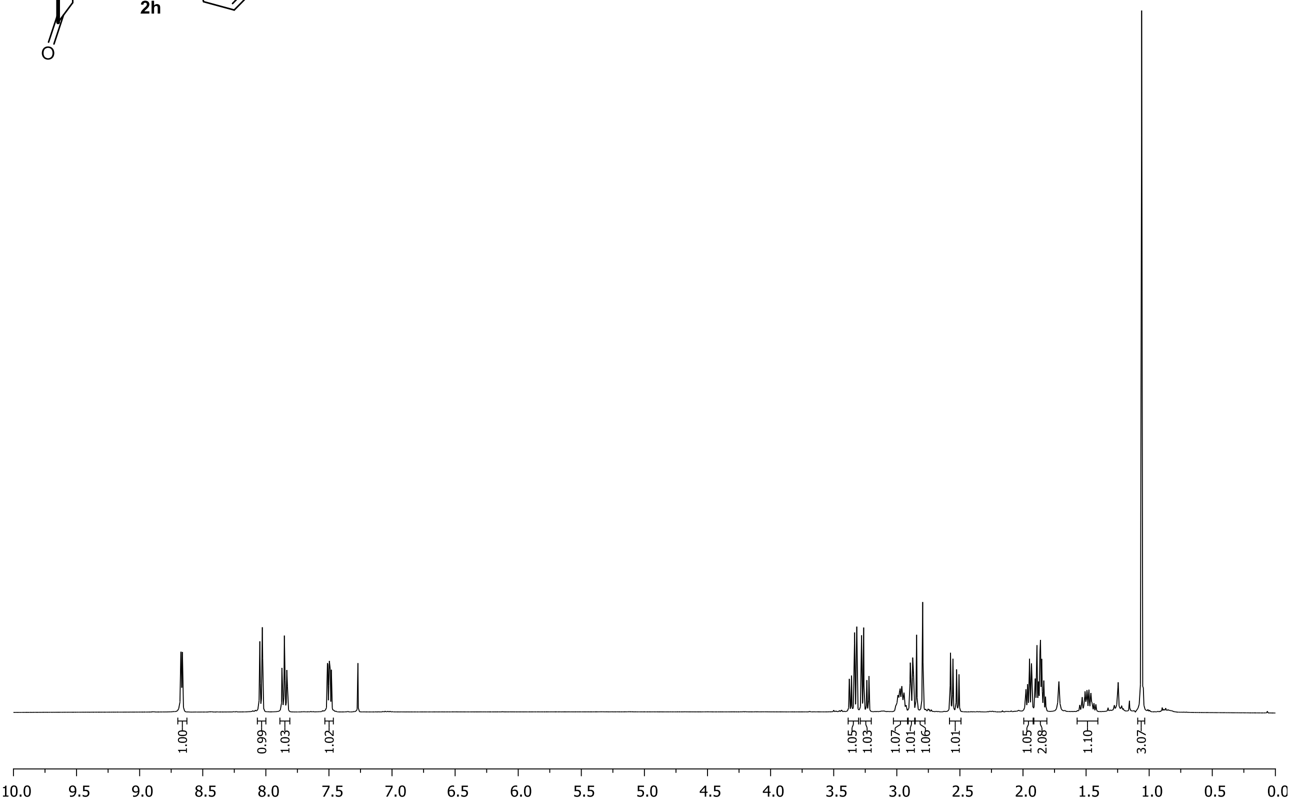
1.00
0.99
1.03
1.02
1.05
1.03
1.07
1.01
1.06
1.01
1.05
2.08
1.10
3.07
10.0 9.5 9.0 8.5 8.0 7.5 7.0 6.5 6.0 5.5 5.0 4.5 4.0 3.5 3.0 2.5 2.0 1.5 1.0 0.5 0.0

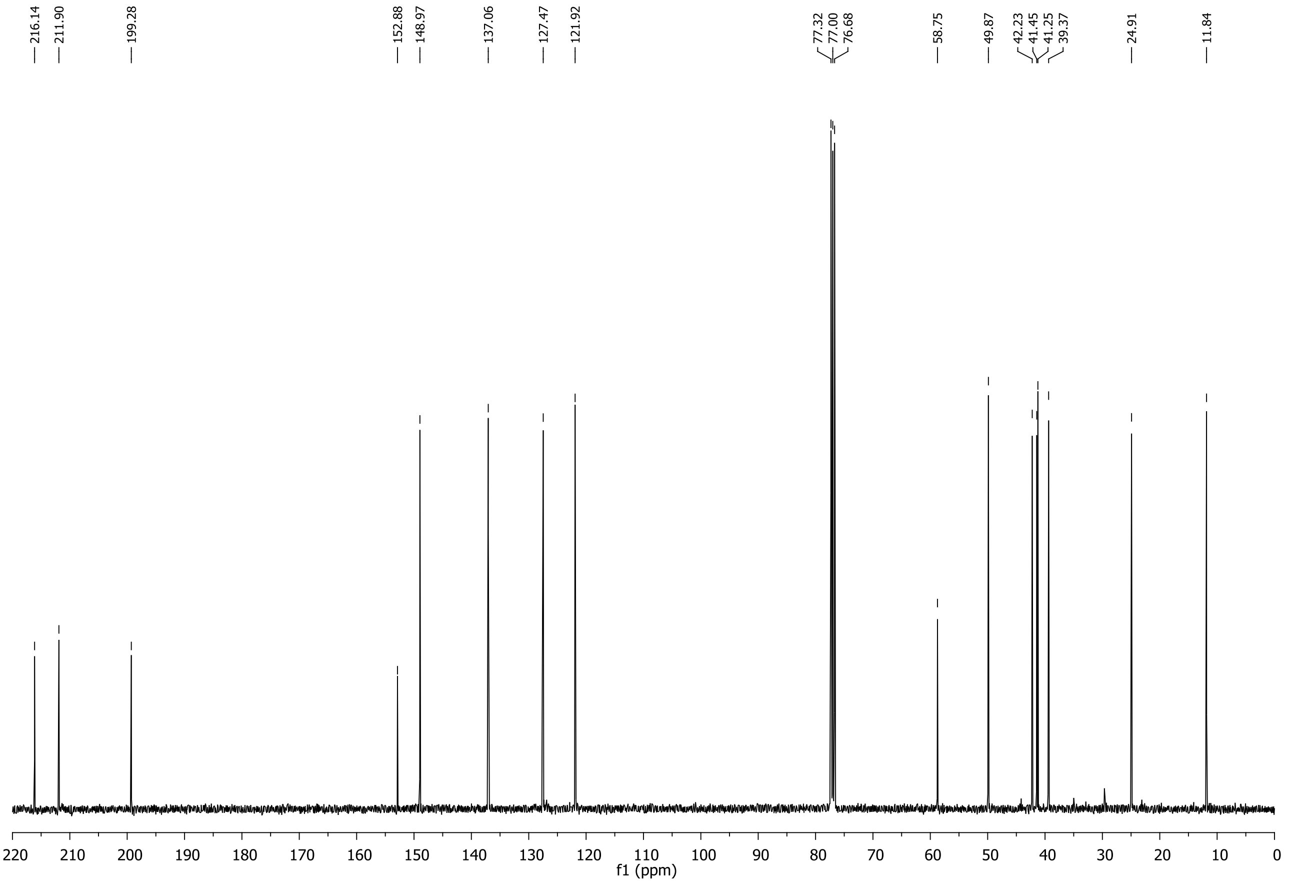
216.14
211.90
199.28
152.88
148.97
137.06
127.47
121.92
77.32
77.00
76.68
58.75
49.87
42.23
41.45
41.25
39.37
24.91
11.84
220 210 200 190 180 170 160 150 140 130 120 110 100 90 80 70 60 50 40 30 20 10 0
f1 (ppm)

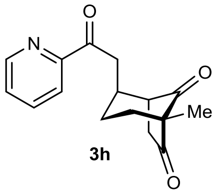
O
N
O
Me
O

3h

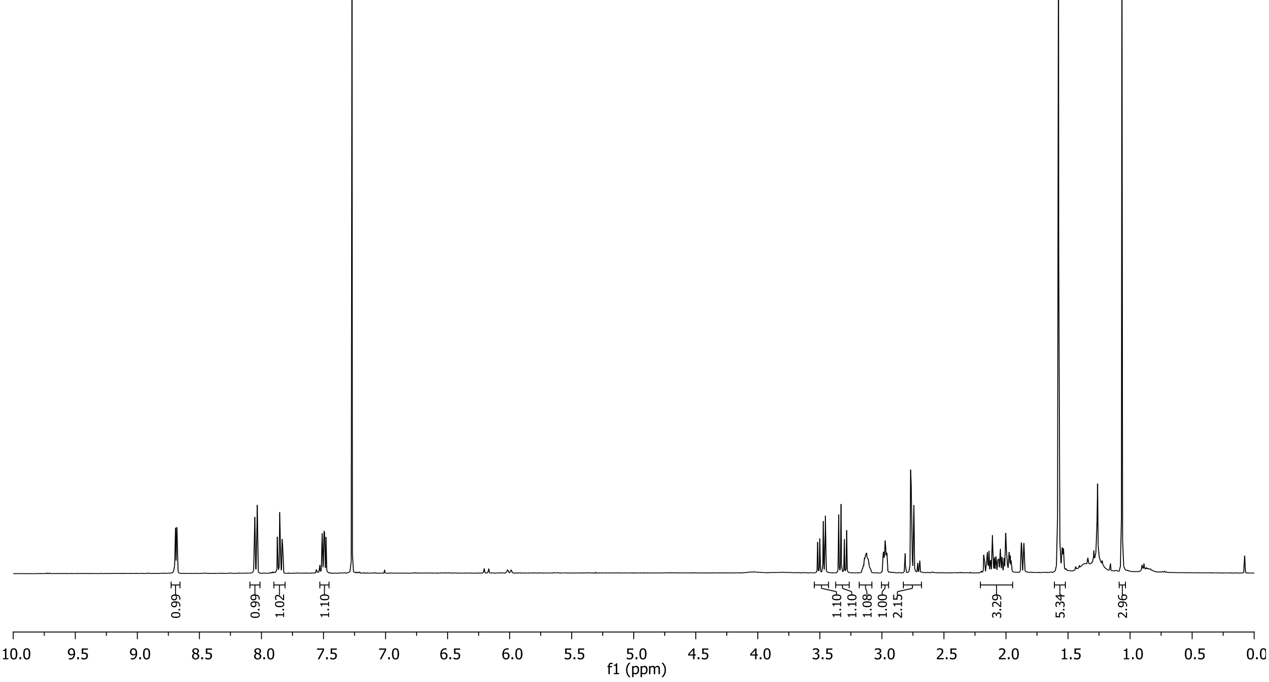
0.99
0.99
1.02
1.10
1.10
1.10
1.08
1.00
2.15
3.29
5.34
2.96
10.0 9.5 9.0 8.5 8.0 7.5 7.0 6.5 6.0 5.5 5.0 4.5 4.0 3.5 3.0 2.5 2.0 1.5 1.0 0.5 0.0
f1 (ppm)

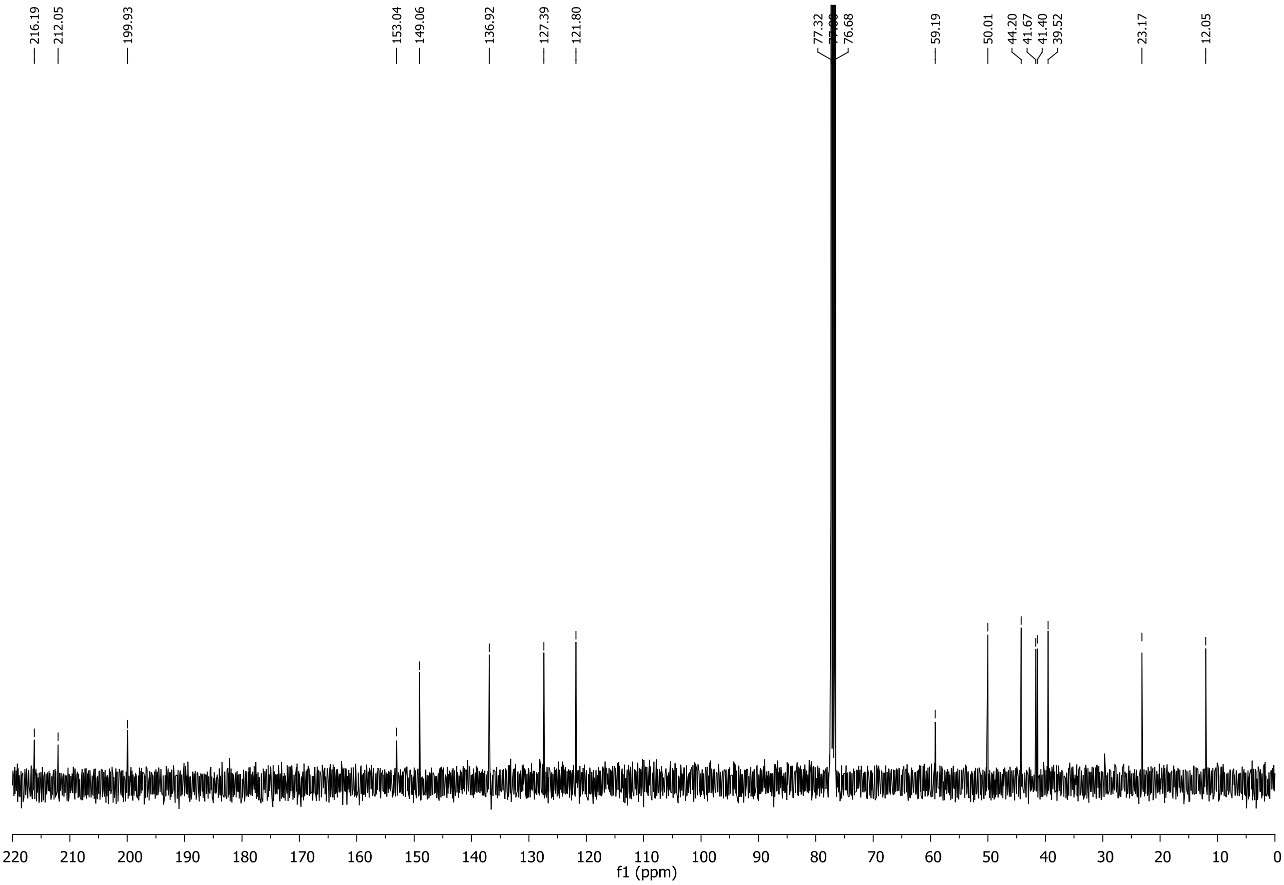
216.19
212.05
199.93
153.04
149.06
136.92
127.39
121.80
77.32
76.68
59.19
50.01
44.20
41.67
41.40
39.52
23.17
12.05
220 210 200 190 180 170 160 150 140 130 120 110 100 90 80 70 60 50 40 30 20 10 0
f1 (ppm)

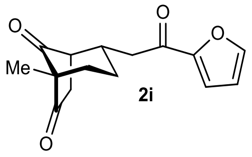
Me
2i

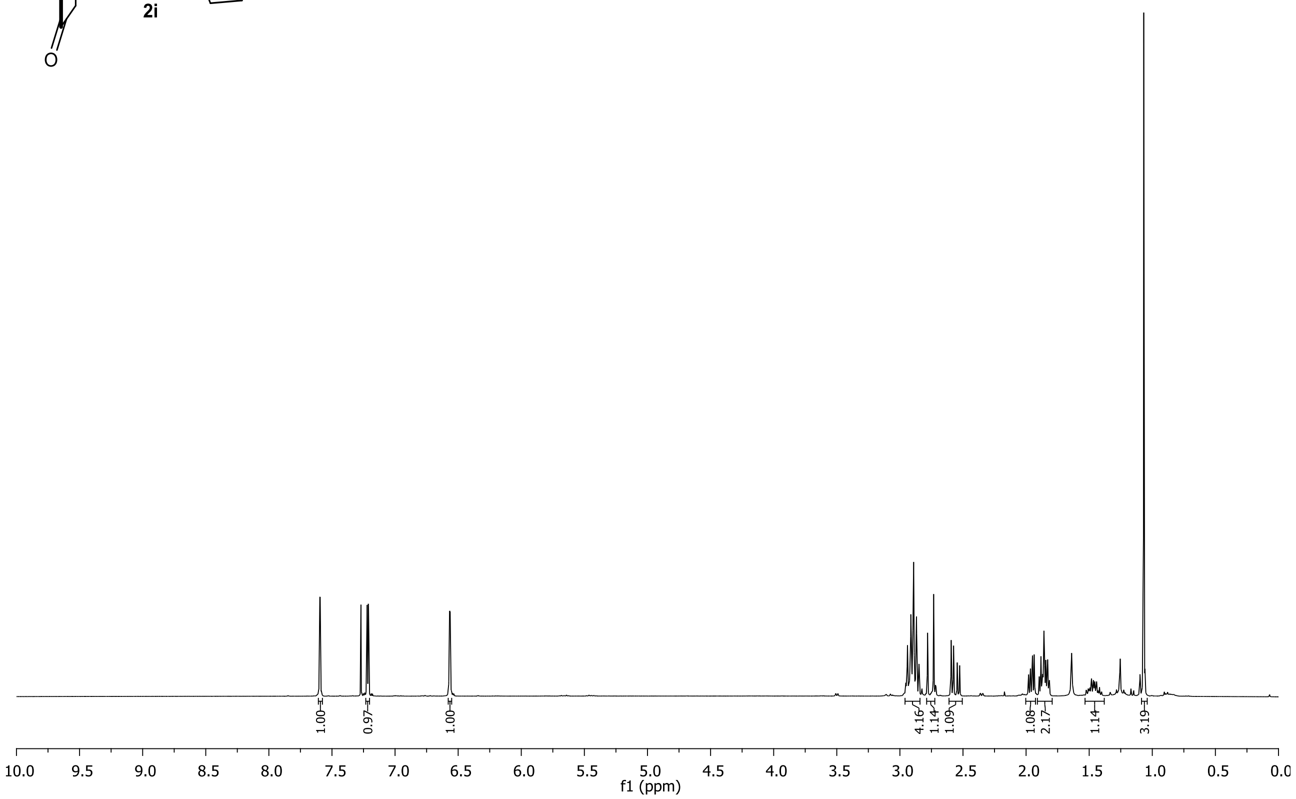

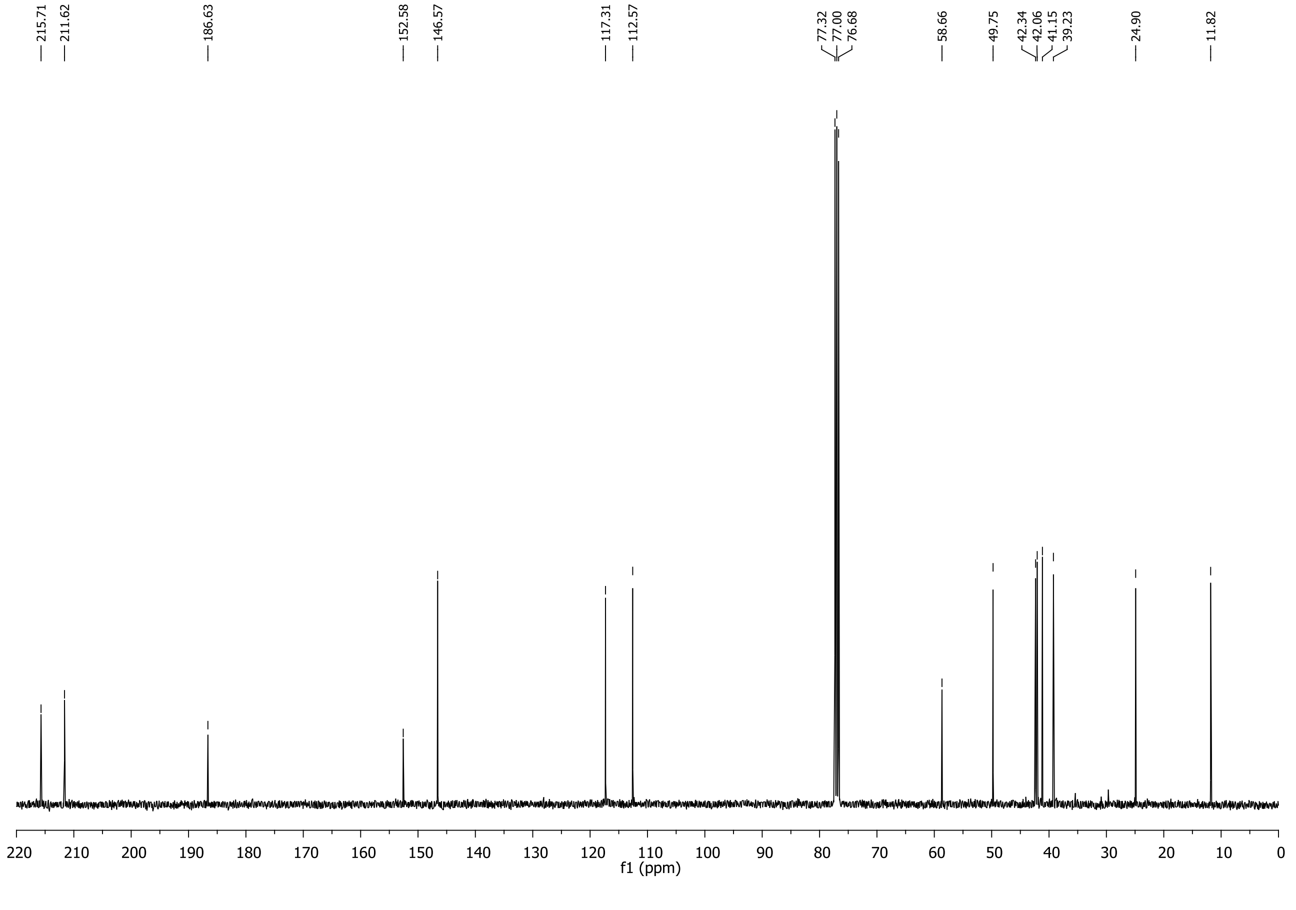

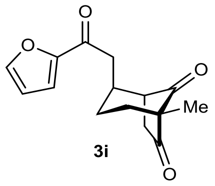
O
O
O
Me
O
3i

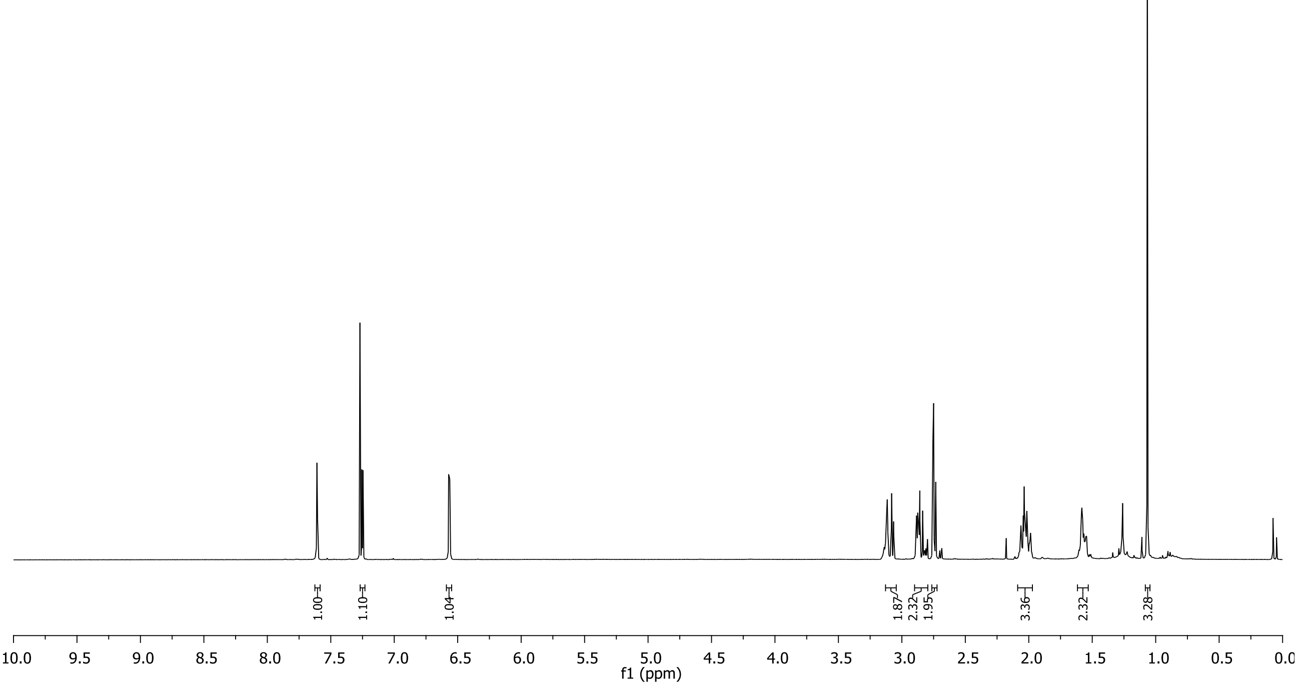
1.00
1.10
1.04
1.87
2.32
1.95
3.36
2.32
3.28
10.0
9.5
9.0
8.5
8.0
7.5
7.0
6.5
6.0
5.5
5.0
4.5
4.0
3.5
3.0
2.5
2.0
1.5
1.0
0.5
0.0
f1 (ppm)

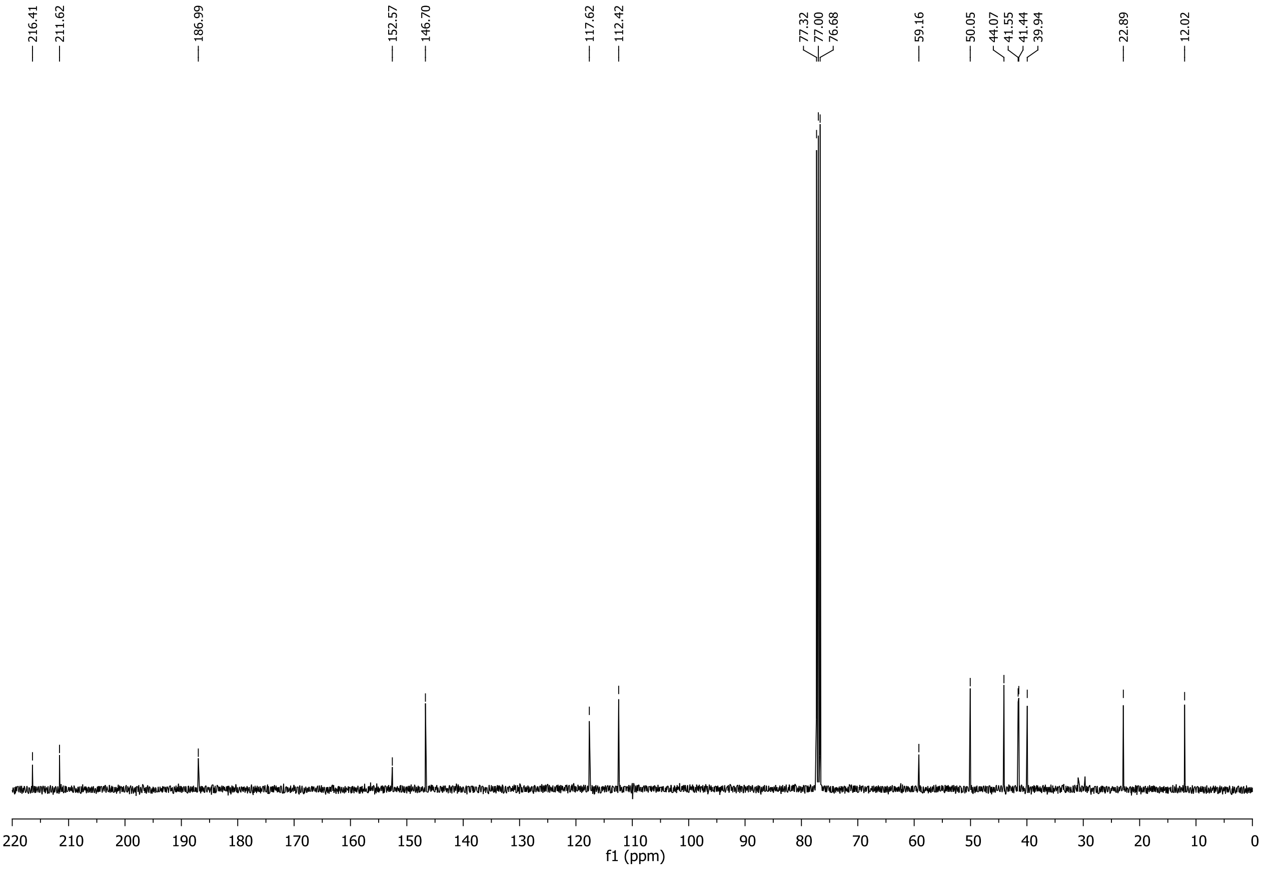
216.41
211.62
186.99
152.57
146.70
117.62
112.42
77.32
77.00
76.68
59.16
50.05
44.07
41.55
41.44
39.94
22.89
12.02
220
210
200
190
180
170
160
150
140
130
120
110
100
90
80
70
60
50
40
30
20
10
0
f1 (ppm)

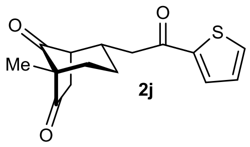
O
O
S
Me
O

2j

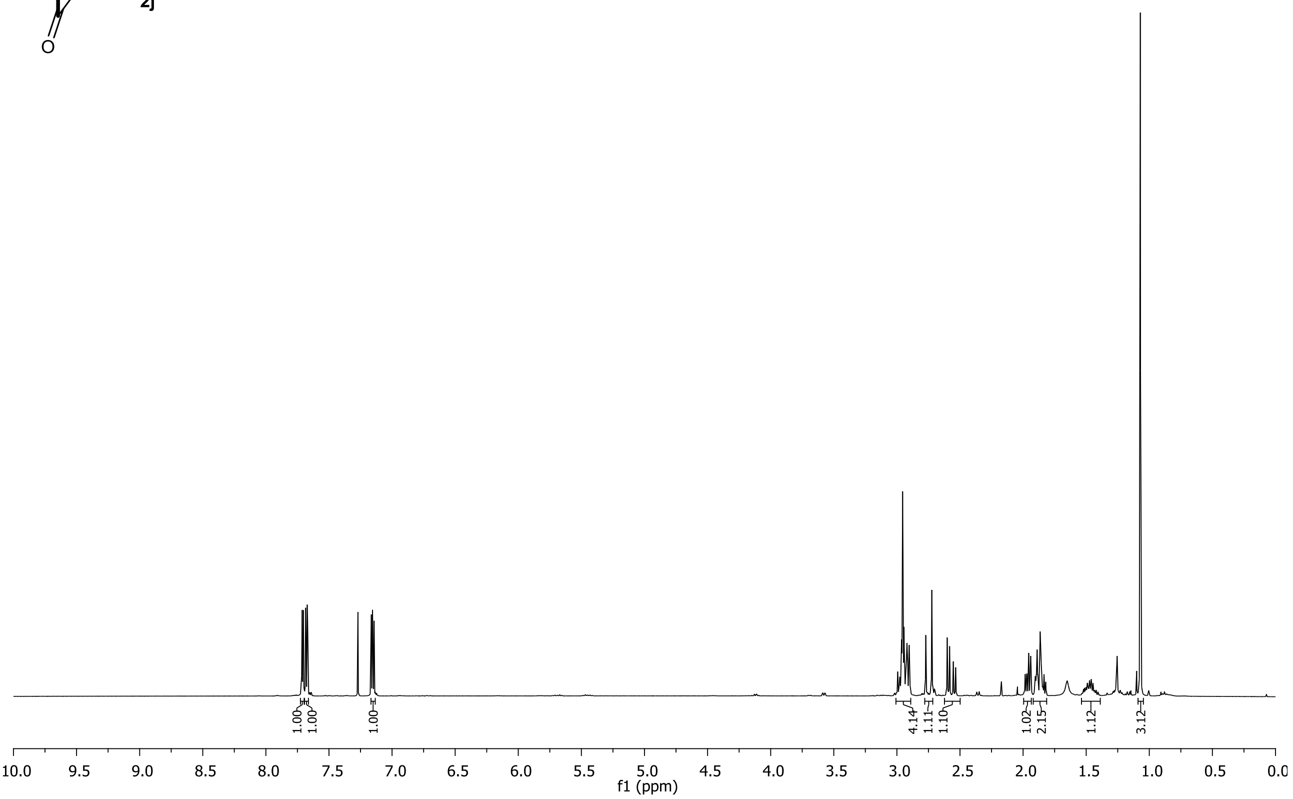
1.00
1.00
1.00
4.14
1.11
1.10
1.02
2.15
1.12
3.12
10.0 9.5 9.0 8.5 8.0 7.5 7.0 6.5 6.0 5.5 5.0 4.5 4.0 3.5 3.0 2.5 2.0 1.5 1.0 0.5 0.0
f1 (ppm)

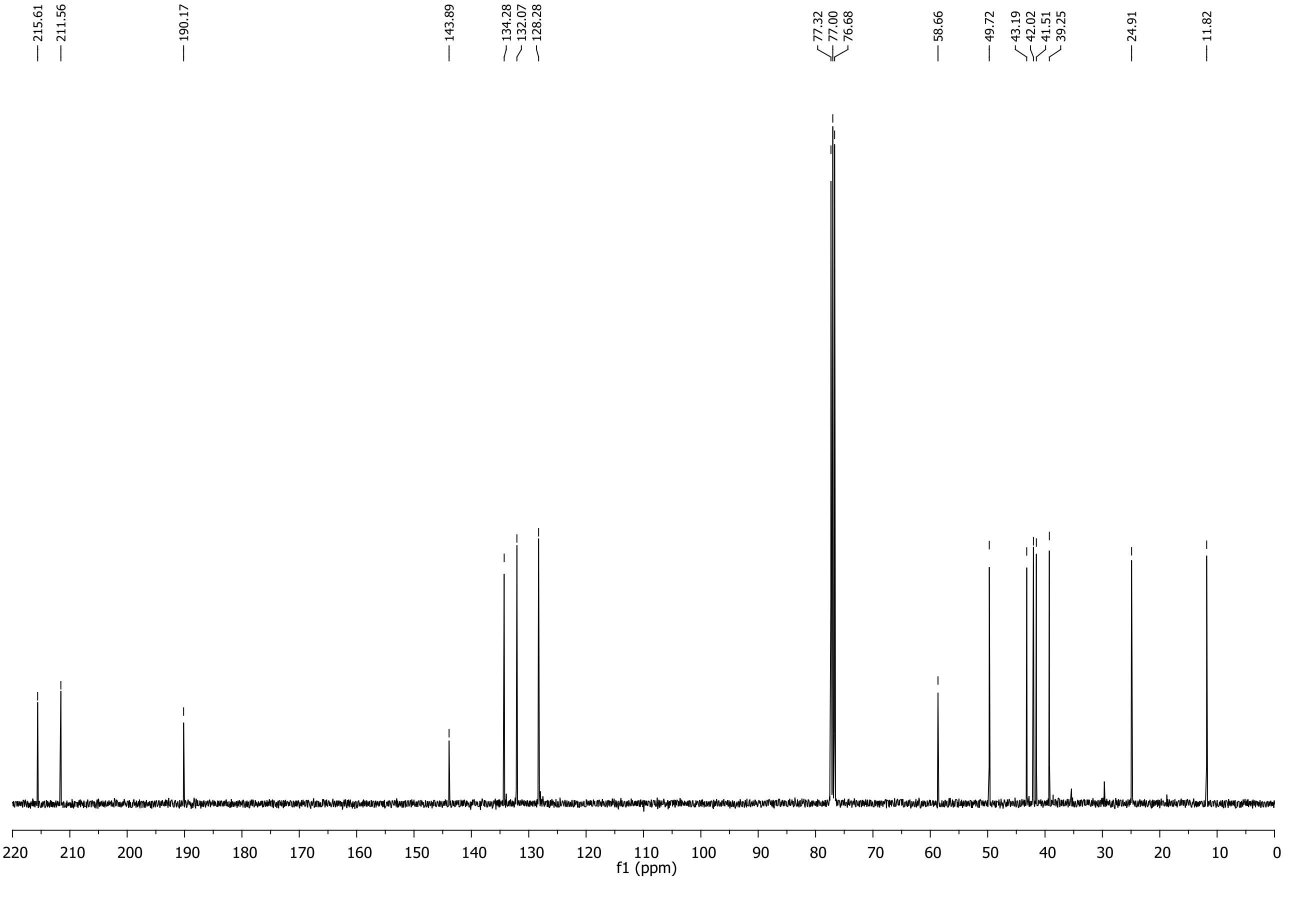
215.61
211.56
190.17
143.89
134.28
132.07
128.28
77.32
77.00
76.68
58.66
49.72
43.19
42.02
41.51
39.25
24.91
11.82
220 210 200 190 180 170 160 150 140 130 120 110 100 90 80 70 60 50 40 30 20 10 0
f1 (ppm)

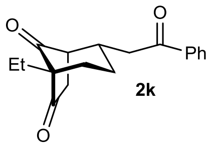

2k

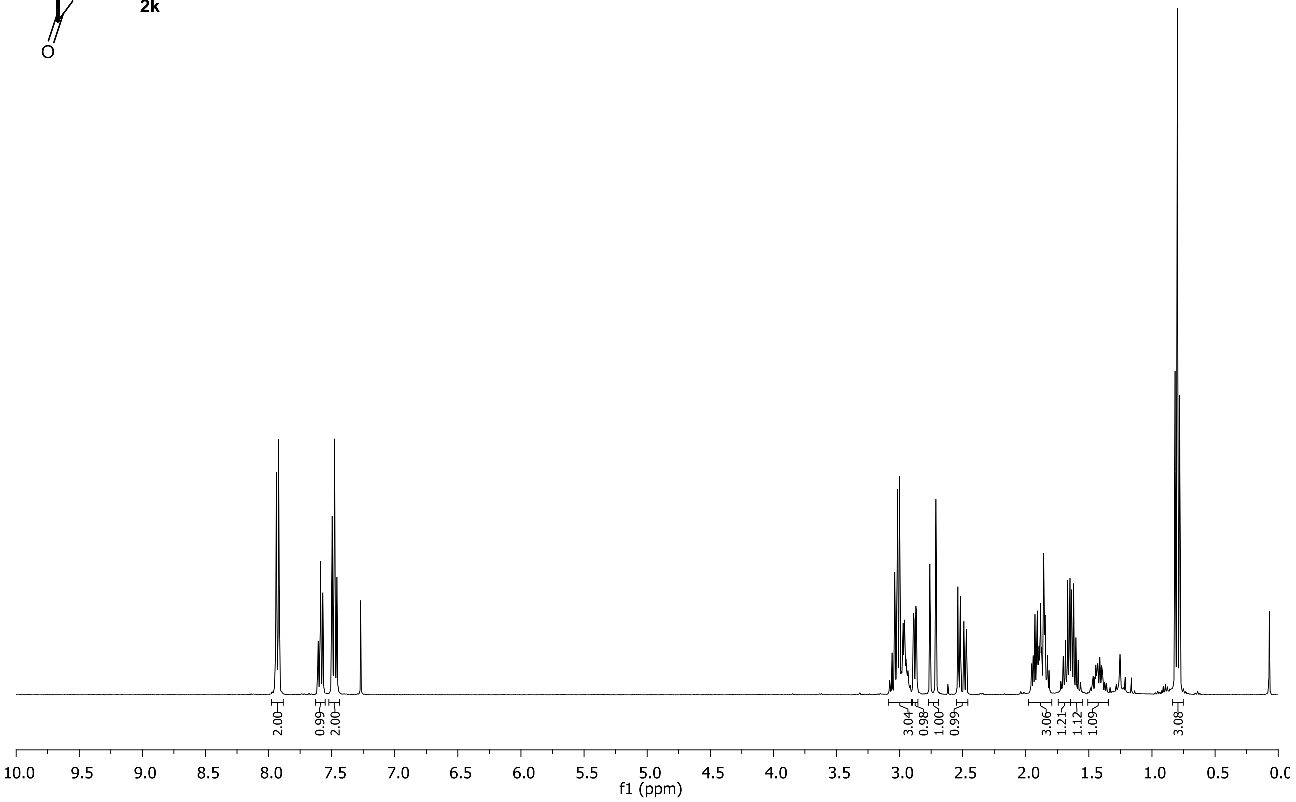
2.00
0.99
2.00
3.04
0.98
1.00
0.99
3.06
1.21
1.12
1.09
3.08
10.0 9.5 9.0 8.5 8.0 7.5 7.0 6.5 6.0 5.5 5.0 4.5 4.0 3.5 3.0 2.5 2.0 1.5 1.0 0.5 0.0
f1 (ppm)

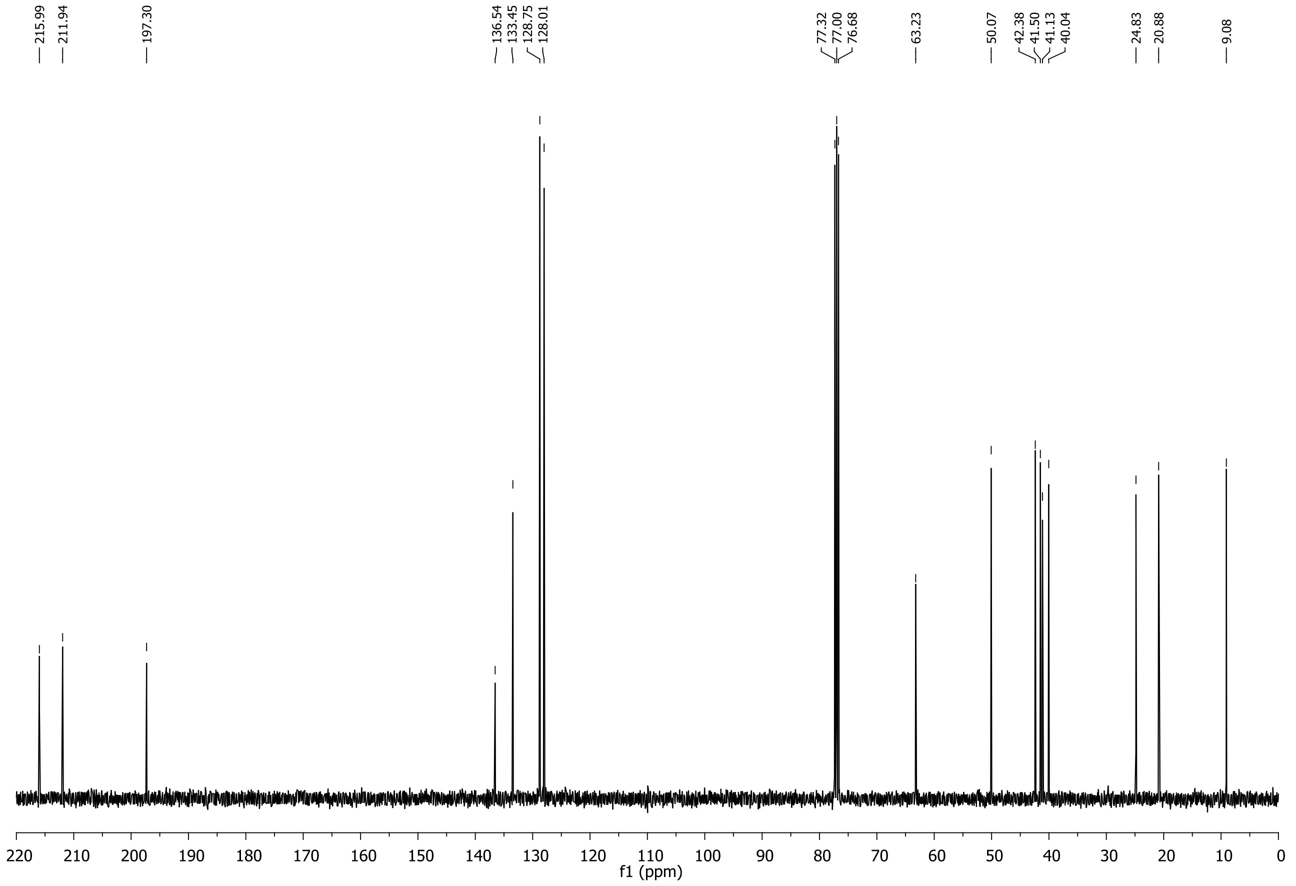
215.99
211.94
197.30
136.54
133.45
128.75
128.01
77.32
77.00
76.68
63.23
50.07
42.38
41.50
41.13
40.04
24.83
20.88
9.08
220 210 200 190 180 170 160 150 140 130 120 110 100 90 80 70 60 50 40 30 20 10 0
f1 (ppm)

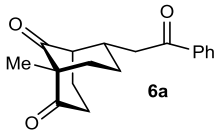
O
O
Me
O
Ph

6a

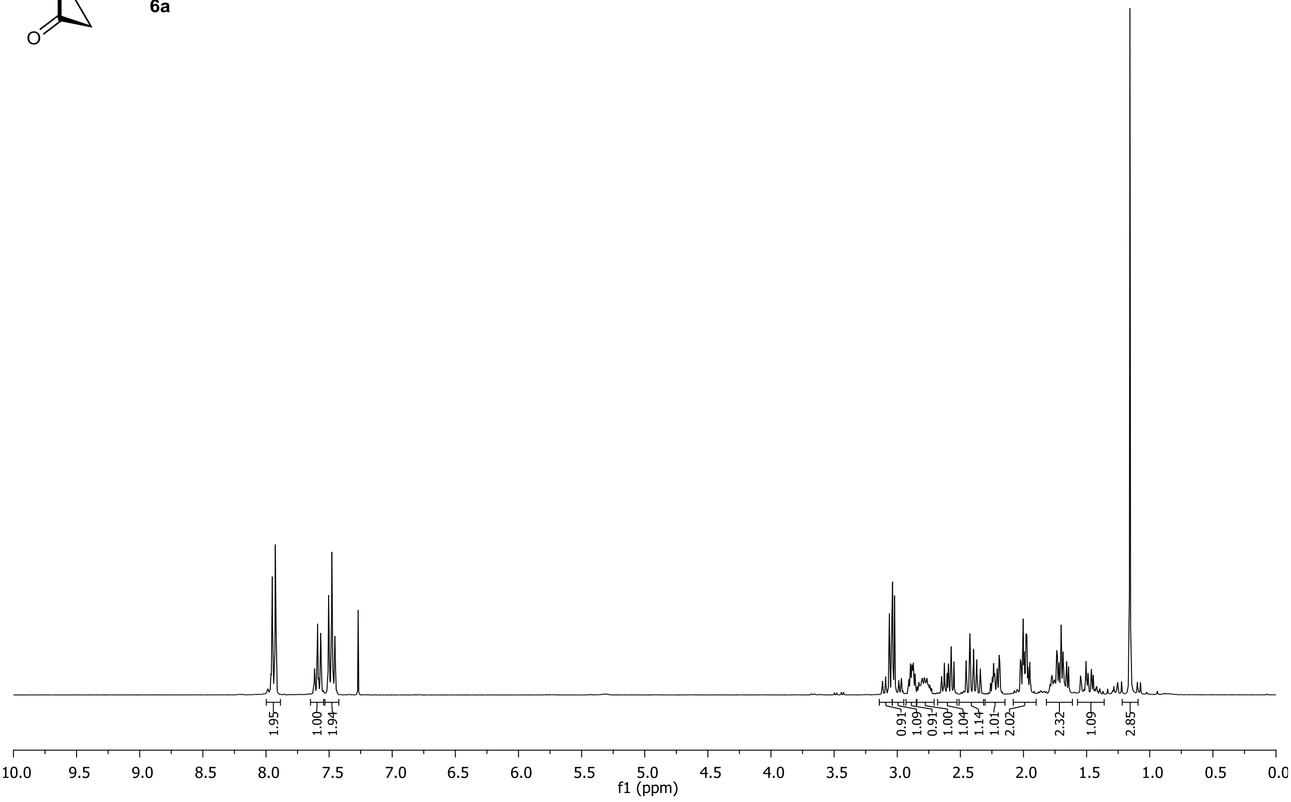
1.95
1.00
1.94
0.91
1.09
0.91
1.00
1.04
1.14
1.01
2.02
2.32
1.09
2.85
10.0 9.5 9.0 8.5 8.0 7.5 7.0 6.5 6.0 5.5 5.0 4.5 4.0 3.5 3.0 2.5 2.0 1.5 1.0 0.5 0.0
f1 (ppm)

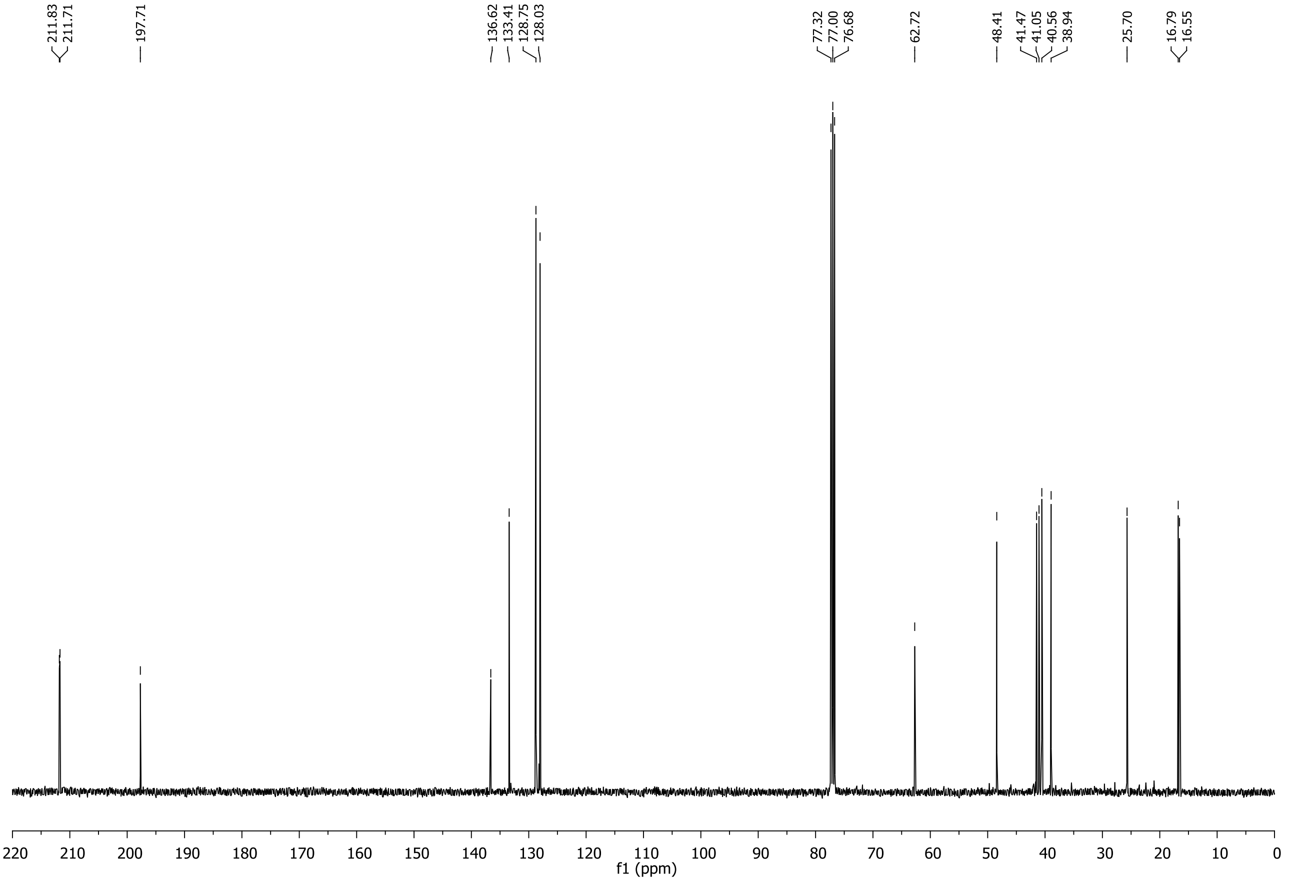
211.83
211.71
197.71
136.62
133.41
128.75
128.03
77.32
77.00
76.68
62.72
48.41
41.47
41.05
40.56
38.94
25.70
16.79
16.55
220 210 200 190 180 170 160 150 140 130 120 110 100 90 80 70 60 50 40 30 20 10 0
f1 (ppm)

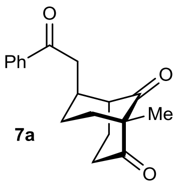
O
Ph
O
Me
O

7a

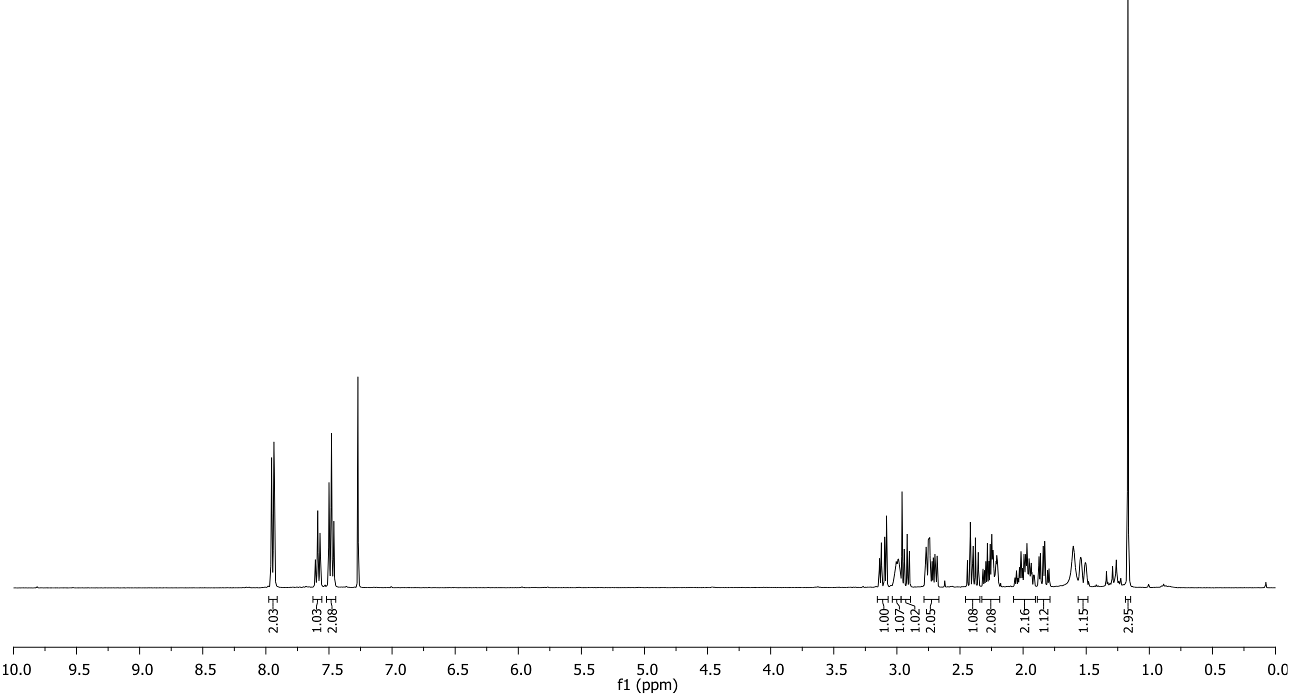
2.03
1.03
2.08
1.00
1.07
1.02
2.05
1.08
2.08
2.16
1.12
1.15
2.95
10.0 9.5 9.0 8.5 8.0 7.5 7.0 6.5 6.0 5.5 5.0 4.5 4.0 3.5 3.0 2.5 2.0 1.5 1.0 0.5 0.0
f1 (ppm)

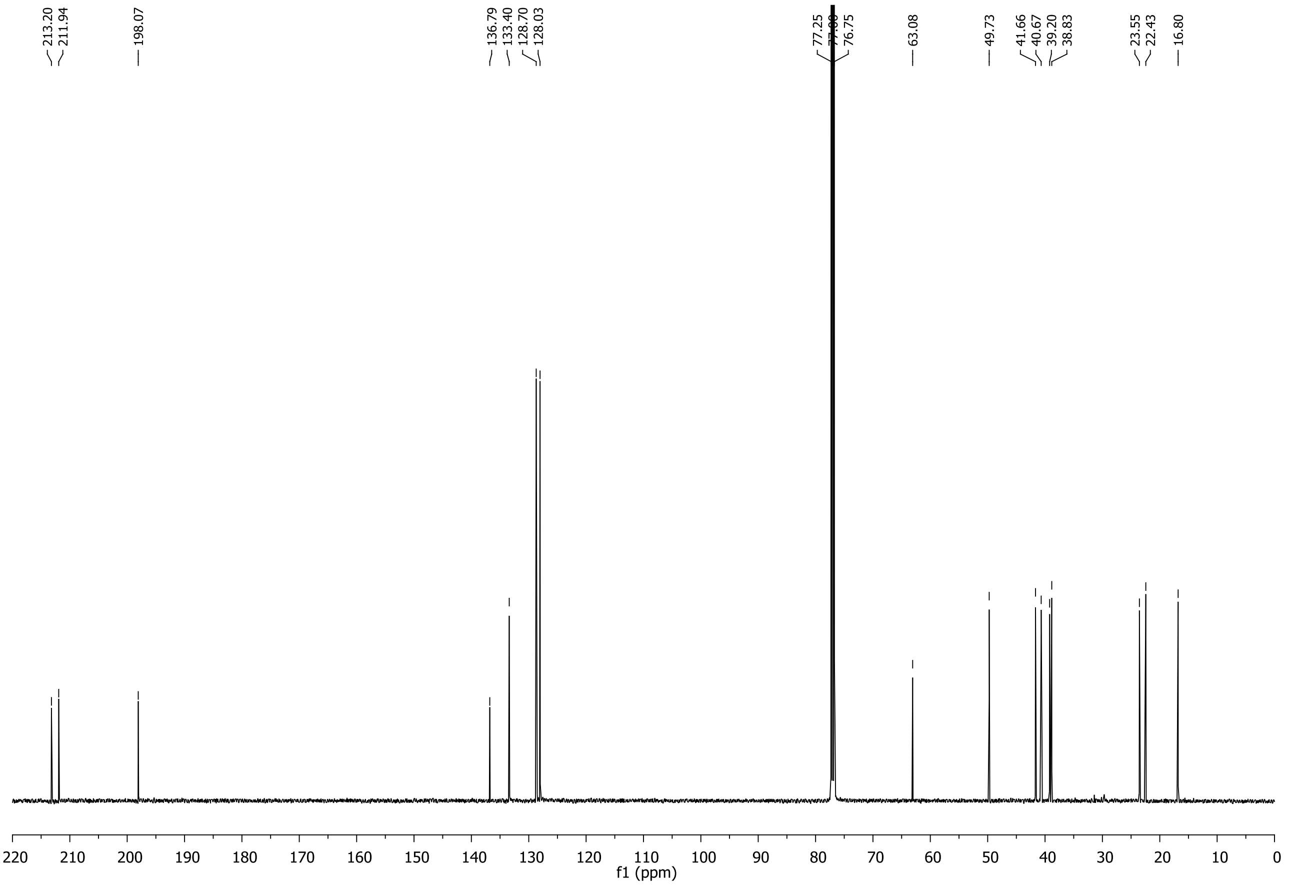
213.20
211.94
198.07
136.79
133.40
128.70
128.03
77.25
76.75
63.08
49.73
41.66
40.67
39.20
38.83
23.55
22.43
16.80
220 210 200 190 180 170 160 150 140 130 120 110 100 90 80 70 60 50 40 30 20 10 0
f1 (ppm)

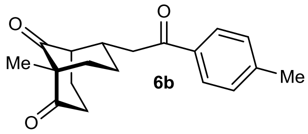
O
Me
O
6b
O
Me

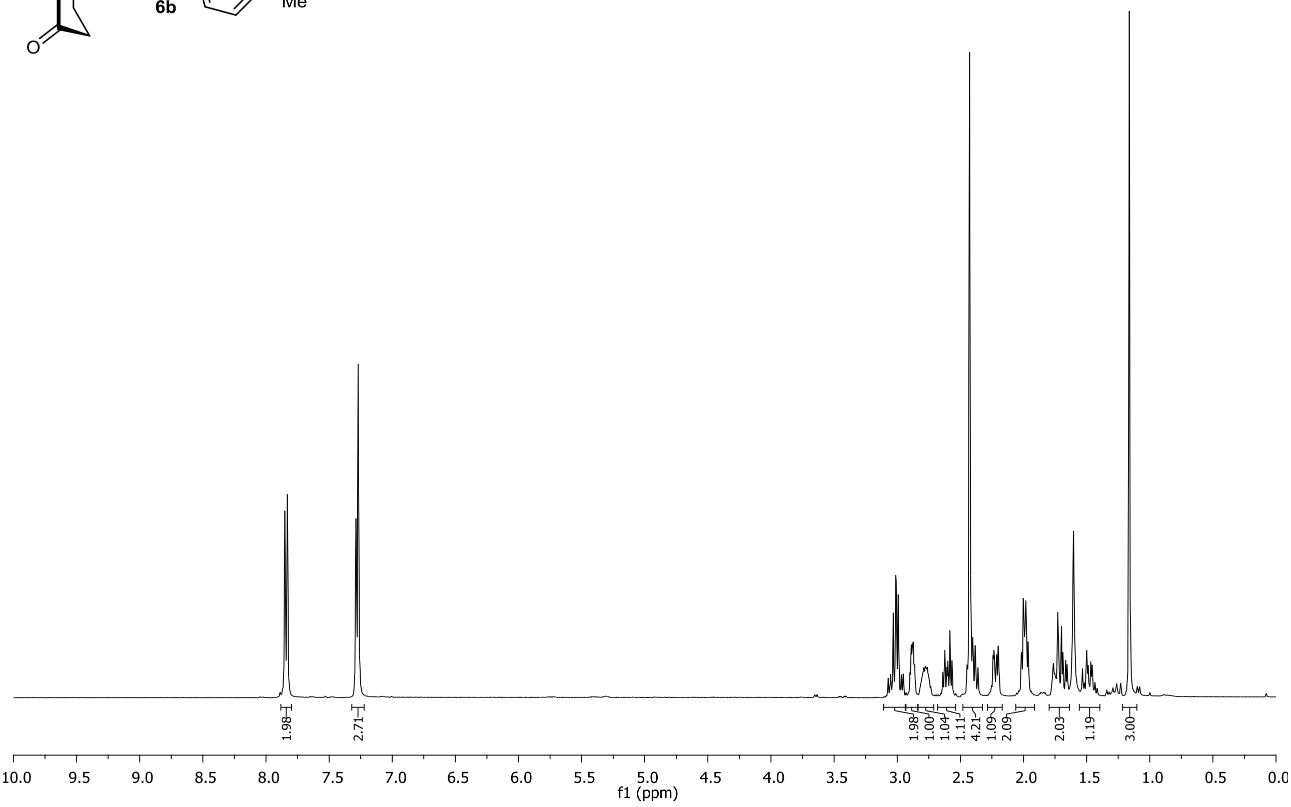
1.98
2.71
1.98
1.00
1.04
1.11
4.21
1.09
2.09
2.03
1.19
3.00
10.0 9.5 9.0 8.5 8.0 7.5 7.0 6.5 6.0 5.5 5.0 4.5 4.0 3.5 3.0 2.5 2.0 1.5 1.0 0.5 0.0
f1 (ppm)

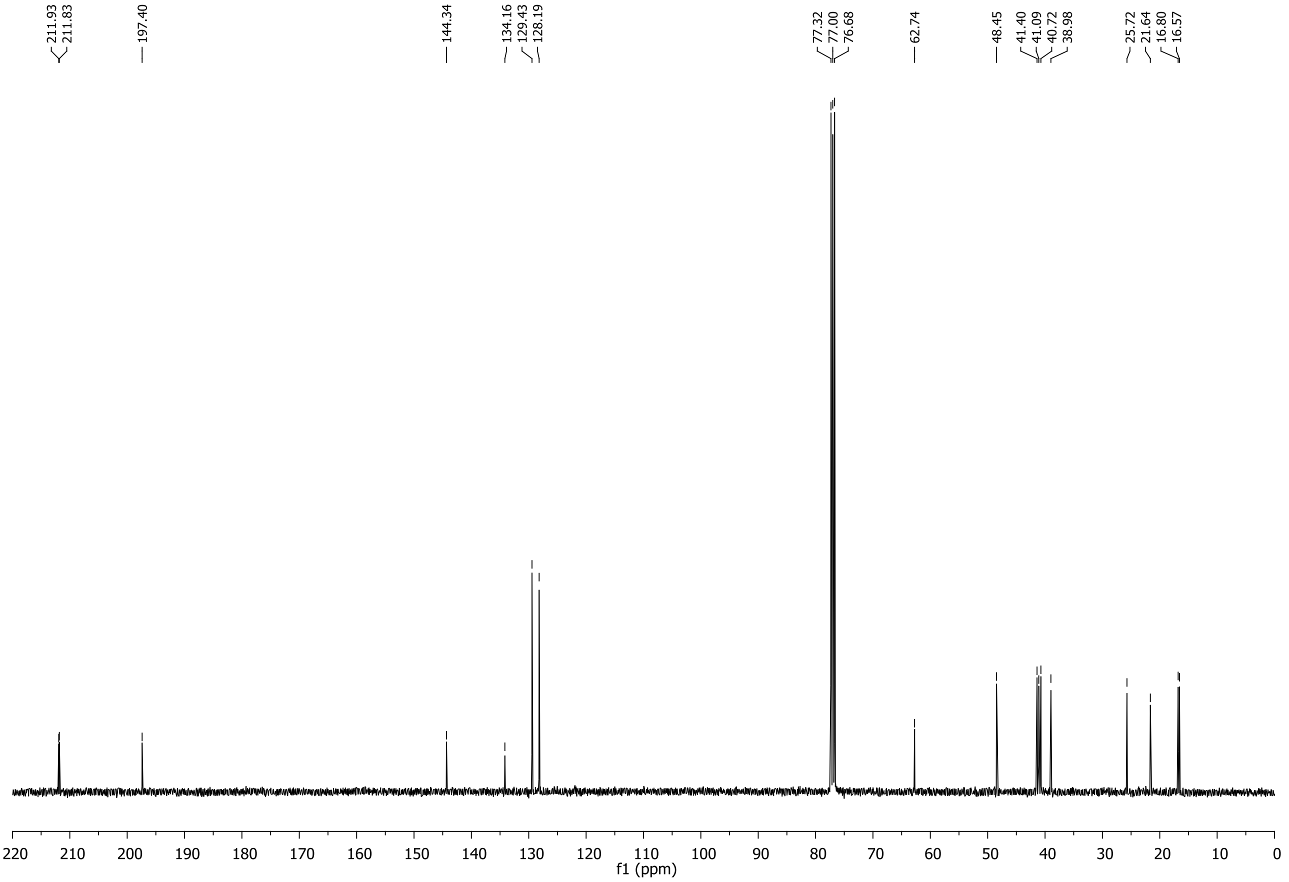
211.93
211.83
197.40
144.34
134.16
129.43
128.19
77.32
77.00
76.68
62.74
48.45
41.40
41.09
40.72
38.98
25.72
21.64
16.80
16.57
220 210 200 190 180 170 160 150 140 130 120 110 100 90 80 70 60 50 40 30 20 10 0
f1 (ppm)

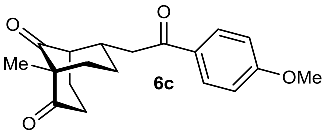
O
O
Me
O
6c
OMe

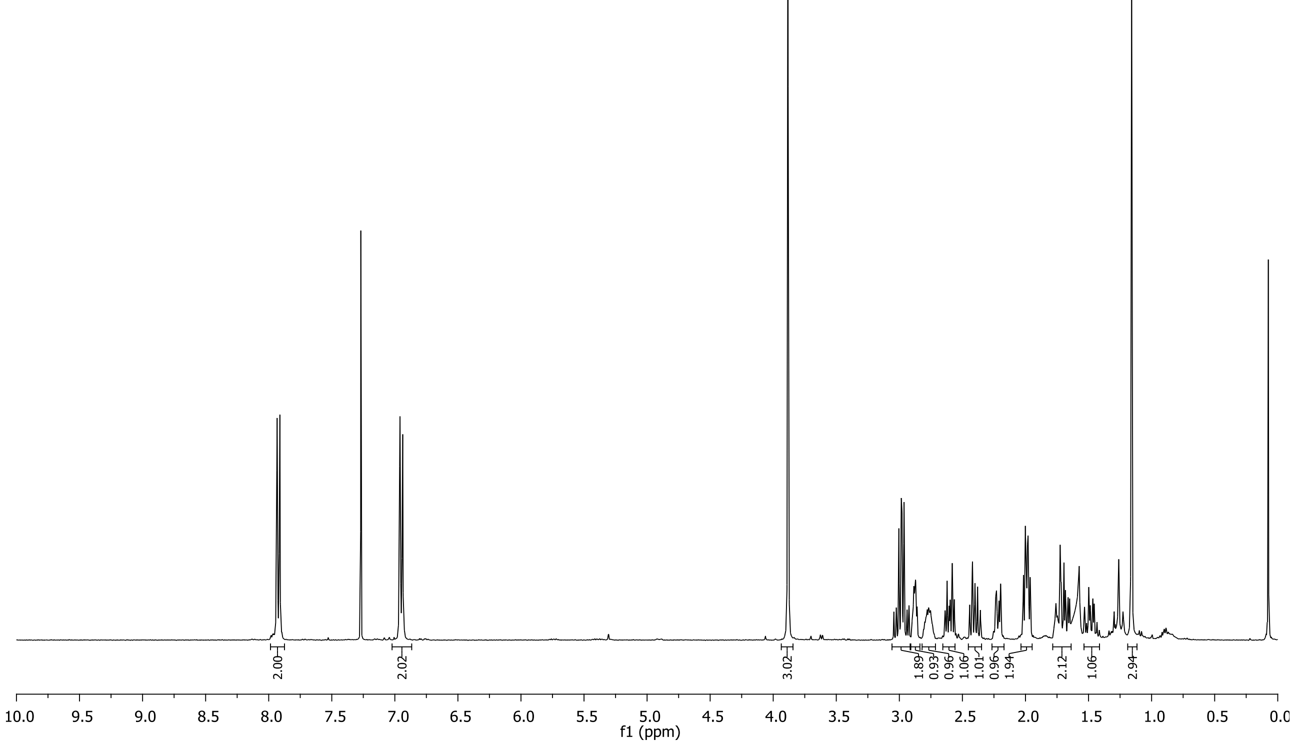
2.00
2.02
3.02
1.89
0.93
0.96
1.06
1.01
0.96
1.94
2.12
1.06
2.94
f1 (ppm)

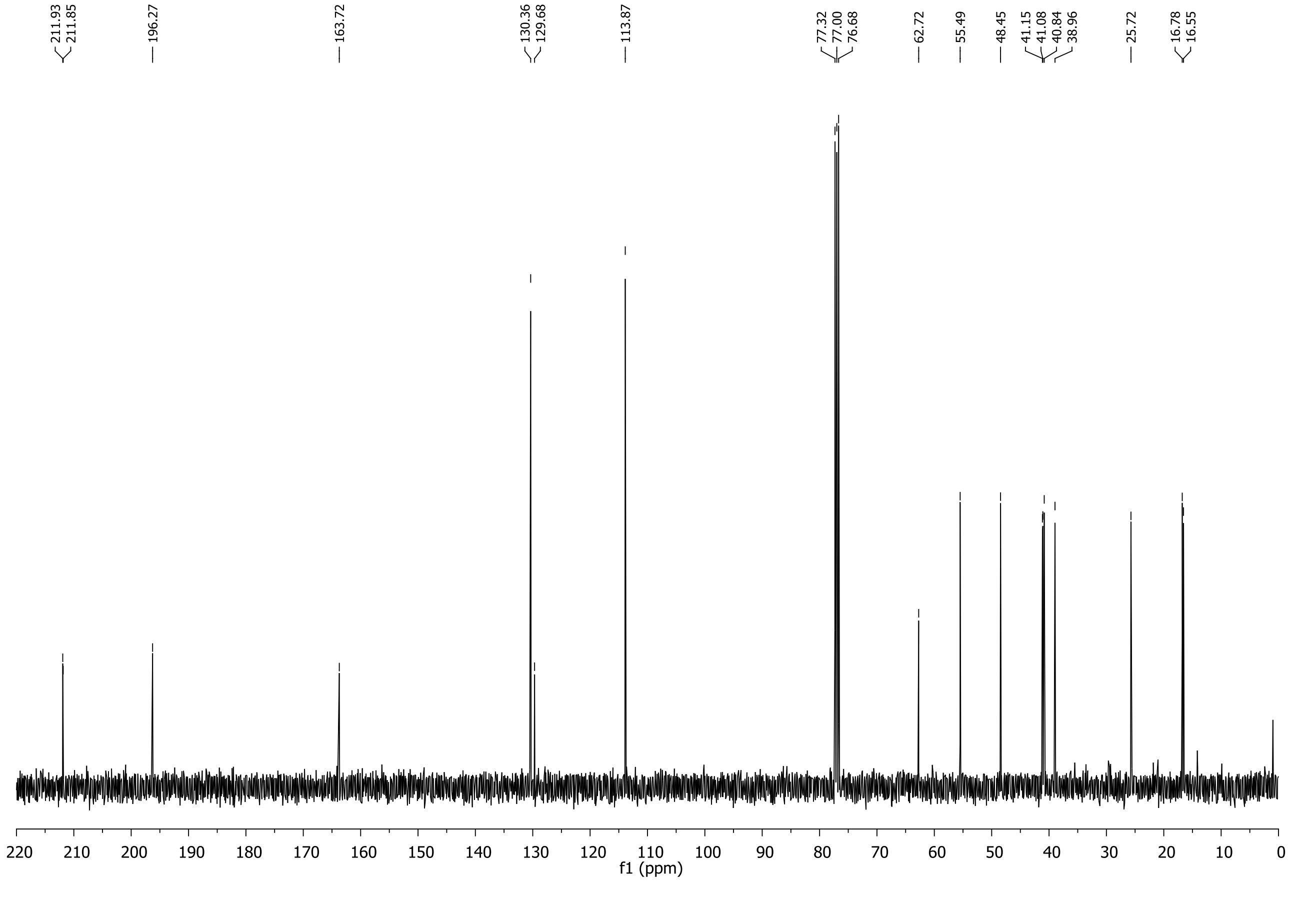
211.93
211.85
196.27
163.72
130.36
129.68
113.87
77.32
77.00
76.68
62.72
55.49
48.45
41.15
41.08
40.84
38.96
25.72
16.78
16.55
f1 (ppm)

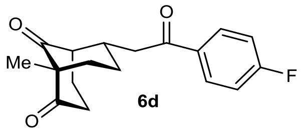

O
O
O
Me
F

6d

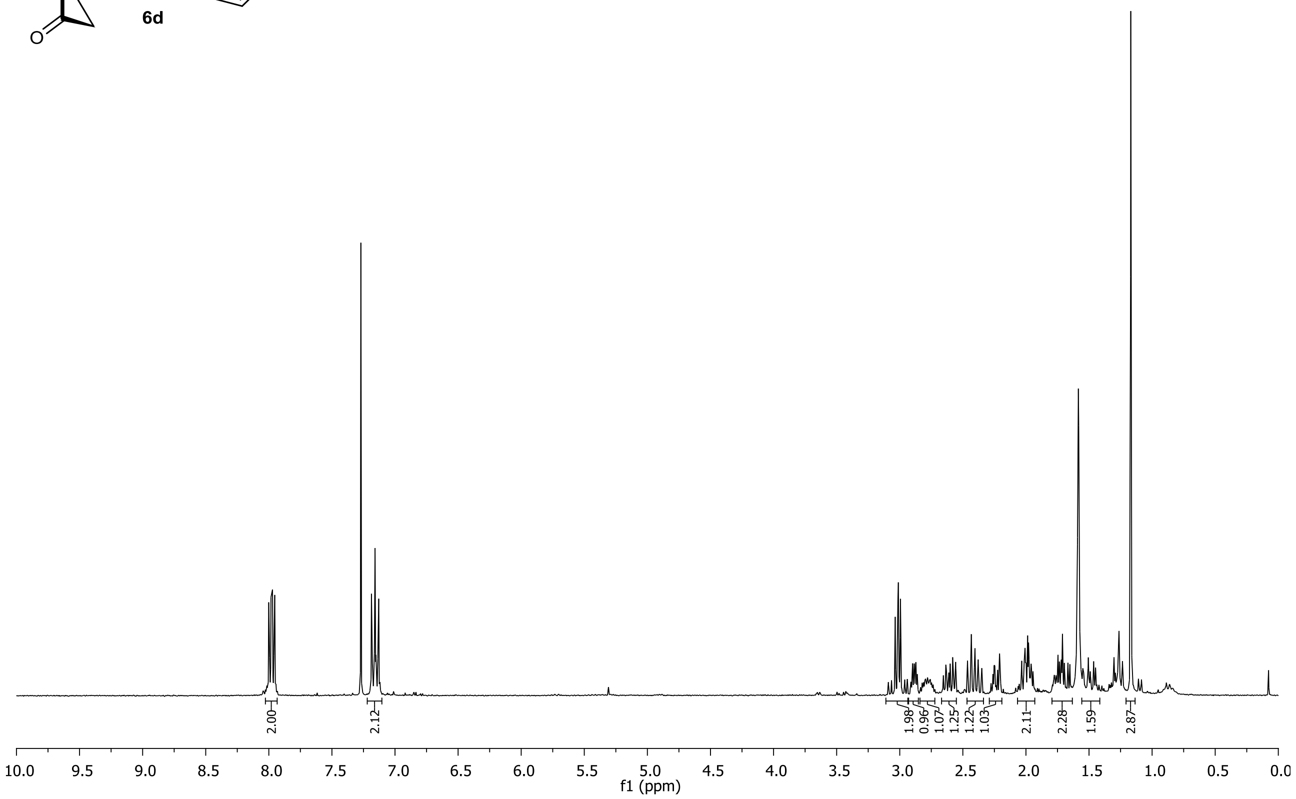

2.00
2.12
1.98
0.96
1.07
1.25
1.22
1.03
2.11
2.28
1.59
2.87
10.0 9.5 9.0 8.5 8.0 7.5 7.0 6.5 6.0 5.5 5.0 4.5 4.0 3.5 3.0 2.5 2.0 1.5 1.0 0.5 0.0
f1 (ppm)

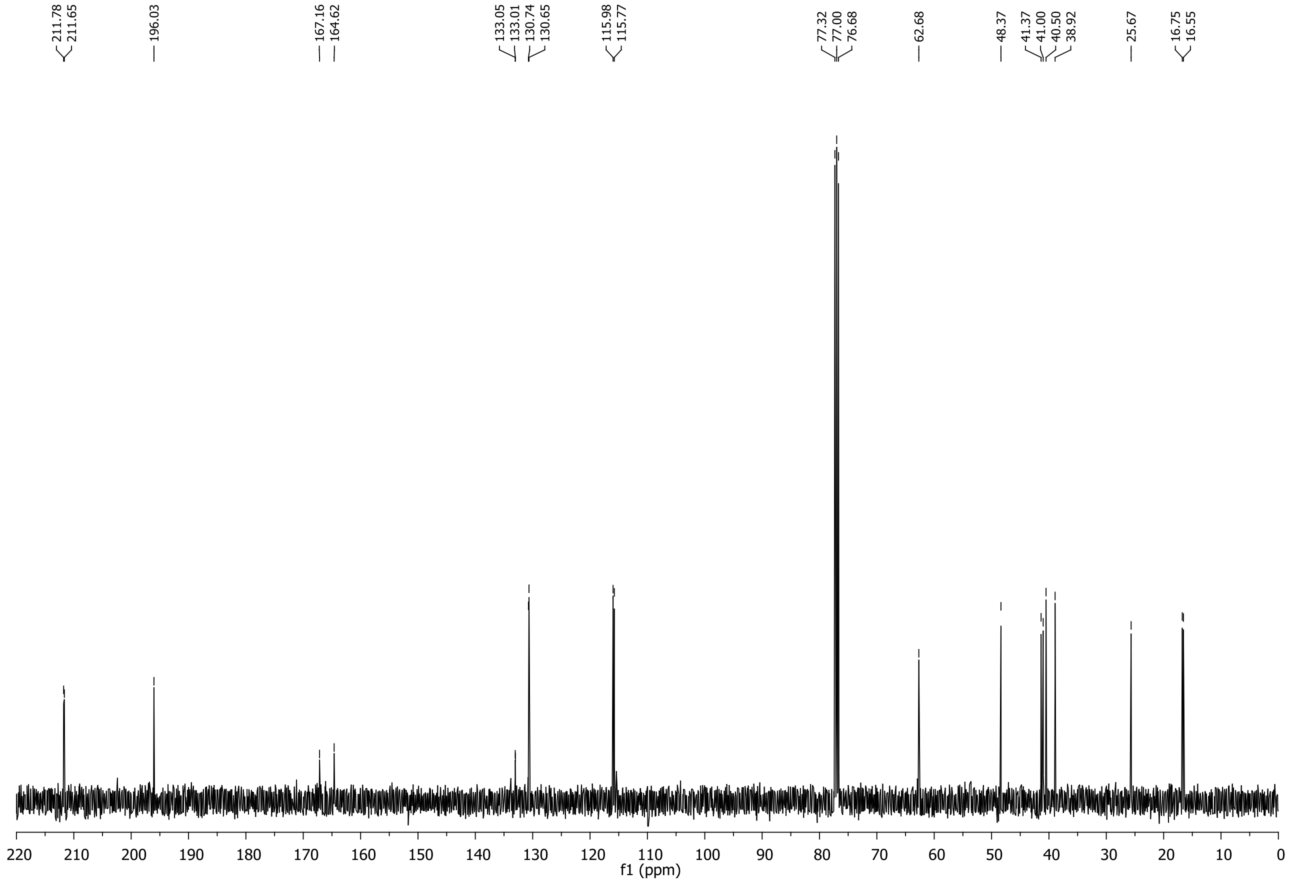

211.78
211.65
196.03
167.16
164.62
133.05
133.01
130.74
130.65
115.98
115.77
77.32
77.00
76.68
62.68
48.37
41.37
41.00
40.50
38.92
25.67
16.75
16.55
220 210 200 190 180 170 160 150 140 130 120 110 100 90 80 70 60 50 40 30 20 10 0
f1 (ppm)

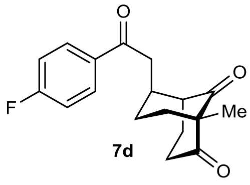
O
F
O
Me
O

7d

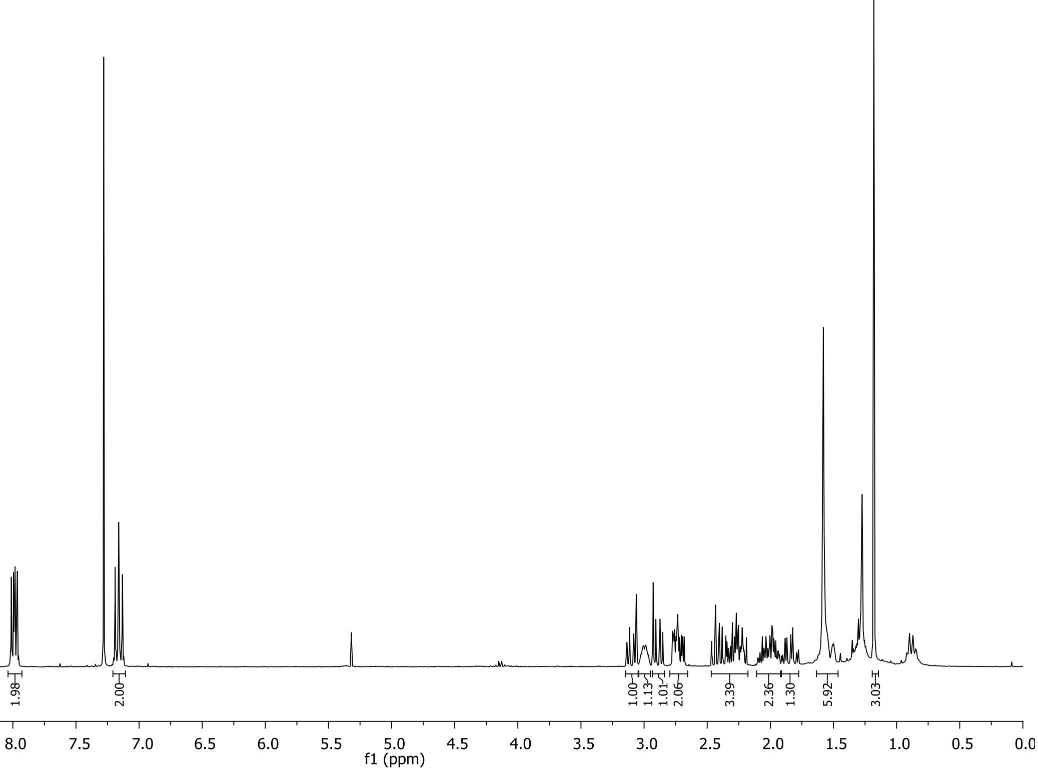
1.98
2.00
1.00
1.13
1.01
2.06
3.39
2.36
1.30
5.92
3.03
10.0 9.5 9.0 8.5 8.0 7.5 7.0 6.5 6.0 5.5 5.0 4.5 4.0 3.5 3.0 2.5 2.0 1.5 1.0 0.5 0.0
f1 (ppm)

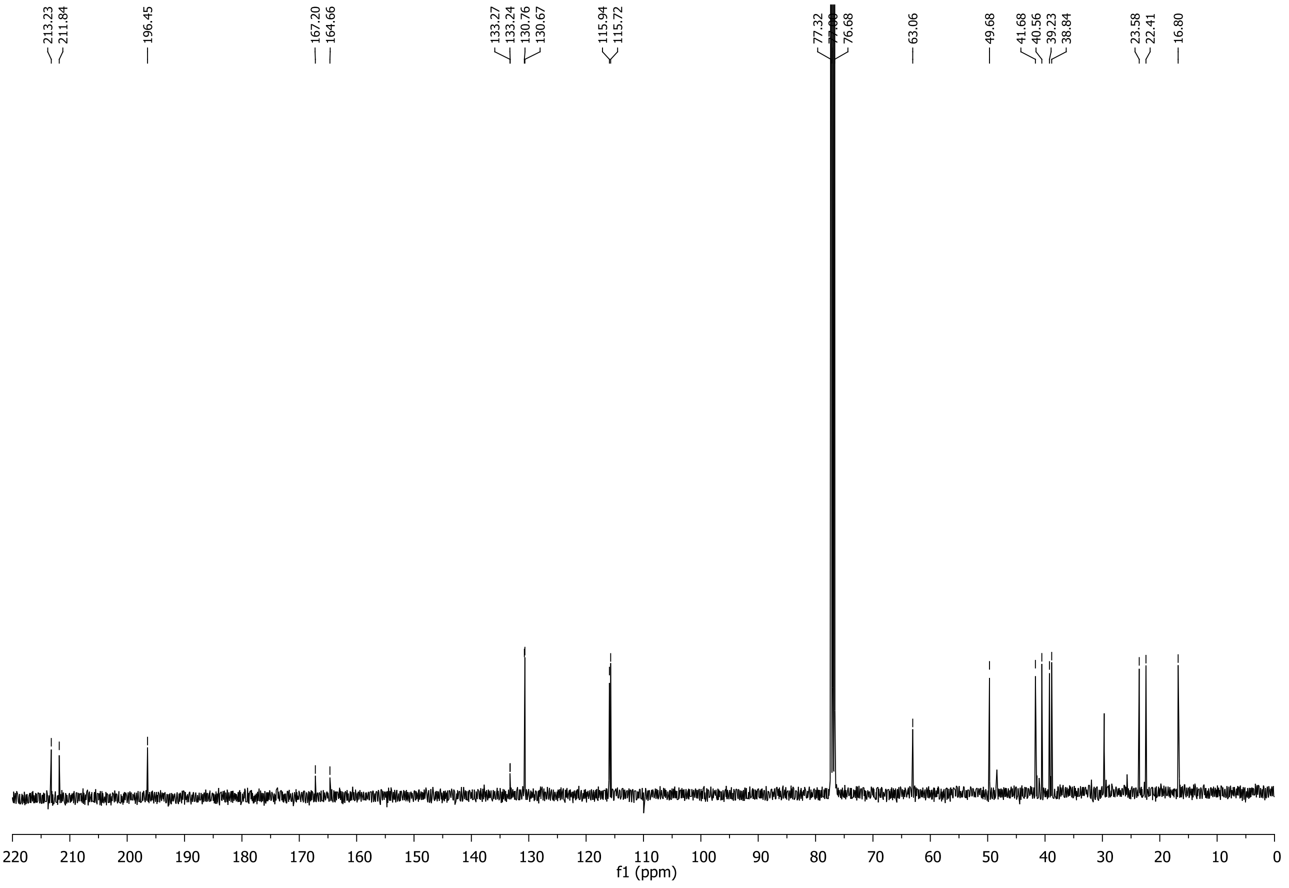
213.23
211.84
196.45
167.20
164.66
133.27
133.24
130.76
130.67
115.94
115.72
77.32
76.68
63.06
49.68
41.68
40.56
39.23
38.84
23.58
22.41
16.80
220 210 200 190 180 170 160 150 140 130 120 110 100 90 80 70 60 50 40 30 20 10 0
f1 (ppm)

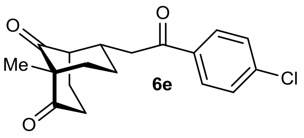
Me
Cl
O
O
O

6e

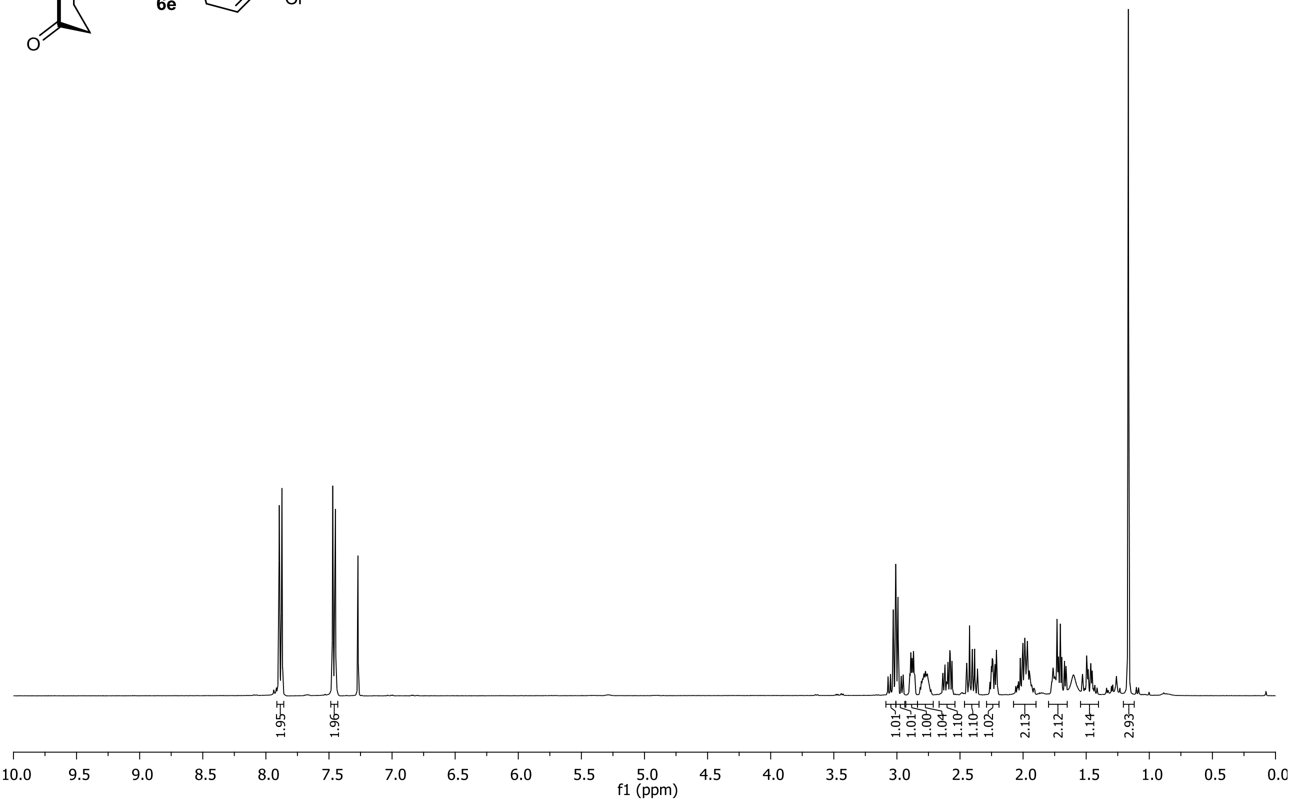
1.95
1.96
1.01
1.01
1.00
1.04
1.10
1.10
1.02
2.13
2.12
1.14
2.93
10.0 9.5 9.0 8.5 8.0 7.5 7.0 6.5 6.0 5.5 5.0 4.5 4.0 3.5 3.0 2.5 2.0 1.5 1.0 0.5 0.0
f1 (ppm)

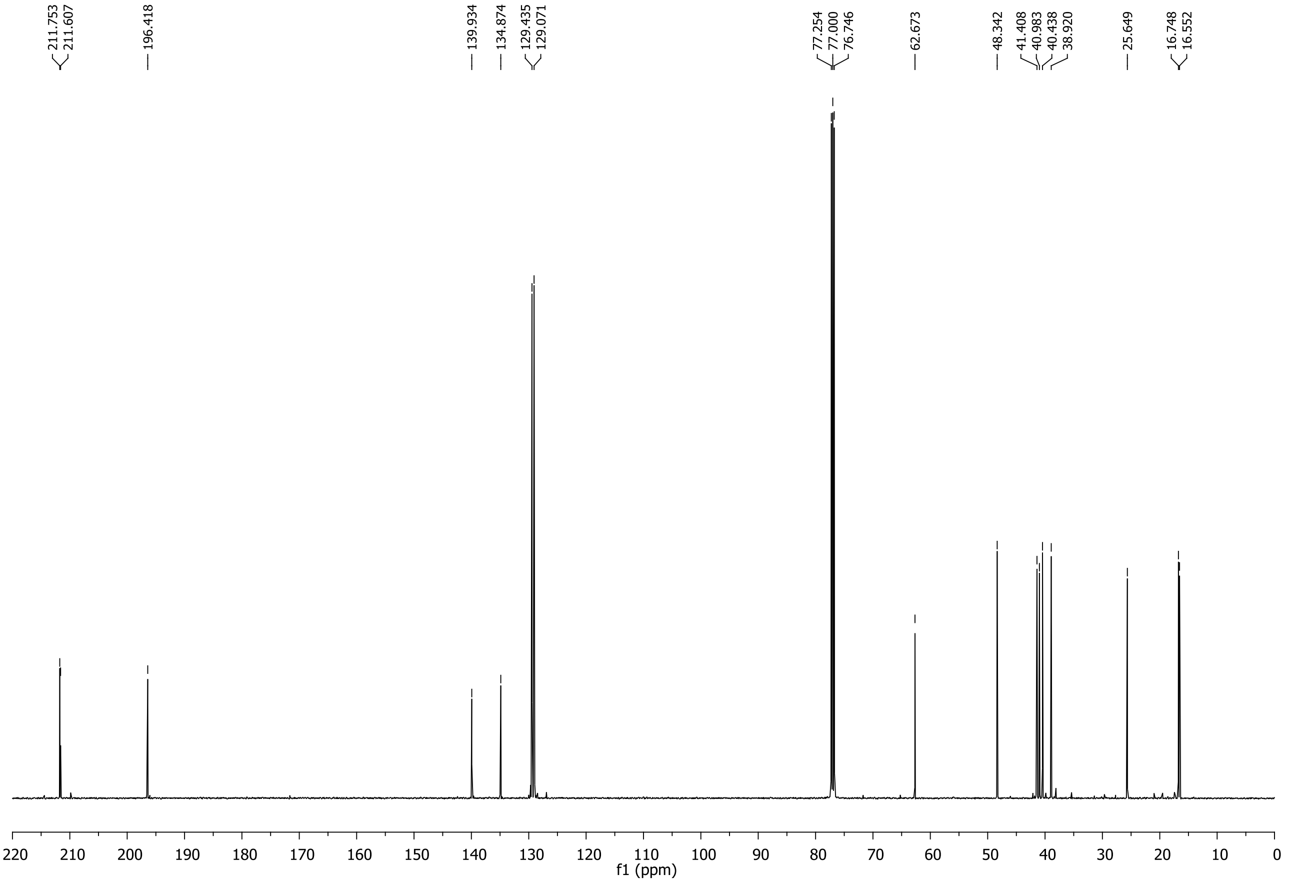
211.753
211.607
196.418
139.934
134.874
129.435
129.071
77.254
77.000
76.746
62.673
48.342
41.408
40.983
40.438
38.920
25.649
16.748
16.552
220 210 200 190 180 170 160 150 140 130 120 110 100 90 80 70 60 50 40 30 20 10 0
f1 (ppm)

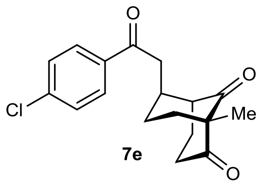
O
O
Me
Cl
O

7e

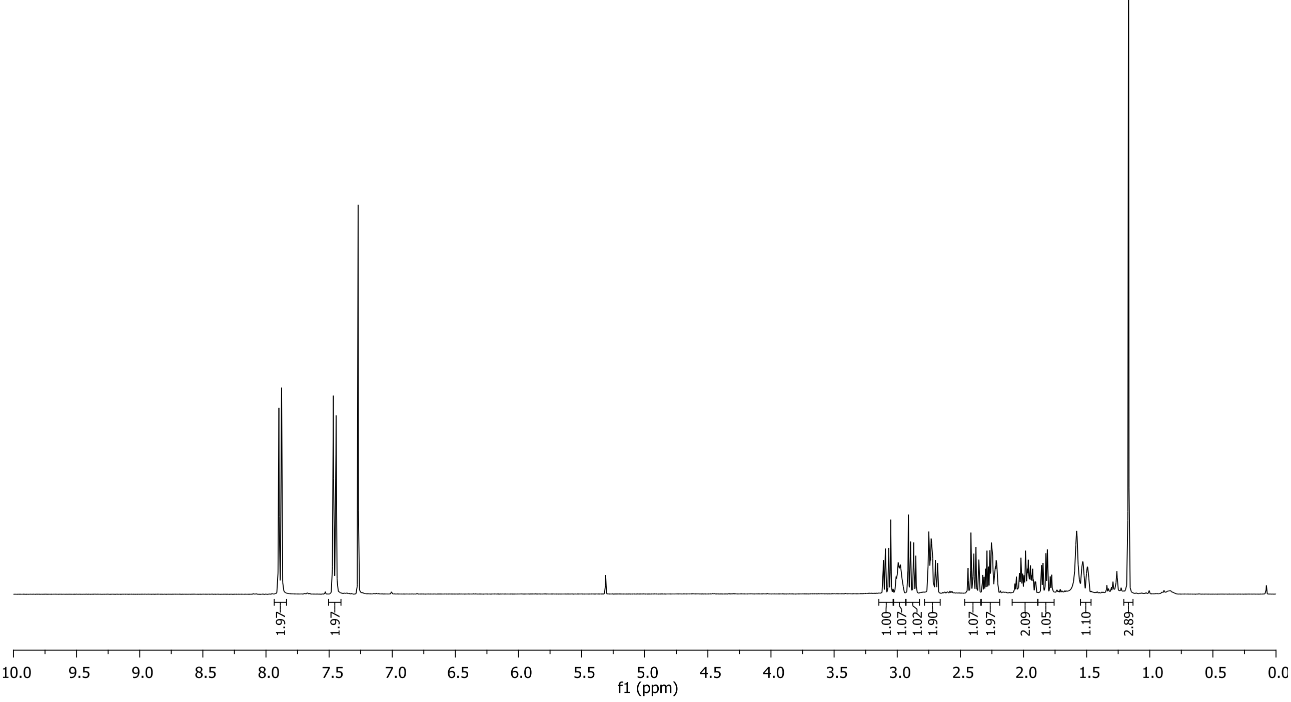
1.97
1.97
1.00
1.07
1.02
1.90
1.07
1.97
2.09
1.05
1.10
2.89
10.0 9.5 9.0 8.5 8.0 7.5 7.0 6.5 6.0 5.5 5.0 4.5 4.0 3.5 3.0 2.5 2.0 1.5 1.0 0.5 0.0
f1 (ppm)

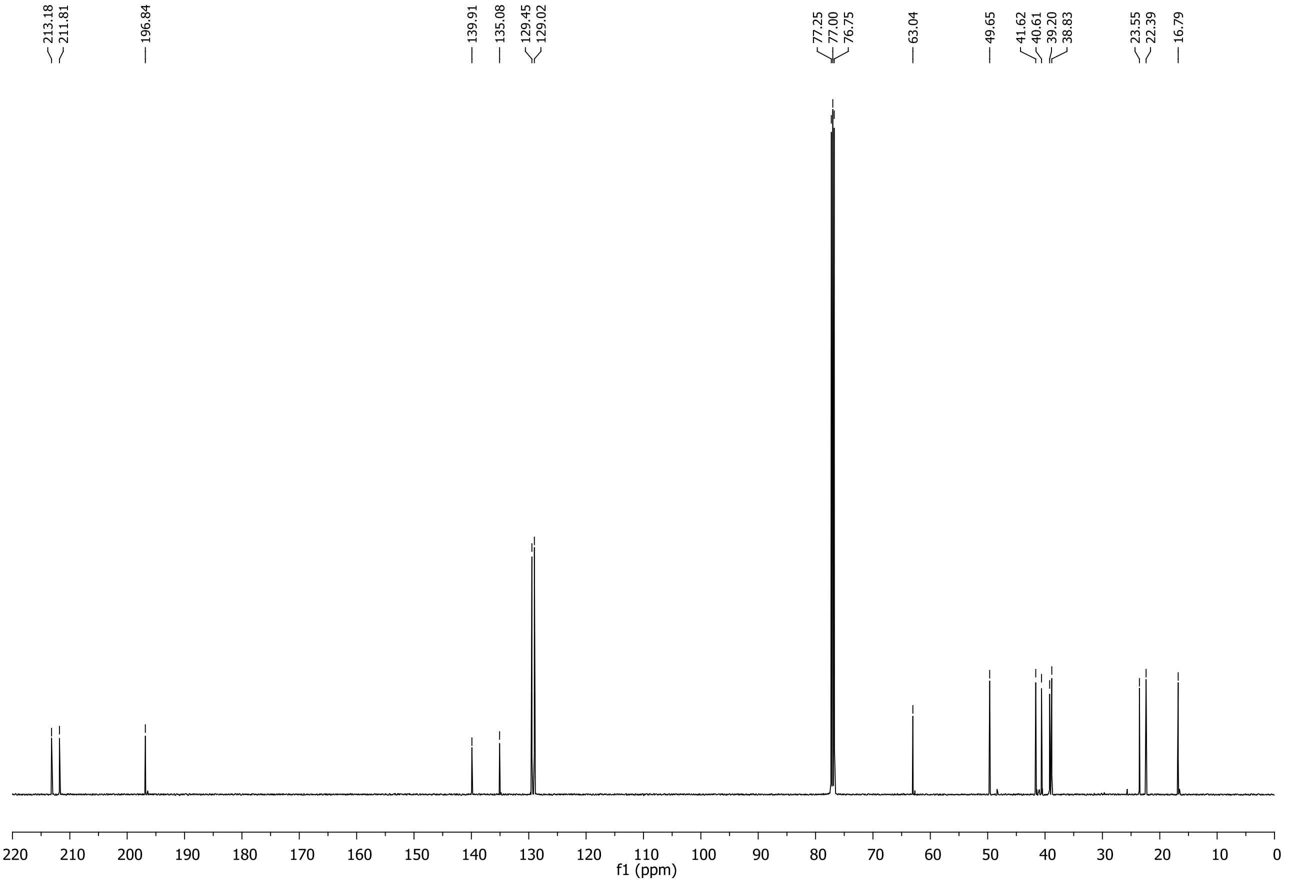
213.18
211.81
196.84
139.91
135.08
129.45
129.02
77.25
77.00
76.75
63.04
49.65
41.62
40.61
39.20
38.83
23.55
22.39
16.79
220 210 200 190 180 170 160 150 140 130 120 110 100 90 80 70 60 50 40 30 20 10 0
f1 (ppm)

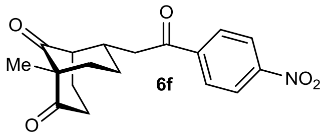
O
Me
O
O
6f
NO2

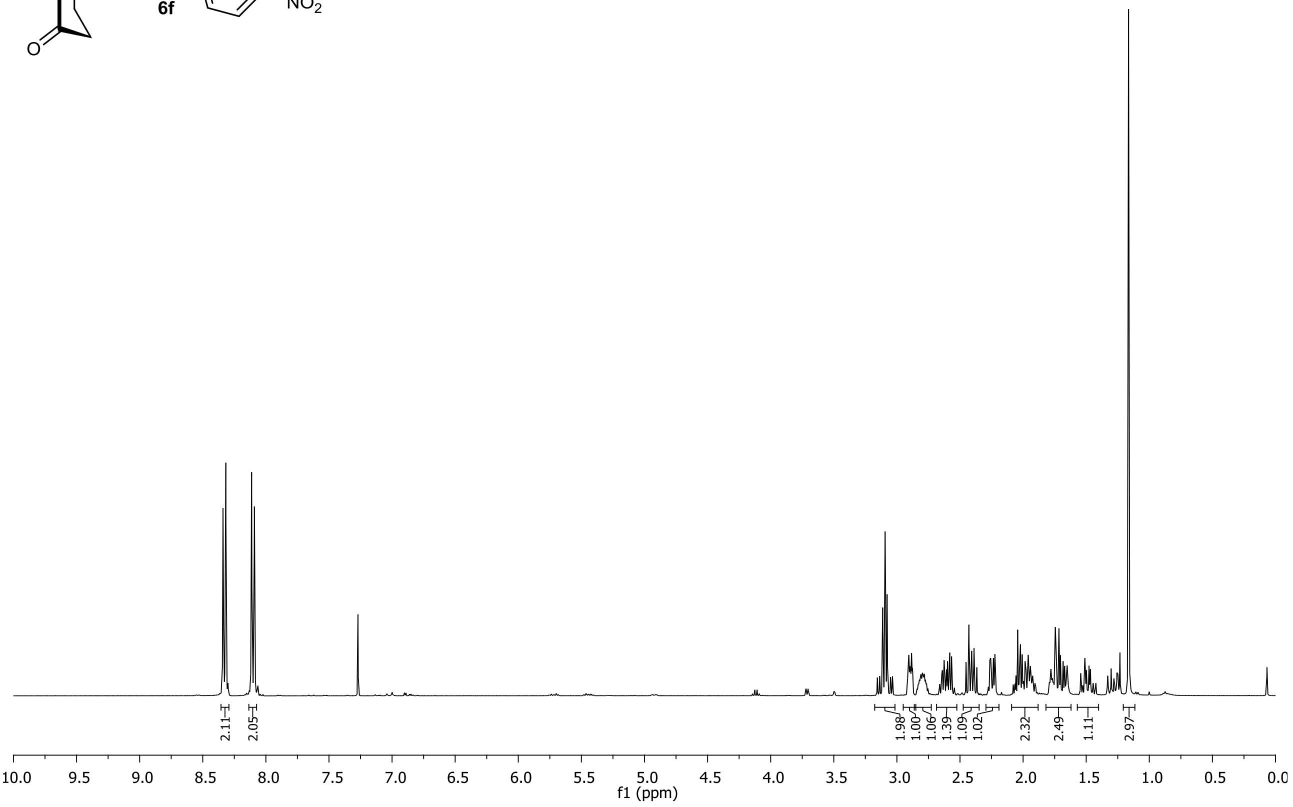
2.11
2.05
1.98
1.00
1.06
1.39
1.09
1.02
2.32
2.49
1.11
2.97
10.0 9.5 9.0 8.5 8.0 7.5 7.0 6.5 6.0 5.5 5.0 4.5 4.0 3.5 3.0 2.5 2.0 1.5 1.0 0.5 0.0
f1 (ppm)

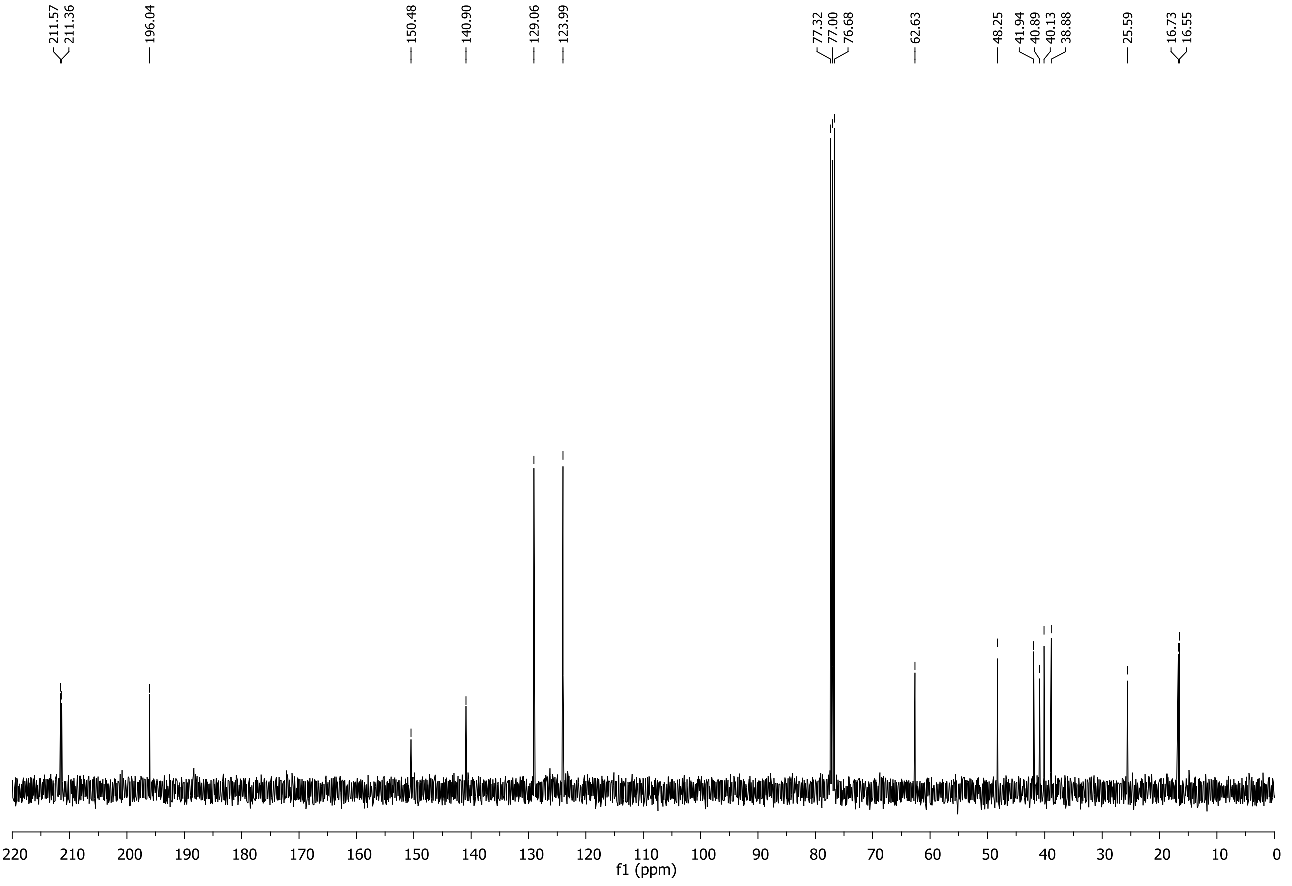
211.57
211.36
196.04
150.48
140.90
129.06
123.99
77.32
77.00
76.68
62.63
48.25
41.94
40.89
40.13
38.88
25.59
16.73
16.55
220 210 200 190 180 170 160 150 140 130 120 110 100 90 80 70 60 50 40 30 20 10 0
f1 (ppm)

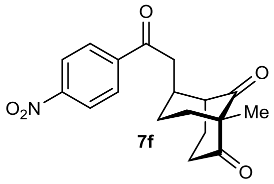
O
O
O
O₂N
Me

7f

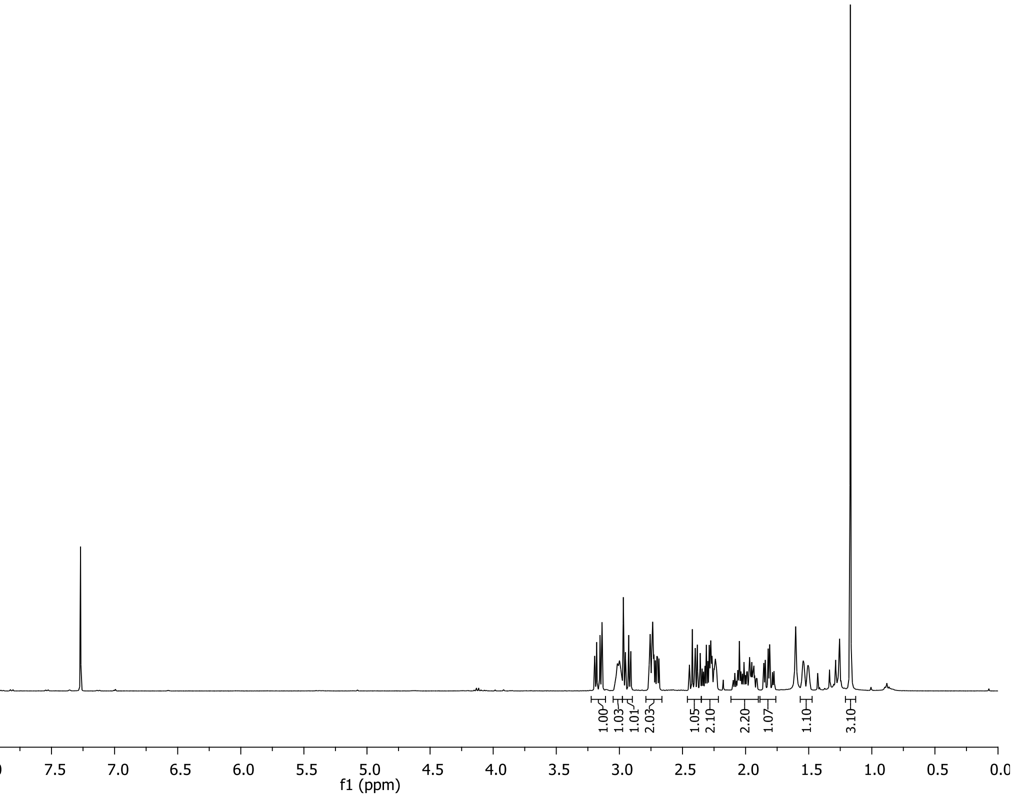
2.01
2.03
1.00
1.03
1.01
2.03
1.05
2.10
2.20
1.07
1.10
3.10
f1 (ppm)

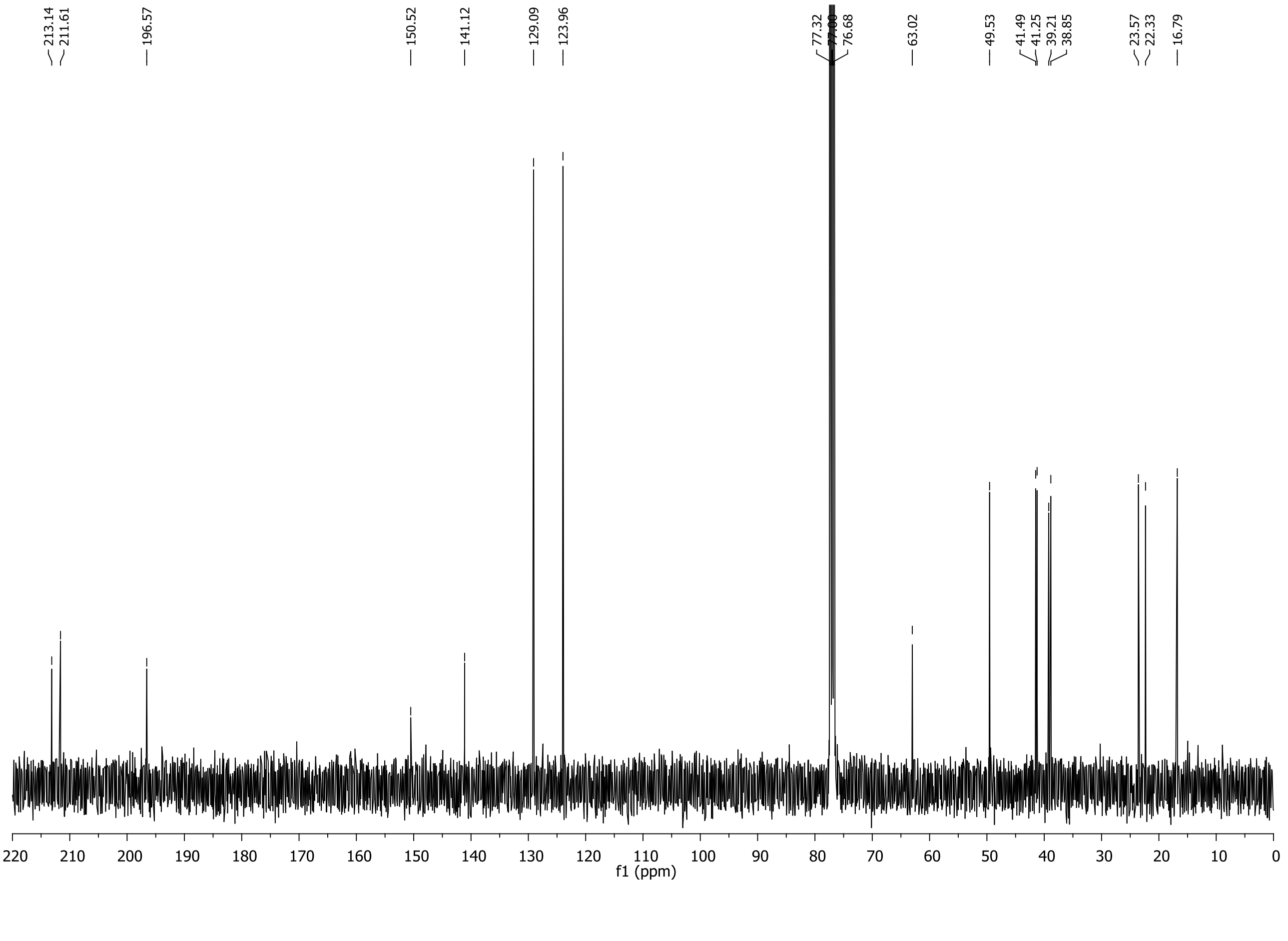
213.14
211.61
196.57
150.52
141.12
129.09
123.96
77.32
[illegible]
76.68
63.02
49.53
41.49
41.25
39.21
38.85
23.57
22.33
16.79
f1 (ppm)

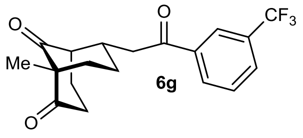
CF3
Me
O
O
O

6g

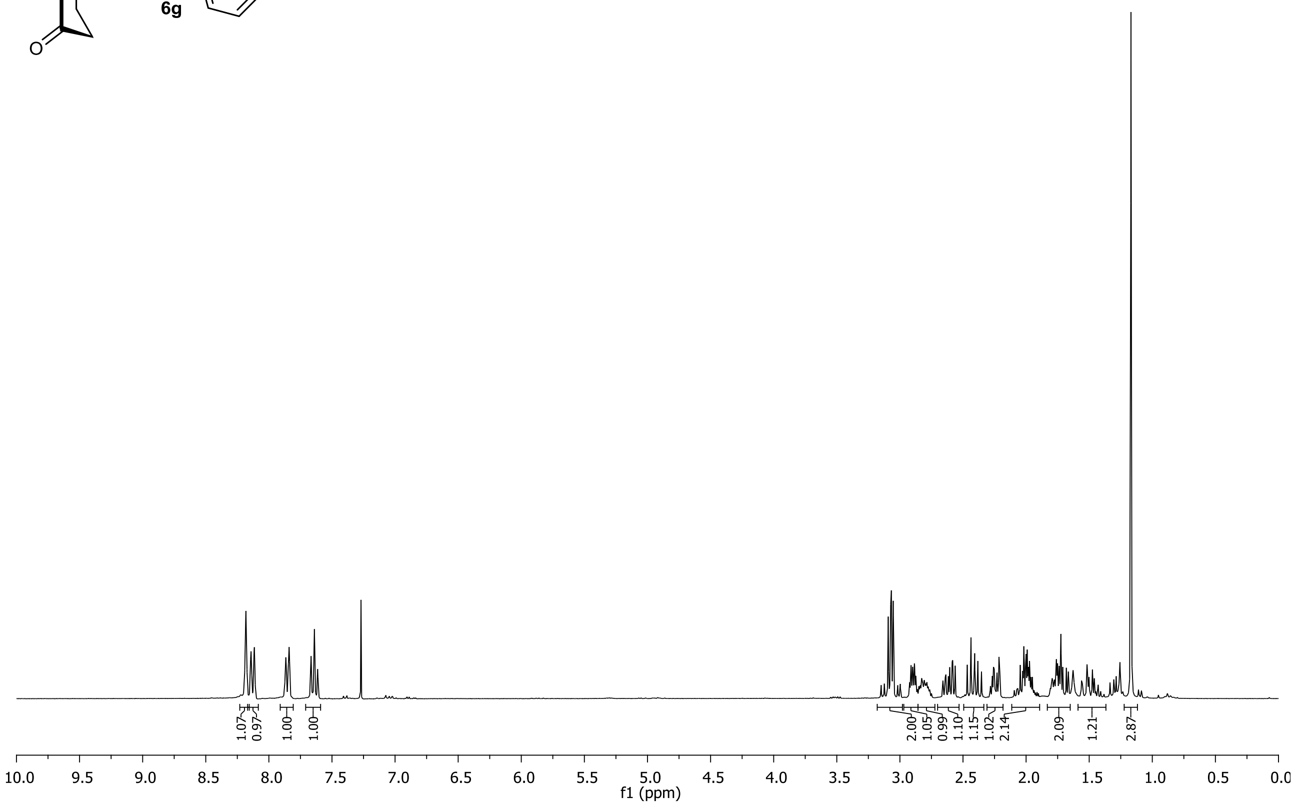
1.07
0.97
1.00
1.00
2.00
1.05
0.99
1.10
1.15
1.02
2.14
2.09
1.21
2.87
f1 (ppm)

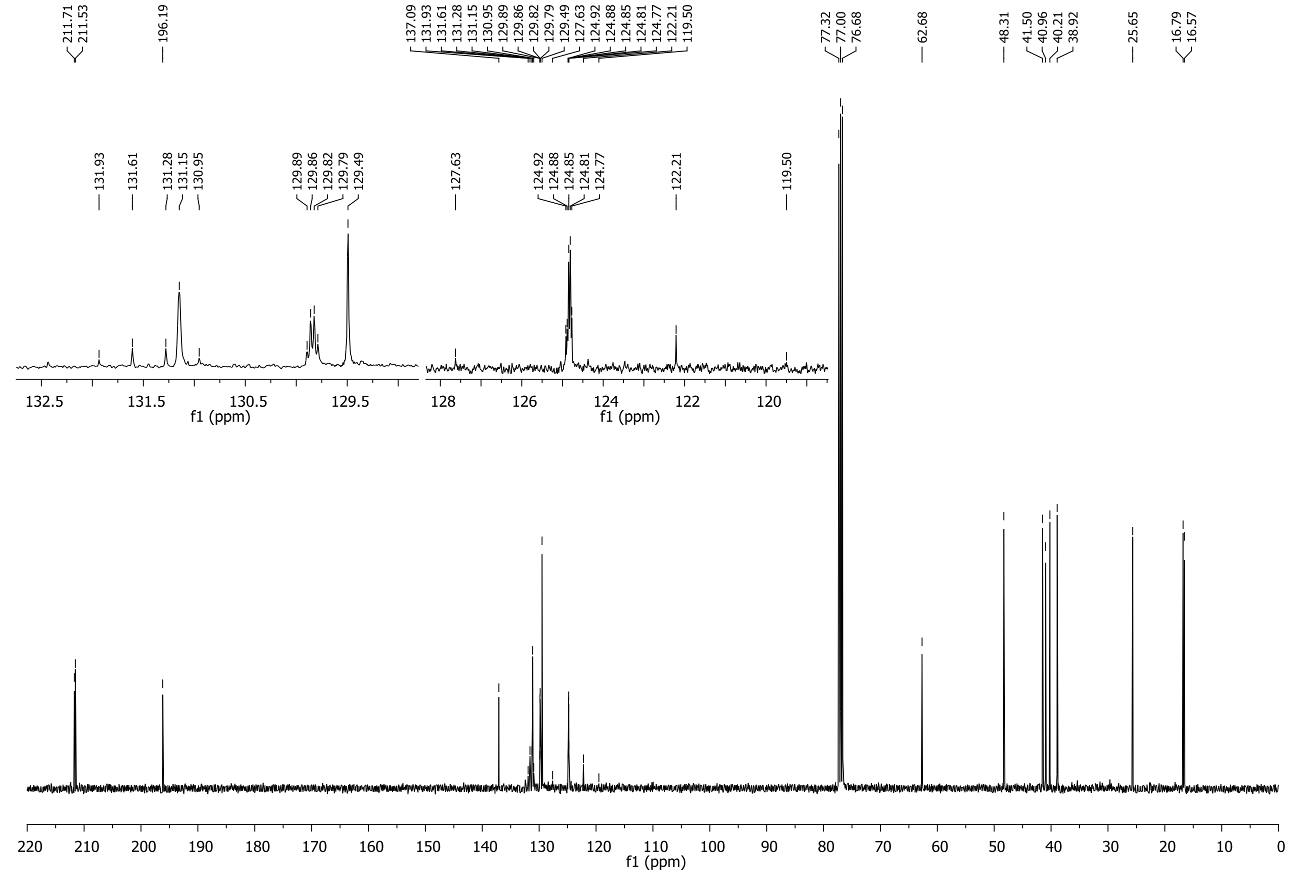
211.71
211.53
196.19
137.09
131.93
131.61
131.28
131.15
130.95
129.89
129.86
129.82
129.79
129.49
127.63
124.92
124.88
124.85
124.81
124.77
122.21
119.50
77.32
77.00
76.68
62.68
48.31
41.50
40.96
40.21
38.92
25.65
16.79
16.57
f1 (ppm)

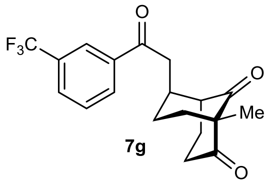
F3C
O
O
Me
O

7g

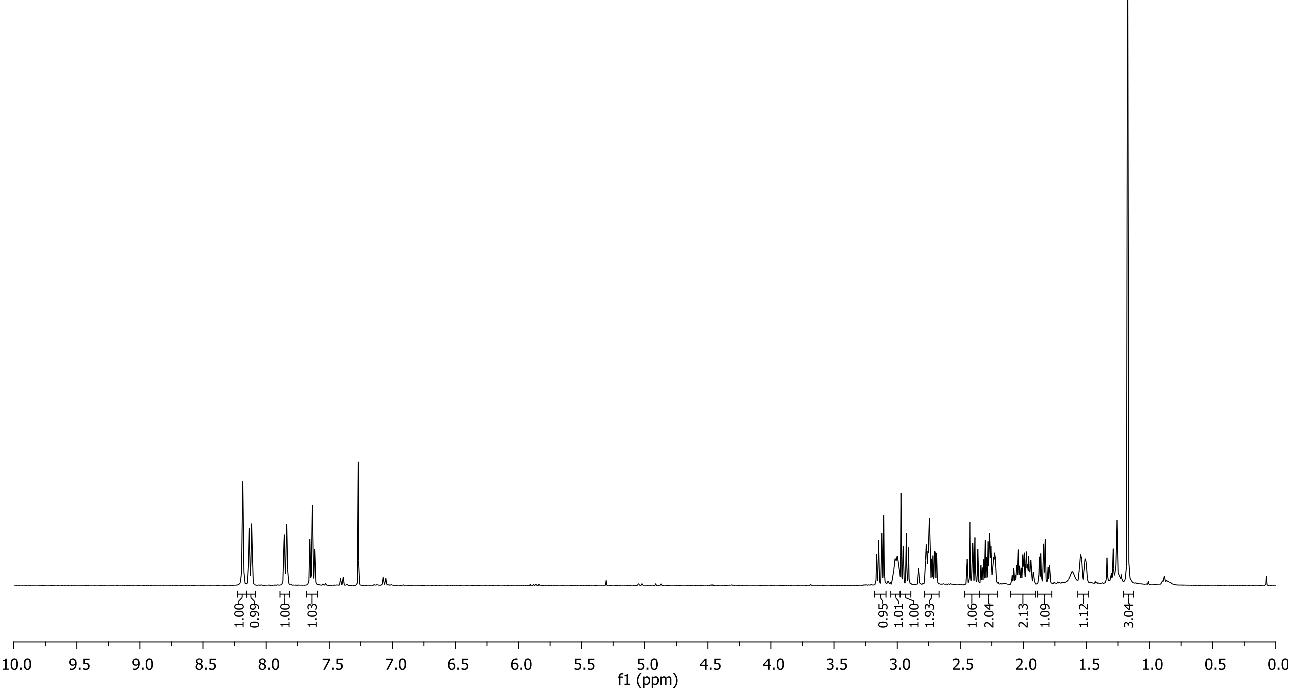
1.00
0.99
1.00
1.03
0.95
1.01
1.00
1.93
1.06
2.04
2.13
1.09
1.12
3.04
10.0 9.5 9.0 8.5 8.0 7.5 7.0 6.5 6.0 5.5 5.0 4.5 4.0 3.5 3.0 2.5 2.0 1.5 1.0 0.5 0.0
f1 (ppm)

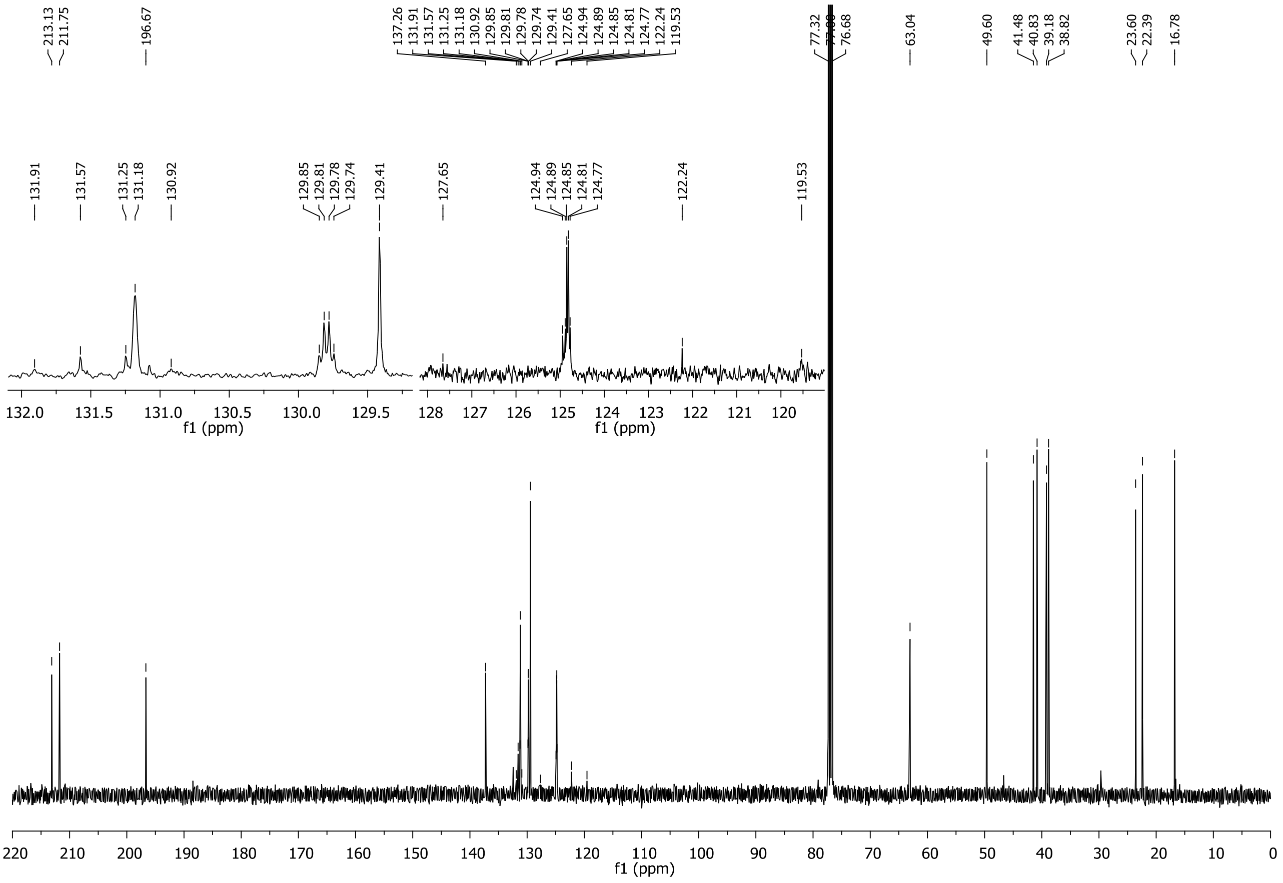
213.13
211.75
196.67
137.26
131.91
131.57
131.25
131.18
130.92
129.85
129.81
129.78
129.74
129.41
127.65
124.94
124.89
124.85
124.81
124.77
122.24
119.53
77.32
76.68
63.04
49.60
41.48
40.83
39.18
38.82
23.60
22.39
16.78
132.0 131.5 131.0 130.5 130.0 129.5
f1 (ppm)
128 127 126 125 124 123 122 121 120
f1 (ppm)
220 210 200 190 180 170 160 150 140 130 120 110 100 90 80 70 60 50 40 30 20 10 0
f1 (ppm)

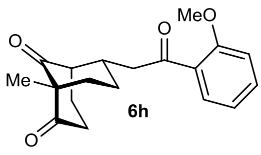
MeO
O
O
Me
O
6h

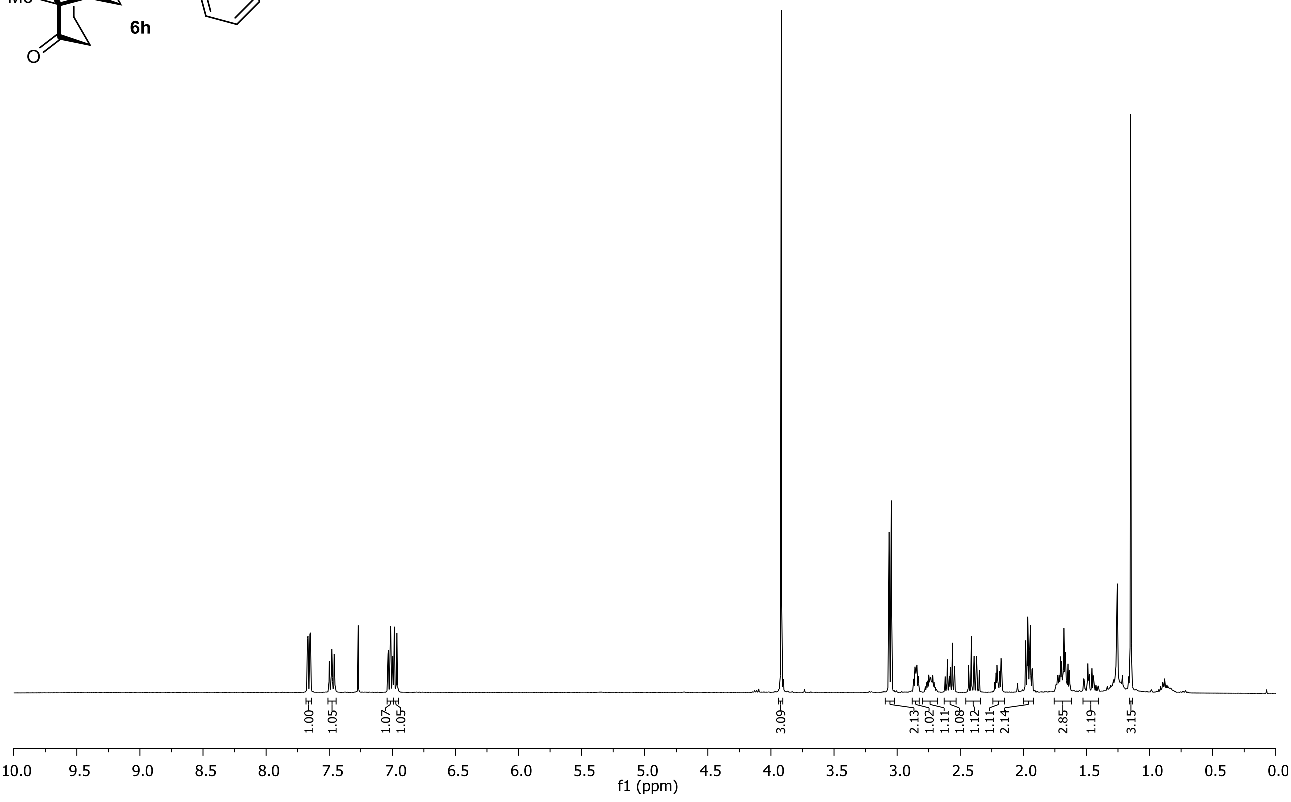
1.00
1.05
1.07
1.05
3.09
2.13
1.02
1.11
1.08
1.12
1.11
2.14
2.85
1.19
3.15
10.0 9.5 9.0 8.5 8.0 7.5 7.0 6.5 6.0 5.5 5.0 4.5 4.0 3.5 3.0 2.5 2.0 1.5 1.0 0.5 0.0
f1 (ppm)

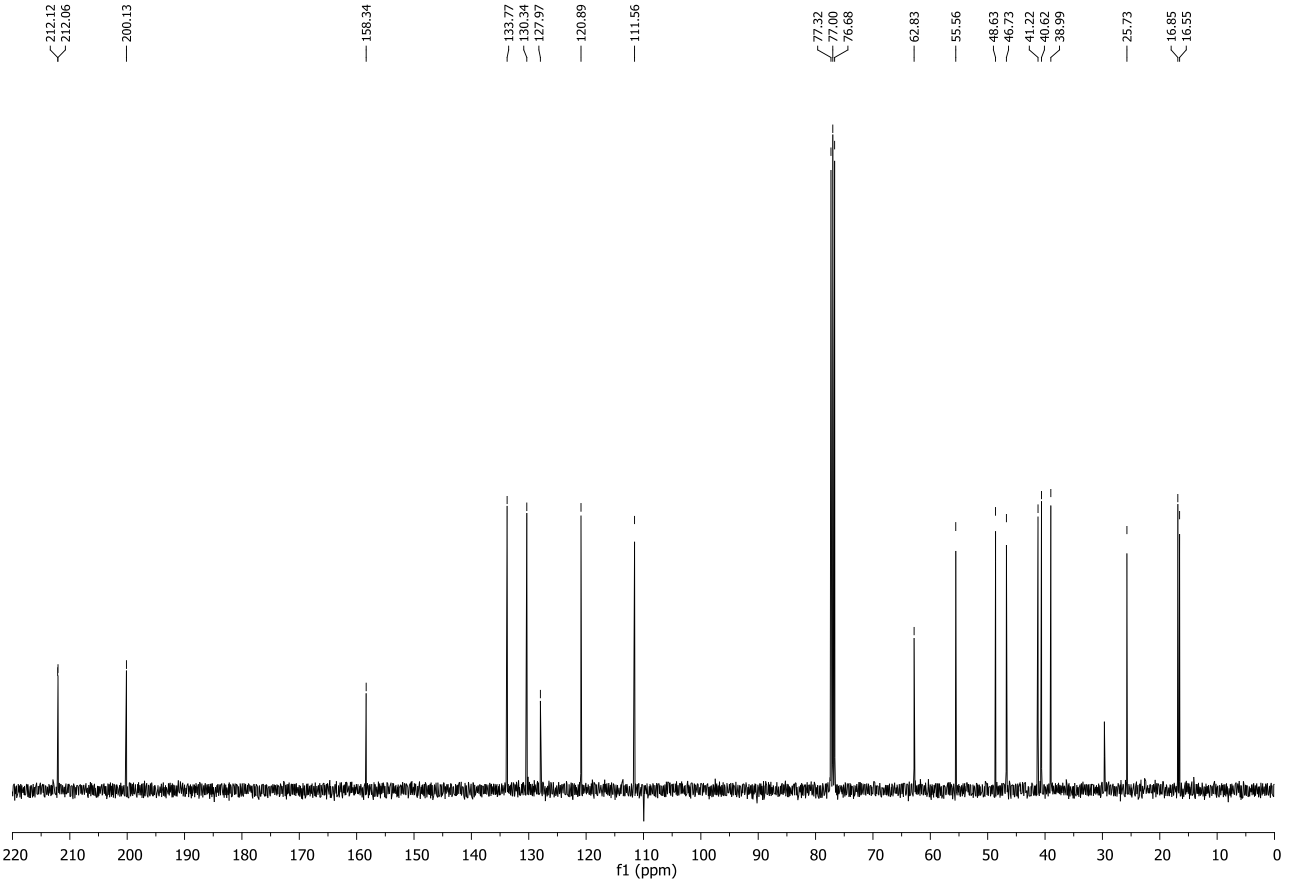
212.12
212.06
200.13
158.34
133.77
130.34
127.97
120.89
111.56
77.32
77.00
76.68
62.83
55.56
48.63
46.73
41.22
40.62
38.99
25.73
16.85
16.55
220 210 200 190 180 170 160 150 140 130 120 110 100 90 80 70 60 50 40 30 20 10 0
f1 (ppm)

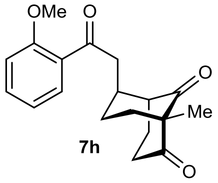
OMe
O
O
Me
O

7h

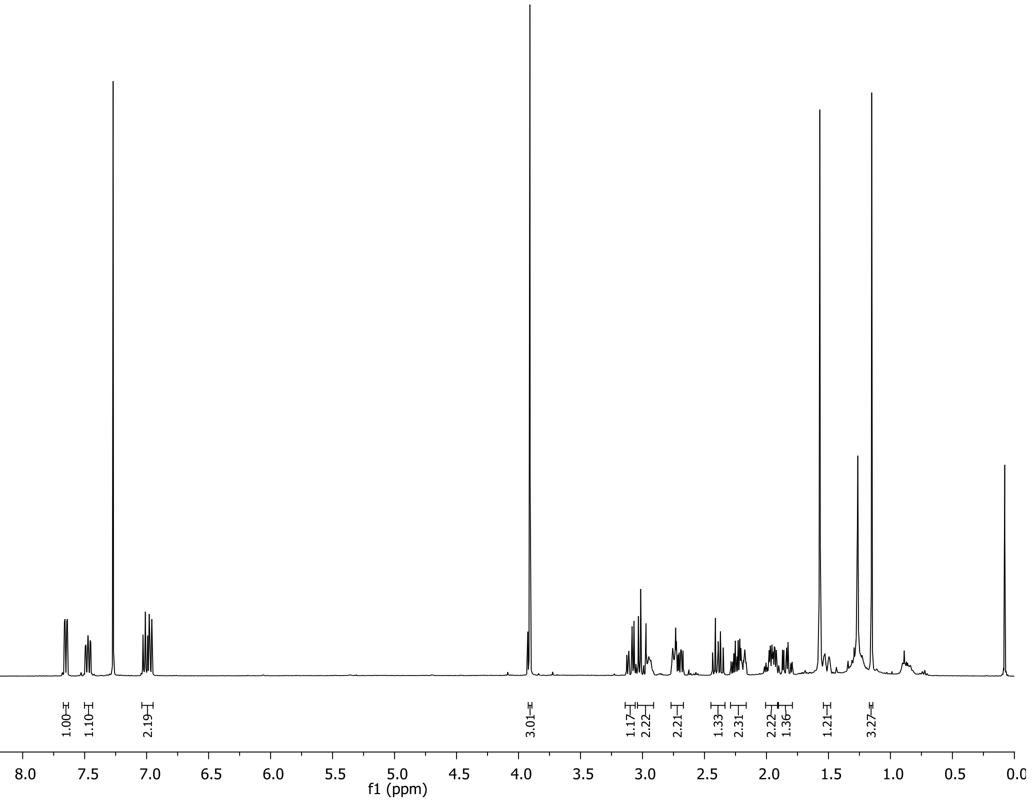
1.00
1.10
2.19
3.01
1.17
2.22
2.21
1.33
2.31
2.22
1.36
1.21
3.27
10.0 9.5 9.0 8.5 8.0 7.5 7.0 6.5 6.0 5.5 5.0 4.5 4.0 3.5 3.0 2.5 2.0 1.5 1.0 0.5 0.0
f1 (ppm)

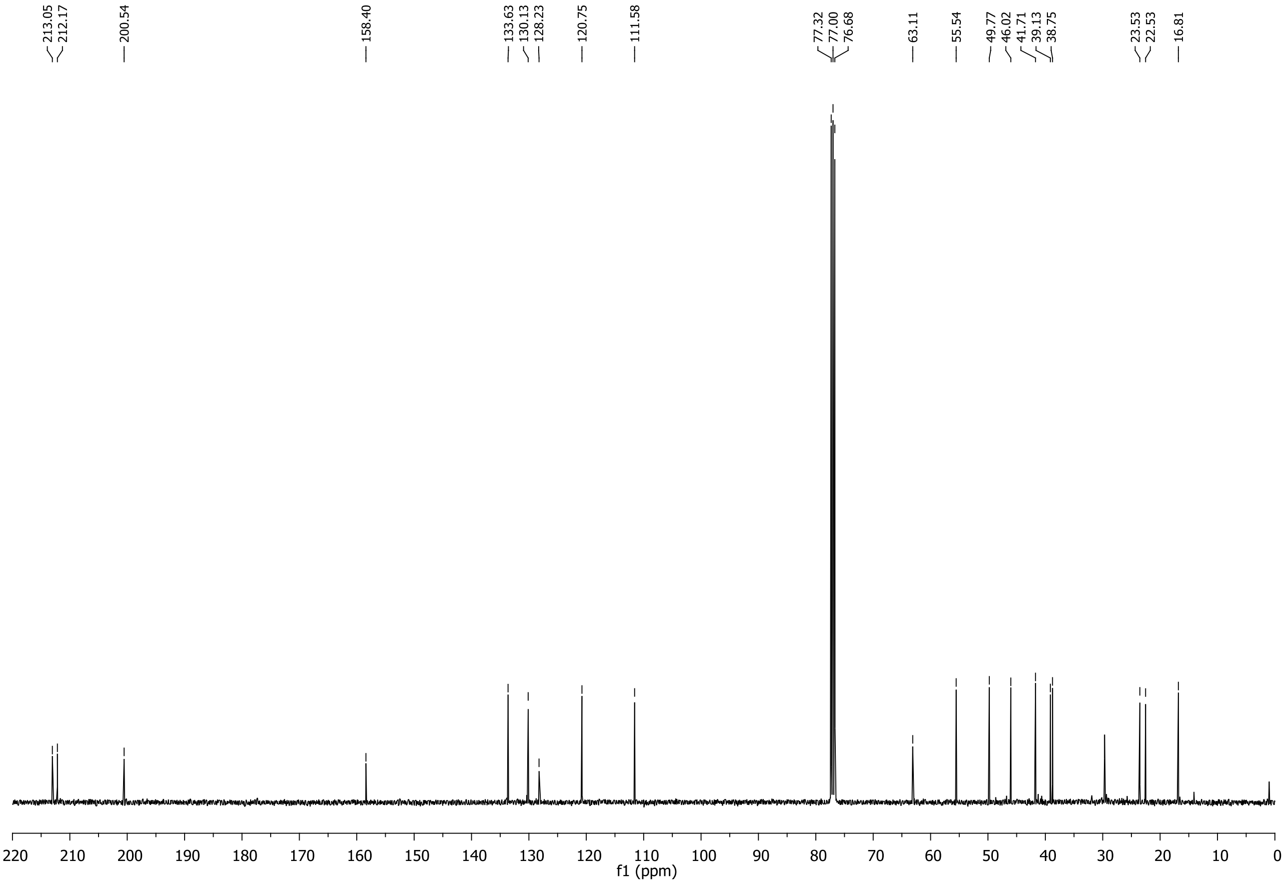
213.05
212.17
200.54
158.40
133.63
130.13
128.23
120.75
111.58
77.32
77.00
76.68
63.11
55.54
49.77
46.02
41.71
39.13
38.75
23.53
22.53
16.81
220 210 200 190 180 170 160 150 140 130 120 110 100 90 80 70 60 50 40 30 20 10 0
f1 (ppm)

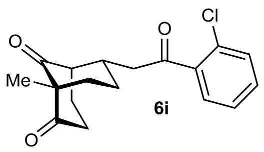
O
O
Cl
Me
6i
O

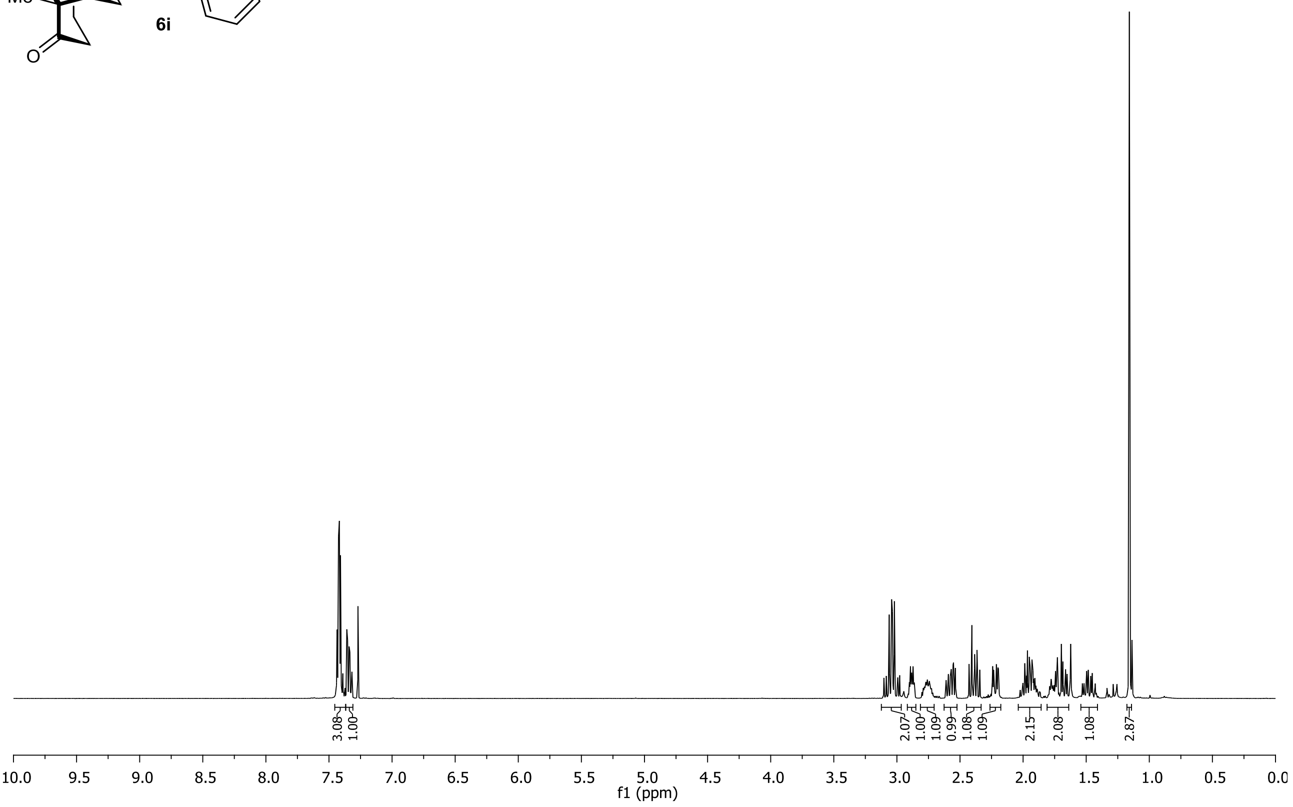
3.08
1.00
2.07
1.00
1.09
0.99
1.08
1.09
2.15
2.08
1.08
2.87
10.0 9.5 9.0 8.5 8.0 7.5 7.0 6.5 6.0 5.5 5.0 4.5 4.0 3.5 3.0 2.5 2.0 1.5 1.0 0.5 0.0
f1 (ppm)

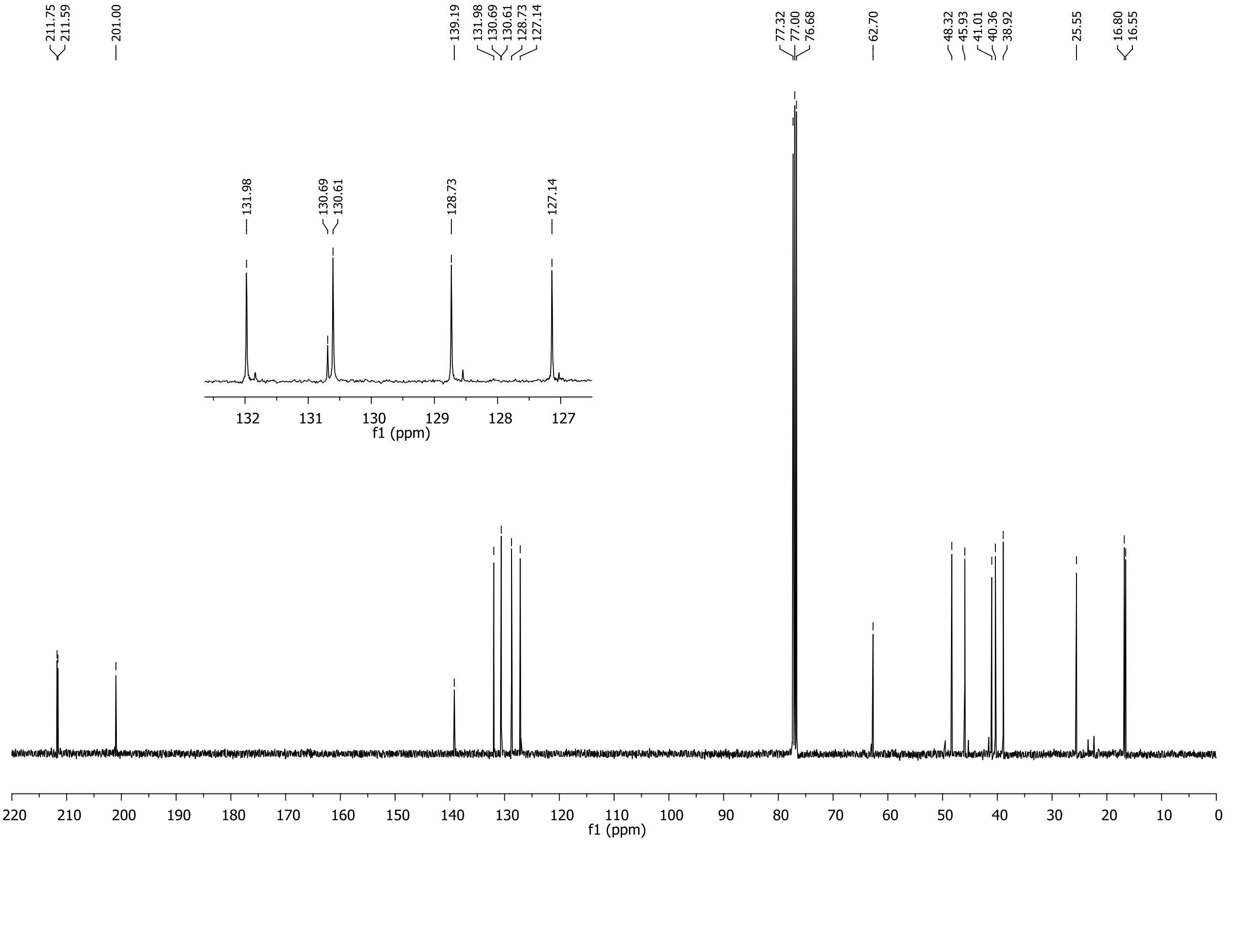
211.75
211.59
201.00
139.19
131.98
130.69
130.61
128.73
127.14
77.32
77.00
76.68
62.70
48.32
45.93
41.01
40.36
38.92
25.55
16.80
16.55
132 131 130 129 128 127
f1 (ppm)
220 210 200 190 180 170 160 150 140 130 120 110 100 90 80 70 60 50 40 30 20 10 0
f1 (ppm)

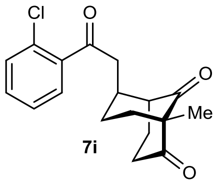
Cl
O
O
Me
O

7i

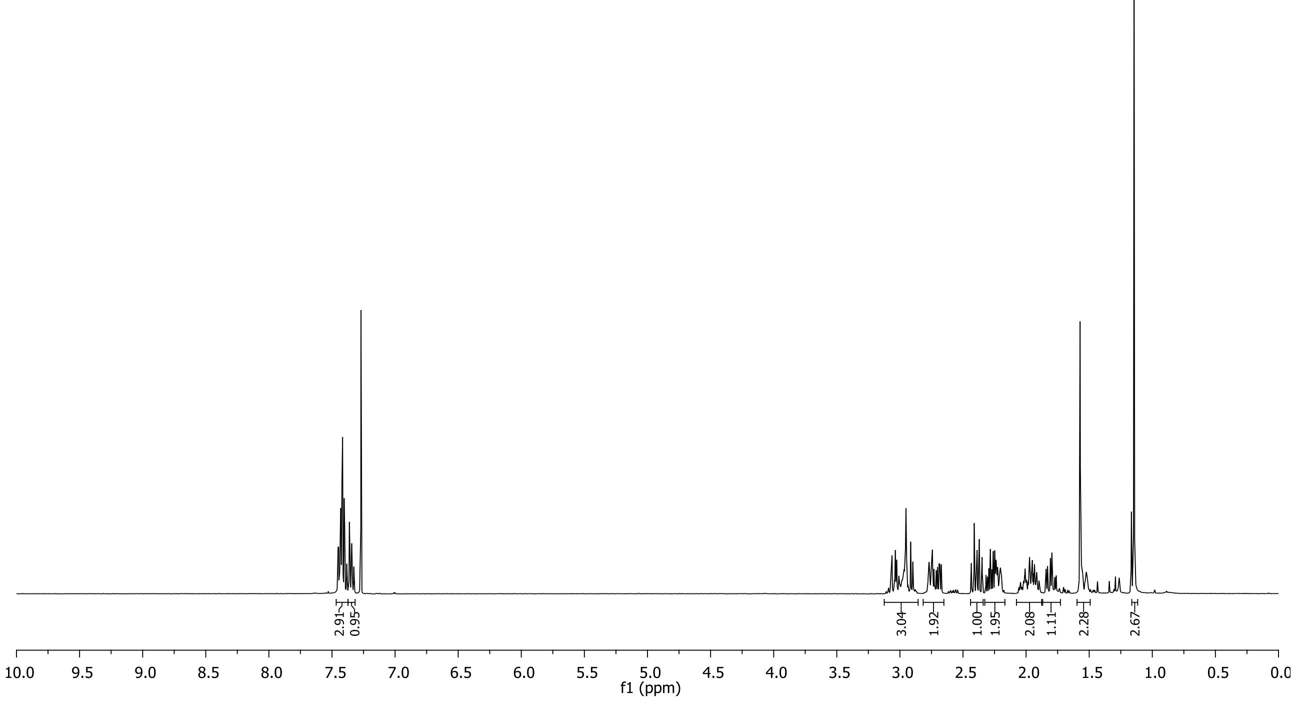
2.91
0.95
3.04
1.92
1.00
1.95
2.08
1.11
2.28
2.67
10.0 9.5 9.0 8.5 8.0 7.5 7.0 6.5 6.0 5.5 5.0 4.5 4.0 3.5 3.0 2.5 2.0 1.5 1.0 0.5 0.0
f1 (ppm)

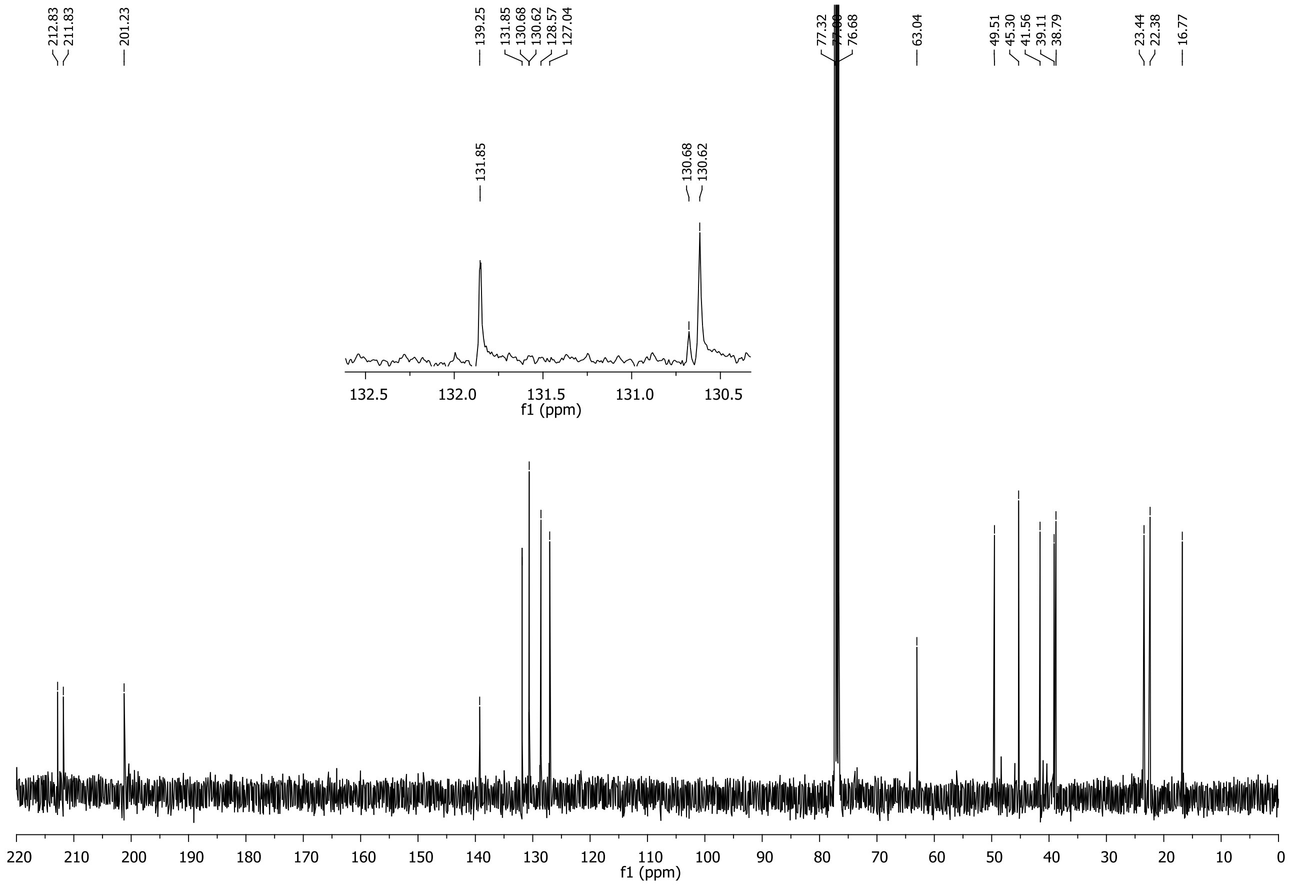
212.83
211.83
201.23
139.25
131.85
130.68
130.62
128.57
127.04
77.32
76.68
63.04
49.51
45.30
41.56
39.11
38.79
23.44
22.38
16.77
131.85
130.68
130.62
132.5 132.0 131.5 131.0 130.5
f1 (ppm)
220 210 200 190 180 170 160 150 140 130 120 110 100 90 80 70 60 50 40 30 20 10 0
f1 (ppm)

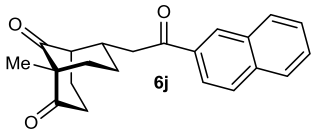
O
O
Me
O

6j

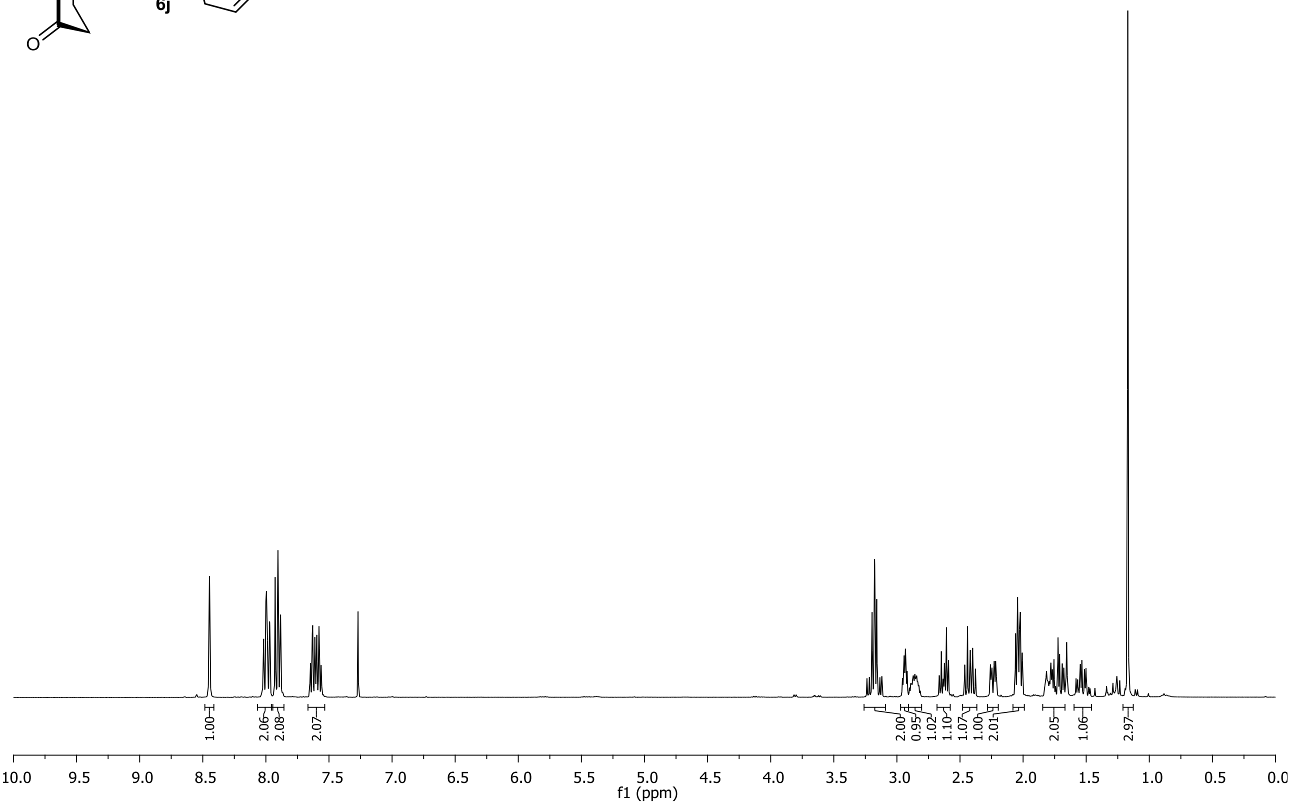
1.00
2.06
2.08
2.07
2.00
0.95
1.02
1.10
1.07
1.00
2.01
2.05
1.06
2.97
10.0 9.5 9.0 8.5 8.0 7.5 7.0 6.5 6.0 5.5 5.0 4.5 4.0 3.5 3.0 2.5 2.0 1.5 1.0 0.5 0.0
f1 (ppm)

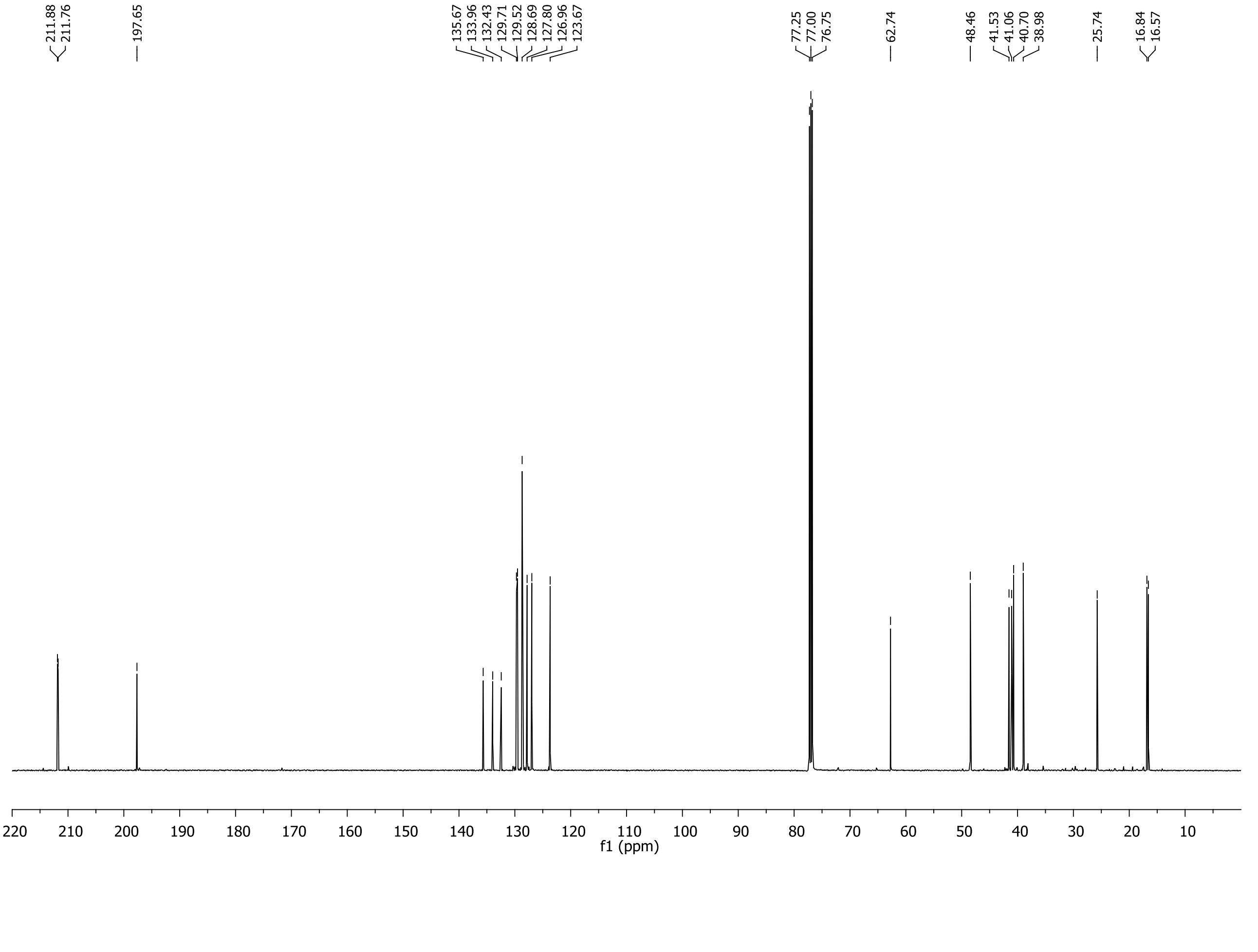
211.88
211.76
197.65
135.67
133.96
132.43
129.71
129.52
128.69
127.80
126.96
123.67
77.25
77.00
76.75
62.74
48.46
41.53
41.06
40.70
38.98
25.74
16.84
16.57
220 210 200 190 180 170 160 150 140 130 120 110 100 90 80 70 60 50 40 30 20 10
f1 (ppm)

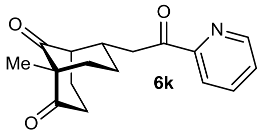
O
O
N
Me
O
6k

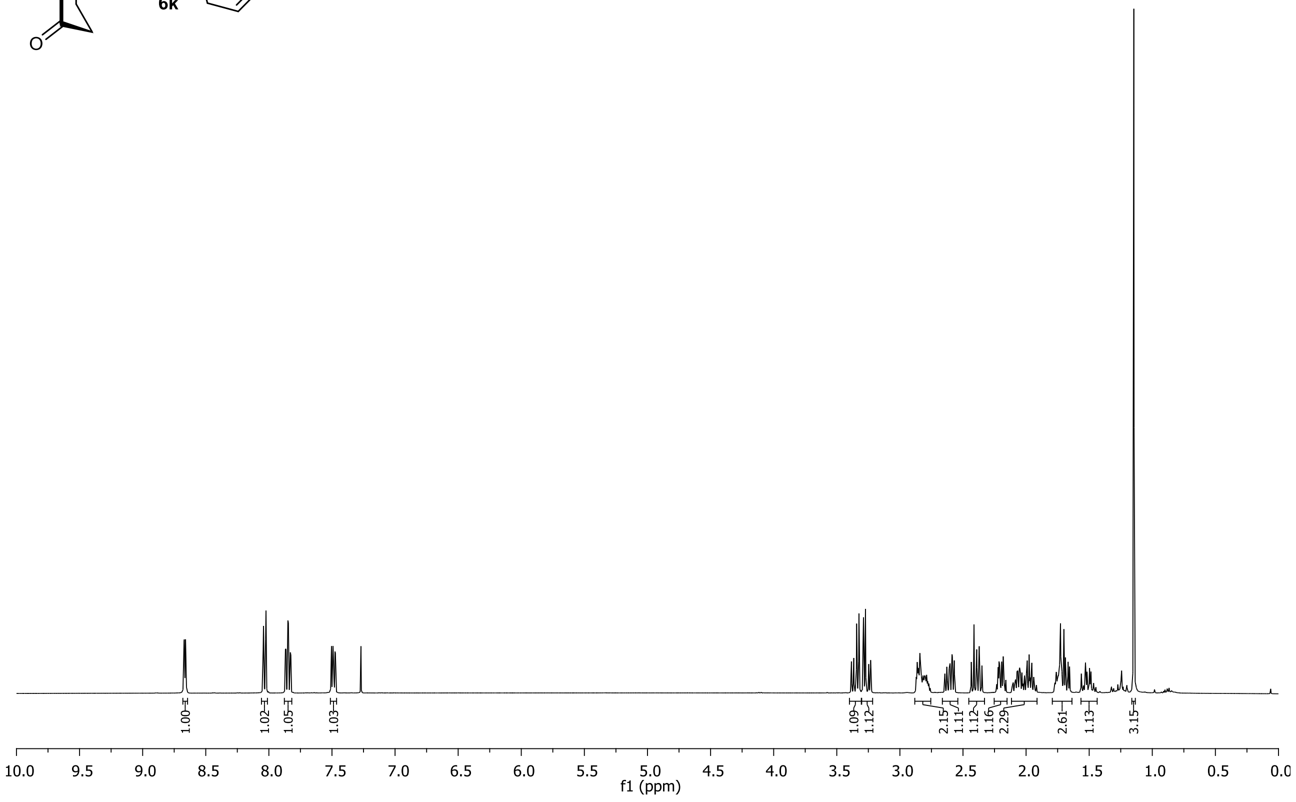
1.00
1.02
1.05
1.03
1.09
1.12
2.15
1.11
1.12
1.16
2.29
2.61
1.13
3.15
10.0 9.5 9.0 8.5 8.0 7.5 7.0 6.5 6.0 5.5 5.0 4.5 4.0 3.5 3.0 2.5 2.0 1.5 1.0 0.5 0.0
f1 (ppm)

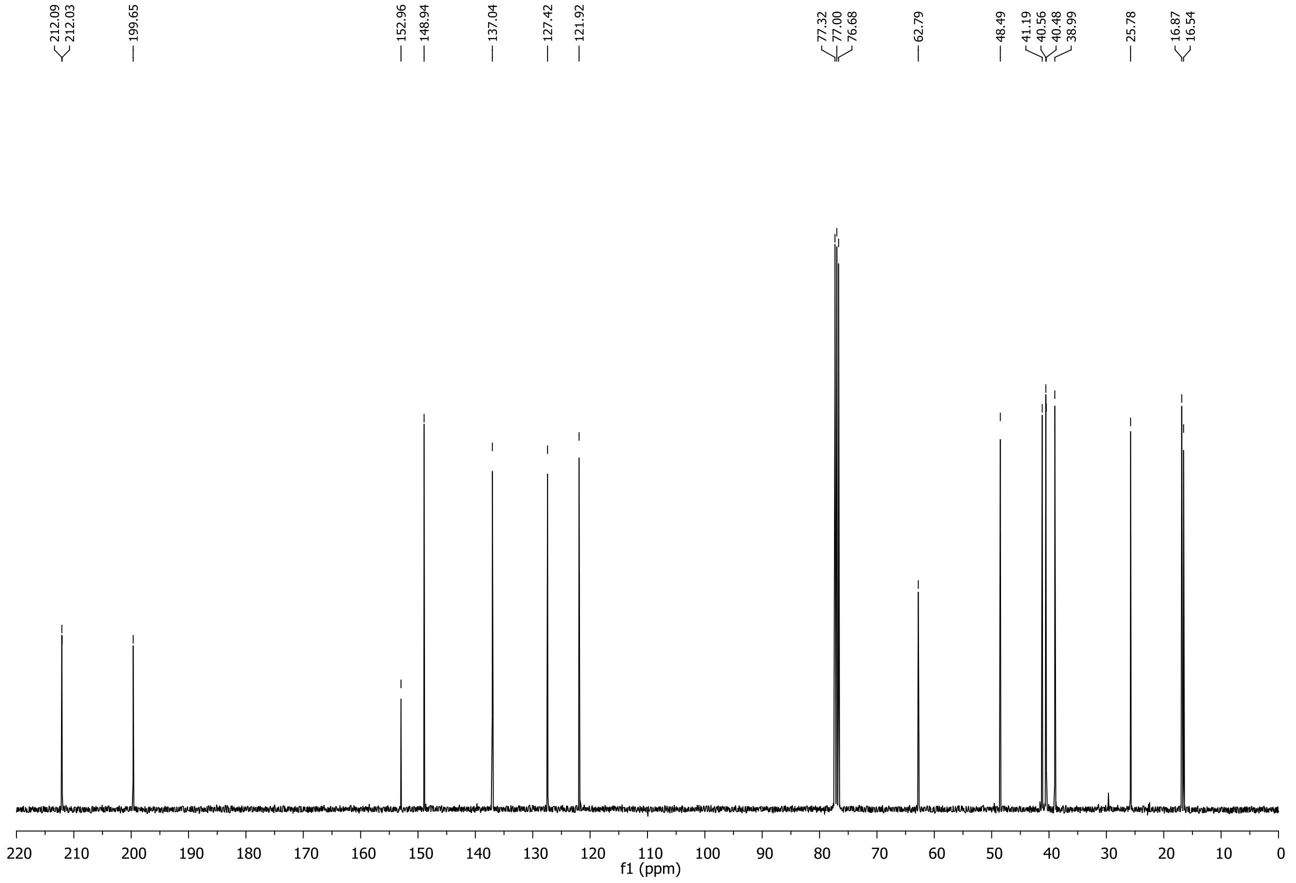
212.09
212.03
199.65
152.96
148.94
137.04
127.42
121.92
77.32
77.00
76.68
62.79
48.49
41.19
40.56
40.48
38.99
25.78
16.87
16.54
220 210 200 190 180 170 160 150 140 130 120 110 100 90 80 70 60 50 40 30 20 10 0
f1 (ppm)

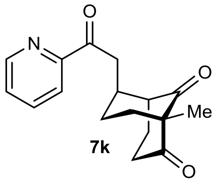
Me
O
N

7k

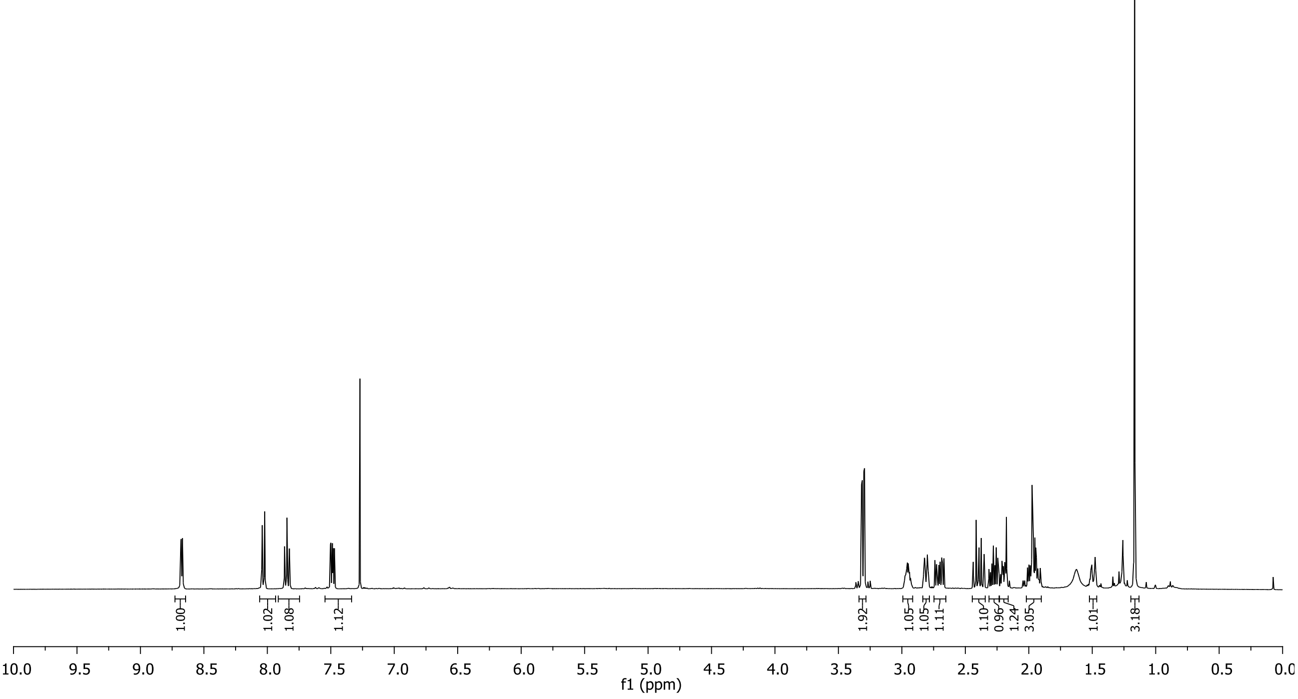
1.00
1.02
1.08
1.12
1.92
1.05
1.05
1.11
1.10
0.96
1.24
3.05
1.01
3.18
f1 (ppm)

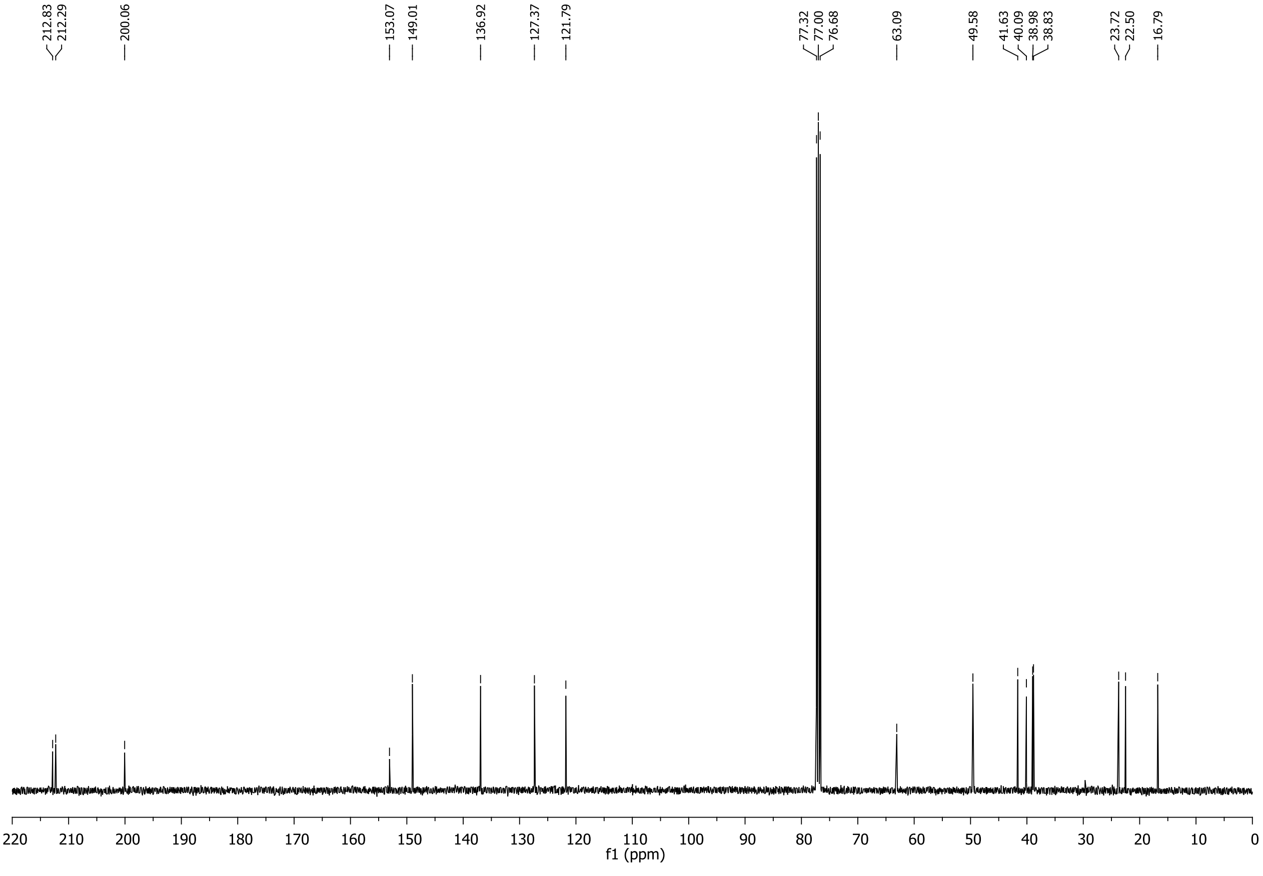
212.83
212.29
200.06
153.07
149.01
136.92
127.37
121.79
77.32
77.00
76.68
63.09
49.58
41.63
40.09
38.98
38.83
23.72
22.50
16.79
f1 (ppm)

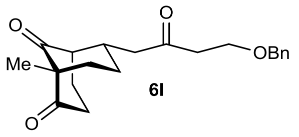
O
O
Me
OBn
6l
O

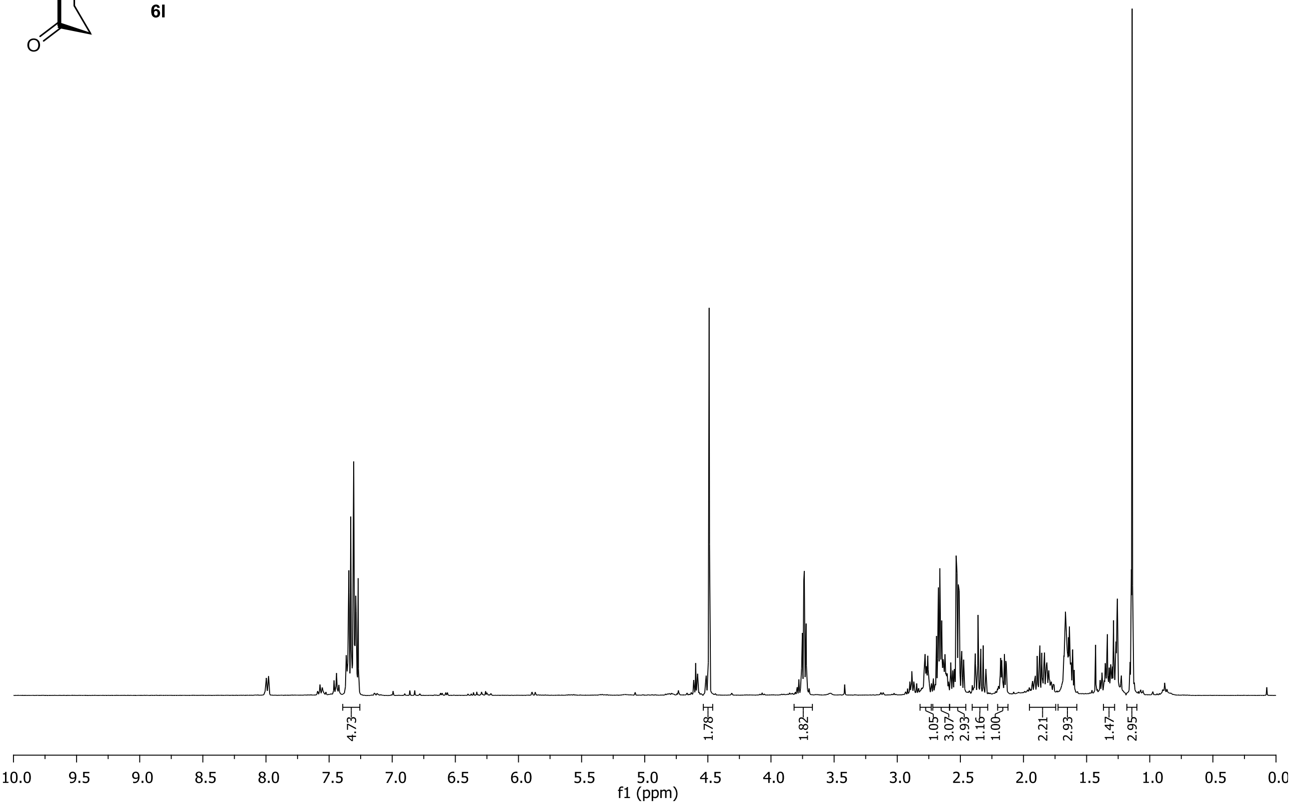
4.73
1.78
1.82
1.05
3.07
2.93
1.16
1.00
2.21
2.93
1.47
2.95
10.0 9.5 9.0 8.5 8.0 7.5 7.0 6.5 6.0 5.5 5.0 4.5 4.0 3.5 3.0 2.5 2.0 1.5 1.0 0.5 0.0
f1 (ppm)

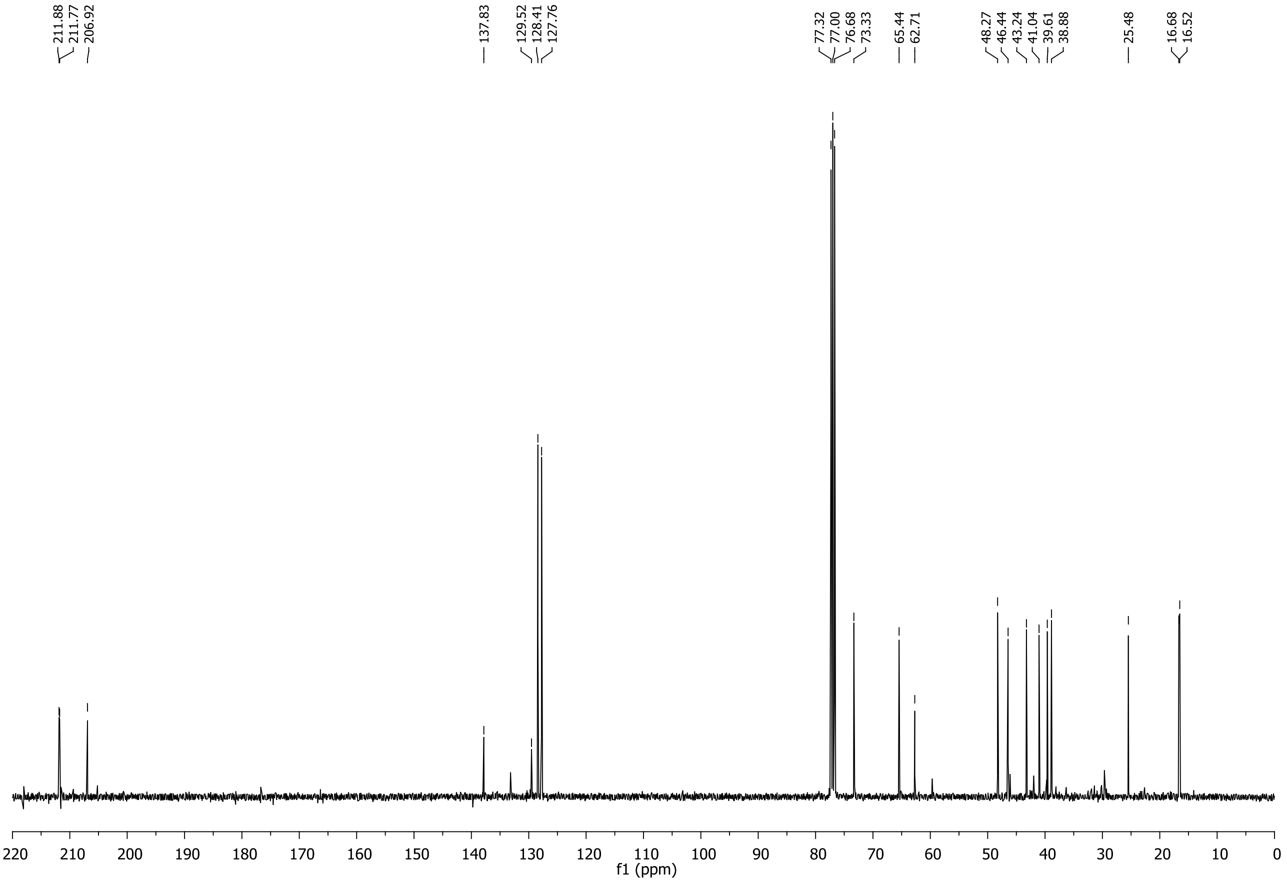
211.88
211.77
206.92
137.83
129.52
128.41
127.76
77.32
77.00
76.68
73.33
65.44
62.71
48.27
46.44
43.24
41.04
39.61
38.88
25.48
16.68
16.52
220 210 200 190 180 170 160 150 140 130 120 110 100 90 80 70 60 50 40 30 20 10 0
f1 (ppm)

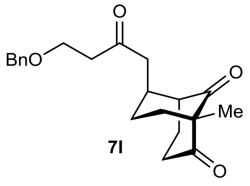
O
BnO
O
Me
O
7l

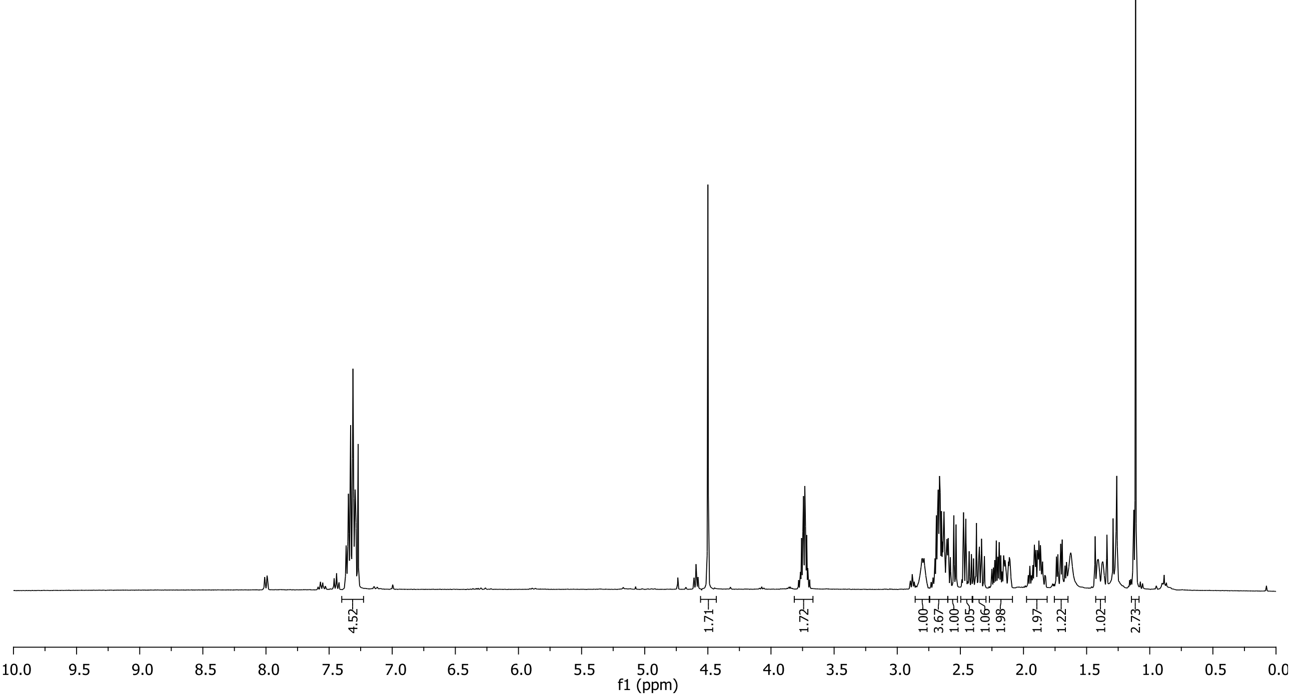
4.52
1.71
1.72
1.00
3.67
1.00
1.05
1.06
1.98
1.97
1.22
1.02
2.73
10.0 9.5 9.0 8.5 8.0 7.5 7.0 6.5 6.0 5.5 5.0 4.5 4.0 3.5 3.0 2.5 2.0 1.5 1.0 0.5 0.0
f1 (ppm)

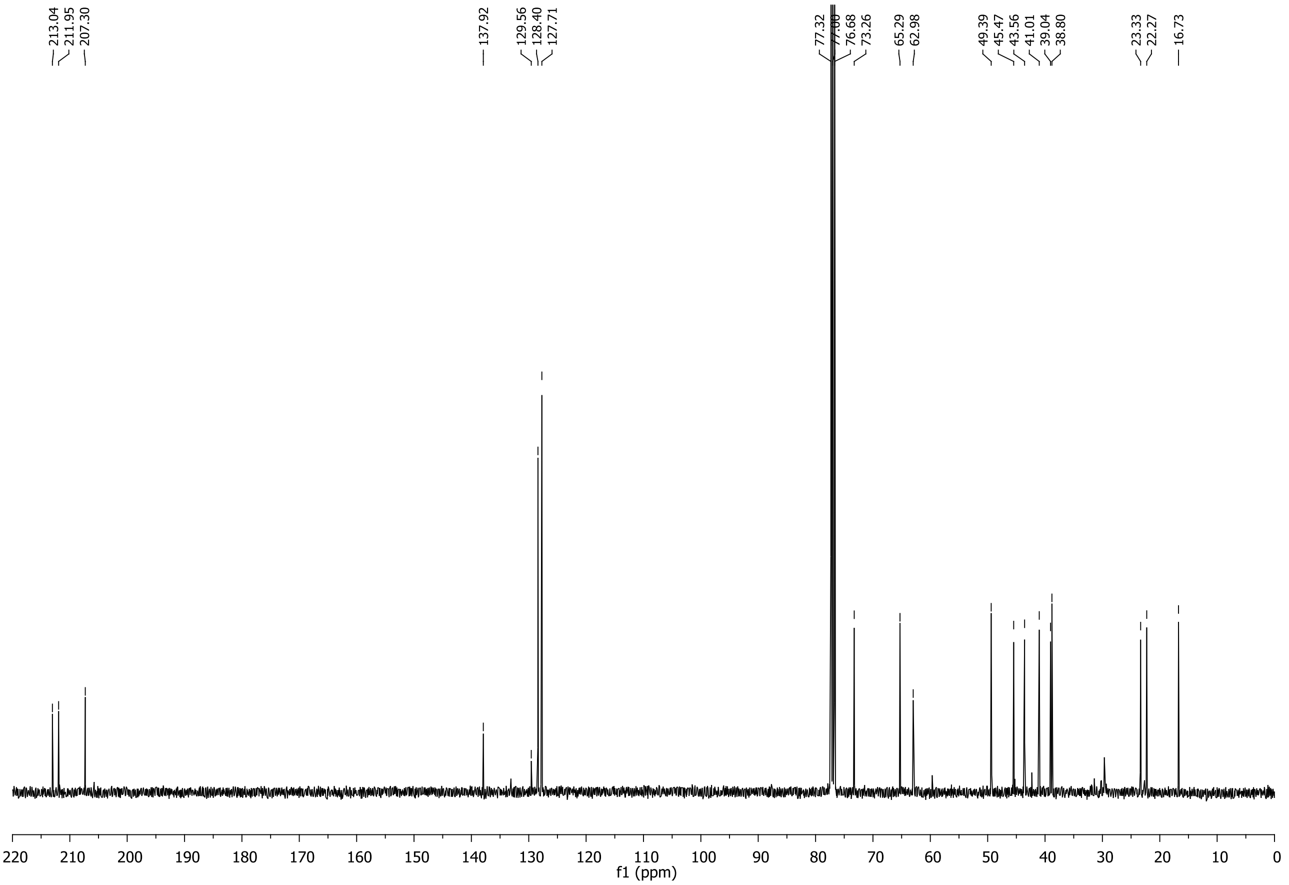
213.04
211.95
207.30
137.92
129.56
128.40
127.71
77.32
76.68
73.26
65.29
62.98
49.39
45.47
43.56
41.01
39.04
38.80
23.33
22.27
16.73
220 210 200 190 180 170 160 150 140 130 120 110 100 90 80 70 60 50 40 30 20 10 0
f1 (ppm)

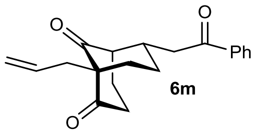
O
O
Ph
O
6m

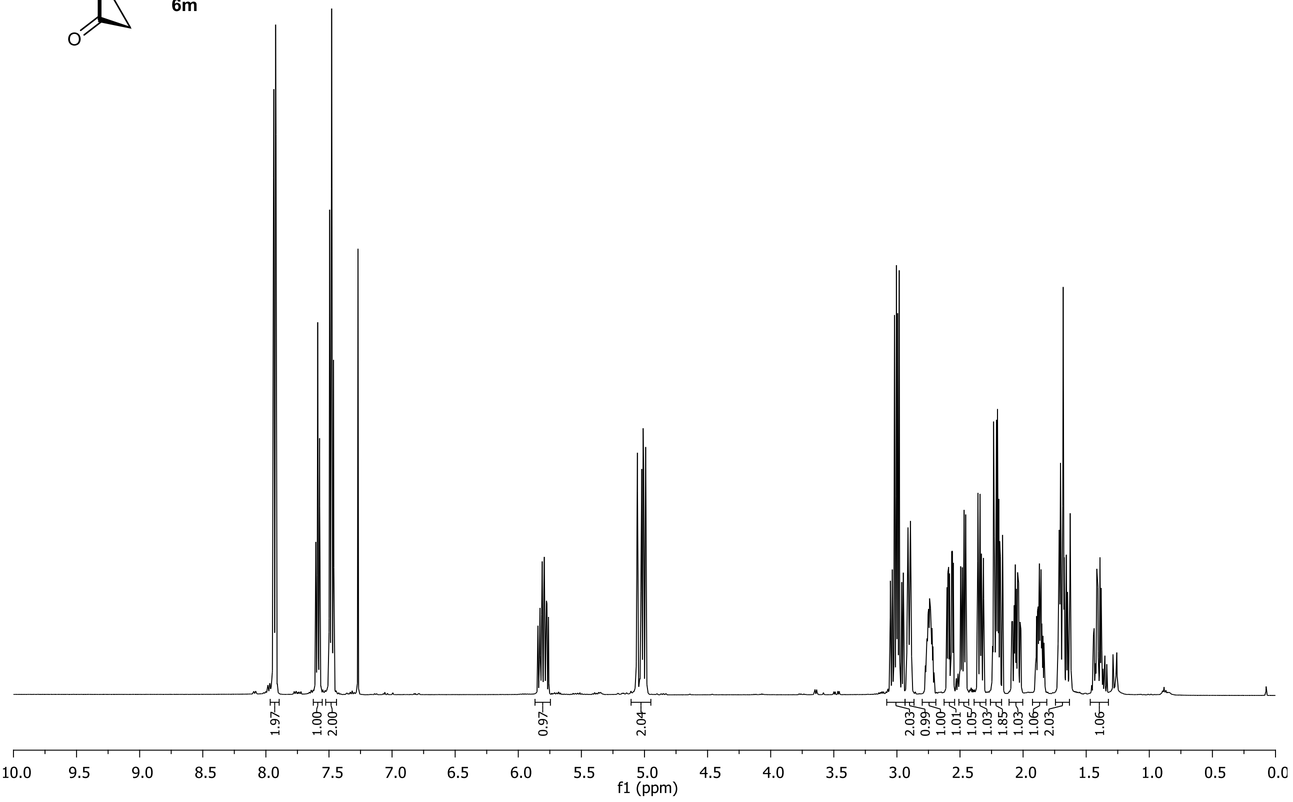
1.97
1.00
2.00
0.97
2.04
2.03
0.99
1.00
1.01
1.05
1.03
1.85
1.03
1.06
2.03
1.06
10.0 9.5 9.0 8.5 8.0 7.5 7.0 6.5 6.0 5.5 5.0 4.5 4.0 3.5 3.0 2.5 2.0 1.5 1.0 0.5 0.0
f1 (ppm)

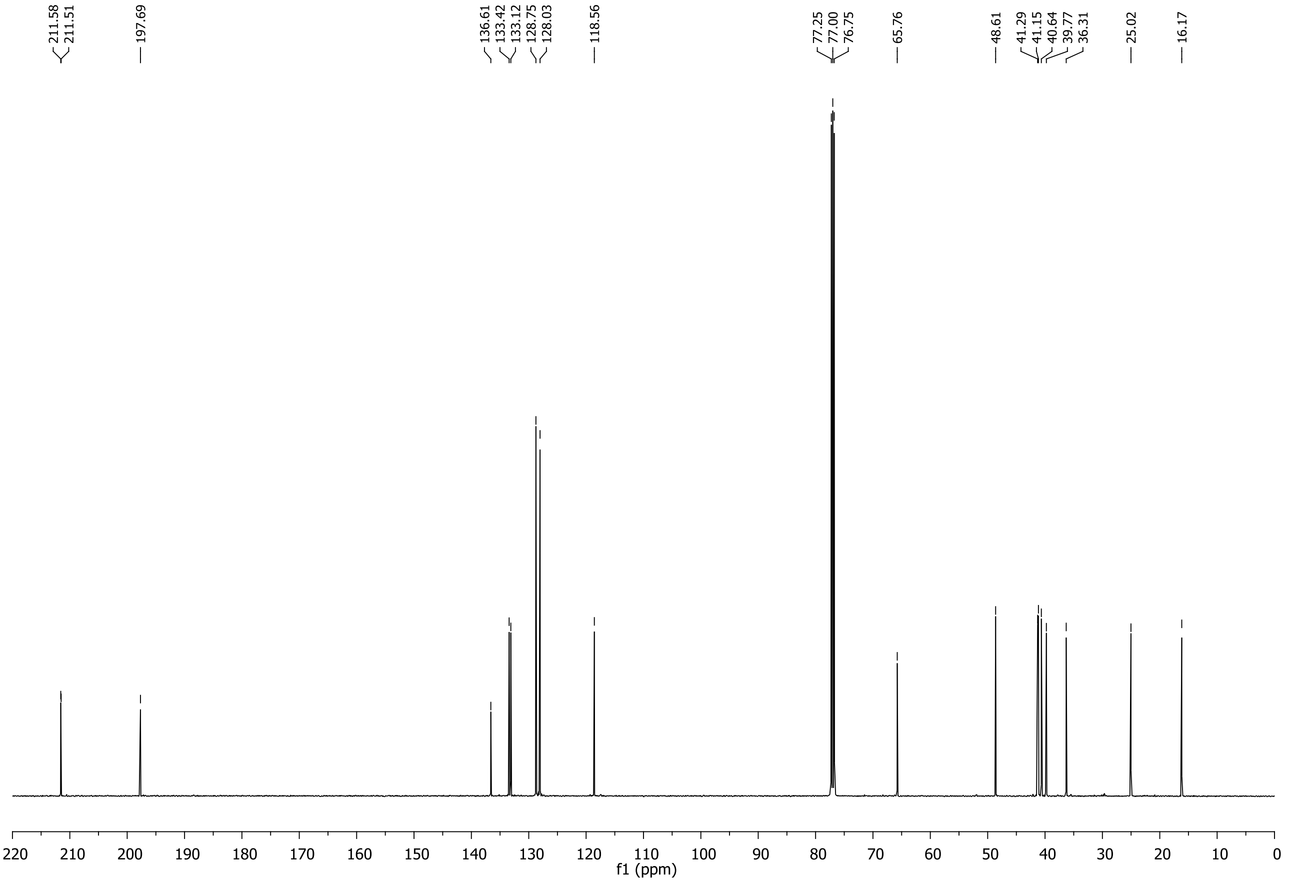
211.58
211.51
197.69
136.61
133.42
133.12
128.75
128.03
118.56
77.25
77.00
76.75
65.76
48.61
41.29
41.15
40.64
39.77
36.31
25.02
16.17
220 210 200 190 180 170 160 150 140 130 120 110 100 90 80 70 60 50 40 30 20 10 0
f1 (ppm)

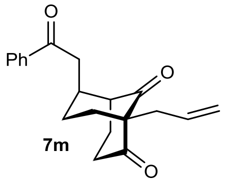
Ph
O
O
O

7m

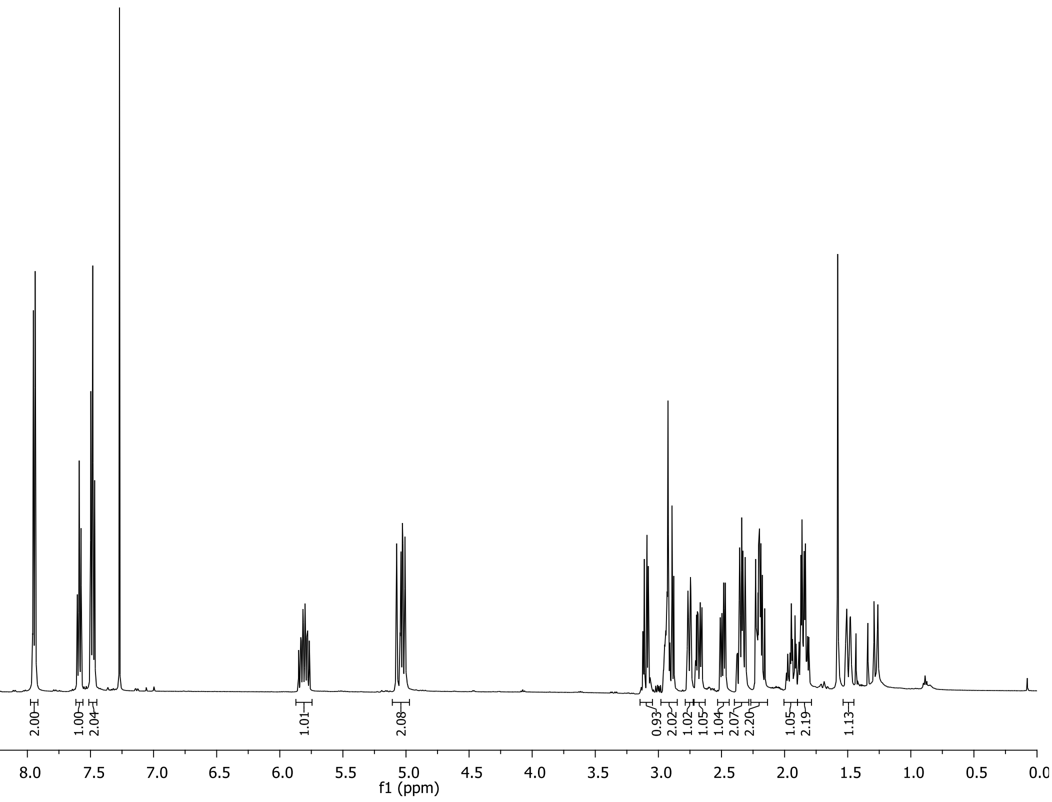
2.00
1.00
2.04
1.01
2.08
0.93
2.02
1.02
1.05
1.04
2.07
2.20
1.05
2.19
1.13
10.0 9.5 9.0 8.5 8.0 7.5 7.0 6.5 6.0 5.5 5.0 4.5 4.0 3.5 3.0 2.5 2.0 1.5 1.0 0.5 0.0
f1 (ppm)

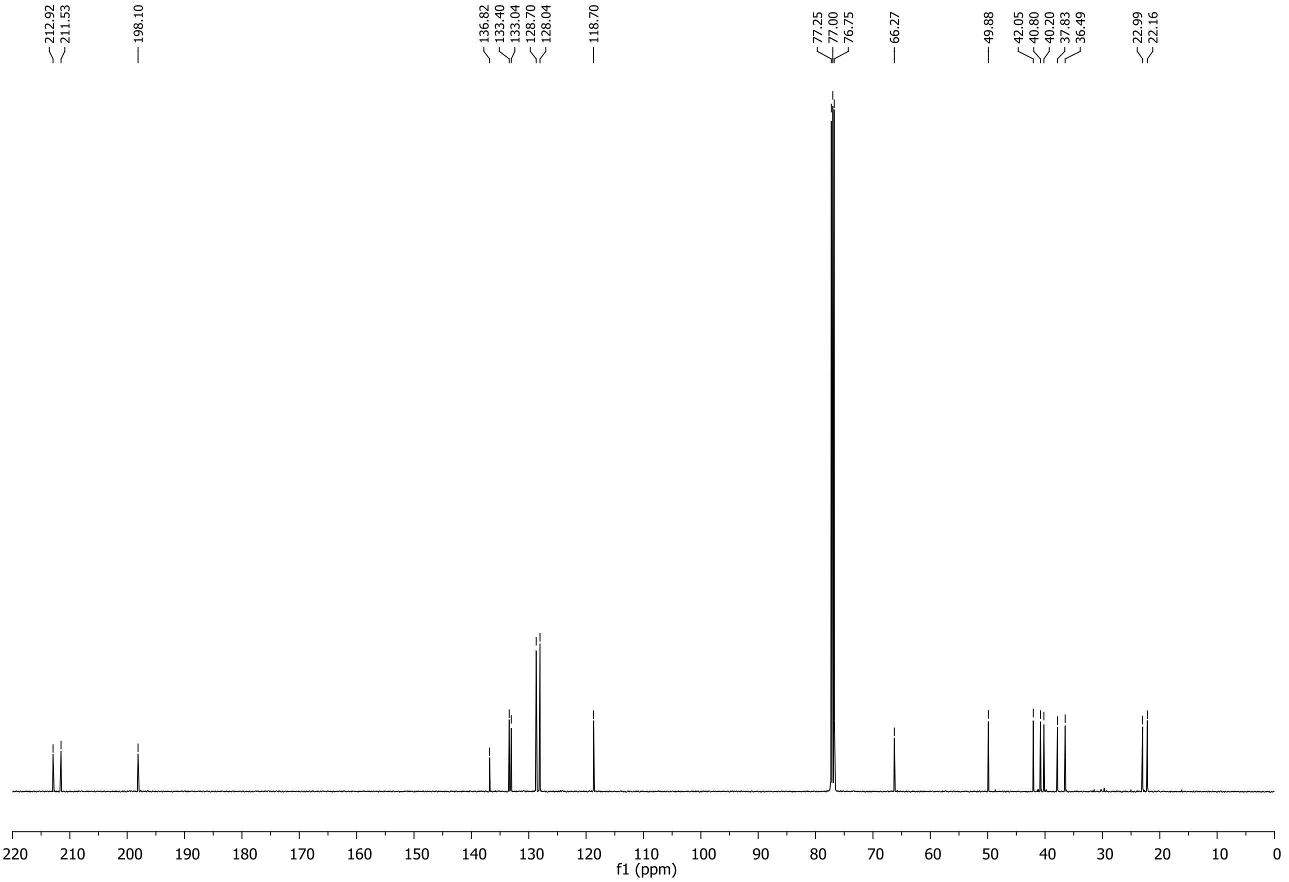
212.92
211.53
198.10
136.82
133.40
133.04
128.70
128.04
118.70
77.25
77.00
76.75
66.27
49.88
42.05
40.80
40.20
37.83
36.49
22.99
22.16
220 210 200 190 180 170 160 150 140 130 120 110 100 90 80 70 60 50 40 30 20 10 0
f1 (ppm)

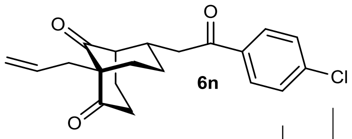
O
O
O
6n
Cl

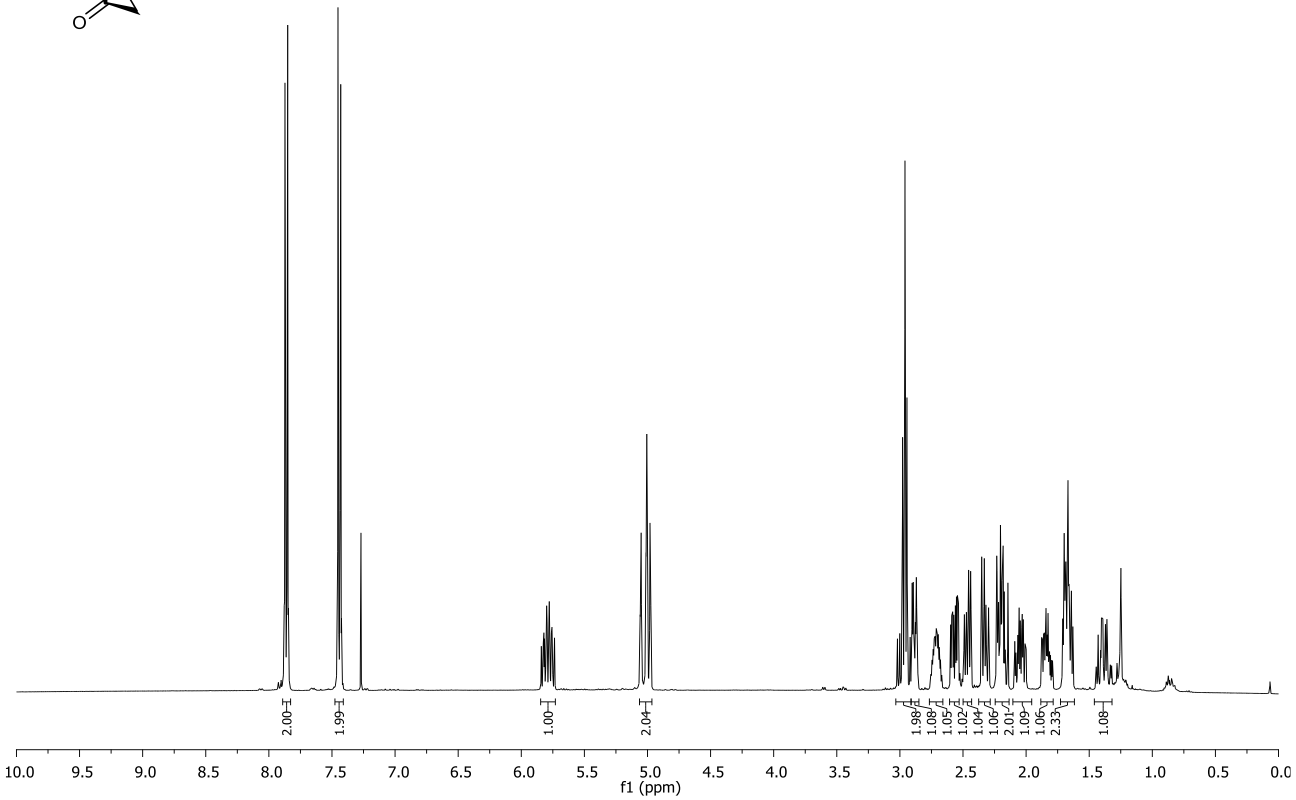
2.00
1.99
1.00
2.04
1.98
1.08
1.05
1.02
1.04
1.06
2.01
1.09
1.06
2.33
1.08
10.0 9.5 9.0 8.5 8.0 7.5 7.0 6.5 6.0 5.5 5.0 4.5 4.0 3.5 3.0 2.5 2.0 1.5 1.0 0.5 0.0
f1 (ppm)

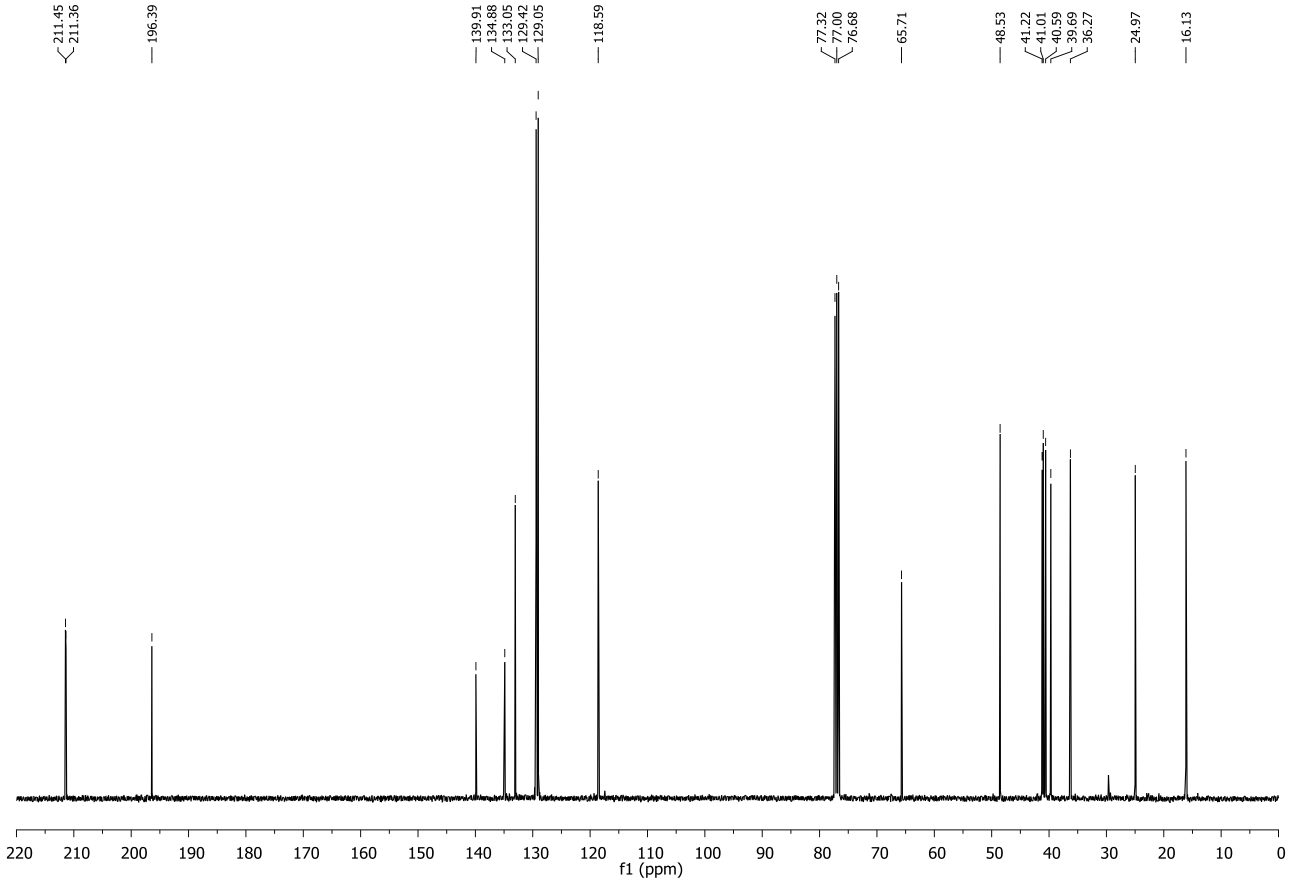
211.45
211.36
196.39
139.91
134.88
133.05
129.42
129.05
118.59
77.32
77.00
76.68
65.71
48.53
41.22
41.01
40.59
39.69
36.27
24.97
16.13
220 210 200 190 180 170 160 150 140 130 120 110 100 90 80 70 60 50 40 30 20 10 0
f1 (ppm)

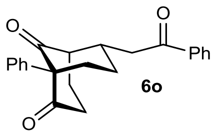
O
O
Ph
Ph
O

6o

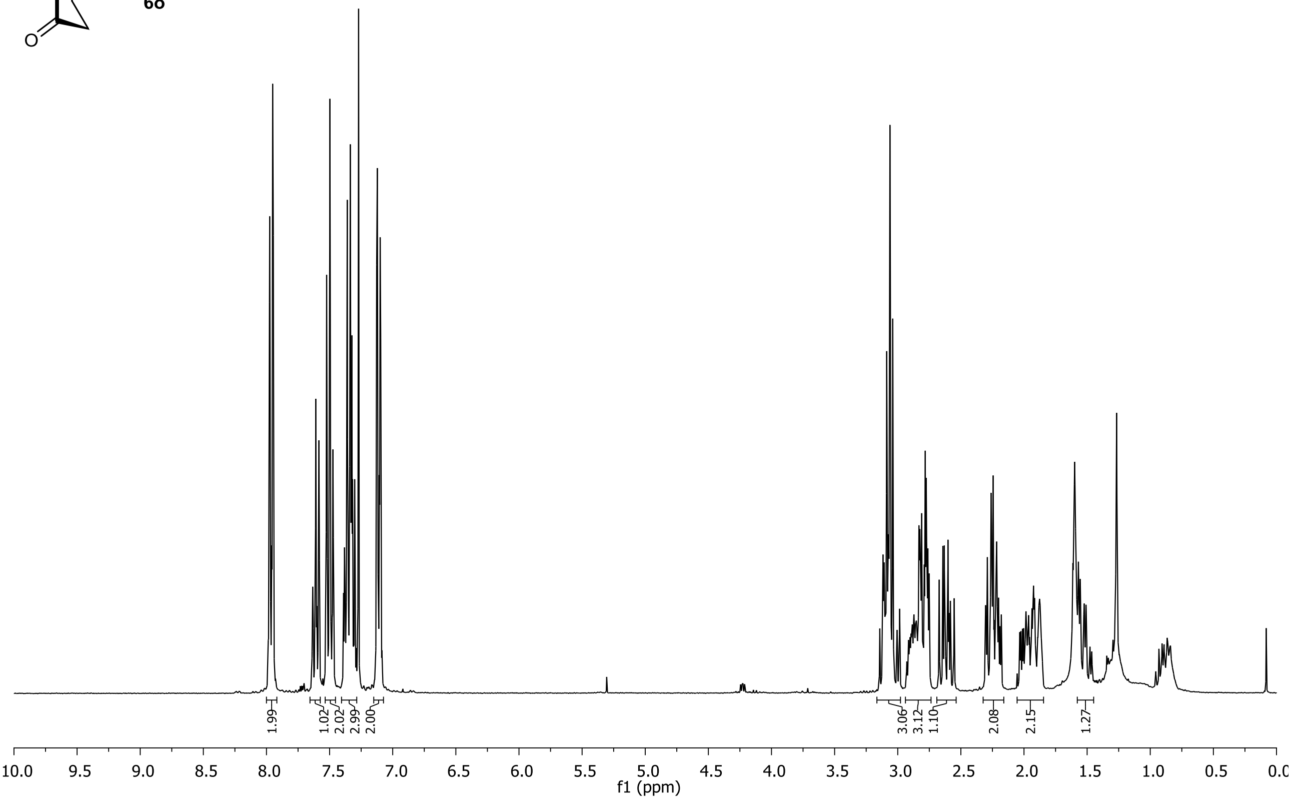
1.99
1.02
2.02
2.99
2.00
3.06
3.12
1.10
2.08
2.15
1.27
10.0 9.5 9.0 8.5 8.0 7.5 7.0 6.5 6.0 5.5 5.0 4.5 4.0 3.5 3.0 2.5 2.0 1.5 1.0 0.5 0.0
f1 (ppm)

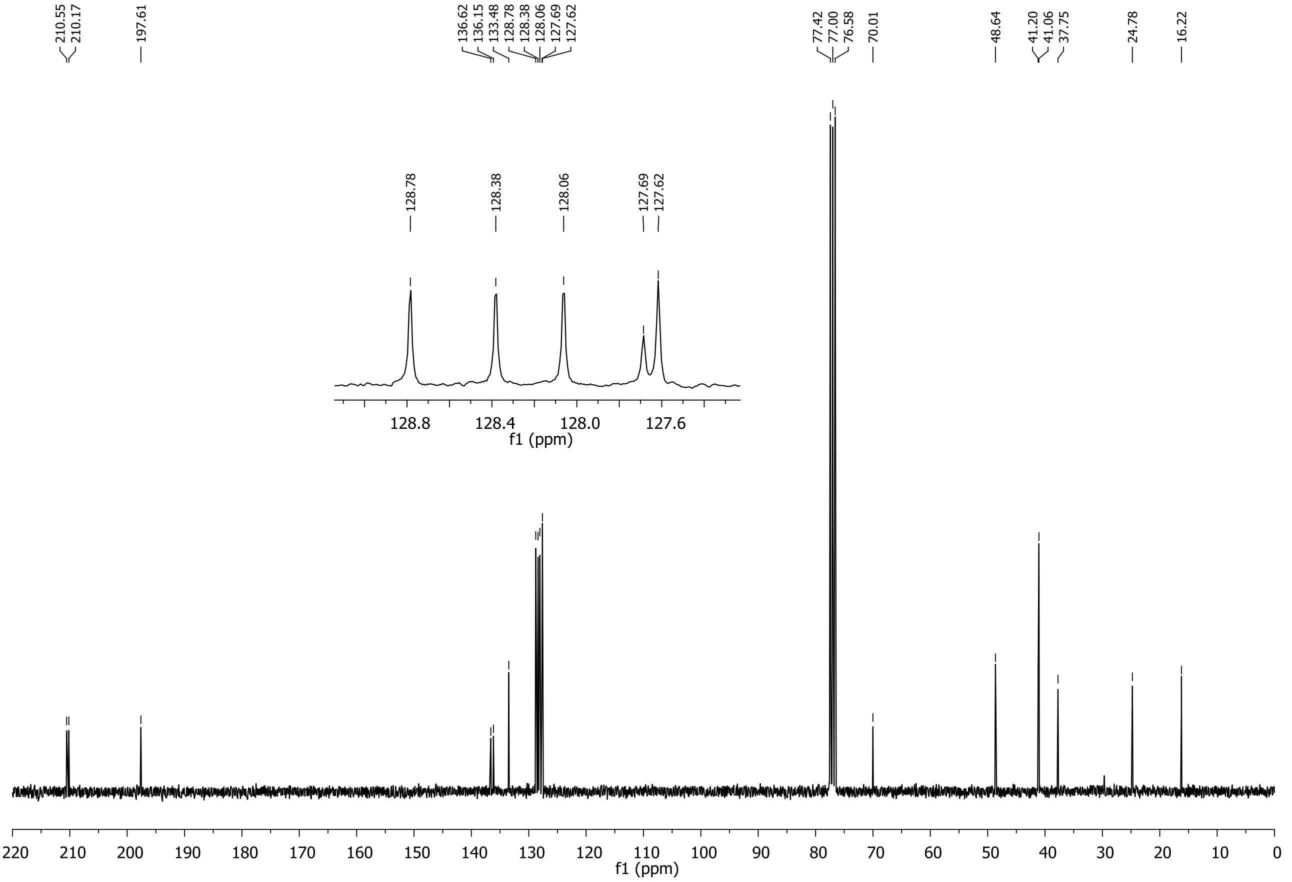
210.55
210.17
197.61
136.62
136.15
133.48
128.78
128.38
128.06
127.69
127.62
77.42
77.00
76.58
70.01
48.64
41.20
41.06
37.75
24.78
16.22
128.78
128.38
128.06
127.69
127.62
128.8 128.4 128.0 127.6
f1 (ppm)
220 210 200 190 180 170 160 150 140 130 120 110 100 90 80 70 60 50 40 30 20 10 0
f1 (ppm)

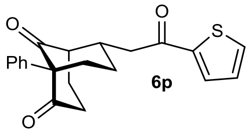
O
O
S
Ph
O

6p

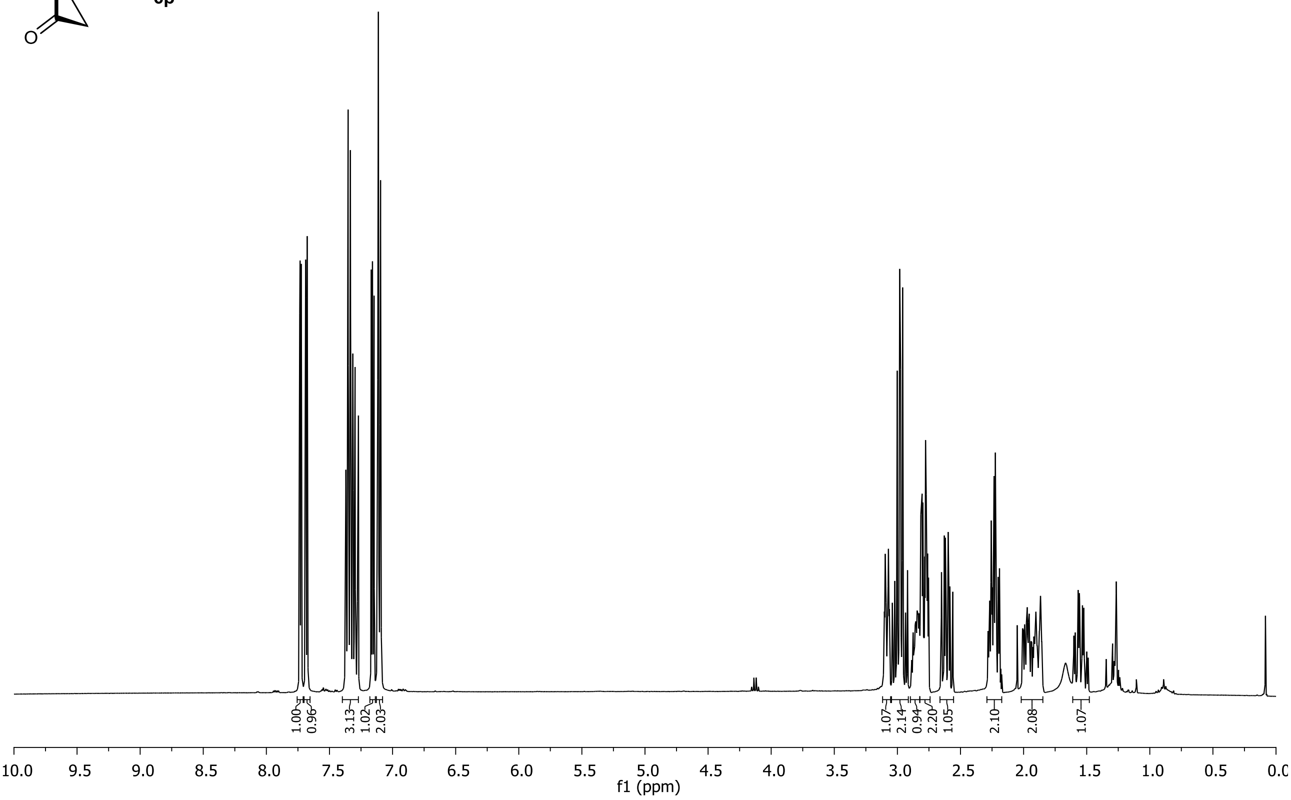
1.00
0.96
3.13
1.02
2.03
1.07
2.14
0.94
2.20
1.05
2.10
2.08
1.07
10.0 9.5 9.0 8.5 8.0 7.5 7.0 6.5 6.0 5.5 5.0 4.5 4.0 3.5 3.0 2.5 2.0 1.5 1.0 0.5 0.0
f1 (ppm)

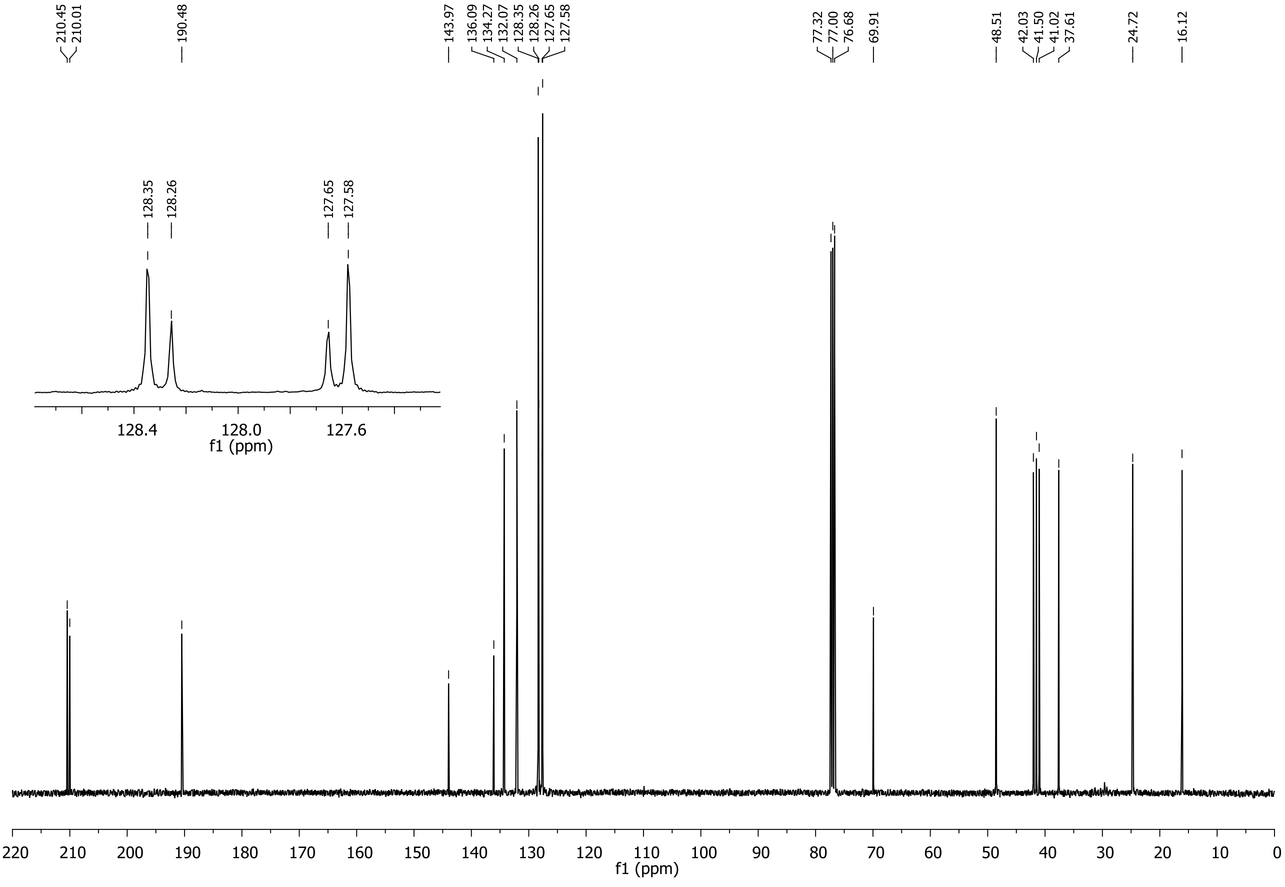
210.45
210.01
190.48
143.97
136.09
134.27
132.07
128.35
128.26
127.65
127.58
77.32
77.00
76.68
69.91
48.51
42.03
41.50
41.02
37.61
24.72
16.12
128.35
128.26
127.65
127.58
128.4 128.0 127.6
f1 (ppm)
220 210 200 190 180 170 160 150 140 130 120 110 100 90 80 70 60 50 40 30 20 10 0
f1 (ppm)

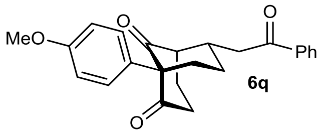
MeO
O
O
O
Ph

6q

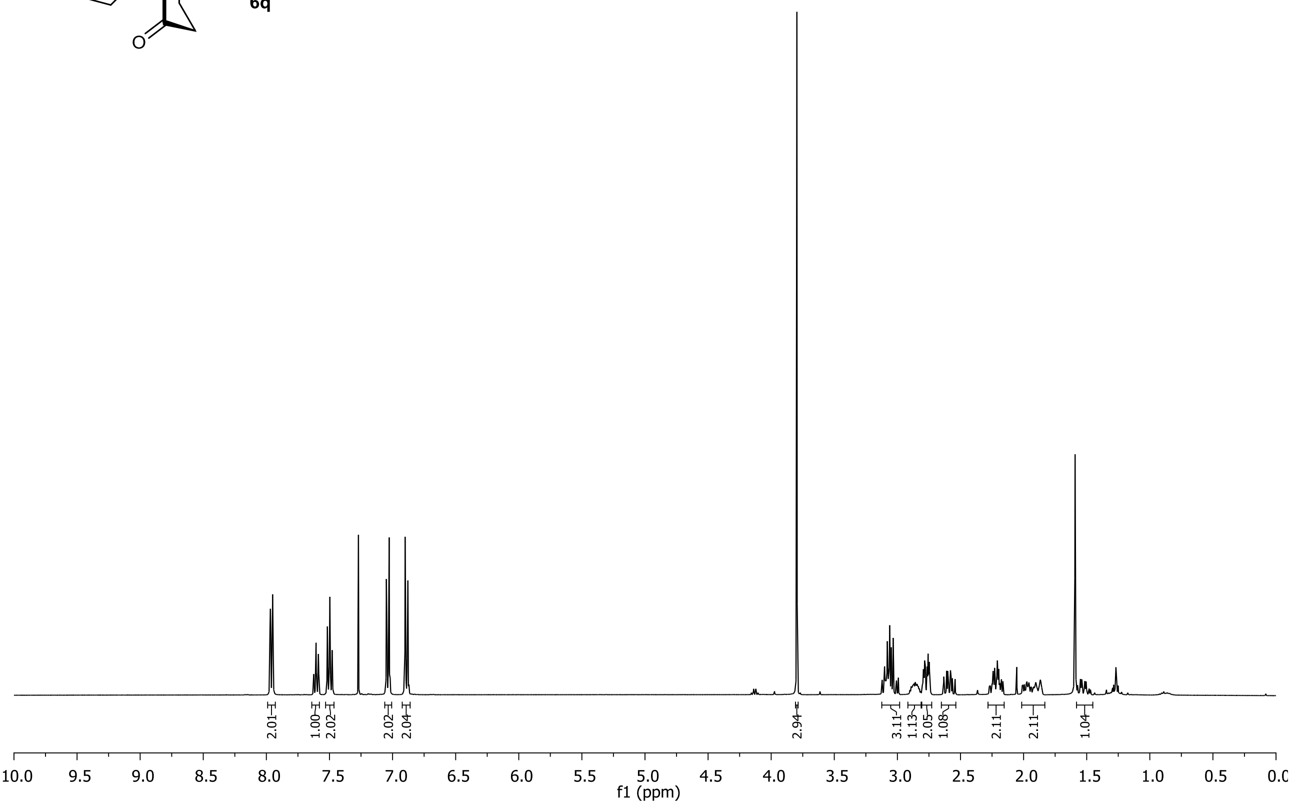
2.01
1.00
2.02
2.02
2.04
2.94
3.11
1.13
2.05
1.08
2.11
2.11
1.04
10.0 9.5 9.0 8.5 8.0 7.5 7.0 6.5 6.0 5.5 5.0 4.5 4.0 3.5 3.0 2.5 2.0 1.5 1.0 0.5 0.0
f1 (ppm)

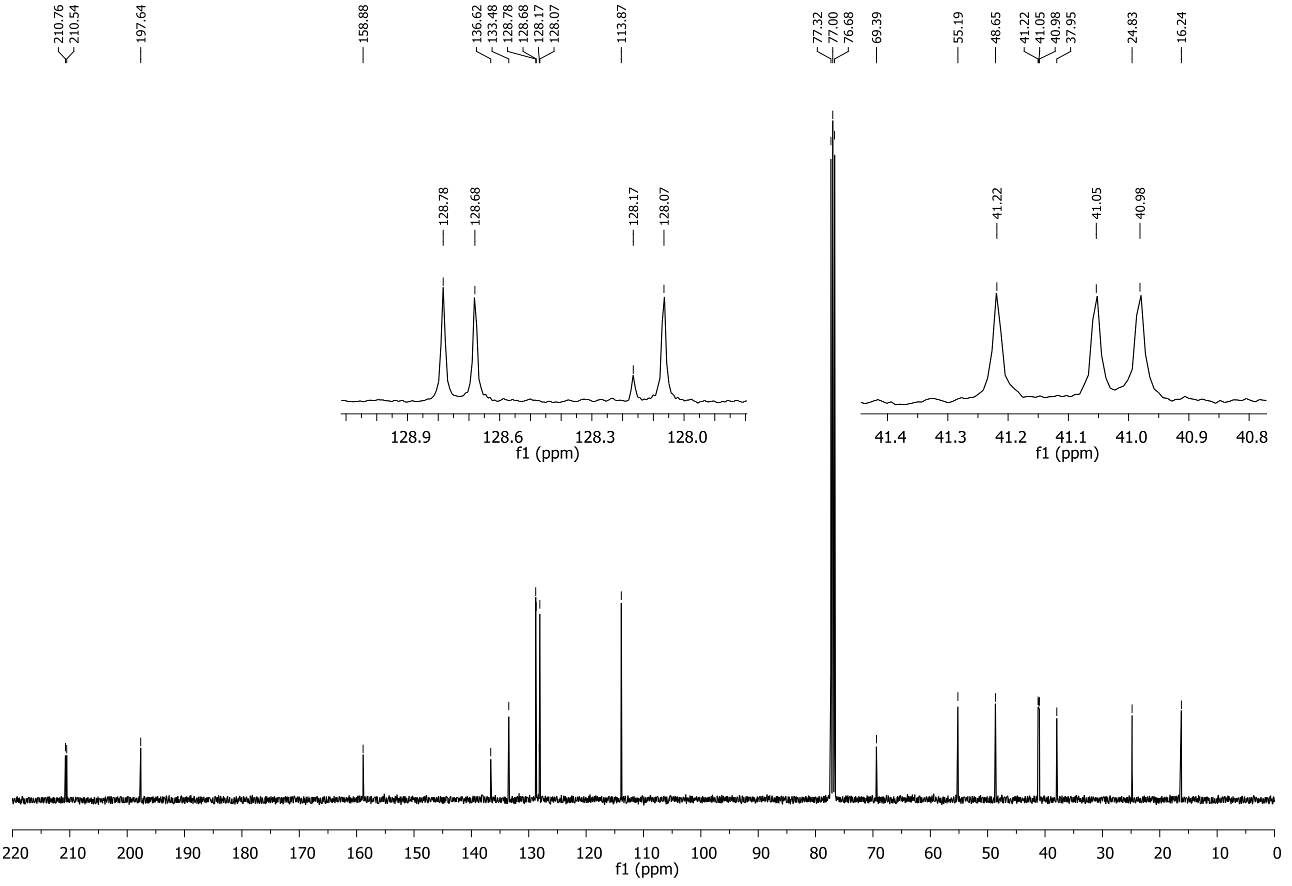
210.76
210.54
197.64
158.88
136.62
133.48
128.78
128.68
128.17
128.07
113.87
77.32
77.00
76.68
69.39
55.19
48.65
41.22
41.05
40.98
37.95
24.83
16.24
128.9 128.6 128.3 128.0
f1 (ppm)
41.4 41.3 41.2 41.1 41.0 40.9 40.8
f1 (ppm)
220 210 200 190 180 170 160 150 140 130 120 110 100 90 80 70 60 50 40 30 20 10 0
f1 (ppm)

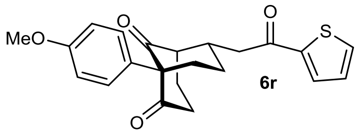
MeO
O
O
S
O

6r

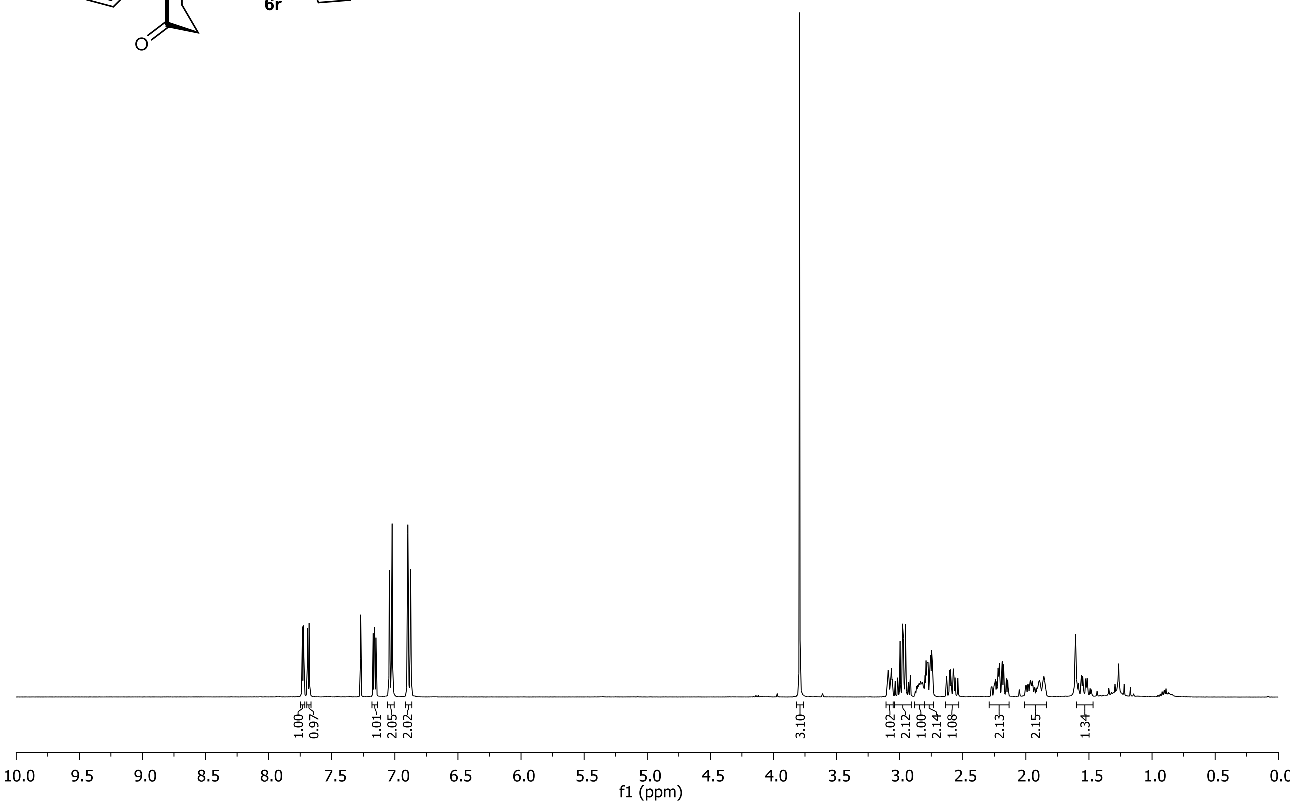
1.00
0.97
1.01
2.05
2.02
3.10
1.02
2.12
1.00
2.14
1.08
2.13
2.15
1.34
f1 (ppm)

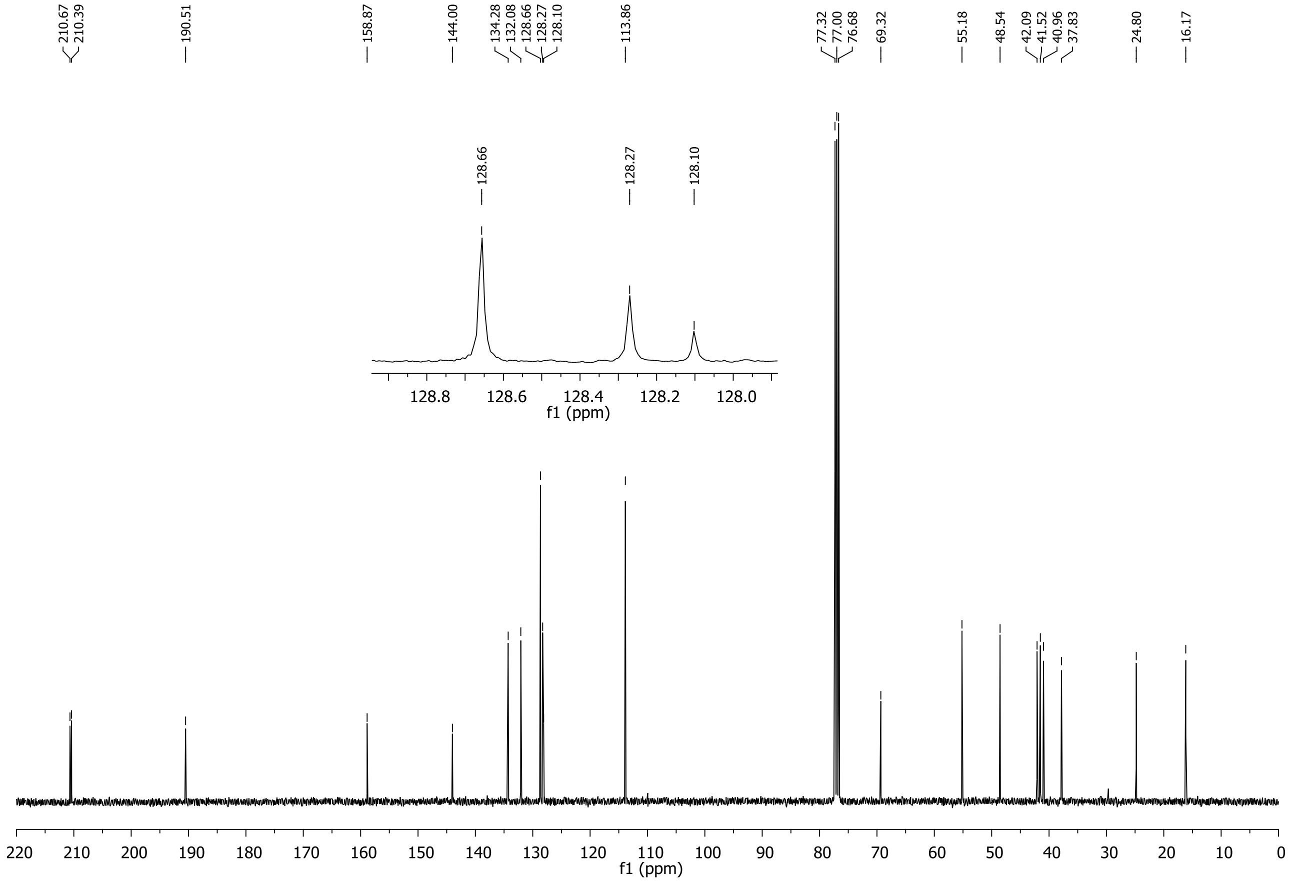
210.67
210.39
190.51
158.87
144.00
134.28
132.08
128.66
128.27
128.10
113.86
77.32
77.00
76.68
69.32
55.18
48.54
42.09
41.52
40.96
37.83
24.80
16.17
f1 (ppm)

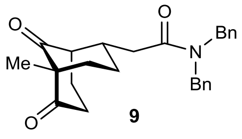
O
O
Me
N
Bn
Bn
O
9

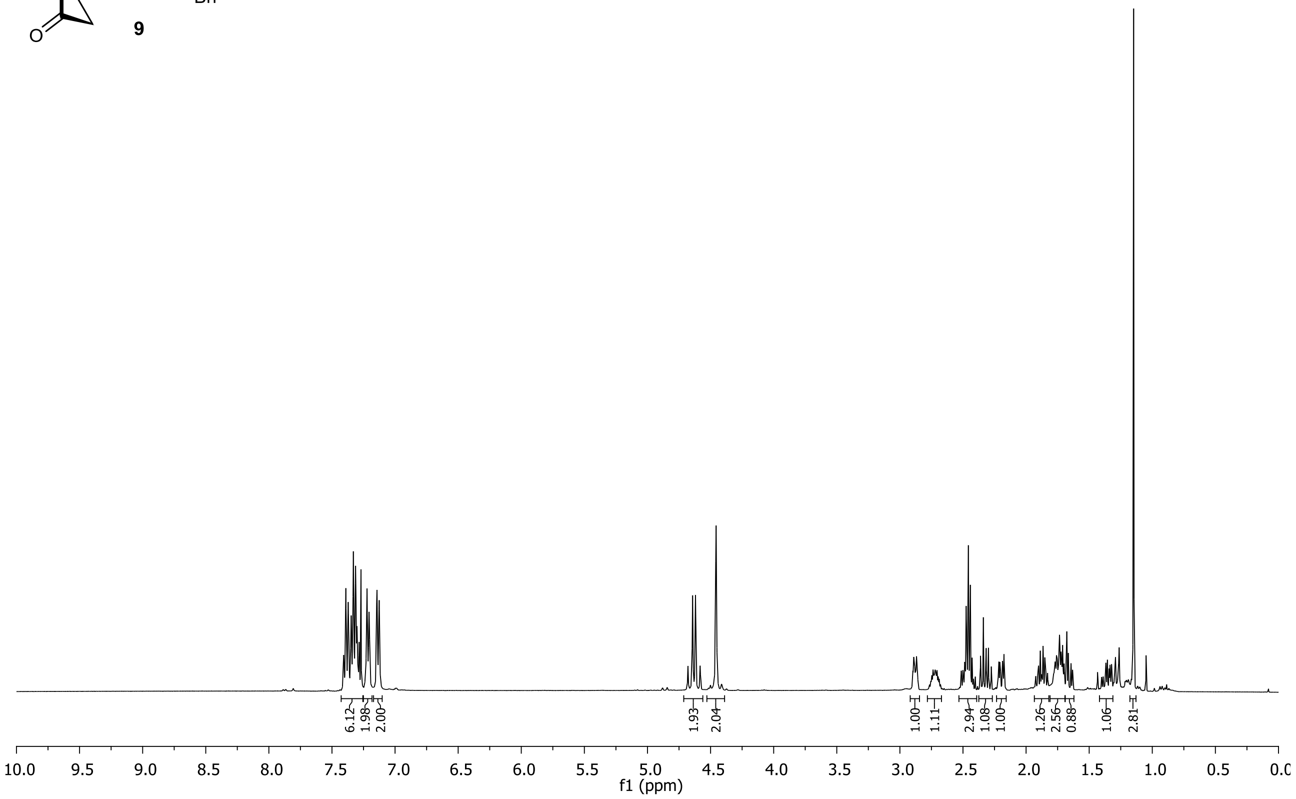
6.12
1.98
2.00
1.93
2.04
1.00
1.11
2.94
1.08
1.00
1.26
2.56
0.88
1.06
2.81
f1 (ppm)

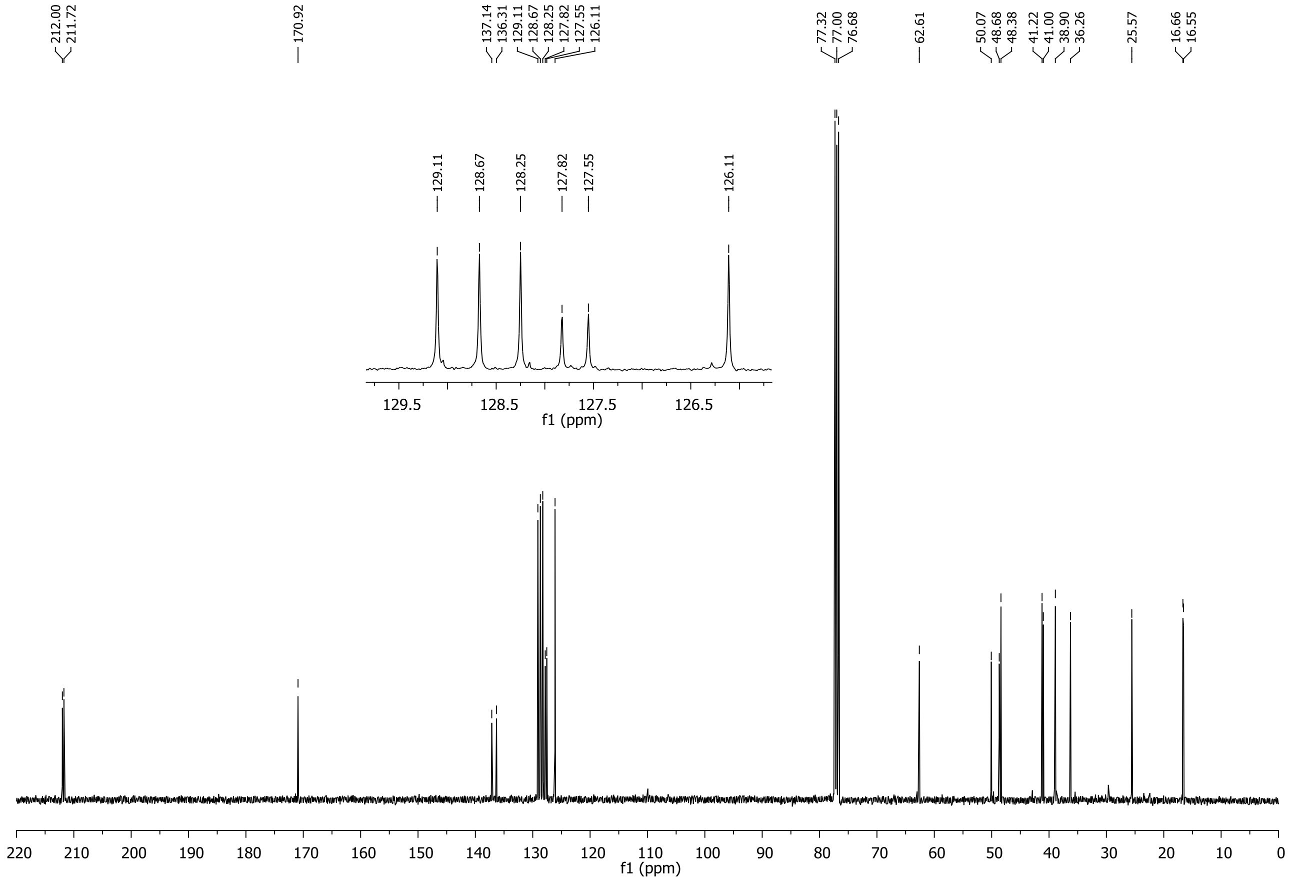
212.00
211.72
170.92
137.14
136.31
129.11
128.67
128.25
127.82
127.55
126.11
77.32
77.00
76.68
62.61
50.07
48.68
48.38
41.22
41.00
38.90
36.26
25.57
16.66
16.55
f1 (ppm)

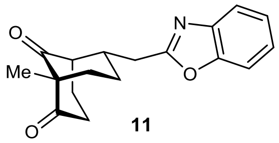
O
Me
O
N
O

11

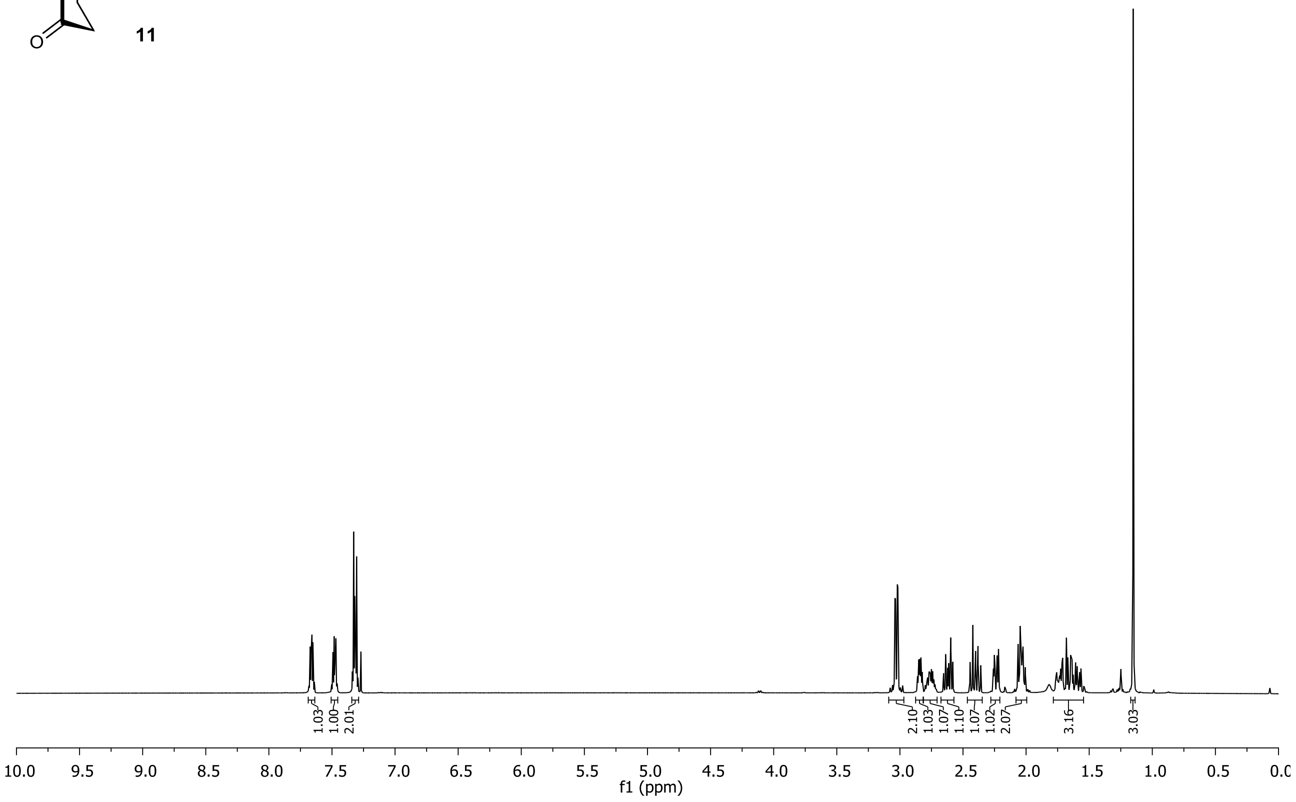
1.03
1.00
2.01
2.10
1.03
1.07
1.10
1.07
1.02
2.07
3.16
3.03
10.0 9.5 9.0 8.5 8.0 7.5 7.0 6.5 6.0 5.5 5.0 4.5 4.0 3.5 3.0 2.5 2.0 1.5 1.0 0.5 0.0
f1 (ppm)

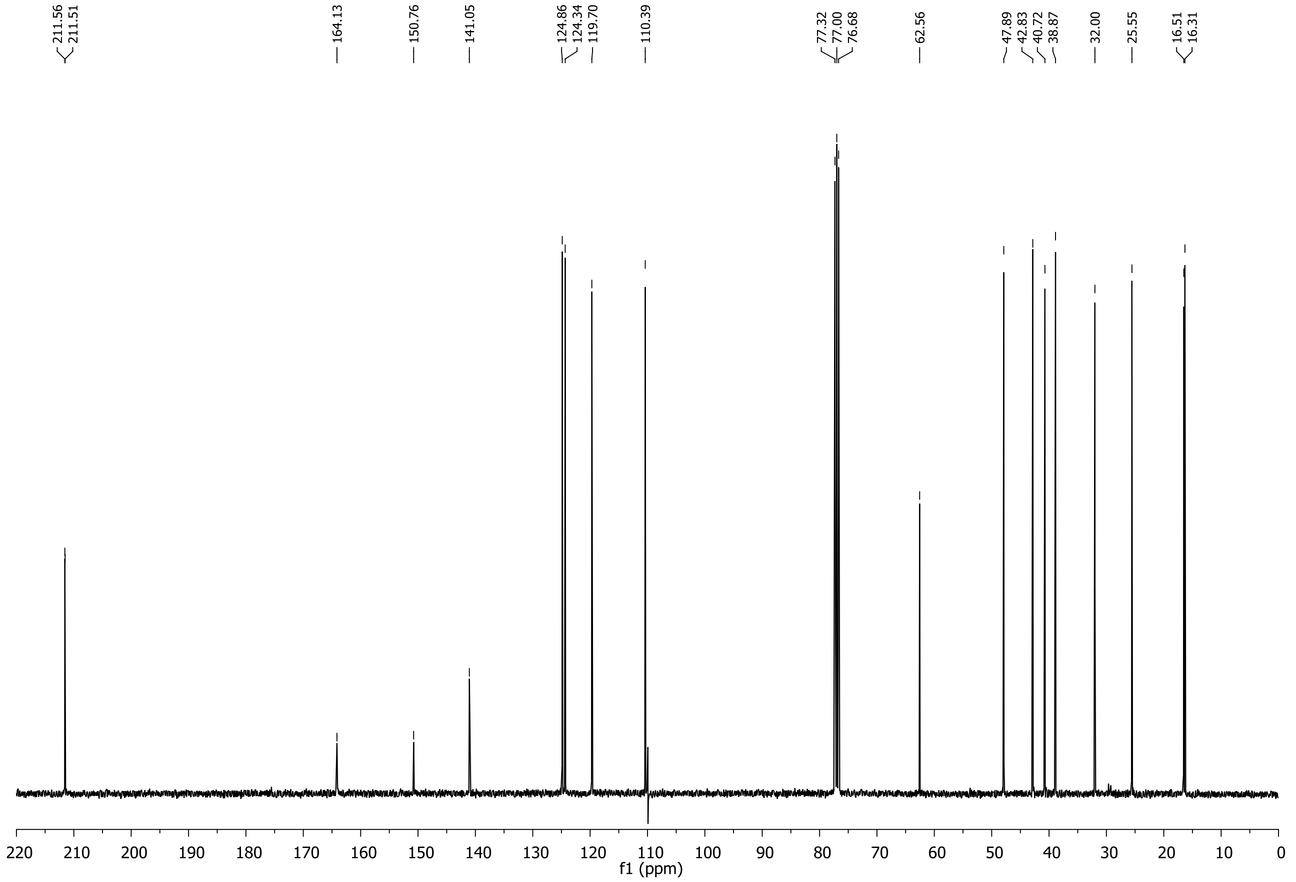
211.56
211.51
164.13
150.76
141.05
124.86
124.34
119.70
110.39
77.32
77.00
76.68
62.56
47.89
42.83
40.72
38.87
32.00
25.55
16.51
16.31
220 210 200 190 180 170 160 150 140 130 120 110 100 90 80 70 60 50 40 30 20 10 0
f1 (ppm)

HPLC Traces

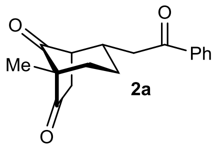

2a

data acquired by: ARB
on: 4/2/2014
location: Vial 57

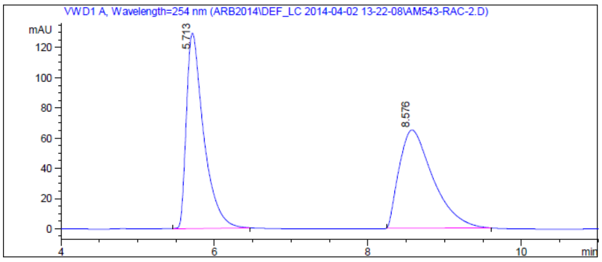

Meas. R	Area %	Width	Symmetr.
5.713	50.459	0.261	0.514
8.576	49.541	0.510	0.588

data acquired by: ARB
on: 4/2/2014
location: Vial 56

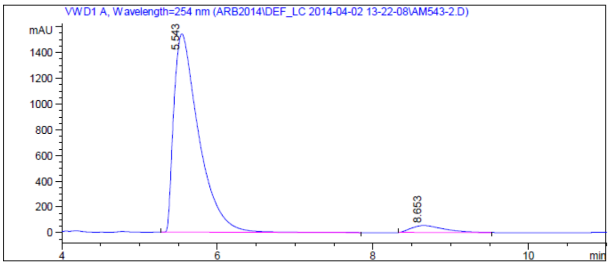

Meas. R	Area %	Width	Symmetr.
5.543	95.449	0.370	0.442
8.653	4.551	0.498	0.575

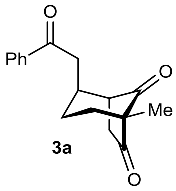

3a

data acquired by: AM
on: 4/15/2014
location: Vial 15

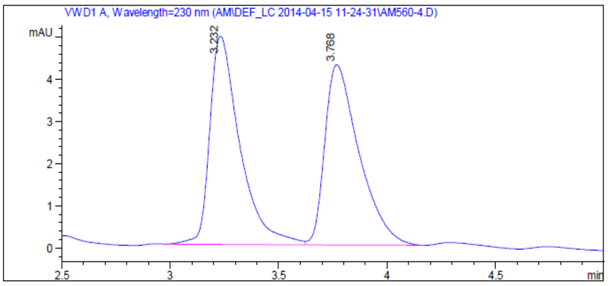

Meas. R	Area %	Width	Symmetr.
3.232	50.355	0.144	0.518
3.768	49.645	0.163	0.499

data acquired by: AM
on: 4/15/2014
location: Vial 16

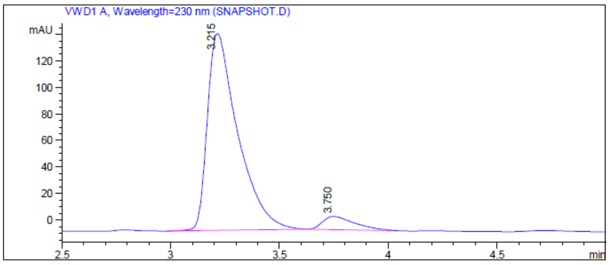

Meas. R	Area %	Width	Symmetr.
3.215	93.448	0.162	0.479
3.750	6.552	0.169	0.509

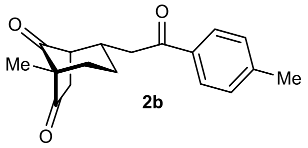

2b

data acquired by: ARB
on: 4/6/2014
location: Vial 77

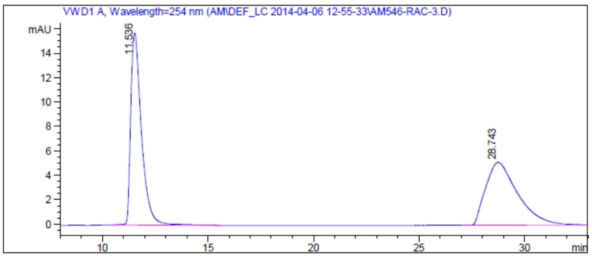

data acquired by: ARB
on: 4/6/2014
location: Vial 76

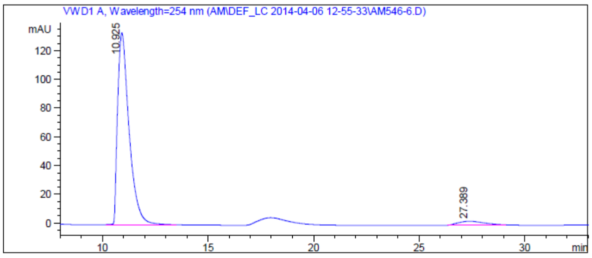

Meas. R	Area %	Width	Symmetr.
11.536	50.600	0.530	0.531
28.743	49.400	1.538	0.583

Meas. R	Area %	Width	Symmetr.
10.925	95.824	0.633	0.569
27.389	4.176	1.374	0.727

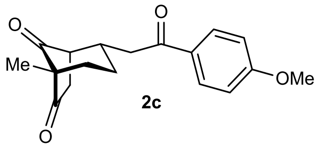

2c

data acquired by: ARB
on: 4/4/2014
location: Vial 77

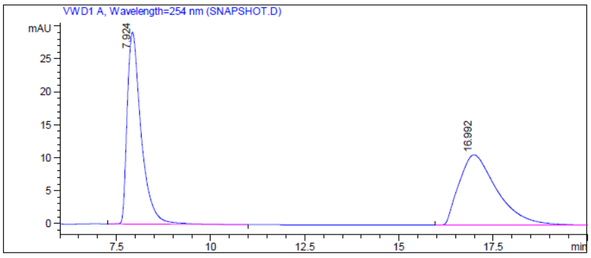

data acquired by: ARB
on: 4/4/2014
location: Vial 76

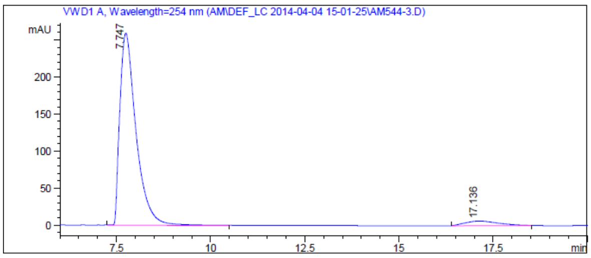

Meas. R	Area %	Width	Symmetr.
7.924	50.273	0.392	0.547
16.992	49.727	1.105	0.605

Meas. R	Area %	Width	Symmetr.
7.747	95.322	0.499	0.548
17.136	4.678	1.015	0.722

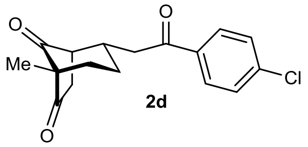

2d

```
data acquired by:   AM
on:                 4/14/2014
location:           Vial 4
```

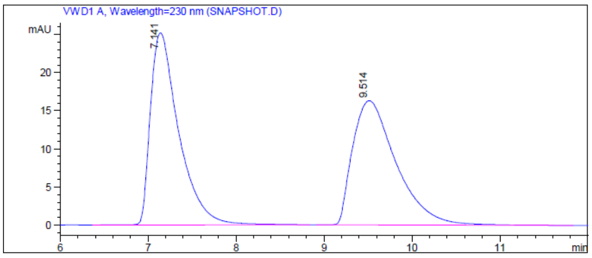


```
data acquired by:   HWL
on:                 12/15/2014
location:           Vial 84
```

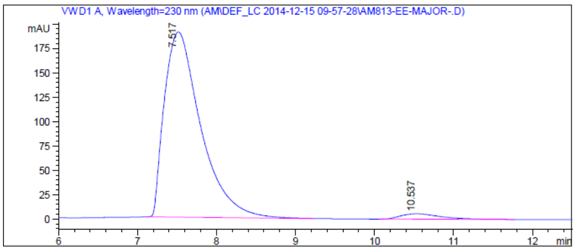

Meas. R	Area %	Width	Symmetr.
7.141	50.185	0.336	0.500
9.514	49.815	0.535	0.572

Meas. R	Area %	Width	Symmetr.
7.517	96.844	0.534	0.582
10.537	3.156	0.594	0.613

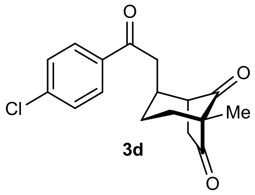

3d

```
data acquired by:   HWL
on:                 11/27/2014
location:           Vial 73
```

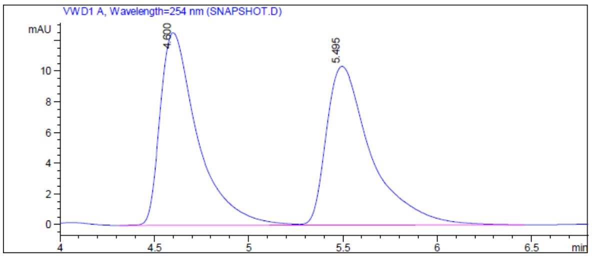


```
data acquired by:   HWL
on:                 11/27/2014
location:           Vial 74
```

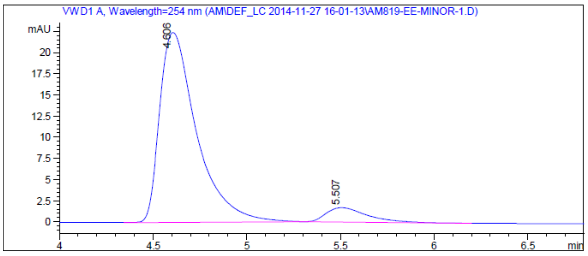

Meas. R	Area %	Width	Symmetr.
4.600	50.329	0.205	0.486
5.495	49.671	0.246	0.485

Meas. R	Area %	Width	Symmetr.
4.606	92.333	0.228	0.542
5.507	7.667	0.252	0.565

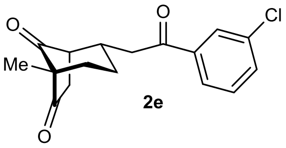

2e

Data file: C:\CHEM32\1\DATA\AMAEL\DEF_LC 2014-05-25 13-25-26\AM590-RAC-2.D
Sample name: AM590-rac-5
Instrument: AGILENT 1260 **Acq. operator:** SYSTEM
Injection date: 5/25/2014 1:37:40 PM
Acq. method: ARB.ADH95B05A.65MI N.1.5ML.10MICROL.M

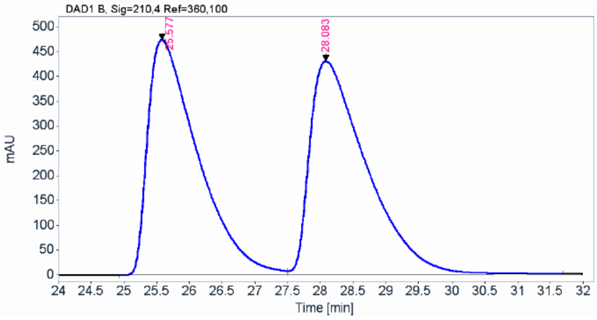

Signal: DAD1 B, Sig=210,4 Ref=360,100

RT [min]	Type	Width [min]	Area	Height	Area%
25.577	BV	0.7828	25158.307	474.3613	49.38
28.083	VB	0.8631	25793.494	430.4529	50.62

Data file: C:\CHEM32\1\DATA\AMAEL\DEF_LC 2014-12-02 11-07-35\AM824-BS-EE-MAJOR.D
Sample name: AM824-bs-ee-major
Instrument: AGILENT 1260 **Acq. operator:** SYSTEM
Injection date: 12/2/2014 2:34:08 PM
Acq. method: ARB.ADH95B05A.65MI N.1.5ML.10MICROL.M

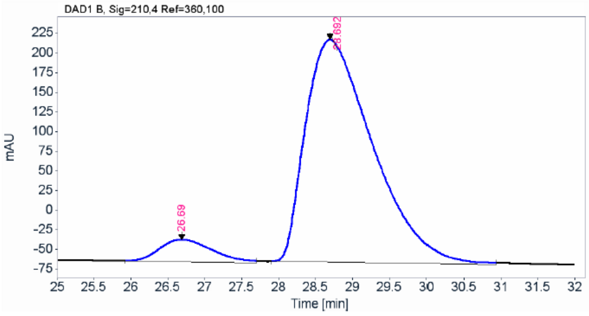

Signal: DAD1 B, Sig=210,4 Ref=360,100

RT [min]	Type	Width [min]	Area	Height	Area%
26.690	MM	0.8299	1397.532	28.0670	7.24
28.692	MM	1.0541	17892.934	282.9161	92.76

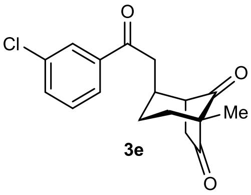

3e

Data file: C:\CHEM32\1\DATA\AMAEL\DEF_LC 2014-11-28 09-51-17\AM582-MINOR-RAC-1.D
Sample name: AM582-minor-rac-1
Instrument: AGILENT 1260 **Acq. operator:** SYSTEM
Injection date: 11/28/2014 1:35:33 PM
Acq. method: ARB.ADH95B10A.65MI N.1.5ML.10MICROL.M

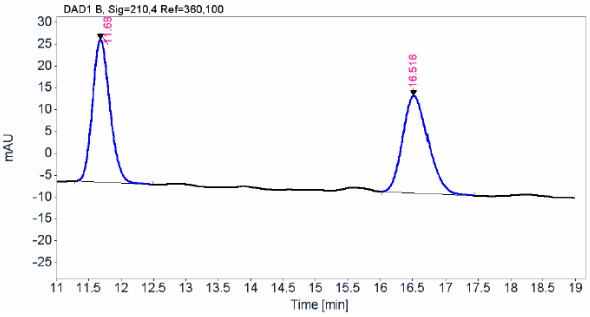

Signal: DAD1 B, Sig=210,4 Ref=360,100

RT [min]	Type	Width [min]	Area	Height	Area%
11.680	BB	0.2881	609.433	32.5929	50.46
16.516	BB	0.4201	598.380	22.2259	49.54

Data file: C:\CHEM32\1\DATA\AMAEL\DEF_LC 2014-12-02 11-07-35\AM824-BS-EE-MINOR-1.D
Sample name: AM824-bs-ee-minor-1
Instrument: AGILENT 1260 **Acq. operator:** SYSTEM
Injection date: 12/2/2014 12:20:22 PM
Acq. method: ARB.ADH95B05A.65MI N.1.5ML.10MICROL.M

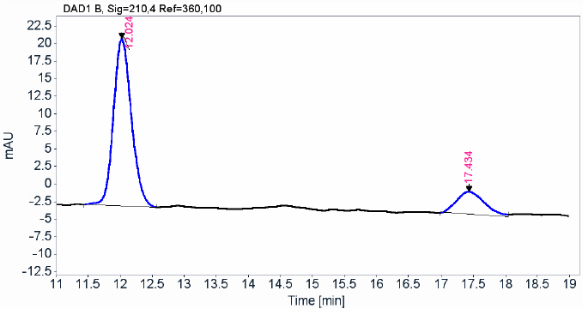

Signal: DAD1 B, Sig=210,4 Ref=360,100

RT [min]	Type	Width [min]	Area	Height	Area%
12.024	BB	0.2987	461.988	23.7714	82.89
17.434	MM	0.4989	95.334	3.1848	17.11

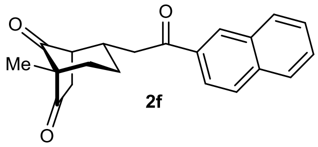

2f

**Data file:**	C:\CHEM32\1\DATA\AMAEL\DEF_LC 2014-04-14 14-37-47\AM549-RAC-2.D		
**Sample name:**	AM549-rac-2		
**Instrument:**	AGILENT 1260	**Acq. operator:**	SYSTEM
**Injection date:**	4/14/2014 5:28:02 PM		
**Acq. method:**	ARB.ADH95B10A.65MI N.1.5ML.10MICROL.M		

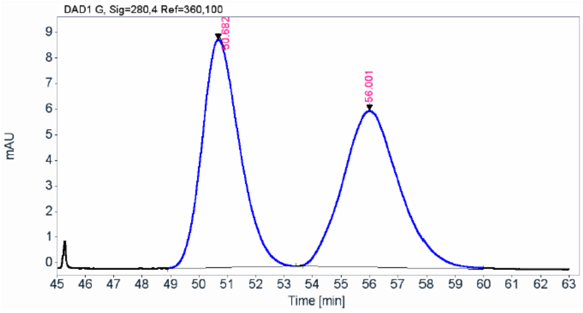

Signal: DAD1 G, Sig=280,4 Ref=360,100

RT [min]	Type	Width [min]	Area	Height	Area%
50.682	BB	1.2927	816.412	8.8956	49.13
56.001	MM	2.3047	845.209	6.1121	50.87

**Data file:**	C:\CHEM32\1\DATA\AMAEL\DEF_LC 2014-04-14 14-37-47\AM549-3.D		
**Sample name:**	AM549-3		
**Instrument:**	AGILENT 1260	**Acq. operator:**	SYSTEM
**Injection date:**	4/14/2014 4:22:03 PM		
**Acq. method:**	ARB.ADH95B10A.65MI N.1.5ML.10MICROL.M		

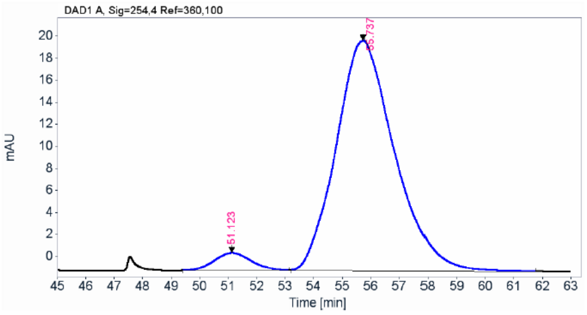

Signal: DAD1 A, Sig=254,4 Ref=360,100

RT [min]	Type	Width [min]	Area	Height	Area%
51.123	MM	1.4748	139.059	1.5716	4.47
55.737	MM	2.3689	2969.889	20.8952	95.53

Gram-scale experiment:

**Data file:**	C:\CHEM32\1\DATA\AM\DEF_LC 2014-12-12 15-03-19\AM684-2.D		
**Sample name:**	AM684-2		
**Instrument:**	AGILENT 1260	**Acq. operator:**	SYSTEM
**Injection date:**	12/12/2014 3:15:52 PM		
**Acq. method:**	ARB.ADH95B05A.65MI N.1.5ML.10MICROL.M		

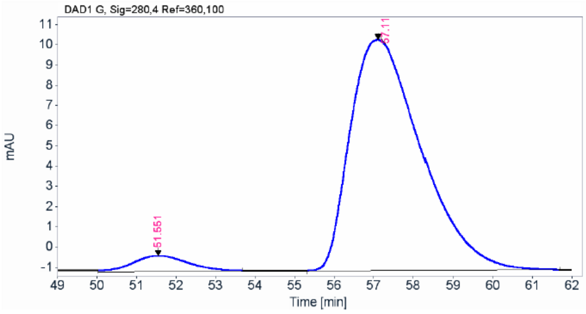

Signal: DAD1 G, Sig=280,4 Ref=360,100

RT [min]	Type	Width [min]	Area	Height	Area%
51.551	MM	1.6188	76.104	0.7835	5.11
57.110	BB	1.4901	1413.736	11.4058	94.89

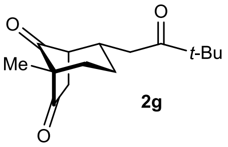

2g

**Data file:**	C:\CHEM32\1\DATA\AMAEL\DEF_LC 2014-09-08 10-50-23\AM669-3.D		
**Sample name:**	AM669-3		
**Instrument:**	AGILENT 1260	**Acq. operator:**	SYSTEM
**Injection date:**	9/8/2014 11:34:20 AM		
**Acq. method:**	ADH85B15A.20MIN.1.0 ML.M		

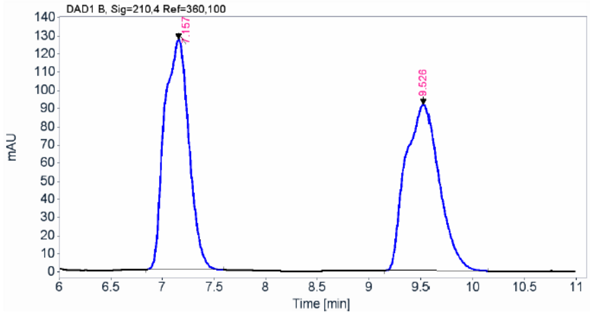

Signal: DAD1 B, Sig=210,4 Ref=360,100

RT [min]	Type	Width [min]	Area	Height	Area%
7.157	MM	0.2735	2083.827	127.0043	50.61
9.526	MM	0.3706	2033.674	91.4678	49.39

**Data file:**	C:\CHEM32\1\DATA\AMAEL\DEF_LC 2014-09-08 10-50-23\AM681-4.D		
**Sample name:**	AM681-4		
**Instrument:**	AGILENT 1260	**Acq. operator:**	SYSTEM
**Injection date:**	9/8/2014 11:02:56 AM		
**Acq. method:**	ADH85B15A.30MIN.1.0 ML.80UL.M		

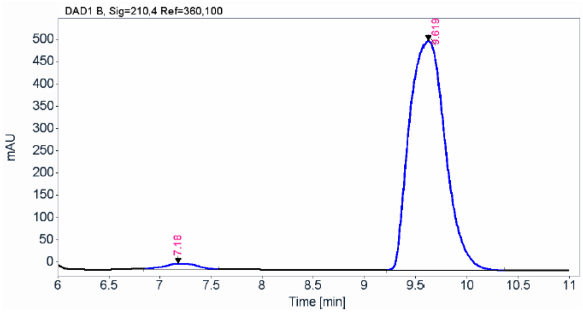

Signal: DAD1 B, Sig=210,4 Ref=360,100

RT [min]	Type	Width [min]	Area	Height	Area%
7.180	MM	0.3866	308.279	13.2885	2.43
9.619	MM	0.3997	12358.928	515.3266	97.57

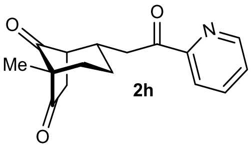

2h

```
data acquired by:   AM
on:                 5/19/2014
location:           Vial 82
```

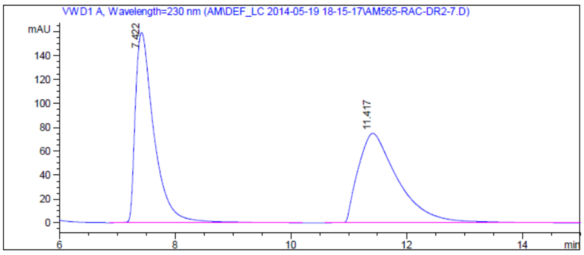

Meas. R	Area %	Width	Symmetr.
7.422	49.869	0.334	0.456
11.417	50.131	0.739	0.525

```
data acquired by:   AM
on:                 5/19/2014
location:           Vial 83
```

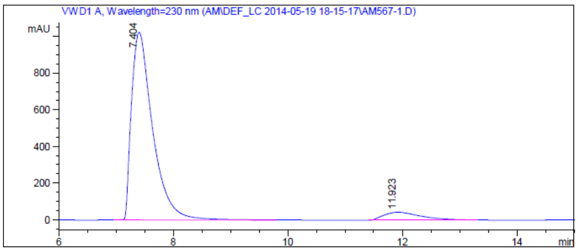

Meas. R	Area %	Width	Symmetr.
7.404	93.350	0.430	0.532
11.923	6.650	0.737	0.585

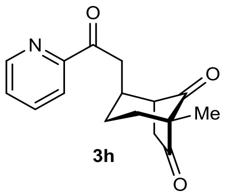

3h

Data file:	C:\CHEM32\1\DATA\AMAEL\DEF_LC 2014-11-24 08-45-52\AM567-EE-MINOR9.D
Sample name:	AM567-ee-minor9
Instrument:	AGILENT 1260
Acq. operator:	SYSTEM
Injection date:	11/24/2014 10:16:29 AM
Acq. method:	ADH90B10A.25MIN.1.0 ML.M

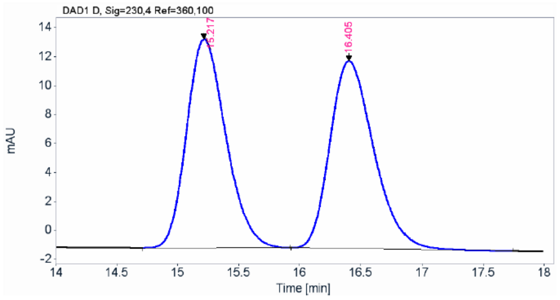

Signal: DAD1 D, Sig=230,4 Ref=360,100

RT [min]	Type	Width [min]	Area	Height	Area%
15.217	BB	0.3478	323.619	14.4225	49.93
16.405	BB	0.3879	324.501	12.9684	50.07

Data file:	C:\CHEM32\1\DATA\AMAEL\DEF_LC 2014-11-24 08-45-52\AM567-EE-MINIOR-1.D
Sample name:	AM567-ee-minior-1
Instrument:	AGILENT 1260
Acq. operator:	SYSTEM
Injection date:	11/24/2014 9:50:31 AM
Acq. method:	ADH90B10A.25MIN.1.0 ML.M

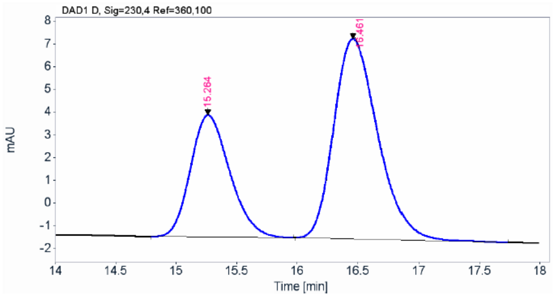

Signal: DAD1 D, Sig=230,4 Ref=360,100

RT [min]	Type	Width [min]	Area	Height	Area%
15.264	BB	0.3477	121.217	5.3633	35.27
16.461	BB	0.3928	222.465	8.8051	64.73

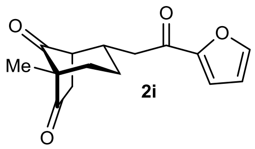

2i

data acquired by: AM
on: 5/21/2014
location: Vial 52

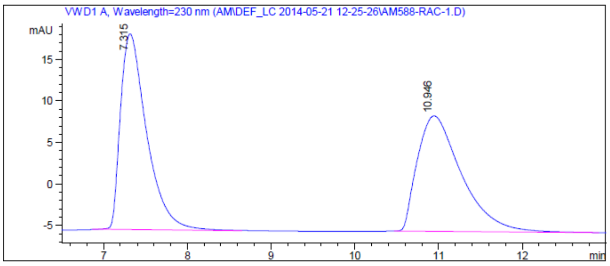

Meas. R	Area %	Width	Symmetr.
7.315	50.239	0.363	0.526
10.946	49.761	0.610	0.566

data acquired by: AM
on: 5/21/2014
location: Vial 56

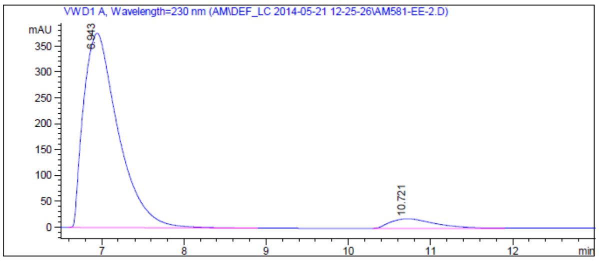

Meas. R	Area %	Width	Symmetr.
6.943	93.898	0.486	0.596
10.721	6.102	0.632	0.604

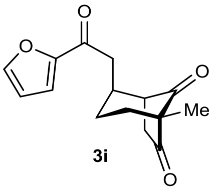

3i

data acquired by: HWL
on: 11/21/2014
location: Vial 63

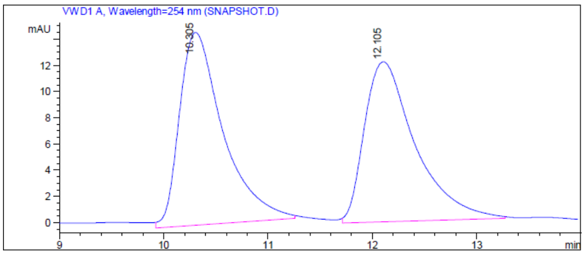

Meas. R	Area %	Width	Symmetr.
10.305	50.785	0.485	0.570
12.105	49.215	0.566	0.573

data acquired by: HWL
on: 11/21/2014
location: Vial 64

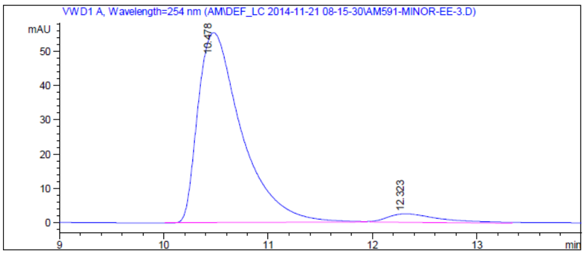

Meas. R	Area %	Width	Symmetr.
10.478	94.780	0.485	0.532
12.323	5.220	0.593	0.481

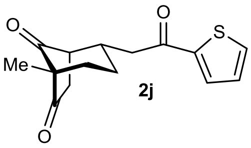

2j

data acquired by: AM
on: 5/22/2014
location: Vial 53

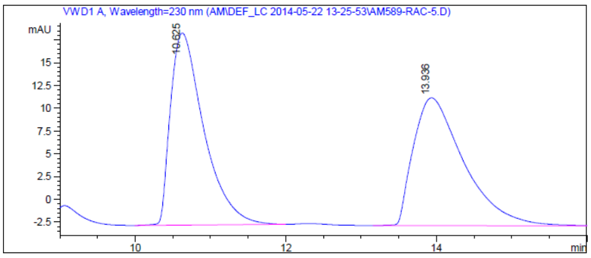

Meas. R	Area %	Width	Symmetr.
10.625	49.780	0.475	0.509
13.936	50.220	0.723	0.524

data acquired by: AM
on: 5/22/2014
location: Vial 63

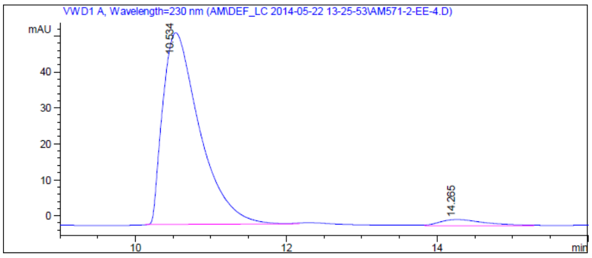

Meas. R	Area %	Width	Symmetr.
10.534	96.097	0.566	0.518
14.265	3.903	0.705	0.579

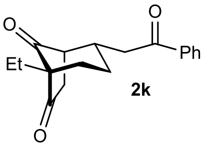

2k

data acquired by: AM
on: 4/14/2014
location: Vial 6

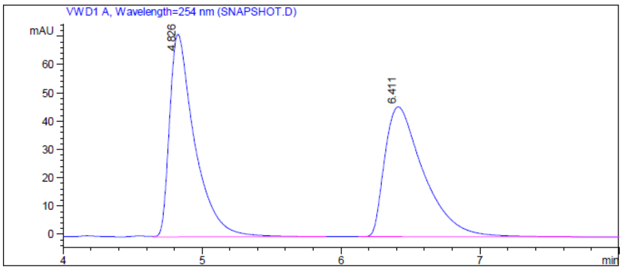

data acquired by: AM
on: 4/14/2014
location: Vial 7

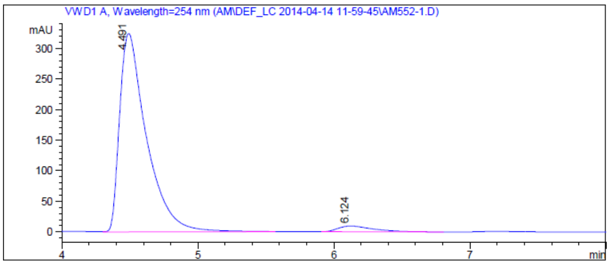

Meas. R	Area %	Width	Symmetr.
4.826	49.898	0.183	0.457
6.411	50.102	0.295	0.482

Meas. R	Area %	Width	Symmetr.
4.491	96.300	0.228	0.482
6.124	3.700	0.302	0.537

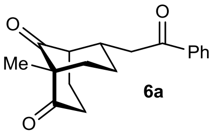

6a

data acquired by: ARB
on: 7/24/2014
location: Vial 30

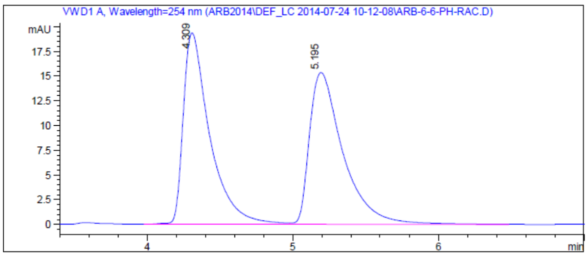

data acquired by: ARB
on: 4/3/2014
location: Vial 13

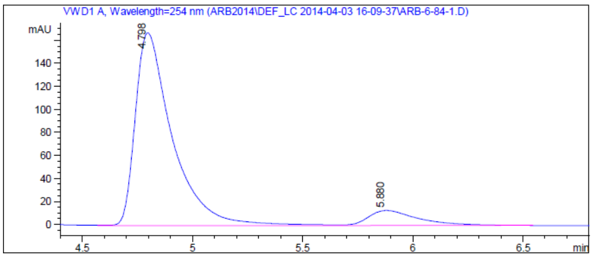

Meas. R	Area %	Width	Symmetr.
4.309	49.818	0.210	0.512
5.195	50.182	0.267	0.492

Meas. R	Area %	Width	Symmetr.
4.798	90.709	0.194	0.483
5.880	9.291	0.257	0.530

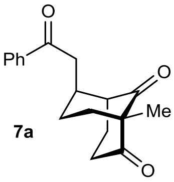

data acquired by: AM
on: 10/20/2014
location: Vial 32

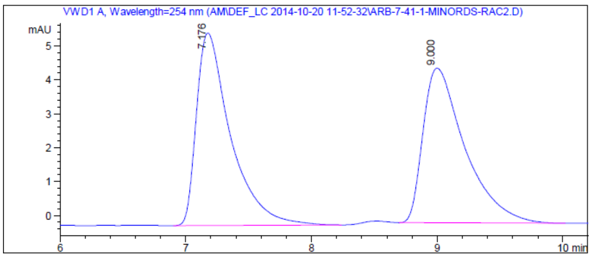

Meas. R	Area %	Width	Symmetr.
7.176	50.300	0.278	0.458
9.000	49.700	0.344	0.469

data acquired by: HWL
on: 10/21/2014
location: Vial 13

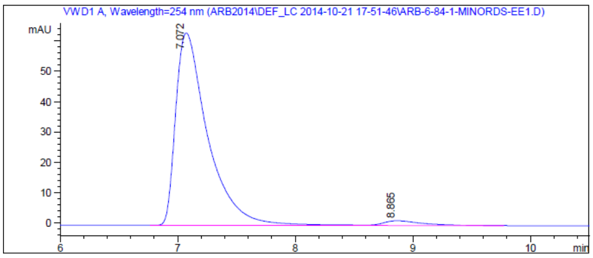

Meas. R	Area %	Width	Symmetr.
7.072	96.837	0.317	0.469
8.865	3.163	0.405	0.501

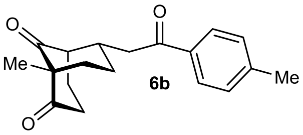

data acquired by: ARB
on: 2/12/2014
location: Vial 14

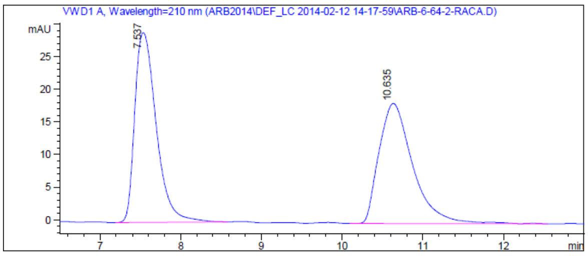

Meas. R	Area %	Width	Symmetr.
7.537	50.050	0.288	0.611
10.635	49.950	0.465	0.676

data acquired by: ARB
on: 4/7/2014
location: Vial 9

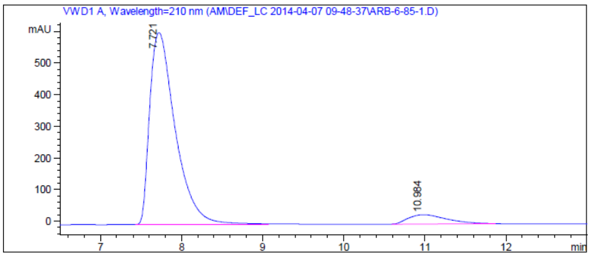

Meas. R	Area %	Width	Symmetr.
7.721	93.242	0.366	0.567
10.984	6.758	0.546	0.640

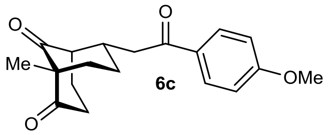

data acquired by: AM
on: 4/14/2014
location: Vial 11

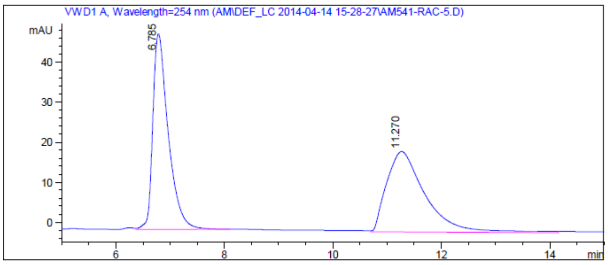

Meas. R	Area %	Width	Symmetr.
6.785	49.464	0.295	0.542
11.270	50.536	0.821	0.610

data acquired by: AM
on: 4/14/2014
location: Vial 12

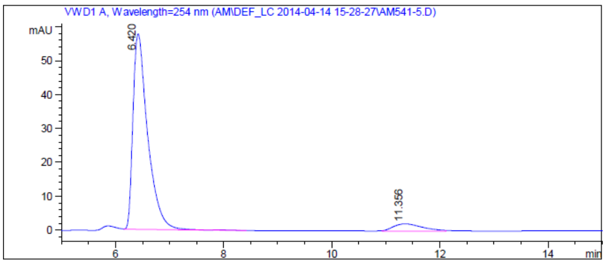

Meas. R	Area %	Width	Symmetr.
6.420	93.398	0.316	0.509
11.356	6.602	0.616	0.649

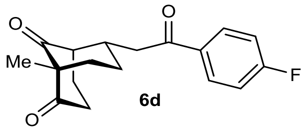

data acquired by: AM
on: 7/14/2014
location: Vial 7

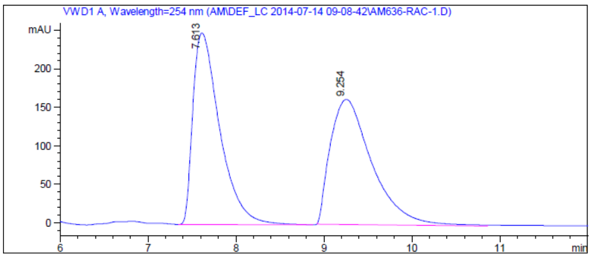

Meas. R	Area %	Width	Symmetr.
7.613	49.738	0.325	0.467
9.254	50.262	0.549	0.594

data acquired by: AM
on: 7/17/2014
location: Vial 77

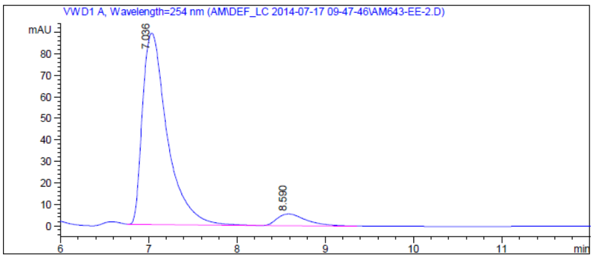

Meas. R	Area %	Width	Symmetr.
7.036	93.221	0.326	0.558
8.590	6.779	0.385	0.597

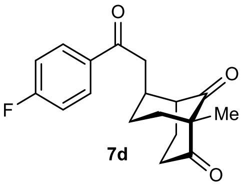

7d

```
data acquired by:   HWL
on:                 11/14/2014
location:           Vial 52
```

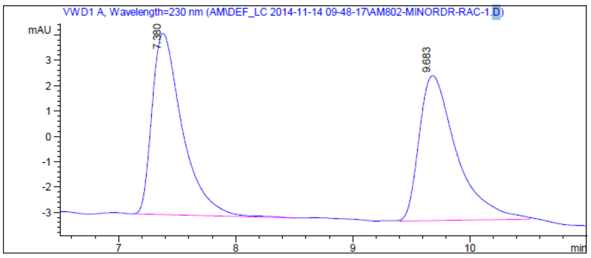

Meas. R	Area %	Width	Symmetr.
7.380	50.254	0.269	0.520
9.683	49.746	0.370	0.541

```
data acquired by:   HWL
on:                 11/14/2014
location:           Vial 53
```

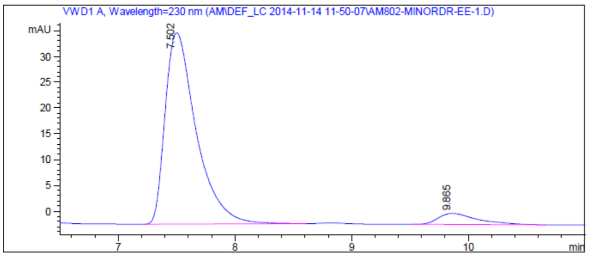

Meas. R	Area %	Width	Symmetr.
7.502	93.222	0.280	0.543
9.865	6.778	0.348	0.529

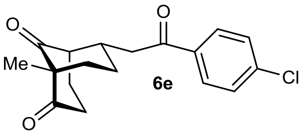

6e

```
data acquired by:   ARB
on:                 2/4/2014
location:           Vial 6
```

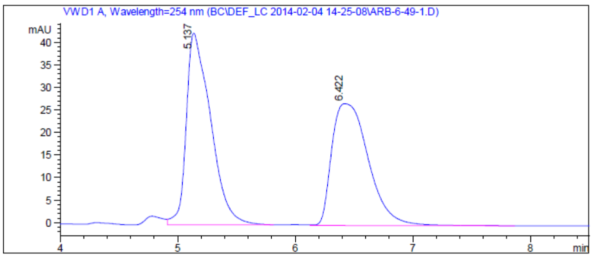

Meas. R	Area %	Width	Symmetr.
5.137	51.169	0.240	0.427
6.422	48.831	0.361	0.517

```
data acquired by:   ARB
on:                 4/9/2014
location:           Vial 96
```

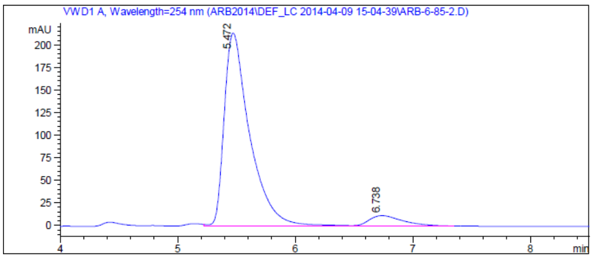

Meas. R	Area %	Width	Symmetr.
5.472	93.389	0.251	0.000
6.738	6.611	0.330	0.622

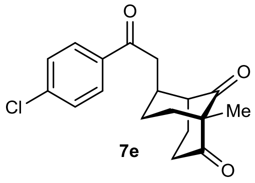

data acquired by:	HWL
on:	10/21/2014
location:	Vial 22

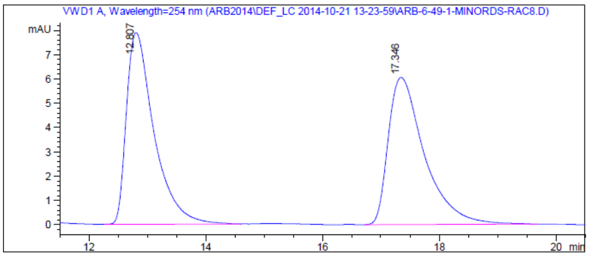

Meas. R	Area %	Width	Symmetr.
12.807	49.848	0.490	0.497
17.346	50.152	0.644	0.504

data acquired by:	HWL
on:	10/22/2014
location:	Vial 25

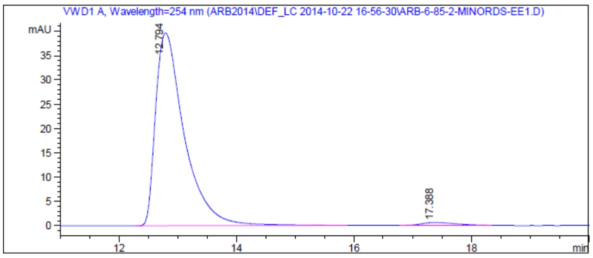

Meas. R	Area %	Width	Symmetr.
12.794	98.146	0.512	0.479
17.388	1.854	0.679	0.628

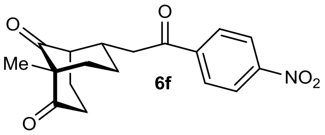

data acquired by:	ARB
on:	7/24/2014
location:	Vial 29

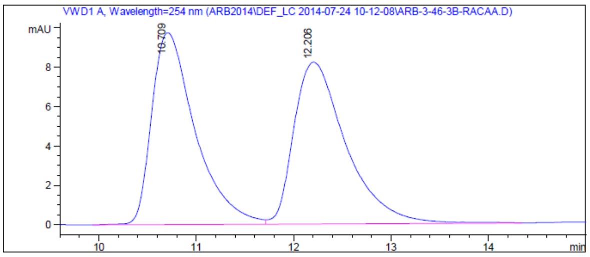

Meas. R	Area %	Width	Symmetr.
10.709	49.666	0.466	0.546
12.206	50.334	0.567	0.565

data acquired by:	ARB
on:	4/10/2014
location:	Vial 7

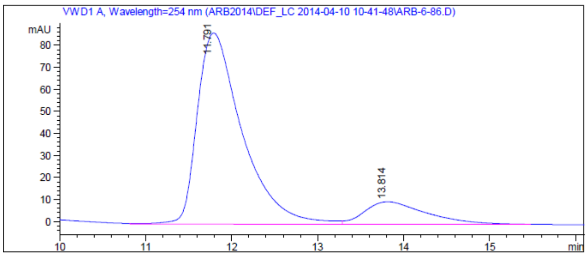

Meas. R	Area %	Width	Symmetr.
11.791	86.037	0.608	0.000
13.814	13.963	0.835	0.583

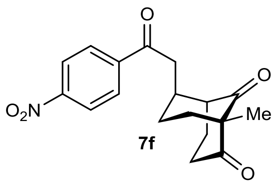

data acquired by: AM
on: 10/20/2014
location: Vial 33

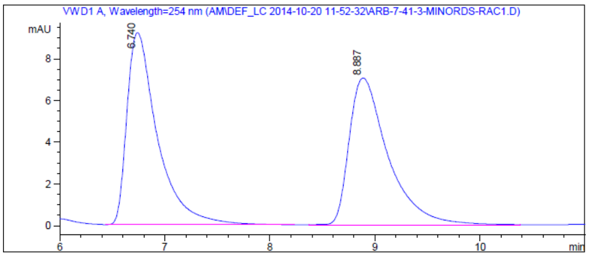

Meas. R	Area %	Width	Symmetr.
6.740	50.587	0.300	0.478
8.887	49.413	0.431	0.533

data acquired by: HWL
on: 10/21/2014
location: Vial 15

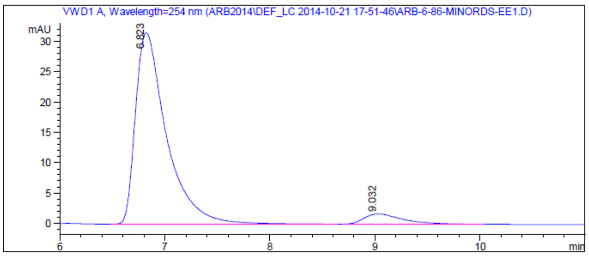

Meas. R	Area %	Width	Symmetr.
6.823	93.851	0.306	0.483
9.032	6.149	0.424	0.560

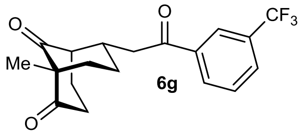

data acquired by: ARB
on: 2/4/2014
location: Vial 5

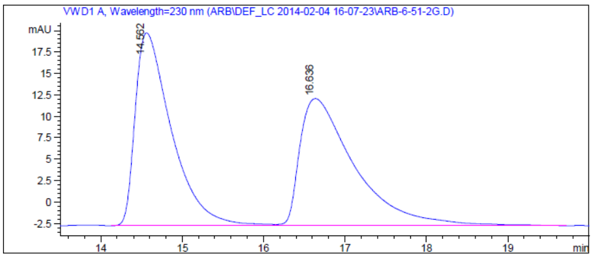

Meas. R	Area %	Width	Symmetr.
14.562	50.073	0.465	0.421
16.636	49.927	0.699	0.357

data acquired by: ARB
on: 4/28/2014
location: Vial 43

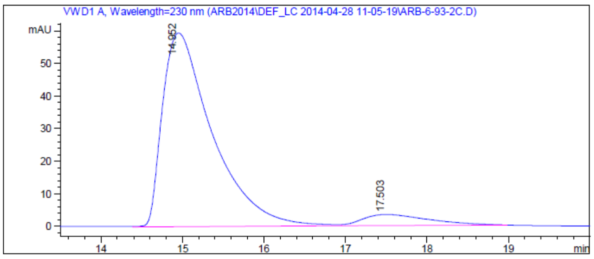

Meas. R	Area %	Width	Symmetr.
14.952	92.927	0.710	0.000
17.503	7.073	0.941	0.543

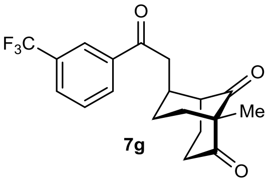

7g

data acquired by:	AM	
on:	10/20/2014	
location:	Vial 35	

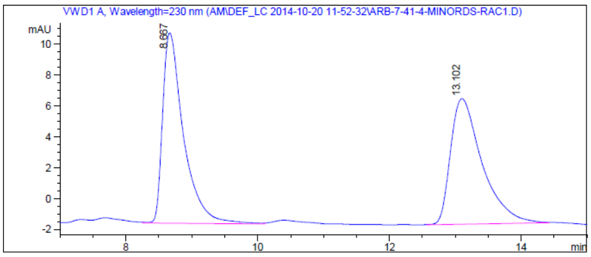

Meas. R	Area %	Width	Symmetr.
8.667	50.143	0.368	0.498
13.102	49.857	0.555	0.549

data acquired by:	HWL	
on:	10/22/2014	
location:	Vial 24	

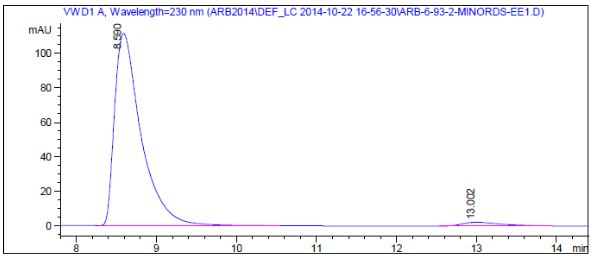

Meas. R	Area %	Width	Symmetr.
8.590	97.108	0.345	0.465
13.002	2.892	0.569	0.536

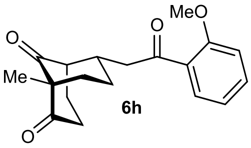

6h

data acquired by:	AM	
on:	4/17/2014	
location:	Vial 32	

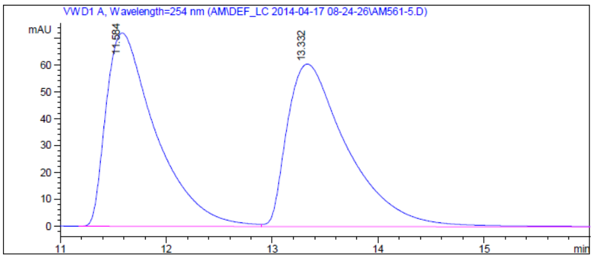

Meas. R	Area %	Width	Symmetr.
11.584	49.464	0.485	0.437
13.332	50.536	0.597	0.455

data acquired by:	AM	
on:	4/17/2014	
location:	Vial 31	

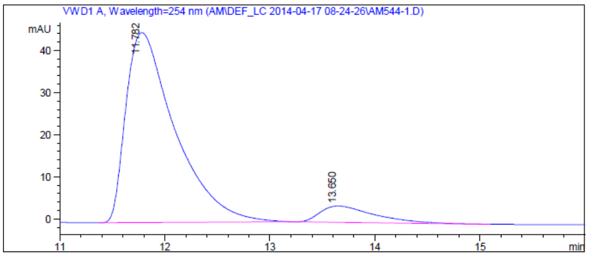

Meas. R	Area %	Width	Symmetr.
11.782	91.349	0.483	0.445
13.650	8.651	0.528	0.462

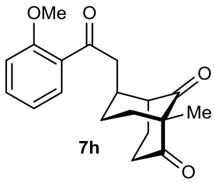

7h

data acquired by: AM
on: 4/17/2014
location: Vial 32

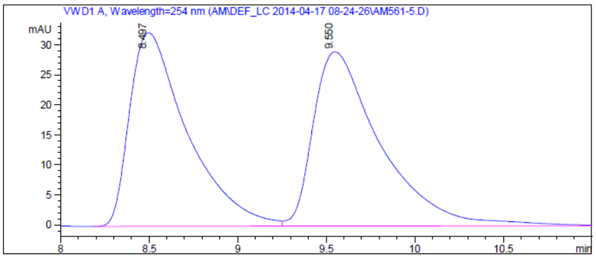

Meas. R	Area %	Width	Symmetr.
8.497	48.716	0.334	0.451
9.550	51.284	0.388	0.442

data acquired by: AM
on: 4/17/2014
location: Vial 31

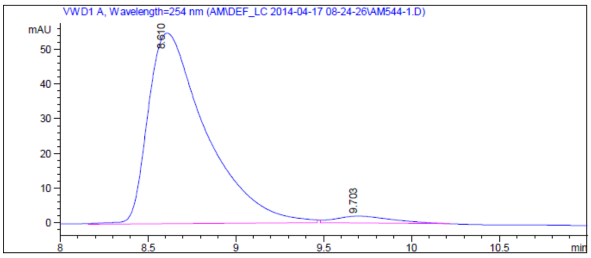

Meas. R	Area %	Width	Symmetr.
8.610	96.626	0.382	0.476
9.703	3.374	0.372	0.629

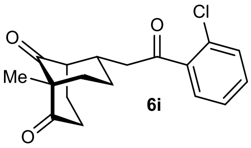

6i

data acquired by: bp
on: 2/11/2014
location: Vial 2

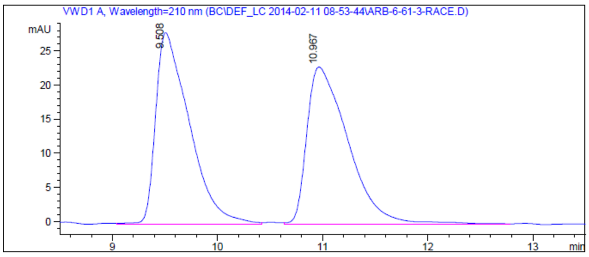

Meas. R	Area %	Width	Symmetr.
9.508	49.728	0.311	0.404
10.967	50.272	0.439	0.393

data acquired by: ARB
on: 4/11/2014
location: Vial 1

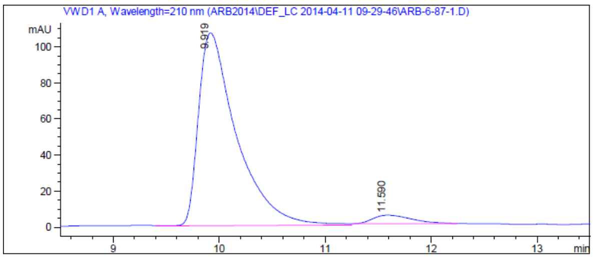

Meas. R	Area %	Width	Symmetr.
9.919	95.755	0.416	0.449
11.590	4.245	0.423	0.580

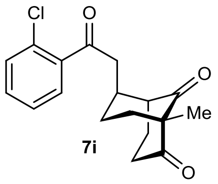

7i

data acquired by: HWL
on: 10/21/2014
location: Vial 36

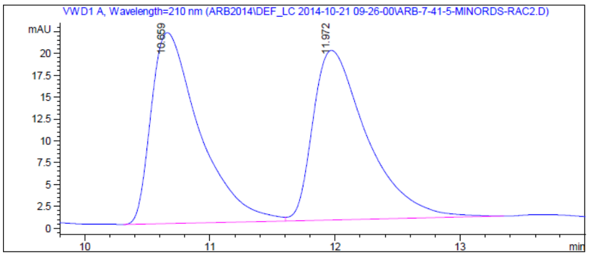

Meas. R	Area %	Width	Symmetr.
10.659	50.163	0.402	0.464
11.972	49.837	0.450	0.486

data acquired by: HWL
on: 10/22/2014
location: Vial 23

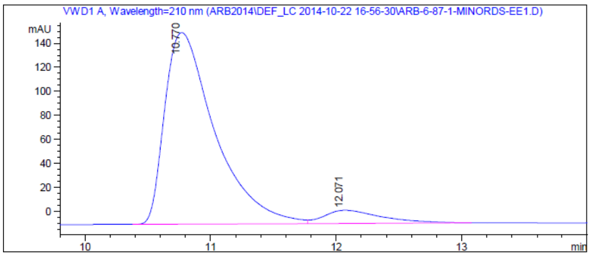

Meas. R	Area %	Width	Symmetr.
10.770	92.735	0.419	0.470
12.071	7.265	0.469	0.534

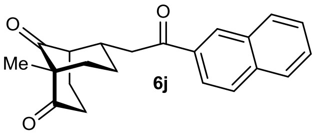

6j

data acquired by: ARB
on: 2/21/2014
location: Vial 54

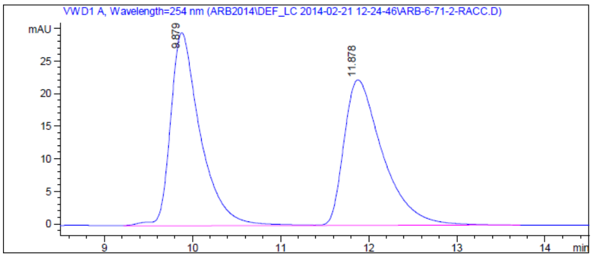

Meas. R	Area %	Width	Symmetr.
9.879	50.558	0.391	0.580
11.878	49.442	0.508	0.541

data acquired by: AM
on: 4/14/2014
location: Vial 14

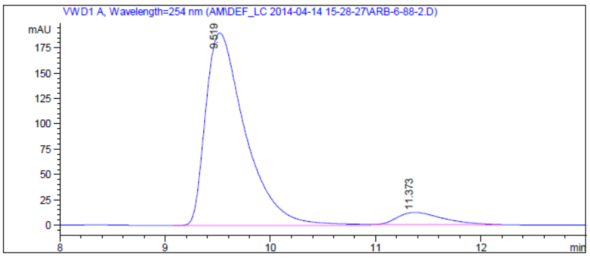

Meas. R	Area %	Width	Symmetr.
9.519	93.289	0.394	0.506
11.373	6.711	0.497	0.604

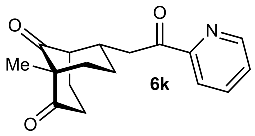

6k

data acquired by: ARB
on: 4/29/2014
location: Vial 44

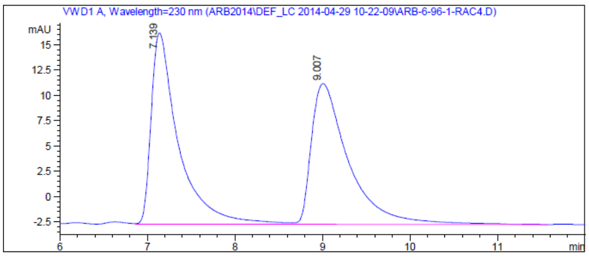

data acquired by: AM
on: 5/25/2014
location: Vial 93

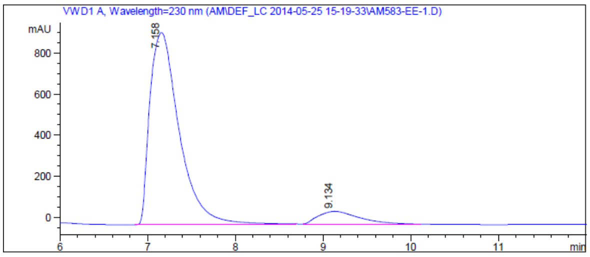

Meas. R	Area %	Width	Symmetr.
7.139	49.956	0.311	0.394
9.007	50.044	0.435	0.414

Meas. R	Area %	Width	Symmetr.
7.158	91.033	0.388	0.607
9.134	8.967	0.557	0.646

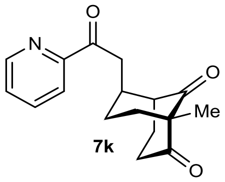

7k

Data file: C:\CHEM32\1\DATA\AMAEL\DEF_LC 2014-11-24 08-45-52\AM583-RAC-MINOR-9.D
Sample name: AM583-rac-minor-9
Instrument: AGILENT 1260 **Acq. operator:** SYSTEM
Injection date: 11/24/2014 9:24:36 AM
Acq. method: ADH90B10A.25MIN.1.0 ML.M

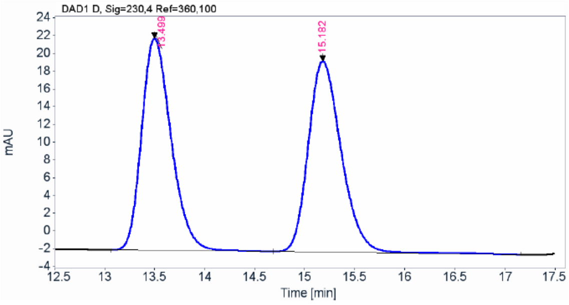

Signal: DAD1 D, Sig=230,4 Ref=360,100

RT [min]	Type	Width [min]	Area	Height	Area%
13.499	BB	0.3171	489.488	23.8868	49.78
15.182	BB	0.3540	493.734	21.4921	50.22

Data file: C:\CHEM32\1\DATA\AMAEL\DEF_LC 2014-11-24 08-45-52\AM583-EE-MINOR-1.D
Sample name: AM583-ee-minor-1
Instrument: AGILENT 1260 **Acq. operator:** SYSTEM
Injection date: 11/24/2014 8:58:40 AM
Acq. method: ADH90B10A.25MIN.1.0 ML.M

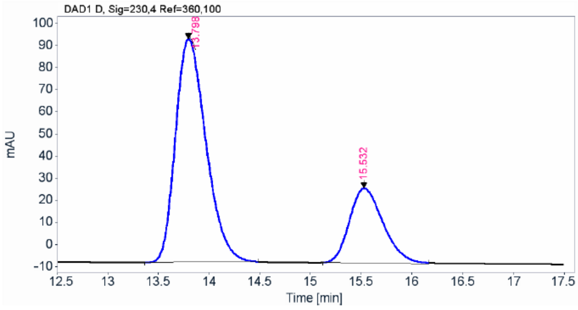

Signal: DAD1 D, Sig=230,4 Ref=360,100

RT [min]	Type	Width [min]	Area	Height	Area%
13.798	MM	0.3477	2104.664	100.8995	72.83
15.532	MM	0.3867	785.140	33.8405	27.17

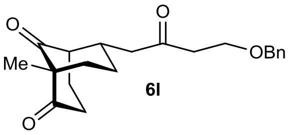

6l

Data file: C:\CHEM32\1\DATA\ARB2014\DEF_LC 2014-09-29 14-25-14\ARB-7-29-3-II-C-RACAA.D
Sample name: arb-7-29-3-ii-c-racAA
Instrument: AGILENT 1260 Acq. operator: SYSTEM
Injection date: 9/29/2014 3:39:44 PM
Acq. method: ARB.ADH90B10A.20MIN.1.5ML.20UL.M

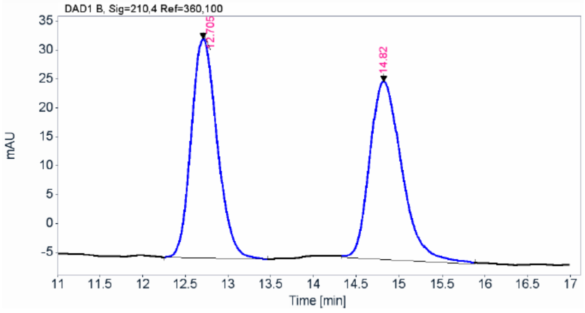

Signal: DAD1 B, Sig=210,4 Ref=360,100

RT [min]	Type	Width [min]	Area	Height	Area%
12.705	BB	0.3263	800.294	37.9107	49.65
14.820	MM	0.4387	811.700	30.8350	50.35

Data file: C:\CHEM32\1\DATA\ARB2014\DEF_LC 2014-09-29 14-25-14\ARB-7-29-1-II-F-EE11.D
Sample name: arb-7-29-1-ii-f-ee11
Instrument: AGILENT 1260 Acq. operator: SYSTEM
Injection date: 9/29/2014 4:21:57 PM
Acq. method: ARB.ADH90B10A.20MIN.1.5ML.20UL.M

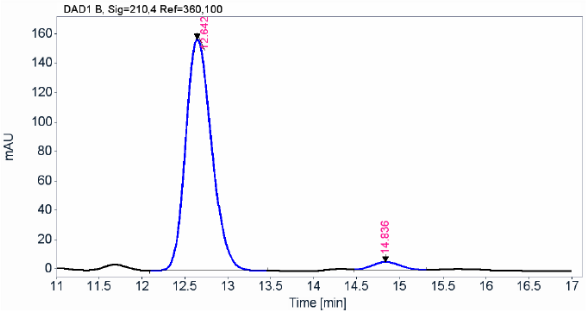

Signal: DAD1 B, Sig=210,4 Ref=360,100

RT [min]	Type	Width [min]	Area	Height	Area%
12.642	BB	0.3276	3311.187	157.3093	96.27
14.836	MM	0.3839	128.420	5.5751	3.73

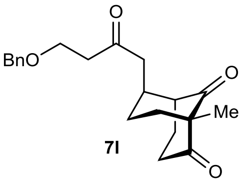

7l

Data file: C:\CHEM32\1\DATA\ARB2014\DEF_LC 2014-09-29 14-25-14\ARB-7-29-3-II-B-RACAB.D
Sample name: arb-7-29-3-ii-b-racAB
Instrument: AGILENT 1260 Acq. operator: SYSTEM
Injection date: 9/29/2014 5:45:56 PM
Acq. method: ARB.ADH90B10A.20MIN.1.5ML.20UL.M

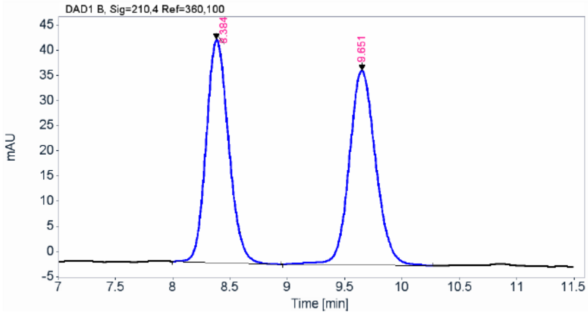

Signal: DAD1 B, Sig=210,4 Ref=360,100

RT [min]	Type	Width [min]	Area	Height	Area%
8.384	BB	0.2104	601.285	44.2806	49.18
9.651	BB	0.2477	621.287	38.6175	50.82

Data file: C:\CHEM32\1\DATA\ARB2014\DEF_LC 2014-09-29 14-25-14\ARB-7-29-1-II-D-EE13.D
Sample name: arb-7-29-1-ii-d-ee13
Instrument: AGILENT 1260 Acq. operator: SYSTEM
Injection date: 9/29/2014 6:28:09 PM
Acq. method: ARB.ADH90B10A.20MIN.1.5ML.20UL.M

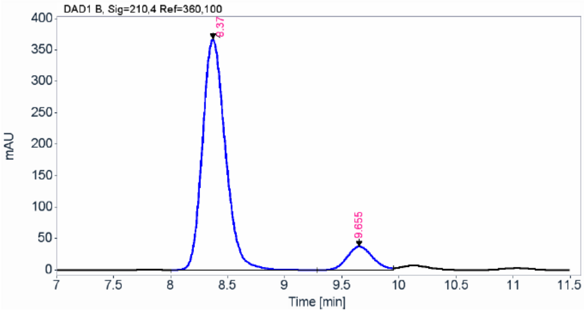

Signal: DAD1 B, Sig=210,4 Ref=360,100

RT [min]	Type	Width [min]	Area	Height	Area%
8.370	BB	0.2169	5119.811	366.6863	89.79
9.655	BV	0.2414	582.450	37.4544	10.21

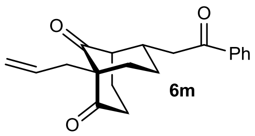

6m

```
data acquired by:   ARB
on:                 2/17/2014
location:           Vial 23
```

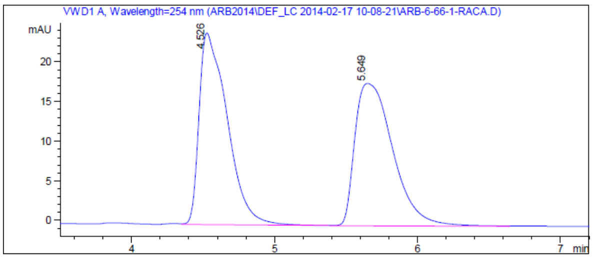


```
data acquired by:   AM
on:                 4/14/2014
location:           Vial 13
```

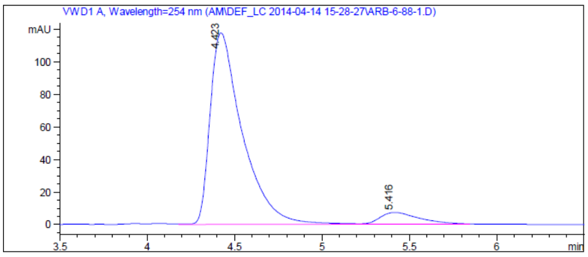

Meas. R	Area %	Width	Symmetr.
4.526	49.894	0.186	0.370
5.649	50.106	0.309	0.471

Meas. R	Area %	Width	Symmetr.
4.423	93.143	0.214	0.000
5.416	6.857	0.257	0.570

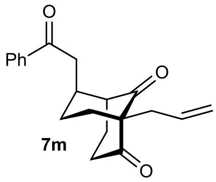

7m

```
data acquired by:   AM
on:                 10/20/2014
location:           Vial 34
```

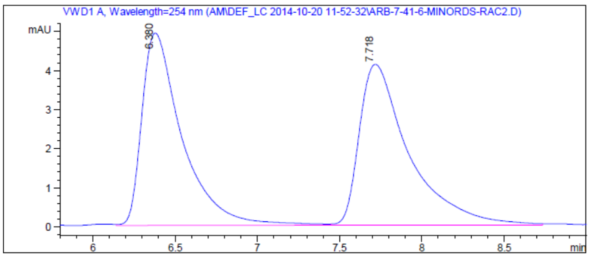


```
data acquired by:   HWL
on:                 10/21/2014
location:           Vial 14
```

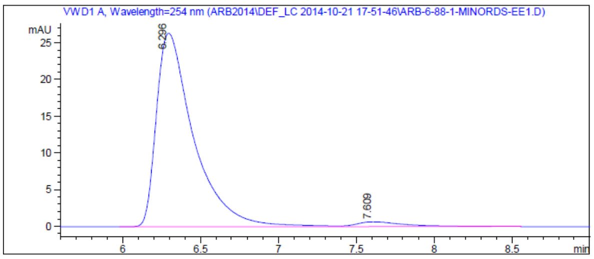

Meas. R	Area %	Width	Symmetr.
6.380	49.152	0.283	0.000
7.718	50.848	0.351	0.459

Meas. R	Area %	Width	Symmetr.
6.296	97.044	0.277	0.000
7.609	2.956	0.340	0.506

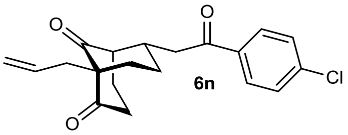

Data file: C:\CHEM32\1\DATA\AMAEL\DEF_LC 2014-05-30 11-40-50\AM593-RAC-1.D
Sample name: AM593-rac-1
Instrument: AGILENT 1260 Acq. operator: SYSTEM
Injection date: 5/30/2014 11:57:58 AM
Acq. method: IC90B10D.30MIN.1.0M L.M

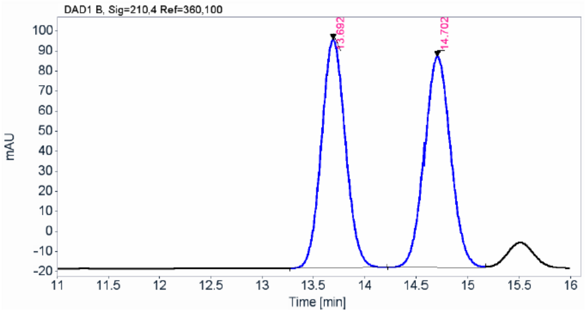

Signal: DAD1 B, Sig=210,4 Ref=360,100

RT [min]	Type	Width [min]	Area	Height	Area%
13.692	BB	0.2541	1852.090	113.6641	49.99
14.702	BV	0.2736	1852.469	105.1156	50.01

Data file: C:\CHEM32\1\DATA\AMAEL\DEF_LC 2014-05-30 11-40-50\AM594-EE-1.D
Sample name: AM594-ee-1
Instrument: AGILENT 1260 Acq. operator: SYSTEM
Injection date: 5/30/2014 12:28:55 PM
Acq. method: IC90B10D.30MIN.1.0M L.M

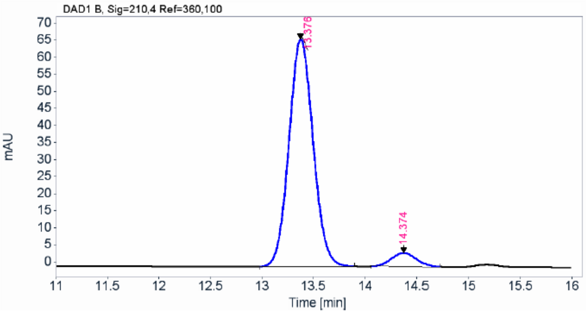

Signal: DAD1 B, Sig=210,4 Ref=360,100

RT [min]	Type	Width [min]	Area	Height	Area%
13.376	MM	0.2721	1086.653	66.5681	94.26
14.374	MM	0.2786	66.140	3.9567	5.74

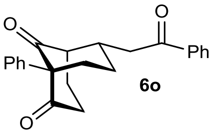

Data file: C:\CHEM32\1\DATA\AMAEL\DEF_LC 2014-07-10 20-04-44\AM614-8.D
Sample name: AM614-8
Instrument: AGILENT 1260 Acq. operator: SYSTEM
Injection date: 7/10/2014 8:48:48 PM
Acq. method: ADH80B20A.30MIN.1.5 ML.50UL.M

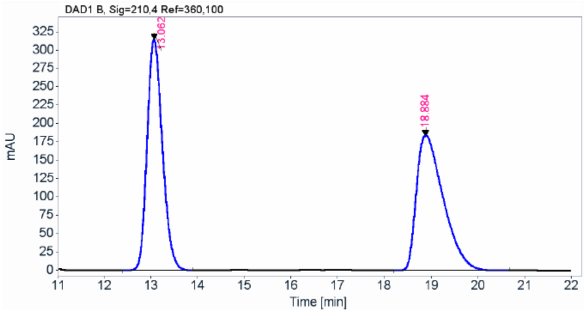

Signal: DAD1 B, Sig=210,4 Ref=360,100

RT [min]	Type	Width [min]	Area	Height	Area%
13.062	BB	0.3520	7165.322	314.2134	49.96
18.884	BB	0.5852	7177.208	183.9321	50.04

Data file: C:\CHEM32\1\DATA\AMAEL\DEF_LC 2014-07-10 20-04-44\AM626-1.D
Sample name: AM626-1
Instrument: AGILENT 1260 Acq. operator: SYSTEM
Injection date: 7/10/2014 8:17:21 PM
Acq. method: ADH80B20A.30MIN.1.5 ML.50UL.M

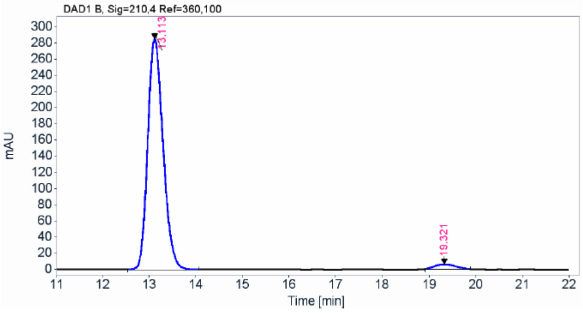

Signal: DAD1 B, Sig=210,4 Ref=360,100

RT [min]	Type	Width [min]	Area	Height	Area%
13.113	MM	0.3834	6559.286	285.1218	96.94
19.321	MM	0.5284	207.225	6.5368	3.06

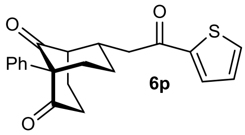

Data file: C:\CHEM32\1\DATA\AMAEL\DEF_LC 2014-06-06 14-22-50\AM601-RAC-1.D
Sample name: AM601-rac-1
Instrument: AGILENT 1260 **Acq. operator:** SYSTEM
Injection date: 6/6/2014 2:30:08 PM
Acq. method: ADH75B25A.25MIN.1.5 ML.30UL.M

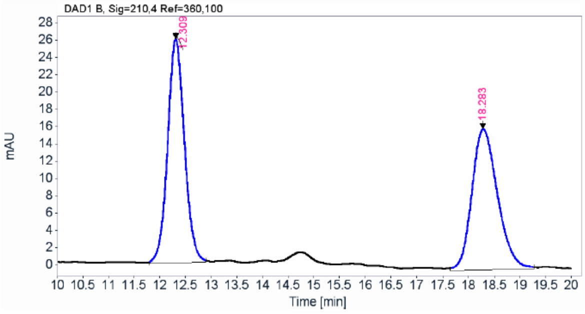

Signal: DAD1 B, Sig=210,4 Ref=360,100

RT [min]	Type	Width [min]	Area	Height	Area%
12.309	MM	0.3793	589.345	25.8947	50.93
18.283	MM	0.5808	567.934	16.2982	49.07

Data file: C:\CHEM32\1\DATA\AMAEL\DEF_LC 2014-06-06 14-22-50\AM601-EE-1.D
Sample name: AM601-ee-1
Instrument: AGILENT 1260 **Acq. operator:** SYSTEM
Injection date: 6/6/2014 3:12:15 PM
Acq. method: ADH75B25A.25MIN.1.5 ML.30UL.M

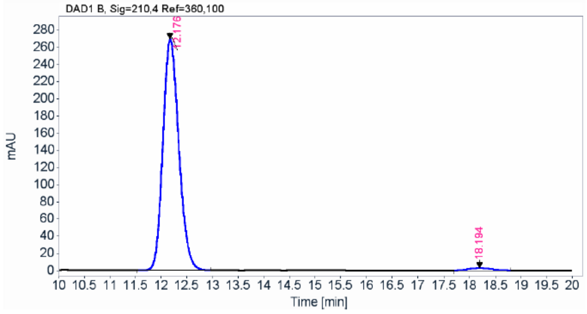

Signal: DAD1 B, Sig=210,4 Ref=360,100

RT [min]	Type	Width [min]	Area	Height	Area%
12.176	BB	0.3330	5792.730	269.2108	98.40
18.194	MM	0.5378	94.238	2.9205	1.60

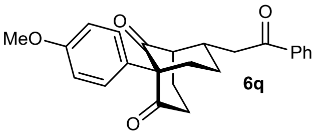

Data file: C:\CHEM32\1\DATA\AMAEL\DEF_LC 2014-07-24 10-56-47\AM644-RAC-2.D
Sample name: AM644-rac-2
Instrument: AGILENT 1260 **Acq. operator:** SYSTEM
Injection date: 7/24/2014 11:51:29 AM
Acq. method: ADH75B25A.45MIN.1.5 ML.80UL.M

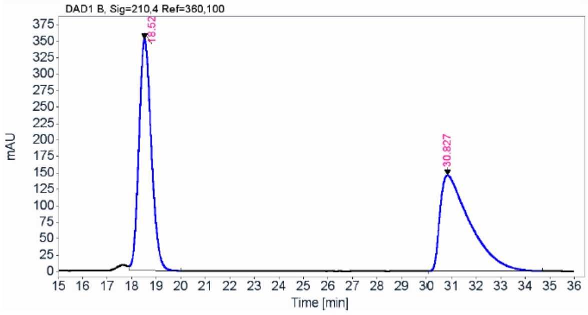

Signal: DAD1 B, Sig=210,4 Ref=360,100

RT [min]	Type	Width [min]	Area	Height	Area%
18.520	VB	0.5486	12373.861	351.6024	50.01
30.827	BB	1.2109	12367.503	145.9034	49.99

Data file: C:\CHEM32\1\DATA\AMAEL\DEF_LC 2014-07-24 10-56-47\AM651-EE-1.D
Sample name: AM651-ee-1
Instrument: AGILENT 1260 **Acq. operator:** SYSTEM
Injection date: 7/24/2014 11:04:41 AM
Acq. method: ADH75B25A.45MIN.1.5 ML.80UL.M

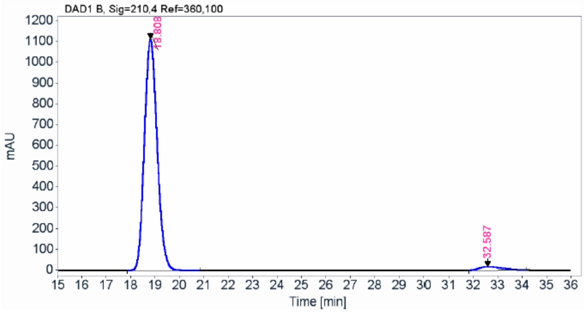

Signal: DAD1 B, Sig=210,4 Ref=360,100

RT [min]	Type	Width [min]	Area	Height	Area%
18.808	BB	0.5680	40431.086	1112.3634	97.05
32.587	MM	1.1097	1230.076	18.4748	2.95

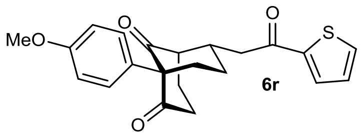

6r

Data file: C:\CHEM32\1\DATA\AMAEL\DEF_LC 2014-07-11 16-55-38\AM633-RAC-3.D
Sample name: AM633-rac-3
Instrument: AGILENT 1260 **Acq. operator:** SYSTEM
Injection date: 7/11/2014 5:45:46 PM
Acq. method: ADH75B25A.45MIN.1.5
ML.80UL.M

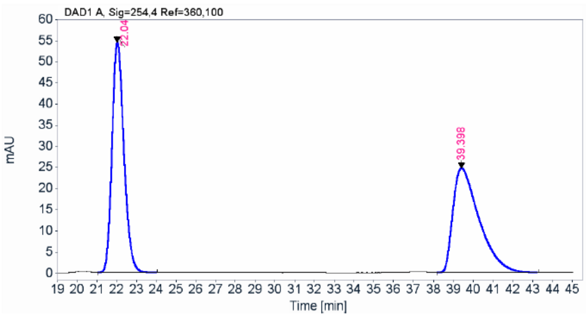

Signal: DAD1 A, Sig=254,4 Ref=360,100

RT [min]	Type	Width [min]	Area	Height	Area%
22.040	BB	0.6455	2270.964	54.3501	50.09
39.398	BB	1.3229	2263.209	24.6101	49.91

Data file: C:\CHEM32\1\DATA\AMAEL\DEF_LC 2014-07-16 21-07-04\AM641-EE-1.D
Sample name: AM641-ee-1
Instrument: AGILENT 1260 **Acq. operator:** SYSTEM
Injection date: 7/16/2014 9:14:35 PM
Acq. method: ADH75B25A.45MIN.1.5
ML.80UL.M

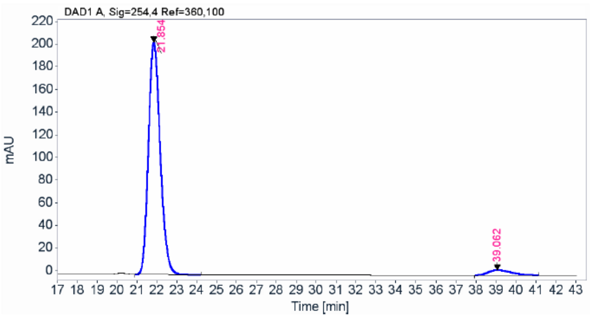

Signal: DAD1 A, Sig=254,4 Ref=360,100

RT [min]	Type	Width [min]	Area	Height	Area%
21.854	BB	0.6371	8413.978	204.9108	95.82
39.062	MM	1.3600	367.122	4.4991	4.18

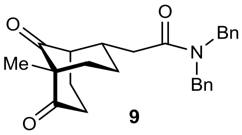

9

```
data acquired by:   ARB
on:                 9/8/2014
location:           Vial 3
```

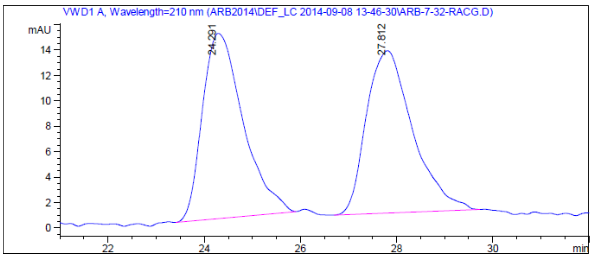

Meas. R	Area %	Width	Symmetr.
24.291	50.071	0.888	0.610
27.812	49.929	1.010	0.775

```
data acquired by:   ARB
on:                 9/8/2014
location:           Vial 6
```

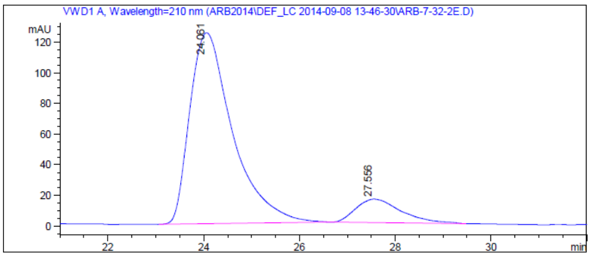

Meas. R	Area %	Width	Symmetr.
24.061	88.447	0.940	0.598
27.556	11.553	1.023	0.623

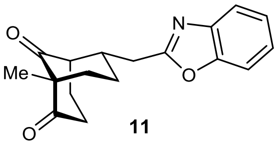

11

Data file:	C:\CHEM32\1\DATA\BC\DEF_LC 2014-06-03 21-22-18\AM604-RAC-1.D
Sample name:	AM604-rac-1
Instrument:	AGILENT 1260
Acq. operator:	SYSTEM
Injection date:	6/3/2014 11:27:12 PM
Acq. method:	ADH95B05A.50MIN.0.8 ML.M

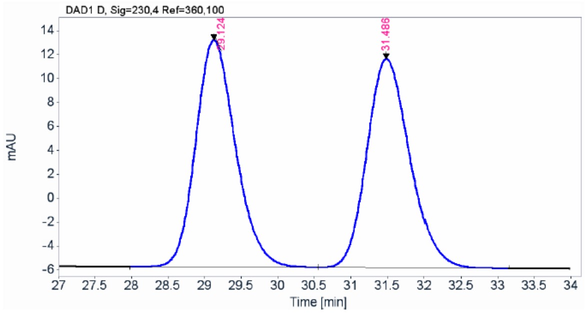

Signal: DAD1 D, Sig=230,4 Ref=360,100

RT [min]	Type	Width [min]	Area	Height	Area%
29.124	BB	0.5806	720.828	18.9985	50.34
31.486	BB	0.6287	711.083	17.4771	49.66

Data file:	C:\CHEM32\1\DATA\BC\DEF_LC 2014-06-03 21-22-18\AM604-EE-1.D
Sample name:	AM604-ee-1
Instrument:	AGILENT 1260
Acq. operator:	SYSTEM
Injection date:	6/4/2014 12:18:09 AM
Acq. method:	ADH95B05A.50MIN.0.8 ML.M

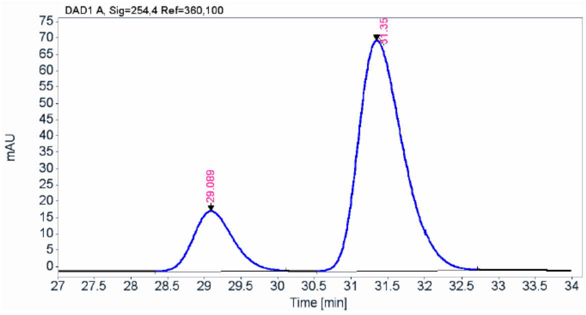

Signal: DAD1 A, Sig=254,4 Ref=360,100

RT [min]	Type	Width [min]	Area	Height	Area%
29.089	MM	0.6393	711.501	18.5485	19.18
31.350	MM	0.7070	2997.518	70.6627	80.82
